# *Chlamydia pneumoniae* Is Genetically Diverse in Animals and Appears to Have Crossed the Host Barrier to Humans on (At Least) Two Occasions

**DOI:** 10.1371/journal.ppat.1000903

**Published:** 2010-05-20

**Authors:** Candice M. Mitchell, Susan Hutton, Garry S. A. Myers, Robert Brunham, Peter Timms

**Affiliations:** 1 Institute of Health and Biomedical Innovation, School of Life Sciences, Queensland University of Technology, Kelvin Grove, Queensland, Australia; 2 Menzies School of Health Research, Royal Darwin Hospital Campus, Casuarina, Northern Territory, Australia; 3 Institute for Genome Sciences, University of Maryland, Baltimore, Maryland, United States of America; 4 British Columbia Centre for Disease Control, University of British Columbia, Vancouver, British Columbia, Canada; SUNY at Stony Brook, United States of America

## Abstract

*Chlamydia pneumoniae* is a common human and animal pathogen associated with a wide range of diseases. Since the first isolation of *C. pneumoniae* TWAR in 1965, all human isolates have been essentially clonal, providing little evolutionary insight. To address this gap, we investigated the genetic diversity of 30 isolates from diverse geographical locations, from both human and animal origin (amphibian, reptilian, equine and marsupial). Based on the level of variation that we observed at 23 discreet gene loci, it was clearly evident that the animal isolates were more diverse than the isolates of human origin. Furthermore, we show that *C. pneumoniae* isolates could be grouped into five major genotypes, A-E, with A, B, D and E genotypes linked by geographical location, whereas genotype C was found across multiple continents. Our evidence strongly supports two separate animal-to-human cross species transfer events in the evolutionary history of this pathogen. The *C. pneumoniae* human genotype identified in the USA, Canada, Taiwan, Iran, Japan, Korea and Australia (non-Indigenous) most likely originated from a single amphibian or reptilian lineage, which appears to have been previously geographically widespread. We identified a separate human lineage present in two Australian Indigenous isolates (independent geographical locations). This lineage is distinct and is present in Australian amphibians as well as a range of Australian marsupials.

## Author Summary

*Chlamydia pneumoniae* is an intracellular bacterial pathogen with an extremely diverse host range (humans, amphibians, reptiles and marsupials). We selected 23 target genes in order to investigate genetic diversity: seven of these had been lost or gained by *C. pneumoniae*, a further six were conserved, four were polymorphic (defined by greater than 20 SNPs per 1 kbp; in this study), and six were truncated or length polymorphic in one strain or the other. Our research highlights that *C. pneumoniae* animal isolates are much more genetically diverse than *C. pneumoniae* human isolates, and have crossed the host barrier to humans on at least two occasions. Our study provides new insights into the evolution of this complex pathogen.

## Introduction

Members of the family *Chlamydiaceae* are obligate intracellular pathogens of a wide range of animals, birds and humans. Of the nine currently recognised species (*Chlamydia pneumoniae, Chlamydia trachomatis, Chlamydia psittaci, Chlamydia suis, Chlamydia pecorum, Chlamydia abortus, Chlamydia felis, Chlamydia muridarum* and *Chlamydia caviae*), *C. pneumoniae* has an extremely diverse host range (like *C. psittaci*), being reported in humans [1], horses [2], reptiles [3], amphibians [3]–[5] and several Australian marsupials, including koalas [6] and bandicoots [7].

*C. pneumoniae* exposure is widespread in humans, with sero-prevalence studies reporting 50% infection levels by age 20 and reaching 80% in the elderly [8]. In humans, *C. pneumoniae* infections can range from asymptomatic to severe respiratory disease, including pneumonia. Less common presentations include bronchitis, pharyngitis, laryngitis and sinusitis, making up 5% of cases [9]. In addition to respiratory infections in humans, *C. pneumoniae* has also been associated with atherosclerosis and stroke [8], [10], myocarditis [11], multiple sclerosis [12] and Alzheimer's disease [13].

Despite the widespread prevalence of *C. pneumoniae* in humans, all isolates studied to date are extremely similar at the DNA level. Four *C. pneumoniae* human isolates have had their full genome sequenced: AR39 [14], CWL029 [15], J138 [16] and TW183 [17]. Genomic comparisons revealed a highly conserved (>99.9%) gene order and organisation, with few deletions and less than 300 single nucleotide polymorphisms (SNPs) distinguishing the isolates. This near clonality of *C. pneumoniae* human isolates that are temporally and geographically separate, has been taken to indicate that human infections are a relatively recent event [18] and that the efficient respiratory spread of the agent explains how 60–80% of adults worldwide have been infected at least once in their lifetime [19].

In addition to infections in humans, *C. pneumoniae* infections have been reported from a range of animals from an equally diverse range of body sites, including respiratory (frog, snake, bandicoot, koala, horse), liver and spleen (frog, iguana, chameleon), heart (turtle, snake, frog), conjunctival (koala) and urogenital tract (koala) [3]–[5], [7], [20].

While there have been some previous genetic studies on the *C. pneumoniae* animal isolates, these have generally been restricted to partial sequencing of three genes, 16S ribosomal (r) RNA, *ompA* (encoding the major outer membrane protein) and *omcB* (encoding a large cysteine-rich protein) [4]–[6], [21]. Recently, Rattei *et al.* [18] examined the relationship of 38 *C. pneumoniae* isolates, from measurement of genetic diversity within a representative set of 232 synonymous (s)SNPs (no amino acid change; reduced evolutionary pressure). Although only two animal isolates (koala and frog) were examined, two major points have emerged from this study (i) there were 15 genotypes and four major clusters among the isolates, and (ii) the animal lineages were basal to human lineages, suggesting recent transmission to human through successive bottlenecks 150,000 years ago (based on an *Escherichia coli* molecular clock). Myers *et al*. [22] recently sequenced the full genome of the *C. pneumoniae* koala respiratory isolate, LPCoLN. *C. pneumoniae* koala LPCoLN was largely homologous to the previously sequenced *C. pneumoniae* human isolates, although it has several key differences. There are 6,213 SNPs between the koala and human isolates, and importantly, there are several examples of genes that are full-length in the koala LPCoLN isolate, but which have become truncated and fragmented in the human isolates [22]. These data strongly suggest that the koala strain is ancestral to the sequenced human isolates, and points to an animal-to-human cross host transmission event in the (recent) past.

The full genome sequence of *C. pneumoniae* koala LPCoLN has also enabled us to select additional target genes as the basis for an extended genetic and phylogenetic comparison between a broad range of *C. pneumoniae* animal (19) and human (11) isolates. The 30 *C. pneumoniae* isolates that we compared in the current study can be grouped into three categories: (i) seven archival samples (frogs BMTF- type 1 and BMTF-type 2, snakes Pufadd and Burpyth, turtle GST, chameleon cham and iguana Iguana) for which material was no longer available, and three isolates (human LKK1, bandicoot WBB and frog CPXT1) with sequences available in GenBank, restricting comparisons to the already published gene sequences, usually partial 16S rRNA, *ompA* and *omcB*, (ii) five *C. pneumoniae* isolates which have had their entire full genome sequenced (koala LPCoLN and humans AR39, J138, CWL029 and TW183), (iii) 15 isolates which were either available as viable cultures (frog GBF, and humans WA97001 and IOL207) or recoverable tissue material (frogs DE177 and 2040.3, bandicoots B10, B26 and B37, koala EBB, potoroo Pot37, horse N16, and humans SH-511, 1979, TOR1 and A03) which were subjected to gene-specific PCR and sequencing.

## Results/Discussion

### Nucleotide diversity reveals distinct geographical lineages

We had access to a total of 19 *C. pneumoniae* animal isolates (seven marsupial, six amphibian, five reptilian and one equine) and 11 *C. pneumoniae* human isolates (Table S1). We targeted 23 genes from these 30 isolates for analysis. However, due to technical difficulties with some of the DNA preparations, we could not obtain reliable sequence for all genes from all the isolates. *C. pneumoniae* genotypes were assigned by sequence and phylogenetic analyses of nucleotide sequences. Bootstrapped phylogenetic trees are shown in Figure S1. Nucleotide and amino acid (aa) alignments for each gene are presented in Figures S2, S3, S4, S5, S6, S7, S8, S9, S10, S11, S12, S13, S14, S15, S16, S17, S18, S19, S20, S21, S22 and S23. We propose, on the basis of 21 sSNPs from various genomic regions, that the *C. pneumoniae* isolates that were sequenced successfully could be assigned to five common genotypes, A-E (Table S2). The same genotypes were assigned to isolates whose Genbank sequences were included in the analysis (Table S2). The five genotypes show a distinct geographic distribution among the isolates examined (Table S2). Genotype A was common among Australian animals. Genotype B may be indicative of African isolates. Genotype C has a worldwide distribution and was the most common genotype identified. Genotype D was unique to Australian Indigenous isolates. Genotype E was the most variable *C. pneumoniae* genotype and comprised the sole isolate from the United Kingdom. Below is a gene-by-gene comparison to highlight the potential evolutionary relationships among *C. pneumoniae* isolates.

### Genes showing significant size variation/truncation/deletion between isolates

**Figure 1:**
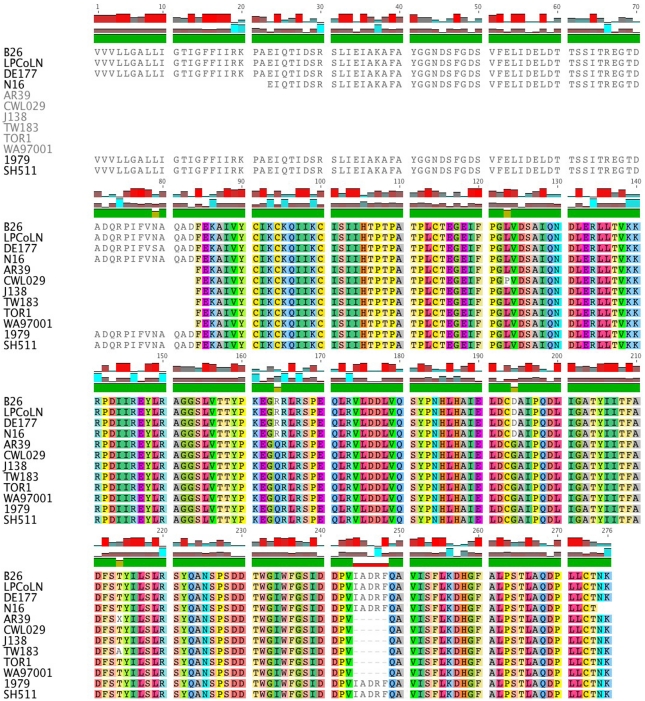
Sequence comparison of CPK_ORF00679. Shown is a multiple sequence alignment of CPK_ORF00679 from bandicoot (B26), koala (LPCoLN), frog (DE177), horse (N16) and human sequences (AR39, CWL029, J138, TW183, TOR1, WA97001, 1979 and SH511. There are two distinct indels: (i) an extended segment at the 5′ end of the amino acid coding sequence, and (ii) a five amino acid indel between positions 244–248. The alignment was generated using Geneious version 4.7, where each amino acid is assigned its own colour. White shading indicates an amino acid variant.

The CPK_ORF00679 (LPCoLN locus designation: CPK) gene encodes a Lamin 2-like protein, which is a conserved hypothetical protein identified in *C. pneumoniae* and *C. felis*. This gene can be used as a target gene for molecular differentiation of *C. pneumoniae.* Sequence analysis of eight *C. pneumoniae* human (AR39, CWL029, TW183, J138, TOR1, WA97001, SH-511 and 1979) and four *C. pneumoniae* animal isolates (koala LPCoLN, bandicoot B26, frog DE177 and horse N16) revealed a distinct size variation at the 5′ end (Figure S2). Three animal isolates (bandicoot B26, koala LPCoLN and frog DE177) had the full-length (833 bp) gene with only five sSNPs among them. Primer walking was used to determine the nucleotide sequence of horse N16 and the Indigenous human isolates when initial attempts with the degenerate primers failed to amplify a product, indicating more distantly related sequences were present. Comparison of the eight *C. pneumoniae* human isolates revealed the absence of 251 bp at the 5′ end of six non-Indigenous human isolates, and only two non-synonymous (n)SNPs (an amino acid change) across the gene. Interestingly, both Australian Indigenous human isolates (SH-511 and 1979) have the 251 bp extended 5′ region of the gene (translating an 83 aa segment), which is identical to the extended 5′ sequence of bandicoot B26, koala LPCoLN and frog DE177 isolates (Figure 1). A second variable region of the gene included a 15 bp indel (insertion/deletion), translating the sequence IADRF position 244-248 aa. This five aa indel is present in isolates koala LPCoLN, bandicoot B26, frog DE177, horse N16, and humans SH-511 and 1979 (Figure 1).

The second length polymorphic gene, Membrane Attack Complex/Perforin (MACPF), remains uncharacterised in *Chlamydia* species, however, its role in other pathogens suggests that it may be virulence-related. Analysis of the partial-length sequence (1,939 bp) revealed only one sSNP differentiating frog DE177 from koala LPCoLN and bandicoots B26 and B37 (Figure S3). PCR confirmed the long version of the gene to be present in horse N16 (data not shown), with 99% identity over the available 1,303 bp to frog DE177, koala LPCoLN and bandicoots B26 and B37 (15 SNPs; data not shown). PCR and sequence analysis of nine *C. pneumoniae* human isolates (AR39, AR39-2 ‘gene sequenced in our laboratory’, CWL029, J138, TW183, TOR1, WA97001, SH511 and 1979) confirmed a length polymorphism (compared to the animal isolates), as the result of a common 840 bp internal deletion. Within this region there was a seven nucleotide indel TGATCCT between positions 1,053–1,055 bp and 1,059–1,062, found in 10 isolates (bandicoots B26 and B37, koala LPCoLN, frog DE177, and humans AR39-2, IOL207, TOR1, WA97001, SH511 and 1979). The indel sequence was absent from the four published *C. pneumoniae* human genomes (AR39, CWL029, J138 and TW183) (Figure S3). A third polymorphism for this gene was evident in Australian Indigenous human isolates SH511 and 1979, which lacked an additional 270 bp fragment between positions 1,333–1,602 bp. The characterisation and identification of polymorphisms within the MACPF should serve as a useful marker in future genetic investigation. For example, the MACPF gene sequence can differentiate *C. pneumoniae* animal isolates from Indigenous human and non-Indigenous human sources.

A significant difference between *C. pneumoniae* and other chlamydial species is the absence of tryptophan biosynthesis genes. Despite this absence, *C. pneumoniae* encodes a functional aromatic amino acid (tryptophan) hydroxylase (AroAA-Hs) [23]. The *C. pneumoniae* aromatic amino acid hyrdoxylase has a unique, extended 5′ region among human isolates, which is absent from koala LPCoLN [23]. To investigate whether the 5′ region may be host specific for *C. pneumoniae* human isolates, we examined the sequence of a bandicoot isolate (B26) and four additional *C. pneumoniae* human isolates (TOR1, WA97001, SH551 and 1979). Sequence analysis confirmed the 244 bp extended 5′ region in all five isolates (identical sequence) (Figure S4). Therefore, the extended 5′ region does not appear to be human host-specific, but may play an important role in regulating its function.

### Polymorphic outermembrane protein (*pmp*) genes

The *pmps* are an important group of *Chlamydia* surface proteins that have been considered as potential vaccine candidates. We selected three variable *pmps* (unknown function) for analysis; (i) *pmp*E/F2, (ii) *pmp*E/F3, and (iii) *pmp*G6. Partial-length sequence comparisons (2,169 bp) of *pmp*E/F2 revealed four unique features (Figure S5), including (i) a total of 64 scattered SNPs (23 nSNPs; 41 sSNPs) differentiating koala LPCoLN and bandicoot B26 from all other isolates, (ii) 10 SNPs (8nSNPs; 2 sSNPs) for frog DE177, six of which were shared with LPCoLN, and four of these were also shared with human SH511 and human 1979 isolates, (iii) four shared SNPs (3 nSNPs; 1 sSNP) between Australian Indigenous human isolates SH511 and 1979, and (iv) one nSNP differentiating human J138 from human isolates AR39, CWL029, J138, TW183, TOR1, and WA97001.

A partial alignment (922 bp) of *pmp*E/F3, revealed four features (Figure S6), including (i) a total of 20 SNPs (8 nSNPs; 12 sSNPs) unique to koala LPCoLN, (ii) two shared SNPs (1 nSNP; 1 sSNP) among koala LPCoLN and frog DE177, (iii) four nSNPs unique to Australian Indigenous human isolates SH511 and 1979, and (iv) no polymorphisms among non-Indigenous human isolates (AR39, TW183, CWL029, J138, TOR1 and WA9001).

**Figure 2:**
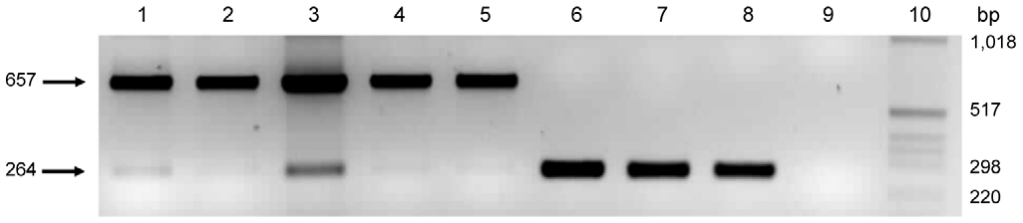
Variable number of tandem repeats polymerase chain reaction (PCR) for the *pmp*G6 gene. Results from isolates with varying tandem repeats are as follows: lane 1 koala LPCoLN/3 repeats; lane 2 bandicoot B26/3 repeats; lane 3 frog DE177/3 repeats; lane 4 human SH511/3 repeats; lane 5 human 1979/3 repeats; lane 6 human AR39/2 repeats; lane 7 human TOR1/2 repeats; lane 8 human WA97001/2 repeats; lane 9 no template control, and; lane 10 MW, molecular weight maker X (Roche, Castle Hill, Australia).

The role of *pmp*G6 is unknown, but owing to the variable number of tandem repeats there may be a functional role *in vivo*. Koala LPCoLN and two *C. pneumoniae* human isolates, TW183 and CWL029, have three tandem repeats of (i) 396 bp, (ii) 393 bp, and (iii) 387 bp, whereas human isolates J138 and AR39 only have two tandem repeats (missing ii – 393 bp). Primers were designed to flank the variable repeat region where a 657 bp product was indicative of 3 tandem repeats, and a 264 bp product was indicative of only 2 tandem repeats (Figure 2). We examined eight *C. pneumoniae* isolates by PCR, including koala LPCoLN and human AR39 positive controls. Five isolates were confirmed to have three tandem repeats (koala LPCoLN, bandicoot B26, frog DE177, and humans SH511 and 1979) and three isolates were identified with only two tandem repeats (humans AR39, WA9701 and TOR1) (Figure 2). This gene can be used as a marker for intra-species variation, differentiating isolates by the size of their PCR product.

### Previously well-studied genes

The 16S rRNA and *ompA* genes have been extensively used to distinguish and phylogenetically group chlamydial species [24]–[27]. Although not as informative as our newly identified target genes, our analysis showed that 16S rRNA, *ompA* and *omcB* were evolving in much the same way as our target genes. A portion of the 16S rRNA gene sequence has been previously determined for several *C. pneumoniae* animal isolates including koala LPCoLN, horse N16, frogs DE177, CPXT1, GBF, BMTF-type 1 and BMTF-type 2, snakes Pufadd and Burpyth, turtle GST, chameleon cham, iguana Iguana, and human isolates AR39, CWL029, TW183, J138, LKK1 and IOL207 [3]–[5], [14]–[17], [22], [28]–[31]. We expanded this comparison to include the additional animal isolates frog 2040.3, bandicoots B10, B26 and B37, potoroo Pot37, koala EBB, and human isolates TOR1, WA97001, SH511 and 1979 (Figure S7). As expected from previous studies, the 16S rRNA sequences were highly conserved between all animal and human isolates. However, the minor differences (SNPs) might indicate some interesting trends in *C. pneumoniae* evolution. Analysis of a 215 bp segment revealed seven SNPs. Of these, two were unique to horse N16 (position 88; G/A and 100; A/C). Frog DE177 had a 2 bp insertion (position 84; T and 85; T), while frog BMTF-type 1 and snake Puffadd shared one unique bp insertion (position 28; A). The two remaining SNPs (position 21; A/G and 51; A/G) differentiated bandicoot (B10, B26, B37 and WBB), koala (LPCoLN and EBB), frog (GBF, 2040.3, DE177 and CPXT1), horse N16 and Australian Indigenous human isolates (SH511 and 1979) from frog (BMTF-type 1 and BMTF-type 2), iguana (Iguana), snake (Puffadd) and non-Indigenous human isolates.

The *C. pneumoniae ompA* gene is much less variable, unlike other species in which several variable domain regions flank diversity. We examined the *ompA* variable domain 4 partial-length (258 bp) sequence of 10 human and 16 animal *C. pneumoniae* isolates from diverse geographical and anatomical locations. From the 258 bp sequence, we identified a total of 27 SNPs (12 nSNPs; 15 sSNPs). Horse N16 had the highest number of SNPs (relative to all other isolates) with 19 unique SNPs (7 nSNPs; 12 sSNPs). All six marsupial isolates (koalas LPCoLN and EBB, bandicoots B26, B37 and WBB, and potoroo Pot37) were identical in sequence, whereas all 10 human isolates (AR39, CWL029, TW183, J138, TOR1, WA97001, IOL207, LKK1, SH511 and 1979) were identical to one another (Figure S8). Region 221–251 bp was the most polymorphic, with a total of 16 SNPs, five of which differentiate marsupial (B26, B37, WBB, EBB, LPCoLN and Pot37) and frog (CPXT1, DE177 and GBF) isolates from frog (BMTF-type 1), snake (Burpyth and puffadd), lizard (chameleon and iguana), turtle (GST), horse (N16) and human isolates (AR39, CWL029, J138, TW183, IOL207, LKK1, TOR1, WA97001, 1979 and SH511) at nucleotide positions 225 (A/G), 227 (G/C), 231 (A/G), 236 (C/T) and 244 (G/A/C).

The *omcB* gene encodes a large cysteine-rich protein, which offers stabilisation through disulphide cross-linkage [32]. Five SNPs were identified from the partial-length (386 bp) comparison of 10 isolates (Figure S9). Three SNPs (2 nSNP; 1 sSNP) were associated with koala LPCoLN, frog DE177 and frog GBF isolates. One sSNP was shared between koala LPCoLN and frog GBF, while another sSNP was unique to horse N16.

Two highly conserved genes from within the plasticity zone include *accC* and *pfk*, encoding acetyl-CoA carboxylase, biotin carboxylase and diphosphate-fructose-6-phosphate 1-phosphotransferase, respectively. A partial length (302 bp) comparison of *accC* revealed only four SNPs, two (position 31; C/A and 171; C/T) differentiated seven non-Indigenous human isolates from two Australian Indigenous human isolates and nine animal isolates. A third SNP (position 185; G/A) was detected in horse N16 and two human isolates (AR39 and J138). The fourth SNP (position 226; T/C) was found in horse N16 and frog 2040.3 isolates (Figure S10). A partial length (263 bp) comparison of *pfk* also revealed four SNPs, two (1 nSNP; 1 sSNP) were unique to horse N16, while two additional SNPs (C/T and G/A) were conserved in koala LPCoLN, bandicoot B37 and frog GBF isolates (Figure S11).

### Hypothetical genes or unique genes showing characteristics specific to *C. pneumoniae*

The CP_1042 (AR39 locus designation: CP) gene encodes a hypothetical protein, which is unique to the *C. pneumoniae* human genome. The four fully-sequenced *C. pneumoniae* human isolates (AR39, CWL029, J138 and TW183) have the full-length (558 bp) gene, whereas koala LPCoLN is missing a 361 bp segment at the 5′ end of the gene; the remaining nucleotides do not translate a protein due to truncation mutations. In order to determine the extent to which genetic diversity occurs in other animal isolates, we examined a frog (DE177), bandicoot (B26), koala (EBB) and horse (N16) isolate, as well as four additional human isolates (TOR1, WA97001, SH511 and 1979) across the 558 bp gene (Figure S12). Like koala LPCoLN, PCR (primers designed for the full-length gene) was unable to detect a product for bandicoot B26 and koala EBB isolates. Interestingly, frog DE177 and horse N16 isolates have the full-length gene, indicating that CP_1042 is not unique to *C. pneumoniae* human isolates. Horse N16 is highly variable with a total of 56 unique SNPs, one of which is shared with frog DE177 and Australian Indigenous human isolates, SH511 and 1979 (Figure S12). All eight human isolates have the full-length gene, with only two SNPs differentiating the non-Indigenous and Australian Indigenous isolates. The fragmentation in the marsupial isolates appears to be host-specific and may not be essential for growth in the marsupial host.

The CP_0880 gene encodes a hypothetical protein, which is variable among *C. pneumoniae* animal isolates, making it difficult to obtain reliable sequence for frog DE177 and horse N16 isolates despite many attempts at different primer set combinations. It was possible to align a partial-length sequence (845 bp) for one bandicoot (B26), two koala (LPCoLN and EBB) and eight human isolates (AR39, CWL029, TW183, J138, TOR1, WA97001, SH511 and 1979) (Figure S13). Of the eight human isolates, four SNPs (2 nSNPs; 2 sSNPS) were detected in SH511 and 1979 Australian Indigenous isolates. Bandicoot B26 and koala LPCoLN isolates were identical in sequence and had diverged from all other isolates, the result of 110 unique SNPs. Of these SNPs, 74 led to an amino acid change, while two SNPs (1nSNP; 1sSNP) were shared with Australian Indigenous human SH511 and 1979 isolates.

The CP_0505 gene encodes a hypothetical protein. Sequence analysis (Figure S14) of the partial-length (723 bp) gene revealed four features (i) 13 SNPs (8 nSNPs; 5 sSNPs) unique to koala LPCoLN and bandicoots B26 and B37, (ii) 42 SNPs (24 nSNPs; 18 sSNPs) unique to horse N16, (iii) two SNPs (1 nSNP; 1 sSNP) unique to Australian Indigenous SH511 and 1979 isolates, while a third sSNP was shared with bandicoots B26 and B37, koala LPCoLN and horse N16 isolates, and (iv) a single bp deletion distinguished frog DE177 from the non-Indigenous isolates (identical sequence).

The SctC gene encodes a type III secretion system protein, which has a *C. pneumoniae-*specific (approximately 700 bp) 5′ region. Type III secretion system proteins are well-conserved among *Chlamydia* species (and other bacteria), therefore, it was not surprising to find little variation among the *C. pneumoniae* isolates. Overall, there were five SNPs. Two nSNPs (position 232; A/C and 273; A/C) distinguished bandicoot B26, koala LPCoLN, frog DE177 and Indigenous human isolates SH511 and 1979 from the non-Indigenous human isolates AR39, CWL029, J138, TW183, TOR1 and WA97001 (identical sequences). Frog DE177 had one sSNP (position 342; T/C). Koala LPCoLN and bandicoot B26 had the same nSNP (position 429; G/T). Australian Indigenous human isolates SH511 1979 shared one nSNP (position 469; A/G) (Figure S15). This 482 bp gene segment might be a useful marker for *C. pneumoniae* detection and differentiation of animal isolates from Indigenous and non-Indigenous human isolates, on the basis of five key SNPs.

The CPK_ORF00201 gene (termed HAF) encodes a HAF family auto-transporter beta domain protein. Nucleotide comparisons of the fully-sequenced *C. pneumoniae* isolates (koala LPCoLN and human AR39, CWL029, TW183 and J138) revealed a truncated (two separate genes) homolog among human isolates; absence of a 120 bp internal segment. A partial-length (1,412 bp) comparison with seven previously un-sequenced *C. pneumoniae* isolates (bandicoot B26, frog DE177, horse N16, human WA97001, TOR1, SH511 and 1979) revealed an intact, full-length gene in all seven isolates (Figure S16). One possible explanation for this difference may be due to sequencing error in the *C. pneumoniae* human genomes; the missing segment is revealed in a BLAST search. Limited sequence was available for horse N16, despite many attempts with different primer sets, suggesting that there may be more sequence variation in the region that could not be amplified. There were, however, nine unique SNPs (4 nSNPs; 5 sSNPs) detected in the horse N16 isolate. It is worth mentioning that horse N16, bandicoot B26, koala LPCoLN, frog DE177 and both Australian Indigenous human isolates (SH511 and 1979) had two common SNPs at nucleotide positions 761/T and 1,020/T, while six non-Indigenous human isolates had C/C at these positions. These polymorphisms could be used to distinguish animal and Indigenous human isolates from non-Indigenous human isolates.

### Strain-specific genes of *C. pneumoniae*

Three genes present in the *C. pneumoniae* human genome, but absent from the koala LPCoLN genome include *guaA* (GMP synthase), *guaB* (IMP dehydrogenase) and *add* (AMP adenosine deaminase). This three-gene cluster, located in the plasticity zone, was also confirmed by PCR in the frog (DE177) and horse (N16) isolates. A comparison of *guaB* (Figure S17) revealed just three SNPs differentiating frog DE177 from the non-Indigenous human *C. pneumoniae* isolates (AR39, CWL029, TW183, J138, TOR1 and WA97001), while no polymorphisms were evident in the *guaA* gene (Figure S18). Reliable *add* sequence was not available for frog DE177 (Figure S19). The horse N16 isolate had four unique SNPs for *guaB* (Figure S17); however, reliable sequence was not available for *guaA* and the presence of *add* was not definitive in the horse N16 isolate. The *guaBA-add* cluster was identified (identical sequence) in human isolates TOR1, WA97001 and IOL207 (limited sequence) (Figures S17, S18 and S19). Both Australian Indigenous human isolates (SH511 and 1979) have the *guaB* gene with six SNPs (4 nSNPs; 2 sSNPs) to the non-Indigenous human isolates; two SNPs were shared with frog DE177 and horse N16 isolates (Figure S17). Since PCR failed to amplify any product for *guaA* and *add* genes in the Australian Indigenous human isolates (using two sets of primers for each gene from either 5′ or 3′ regions), these two genes are either absent from the genome, or are highly divergent and undetectable with our primer sets. Three additional animal isolates (koala EBB, bandicoots B26 and B37) also lack the *guaBA*-*add* cluster which suggests that it is not a core component required for *C. pneumoniae* survival.

The CPK_ORF00678 gene encodes a hypothetical protein, which is located within the plasticity zone of the koala LPCoLN genome. This gene was not identified in any of the four fully-sequenced *C. pneumoniae* human isolates (AR39, CWL029, J138 and TW183) or subsequent human isolates (WA97001, TOR1, SH511 and 1979), but was detected in three additional animal isolates (bandicoot B26, frog DE177 and horse N16) (Figure S20). A partial-length sequence comparison (698 bp) revealed 10 SNPs (4 nSNPs; 6 sSNPs) differentiating frog DE177 from koala LPCoLN and bandicoot B26 isolates (identical sequence). Four of these SNPs were also shared with horse N16 (Figure S20). Of the animal isolates, horse N16 showed greater sequence divergence with 11 unique SNPs and four indels (positions 73–84, 151–305, 387–395 and 419–425). The absence of CPK_ORF00678 from the *C. pneumoniae* human genome suggests that this gene may have been selected for a specific function in the animal host, and thus, provides a new animal *C. pneumoniae*-specific target gene.

Koala LPCoLN encodes a plasmid (7,655 bp) with eight ORFs (open reading frames) [22]. This plasmid is absent from *C. pneumoniae* human isolates [14]–[17]. Thomas *et al*. [33] detected a plasmid in the horse N16 isolate, and so it was of interest to see whether additional *C. pneumoniae* isolates have a plasmid. Primers (Table S3) were designed to investigate three plasmid ORFs: (i) site-specific recombinase II; SSR2, (ii) replicative DNA helicase dnaB; helicase, and (iii) a conserved hypothetical protein; PGP3D. Seventeen isolates were examined by PCR for the presence of a plasmid, including three bandicoots (B10, B26 and B37), three frogs (GBF, DE177 and 2040.3), two koalas (LPCoLN and EBB), one potoroo (Pot37), one horse (N16) and seven human isolates (AR39, IOL207, WA97001, A03, TOR1, SH511 and 1979). All three plasmid ORFs were confirmed by PCR in each of the 10 animal isolates, although no human isolate was found to have SSR2 (Figure S21), helicase (Figure S22) or PGP3D (Figure S23) ORFs. Of the three ORFs, SSR2 showed evidence of a length difference when compared to the horse N16 isolate. This isolate was missing a 165 bp segment between positions 66–230 bp, while bandicoots B10 and B37, frogs DE177, 2040.3, GBF and koala LPCoLN sequences were identical and contained the whole 369 bp segment under examination (Figure S21).

### Phylogenetic relationships among *C. pneumoniae* isolates

Despite the importance and widespread prevalence of *C. pneumoniae*, there has been little phylogenetic analysis to assist evolutionary and epidemiological investigations. Therefore, in order to examine in-depth the evolutionary relationships within *C. pneumoniae,* we examined several previously studied isolates in addition to 10 novel isolates using 22 target genes selected from various regions of the genome. We constructed rooted phylogenetic trees from all available *C. pneumoniae* isolates by Neighbour-Joining and UPGMA (Unweighted Pair Group Method with Arithmetic mean) methods. As both trees were of similar structure, only the Neighbour-Joining tree is presented (Figure S1). The phylogenetic patterns that we observed were congruent in 19 out of the 22 genes that we analysed. This gives us confidence that our interpretations are sound and suggests that horizontal gene transfer is not a major contributor to genetic changes in *C. pneumoniae*. These phylogenetic analyses led us to propose several key new findings. The Australian Indigenous human isolates (genotype D) formed a unique group in 10 out of the 15 trees analysed. These could be clearly distinguished from the non-Indigenous human isolates (genotype C) which formed a tight cluster, regardless of their geographical origin. The evolutionary position of horse N16 (genotype E) is somewhat less confident. Horse N16 was distinct from all other isolates in at least 9 out of the 13 trees examined. However, the bootstrap values were low at 54–78%. It will be necessary to obtain additional equine isolates to confirm the correct lineage of this strain. The Australian marsupial isolates formed a tight grouping (genotype A) with all genes analysed, even when they were obtained from different geographical regions within Australia. The Australian marsupial isolates shared common nucleotide substitutions with the amphibian and horse isolates originating from Australia, Africa and the United Kingdom, as did the Australian Indigenous human isolates. Analysis of the Australian frog isolates resulted in the identification of two distinct genotypes. Genotype C was identified in two captive frog isolates from New South Wales (BMTF-type 1 and BMTF- type 2). A third free-range frog (GBF) isolate, also from New South Wales, was identical to the sequence derived from Australian marsupial isolates, from independent regions. The third isolate was therefore clustered into genotype A. The degree of variation in the limited sequence analysed was unusual given the geographical distance from other world regions. Moreover, two African frog isolates from different regions were clustered together, in genotype B. However, a third African isolate from a chameleon was grouped with the international cluster in genotype C, comprising American snakes, an American turtle, a Central American iguana, Australian frogs, as well as the non-Indigenous human isolates. Members of genotype C are both common and present across the globe, possibly indicating that it is a relatively recent expansion, as proposed by Rattei *et al.* [18].

### Conclusions

#### Sequence data suggests that two separate animal to human transmission events have occurred

Our aim was to determine whether our target genes could distinguish *C. pneumoniae* isolates from human and animal sources and to investigate their genetic diversity. Previous analyses have shown that *C. pneumoniae* human isolates are essentially clonal and do not provide much evolutionary insight. To our knowledge, all previous analyses of *C. pneumoniae* human have focussed on non-Indigenous isolates: AR39 (USA), CWL029 (USA), TW183 (Taiwan), J138 (Japan), LKK1 (Korea), A03 (USA), TOR1 (Canada) and IOL207 (Iran). Our analysis also included a non-Indigenous human isolate from Australia (WA97001), and more importantly, we were able to analyse two Australian Indigenous human isolates (SH511 and 1979) which originated from geographically separate communities.

Our collective sequence data (using 23 target genes from 30 isolates) and phylogenetic analyses strongly support both the whole genome findings of Myers *et al.* [22] as well as the synonymous SNP conclusions of Rattei *et al.* [18] in that the extant *C. pneumoniae* human isolates have derived from *C. pneumoniae* animal isolates. Our data extends both of these studies, however, and strongly suggests two (or more) lineages of *C. pneumoniae*. One lineage involves amphibian isolates (DE177, CPXT1, BMTF-type 1 and BMTF-type 2), which subsequently ‘evolved’ to *C. pneumoniae* infections in reptiles (Iguana, Pufadd, Burpyth, GST, cham) and via as yet undiscovered intermediates, to the dominant *C. pneumoniae* human clone present in the world today (AR39, TW183, CWL029, J138, TOR1, A03, IOL207, LKK1, and WA97001). The second presumably also started with amphibian infections (as above), but then diverged into other amphibian and reptilian infections (frogs GBF and 2040.3). At this point (there are probably intermediate hosts and *C. pneumoniae* strains that are either extinct or as yet, undiscovered) two sub-lines are evident. One lineage has resulted in the widespread *C. pneumoniae* infections seen in Australian marsupials today (koala LPCoLN, koala EBB, bandicoot B10, bandicoot B26, bandicoot B37, bandicoot WBB and potoroo Pot37) while the second lineage has crossed the animal-human barrier to infect Australian Aboriginals (SH511 and 1979). The key genomic data, which supports these lineages and their divergence is summarised in Supporting Information Text S1.

Although the evolutionary time-frame of *C. pneumoniae* is unclear, we have described patterns of genetic differentiation whereby *C. pneumoniae* profiles from the Australian Indigenous human populations appear to be negatively correlated (for the most part) with the genetic differentiation in non-Indigenous human populations. The possibility that *C. pneumoniae* was an established pathogen in Australia before the settlement of Europeans in 1788, is one possible theory considering the characteristic diseases of a hunter-gatherer population have been described as those with low morbidity, long illness and long infection stages, such as chlamydial diseases [34]. From ancient times, hunter-gatherer Aboriginal communities have lived in close proximity to animals, and may have been predisposed to *C. pneumoniae* infected animals. However, if *C. pneumoniae* had been present in the Australian Indigenous population prior to European settlement, the differences observed in our target genes would suggest that the Australian Indigenous isolates have evolved independently from the non-Indigenous isolates. This trend was also observed in an Australian Indigenous and non-Indigenous cohort of *Haemophillus influenzae* isolates from Australia [35] where the genetic diversity and the time span from European settlement was not likely to support the amount of differentiation observed. A likely explanation for the observed diversity in our human isolates would be that *C. pneumoniae* may have been more recently introduced to urban non-Aboriginals from infected animals, or alternatively via native or non-native inhabitants in other regions around the world, and the infections were not derived from the Indigenous Australians; the United Kingdom horse (N16) and African frog (DE177) isolates also had indels in common to the Australian marsupials (koalas LPCoLN and EBB, bandicoots B10, B26, B37 and WBB, and potoroo Pot37) and Australian Indigenous human isolates, despite being geographically isolated from the Australian population. There is convincing evidence that zoonotic transmission of *C. pneumoniae* can/has occur/ed. The finding that a genotype common to non-human and human hosts was dispersed in human carotid plaques [36] is not by itself conclusive evidence of zoonotic transmission, but rather highlights the need for consideration of its zoonotic potential, particularly for animal handlers and laboratory personnel.

In the present study, we observed five genotypes (A-E) based on genetic and phylogenetic data. These data revealed dominant genotypes in various regions of the world. Genotype A was prevalent among Australian animals, genotype B was identified in Africa, genotype C had the broadest distribution and was worldwide, genotype D was predominant among Indigenous Australians, while genotype E was unique to the United Kingdom. Although a very limited number of isolates were used in this study, the results clearly showed genetic diversity associated with host type and geographic locations. Based on the large number of polymorphisms between horse N16 and all other *C. pneumoniae* isolates, we believe that we have identified sufficient genetic differences that would accommodate the classification of horse N16 into a subspecies of *C. pneumoniae*, designated *C. pneumoniae* subsp. *equi*, following the proposal of Pettersson *et al.* [29]. These findings of high genetic variation may represent mosaic genotypes in the population and may need to be considered as a subspecies variant within *C. pneumoniae.*

## Materials and Methods

### *Chlamydia pneumoniae* isolates

30 isolates from 11 human, 7 marsupial, 5 reptilian, 6 amphibian and 1 equine host were analysed (Table S1).

The *C. pneumoniae* Pot37 isolate was initially obtained from a pharyngeal swab of a Gilbert's potoroo (*Potorous gilbertii*) from Perth, Western Australia in 2006. The *C. pneumoniae* EBB isolate was isolated from a pharyngeal swab of a koala (*Phascolarctos cinereus*) from Queensland, Australia in 2005.

All analyses involving the Australian Indigenous human samples were approved by the Queensland University of Technology and the Menzies School of Health Research, Human Research Ethics Committee. All human samples were obtained from a previous study, in which informed written consent for the collection of samples and subsequent analysis was correctly obtained and ethics approval obtained. All animal samples were obtained from previous studies and experiments were performed according to national and institutional guidelines with ethics, biosafety and animal care committee approval (QUT IBC No. 1413/2A, QUT IBC No. 0900000267, QUT IBC No. 928A).

### Strategy for selection of genes used for comparisons

Recently, we examined five complete *C. pneumoniae* genomes, including koala LPCoLN and the four available *C. pneumoniae* human (AR39, TW183, CWL029 and J138) genomes (Mitchell *et al.*, unpublished data). This approach allowed us to identify regions of high SNP accumulation and to select candidate genes for comparison. In this study, we selected 13 of these genes for comparative analysis using a broader selection of isolates, while 1 additional length variable gene, 1 polymorphic gene, 5 highly conserved genes and three plasmid genes were also selected for analysis. Our target genes could be grouped into six categories:

- three genes (CPK_ORF00679, AroAA-Hs and MACPF) showing length polymorphisms, of at least 100 bp between *C. pneumoniae* koala and human isolates. Aromatic amino acid hydroxylases (AroAA-Hs) hydroxylate phenylalanine, tyrosine, and tryptophan into tyrosine, dihydroxyphenylalanine, and 5-hydroxytryptophan, respectively [23]. CPK_ORF00679 and MAC/perforin (MACPF) genes have an unknown role, although the MACPF may be a potential virulence gene based on the function in other intracellular pathogens.
- three polymorphic outer membrane protein genes; two (*pmp*E/F2 and *pmp*E/F3) have regions with more than 20 SNPs per 1 kbp between *C. pneumoniae* koala and human isolates, and one (*pmp*G6) varies in the number of tandem repeats.
- three previously well-studied ‘conserved’ genes: 16S rRNA belongs to a small unit of ribosomes, *ompA* is a major outermembrane protein and *omcB* is a large cysteine-rich outer membrane protein; and two highly conserved genes (*accC* and *pfk*) located within the plasticity zone – *accC* (acetyl-CoA carboxylase, biotin carboxylase) is involved in fatty acid/phospholipid metabolism and *pfk* (diphosphate-fructose-6-phosphate 1-phosphotransferase) is involved in glycolysis/gluconeogenesis.
- three hypothetical genes (CP_1042, CP_0880 and CP_0505) with an unknown function.
- two *C. pneumoniae*-specific genes (SctC and HAF). SctC (highly conserved) forms part of the type III secretion system and has a unique 700 bp at the 5′ region of the gene. HAF is predicted to be a HAF family-autotransporter (beta domain) protein.
- seven strain-specific genes: *guaA* (GMP synthase), *guaB* (IMP dehydrogenase) and *add* (AMP adenosine deaminase) are located in the plasticity zone and were absent from koala LPCoLN; CPK_ORF00678 is a hypothetical gene that is absent from all four full-sequenced *C. pneumoniae* human isolates, and; the plasmid is an extra-chromosomal element identified in the koala LPCoLN genome. Three plasmid genes were examined: (i) site-specific recombinase II; SSR2, (ii) replicative DNA helicase dnaB; helicase, and (iii) a conserved hypothetical protein; PGP3D.

The strategy involved PCR-based amplification of six human isolates and nine animal isolates, followed by sequencing of the PCR products. Additional sequences for 16S rRNA, *ompA* and *omcB* were retrieved for previously sequenced human and animal *C. pneumoniae* isolates (refer to accession numbers). The analysis included; (i) gene by gene sequence alignments to identify SNPs and indels, and (ii) generation of bootstrapped phylogenetic trees.

### PCR and sequencing

Oligonucleotide primers (Sigma-Aldrich, Castle Hill, Australia) were designed based on homology of *C. pneumoniae* LPCoLN and AR39 sequences (GenBank accession numbers CP001713 and AE002161) using Primer3 v. 0.4.0 (denoted by an asterisk) (http://biotools.umassmed.edu/bioapps/primer3_www.cgi) [37] and two primers already published (Table S3). The primer pairs, expected PCR product sizes and annealing temperatures are summarised, and several of the target genes have internal primers to sequence a particular region because of sequence variation or to enable sequencing of whole PCR products (Table S3). PCR reactions were performed in a final volume of 50 µl, including 5.0 µl 10X PCR reaction buffer (Roche, Castle Hill, Australia), 1.0 µl PCR nucleotide mix (Roche, Castle Hill, Australia), 2.0 µl of each (10 µM) primer (Sigma-Aldrich, Castle Hill, Australia), 0.2 µl of 5 U/µl Taq DNA polymerase (Roche, Castle Hill, Australia), 2.0 µl of template and PCR grade water to a final volume of 50 µl. Amplification conditions consisted of an initial denaturation at 94°C, followed by 30 cycles of 1 min at 94°C, 1 min at the specified annealing temperature (refer to Table S3), 1 min at 72°C, and a final extension for 10 min at 72°C. PCR products were separated by electrophoresis and visualised by ethidium bromide (10 µg/ml) staining of a 2% agarose gel in Tris Borate EDTA (TBE) buffer.

PCR products were purified with a PureLink PCR purification kit (Invitrogen, Australia). To confirm sequence confirmation, DNA sequencing was performed in both directions using a BigDye terminator Cycle Sequencing Ready Reaction Kit and an automated DNA sequencer AB 3730xl (Australian Genome Research Facility, University of Queensland, Australia). A third sequence was obtained if required.

### Sequences and phylogeny

Nucleotide sequences were translated to amino acids using blastx (www.ncbi.nlm.nih.gov/blast). Nucleotide and derived amino acid sequences were then trimmed to a uniform length. Sequence alignments were generated with Geneious version 4.7 using the pairwise alignment default settings (Nucleotide Cost Matrix = 65% similarity 5.0/−4.0, Protein Cost Matrix = Blosum62, Gap Open = 12.0, Gap Extension = 3.0, Alignment Type  =  global alignment with free end gaps) [38; http://www.geneious.com]. The amino acid colour scheme is standard where each amino acid has an assigned colour. Phylogenetic trees were generated with Geneious version 4.7 using the pairwise alignment default settings (described above), and a bootstrap consensus phylogenetic tree was constructed by Neighbor-Joining or UPGMA analysis and Jukes-Cantor correction using 1,000 bootstrap replicates [38]. Bootstrap values greater than or equal to 50% are shown at the nodes.

### Nucleotide and amino acid sequence accession numbers

The nucleotide and amino acid sequence of the target genes have been deposited in the GenBank database, and the accession numbers are as follows: CPK_ORF00679 GQ918195 to GQ918201; MACPF GQ918233 to GQ918240; AroAA-Hs GQ918166 to GQ918170; *pmp*E/F2 GQ507462 to GQ507467; *pmp*E/F3 GQ918162 to GQ918165; 16S rRNA GQ507433 to GQ507442; *ompA* GQ918216 to GQ918222; *accC* GQ918224 to GQ918232, GU013548; *pfk* GQ918243 to GQ918254; CP_1042 GQ918189 to GQ918194; CP_0880 GQ507456 to 507461; CP_0505 GQ507449 to GQ507455; SctC GQ507443 to GQ507448; HAF GQ918202 to GQ918208; *guaA* GQ918156 to GQ918158; *guaB* GQ918209 to GQ918215; *add* GQ918154 to GQ918155; CPK_ORF00678 GQ918159 to GQ918161; SSR2 GQ918171 to GQ918176; helicase GQ918183 to GQ918188; PGP3D GQ918177 to GQ918182.

## Supporting Information

Figure S1 **Phylogenetic trees (A-V) of** ***C. pneumoniae*** **isolates.** Phylogenetic relationships of *C. pneumoniae* isolates were inferred from partial nucleotide sequences, and were constructed by Neighbor-Joining analysis and the Jukes-Cantor correction model using 1,000 bootstrap replicates. (A) CPK_ORF00679, (B) MACPF, (C) AroAA-Hs, (D) *pmp*E/F2, (E) *pmp*E/F3, (F) 16S rRNA, (G) *ompA*, (H) *omcB*, (I) *accC*, (J) *pfk*, (K) CP_1042, (L) CP_0880, (M) CP_0505, (N) ScTc, (O) HAF, (P) *guaB*, (Q) *guaA*, (R) *add*, (S) CPK_ORF00678, (T) SSR2, (U) helicase, and (V) PGP3D. There is evidence of five distinct phylogenetic groupings among isolates. These groupings correspond to the five proposed genotypes, A-E (Table S2). The 19/22 trees were congruent, while the phylogenetic incongruities that were observed in 3 trees (H, J and M) may be the result of host interactions or adaptation, rather than acquisition through horizontal transfer. The nomenclature for each isolate is shown in Table S1.
(7.50 MB PDF)

Figure S2 **Multiple sequence alignment of CPK_ORF00679.** Note the size variation at the 5′ end and the indel at nucleotide positions 732-746.
(3.29 MB PDF)

Figure S3 **Multiple sequence alignment of MACPF.** Animal isolates have the full-length gene, whereas human isolates have an 840 bp/280 aa internal deletion. There is a seven nucleotide indel (TGATCCT) present between 1,053-1,055 bp and 1,059-1,062 bp. An additional polymorphism (270 bp deletion) was present in two Australian Indigenous isolates SH511 and 1979 between positions 1,333-1,602.
(4.11 MB PDF)

Figure S4 **Multiple sequence alignment of AroAA-Hs.** Sequence from bandicoot (B26), koala (LPCoLN) and six human isolates (AR39, TW183, CWL029, J138, SH511 and 1979) identified a 244 bp length polymorphism at the 5′ end.
(6.05 MB PDF)

Figure S5 **Multiple sequence alignment of** ***pmp*** **E/F2.** The nucleotide and amino acid alignments were generated using Geneious version 4.7, where each nucleotide and amino acid is assigned its own colour. White shading indicates an amino acid variant.
(6.27 MB PDF)

Figure S6 **Multiple sequence alignment of** ***pmp*** **E/F3.** The nucleotide and amino acid alignments were generated using Geneious version 4.7, where each nucleotide and amino acid is assigned its own colour. White shading indicates an amino acid variant.
(1.72 MB PDF)

Figure S7 **Multiple sequence alignment of 16S rRNA.** The nucleotide alignment was generated using Geneious version 4.7, where each nucleotide is assigned its own colour. The total alignment length is 215 bp.
(4.92 MB PDF)

Figure S8 **Multiple sequence alignment of** ***ompA*** **.** The nucleotide and amino acid alignments were generated using Geneious version 4.7, where each nucleotide and amino acid is assigned its own colour. White shading indicates an amino acid variant.
(1.42 MB PDF)

Figure S9 **Multiple sequence alignment of** ***omcB*** **.** The nucleotide and amino acid alignments were generated using Geneious version 4.7, where each nucleotide and amino acid is assigned its own colour. White shading indicates an amino acid variant.
(0.94 MB PDF)

Figure S10 **Multiple sequence alignment of** ***accC*** **.** The nucleotide and amino acid alignments were generated using Geneious version 4.7, where each nucleotide and amino acid is assigned its own colour. White shading indicates an amino acid variant.
(0.71 MB PDF)

Figure S11 **Multiple sequence alignment of** ***pfk*** **.** The nucleotide and amino acid alignments were generated using Geneious version 4.7, where each nucleotide and amino acid is assigned its own colour. White shading indicates an amino acid variant.
(0.96 MB PDF)

Figure S12 **Multiple sequence alignment of CP_1042.** Sequence from koala (LPCoLN), frog (DE177), horse (N16), and six human isolates (AR39, CWL029, J138, TW183, SH511 and 1979) revealed gene fragmentation in the koala LPCoLN isolate.
(1.13 MB PDF)

Figure S13 **Multiple sequence alignment of CP_0880.** The two koala isolates (LPCoLN and EBB) and bandicoot isolate (B26) are identical in sequence and have diverged genetically (110 SNPs) from the eight human isolates (AR39, CWL029, J138, TW183, TOR1, WA97001, SH511 and 1979). There are two shared polymorphisms (positions 714 and 818 bp) among koala LPCoLN, bandicoot B26 and Indigenous human isolates SH511 and 1979.
(5.80 MB PDF)

Figure S14 **Multiple sequence alignment of CP_0505.** There are four features of interest: (i) 13 SNPs unique to koala LPCoLN and bandicoots B26 and B37, (ii) 42 SNPs unique to horse N16, (iii) two SNPs unique to Australian Indigenous SH511 and 1979 isolates, and a third sSNP is shared with horse N16, koala LPCoLN, bandicoot B26 and bandicoot B37 isolates, and (iv) a single bp deletion distinguishing frog DE177 from the non-Indigenous isolates (identical sequence).
(5.43 MB PDF)

Figure S15 **Multiple sequence alignment of SctC.** This gene segment can differentiate animal isolates from Indigenous and non-Indigenous human isolates, on the basis of five key SNPs: Two SNPs (position 232; A/C and 273; A/C) distinguish bandicoot B26, koala LPCoLN, frog DE177 and Indigenous human isolates SH511 and 1979 from the non-Indigenous human isolates AR39, CWL029, J138, TW183, TOR1 and WA97001 (identical sequences); frog DE177 has one unique SNP (position 342; T); koala LPCoLN and bandicoot B26 have one shared SNP (position 429; G); Australian Indigenous human isolates SH511 1979 have one shared SNP (position 469; A).
(1.17 MB PDF)

Figure S16 **Multiple sequence alignment of HAF.** The sequenced human *C. pneumoniae* genomes are truncated (120 bp deletion), whereas additional human isolates (TOR1, WA97001, SH511, and 1979) and animal isolates (B26, DE177 and N16) are not truncated.
(2.31 MB PDF)

Figure S17 **Multiple sequence alignment of** ***guaB*** **.** The nucleotide and amino acid alignments were generated using Geneious version 4.7, where each nucleotide and amino acid is assigned its own colour. White shading indicates an amino acid variant.
(1.26 MB PDF)

Figure S18 **Multiple sequence alignment of** ***guaA*** **.** The nucleotide and amino acid alignments were generated using Geneious version 4.7, where each nucleotide and amino acid is assigned its own colour. White shading indicates an amino acid variant.
(1.08 MB PDF)

Figure S19 **Multiple sequence alignment of** ***add*** **.** The nucleotide and amino acid alignments were generated using Geneious version 4.7, where each nucleotide and amino acid is assigned its own colour. White shading indicates an amino acid variant.
(2.87 MB PDF)

Figure S20 **Multiple sequence alignment of CPK_ORF00678.** Horse N16 has four indels (positions 73-84, 151-305, 387-395 and 419-425), relative to frog DE177, bandicoot B26 and koala LPCoLN.
(0.77 MB PDF)

Figure S21 **Multiple sequence alignment of SSR2.** Horse N16 has a 165 bp indel at positions 66-230 bp.
(0.58 MB PDF)

Figure S22 **Multiple sequence alignment of helicase.** The nucleotide and amino acid alignments were generated using Geneious version 4.7, where each nucleotide and amino acid is assigned its own colour. White shading indicates an amino acid variant.
(0.64 MB PDF)

Figure S23 **Multiple sequence alignment of PGP3D.** The nucleotide and amino acid alignments were generated using Geneious version 4.7, where each nucleotide and amino acid is assigned its own colour. White shading indicates an amino acid variant.
(0.47 MB PDF)

Table S1 ***Chlamydia pneumoniae*** **isolates used in this study.** Complete list of *C. pneumoniae* isolates and description of their natural host, year of isolation, specimen type and reference. NA, information not available.
(0.05 MB DOC)

Table S2 **Synonymous SNP profile for 10 selected genes and their genotype designation.** Complete list of *C. pneumoniae* isolates and corresponding nucleotide base pairs at particular locations, across eight genes. Genotypes were designated A-E. Dashes indicate no sequence analysed.
(0.03 MB XLS)

Table S3 **Oligonucleotide primers used in this study.** The fragment sizes are estimated from koala LPCoLN and human AR39 sequences. * Designed using Primer3 v.0.4.0.
(0.06 MB DOC)

Text S1 **Key genomic data supporting two evolutionary lineages.**
(0.03 MB DOC)
