# Supplementary figures and images for: Chlamydia pneumoniae Is Genetically Diverse in Animals and Appears to Have Crossed the Host Barrier to Humans on (At Least) Two Occasions
Source: PLoS Pathog. 2010 May 20;6(5):e1000903. doi: 10.1371/journal.ppat.1000903 (PMC2873915; doi:10.1371/journal.ppat.1000903)

**A**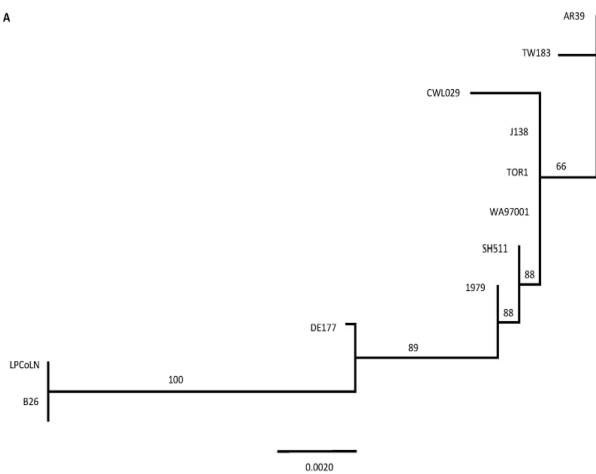**B**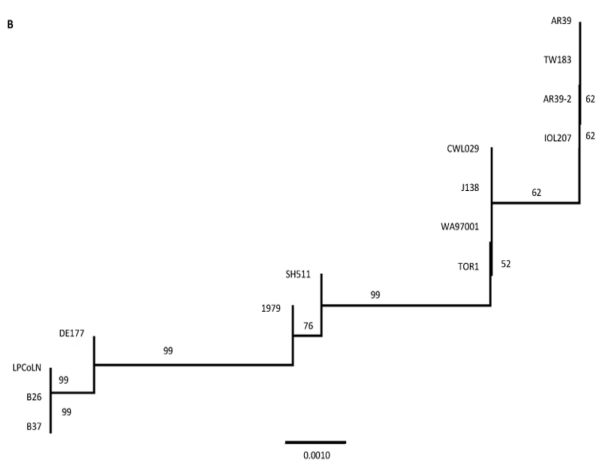**C**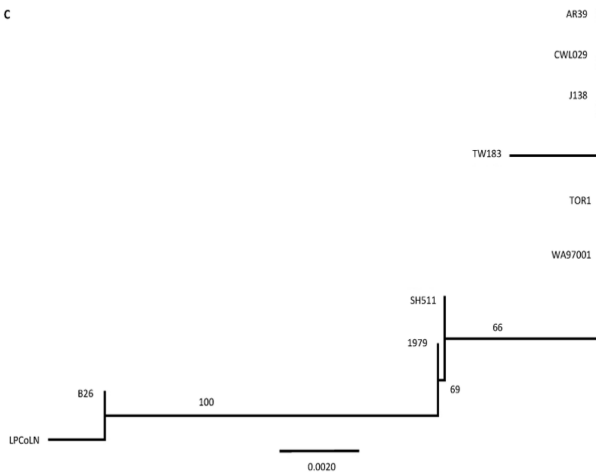**D**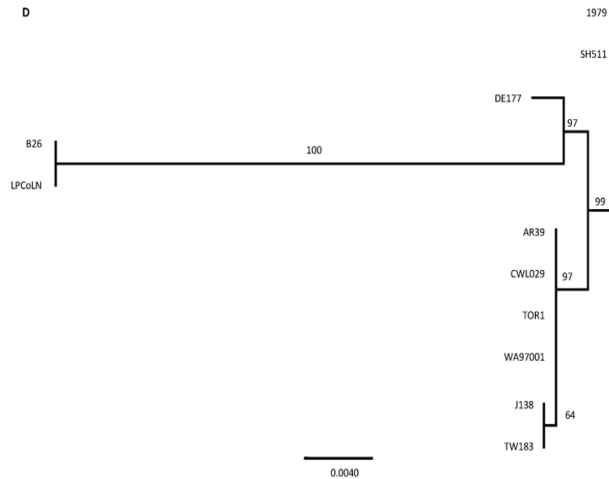

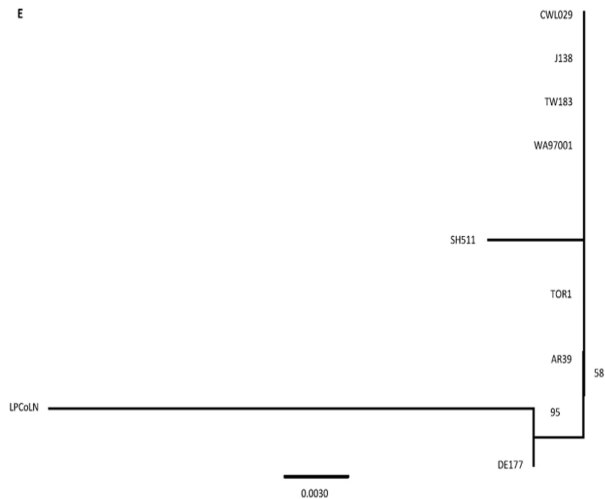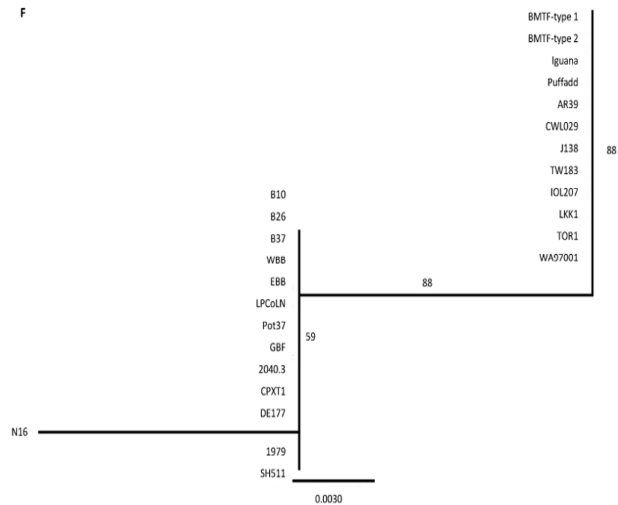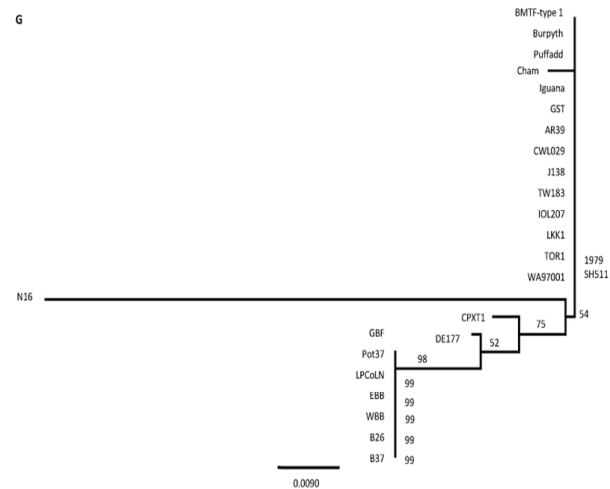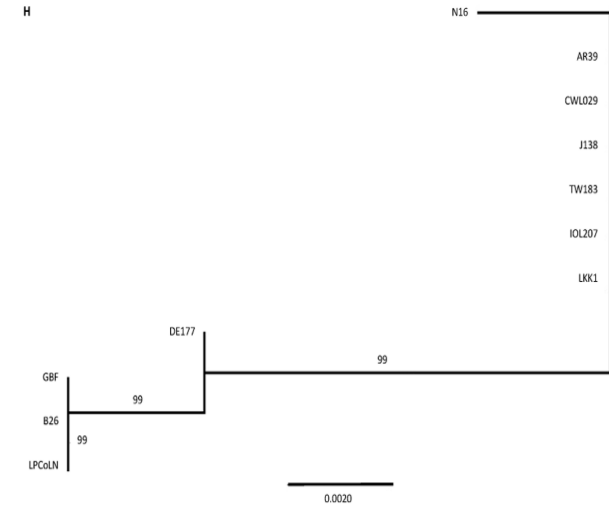

I

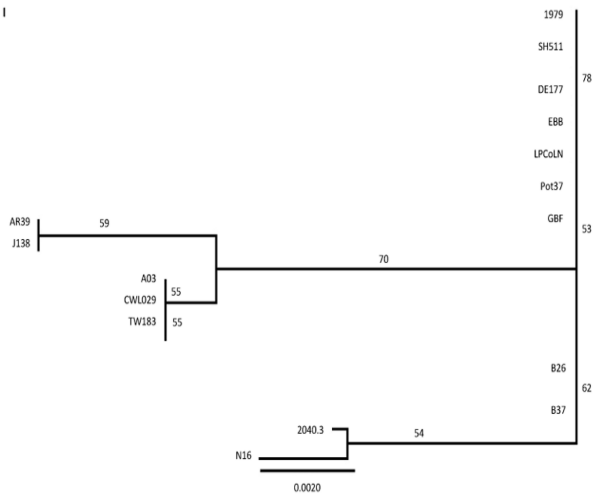

J

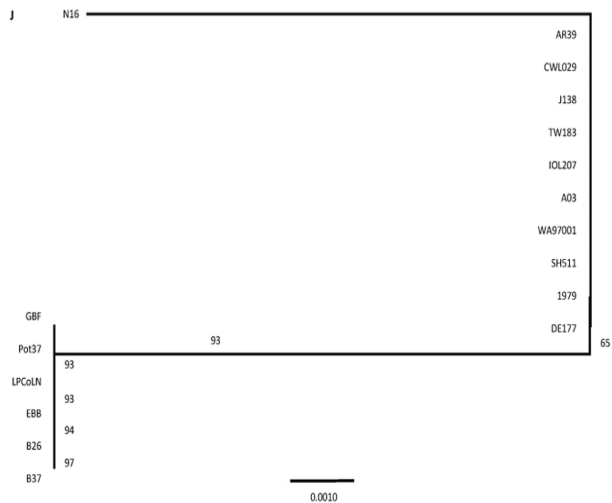

K

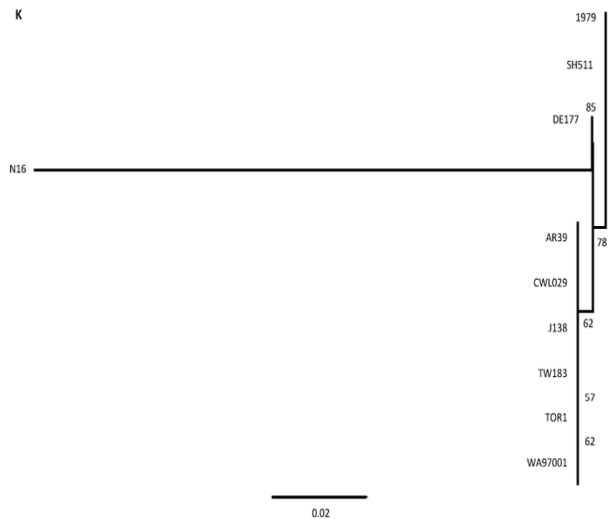

L

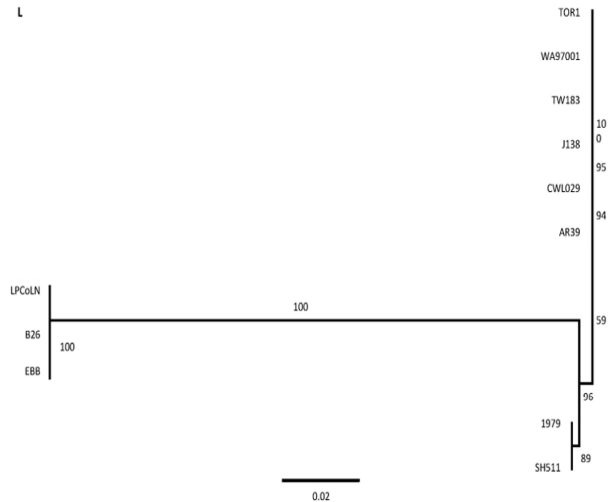

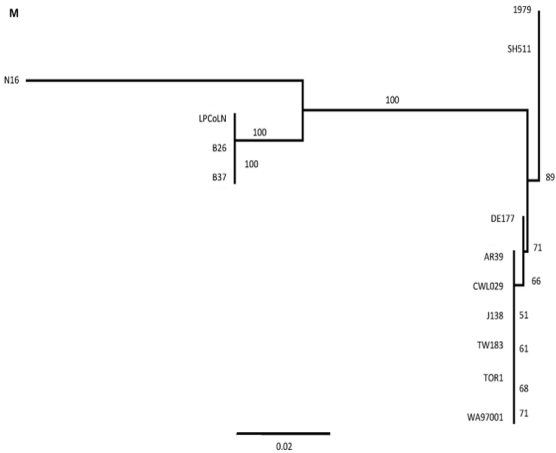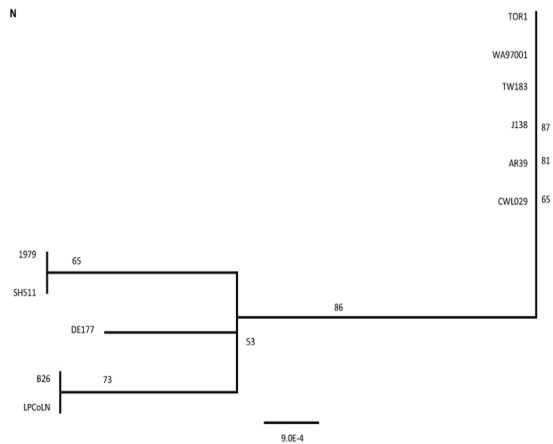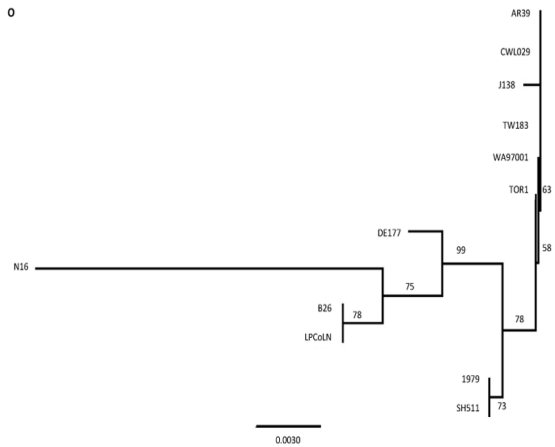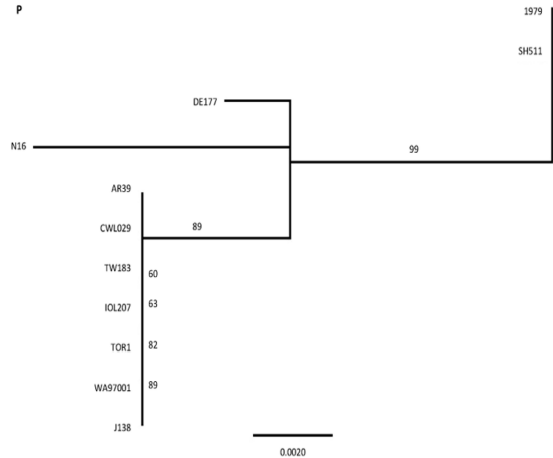

Q

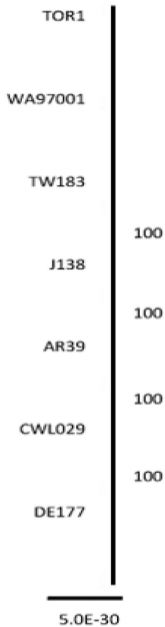

R

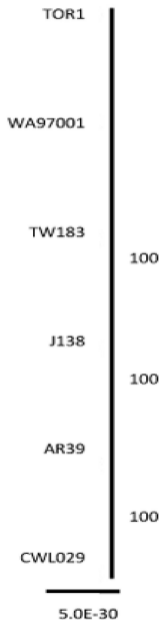

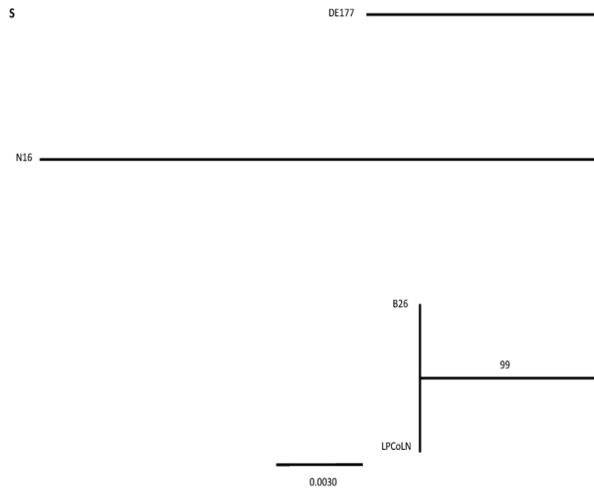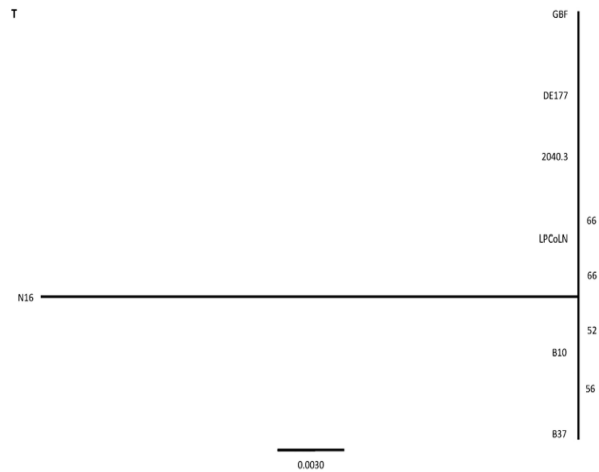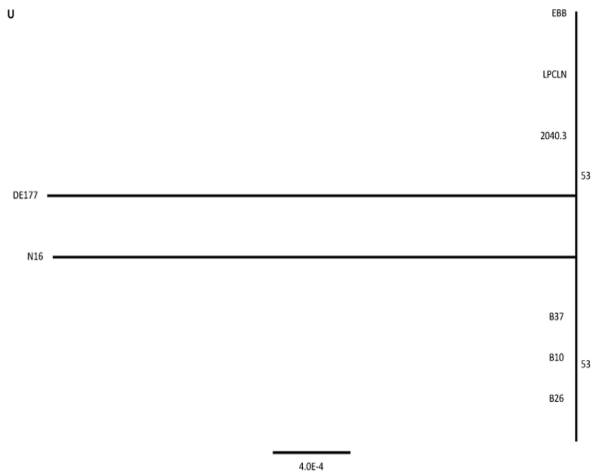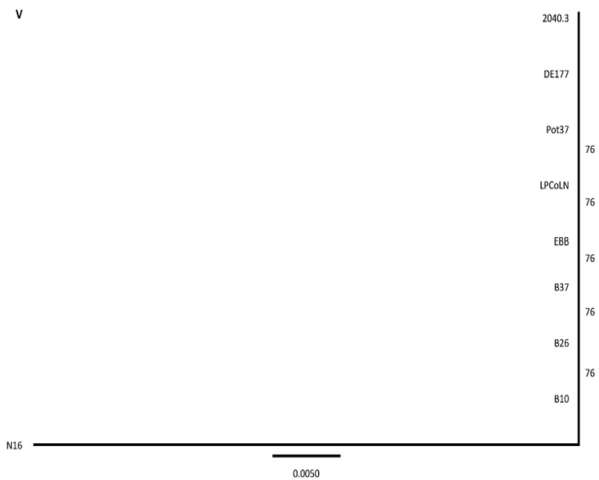

Supplement: Figure S1 — Phylogenetic trees (A-V) of C. pneumoniae isolates. Phylogenetic relationships of C. pneumoniae isolates were inferred from partial nucleotide sequences, and were constructed by Neighbor-Joining analysis and the Jukes-Cantor correction model using 1,000 bootstrap replicates. (A) CPK_ORF00679, (B) MACPF, (C) AroAA-Hs, (D) pmpE/F2, (E) pmpE/F3, (F) 16S rRNA, (G) ompA, (H) omcB, (I) accC, (J) pfk, (K) CP_1042, (L) CP_0880, (M) CP_0505, (N) ScTc, (O) HAF, (P) guaB, (Q) guaA, (R) add, (S) CPK_ORF00678, (T) SSR2, (U) helicase, and (V) PGP3D. There is evidence of five distinct phylogenetic groupings among isolates. These groupings correspond to the five proposed genotypes, A-E (Table S2). The 19/22 trees were congruent, while the phylogenetic incongruities that were observed in 3 trees (H, J and M) may be the result of host interactions or adaptation, rather than acquisition through horizontal transfer. The nomenclature for each isolate is shown in Table S1. (7.50 MB PDF) [file ppat.1000903.s001.pdf]
