# Supplementary material for: Chlamydia pneumoniae Is Genetically Diverse in Animals and Appears to Have Crossed the Host Barrier to Humans on (At Least) Two Occasions
Source: PLoS Pathog. 2010 May 20;6(5):e1000903. doi: 10.1371/journal.ppat.1000903 (PMC2873915; doi:10.1371/journal.ppat.1000903)
Supplement: Figure S2 — Multiple sequence alignment of CPK_ORF00679. Note the size variation at the 5′ end and the indel at nucleotide positions 732-746. (3.29 MB PDF) [file ppat.1000903.s002.pdf]

```
         1        10         20         30         40         50         60
Identity
B26      TTGTAGTTGT TTTATTGGGT GCTTTGCTAA TCGGAACAAT AGGTTTTTTT ATTATAAGAA
LPCoLN   TTGTAGTTGT TTTATTGGGT GCTTTGCTAA TCGGAACAAT AGGTTTTTTT ATTATAAGAA
DE177    TTGTAGTTGT TTTATTGGGT GCTTTGCTAA TCGGAACAAT AGGTTTTTTT ATTATAAGAA
N16
AR39
CWL029
J138
TW183
TOR1
WA97001
1979     TTGTAGTTGT TTTATTGGGT GCTTTGCTAA TCGGAACAAT AGGTTTTTTT ATTATAAGAA
SH511    TTGTAGTTGT TTTATTGGGT GCTTTGCTAA TCGGAACAAT AGGTTTTTTT ATTATAAGAA
```

```
                  70         80         90        100        110        120
Identity
B26      AACCTGCCGA AATTCAGACT ATCGATTCAC GATCATTAAT AGAAATCGCG AAAGCCTTTG
LPCoLN   AACCTGCCGA AATTCAGACT ATCGATTCAC GATCATTAAT AGAAATCGCG AAAGCCTTTG
DE177    AACCTGCCGA AATTCAGACT ATCGATTCAC GATCATTAAT AGAAATCGCG AAAGCCTTTG
N16              GA AATTCAGACT ATCGATTCAC GATCATTAAT AGAAATCGCG AAAGCCTTTG
AR39
CWL029
J138
TW183
TOR1
WA97001
1979     AACCTGCCGA AATTCAGACT ATCGATTCAC GATCATTAAT AGAAATCGCG AAAGCCTTTG
SH511    AACCTGCCGA AATTCAGACT ATCGATTCAC GATCATTAAT AGAAATCGCG AAAGCCTTTG
```

```
                 130        140        150        160        170        180
Identity
B26      CTTACGGTGG GAATGACTCA TTTGGGGATT CTGTTTTTGA ACTGATTGAC GAGTTAGATA
LPCoLN   CTTACGGTGG GAATGACTCA TTTGGGGATT CTGTTTTTGA ACTGATTGAC GAGTTAGATA
DE177    CTTACGGTGG GAATGACTCA TTTGGGGATT CTGTTTTTGA ACTGATTGAC GAGTTAGATA
N16      CTTACGGTGG GAATGACTCA TTTGGGGATT CTGTTTTTGA ACTGATTGAC GAGTTAGATA
AR39
CWL029
J138
TW183
TOR1
WA97001
1979     CTTACGGTGG GAATGACTCA TTTGGGGATT CTGTTTTTGA ACTGATTGAC GAGTTAGATA
SH511    CTTACGGTGG GAATGACTCA TTTGGGGATT CTGTTTTTGA ACTGATTGAC GAGTTAGATA
```

	190	200	210	220	230	240
Identity						
B26	CGACTTCTTC	TATCACTCGT	GAAGGAACTG	ATGCAGATCA	ACGTCCTATC	TTTGTGAATG
LPCoLN	CGACTTCTTC	TATCACTCGT	GAAGGAACTG	ATGCAGATCA	ACGTCCTATC	TTTGTGAATG
DE177	CGACTTCTTC	TATCACTCGT	GAAGGAACTG	ATGCAGATCA	ACGTCCTATC	TTTGTGAGTG
N16	CGACTTCTTC	TATCACTCGT	GAAGGAACTG	ATGCAGATCA	ACGTCCTATC	TTTGTGAATG
AR39						
CWL029						
J138						
TW183						
TOR1						
WA97001						
1979	CGACTTCTTC	TATCACTCGT	GAAGGAACTG	ATGCAGATCA	ACGTCCTATC	TTTGTGAATG
SH511	CGACTTCTTC	TATCACTCGT	GAAGGAACTG	ATGCAGATCA	ACGTCCTATC	TTTGTGAATG

	250	260	270	280	290	300
Identity						
B26	CTCAGGCAGA	TTTCGAGAAG	GCGATTGTAT	ATTGTATAAA	GTGTAAACAA	ATTATAAAAT
LPCoLN	CTCAGGCAGA	TTTCGAGAAG	GCGATTGTAT	ATTGTATAAA	GTGTAAACAA	ATTATAAAAT
DE177	CTCAGGCAGA	TTTCGAAAAG	GCGATTGTAT	ATTGTATAAA	GTGTAAACAA	ATTATAAAAT
N16	CTCAGGCAGA	TTTCGAGAAG	GCGATTGTAT	ATTGTATAAA	GTGTAAACAA	ATTATAAAAT
AR39		TTCGAAAAG	GCGATTGTAT	ATTGTATAAA	GTGTAAACAA	ATTATAAAAT
CWL029		TTCGAAAAG	GCGATTGTAT	ATTGTATAAA	GTGTAAACAA	ATTATAAAAT
J138		TTCGAAAAG	GCGATTGTAT	ATTGTATAAA	GTGTAAACAA	ATTATAAAAT
TW183		TTCGAAAAG	GCGATTGTAT	ATTGTATAAA	GTGTAAACAA	ATTATAAAAT
TOR1		TTCGAAAAG	GCGATTGTAT	ATTGTATAAA	GTGTAAACAA	ATTATAAAAT
WA97001		TTCGAAAAG	GCGATTGTAT	ATTGTATAAA	GTGTAAACAA	ATTATAAAAT
1979	CTCAGGCAGA	TTTCGAAAAG	GCGATTGTAT	ATTGTATAAA	GTGTAAACAA	ATTATAAAAT
SH511	CTCAGGCAGA	TTTCGAAAAG	GCGATTGTAT	ATTGTATAAA	GTGTAAACAA	ATTATAAAAT

	310	320	330	340	350	360
Identity						
B26	GTATATCGAT	CATTCATACC	CCTACACCAG	CGACACCTCT	TTGCACGGAG	GGGGAAATTT
LPCoLN	GTATATCGAT	CATTCATACC	CCTACACCAG	CGACACCTCT	TTGCACGGAG	GGGGAAATTT
DE177	GTATATCGAT	CATTCATACC	CCTACACCAG	CGACACCTCT	TTGCACGGAG	GGGGAAATTT
N16	GTATATCGAT	CATTCATACC	CCTACACCAG	CGACACCTCT	TTGCACGGAG	GGGGAAATTT
AR39	GTATATCGAT	CATTCATACC	CCTACACCAG	CGACACCTCT	TTGCACGGAG	GGGGAAATTT
CWL029	GTATATCGAT	CATTCATACC	CCTACACCAG	CGACACCTCT	TTGCACGGAG	GGGGAAATTT
J138	GTATATCGAT	CATTCATACC	CCTACACCAG	CGACACCTCT	TTGCACGGAG	GGGGAAATTT
TW183	GTATATCGAT	CATTCATACC	CCTACACCAG	CGACACCTCT	TTGCACGGAG	GGGGAAATTT
TOR1	GTATATCGAT	CATTCATACC	CCTACACCAG	CGACACCTCT	TTGCACGGAG	GGGGAAATTT
WA97001	GTATATCGAT	CATTCATACC	CCTACACCAG	CGACACCTCT	TTGCACGGAG	GGGGAAATTT
1979	GTATATCGAT	CATTCATACC	CCTACACCAG	CGACACCTCT	TTGCACGGAG	GGGGAAATTT
SH511	GTATATCGAT	CATTCATACC	CCTACACCAG	CGACACCTCT	TTGCACGGAG	GGGGAAATTT

	370	380	390	400	410	420
Identity						
B26	TTCCGGGTCT	TGTAGATTCT	GCAATTCAGA	ATGATCTCGA	GAGATTACTC	ACAGTAAAGA
LPCoLN	TTCCGGGTCT	TGTAGATTCT	GCAATTCAGA	ATGATCTCGA	GAGATTACTC	ACAGTAAAGA
DE177	TTCCAGGTCT	TGTAGATTCT	GCAATTCAGA	ATGATCTCGA	GAGATTACTC	ACAGTAAAGA
N16	TTCCGGGTCT	TGTAGATTCT	GCAATTCAGA	ATGATCTCGA	GAGATTACTC	ACAGTAAAGA
AR39	TTCCAGGTCT	TGTAGATTCT	GCAATTCAGA	ATGATCTCGA	GAGATTACTC	ACAGTAAAGA
CWL029	TTCCAGGTCC	TGTAGATTCT	GCAATTCAGA	ATGATCTCGA	GAGATTACTC	ACAGTAAAGA
J138	TTCCAGGTCT	TGTAGATTCT	GCAATTCAGA	ATGATCTCGA	GAGATTACTC	ACAGTAAAGA
TW183	TTCCAGGTCT	TGTAGATTCT	GCAATTCAGA	ATGATCTCGA	GAGATTACTC	ACAGTAAAGA
TOR1	TTCCAGGTCT	TGTAGATTCT	GCAATTCAGA	ATGATCTCGA	GAGATTACTC	ACAGTAAAGA
WA97001	TTCCAGGTCT	TGTAGATTCT	GCAATTCAGA	ATGATCTCGA	GAGATTACTC	ACAGTAAAGA
1979	TTCCAGGTCT	TGTAGATTCT	GCAATTCAGA	ATGATCTCGA	GAGATTACTC	ACAGTAAAGA
SH511	TTCCAGGTCT	TGTAGATTCT	GCAATTCAGA	ATGATCTCGA	GAGATTACTC	ACAGTAAAGA

	430	440	450	460	470	480
Identity						
B26	AAAGACCTGA	CATCATCCGG	GAGTATTTGC	GAGCAGGAGG	TAGTCTTGTT	ACAACATACC
LPCoLN	AAAGACCTGA	CATCATCCGG	GAGTATTTGC	GAGCAGGAGG	TAGTCTTGTT	ACAACATACC
DE177	AAAGACCTGA	TATCATCCGG	GAGTATTTGC	GAGCAGGAGG	TAGTCTTGTT	ACAACATACC
N16	AAAGACCTGA	CATCATCCGG	GAGTATTTGC	GAGCAGGAGG	TAGTCTTGTT	ACAACATACC
AR39	AAAGACCTGA	TATCATCCGG	GAGTATTTGC	GAGCAGGAGG	TAGTCTTGTT	ACAACATACC
CWL029	AAAGACCTGA	TATCATCCGG	GAGTATTTGC	GAGCAGGAGG	TAGTCTTGTT	ACAACATACC
J138	AAAGACCTGA	TATCATCCGG	GAGTATTTGC	GAGCAGGAGG	TAGTCTTGTT	ACAACATACC
TW183	AAAGACCTGA	TATCATCCGG	GAGTATTTGC	GAGCAGGAGG	TAGTCTTGTT	ACAACATACC
TOR1	AAAGACCTGA	TATCATCCGG	GAGTATTTGC	GAGCAGGAGG	TAGTCTTGTT	ACAACATACC
WA97001	AAAGACCTGA	TATCATCCGG	GAGTATTTGC	GAGCAGGAGG	TAGTCTTGTT	ACAACATACC
1979	AAAGACCTGA	TATCATCCGG	GAGTATTTGC	GAGCAGGAGG	TAGTCTTGTT	ACAACATACC
SH511	AAAGACCTGA	TATCATCCGG	GAGTATTTGC	GAGCAGGAGG	TAGTCTTGTT	ACAACATACC

	490	500	510	520	530	540
Identity						
B26	CTAAGGAAGG	TCGGAGATTG	CGCTCCCCAG	AACAGTTAAG	AGTTCTGGAT	GATTTAGTGC
LPCoLN	CTAAGGAAGG	TCGGAGATTG	CGCTCCCCAG	AACAGTTAAG	AGTTCTGGAT	GATTTAGTGC
DE177	CTAAGGAAGG	TCGGAGATTG	CGCTCCCCAG	AACAGTTAAG	AGTTCTGGAT	GATTTAGTGC
N16	CTAAGGAAGG	TCGGAGATTG	CGCTCCCCAG	AACAGTTAAG	AGTTCTGGAT	GATTTAGTGC
AR39	CTAAGGAAGG	TCAGAGATTG	CGCTCCCCAG	AACAGTTAAG	AGTTCTGGAT	GATTTAGTGC
CWL029	CTAAGGAAGG	TCAGAGATTG	CGCTCCCCAG	AACAGTTAAG	AGTTCTGGAT	GATTTAGTGC
J138	CTAAGGAAGG	TCAGAGATTG	CGCTCCCCAG	AACAGTTAAG	AGTTCTGGAT	GATTTAGTGC
TW183	CTAAGGAAGG	TCAGAGATTG	CGCTCCCCAG	AACAGTTAAG	AGTTCTGGAT	GATTTAGTGC
TOR1	CTAAGGAAGG	TCAGAGATTG	CGCTCCCCAG	AACAGTTAAG	AGTTCTGGAT	GATTTAGTGC
WA97001	CTAAGGAAGG	TCAGAGATTG	CGCTCCCCAG	AACAGTTAAG	AGTTCTGGAT	GATTTAGTGC
1979	CTAAGGAAGG	TCAGAGATTG	CGCTCCCCAG	AACAGTTAAG	AGTTCTGGAT	GATTTAGTGC
SH511	CTAAGGAAGG	TCAGAGATTG	CGCTCCCCAG	AACAGTTAAG	AGTTCTGGAT	GATTTAGTGC

	550	560	570	580	590	600
Identity						
B26	AAAGCTATCC	AAATCACCTG	CATGCGATTG	AACTTGATTG	TGATGCAATC	CCTCAAGATT
LPCoLN	AAAGCTATCC	AAATCACCTG	CATGCGATTG	AACTTGATTG	TGATGCAATC	CCTCAAGATT
DE177	AAAGCTATCC	AAATCACCTA	CATGCGATTG	AACTTGATTG	TGATGCAATC	CCTCAAGATT
N16	AAAGCTATCC	AAATCACCTG	CATGCGATTG	AACTTGATTG	TGATGCAATC	CCTCAAGATT
AR39	AAAGCTATCC	AAATCACCTA	CATGCGATTG	AACTTGATTG	TGGTGCAATC	CCTCAAGATT
CWL029	AAAGCTATCC	AAATCACCTA	CATGCGATTG	AACTTGATTG	TGGTGCAATC	CCTCAAGATT
J138	AAAGCTATCC	AAATCACCTA	CATGCGATTG	AACTTGATTG	TGGTGCAATC	CCTCAAGATT
TW183	AAAGCTATCC	AAATCACCTA	CATGCGATTG	AACTTGATTG	TGGTGCAATC	CCTCAAGATT
TOR1	AAAGCTATCC	AAATCACCTA	CATGCGATTG	AACTTGATTG	TGGTGCAATC	CCTCAAGATT
WA97001	AAAGCTATCC	AAATCACCTA	CATGCGATTG	AACTTGATTG	TGGTGCAATC	CCTCAAGATT
1979	AAAGCTATCC	AAATCACCTA	CATGCGATTG	AACTTGATTG	TGGTGCAATC	CCTCAAGATT
SH511	AAAGCTATCC	AAATCACCTA	CATGCGATTG	AACTTGATTG	TGGTGCAATC	CCTCAAGATT

	610	620	630	640	650	660
Identity						
B26	TGATCGGAGC	TACCTATATC	ATCACGTTCG	CCGATTTTTC	CACCTATATT	CTCTCTTTAA
LPCoLN	TGATCGGAGC	TACCTATATC	ATCACGTTCG	CCGATTTTTC	CACCTATATT	CTCTCTTTAA
DE177	TGATCGGAGC	CACCTATATC	ATCACGTTCG	CCGATTTTTC	CACCTATATT	CTCTCTTTAA
N16	TGATCGGAGC	TACCTATATC	ATCACGTTCG	CCGATTTTTC	CACCTATATT	CTCTCTTTAA
AR39	TGATCGGAGC	CACCTATATC	ATCACGTTCG	CCGATTTTTC	CRCCTATATT	CTCTCTTTAA
CWL029	TGATCGGAGC	CACCTATATC	ATCACGTTCG	CCGATTTTTC	CACCTATATT	CTCTCTTTAA
J138	TGATCGGAGC	CACCTATATC	ATCACGTTCG	CCGATTTTTC	CACCTATATT	CTCTCTTTAA
TW183	TGATCGGAGC	CACCTATATC	ATCACGTTCG	CCGATTTTTC	CGCCTATATT	CTCTCTTTAA
TOR1	TGATCGGAGC	CACCTATATC	ATCACGTTCG	CCGATTTTTC	CACCTATATT	CTCTCTTTAA
WA97001	TGATCGGAGC	CACCTATATC	ATCACGTTCG	CCGATTTTTC	CACCTATATT	CTCTCTTTAA
1979	TGATCGGAGC	CACCTATATC	ATCACGTTCG	CCGATTTTTC	CACCTATATT	CTCTCTTTAA
SH511	TGATCGGAGC	CACCTATATC	ATCACGTTCG	CCGATTTTTC	CACCTATATT	CTCTCTTTAA

	670	680	690	700	710	720
Identity						
B26	GAAGCTACCA	AGCCAATTCT	CCCTCCGATG	ATACATGGGG	GATTTGGTTT	GGATCTATTG
LPCoLN	GAAGCTACCA	AGCCAATTCT	CCCTCCGATG	ATACATGGGG	GATTTGGTTT	GGATCTATTG
DE177	GAAGCTACCA	AGCCAATTCT	CCCTCCGATG	ATACATGGGG	GATTTGGTTT	GGATCTATTG
N16	GAAGCTACCA	AGCCAATTCT	CCCTCCGATG	ATACATGGGG	GATTTGGTTT	GGATCTATTG
AR39	GAAGCTACCA	AGCCAATTCT	CCCTCCGATG	ATACATGGGG	GATTTGGTTT	GGATCTATTG
CWL029	GAAGCTACCA	AGCCAATTCT	CCCTCCGATG	ATACATGGGG	GATTTGGTTT	GGATCTATTG
J138	GAAGCTACCA	AGCCAATTCT	CCCTCCGATG	ATACATGGGG	GATTTGGTTT	GGATCTATTG
TW183	GAAGCTACCA	AGCCAATTCT	CCCTCCGATG	ATACATGGGG	GATTTGGTTT	GGATCTATTG
TOR1	GAAGCTACCA	AGCCAATTCT	CCCTCCGATG	ATACATGGGG	GATTTGGTTT	GGATCTATTG
WA97001	GAAGCTACCA	AGCCAATTCT	CCCTCCGATG	ATACATGGGG	GATTTGGTTT	GGATCTATTG
1979	GAAGCTACCA	AGCCAATTCT	CCCTCCGATG	ATACATGGGG	GATTTGGTTT	GGATCTATTG
SH511	GAAGCTACCA	AGCCAATTCT	CCCTCCGATG	ATACATGGGG	GATTTGGTTT	GGATCTATTG

	730	740	750	760	770	780
Identity						
B26	ACGATCCTGT	TATAGCAGAT	CGTTTTCAAG	CAGTCATATC	ATTTTTAAAA	GATCATGGAT
LPCoLN	ACGATCCTGT	TATAGCAGAT	CGTTTTCAAG	CAGTCATATC	ATTTTTAAAA	GATCATGGAT
DE177	ACGATCCTGT	TATAGCAGAT	CGTTTTCAAG	CAGTCATATC	ATTTTTAAAA	GATCATGGAT
N16	ACGATCCTGT	TATAGCAGAT	CGTTTTCAAG	CAGTCATATC	ATTTTTAAAA	GATCATGGAT
AR39	ACGATCCTGT	T---------	------CAAG	CAGTCATATC	ATTTTTAAAA	GATCATGGAT
CWL029	ACGATCCTGT	T---------	------CAAG	CAGTCATATC	ATTTTTAAAA	GATCATGGAT
J138	ACGATCCTGT	T---------	------CAAG	CAGTCATATC	ATTTTTAAAA	GATCATGGAT
TW183	ACGATCCTGT	T---------	------CAAG	CAGTCATATC	ATTTTTAAAA	GATCATGGAT
TOR1	ACGATCCTGT	T---------	------CAAG	CAGTCATATC	ATTTTTAAAA	GATCATGGAT
WA97001	ACGATCCTGT	T---------	------CAAG	CAGTCATATC	ATTTTTAAAA	GATCATGGAT
1979	ACGATCCTGT	TATAGCAGAT	CGTTTTCAAG	CAGTCATATC	ATTTTTAAAA	GATCATGGAT
SH511	ACGATCCTGT	TATAGCAGAT	CGTTTTCAAG	CAGTCATATC	ATTTTTAAAA	GATCATGGAT

	790	800	810	820	830	833
Identity						
B26	TTGCTCTTCC	CTCGACCTTA	GCTCAAGATC	CTTTGCTTTG	TACTAACAAG	TAA
LPCoLN	TTGCTCTTCC	CTCGACCTTA	GCTCAAGATC	CTTTGCTTTG	TACTAACAAG	TAA
DE177	TTGCTCTTCC	CTCGACCTTA	GCTCAAGATC	CTTTGCTTTG	TACTAACAAG	TAA
N16	TTGCTCTTCC	CTCGACCTTA	GCTCAAGATC	CTTTGCTTTG	TACTA	
AR39	TTGCTCTTCC	CTCGACCTTA	GCTCAAGATC	CTTTGCTTTG	TACTAACAAG	TAA
CWL029	TTGCTCTTCC	CTCGACCTTA	GCTCAAGATC	CTTTGCTTTG	TACTAACAAG	TAA
J138	TTGCTCTTCC	CTCGACCTTA	GCTCAAGATC	CTTTGCTTTG	TACTAACAAG	TAA
TW183	TTGCTCTTCC	CTCGACCTTA	GCTCAAGATC	CTTTGCTTTG	TACTAACAAG	TAA
TOR1	TTGCTCTTCC	CTCGACCTTA	GCTCAAGATC	CTTTGCTTTG	TACTAACAAG	TAA
WA97001	TTGCTCTTCC	CTCGACCTTA	GCTCAAGATC	CTTTGCTTTG	TACTAACAAG	TAA
1979	TTGCTCTTCC	CTCGACCTTA	GCTCAAGATC	CTTTGCTTTG	TACTAACAAG	TAA
SH511	TTGCTCTTCC	CTCGACCTTA	GCTCAAGATC	CTTTGCTTTG	TACTAACAAG	TAA
