# Supplementary material for: Chlamydia pneumoniae Is Genetically Diverse in Animals and Appears to Have Crossed the Host Barrier to Humans on (At Least) Two Occasions
Source: PLoS Pathog. 2010 May 20;6(5):e1000903. doi: 10.1371/journal.ppat.1000903 (PMC2873915; doi:10.1371/journal.ppat.1000903)
Supplement: Figure S3 — Multiple sequence alignment of MACPF. Animal isolates have the full-length gene, whereas human isolates have an 840 bp/280 aa internal deletion. There is a seven nucleotide indel (TGATCCT) present between 1,053-1,055 bp and 1,059-1,062 bp. An additional polymorphism (270 bp deletion) was present in two Australian Indigenous isolates SH511 and 1979 between positions 1,333-1,602. (4.11 MB PDF) [file ppat.1000903.s003.pdf]

	1–10	11–20	21–30	31–40	41–50
Identity					
B26	AATAGCTTTC	CTTTAGAGGA	AGTTGCCATT	CCCATCCTAC	CAGGTTATCA
B37	AATAGCTTTC	CTTTAGAGGA	AGTTGCCATT	CCCATCCTAC	CAGGTTATCA
LPCoLN	AATAGCTTTC	CTTTAGAGGA	AGTTGCCATT	CCCATCCTAC	CAGGTTATCA
DE177	AATAGCTTTC	CTTTAGAGGA	AGTTGCCATT	CCCATCCTAC	CAGGTTATCA
AR39	AATAGCTTTC	CTTTAGAGGA	AGTTGCCATT	CCCATCCTGC	CAGGTTATCA
AR39-2	AATAGCTTTC	CTTTAGAGGA	AGTTGCCATT	CCCATCCTGC	CAGGTTATCA
CWL029	AATAGCTTTC	CTTTAGAGGA	AGTTGCCATT	CCCATCCTGC	CAGGTTATCA
J138	AATAGCTTTC	CTTTAGAGGA	AGTTGCCATT	CCCATCCTGC	CAGGTTATCA
TW183	AATAGCTTTC	CTTTAGAGGA	AGTTGCCATT	CCCATCCTGC	CAGGTTATCA
IOL207	AATAGCTTTC	CTTTAGAGGA	AGTTGCCATT	CCCATCCTGC	CAGGTTATCA
TOR1	AATAGCTTTC	CTTTAGAGGA	AGTTGCCATT	CCCATCCTGC	CAGGTTATCA
WA97001	AATAGCTTTC	CTTTAGAGGA	AGTTGCCATT	CCCATCCTGC	CAGGTTATCA
1979	AATAGCTTTC	CTTTAGAGGA	AGTTGCCATT	CCCATCCTGC	CAGGTTATCA
SH511	AATAGCTTTC	CTTTAGAGGA	AGTTGCCATT	CCCATCCTGC	CAGGTTATCA

	60	70	80	90	100
Identity					
B26	CCCTAAGTTT	TATTTATCTT	TCATAGATAG	GGACGATCAA	GGTGTCCACT
B37	CCCTAAGTTT	TATTTATCTT	TCATAGATAG	GGACGATCAA	GGTGTCCACT
LPCoLN	CCCTAAGTTT	TATTTATCTT	TCATAGATAG	GGACGATCAA	GGTGTCCACT
DE177	CCCTAAGTTT	TATTTATCTT	TCATAGATAG	GGACGATCAA	GGTGTCCACT
AR39	CCCTAAGTTT	TATTTATCTT	TCATAGATAG	GGACGATCAA	GGTGTCCACT
AR39-2	CCCTAAGTTT	TATTTATCTT	TCATAGATAG	GGACGATCAA	GGTGTCCACT
CWL029	CCCTAAGTTT	TATTTATCTT	TCATAGATAG	GGACGATCAA	GGTGTCCACT
J138	CCCTAAGTTT	TATTTATCTT	TCATAGATAG	GGACGATCAA	GGTGTCCACT
TW183	CCCTAAGTTT	TATTTATCTT	TCATAGATAG	GGACGATCAA	GGTGTCCACT
IOL207	CCCTAAGTTT	TATTTATCTT	TCATAGATAG	GGACGATCAA	GGTGTCCACT
TOR1	CCCTAAGTTT	TATTTATCTT	TCATAGATAG	GGACGATCAA	GGTGTCCACT
WA97001	CCCTAAGTTT	TATTTATCTT	TCATAGATAG	GGACGATCAA	GGTGTCCACT
1979	CCCTAAGTTT	TATTTATCTT	TCATAGATAG	GGACGATCAA	GGTGTCCACT
SH511	CCCTAAGTTT	TATTTATCTT	TCATAGATAG	GGACGATCAA	GGTGTCCACT

	110	120	130	140	150
Identity					
B26	ATGAAGTTTT	AGATGGCGTA	TTTTTAAAGA	CAGTCGCTGC	TTGTATTATA
B37	ATGAAGTTTT	AGATGGCGTA	TTTTTAAAGA	CAGTCGCTGC	TTGTATTATA
LPCoLN	ATGAAGTTTT	AGATGGCGTA	TTTTTAAAGA	CAGTCGCTGC	TTGTATTATA
DE177	ATGAAGTTTT	AGATGGCGTA	TTTTTAAAGA	CAGTCGCTGC	TTGTATTATA
AR39	ATGAAGTTTT	AGATGGCGTA	TTTTTAAAGA	CAGTCGCTGC	TTGTATTATA
AR39-2	ATGAAGTTTT	AGATGGCGTA	TTTTTAAAGA	CAGTCGCTGC	TTGTATTATA
CWL029	ATGAAGTTTT	AGATGGCGTA	TTTTTAAAGA	CAGTCGCTGC	TTGTATTATA
J138	ATGAAGTTTT	AGATGGCGTA	TTTTTAAAGA	CAGTCGCTGC	TTGTATTATA
TW183	ATGAAGTTTT	AGATGGCGTA	TTTTTAAAGA	CAGTCGCTGC	TTGTATTATA
IOL207	ATGAAGTTTT	AGATGGCGTA	TTTTTAAAGA	CAGTCGCTGC	TTGTATTATA
TOR1	ATGAAGTTTT	AGATGGCGTA	TTTTTAAAGA	CAGTCGCTGC	TTGTATTATA
WA97001	ATGAAGTTTT	AGATGGCGTA	TTTTTAAAGA	CAGTCGCTGC	TTGTATTATA
1979	ATGAAGTTTT	AGATGGCGTA	TTTTTAAAGA	CAGTCGCTGC	TTGTATTATA
SH511	ATGAAGTTTT	AGATGGCGTA	TTTTTAAAGA	CAGTCGCTGC	TTGTATTATA

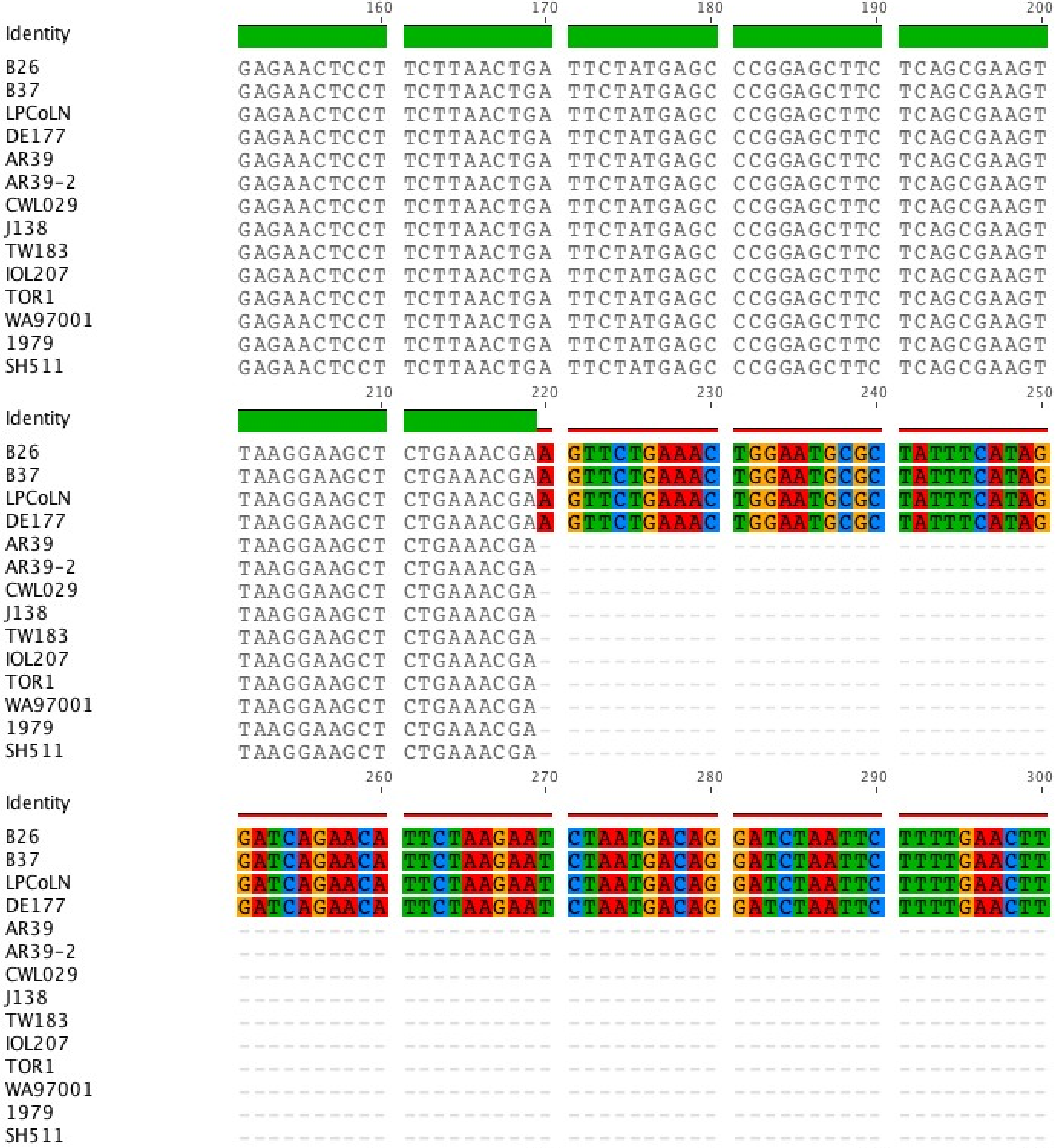

160 170 180 190 200
Identity
B26 GAGAACTCCT TCTTAACTGA TTCTATGAGC CCGGAGCTTC TCAGCGAAGT
B37 GAGAACTCCT TCTTAACTGA TTCTATGAGC CCGGAGCTTC TCAGCGAAGT
LPCoLN GAGAACTCCT TCTTAACTGA TTCTATGAGC CCGGAGCTTC TCAGCGAAGT
DE177 GAGAACTCCT TCTTAACTGA TTCTATGAGC CCGGAGCTTC TCAGCGAAGT
AR39 GAGAACTCCT TCTTAACTGA TTCTATGAGC CCGGAGCTTC TCAGCGAAGT
AR39-2 GAGAACTCCT TCTTAACTGA TTCTATGAGC CCGGAGCTTC TCAGCGAAGT
CWL029 GAGAACTCCT TCTTAACTGA TTCTATGAGC CCGGAGCTTC TCAGCGAAGT
J138 GAGAACTCCT TCTTAACTGA TTCTATGAGC CCGGAGCTTC TCAGCGAAGT
TW183 GAGAACTCCT TCTTAACTGA TTCTATGAGC CCGGAGCTTC TCAGCGAAGT
IOL207 GAGAACTCCT TCTTAACTGA TTCTATGAGC CCGGAGCTTC TCAGCGAAGT
TOR1 GAGAACTCCT TCTTAACTGA TTCTATGAGC CCGGAGCTTC TCAGCGAAGT
WA97001 GAGAACTCCT TCTTAACTGA TTCTATGAGC CCGGAGCTTC TCAGCGAAGT
1979 GAGAACTCCT TCTTAACTGA TTCTATGAGC CCGGAGCTTC TCAGCGAAGT
SH511 GAGAACTCCT TCTTAACTGA TTCTATGAGC CCGGAGCTTC TCAGCGAAGT
210 220 230 240 250
Identity
B26 TAAGGAAGCT CTGAAACGAA GTTCTGAAAC TGGAATGCGC TATTTCATAG
B37 TAAGGAAGCT CTGAAACGAA GTTCTGAAAC TGGAATGCGC TATTTCATAG
LPCoLN TAAGGAAGCT CTGAAACGAA GTTCTGAAAC TGGAATGCGC TATTTCATAG
DE177 TAAGGAAGCT CTGAAACGAA GTTCTGAAAC TGGAATGCGC TATTTCATAG
AR39 TAAGGAAGCT CTGAAACGA-
AR39-2 TAAGGAAGCT CTGAAACGA-
CWL029 TAAGGAAGCT CTGAAACGA-
J138 TAAGGAAGCT CTGAAACGA-
TW183 TAAGGAAGCT CTGAAACGA-
IOL207 TAAGGAAGCT CTGAAACGA-
TOR1 TAAGGAAGCT CTGAAACGA-
WA97001 TAAGGAAGCT CTGAAACGA-
1979 TAAGGAAGCT CTGAAACGA-
SH511 TAAGGAAGCT CTGAAACGA-
260 270 280 290 300
Identity
B26 GATCAGAACA TTCTAAGAAT CTAATGACAG GATCTAATTC TTTTGAACTT
B37 GATCAGAACA TTCTAAGAAT CTAATGACAG GATCTAATTC TTTTGAACTT
LPCoLN GATCAGAACA TTCTAAGAAT CTAATGACAG GATCTAATTC TTTTGAACTT
DE177 GATCAGAACA TTCTAAGAAT CTAATGACAG GATCTAATTC TTTTGAACTT
AR39
AR39-2
CWL029
J138
TW183
IOL207
TOR1
WA97001
1979
SH511

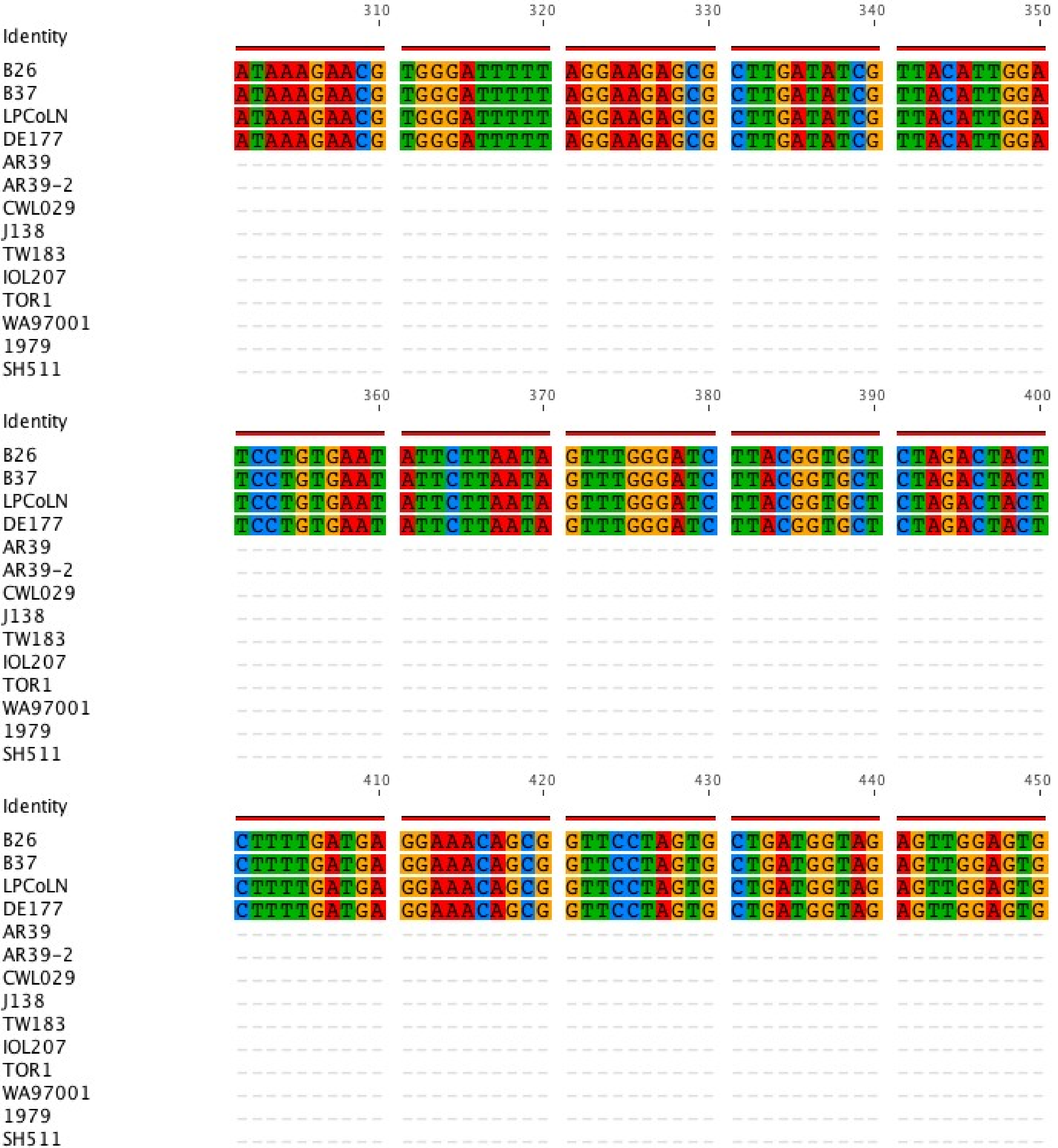
310 320 330 340 350
Identity
B26 ATAAAGAACG TGGGATTTTT AGGAAGAGCG CTTGATATCG TTACATTGGA
B37 ATAAAGAACG TGGGATTTTT AGGAAGAGCG CTTGATATCG TTACATTGGA
LPCoLN ATAAAGAACG TGGGATTTTT AGGAAGAGCG CTTGATATCG TTACATTGGA
DE177 ATAAAGAACG TGGGATTTTT AGGAAGAGCG CTTGATATCG TTACATTGGA
AR39
AR39-2
CWL029
J138
TW183
IOL207
TOR1
WA97001
1979
SH511
360 370 380 390 400
Identity
B26 TCCTGTGAAT ATTCTTAATA GTTTGGGATC TTACGGTGCT CTAGACTACT
B37 TCCTGTGAAT ATTCTTAATA GTTTGGGATC TTACGGTGCT CTAGACTACT
LPCoLN TCCTGTGAAT ATTCTTAATA GTTTGGGATC TTACGGTGCT CTAGACTACT
DE177 TCCTGTGAAT ATTCTTAATA GTTTGGGATC TTACGGTGCT CTAGACTACT
AR39
AR39-2
CWL029
J138
TW183
IOL207
TOR1
WA97001
1979
SH511
410 420 430 440 450
Identity
B26 CTTTTGATGA GGAAACAGCG GTTCCTAGTG CTGATGGTAG AGTTGGAGTG
B37 CTTTTGATGA GGAAACAGCG GTTCCTAGTG CTGATGGTAG AGTTGGAGTG
LPCoLN CTTTTGATGA GGAAACAGCG GTTCCTAGTG CTGATGGTAG AGTTGGAGTG
DE177 CTTTTGATGA GGAAACAGCG GTTCCTAGTG CTGATGGTAG AGTTGGAGTG
AR39
AR39-2
CWL029
J138
TW183
IOL207
TOR1
WA97001
1979
SH511

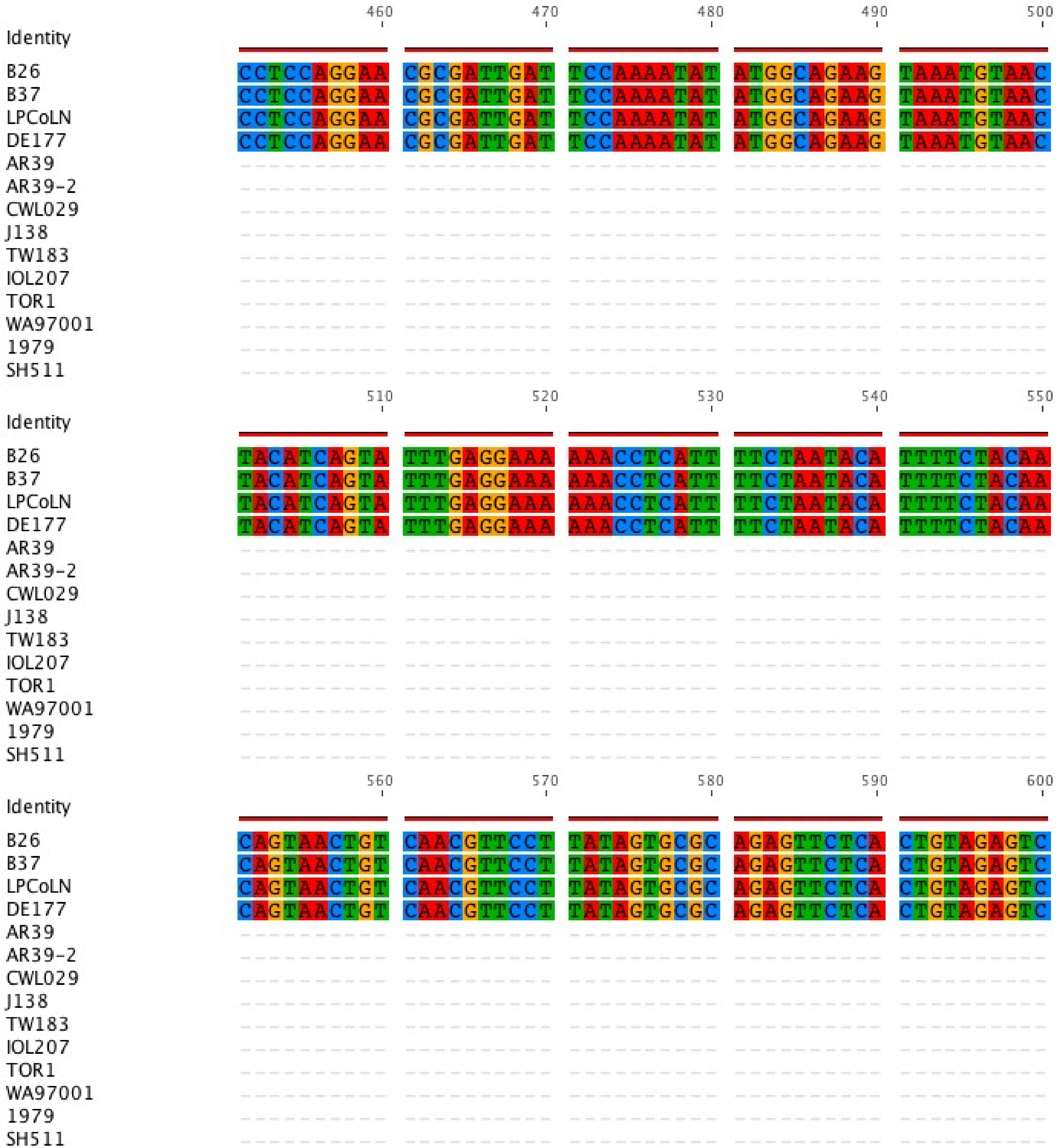

460 470 480 490 500
Identity
B26 CCTCCAGGAA CGCGATTGAT TCCAAAATAT ATGGCAGAAG TAAATGTAAC
B37 CCTCCAGGAA CGCGATTGAT TCCAAAATAT ATGGCAGAAG TAAATGTAAC
LPCoLN CCTCCAGGAA CGCGATTGAT TCCAAAATAT ATGGCAGAAG TAAATGTAAC
DE177 CCTCCAGGAA CGCGATTGAT TCCAAAATAT ATGGCAGAAG TAAATGTAAC
AR39
AR39-2
CWL029
J138
TW183
IOL207
TOR1
WA97001
1979
SH511
510 520 530 540 550
Identity
B26 TACATCAGTA TTTGAGGAAA AAACCTCATT TTCTAATACA TTTTCTACAA
B37 TACATCAGTA TTTGAGGAAA AAACCTCATT TTCTAATACA TTTTCTACAA
LPCoLN TACATCAGTA TTTGAGGAAA AAACCTCATT TTCTAATACA TTTTCTACAA
DE177 TACATCAGTA TTTGAGGAAA AAACCTCATT TTCTAATACA TTTTCTACAA
AR39
AR39-2
CWL029
J138
TW183
IOL207
TOR1
WA97001
1979
SH511
560 570 580 590 600
Identity
B26 CAGTAACTGT CAACGTTCCT TATAGTGCGC AGAGTTCTCA CTGTAGAGTC
B37 CAGTAACTGT CAACGTTCCT TATAGTGCGC AGAGTTCTCA CTGTAGAGTC
LPCoLN CAGTAACTGT CAACGTTCCT TATAGTGCGC AGAGTTCTCA CTGTAGAGTC
DE177 CAGTAACTGT CAACGTTCCT TATAGTGCGC AGAGTTCTCA CTGTAGAGTC
AR39
AR39-2
CWL029
J138
TW183
IOL207
TOR1
WA97001
1979
SH511

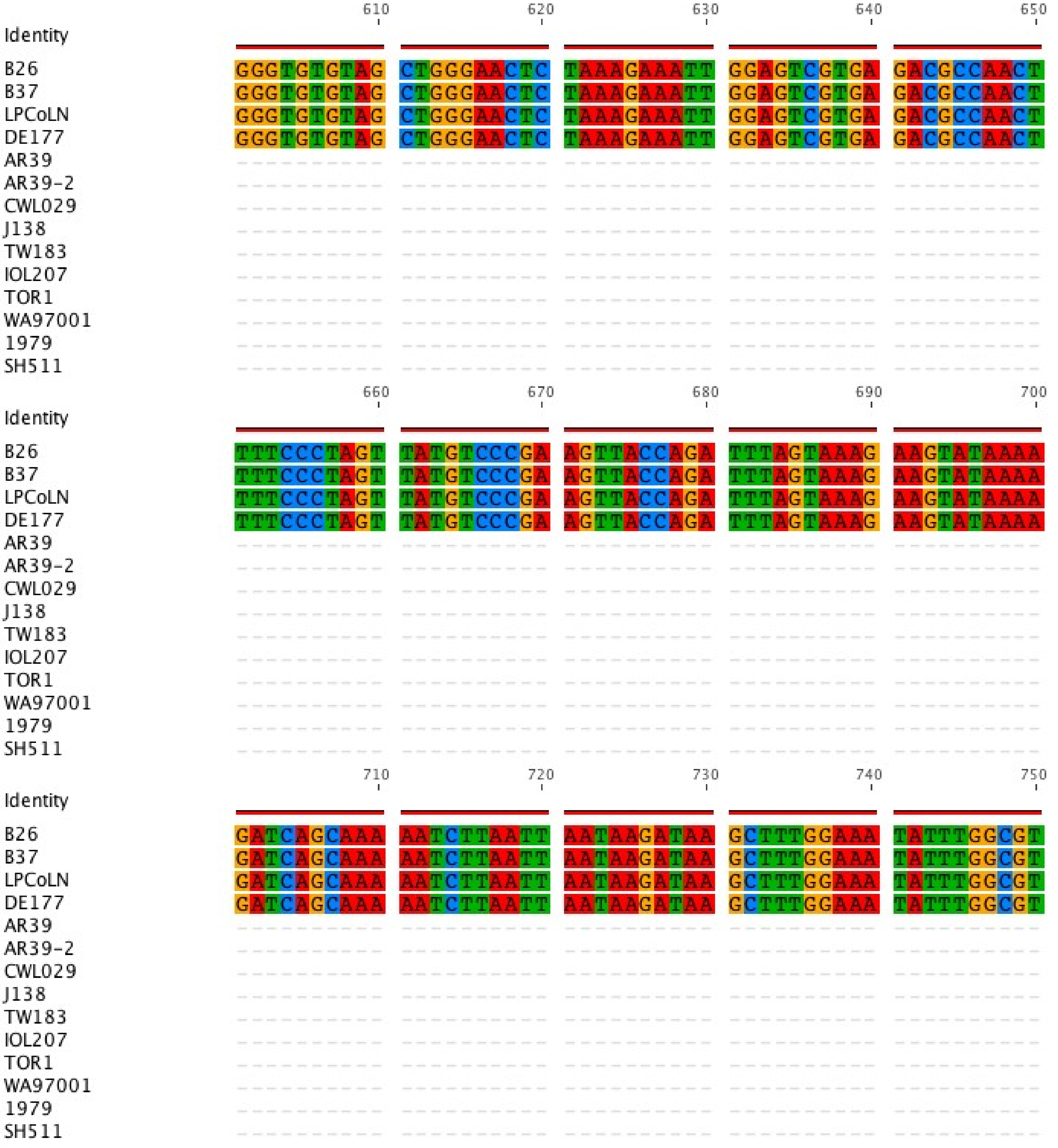
610 620 630 640 650
Identity
B26 GGGTGTGTAG CTGGGAACTC TAAAGAAATT GGAGTCGTGA GACGCCAACT
B37 GGGTGTGTAG CTGGGAACTC TAAAGAAATT GGAGTCGTGA GACGCCAACT
LPCoLN GGGTGTGTAG CTGGGAACTC TAAAGAAATT GGAGTCGTGA GACGCCAACT
DE177 GGGTGTGTAG CTGGGAACTC TAAAGAAATT GGAGTCGTGA GACGCCAACT
AR39
AR39-2
CWL029
J138
TW183
IOL207
TOR1
WA97001
1979
SH511
660 670 680 690 700
Identity
B26 TTTCCCTAGT TATGTCCCGA AGTTACCAGA TTTAGTAAAG AAGTATAAAA
B37 TTTCCCTAGT TATGTCCCGA AGTTACCAGA TTTAGTAAAG AAGTATAAAA
LPCoLN TTTCCCTAGT TATGTCCCGA AGTTACCAGA TTTAGTAAAG AAGTATAAAA
DE177 TTTCCCTAGT TATGTCCCGA AGTTACCAGA TTTAGTAAAG AAGTATAAAA
AR39
AR39-2
CWL029
J138
TW183
IOL207
TOR1
WA97001
1979
SH511
710 720 730 740 750
Identity
B26 GATCAGCAAA AATCTTAATT AATAAGATAA GCTTTGGAAA TATTTGGCGT
B37 GATCAGCAAA AATCTTAATT AATAAGATAA GCTTTGGAAA TATTTGGCGT
LPCoLN GATCAGCAAA AATCTTAATT AATAAGATAA GCTTTGGAAA TATTTGGCGT
DE177 GATCAGCAAA AATCTTAATT AATAAGATAA GCTTTGGAAA TATTTGGCGT
AR39
AR39-2
CWL029
J138
TW183
IOL207
TOR1
WA97001
1979
SH511

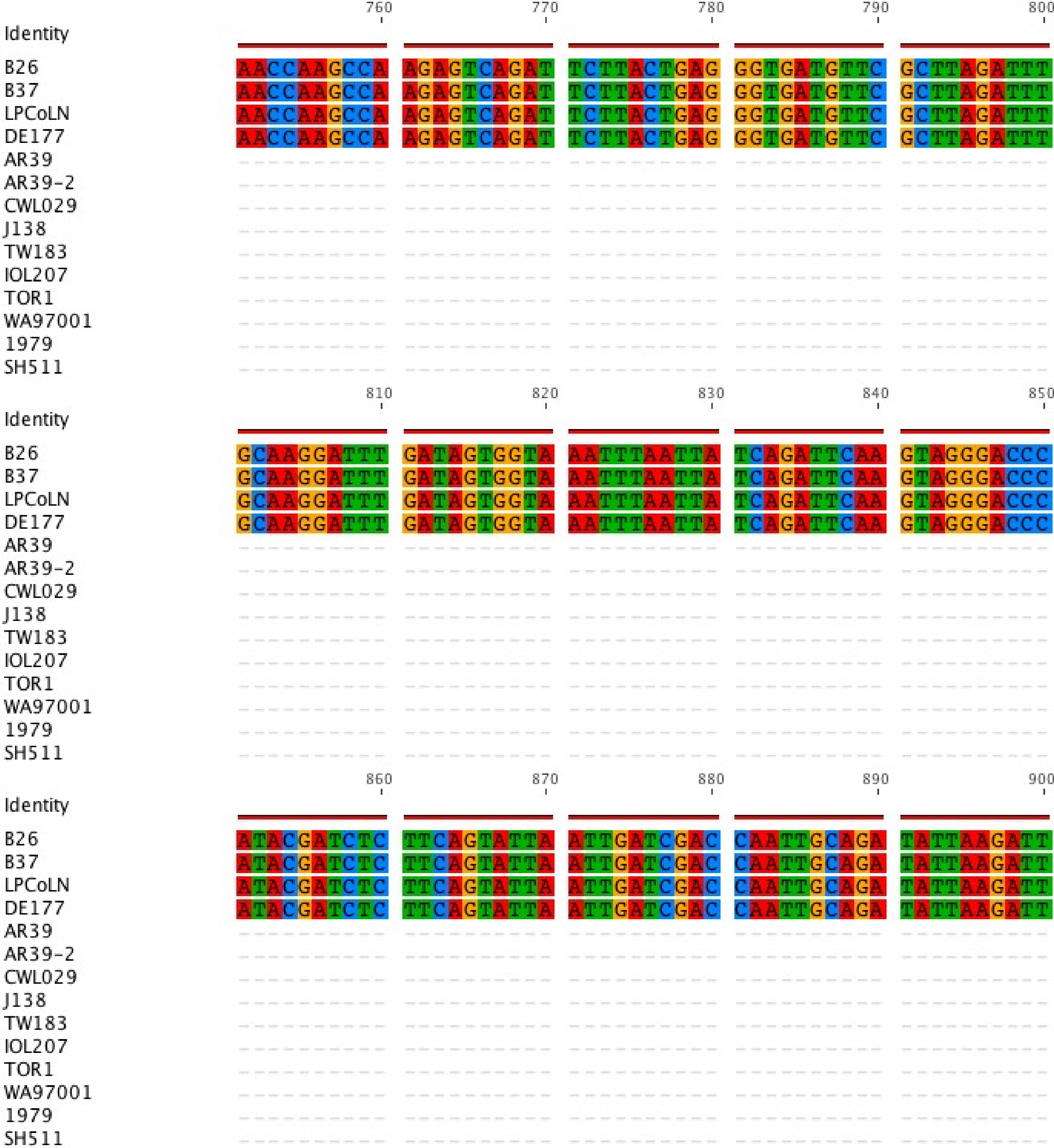

760 770 780 790 800
Identity
B26 AACCAAGCCA AGAGTCAGAT TCTTACTGAG GGTGATGTTC GCTTAGATTT
B37 AACCAAGCCA AGAGTCAGAT TCTTACTGAG GGTGATGTTC GCTTAGATTT
LPCoLN AACCAAGCCA AGAGTCAGAT TCTTACTGAG GGTGATGTTC GCTTAGATTT
DE177 AACCAAGCCA AGAGTCAGAT TCTTACTGAG GGTGATGTTC GCTTAGATTT
AR39
AR39-2
CWL029
J138
TW183
IOL207
TOR1
WA97001
1979
SH511
810 820 830 840 850
Identity
B26 GCAAGGATTT GATAGTGGTA AATTTAATTA TCAGATTCAA GTAGGGACCC
B37 GCAAGGATTT GATAGTGGTA AATTTAATTA TCAGATTCAA GTAGGGACCC
LPCoLN GCAAGGATTT GATAGTGGTA AATTTAATTA TCAGATTCAA GTAGGGACCC
DE177 GCAAGGATTT GATAGTGGTA AATTTAATTA TCAGATTCAA GTAGGGACCC
AR39
AR39-2
CWL029
J138
TW183
IOL207
TOR1
WA97001
1979
SH511
860 870 880 890 900
Identity
B26 ATACGATCTC TTCAGTATTA ATTGATCGAC CAATTGCAGA TATTAAGATT
B37 ATACGATCTC TTCAGTATTA ATTGATCGAC CAATTGCAGA TATTAAGATT
LPCoLN ATACGATCTC TTCAGTATTA ATTGATCGAC CAATTGCAGA TATTAAGATT
DE177 ATACGATCTC TTCAGTATTA ATTGATCGAC CAATTGCAGA TATTAAGATT
AR39
AR39-2
CWL029
J138
TW183
IOL207
TOR1
WA97001
1979
SH511

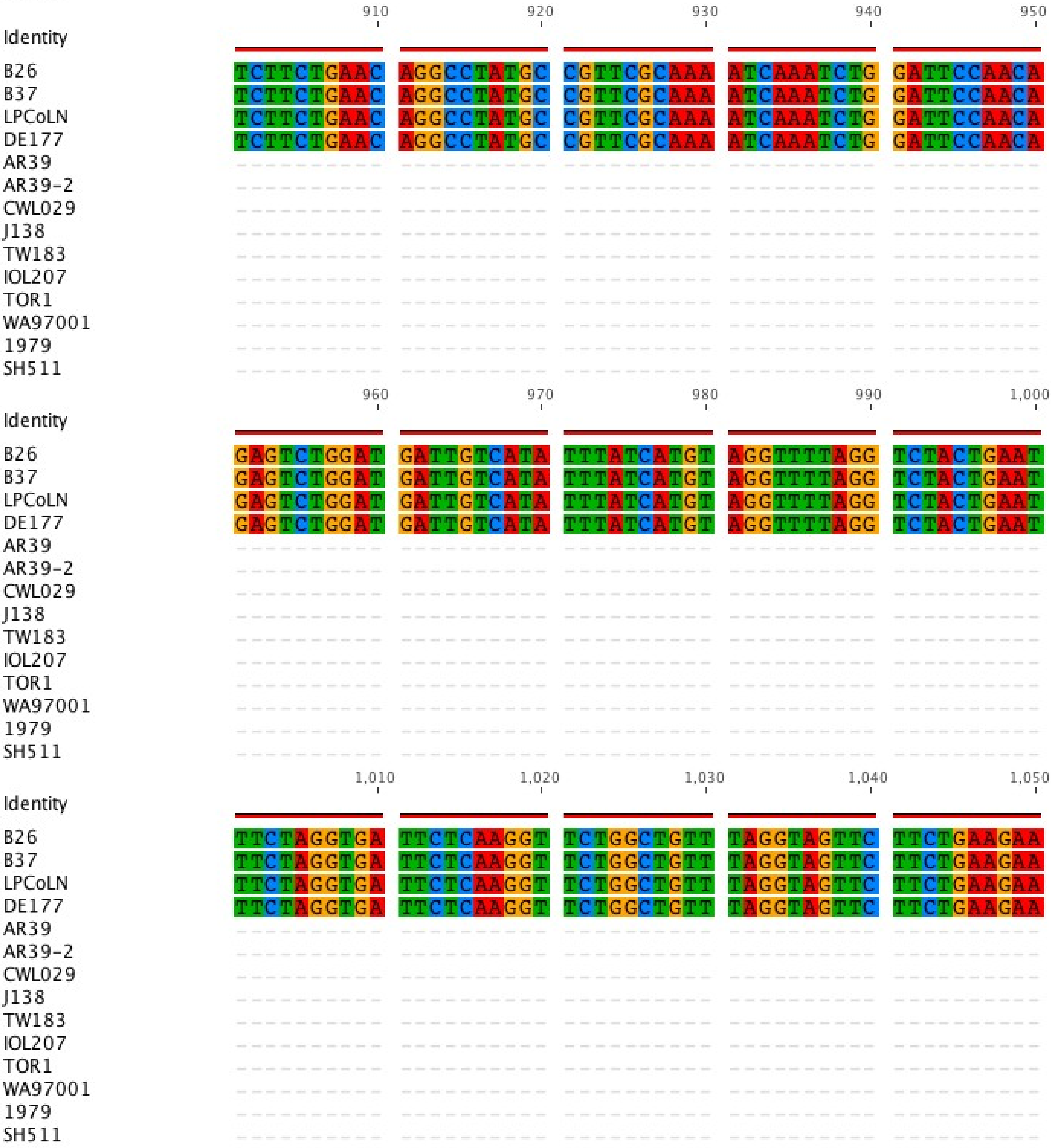

910
920
930
940
950
Identity
B26 TCTTCTGAAC AGGCCTATGC CGTTCGCAAA ATCAAATCTG GATTCCAACA
B37 TCTTCTGAAC AGGCCTATGC CGTTCGCAAA ATCAAATCTG GATTCCAACA
LPCoLN TCTTCTGAAC AGGCCTATGC CGTTCGCAAA ATCAAATCTG GATTCCAACA
DE177 TCTTCTGAAC AGGCCTATGC CGTTCGCAAA ATCAAATCTG GATTCCAACA
AR39
AR39-2
CWL029
J138
TW183
IOL207
TOR1
WA97001
1979
SH511
960
970
980
990
1,000
Identity
B26 GAGTCTGGAT GATTGTCATA TTTATCATGT AGGTTTTAGG TCTACTGAAT
B37 GAGTCTGGAT GATTGTCATA TTTATCATGT AGGTTTTAGG TCTACTGAAT
LPCoLN GAGTCTGGAT GATTGTCATA TTTATCATGT AGGTTTTAGG TCTACTGAAT
DE177 GAGTCTGGAT GATTGTCATA TTTATCATGT AGGTTTTAGG TCTACTGAAT
AR39
AR39-2
CWL029
J138
TW183
IOL207
TOR1
WA97001
1979
SH511
1,010
1,020
1,030
1,040
1,050
Identity
B26 TTCTAGGTGA TTCTCAAGGT TCTGGCTGTT TAGGTAGTTC TTCTGAAGAA
B37 TTCTAGGTGA TTCTCAAGGT TCTGGCTGTT TAGGTAGTTC TTCTGAAGAA
LPCoLN TTCTAGGTGA TTCTCAAGGT TCTGGCTGTT TAGGTAGTTC TTCTGAAGAA
DE177 TTCTAGGTGA TTCTCAAGGT TCTGGCTGTT TAGGTAGTTC TTCTGAAGAA
AR39
AR39-2
CWL029
J138
TW183
IOL207
TOR1
WA97001
1979
SH511

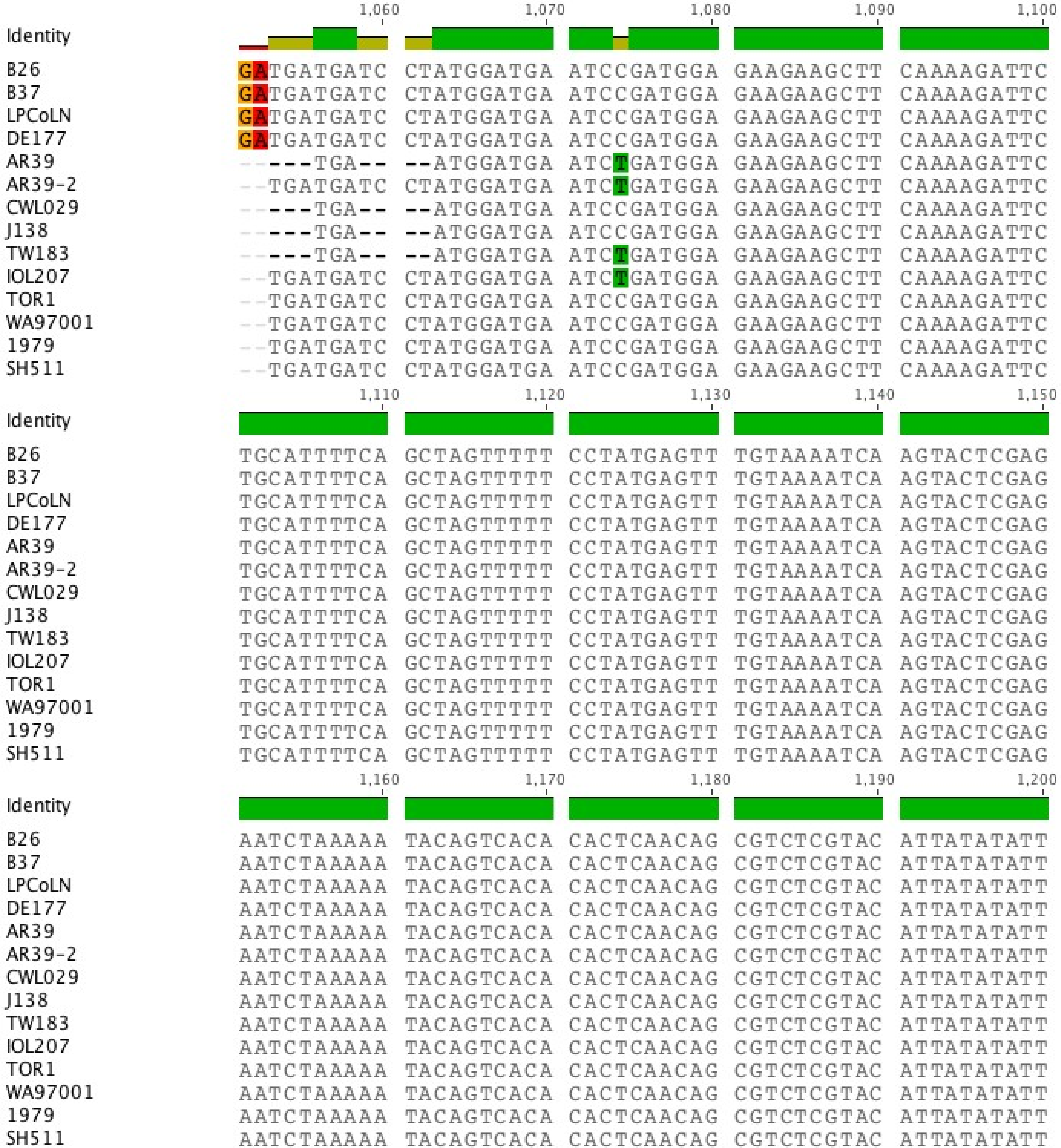

1,060
1,070
1,080
1,090
1,100
Identity
B26 GATGATGATC CTATGGATGA ATCCGATGGA GAAGAAGCTT CAAAAGATTC
B37 GATGATGATC CTATGGATGA ATCCGATGGA GAAGAAGCTT CAAAAGATTC
LPCoLN GATGATGATC CTATGGATGA ATCCGATGGA GAAGAAGCTT CAAAAGATTC
DE177 GATGATGATC CTATGGATGA ATCCGATGGA GAAGAAGCTT CAAAAGATTC
AR39 -----TGA-- --ATGGATGA ATCTGATGGA GAAGAAGCTT CAAAAGATTC
AR39-2 --TGATGATC CTATGGATGA ATCTGATGGA GAAGAAGCTT CAAAAGATTC
CWL029 -----TGA-- --ATGGATGA ATCCGATGGA GAAGAAGCTT CAAAAGATTC
J138 -----TGA-- --ATGGATGA ATCCGATGGA GAAGAAGCTT CAAAAGATTC
TW183 -----TGA-- --ATGGATGA ATCTGATGGA GAAGAAGCTT CAAAAGATTC
IOL207 --TGATGATC CTATGGATGA ATCTGATGGA GAAGAAGCTT CAAAAGATTC
TOR1 --TGATGATC CTATGGATGA ATCCGATGGA GAAGAAGCTT CAAAAGATTC
WA97001 --TGATGATC CTATGGATGA ATCCGATGGA GAAGAAGCTT CAAAAGATTC
1979 --TGATGATC CTATGGATGA ATCCGATGGA GAAGAAGCTT CAAAAGATTC
SH511 --TGATGATC CTATGGATGA ATCCGATGGA GAAGAAGCTT CAAAAGATTC
1,110
1,120
1,130
1,140
1,150
Identity
B26 TGCATTTTCA GCTAGTTTTT CCTATGAGTT TGTAAAATCA AGTACTCGAG
B37 TGCATTTTCA GCTAGTTTTT CCTATGAGTT TGTAAAATCA AGTACTCGAG
LPCoLN TGCATTTTCA GCTAGTTTTT CCTATGAGTT TGTAAAATCA AGTACTCGAG
DE177 TGCATTTTCA GCTAGTTTTT CCTATGAGTT TGTAAAATCA AGTACTCGAG
AR39 TGCATTTTCA GCTAGTTTTT CCTATGAGTT TGTAAAATCA AGTACTCGAG
AR39-2 TGCATTTTCA GCTAGTTTTT CCTATGAGTT TGTAAAATCA AGTACTCGAG
CWL029 TGCATTTTCA GCTAGTTTTT CCTATGAGTT TGTAAAATCA AGTACTCGAG
J138 TGCATTTTCA GCTAGTTTTT CCTATGAGTT TGTAAAATCA AGTACTCGAG
TW183 TGCATTTTCA GCTAGTTTTT CCTATGAGTT TGTAAAATCA AGTACTCGAG
IOL207 TGCATTTTCA GCTAGTTTTT CCTATGAGTT TGTAAAATCA AGTACTCGAG
TOR1 TGCATTTTCA GCTAGTTTTT CCTATGAGTT TGTAAAATCA AGTACTCGAG
WA97001 TGCATTTTCA GCTAGTTTTT CCTATGAGTT TGTAAAATCA AGTACTCGAG
1979 TGCATTTTCA GCTAGTTTTT CCTATGAGTT TGTAAAATCA AGTACTCGAG
SH511 TGCATTTTCA GCTAGTTTTT CCTATGAGTT TGTAAAATCA AGTACTCGAG
1,160
1,170
1,180
1,190
1,200
Identity
B26 AATCTAAAAA TACAGTCACA CACTCAACAG CGTCTCGTAC ATTATATATT
B37 AATCTAAAAA TACAGTCACA CACTCAACAG CGTCTCGTAC ATTATATATT
LPCoLN AATCTAAAAA TACAGTCACA CACTCAACAG CGTCTCGTAC ATTATATATT
DE177 AATCTAAAAA TACAGTCACA CACTCAACAG CGTCTCGTAC ATTATATATT
AR39 AATCTAAAAA TACAGTCACA CACTCAACAG CGTCTCGTAC ATTATATATT
AR39-2 AATCTAAAAA TACAGTCACA CACTCAACAG CGTCTCGTAC ATTATATATT
CWL029 AATCTAAAAA TACAGTCACA CACTCAACAG CGTCTCGTAC ATTATATATT
J138 AATCTAAAAA TACAGTCACA CACTCAACAG CGTCTCGTAC ATTATATATT
TW183 AATCTAAAAA TACAGTCACA CACTCAACAG CGTCTCGTAC ATTATATATT
IOL207 AATCTAAAAA TACAGTCACA CACTCAACAG CGTCTCGTAC ATTATATATT
TOR1 AATCTAAAAA TACAGTCACA CACTCAACAG CGTCTCGTAC ATTATATATT
WA97001 AATCTAAAAA TACAGTCACA CACTCAACAG CGTCTCGTAC ATTATATATT
1979 AATCTAAAAA TACAGTCACA CACTCAACAG CGTCTCGTAC ATTATATATT
SH511 AATCTAAAAA TACAGTCACA CACTCAACAG CGTCTCGTAC ATTATATATT

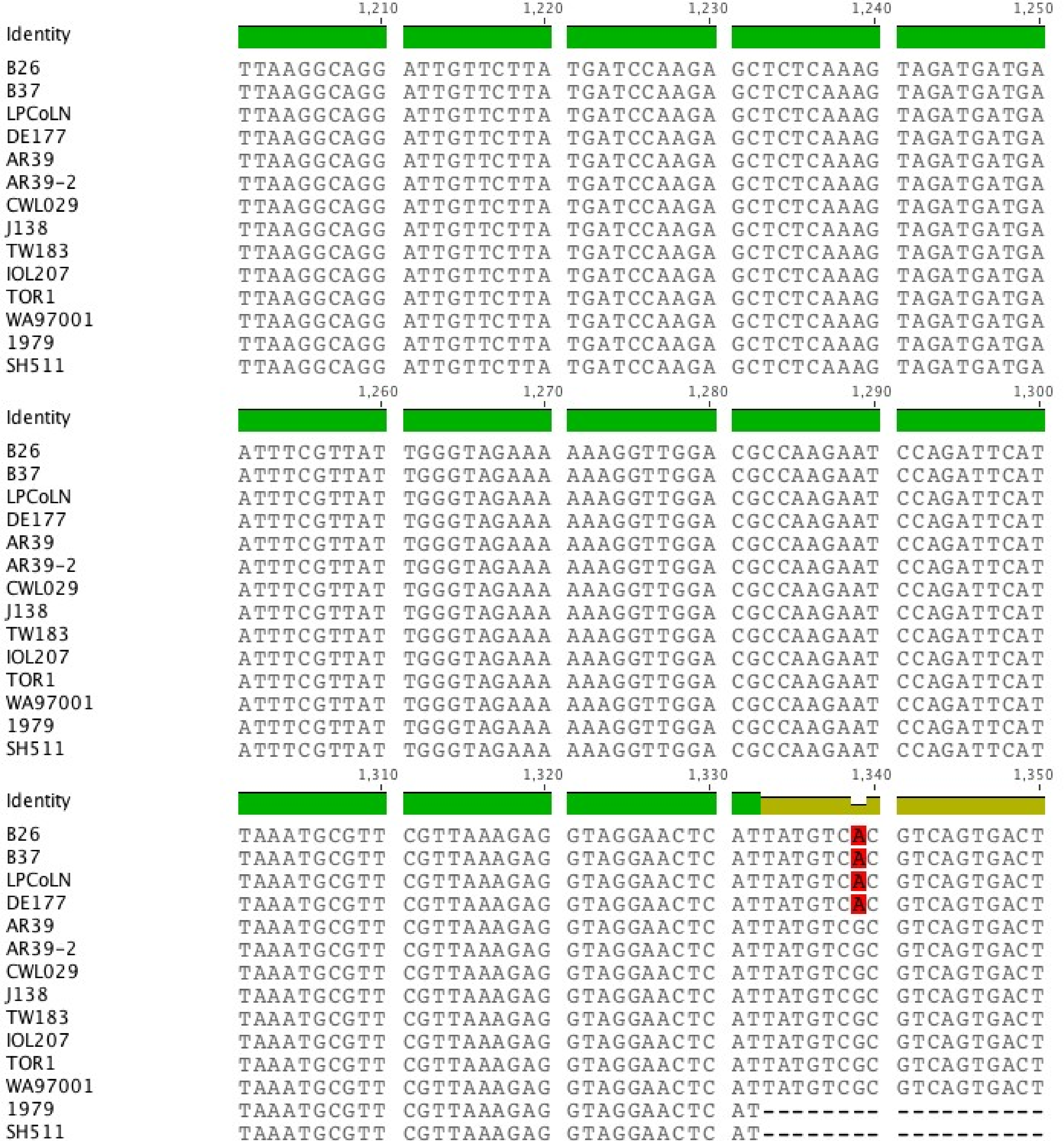
1,210 1,220 1,230 1,240 1,250
Identity
B26 TTAAGGCAGG ATTGTTCTTA TGATCCAAGA GCTCTCAAAG TAGATGATGA
B37 TTAAGGCAGG ATTGTTCTTA TGATCCAAGA GCTCTCAAAG TAGATGATGA
LPCoLN TTAAGGCAGG ATTGTTCTTA TGATCCAAGA GCTCTCAAAG TAGATGATGA
DE177 TTAAGGCAGG ATTGTTCTTA TGATCCAAGA GCTCTCAAAG TAGATGATGA
AR39 TTAAGGCAGG ATTGTTCTTA TGATCCAAGA GCTCTCAAAG TAGATGATGA
AR39-2 TTAAGGCAGG ATTGTTCTTA TGATCCAAGA GCTCTCAAAG TAGATGATGA
CWL029 TTAAGGCAGG ATTGTTCTTA TGATCCAAGA GCTCTCAAAG TAGATGATGA
J138 TTAAGGCAGG ATTGTTCTTA TGATCCAAGA GCTCTCAAAG TAGATGATGA
TW183 TTAAGGCAGG ATTGTTCTTA TGATCCAAGA GCTCTCAAAG TAGATGATGA
IOL207 TTAAGGCAGG ATTGTTCTTA TGATCCAAGA GCTCTCAAAG TAGATGATGA
TOR1 TTAAGGCAGG ATTGTTCTTA TGATCCAAGA GCTCTCAAAG TAGATGATGA
WA97001 TTAAGGCAGG ATTGTTCTTA TGATCCAAGA GCTCTCAAAG TAGATGATGA
1979 TTAAGGCAGG ATTGTTCTTA TGATCCAAGA GCTCTCAAAG TAGATGATGA
SH511 TTAAGGCAGG ATTGTTCTTA TGATCCAAGA GCTCTCAAAG TAGATGATGA
1,260 1,270 1,280 1,290 1,300
Identity
B26 ATTTCGTTAT TGGGTAGAAA AAAGGTTGGA CGCCAAGAAT CCAGATTCAT
B37 ATTTCGTTAT TGGGTAGAAA AAAGGTTGGA CGCCAAGAAT CCAGATTCAT
LPCoLN ATTTCGTTAT TGGGTAGAAA AAAGGTTGGA CGCCAAGAAT CCAGATTCAT
DE177 ATTTCGTTAT TGGGTAGAAA AAAGGTTGGA CGCCAAGAAT CCAGATTCAT
AR39 ATTTCGTTAT TGGGTAGAAA AAAGGTTGGA CGCCAAGAAT CCAGATTCAT
AR39-2 ATTTCGTTAT TGGGTAGAAA AAAGGTTGGA CGCCAAGAAT CCAGATTCAT
CWL029 ATTTCGTTAT TGGGTAGAAA AAAGGTTGGA CGCCAAGAAT CCAGATTCAT
J138 ATTTCGTTAT TGGGTAGAAA AAAGGTTGGA CGCCAAGAAT CCAGATTCAT
TW183 ATTTCGTTAT TGGGTAGAAA AAAGGTTGGA CGCCAAGAAT CCAGATTCAT
IOL207 ATTTCGTTAT TGGGTAGAAA AAAGGTTGGA CGCCAAGAAT CCAGATTCAT
TOR1 ATTTCGTTAT TGGGTAGAAA AAAGGTTGGA CGCCAAGAAT CCAGATTCAT
WA97001 ATTTCGTTAT TGGGTAGAAA AAAGGTTGGA CGCCAAGAAT CCAGATTCAT
1979 ATTTCGTTAT TGGGTAGAAA AAAGGTTGGA CGCCAAGAAT CCAGATTCAT
SH511 ATTTCGTTAT TGGGTAGAAA AAAGGTTGGA CGCCAAGAAT CCAGATTCAT
1,310 1,320 1,330 1,340 1,350
Identity
B26 TAAATGCGTT CGTTAAAGAG GTAGGAACTC ATTATGTCAC GTCAGTGACT
B37 TAAATGCGTT CGTTAAAGAG GTAGGAACTC ATTATGTCAC GTCAGTGACT
LPCoLN TAAATGCGTT CGTTAAAGAG GTAGGAACTC ATTATGTCAC GTCAGTGACT
DE177 TAAATGCGTT CGTTAAAGAG GTAGGAACTC ATTATGTCAC GTCAGTGACT
AR39 TAAATGCGTT CGTTAAAGAG GTAGGAACTC ATTATGTCGC GTCAGTGACT
AR39-2 TAAATGCGTT CGTTAAAGAG GTAGGAACTC ATTATGTCGC GTCAGTGACT
CWL029 TAAATGCGTT CGTTAAAGAG GTAGGAACTC ATTATGTCGC GTCAGTGACT
J138 TAAATGCGTT CGTTAAAGAG GTAGGAACTC ATTATGTCGC GTCAGTGACT
TW183 TAAATGCGTT CGTTAAAGAG GTAGGAACTC ATTATGTCGC GTCAGTGACT
IOL207 TAAATGCGTT CGTTAAAGAG GTAGGAACTC ATTATGTCGC GTCAGTGACT
TOR1 TAAATGCGTT CGTTAAAGAG GTAGGAACTC ATTATGTCGC GTCAGTGACT
WA97001 TAAATGCGTT CGTTAAAGAG GTAGGAACTC ATTATGTCGC GTCAGTGACT
1979 TAAATGCGTT CGTTAAAGAG GTAGGAACTC AT-------- ----------
SH511 TAAATGCGTT CGTTAAAGAG GTAGGAACTC AT-------- ----------

```
                                           1,360           1,370           1,380           1,390           1,400
Identity
B26        TATGGTGGCA TTGGTTTTCA AGTGCTAAAG ATGTCTTATC TCCAAGTCGA
B37        TATGGTGGCA TTGGTTTTCA AGTGCTAAAG ATGTCTTATC TCCAAGTCGA
LPCoLN     TATGGTGGCA TTGGTTTTCA AGTGCTAAAG ATGTCTTATC TCCAAGTCGA
DE177      TATGGTGGCA TTGGTTTTCA AGTGCTAAAG ATGTCTTATC TCCAAGTCGA
AR39       TACGGTGGCA TTGGTTTTCA AGTGCTAAAG ATGTCTTATC TCCAAGTCGA
AR39-2     TACGGTGGCA TTGGTTTTCA AGTGCTAAAG ATGTCTTATC TCCAAGTCGA
CWL029     TACGGTGGCA TTGGTTTTCA AGTGCTAAAG ATGTCTTATC TCCAAGTCGA
J138       TACGGTGGCA TTGGTTTTCA AGTGCTAAAG ATGTCTTATC TCCAAGTCGA
TW183      TACGGTGGCA TTGGTTTTCA AGTGCTAAAG ATGTCTTATC TCCAAGTCGA
IOL207     TACGGTGGCA TTGGTTTTCA AGTGCTAAAG ATGTCTTATC TCCAAGTCGA
TOR1       TACGGTGGCA TTGGTTTTCA AGTGCTAAAG ATGTCTTATC TCCAAGTCGA
WA97001    TACGGTGGCA TTGGTTTTCA AGTGCTAAAG ATGTCTTATC TCCAAGTCGA
1979       ---------- ---------- ---------- ---------- ----------
SH511      ---------- ---------- ---------- ---------- ----------

                                           1,410           1,420           1,430           1,440           1,450
Identity
B26        GGAGTTAGAG AAAGAAAAAA TCTCGATATC TGTAGCTGCA GCAAGTTCTT
B37        GGAGTTAGAG AAAGAAAAAA TCTCGATATC TGTAGCTGCA GCAAGTTCTT
LPCoLN     GGAGTTAGAG AAAGAAAAAA TCTCGATATC TGTAGCTGCA GCAAGTTCTT
DE177      GGAGTTAGAG AAAGAAAAAA TCTCGATATC TGTAGCTGCA GCAAGTTCTT
AR39       GGAGTTAGAG AAAGAAAAAA TCTCGATATC TGTAGCTGCA GCAAGTTCTT
AR39-2     GGAGTTAGAG AAAGAAAAAA TCTCGATATC TGTAGCTGCA GCAAGTTCTT
CWL029     GGAGTTAGAG AAAGAAAAAA TCTCGATATC TGTAGCTGCA GCAAGTTCTT
J138       GGAGTTAGAG AAAGAAAAAA TCTCGATATC TGTAGCTGCA GCAAGTTCTT
TW183      GGAGTTAGAG AAAGAAAAAA TCTCGATATC TGTAGCTGCA GCAAGTTCTT
IOL207     GGAGTTAGAG AAAGAAAAAA TCTCGATATC TGTAGCTGCA GCAAGTTCTT
TOR1       GGAGTTAGAG AAAGAAAAAA TCTCGATATC TGTAGCTGCA GCAAGTTCTT
WA97001    GGAGTTAGAG AAAGAAAAAA TCTCGATATC TGTAGCTGCA GCAAGTTCTT
1979       ---------- ---------- ---------- ---------- ----------
SH511      ---------- ---------- ---------- ---------- ----------

                                           1,460           1,470           1,480           1,490           1,500
Identity
B26        TATTAAAAAG TAAAACATCG AACGCGACAG AGAAAGGTTA TTCTTCGTAT
B37        TATTAAAAAG TAAAACATCG AACGCGACAG AGAAAGGTTA TTCTTCGTAT
LPCoLN     TATTAAAAAG TAAAACATCG AACGCGACAG AGAAAGGTTA TTCTTCGTAT
DE177      TATTAAAAAG TAAAACATCG AACGCGACAG AGAAAGGTTA TTCTTCGTAT
AR39       TATTAAAAAG TAAAACATCG AACGCGACAG AGAAAGGTTA TTCTTCGTAT
AR39-2     TATTAAAAAG TAAAACATCG AACGCGACAG AGAAAGGTTA TTCTTCGTAT
CWL029     TATTAAAAAG TAAAACATCG AACGCGACAG AGAAAGGTTA TTCTTCGTAT
J138       TATTAAAAAG TAAAACATCG AACGCGACAG AGAAAGGTTA TTCTTCGTAT
TW183      TATTAAAAAG TAAAACATCG AACGCGACAG AGAAAGGTTA TTCTTCGTAT
IOL207     TATTAAAAAG TAAAACATCG AACGCGACAG AGAAAGGTTA TTCTTCGTAT
TOR1       TATTAAAAAG TAAAACATCG AACGCGACAG AGAAAGGTTA TTCTTCGTAT
WA97001    TATTAAAAAG TAAAACATCG AACGCGACAG AGAAAGGTTA TTCTTCGTAT
1979       ---------- ---------- ---------- ---------- ----------
SH511      ---------- ---------- ---------- ---------- ----------
```

	1,510	1,520	1,530	1,540	1,550
Identity					
B26	CAGTCGGAAT	CATCAGCTCA	AACAGTATTT	CTTGGTGGAA	CAGTATTACC
B37	CAGTCGGAAT	CATCAGCTCA	AACAGTATTT	CTTGGTGGAA	CAGTATTACC
LPCoLN	CAGTCGGAAT	CATCAGCTCA	AACAGTATTT	CTTGGTGGAA	CAGTATTACC
DE177	CAGTCGGAAT	CATCAGCTCA	AACAGTATTT	CTTGGTGGAA	CAGTATTACC
AR39	CAGTCGGAAT	CATCAGCTCA	AACAGTATTT	CTTGGTGGAA	CAGTATTACC
AR39-2	CAGTCGGAAT	CATCAGCTCA	AACAGTATTT	CTTGGTGGAA	CAGTATTACC
CWL029	CAGTCGGAAT	CATCAGCTCA	AACAGTATTT	CTTGGTGGAA	CAGTATTACC
J138	CAGTCGGAAT	CATCAGCTCA	AACAGTATTT	CTTGGTGGAA	CAGTATTACC
TW183	CAGTCGGAAT	CATCAGCTCA	AACAGTATTT	CTTGGTGGAA	CAGTATTACC
IOL207	CAGTCGGAAT	CATCAGCTCA	AACAGTATTT	CTTGGTGGAA	CAGTATTACC
TOR1	CAGTCGGAAT	CATCAGCTCA	AACAGTATTT	CTTGGTGGAA	CAGTATTACC
WA97001	CAGTCGGAAT	CATCAGCTCA	AACAGTATTT	CTTGGTGGAA	CAGTATTACC
1979	----------	----------	----------	----------	----------
SH511	----------	----------	----------	----------	----------

	1,560	1,570	1,580	1,590	1,600
Identity					
B26	TGATCTCCAG	CAAGACAAGT	TGGATTTCAA	AGATTGGTCT	GAAAGCGTTC
B37	TGATCTCCAG	CAAGACAAGT	TGGATTTCAA	AGATTGGTCT	GAAAGCGTTC
LPCoLN	TGATCTCCAG	CAAGACAAGT	TGGATTTCAA	AGATTGGTCT	GAAAGCGTTC
DE177	TGATCTCCAG	CAAGACAAGT	TGGATTTCAA	AGATTGGTCT	GAAAGCGTTC
AR39	TGATCTCCAG	CAAGACAAGT	TGGATTTCAA	AGATTGGTCT	GAAAGCATTC
AR39-2	TGATCTCCAG	CAAGACAAGT	TGGATTTCAA	AGATTGGTCT	GAAAGCATTC
CWL029	TGATCTCCAG	CAAGACAAGT	TGGATTTCAA	AGATTGGTCT	GAAAGCATTC
J138	TGATCTCCAG	CAAGACAAGT	TGGATTTCAA	AGATTGGTCT	GAAAGCATTC
TW183	TGATCTCCAG	CAAGACAAGT	TGGATTTCAA	AGATTGGTCT	GAAAGCATTC
IOL207	TGATCTCCAG	CAAGACAAGT	TGGATTTCAA	AGATTGGTCT	GAAAGCATTC
TOR1	TGATCTCCAG	CAAGACAAGT	TGGATTTCAA	AGATTGGTCT	GAAAGCATTC
WA97001	TGATCTCCAG	CAAGACAAGT	TGGATTTCAA	AGATTGGTCT	GAAAGCATTC
1979	----------	----------	----------	----------	----------
SH511	----------	----------	----------	----------	----------

	1,610	1,620	1,630	1,640	1,650
Identity					
B26	CTAATGAGCC	CATTCCTCTA	GCTATTAGTG	TATCTTCAAT	TACAGATCTC
B37	CTAATGAGCC	CATTCCTCTA	GCTATTAGTG	TATCTTCAAT	TACAGATCTC
LPCoLN	CTAATGAGCC	CATTCCTCTA	GCTATTAGTG	TATCTTCAAT	TACAGATCTC
DE177	CTAATGAGCC	CATTCCTCTA	GCTATTAGTG	TATCTTCAAT	TACAGATCTC
AR39	CTAATGAGCC	CATTCCTCTA	GCTATTAGTG	TATCTTCAAT	TACAGATCTC
AR39-2	CTAATGAGCC	CATTCCTCTA	GCTATTAGTG	TATCTTCAAT	TACAGATCTC
CWL029	CTAATGAGCC	CATTCCTCTA	GCTATTAGTG	TATCTTCAAT	TACAGATCTC
J138	CTAATGAGCC	CATTCCTCTA	GCTATTAGTG	TATCTTCAAT	TACAGATCTC
TW183	CTAATGAGCC	CATTCCTCTA	GCTATTAGTG	TATCTTCAAT	TACAGATCTC
IOL207	CTAATGAGCC	CATTCCTCTA	GCTATTAGTG	TATCTTCAAT	TACAGATCTC
TOR1	CTAATGAGCC	CATTCCTCTA	GCTATTAGTG	TATCTTCAAT	TACAGATCTC
WA97001	CTAATGAGCC	CATTCCTCTA	GCTATTAGTG	TATCTTCAAT	TACAGATCTC
1979	--AATGAGCC	CATTCCTCTA	GCTATTAGTG	TATCTTCAAT	TACAGATCTC
SH511	--AATGAGCC	CATTCCTCTA	GCTATTAGTG	TATCTTCAAT	TACAGATCTC

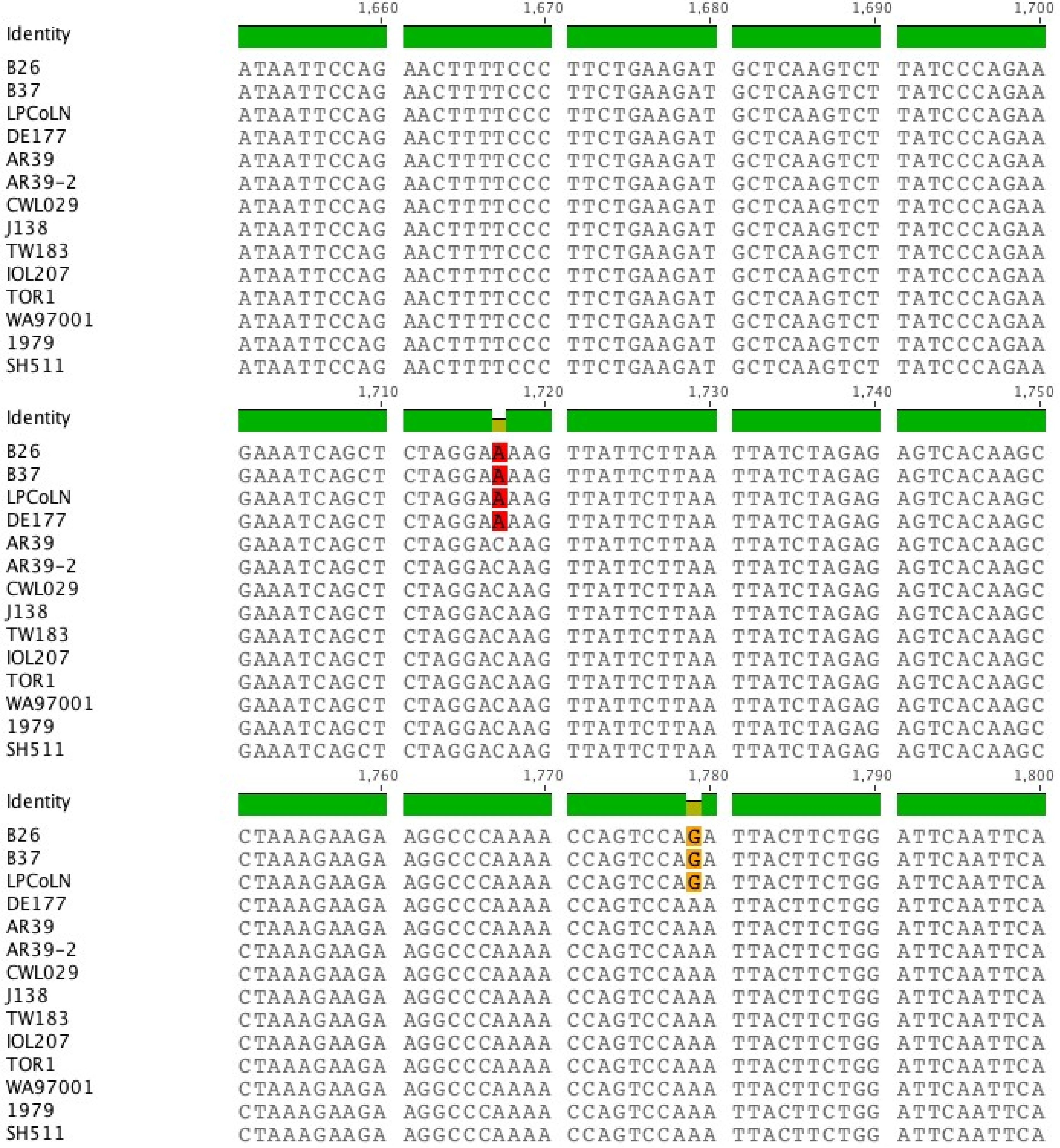
1,660 1,670 1,680 1,690 1,700
Identity
B26 ATAATTCCAG AACTTTTCCC TTCTGAAGAT GCTCAAGTCT TATCCCAGAA
B37 ATAATTCCAG AACTTTTCCC TTCTGAAGAT GCTCAAGTCT TATCCCAGAA
LPCoLN ATAATTCCAG AACTTTTCCC TTCTGAAGAT GCTCAAGTCT TATCCCAGAA
DE177 ATAATTCCAG AACTTTTCCC TTCTGAAGAT GCTCAAGTCT TATCCCAGAA
AR39 ATAATTCCAG AACTTTTCCC TTCTGAAGAT GCTCAAGTCT TATCCCAGAA
AR39-2 ATAATTCCAG AACTTTTCCC TTCTGAAGAT GCTCAAGTCT TATCCCAGAA
CWL029 ATAATTCCAG AACTTTTCCC TTCTGAAGAT GCTCAAGTCT TATCCCAGAA
J138 ATAATTCCAG AACTTTTCCC TTCTGAAGAT GCTCAAGTCT TATCCCAGAA
TW183 ATAATTCCAG AACTTTTCCC TTCTGAAGAT GCTCAAGTCT TATCCCAGAA
IOL207 ATAATTCCAG AACTTTTCCC TTCTGAAGAT GCTCAAGTCT TATCCCAGAA
TOR1 ATAATTCCAG AACTTTTCCC TTCTGAAGAT GCTCAAGTCT TATCCCAGAA
WA97001 ATAATTCCAG AACTTTTCCC TTCTGAAGAT GCTCAAGTCT TATCCCAGAA
1979 ATAATTCCAG AACTTTTCCC TTCTGAAGAT GCTCAAGTCT TATCCCAGAA
SH511 ATAATTCCAG AACTTTTCCC TTCTGAAGAT GCTCAAGTCT TATCCCAGAA
1,710 1,720 1,730 1,740 1,750
Identity
B26 GAAATCAGCT CTAGGAAAAG TTATTCTTAA TTATCTAGAG AGTCACAAGC
B37 GAAATCAGCT CTAGGAAAAG TTATTCTTAA TTATCTAGAG AGTCACAAGC
LPCoLN GAAATCAGCT CTAGGAAAAG TTATTCTTAA TTATCTAGAG AGTCACAAGC
DE177 GAAATCAGCT CTAGGAAAAG TTATTCTTAA TTATCTAGAG AGTCACAAGC
AR39 GAAATCAGCT CTAGGACAAG TTATTCTTAA TTATCTAGAG AGTCACAAGC
AR39-2 GAAATCAGCT CTAGGACAAG TTATTCTTAA TTATCTAGAG AGTCACAAGC
CWL029 GAAATCAGCT CTAGGACAAG TTATTCTTAA TTATCTAGAG AGTCACAAGC
J138 GAAATCAGCT CTAGGACAAG TTATTCTTAA TTATCTAGAG AGTCACAAGC
TW183 GAAATCAGCT CTAGGACAAG TTATTCTTAA TTATCTAGAG AGTCACAAGC
IOL207 GAAATCAGCT CTAGGACAAG TTATTCTTAA TTATCTAGAG AGTCACAAGC
TOR1 GAAATCAGCT CTAGGACAAG TTATTCTTAA TTATCTAGAG AGTCACAAGC
WA97001 GAAATCAGCT CTAGGACAAG TTATTCTTAA TTATCTAGAG AGTCACAAGC
1979 GAAATCAGCT CTAGGACAAG TTATTCTTAA TTATCTAGAG AGTCACAAGC
SH511 GAAATCAGCT CTAGGACAAG TTATTCTTAA TTATCTAGAG AGTCACAAGC
1,760 1,770 1,780 1,790 1,800
Identity
B26 CTAAAGAAGA AGGCCCAAAA CCAGTCCAGA TTACTTCTGG ATTCAATTCA
B37 CTAAAGAAGA AGGCCCAAAA CCAGTCCAGA TTACTTCTGG ATTCAATTCA
LPCoLN CTAAAGAAGA AGGCCCAAAA CCAGTCCAGA TTACTTCTGG ATTCAATTCA
DE177 CTAAAGAAGA AGGCCCAAAA CCAGTCCAAA TTACTTCTGG ATTCAATTCA
AR39 CTAAAGAAGA AGGCCCAAAA CCAGTCCAAA TTACTTCTGG ATTCAATTCA
AR39-2 CTAAAGAAGA AGGCCCAAAA CCAGTCCAAA TTACTTCTGG ATTCAATTCA
CWL029 CTAAAGAAGA AGGCCCAAAA CCAGTCCAAA TTACTTCTGG ATTCAATTCA
J138 CTAAAGAAGA AGGCCCAAAA CCAGTCCAAA TTACTTCTGG ATTCAATTCA
TW183 CTAAAGAAGA AGGCCCAAAA CCAGTCCAAA TTACTTCTGG ATTCAATTCA
IOL207 CTAAAGAAGA AGGCCCAAAA CCAGTCCAAA TTACTTCTGG ATTCAATTCA
TOR1 CTAAAGAAGA AGGCCCAAAA CCAGTCCAAA TTACTTCTGG ATTCAATTCA
WA97001 CTAAAGAAGA AGGCCCAAAA CCAGTCCAAA TTACTTCTGG ATTCAATTCA
1979 CTAAAGAAGA AGGCCCAAAA CCAGTCCAAA TTACTTCTGG ATTCAATTCA
SH511 CTAAAGAAGA AGGCCCAAAA CCAGTCCAAA TTACTTCTGG ATTCAATTCA

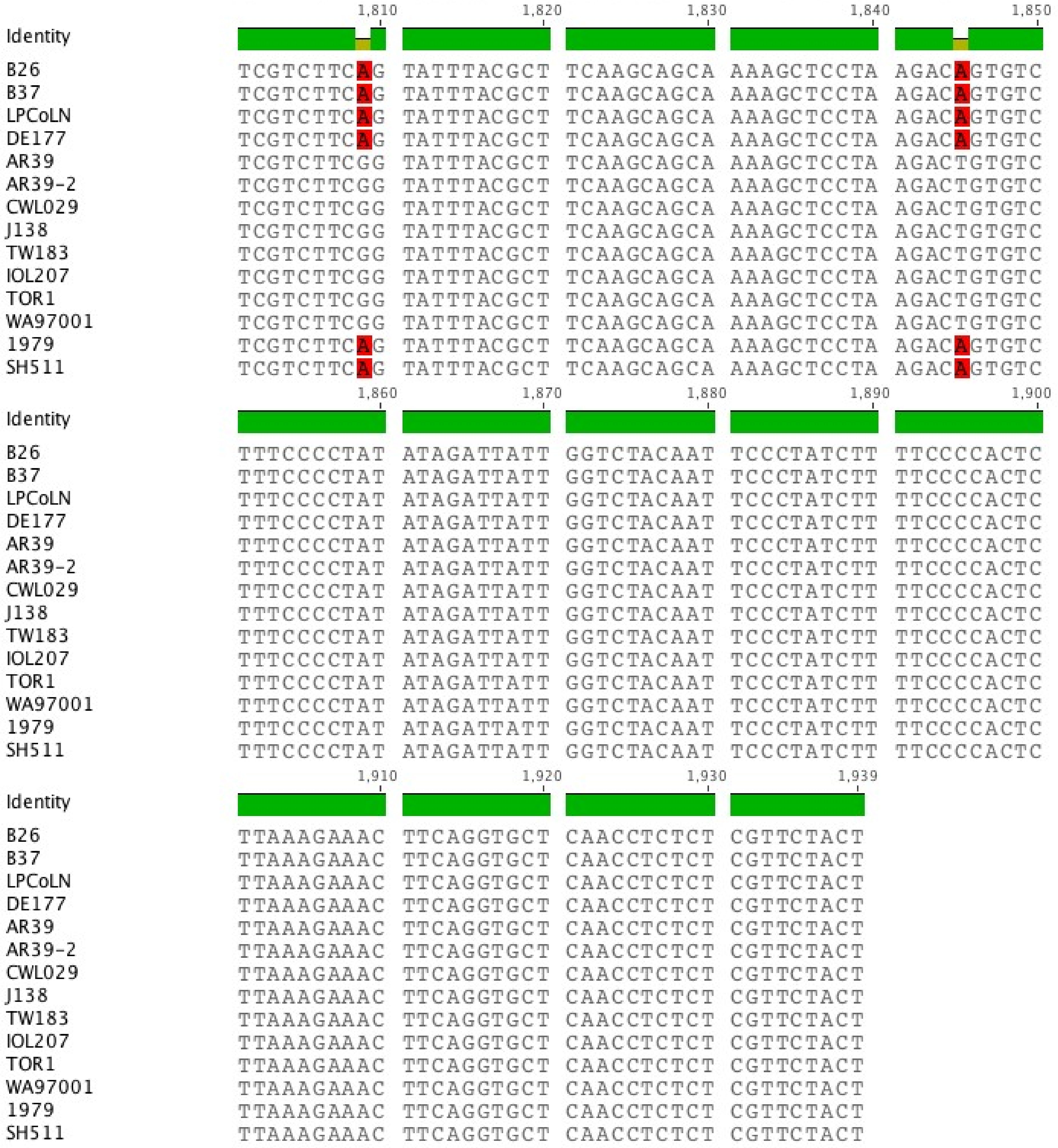
1,810 1,820 1,830 1,840 1,850
Identity
B26 TCGTCTTCAG TATTTACGCT TCAAGCAGCA AAAGCTCCTA AGACAGTGTC
B37 TCGTCTTCAG TATTTACGCT TCAAGCAGCA AAAGCTCCTA AGACAGTGTC
LPCoLN TCGTCTTCAG TATTTACGCT TCAAGCAGCA AAAGCTCCTA AGACAGTGTC
DE177 TCGTCTTCAG TATTTACGCT TCAAGCAGCA AAAGCTCCTA AGACAGTGTC
AR39 TCGTCTTCGG TATTTACGCT TCAAGCAGCA AAAGCTCCTA AGACTGTGTC
AR39-2 TCGTCTTCGG TATTTACGCT TCAAGCAGCA AAAGCTCCTA AGACTGTGTC
CWL029 TCGTCTTCGG TATTTACGCT TCAAGCAGCA AAAGCTCCTA AGACTGTGTC
J138 TCGTCTTCGG TATTTACGCT TCAAGCAGCA AAAGCTCCTA AGACTGTGTC
TW183 TCGTCTTCGG TATTTACGCT TCAAGCAGCA AAAGCTCCTA AGACTGTGTC
IOL207 TCGTCTTCGG TATTTACGCT TCAAGCAGCA AAAGCTCCTA AGACTGTGTC
TOR1 TCGTCTTCGG TATTTACGCT TCAAGCAGCA AAAGCTCCTA AGACTGTGTC
WA97001 TCGTCTTCGG TATTTACGCT TCAAGCAGCA AAAGCTCCTA AGACTGTGTC
1979 TCGTCTTCAG TATTTACGCT TCAAGCAGCA AAAGCTCCTA AGACAGTGTC
SH511 TCGTCTTCAG TATTTACGCT TCAAGCAGCA AAAGCTCCTA AGACAGTGTC
1,860 1,870 1,880 1,890 1,900
Identity
B26 TTTCCCCTAT ATAGATTATT GGTCTACAAT TCCCTATCTT TTCCCCACTC
B37 TTTCCCCTAT ATAGATTATT GGTCTACAAT TCCCTATCTT TTCCCCACTC
LPCoLN TTTCCCCTAT ATAGATTATT GGTCTACAAT TCCCTATCTT TTCCCCACTC
DE177 TTTCCCCTAT ATAGATTATT GGTCTACAAT TCCCTATCTT TTCCCCACTC
AR39 TTTCCCCTAT ATAGATTATT GGTCTACAAT TCCCTATCTT TTCCCCACTC
AR39-2 TTTCCCCTAT ATAGATTATT GGTCTACAAT TCCCTATCTT TTCCCCACTC
CWL029 TTTCCCCTAT ATAGATTATT GGTCTACAAT TCCCTATCTT TTCCCCACTC
J138 TTTCCCCTAT ATAGATTATT GGTCTACAAT TCCCTATCTT TTCCCCACTC
TW183 TTTCCCCTAT ATAGATTATT GGTCTACAAT TCCCTATCTT TTCCCCACTC
IOL207 TTTCCCCTAT ATAGATTATT GGTCTACAAT TCCCTATCTT TTCCCCACTC
TOR1 TTTCCCCTAT ATAGATTATT GGTCTACAAT TCCCTATCTT TTCCCCACTC
WA97001 TTTCCCCTAT ATAGATTATT GGTCTACAAT TCCCTATCTT TTCCCCACTC
1979 TTTCCCCTAT ATAGATTATT GGTCTACAAT TCCCTATCTT TTCCCCACTC
SH511 TTTCCCCTAT ATAGATTATT GGTCTACAAT TCCCTATCTT TTCCCCACTC
1,910 1,920 1,930 1,939
Identity
B26 TTAAAGAAAC TTCAGGTGCT CAACCTCTCT CGTTCTACT
B37 TTAAAGAAAC TTCAGGTGCT CAACCTCTCT CGTTCTACT
LPCoLN TTAAAGAAAC TTCAGGTGCT CAACCTCTCT CGTTCTACT
DE177 TTAAAGAAAC TTCAGGTGCT CAACCTCTCT CGTTCTACT
AR39 TTAAAGAAAC TTCAGGTGCT CAACCTCTCT CGTTCTACT
AR39-2 TTAAAGAAAC TTCAGGTGCT CAACCTCTCT CGTTCTACT
CWL029 TTAAAGAAAC TTCAGGTGCT CAACCTCTCT CGTTCTACT
J138 TTAAAGAAAC TTCAGGTGCT CAACCTCTCT CGTTCTACT
TW183 TTAAAGAAAC TTCAGGTGCT CAACCTCTCT CGTTCTACT
IOL207 TTAAAGAAAC TTCAGGTGCT CAACCTCTCT CGTTCTACT
TOR1 TTAAAGAAAC TTCAGGTGCT CAACCTCTCT CGTTCTACT
WA97001 TTAAAGAAAC TTCAGGTGCT CAACCTCTCT CGTTCTACT
1979 TTAAAGAAAC TTCAGGTGCT CAACCTCTCT CGTTCTACT
SH511 TTAAAGAAAC TTCAGGTGCT CAACCTCTCT CGTTCTACT

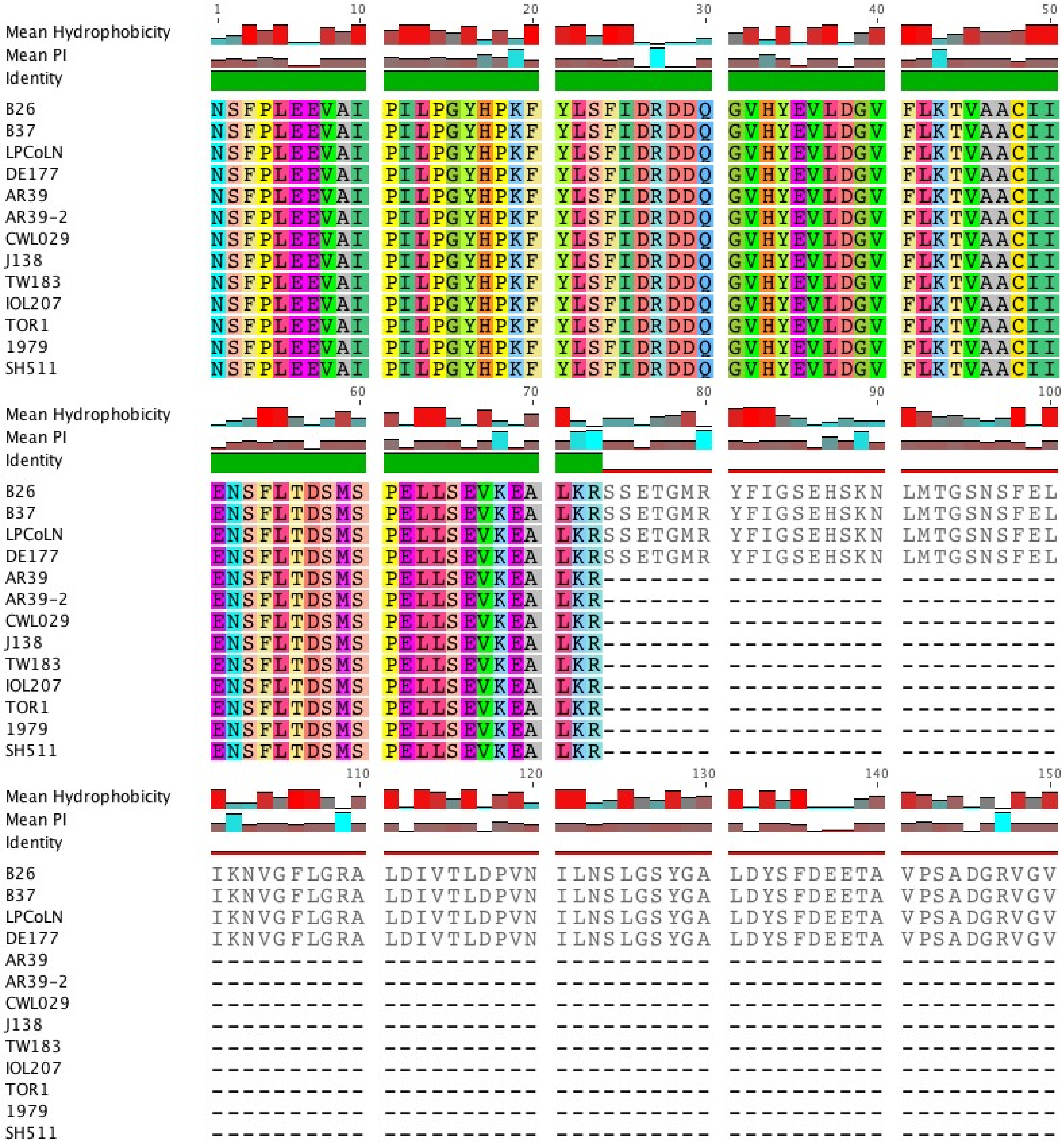

Mean Hydrophobicity
Mean PI
Identity
1 10 20 30 40 50 60 70 80 90 100 110 120 130 140 150
B26 NSFPLEEVAI PILPGYHPKF YLSFIDRDDQ GVHYEVLDGV FLKTVAACII ENSFLTDSMS PELLSEVKEA LKRSSETGMR YFIGSEHSKN LMTGSNSFEL IKNVGFLGRA LDIVTLDPVN ILNSLGSYGA LDYSFDEETA VPSADGRVGV
B37 NSFPLEEVAI PILPGYHPKF YLSFIDRDDQ GVHYEVLDGV FLKTVAACII ENSFLTDSMS PELLSEVKEA LKRSSETGMR YFIGSEHSKN LMTGSNSFEL IKNVGFLGRA LDIVTLDPVN ILNSLGSYGA LDYSFDEETA VPSADGRVGV
LPCoLN NSFPLEEVAI PILPGYHPKF YLSFIDRDDQ GVHYEVLDGV FLKTVAACII ENSFLTDSMS PELLSEVKEA LKRSSETGMR YFIGSEHSKN LMTGSNSFEL IKNVGFLGRA LDIVTLDPVN ILNSLGSYGA LDYSFDEETA VPSADGRVGV
DE177 NSFPLEEVAI PILPGYHPKF YLSFIDRDDQ GVHYEVLDGV FLKTVAACII ENSFLTDSMS PELLSEVKEA LKRSSETGMR YFIGSEHSKN LMTGSNSFEL IKNVGFLGRA LDIVTLDPVN ILNSLGSYGA LDYSFDEETA VPSADGRVGV
AR39 NSFPLEEVAI PILPGYHPKF YLSFIDRDDQ GVHYEVLDGV FLKTVAACII ENSFLTDSMS PELLSEVKEA LKR-------
AR39-2 NSFPLEEVAI PILPGYHPKF YLSFIDRDDQ GVHYEVLDGV FLKTVAACII ENSFLTDSMS PELLSEVKEA LKR-------
CWL029 NSFPLEEVAI PILPGYHPKF YLSFIDRDDQ GVHYEVLDGV FLKTVAACII ENSFLTDSMS PELLSEVKEA LKR-------
J138 NSFPLEEVAI PILPGYHPKF YLSFIDRDDQ GVHYEVLDGV FLKTVAACII ENSFLTDSMS PELLSEVKEA LKR-------
TW183 NSFPLEEVAI PILPGYHPKF YLSFIDRDDQ GVHYEVLDGV FLKTVAACII ENSFLTDSMS PELLSEVKEA LKR-------
IOL207 NSFPLEEVAI PILPGYHPKF YLSFIDRDDQ GVHYEVLDGV FLKTVAACII ENSFLTDSMS PELLSEVKEA LKR-------
TOR1 NSFPLEEVAI PILPGYHPKF YLSFIDRDDQ GVHYEVLDGV FLKTVAACII ENSFLTDSMS PELLSEVKEA LKR-------
1979 NSFPLEEVAI PILPGYHPKF YLSFIDRDDQ GVHYEVLDGV FLKTVAACII ENSFLTDSMS PELLSEVKEA LKR-------
SH511 NSFPLEEVAI PILPGYHPKF YLSFIDRDDQ GVHYEVLDGV FLKTVAACII ENSFLTDSMS PELLSEVKEA LKR-------

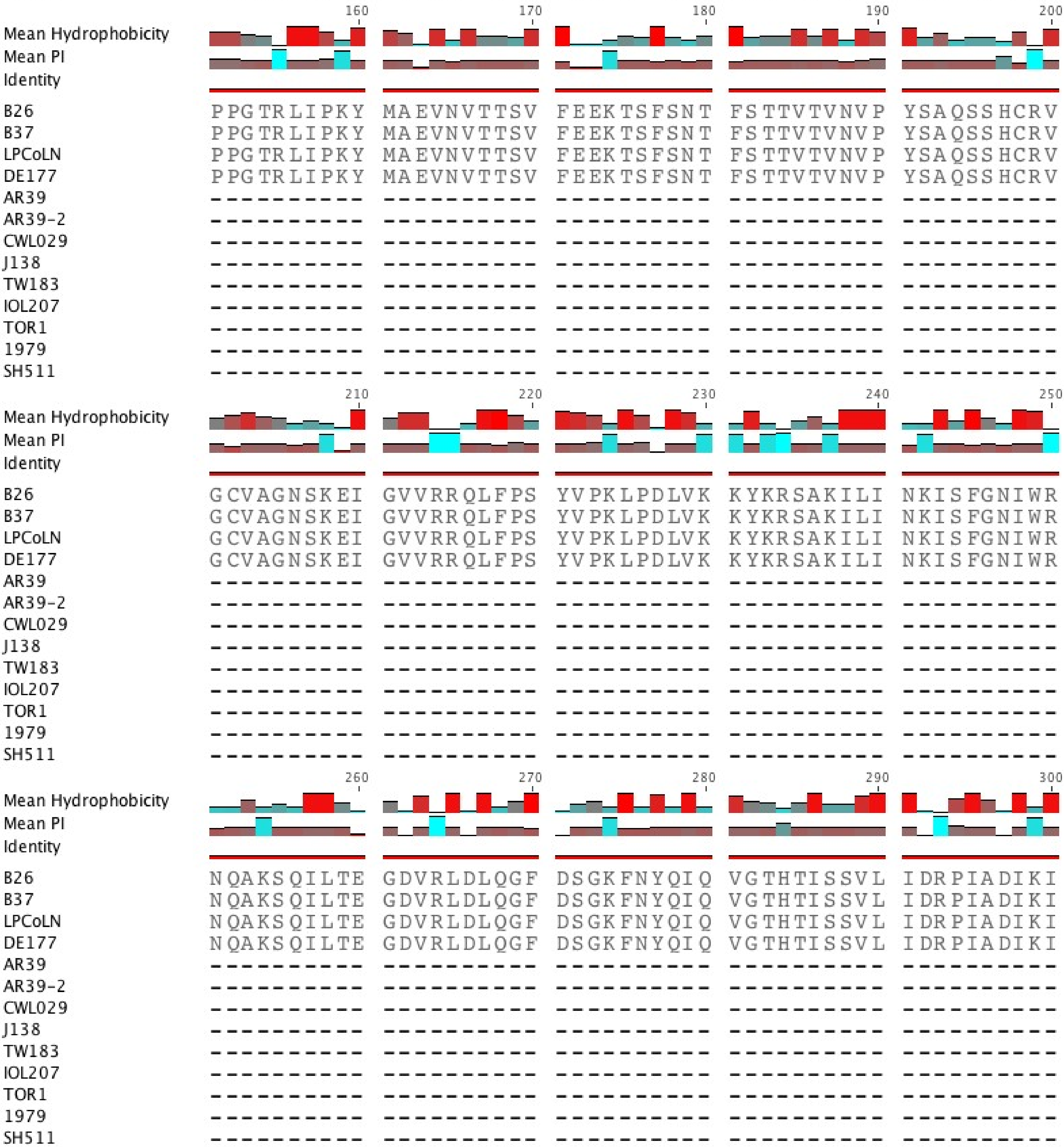
160
170
180
190
200
Mean Hydrophobicity
Mean PI
Identity
B26 PPGTRLIPKY MAEVNVTTSV FEEKTSFSNT FSTTVTVNVP YSAQSSHCRV
B37 PPGTRLIPKY MAEVNVTTSV FEEKTSFSNT FSTTVTVNVP YSAQSSHCRV
LPCoLN PPGTRLIPKY MAEVNVTTSV FEEKTSFSNT FSTTVTVNVP YSAQSSHCRV
DE177 PPGTRLIPKY MAEVNVTTSV FEEKTSFSNT FSTTVTVNVP YSAQSSHCRV
AR39
AR39-2
CWL029
J138
TW183
IOL207
TOR1
1979
SH511
210
220
230
240
250
Mean Hydrophobicity
Mean PI
Identity
B26 GCVAGNSKEI GVVRRQLFPS YVPKLPDLVK KYKRSAKILI NKISFGNIWR
B37 GCVAGNSKEI GVVRRQLFPS YVPKLPDLVK KYKRSAKILI NKISFGNIWR
LPCoLN GCVAGNSKEI GVVRRQLFPS YVPKLPDLVK KYKRSAKILI NKISFGNIWR
DE177 GCVAGNSKEI GVVRRQLFPS YVPKLPDLVK KYKRSAKILI NKISFGNIWR
AR39
AR39-2
CWL029
J138
TW183
IOL207
TOR1
1979
SH511
260
270
280
290
300
Mean Hydrophobicity
Mean PI
Identity
B26 NQAKSQILTE GDVRLDLQGF DSGKFNYQIQ VGTHTISSVL IDRPIADIKI
B37 NQAKSQILTE GDVRLDLQGF DSGKFNYQIQ VGTHTISSVL IDRPIADIKI
LPCoLN NQAKSQILTE GDVRLDLQGF DSGKFNYQIQ VGTHTISSVL IDRPIADIKI
DE177 NQAKSQILTE GDVRLDLQGF DSGKFNYQIQ VGTHTISSVL IDRPIADIKI
AR39
AR39-2
CWL029
J138
TW183
IOL207
TOR1
1979
SH511

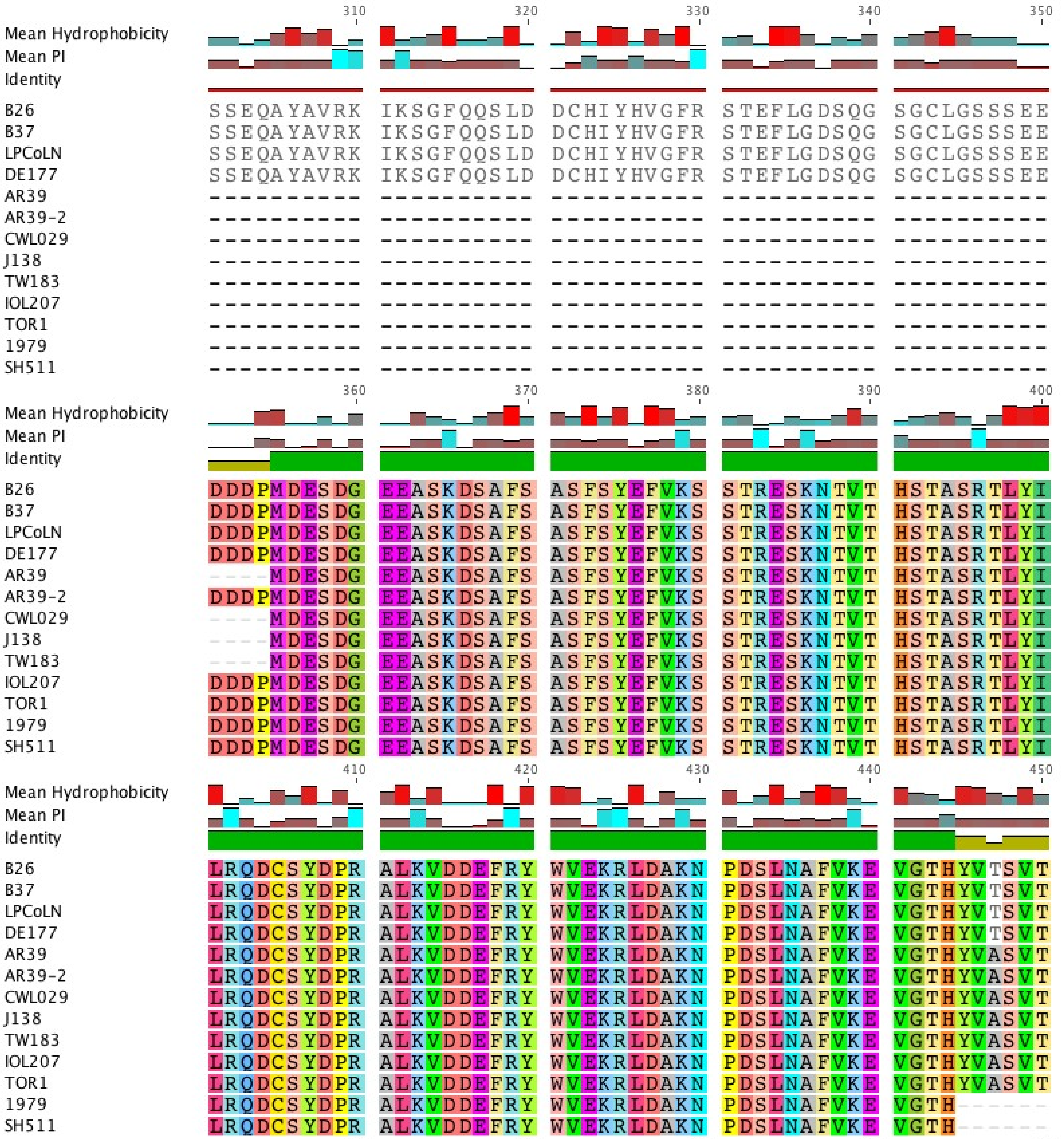
310 320 330 340 350
Mean Hydrophobicity
Mean PI
Identity
B26 SSEQAYAVRK IKSGFQQSLD DCHIYHVGFR STEFLGDSQG SGCLGSSSEE
B37 SSEQAYAVRK IKSGFQQSLD DCHIYHVGFR STEFLGDSQG SGCLGSSSEE
LPCoLN SSEQAYAVRK IKSGFQQSLD DCHIYHVGFR STEFLGDSQG SGCLGSSSEE
DE177 SSEQAYAVRK IKSGFQQSLD DCHIYHVGFR STEFLGDSQG SGCLGSSSEE
AR39 ---------- ---------- ---------- ---------- ----------
AR39-2 ---------- ---------- ---------- ---------- ----------
CWL029 ---------- ---------- ---------- ---------- ----------
J138 ---------- ---------- ---------- ---------- ----------
TW183 ---------- ---------- ---------- ---------- ----------
IOL207 ---------- ---------- ---------- ---------- ----------
TOR1 ---------- ---------- ---------- ---------- ----------
1979 ---------- ---------- ---------- ---------- ----------
SH511 ---------- ---------- ---------- ---------- ----------
360 370 380 390 400
Mean Hydrophobicity
Mean PI
Identity
B26 DDDPMDESDG EEASKDSAFS ASFSYEFVKS STRESKNTVT HSTASRTLYI
B37 DDDPMDESDG EEASKDSAFS ASFSYEFVKS STRESKNTVT HSTASRTLYI
LPCoLN DDDPMDESDG EEASKDSAFS ASFSYEFVKS STRESKNTVT HSTASRTLYI
DE177 DDDPMDESDG EEASKDSAFS ASFSYEFVKS STRESKNTVT HSTASRTLYI
AR39 ----MDESDG EEASKDSAFS ASFSYEFVKS STRESKNTVT HSTASRTLYI
AR39-2 DDDPMDESDG EEASKDSAFS ASFSYEFVKS STRESKNTVT HSTASRTLYI
CWL029 ----MDESDG EEASKDSAFS ASFSYEFVKS STRESKNTVT HSTASRTLYI
J138 ----MDESDG EEASKDSAFS ASFSYEFVKS STRESKNTVT HSTASRTLYI
TW183 ----MDESDG EEASKDSAFS ASFSYEFVKS STRESKNTVT HSTASRTLYI
IOL207 DDDPMDESDG EEASKDSAFS ASFSYEFVKS STRESKNTVT HSTASRTLYI
TOR1 DDDPMDESDG EEASKDSAFS ASFSYEFVKS STRESKNTVT HSTASRTLYI
1979 DDDPMDESDG EEASKDSAFS ASFSYEFVKS STRESKNTVT HSTASRTLYI
SH511 DDDPMDESDG EEASKDSAFS ASFSYEFVKS STRESKNTVT HSTASRTLYI
410 420 430 440 450
Mean Hydrophobicity
Mean PI
Identity
B26 LRQDCSYDPR ALKVDDEFRY WVEKRLDAKN PDSLNAFVKE VGTHYVTSVT
B37 LRQDCSYDPR ALKVDDEFRY WVEKRLDAKN PDSLNAFVKE VGTHYVTSVT
LPCoLN LRQDCSYDPR ALKVDDEFRY WVEKRLDAKN PDSLNAFVKE VGTHYVTSVT
DE177 LRQDCSYDPR ALKVDDEFRY WVEKRLDAKN PDSLNAFVKE VGTHYVTSVT
AR39 LRQDCSYDPR ALKVDDEFRY WVEKRLDAKN PDSLNAFVKE VGTHYVASVT
AR39-2 LRQDCSYDPR ALKVDDEFRY WVEKRLDAKN PDSLNAFVKE VGTHYVASVT
CWL029 LRQDCSYDPR ALKVDDEFRY WVEKRLDAKN PDSLNAFVKE VGTHYVASVT
J138 LRQDCSYDPR ALKVDDEFRY WVEKRLDAKN PDSLNAFVKE VGTHYVASVT
TW183 LRQDCSYDPR ALKVDDEFRY WVEKRLDAKN PDSLNAFVKE VGTHYVASVT
IOL207 LRQDCSYDPR ALKVDDEFRY WVEKRLDAKN PDSLNAFVKE VGTHYVASVT
TOR1 LRQDCSYDPR ALKVDDEFRY WVEKRLDAKN PDSLNAFVKE VGTHYVASVT
1979 LRQDCSYDPR ALKVDDEFRY WVEKRLDAKN PDSLNAFVKE VGTH------
SH511 LRQDCSYDPR ALKVDDEFRY WVEKRLDAKN PDSLNAFVKE VGTH------

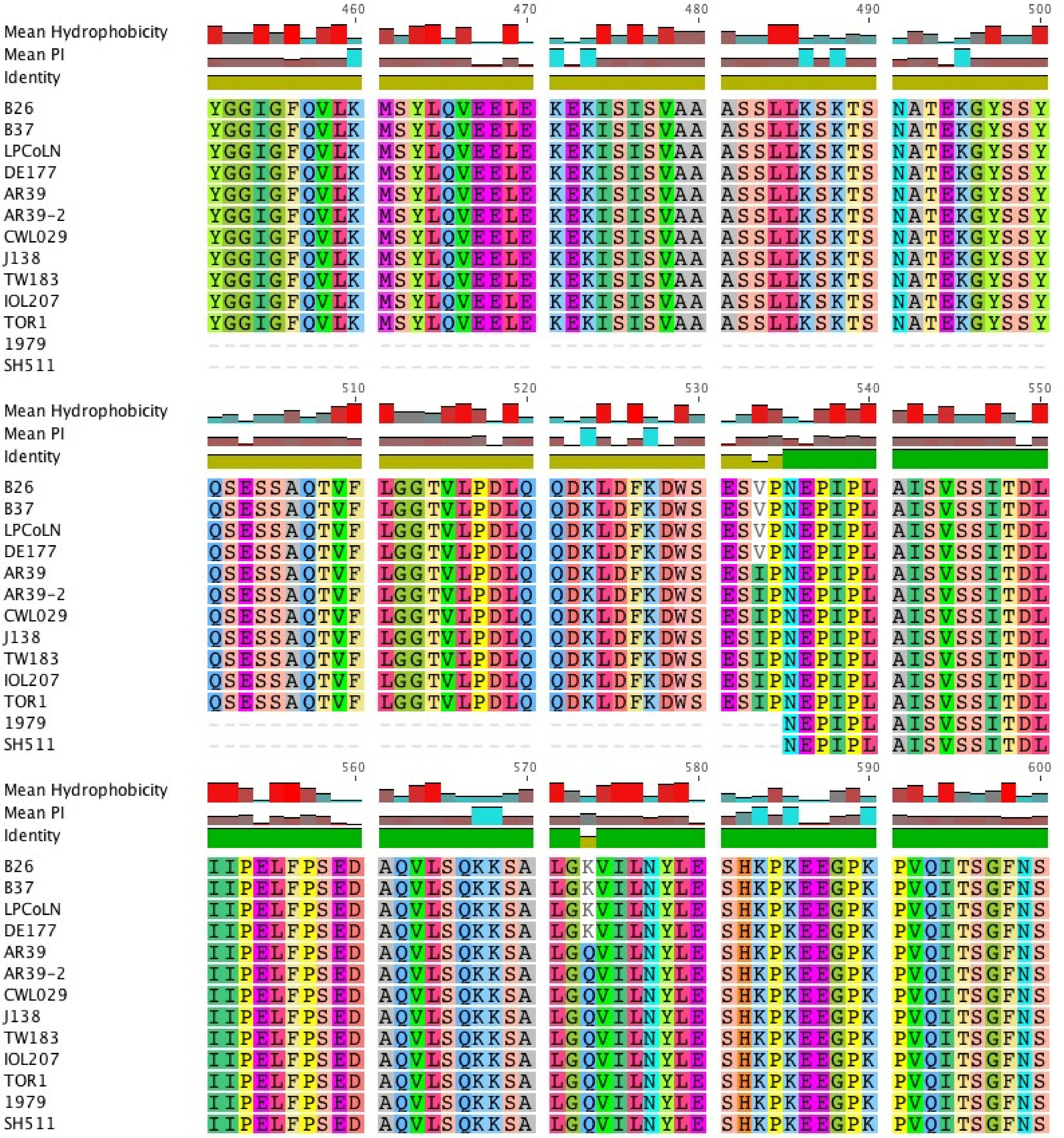
460 470 480 490 500
Mean Hydrophobicity
Mean PI
Identity
B26 YGGIGFQVLK MSYLQVEELE KEKISISVAA ASSLLKSKTS NATEKGYSSY
B37 YGGIGFQVLK MSYLQVEELE KEKISISVAA ASSLLKSKTS NATEKGYSSY
LPCoLN YGGIGFQVLK MSYLQVEELE KEKISISVAA ASSLLKSKTS NATEKGYSSY
DE177 YGGIGFQVLK MSYLQVEELE KEKISISVAA ASSLLKSKTS NATEKGYSSY
AR39 YGGIGFQVLK MSYLQVEELE KEKISISVAA ASSLLKSKTS NATEKGYSSY
AR39-2 YGGIGFQVLK MSYLQVEELE KEKISISVAA ASSLLKSKTS NATEKGYSSY
CWL029 YGGIGFQVLK MSYLQVEELE KEKISISVAA ASSLLKSKTS NATEKGYSSY
J138 YGGIGFQVLK MSYLQVEELE KEKISISVAA ASSLLKSKTS NATEKGYSSY
TW183 YGGIGFQVLK MSYLQVEELE KEKISISVAA ASSLLKSKTS NATEKGYSSY
IOL207 YGGIGFQVLK MSYLQVEELE KEKISISVAA ASSLLKSKTS NATEKGYSSY
TOR1 YGGIGFQVLK MSYLQVEELE KEKISISVAA ASSLLKSKTS NATEKGYSSY
1979
SH511
510 520 530 540 550
Mean Hydrophobicity
Mean PI
Identity
B26 QSESSAQTVF LGGTVLPDLQ QDKLDFKDWS ESVPNEPIPL AISVSSITDL
B37 QSESSAQTVF LGGTVLPDLQ QDKLDFKDWS ESVPNEPIPL AISVSSITDL
LPCoLN QSESSAQTVF LGGTVLPDLQ QDKLDFKDWS ESVPNEPIPL AISVSSITDL
DE177 QSESSAQTVF LGGTVLPDLQ QDKLDFKDWS ESVPNEPIPL AISVSSITDL
AR39 QSESSAQTVF LGGTVLPDLQ QDKLDFKDWS ESIPNEPIPL AISVSSITDL
AR39-2 QSESSAQTVF LGGTVLPDLQ QDKLDFKDWS ESIPNEPIPL AISVSSITDL
CWL029 QSESSAQTVF LGGTVLPDLQ QDKLDFKDWS ESIPNEPIPL AISVSSITDL
J138 QSESSAQTVF LGGTVLPDLQ QDKLDFKDWS ESIPNEPIPL AISVSSITDL
TW183 QSESSAQTVF LGGTVLPDLQ QDKLDFKDWS ESIPNEPIPL AISVSSITDL
IOL207 QSESSAQTVF LGGTVLPDLQ QDKLDFKDWS ESIPNEPIPL AISVSSITDL
TOR1 QSESSAQTVF LGGTVLPDLQ QDKLDFKDWS ESIPNEPIPL AISVSSITDL
1979 NEPIPL AISVSSITDL
SH511 NEPIPL AISVSSITDL
560 570 580 590 600
Mean Hydrophobicity
Mean PI
Identity
B26 IIPELFPSED AQVLSQKKSA LGKVILNYLE SHKPKEEGPK PVQITSGFNS
B37 IIPELFPSED AQVLSQKKSA LGKVILNYLE SHKPKEEGPK PVQITSGFNS
LPCoLN IIPELFPSED AQVLSQKKSA LGKVILNYLE SHKPKEEGPK PVQITSGFNS
DE177 IIPELFPSED AQVLSQKKSA LGKVILNYLE SHKPKEEGPK PVQITSGFNS
AR39 IIPELFPSED AQVLSQKKSA LGQVILNYLE SHKPKEEGPK PVQITSGFNS
AR39-2 IIPELFPSED AQVLSQKKSA LGQVILNYLE SHKPKEEGPK PVQITSGFNS
CWL029 IIPELFPSED AQVLSQKKSA LGQVILNYLE SHKPKEEGPK PVQITSGFNS
J138 IIPELFPSED AQVLSQKKSA LGQVILNYLE SHKPKEEGPK PVQITSGFNS
TW183 IIPELFPSED AQVLSQKKSA LGQVILNYLE SHKPKEEGPK PVQITSGFNS
IOL207 IIPELFPSED AQVLSQKKSA LGQVILNYLE SHKPKEEGPK PVQITSGFNS
TOR1 IIPELFPSED AQVLSQKKSA LGQVILNYLE SHKPKEEGPK PVQITSGFNS
1979 IIPELFPSED AQVLSQKKSA LGQVILNYLE SHKPKEEGPK PVQITSGFNS
SH511 IIPELFPSED AQVLSQKKSA LGQVILNYLE SHKPKEEGPK PVQITSGFNS

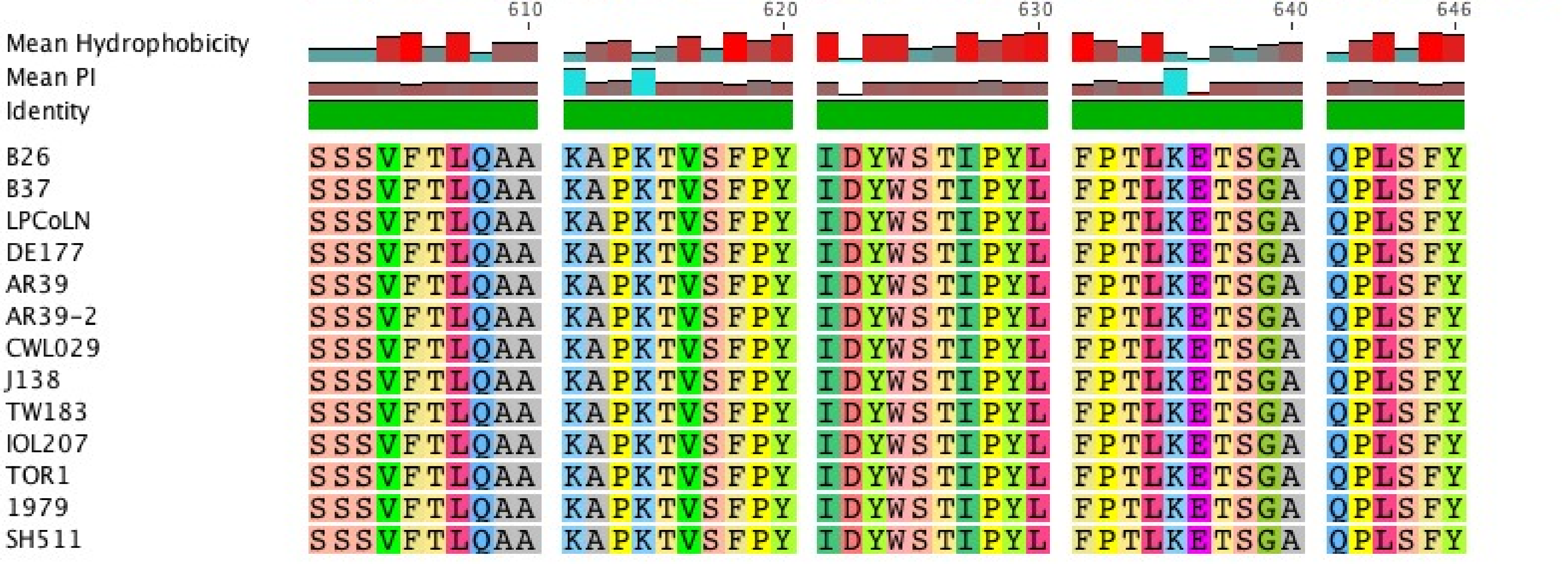
610
620
630
640
646
Mean Hydrophobicity
Mean PI
Identity
B26 SSSVFTLQAA KAPKTVSFPY IDYWSTIPYL FPTLKETSGA QPLSFY
B37 SSSVFTLQAA KAPKTVSFPY IDYWSTIPYL FPTLKETSGA QPLSFY
LPCoLN SSSVFTLQAA KAPKTVSFPY IDYWSTIPYL FPTLKETSGA QPLSFY
DE177 SSSVFTLQAA KAPKTVSFPY IDYWSTIPYL FPTLKETSGA QPLSFY
AR39 SSSVFTLQAA KAPKTVSFPY IDYWSTIPYL FPTLKETSGA QPLSFY
AR39-2 SSSVFTLQAA KAPKTVSFPY IDYWSTIPYL FPTLKETSGA QPLSFY
CWL029 SSSVFTLQAA KAPKTVSFPY IDYWSTIPYL FPTLKETSGA QPLSFY
J138 SSSVFTLQAA KAPKTVSFPY IDYWSTIPYL FPTLKETSGA QPLSFY
TW183 SSSVFTLQAA KAPKTVSFPY IDYWSTIPYL FPTLKETSGA QPLSFY
IOL207 SSSVFTLQAA KAPKTVSFPY IDYWSTIPYL FPTLKETSGA QPLSFY
TOR1 SSSVFTLQAA KAPKTVSFPY IDYWSTIPYL FPTLKETSGA QPLSFY
1979 SSSVFTLQAA KAPKTVSFPY IDYWSTIPYL FPTLKETSGA QPLSFY
SH511 SSSVFTLQAA KAPKTVSFPY IDYWSTIPYL FPTLKETSGA QPLSFY
