# Supplementary material for: Chlamydia pneumoniae Is Genetically Diverse in Animals and Appears to Have Crossed the Host Barrier to Humans on (At Least) Two Occasions
Source: PLoS Pathog. 2010 May 20;6(5):e1000903. doi: 10.1371/journal.ppat.1000903 (PMC2873915; doi:10.1371/journal.ppat.1000903)
Supplement: Figure S4 — Multiple sequence alignment of AroAA-Hs. Sequence from bandicoot (B26), koala (LPCoLN) and six human isolates (AR39, TW183, CWL029, J138, SH511 and 1979) identified a 244 bp length polymorphism at the 5′ end. (6.05 MB PDF) [file ppat.1000903.s004.pdf]

	1–10	20	30	40	50	60	70	80
Identity								
B26	TATTCTGAAG	ATTGCTCTAA	AGCTGAGACA	ATCACTTTCC	CTGTTCTTCC	AGAACAGCCA	ATCACTCCAA	CGTGCATACT
LPCoLN								
AR39	TATTCTGAAG	ATTGCTCTAA	AGCTGAGACA	ATCACTTTCC	CTGTTCTTCC	AGAACAGCCA	ATCACTCCAA	CGTGCATACT
CWL029	TATTCTGAAG	ATTGCTCTAA	AGCTGAGACA	ATCACTTTCC	CTGTTCTTCC	AGAACAGCCA	ATCACTCCAA	CGTGCATACT
J138	TATTCTGAAG	ATTGCTCTAA	AGCTGAGACA	ATCACTTTCC	CTGTTCTTCC	AGAACAGCCA	ATCACTCCAA	CGTGCATACT
TW183	TATTCTGAAG	ATTGCTCTAA	AGCTGAGACA	ATCACTTTCC	CTGTTCTTCC	AGAACAGCCA	ATCACTCCAA	CGTGCATACT
TOR1	TATTCTGAAG	ATTGCTCTAA	AGCTGAGACA	ATCACTTTCC	CTGTTCTTCC	AGAACAGCCA	ATCACTCCAA	CGTGCATACT
WA97001	TATTCTGAAG	ATTGCTCTAA	AGCTGAGACA	ATCACTTTCC	CTGTTCTTCC	AGAACAGCCA	ATCACTCCAA	CGTGCATACT
1979	TATTCTGAAG	ATTGCTCTAA	AGCTGAGACA	ATCACTTTCC	CTGTTCTTCC	AGAACAGCCA	ATCACTCCAA	CGTGCATACT
SH511	TATTCTGAAG	ATTGCTCTAA	AGCTGAGACA	ATCACTTTCC	CTGTTCTTCC	AGAACAGCCA	ATCACTCCAA	CGTGCATACT

	90	100	110	120	130	140	150	160
Identity								
B26	CGACCCCATA	TTCCTACTAC	CGAATCATTC	TACAAAAGGA	AAATAAAGAG	AAGCAAGCTT	TAGCTCGACA	CAAATGCATT
LPCoLN								
AR39	CGACCCCATA	TTCCTACTAC	CGAATCATTC	TACAAAAGGA	AAATAAAGAG	AAGCAAGCTT	TAGCTCGACA	CAAATGCATT
CWL029	CGACCCCATA	TTCCTACTAC	CGAATCATTC	TACAAAAGGA	AAATAAAGAG	AAGCAAGCTT	TAGCTCGACA	CAAATGCATT
J138	CGACCCCATA	TTCCTACTAC	CGAATCATTC	TACAAAAGGA	AAATAAAGAG	AAGCAAGCTT	TAGCTCGACA	CAAATGCATT
TW183	CGACCCCATA	TTCCTACTAC	CGAATCATTC	TACAAAAGGA	AAATAAAGAG	AAGCAAGCTT	TAGCTCGACA	CAAATGCATT
TOR1	CGACCCCATA	TTCCTACTAC	CGAATCATTC	TACAAAAGGA	AAATAAAGAG	AAGCAAGCTT	TAGCTCGACA	CAAATGCATT
WA97001	CGACCCCATA	TTCCTACTAC	CGAATCATTC	TACAAAAGGA	AAATAAAGAG	AAGCAAGCTT	TAGCTCGACA	CAAATGCATT
1979	CGACCCCATA	TTCCTACTAC	CGAATCATTC	TACAAAAGGA	AAATAAAGAG	AAGCAAGCTT	TAGCTCGACA	CAAATGCATT
SH511	CGACCCCATA	TTCCTACTAC	CGAATCATTC	TACAAAAGGA	AAATAAAGAG	AAGCAAGCTT	TAGCTCGACA	CAAATGCATT

	170	180	190	200	210	220	230	240
Identity								
B26	TCTATTTTAG	AATTTTTCAA	AAACTTACTC	TTTGTTCATC	TTCTGTCATT	ATCAAAGAAT	CAAAGGGAAG	GTTGCTCCAC
LPCoLN								
AR39	TCTATTTTAG	AATTTTTCAA	AAACTTACTC	TTTGTTCATC	TTCTGTCATT	ATCAAAGAAT	CAAAGGGAAG	GTTGCTCCAC
CWL029	TCTATTTTAG	AATTTTTCAA	AAACTTACTC	TTTGTTCATC	TTCTGTCATT	ATCAAAGAAT	CAAAGGGAAG	GTTGCTCCAC
J138	TCTATTTTAG	AATTTTTCAA	AAACTTACTC	TTTGTTCATC	TTCTGTCATT	ATCAAAGAAT	CAAAGGGAAG	GTTGCTCCAC
TW183	TCTATTTTAG	AATTTTTCAA	AAACTTACTC	TTTGTTCATC	TTCTGTCATT	ATCAAAGAAT	CAAAGGGAAG	GTTGCTCCAC
TOR1	TCTATTTTAG	AATTTTTCAA	AAACTTACTC	TTTGTTCATC	TTCTGTCATT	ATCAAAGAAT	CAAAGGGAAG	GTTGCTCCAC
WA97001	TCTATTTTAG	AATTTTTCAA	AAACTTACTC	TTTGTTCATC	TTCTGTCATT	ATCAAAGAAT	CAAAGGGAAG	GTTGCTCCAC
1979	TCTATTTTAG	AATTTTTCAA	AAACTTACTC	TTTGTTCATC	TTCTGTCATT	ATCAAAGAAT	CAAAGGGAAG	GTTGCTCCAC
SH511	TCTATTTTAG	AATTTTTCAA	AAACTTACTC	TTTGTTCATC	TTCTGTCATT	ATCAAAGAAT	CAAAGGGAAG	GTTGCTCCAC

	250	260	270	280	290	300	310	320
Identity								
B26	TGATATGGCT	GTTGTAAGCA	CTCCGTTTTT	TAATCAGAAT	TTATGGTATC	GACTCCTTTC	CTCACGGTTT	TCTCTATGGA
LPCoLN	ATGGCT	GTTGTAAGCA	CTCCGTTTTT	TAATCAGAAT	TTATGGTATC	GACTCCTTTC	CTCACGGTTT	TCTCTATGGA
AR39	TGATATGGCT	GTTGTAAGCA	CTCCCTTTTT	TAATCGGAAT	TTATGGTATC	GACTCCTTTC	CTCACGGTTT	TCTCTATGGA
CWL029	TGATATGGCT	GTTGTAAGCA	CTCCCTTTTT	TAATCGGAAT	TTATGGTATC	GACTCCTTTC	CTCACGGTTT	TCTCTATGGA
J138	TGATATGGCT	GTTGTAAGCA	CTCCCTTTTT	TAATCGGAAT	TTATGGTATC	GACTCCTTTC	CTCACGGTTT	TCTCTATGGA
TW183	TGATATGGCT	GTTGTAAGCA	CTCCCTTTTT	TAATCGGAAT	TTATGGTATC	GACTCCTTTC	CTCACGGTTT	TCTCTATGGA
TOR1	TGATATGGCT	GTTGTAAGCA	CTCCCTTTTT	TAATCGGAAT	TTATGGTATC	GACTCCTTTC	CTCACGGTTT	TCTCTATGGA
WA97001	TGATATGGCT	GTTGTAAGCA	CTCCCTTTTT	TAATCGGAAT	TTATGGTATC	GACTCCTTTC	CTCACGGTTT	TCTCTATGGA
1979	TGATATGGCT	GTTGTAAGCA	CTCCCTTTTT	TAATCGGAAT	TTATGGTATC	GACTCCTTTC	CTCACGGTTT	TCTCTATGGA
SH511	TGATATGGCT	GTTGTAAGCA	CTCCCTTTTT	TAATCGGAAT	TTATGGTATC	GACTCCTTTC	CTCACGGTTT	TCTCTATGGA

	330	340	350	360	370	380	390	400
Identity								
B26	AAAGCTATTG	TCCAAGATTT	TTTCTTGATT	ACTTAGAAGC	TTTCGGTCTC	CTTTCTGATT	TCTTAGACCA	TCAAGCAGTC
LPCoLN	AAAGCTATTG	TCCAAGATTT	TTTCTTGATT	ACTTAGAAGC	TTTCGGTCTC	CTTTCTGATT	TCTTAGACCA	TCAAGCAGTC
AR39	AAAGCTATTG	TCCAAGATTT	TTTCTTGATT	ACTTAGAAGC	TTTCGGTCTC	CTTTCTGATT	TCTTAGACCA	TCAAGCAGTC
CWL029	AAAGCTATTG	TCCAAGATTT	TTTCTTGATT	ACTTAGAAGC	TTTCGGTCTC	CTTTCTGATT	TCTTAGACCA	TCAAGCAGTC
J138	AAAGCTATTG	TCCAAGATTT	TTTCTTGATT	ACTTAGAAGC	TTTCGGTCTC	CTTTCTGATT	TCTTAGACCA	TCAAGCAGTC
TW183	AAAGCTATTG	TCCAAGATTT	TTTCTTGATT	ACTTAGACGC	TTTCGGTCTC	CTTTCTGATT	TCTTAGACCA	TCAAGCAGTC
TOR1	AAAGCTATTG	TCCAAGATTT	TTTCTTGATT	ACTTAGAAGC	TTTCGGTCTC	CTTTCTGATT	TCTTAGACCA	TCAAGCAGTC
WA97001	AAAGCTATTG	TCCAAGATTT	TTTCTTGATT	ACTTAGAAGC	TTTCGGTCTC	CTTTCTGATT	TCTTAGACCA	TCAAGCAGTC
1979	AAAGCTATTG	TCCAAGATTT	TTTCTTGATT	ACTTAGAAGC	TTTCGGTCTC	CTTTCTGATT	TCTTAGACCA	TCAAGCAGTC
SH511	AAAGCTATTG	TCCAAGATTT	TTTCTTGATT	ACTTAGAAGC	TTTCGGTCTC	CTTTCTGATT	TCTTAGACCA	TCAAGCAGTC

	410	420	430	440	450	460	470	480
Identity								
B26	ATTAAATTCT	TCGAATTAGA	AACACATTTT	TCCTATTATC	CCGTTTCAGG	ATTTGTAGCT	CCCCATCAAT	ACTTGTCTCT
LPCoLN	ATTAAATTCT	TCGAATTAGA	AACACATTTT	TCCTATTATC	CCGTTTCAGG	ATTTGTAGCT	CCCCATCAAT	ACTTGTCTCT
AR39	ATTAAATTCT	TCGAATTAGA	AACACATTTT	TCCTATTATC	CCGTTTCAGG	ATTTGTAGCT	CCCCATCAAT	ACTTGTCTCT
CWL029	ATTAAATTCT	TCGAATTAGA	AACACATTTT	TCCTATTATC	CCGTTTCAGG	ATTTGTAGCT	CCCCATCAAT	ACTTGTCTCT
J138	ATTAAATTCT	TCGAATTAGA	AACACATTTT	TCCTATTATC	CCGTTTCAGG	ATTTGTAGCT	CCCCATCAAT	ACTTGTCTCT
TW183	ATTAAATTCT	TCGAATTAGA	AACACATTTT	TCCTATTATC	CCGTTTCAGG	ATTTGTAGCT	CCCCATCAAT	ACTTGTCTCT
TOR1	ATTAAATTCT	TCGAATTAGA	AACACATTTT	TCCTATTATC	CCGTTTCAGG	ATTTGTAGCT	CCCCATCAAT	ACTTGTCTCT
WA97001	ATTAAATTCT	TCGAATTAGA	AACACATTTT	TCCTATTATC	CCGTTTCAGG	ATTTGTAGCT	CCCCATCAAT	ACTTGTCTCT
1979	ATTAAATTCT	TCGAATTAGA	AACACATTTT	TCCTATTATC	CCGTTTCAGG	ATTTGTAGCT	CCCCATCAAT	ACTTGTCTCT
SH511	ATTAAATTCT	TCGAATTAGA	AACACATTTT	TCCTATTATC	CCGTTTCAGG	ATTTGTAGCT	CCCCATCAAT	ACTTGTCTCT

	490	500	510	520	530	540	550	560
Identity								
B26	GTTGCAGGAC	CGTTACTTTC	CCATTGCCTC	TGTAATGCGA	ACTCTCGATA	AAGATAATTT	CTCCTTAACT	CCTGATCTCA
LPCoLN	GTTGCAGGAC	CGTTACTTTC	CCATTGCCTC	TGTAATGCGA	ACTCTCGATA	AAGATAATTT	CTCCTTAACT	CCTGATCTCA
AR39	GTTGCAGGAC	CGTTACTTTC	CCATTGCCTC	TGTAATGCGA	ACTCTCGATA	AAGATAATTT	CTCCTTAACT	CCTGATCTCA
CWL029	GTTGCAGGAC	CGTTACTTTC	CCATTGCCTC	TGTAATGCGA	ACTCTCGATA	AAGATAATTT	CTCCTTAACT	CCTGATCTCA
J138	GTTGCAGGAC	CGTTACTTTC	CCATTGCCTC	TGTAATGCGA	ACTCTCGATA	AAGATAATTT	CTCCTTAACT	CCTGATCTCA
TW183	GTTGCAGGAC	CGTTACTTTC	CCATTGCCTC	TGTAATGCGA	ACTCTCGATA	AAGATAATTT	CTCCTTAACT	CCTGATCTCA
TOR1	GTTGCAGGAC	CGTTACTTTC	CCATTGCCTC	TGTAATGCGA	ACTCTCGATA	AAGATAATTT	CTCCTTAACT	CCTGATCTCA
WA97001	GTTGCAGGAC	CGTTACTTTC	CCATTGCCTC	TGTAATGCGA	ACTCTCGATA	AAGATAATTT	CTCCTTAACT	CCTGATCTCA
1979	GTTGCAGGAC	CGTTACTTTC	CCATTGCCTC	TGTAATGCGA	ACTCTCGATA	AAGATAATTT	CTCCTTAACT	CCTGATCTCA
SH511	GTTGCAGGAC	CGTTACTTTC	CCATTGCCTC	TGTAATGCGA	ACTCTCGATA	AAGATAATTT	CTCCTTAACT	CCTGATCTCA

	570	580	590	600	610	620	630	640
Identity								
B26	TCCATGACCT	TTTAGGGCAC	GTGCCTTGGC	TTCTACATCC	CTCATTTTCT	GAATTTTTCA	TAAACATGGG	AAGACTCTTC
LPCoLN	TCCATGACCT	TTTAGGGCAC	GTGCCTTGGC	TTCTACATCC	CTCATTTTCT	GAATTTTTCA	TAAACATGGG	AAGACTCTTC
AR39	TCCATGACCT	TTTAGGGCAC	GTGCCTTGGC	TTCTACATCC	CTCATTTTCT	GAATTTTTCA	TAAACATGGG	AAGACTCTTC
CWL029	TCCATGACCT	TTTAGGGCAC	GTGCCTTGGC	TTCTACATCC	CTCATTTTCT	GAATTTTTCA	TAAACATGGG	AAGACTCTTC
J138	TCCATGACCT	TTTAGGGCAC	GTGCCTTGGC	TTCTACATCC	CTCATTTTCT	GAATTTTTCA	TAAACATGGG	AAGACTCTTC
TW183	TCCATGACCT	TTTAGGGCAC	GTGCCTTGGC	TTCTACATCC	CTCATTTTCT	GAATTTTTCA	TAAACATGGG	AAGACTCTTC
TOR1	TCCATGACCT	TTTAGGGCAC	GTGCCTTGGC	TTCTACATCC	CTCATTTTCT	GAATTTTTCA	TAAACATGGG	AAGACTCTTC
WA97001	TCCATGACCT	TTTAGGGCAC	GTGCCTTGGC	TTCTACATCC	CTCATTTTCT	GAATTTTTCA	TAAACATGGG	AAGACTCTTC
1979	TCCATGACCT	TTTAGGGCAC	GTGCCTTGGC	TTCTACATCC	CTCATTTTCT	GAATTTTTCA	TAAACATGGG	AAGACTCTTC
SH511	TCCATGACCT	TTTAGGGCAC	GTGCCTTGGC	TTCTACATCC	CTCATTTTCT	GAATTTTTCA	TAAACATGGG	AAGACTCTTC

	650	660	670	680	690	700	710	720
B26	ACTAAAGTCA	TAGAAAAAGT	ACAAGCTCTT	CCTAGTAAAA	AACAACGCAT	ACAAACCCTA	CAAAGCAATC	TGATCGCTAT
LPCoLN	ACTAAAGTCA	TAGAAAAAGT	ACAAGCTCTT	CCTAGTAAAA	AACAACGCAT	ACAAACCCTA	CAAAGCAATC	TGATCGCTAT
AR39	ACTAAAGTCA	TAGAAAAAGT	ACAAGCTCTT	CCTAGTAAAA	AACAACGCAT	ACAAACCCTA	CAAAGCAATC	TGATCGCTAT
CWL029	ACTAAAGTCA	TAGAAAAAGT	ACAAGCTCTT	CCTAGTAAAA	AACAACGCAT	ACAAACCCTA	CAAAGCAATC	TGATCGCTAT
J138	ACTAAAGTCA	TAGAAAAAGT	ACAAGCTCTT	CCTAGTAAAA	AACAACGCAT	ACAAACCCTA	CAAAGCAATC	TGATCGCTAT
TW183	ACTAAAGTCA	TAGAAAAAGT	ACAAGCTCTT	CCTAGTAAAA	AACAACGCAT	ACAAACCCTA	CAAAGCAATC	TGATCGCTAT
TOR1	ACTAAAGTCA	TAGAAAAAGT	ACAAGCTCTT	CCTAGTAAAA	AACAACGCAT	ACAAACCCTA	CAAAGCAATC	TGATCGCTAT
WA97001	ACTAAAGTCA	TAGAAAAAGT	ACAAGCTCTT	CCTAGTAAAA	AACAACGCAT	ACAAACCCTA	CAAAGCAATC	TGATCGCTAT
1979	ACTAAAGTCA	TAGAAAAAGT	ACAAGCTCTT	CCTAGTAAAA	AACAACGCAT	ACAAACCCTA	CAAAGCAATC	TGATCGCTAT
SH511	ACTAAAGTCA	TAGAAAAAGT	ACAAGCTCTT	CCTAGTAAAA	AACAACGCAT	ACAAACCCTA	CAAAGCAATC	TGATCGCTAT

	730	740	750	760	770	780	790	800
B26	TATACGCTGC	TTTTGGTTTA	CTGTTGAAAG	CGGACTTATT	GAAAACCATG	AAGGAAGAAA	AGCATATGGA	GCCGTTCTTA
LPCoLN	TATACGCTGC	TTTTGGTTTA	CTGTTGAAAG	CGGACTTATT	GAAAACCATG	AAGGAAGAAA	AGCATATGGA	GCCGTTCTTA
AR39	TGTACGCTGC	TTTTGGTTTA	CTGTTGAAAG	CGGACTTATT	GAAAACCATG	AAGGAAGAAA	AGCATATGGA	GCCGTTCTTA
CWL029	TGTACGCTGC	TTTTGGTTTA	CTGTTGAAAG	CGGACTTATT	GAAAACCATG	AAGGAAGAAA	AGCATATGGA	GCCGTTCTTA
J138	TGTACGCTGC	TTTTGGTTTA	CTGTTGAAAG	CGGACTTATT	GAAAACCATG	AAGGAAGAAA	AGCATATGGA	GCCGTTCTTA
TW183	TGTACGCTGC	TTTTGGTTTA	CTGTTGAAAG	CGGACTTATT	GAAAACCATG	AAGGAAGAAA	AGCATATGGA	GCCGTTCTTA
TOR1	TGTACGCTGC	TTTTGGTTTA	CTGTTGAAAG	CGGACTTATT	GAAAACCATG	AAGGAAGAAA	AGCATATGGA	GCCGTTCTTA
WA97001	TGTACGCTGC	TTTTGGTTTA	CTGTTGAAAG	CGGACTTATT	GAAAACCATG	AAGGAAGAAA	AGCATATGGA	GCCGTTCTTA
1979	TGTACGCTGC	TTTTGGTTTA	CTGTTGAAAG	CGGACTTATT	GAAAACCATG	AAGGAAGAAA	AGCATATGGA	GCCGTTCTTA
SH511	TGTACGCTGC	TTTTGGTTTA	CTGTTGAAAG	CGGACTTATT	GAAAACCATG	AAGGAAGAAA	AGCATATGGA	GCCGTTCTTA

	810	820	830	840	850	860	870	880
B26	TCAGTTCTCC	TCAGGAACTT	AGACACGCTT	TCATTGATAA	CGTACGTGTT	CTCCCTTTAG	AATTGGATCA	GATTATTCGT
LPCoLN	TCAGTTCTCC	TCAGGAACTT	AGACACGCTT	TCATTGATAA	CGTACGTGTT	CTCCCTTTAG	AATTGGATCA	GATTATTCGT
AR39	TCAGTTCTCC	TCAGGAACTT	GGACACGCTT	TCATTGATAA	CGTACGTGTT	CTCCCTTTAG	AATTGGATCA	GATTATTCGT
CWL029	TCAGTTCTCC	TCAGGAACTT	GGACACGCTT	TCATTGATAA	CGTACGTGTT	CTCCCTTTAG	AATTGGATCA	GATTATTCGT
J138	TCAGTTCTCC	TCAGGAACTT	GGACACGCTT	TCATTGATAA	CGTACGTGTT	CTCCCTTTAG	AATTGGATCA	GATTATTCGT
TW183	TCAGTTCTCC	TCAGGAACTT	GGACACGCTT	TCATTGATAA	CGTACGTGTT	CTCCCTTTAG	AATTGGATCA	GATTATTCGT
TOR1	TCAGTTCTCC	TCAGGAACTT	GGACACGCTT	TCATTGATAA	CGTACGTGTT	CTCCCTTTAG	AATTGGATCA	GATTATTCGT
WA97001	TCAGTTCTCC	TCAGGAACTT	GGACACGCTT	TCATTGATAA	CGTACGTGTT	CTCCCTTTAG	AATTGGATCA	GATTATTCGT
1979	TCAGTTCTCC	TCAGGAACTT	AGACACGCTT	TCATTGATAA	CGTACGTGTT	CTCCCTTTAG	AATTGGATCA	GATTATTCGT
SH511	TCAGTTCTCC	TCAGGAACTT	AGACACGCTT	TCATTGATAA	CGTACGTGTT	CTCCCTTTAG	AATTGGATCA	GATTATTCGT

	890	900	910	920	930	940	950	960
B26	CTTCCCTTCA	ATACATCAAC	TCCACAAGAG	ACTTTATTTT	CAATAAGACA	TTTTGATGAA	CTGGTAGAAC	TCACTTCAAA
LPCoLN	CTTCCCTTCA	ATACATCAAC	TCCACAAGAG	ACTTTATTTT	CAATAAGACA	TTTTGATGAA	CTGGTAGAAC	TCACTTCAAA
AR39	CTTCCCTTCA	ATACATCAAC	TCCACAAGAG	ACTTTATTTT	CAATAAGACA	TTTTGATGAA	CTGGTAGAAC	TCACTTCAAA
CWL029	CTTCCCTTCA	ATACATCAAC	TCCACAAGAG	ACTTTATTTT	CAATAAGACA	TTTTGATGAA	CTGGTAGAAC	TCACTTCAAA
J138	CTTCCCTTCA	ATACATCAAC	TCCACAAGAG	ACTTTATTTT	CAATAAGACA	TTTTGATGAA	CTGGTAGAAC	TCACTTCAAA
TW183	CTTCCCTTCA	ATACATCAAC	TCCACAAGAG	ACTTTATTTT	CAATAAGACA	TTTTGATGAA	CTGGTAGAAC	TCACTTCAAA
TOR1	CTTCCCTTCA	ATACATCAAC	TCCACAAGAG	ACTTTATTTT	CAATAAGACA	TTTTGATGAA	CTGGTAGAAC	TCACTTCAAA
WA97001	CTTCCCTTCA	ATACATCAAC	TCCACAAGAG	ACTTTATTTT	CAATAAGACA	TTTTGATGAA	CTGGTAGAAC	TCACTTCAAA
1979	CTTCCCTTCA	ATACATCAAC	TCCACAAGAG	ACTTTATTTT	CAATAAGACA	TTTTGATGAA	CTGGTAGAAC	TCACTTCAAA
SH511	CTTCCCTTCA	ATACATCAAC	TCCACAAGAG	ACTTTATTTT	CAATAAGACA	TTTTGATGAA	CTGGTAGAAC	TCACTTCAAA

	970	980	990	1,000	1,010	1,020	1,030	1,039
Identity								
B26	ATTAGAATGG	ATGCTCGACC	AAGGTCTGTT	AGAATCAATT	CCCCTTTACA	ATCAAGAGAA	ATATCTTTCT	GGTTTTGAG
LPCoLN	ATTAGAATGG	ATGCTCGACC	AAGGTCTGTT	AGAATCAATT	CCCCTTTACA	ATCAAGAGAA	ATATCTTTCT	GGTTTTGAG
AR39	ATTAGAATGG	ATGCTCGACC	AAGGTCTGTT	AGAATCAATT	CCCCTTTACA	ATCAAGAGAA	ATATCTTTCT	GGTTTTGAG
CWL029	ATTAGAATGG	ATGCTCGACC	AAGGTCTGTT	AGAATCAATT	CCCCTTTACA	ATCAAGAGAA	ATATCTTTCT	GGTTTTGAG
J138	ATTAGAATGG	ATGCTCGACC	AAGGTCTGTT	AGAATCAATT	CCCCTTTACA	ATCAAGAGAA	ATATCTTTCT	GGTTTTGAG
TW183	ATTAGAATGG	ATGCTCGACC	AAGGTCTGTT	AGAATCAATT	CCCCTTTACA	ATCAAGAGAA	ATATCTTTCT	GGTTTTGAG
TOR1	ATTAGAATGG	ATGCTCGACC	AAGGTCTGTT	AGAATCAATT	CCCCTTTACA	ATCAAGAGAA	ATATCTTTCT	GGTTTTGAG
WA97001	ATTAGAATGG	ATGCTCGACC	AAGGTCTGTT	AGAATCAATT	CCCCTTTACA	ATCAAGAGAA	ATATCTTTCT	GGTTTTGAG
1979	ATTAGAATGG	ATGCTCGACC	AAGGTCTGTT	AGAATCAATT	CCCCTTTACA	ATCAAGAGAA	ATATCTTTCT	GGTTTTGAG
SH511	ATTAGAATGG	ATGCTCGACC	AAGGTCTGTT	AGAATCAATT	CCCCTTTACA	ATCAAGAGAA	ATATCTTTCT	GGTTTTGAG

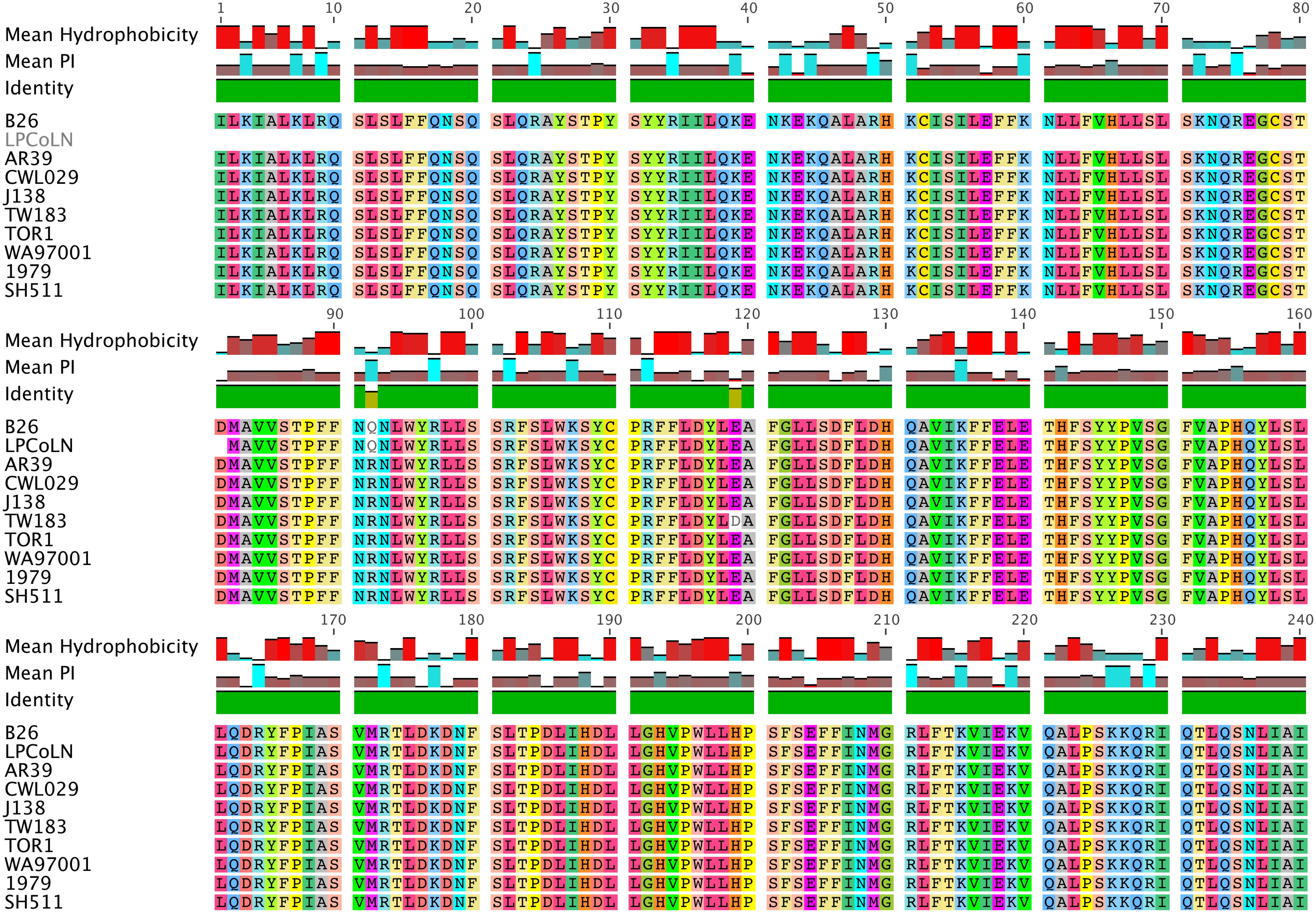
1 10 20 30 40 50 60 70 80
Mean Hydrophobicity
Mean PI
Identity
B26 ILKIALKLRQ SLSLFFQNSQ SLQRAYSTPY SYYRIILQKE NKEKQALARH KCISILEFFK NLLFVHLLSL SKNQREGCST
LPCoLN
AR39 ILKIALKLRQ SLSLFFQNSQ SLQRAYSTPY SYYRIILQKE NKEKQALARH KCISILEFFK NLLFVHLLSL SKNQREGCST
CWL029 ILKIALKLRQ SLSLFFQNSQ SLQRAYSTPY SYYRIILQKE NKEKQALARH KCISILEFFK NLLFVHLLSL SKNQREGCST
J138 ILKIALKLRQ SLSLFFQNSQ SLQRAYSTPY SYYRIILQKE NKEKQALARH KCISILEFFK NLLFVHLLSL SKNQREGCST
TW183 ILKIALKLRQ SLSLFFQNSQ SLQRAYSTPY SYYRIILQKE NKEKQALARH KCISILEFFK NLLFVHLLSL SKNQREGCST
TOR1 ILKIALKLRQ SLSLFFQNSQ SLQRAYSTPY SYYRIILQKE NKEKQALARH KCISILEFFK NLLFVHLLSL SKNQREGCST
WA97001 ILKIALKLRQ SLSLFFQNSQ SLQRAYSTPY SYYRIILQKE NKEKQALARH KCISILEFFK NLLFVHLLSL SKNQREGCST
1979 ILKIALKLRQ SLSLFFQNSQ SLQRAYSTPY SYYRIILQKE NKEKQALARH KCISILEFFK NLLFVHLLSL SKNQREGCST
SH511 ILKIALKLRQ SLSLFFQNSQ SLQRAYSTPY SYYRIILQKE NKEKQALARH KCISILEFFK NLLFVHLLSL SKNQREGCST
90 100 110 120 130 140 150 160
Mean Hydrophobicity
Mean PI
Identity
B26 DMAVVSTPFF NQNLWYRLLS SRFSLWKSYC PRFFLDYLEA FGLLSDFLDH QAVIKFFELE THFSYYPVSG FVAPHQYLSL
LPCoLN MAVVSTPFF NQNLWYRLLS SRFSLWKSYC PRFFLDYLEA FGLLSDFLDH QAVIKFFELE THFSYYPVSG FVAPHQYLSL
AR39 DMAVVSTPFF NRNLWYRLLS SRFSLWKSYC PRFFLDYLEA FGLLSDFLDH QAVIKFFELE THFSYYPVSG FVAPHQYLSL
CWL029 DMAVVSTPFF NRNLWYRLLS SRFSLWKSYC PRFFLDYLEA FGLLSDFLDH QAVIKFFELE THFSYYPVSG FVAPHQYLSL
J138 DMAVVSTPFF NRNLWYRLLS SRFSLWKSYC PRFFLDYLEA FGLLSDFLDH QAVIKFFELE THFSYYPVSG FVAPHQYLSL
TW183 DMAVVSTPFF NRNLWYRLLS SRFSLWKSYC PRFFLDYLDA FGLLSDFLDH QAVIKFFELE THFSYYPVSG FVAPHQYLSL
TOR1 DMAVVSTPFF NRNLWYRLLS SRFSLWKSYC PRFFLDYLEA FGLLSDFLDH QAVIKFFELE THFSYYPVSG FVAPHQYLSL
WA97001 DMAVVSTPFF NRNLWYRLLS SRFSLWKSYC PRFFLDYLEA FGLLSDFLDH QAVIKFFELE THFSYYPVSG FVAPHQYLSL
1979 DMAVVSTPFF NRNLWYRLLS SRFSLWKSYC PRFFLDYLEA FGLLSDFLDH QAVIKFFELE THFSYYPVSG FVAPHQYLSL
SH511 DMAVVSTPFF NRNLWYRLLS SRFSLWKSYC PRFFLDYLEA FGLLSDFLDH QAVIKFFELE THFSYYPVSG FVAPHQYLSL
170 180 190 200 210 220 230 240
Mean Hydrophobicity
Mean PI
Identity
B26 LQDRYFPIAS VMRTLDKDNF SLTPDLIHDL LGHVPWLLHP SFSEFFINMG RLFTKVIEKV QALPSKKQRI QTLQSNLIAI
LPCoLN LQDRYFPIAS VMRTLDKDNF SLTPDLIHDL LGHVPWLLHP SFSEFFINMG RLFTKVIEKV QALPSKKQRI QTLQSNLIAI
AR39 LQDRYFPIAS VMRTLDKDNF SLTPDLIHDL LGHVPWLLHP SFSEFFINMG RLFTKVIEKV QALPSKKQRI QTLQSNLIAI
CWL029 LQDRYFPIAS VMRTLDKDNF SLTPDLIHDL LGHVPWLLHP SFSEFFINMG RLFTKVIEKV QALPSKKQRI QTLQSNLIAI
J138 LQDRYFPIAS VMRTLDKDNF SLTPDLIHDL LGHVPWLLHP SFSEFFINMG RLFTKVIEKV QALPSKKQRI QTLQSNLIAI
TW183 LQDRYFPIAS VMRTLDKDNF SLTPDLIHDL LGHVPWLLHP SFSEFFINMG RLFTKVIEKV QALPSKKQRI QTLQSNLIAI
TOR1 LQDRYFPIAS VMRTLDKDNF SLTPDLIHDL LGHVPWLLHP SFSEFFINMG RLFTKVIEKV QALPSKKQRI QTLQSNLIAI
WA97001 LQDRYFPIAS VMRTLDKDNF SLTPDLIHDL LGHVPWLLHP SFSEFFINMG RLFTKVIEKV QALPSKKQRI QTLQSNLIAI
1979 LQDRYFPIAS VMRTLDKDNF SLTPDLIHDL LGHVPWLLHP SFSEFFINMG RLFTKVIEKV QALPSKKQRI QTLQSNLIAI
SH511 LQDRYFPIAS VMRTLDKDNF SLTPDLIHDL LGHVPWLLHP SFSEFFINMG RLFTKVIEKV QALPSKKQRI QTLQSNLIAI

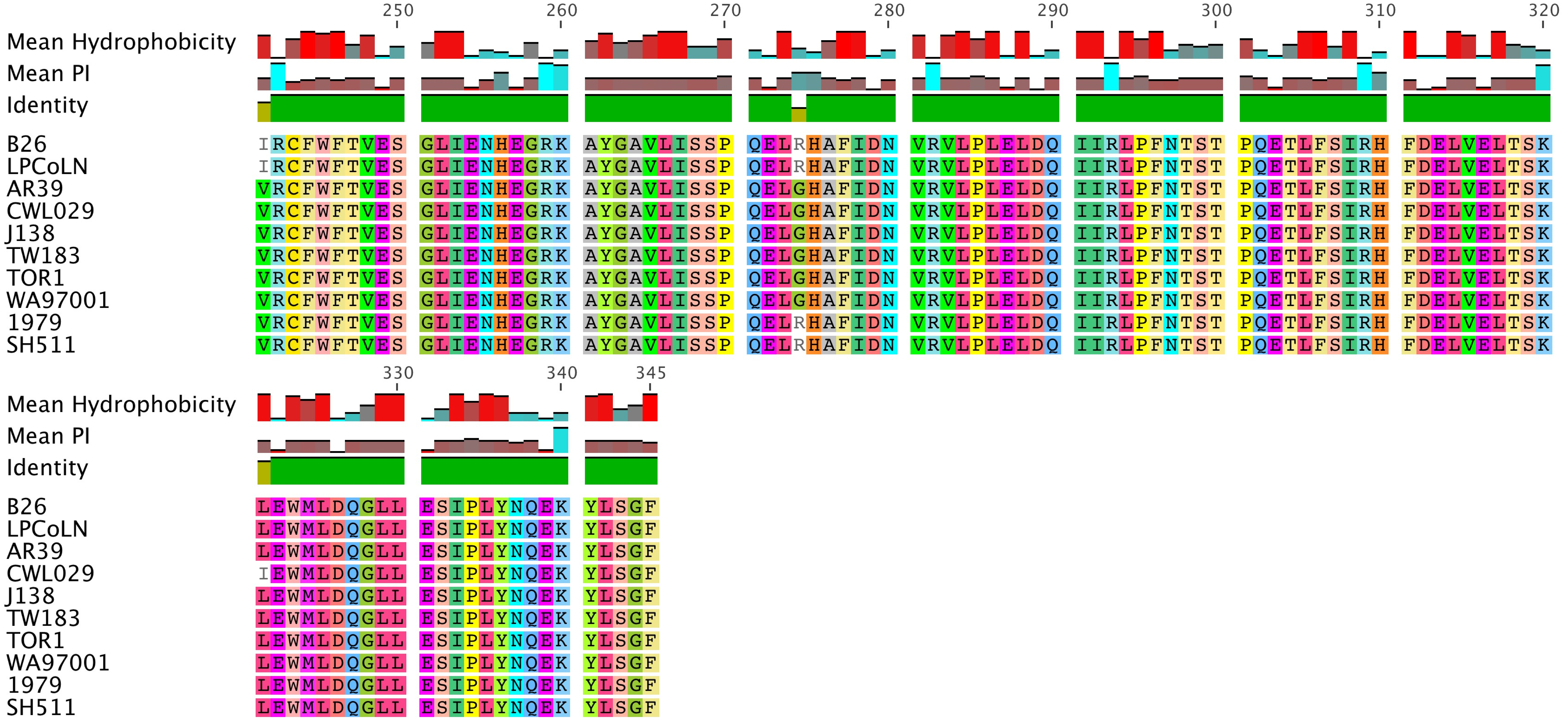
250
260
270
280
290
300
310
320
Mean Hydrophobicity
Mean PI
Identity
B26 IRCFWFTVES GLIENHEGRK AYGAVLISSP QELRHAFIDN VRVLPLELDQ IIRLPFNTST PQETLFSIRH FDELVELTSK
LPCoLN IRCFWFTVES GLIENHEGRK AYGAVLISSP QELRHAFIDN VRVLPLELDQ IIRLPFNTST PQETLFSIRH FDELVELTSK
AR39 VRCFWFTVES GLIENHEGRK AYGAVLISSP QELGHAFIDN VRVLPLELDQ IIRLPFNTST PQETLFSIRH FDELVELTSK
CWL029 VRCFWFTVES GLIENHEGRK AYGAVLISSP QELGHAFIDN VRVLPLELDQ IIRLPFNTST PQETLFSIRH FDELVELTSK
J138 VRCFWFTVES GLIENHEGRK AYGAVLISSP QELGHAFIDN VRVLPLELDQ IIRLPFNTST PQETLFSIRH FDELVELTSK
TW183 VRCFWFTVES GLIENHEGRK AYGAVLISSP QELGHAFIDN VRVLPLELDQ IIRLPFNTST PQETLFSIRH FDELVELTSK
TOR1 VRCFWFTVES GLIENHEGRK AYGAVLISSP QELGHAFIDN VRVLPLELDQ IIRLPFNTST PQETLFSIRH FDELVELTSK
WA97001 VRCFWFTVES GLIENHEGRK AYGAVLISSP QELGHAFIDN VRVLPLELDQ IIRLPFNTST PQETLFSIRH FDELVELTSK
1979 VRCFWFTVES GLIENHEGRK AYGAVLISSP QELRHAFIDN VRVLPLELDQ IIRLPFNTST PQETLFSIRH FDELVELTSK
SH511 VRCFWFTVES GLIENHEGRK AYGAVLISSP QELRHAFIDN VRVLPLELDQ IIRLPFNTST PQETLFSIRH FDELVELTSK
330
340
345
Mean Hydrophobicity
Mean PI
Identity
B26 LEWMLDQGLL ESIPLYNQEK YLSGF
LPCoLN LEWMLDQGLL ESIPLYNQEK YLSGF
AR39 LEWMLDQGLL ESIPLYNQEK YLSGF
CWL029 IEWMLDQGLL ESIPLYNQEK YLSGF
J138 LEWMLDQGLL ESIPLYNQEK YLSGF
TW183 LEWMLDQGLL ESIPLYNQEK YLSGF
TOR1 LEWMLDQGLL ESIPLYNQEK YLSGF
WA97001 LEWMLDQGLL ESIPLYNQEK YLSGF
1979 LEWMLDQGLL ESIPLYNQEK YLSGF
SH511 LEWMLDQGLL ESIPLYNQEK YLSGF
