# Supplementary material for: Chlamydia pneumoniae Is Genetically Diverse in Animals and Appears to Have Crossed the Host Barrier to Humans on (At Least) Two Occasions
Source: PLoS Pathog. 2010 May 20;6(5):e1000903. doi: 10.1371/journal.ppat.1000903 (PMC2873915; doi:10.1371/journal.ppat.1000903)
Supplement: Figure S5 — Multiple sequence alignment of pmp E/F2. The nucleotide and amino acid alignments were generated using Geneious version 4.7, where each nucleotide and amino acid is assigned its own colour. White shading indicates an amino acid variant. (6.27 MB PDF) [file ppat.1000903.s005.pdf]

	1 10	20	30	40	50
Identity					
B26	ATGACTCCTG	CAGTGTATAG	TGTACAAACG	AACTCTCTTG	AAAAGTCTGC
LPCoLN	ATGACTCCTG	CAGTGTATAG	TGTACAAACG	AACTCTCTTG	AAAAGTCTGC
DE177	ATGACTCCTG	CAGTGTATAG	TGTACAAACG	GACTCCCTTG	AAAAGTTTGC
AR39	ATGACTCCTG	CAGTGTATAG	TTTACAAACG	GACTCCCTTG	AAAAGTTTGC
CWL029	ATGACTCCTG	CAGTGTATAG	TTTACAAACG	GACTCCCTTG	AAAAGTTTGC
J138	ATGACTCCTG	CAGTGTATAG	TTTACAAACG	GACTCCCTTG	AAAAGTTTGC
TW183	ATGACTCCTG	CAGTGTATAG	TTTACAAACG	GACTCCCTTG	AAAAGTTTGC
TOR1	ATGACTCCTG	CAGTGTATAG	TTTACAAACG	GACTCCCTTG	AAAAGTTTGC
WA97001	ATGACTCCTG	CAGTGTATAG	TTTACAAACG	GACTCCCTTG	AAAAGTTTGC
1979	ATGACTCCTG	CAGTGTATAG	TGTACAAACG	GACTCCCTTG	AAAAGTTTGC
SH511	ATGACTCCTG	CAGTGTATAG	TGTACAAACG	GACTCCCTTG	AAAAGTTTGC

	60	70	80	90	100
Identity					
B26	TTTGGAGAGA	GATGAAGAGT	TCCGTACGAG	CTTTCCTCTC	TTAGACTCTC
LPCoLN	TTTGGAGAGA	GATGAAGAGT	TCCGTACGAG	CTTTCCTCTC	TTAGACTCTC
DE177	TTTAGAGAGG	GATGAAGAGT	TTCGTACGAG	CTTTCCTCTC	TTAGACTCTC
AR39	TTTAGAGAGG	GATGAAGAGT	TTCGTACGAG	CTTTCCTCTC	TTAGACTCTC
CWL029	TTTAGAGAGG	GATGAAGAGT	TTCGTACGAG	CTTTCCTCTC	TTAGACTCTC
J138	TTTAGAGAGG	GATGAAGAGT	TTCGTACGAG	CTTTCCTCTC	TTAGACTCTC
TW183	TTTAGAGAGG	GATGAAGAGT	TTCGTACGAG	CTTTCCTCTC	TTAGACTCTC
TOR1	TTTAGAGAGG	GATGAAGAGT	TTCGTACGAG	CTTTCCTCTC	TTAGACTCTC
WA97001	TTTAGAGAGG	GATGAAGAGT	TTCGTACGAG	CTTTCCTCTC	TTAGACTCTC
1979	TTTAGAGAGG	GATGAAGAGT	TTCGTACGAG	CTTTCCTCTC	TTAGACTCTC
SH511	TTTAGAGAGG	GATGAAGAGT	TTCGTACGAG	CTTTCCTCTC	TTAGACTCTC

	110	120	130	140	150
Identity					
B26	TCTCCACCCT	TACAGGATTT	TCTCCAATAA	CTACATTTGT	TGGAAATAGA
LPCoLN	TCTCCACCCT	TACAGGATTT	TCTCCAATAA	CTACATTTGT	TGGAAATAGA
DE177	TCTCCACTCT	TACAGGATTT	TCTCCAATAA	CTACGTTTGT	TGGAAATAGA
AR39	TCTCCACTCT	TACAGGATTT	TCTCCAATAA	CTACGTTTGT	TGGAAATAGA
CWL029	TCTCCACTCT	TACAGGATTT	TCTCCAATAA	CTACGTTTGT	TGGAAATAGA
J138	TCTCCACTCT	TACAGGATTT	TCTCCAATAA	CTACGTTTGT	TGGAAATAGA
TW183	TCTCCACTCT	TACAGGATTT	TCTCCAATAA	CTACGTTTGT	TGGAAATAGA
TOR1	TCTCCACTCT	TACAGGATTT	TCTCCAATAA	CTACGTTTGT	TGGAAATAGA
WA97001	TCTCCACTCT	TACAGGATTT	TCTCCAATAA	CTACGTTTGT	TGGAAATAGA
1979	TCTCCACTCT	TACAGGATTT	TCTCCAATAA	CTACGTTTGT	TGGAAATAGA
SH511	TCTCCACTCT	TACAGGATTT	TCTCCAATAA	CTACGTTTGT	TGGAAATAGA

	160	170	180	190	200
Identity					
B26	CATAATTCCC	CTCAAGATAT	TGTACTTTCT	AACTACAAGT	CTATTGATAA
LPCoLN	CATAATTCCC	CTCAAGATAT	TGTACTTTCT	AACTACAAGT	CTATTGATAA
DE177	CATAATTCCT	CTCAAGACAT	TGTACTTTCT	AACTACAAGT	CTATTGATAA
AR39	CATAATTCCT	CTCAAGACAT	TGTACTTTCT	AACTACAAGT	CTATTGATAA
CWL029	CATAATTCCT	CTCAAGACAT	TGTACTTTCT	AACTACAAGT	CTATTGATAA
J138	CATAATTCCT	CTCAAGACAT	TGTACTTTCT	AACTACAAGT	CTATTGATAA
TW183	CATAATTCCT	CTCAAGACAT	TGTACTTTCT	AACTACAAGT	CTATTGATAA
TOR1	CATAATTCCT	CTCAAGACAT	TGTACTTTCT	AACTACAAGT	CTATTGATAA
WA97001	CATAATTCCT	CTCAAGACAT	TGTACTTTCT	AACTACAAGT	CTATTGATAA
1979	CATAATTCCT	CTCAAGACAT	TGTACTTTCT	AACTACAAGT	CTATTGATAA
SH511	CATAATTCCT	CTCAAGACAT	TGTACTTTCT	AACTACAAGT	CTATTGATAA

	210	220	230	240	250
Identity					
B26	CATCCTTCTT	CTTTGGACAT	CGGCTGGGGG	AGCTTTGTCC	TGTAATAATT
LPCoLN	CATCCTTCTT	CTTTGGACAT	CGGCTGGGGG	AGCTTTGTCC	TGTAATAATT
DE177	CATCCTTCTT	CTTTGGACAT	CGGCTGGGGG	AGCTGTGTCC	TGTAATAATT
AR39	CATCCTTCTT	CTTTGGACAT	CGGCTGGGGG	AGCTGTGTCC	TGTAATAATT
CWL029	CATCCTTCTT	CTTTGGACAT	CGGCTGGGGG	AGCTGTGTCC	TGTAATAATT
J138	CATCCTTCTT	CTTTGGACAT	CGGCTGGGGG	AGCTGTGTCC	TGTAATAATT
TW183	CATCCTTCTT	CTTTGGACAT	CGGCTGGGGG	AGCTGTGTCC	TGTAATAATT
TOR1	CATCCTTCTT	CTTTGGACAT	CGGCTGGGGG	AGCTGTGTCC	TGTAATAATT
WA97001	CATCCTTCTT	CTTTGGACAT	CGGCTGGGGG	AGCTGTGTCC	TGTAATAATT
1979	CATCCTTCTT	CTTTGGACAT	CGGCTGGGGG	AGCTGTGTCC	TGTAATAATT
SH511	CATCCTTCTT	CTTTGGACAT	CGGCTGGGGG	AGCTGTGTCC	TGTAATAATT

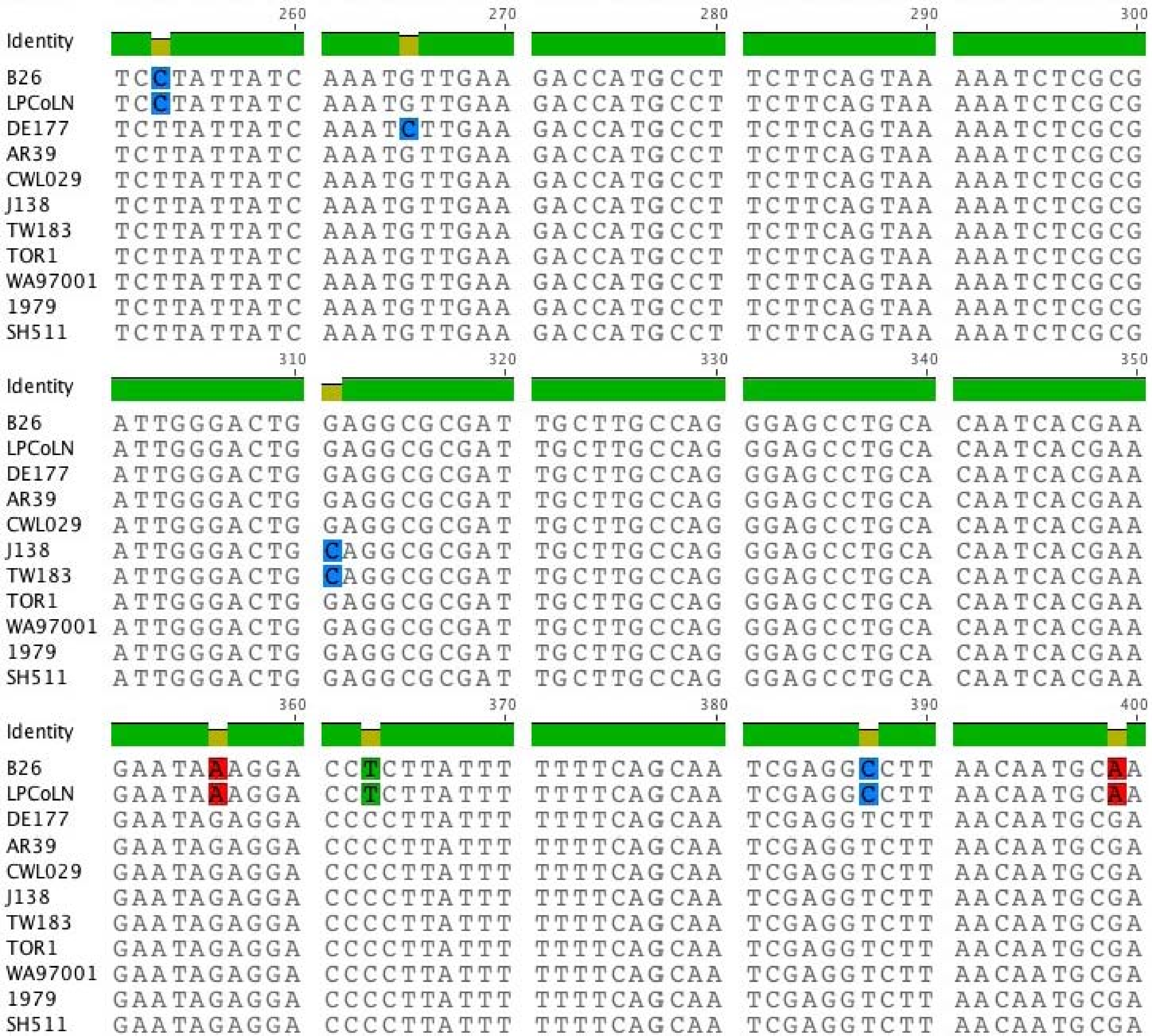

260 270 280 290 300
Identity
B26 TCCTATTATC AAATGTTGAA GACCATGCCT TCTTCAGTAA AAATCTCGCG
LPCoLN TCCTATTATC AAATGTTGAA GACCATGCCT TCTTCAGTAA AAATCTCGCG
DE177 TCTTATTATC AAATCTTGAA GACCATGCCT TCTTCAGTAA AAATCTCGCG
AR39 TCTTATTATC AAATGTTGAA GACCATGCCT TCTTCAGTAA AAATCTCGCG
CWL029 TCTTATTATC AAATGTTGAA GACCATGCCT TCTTCAGTAA AAATCTCGCG
J138 TCTTATTATC AAATGTTGAA GACCATGCCT TCTTCAGTAA AAATCTCGCG
TW183 TCTTATTATC AAATGTTGAA GACCATGCCT TCTTCAGTAA AAATCTCGCG
TOR1 TCTTATTATC AAATGTTGAA GACCATGCCT TCTTCAGTAA AAATCTCGCG
WA97001 TCTTATTATC AAATGTTGAA GACCATGCCT TCTTCAGTAA AAATCTCGCG
1979 TCTTATTATC AAATGTTGAA GACCATGCCT TCTTCAGTAA AAATCTCGCG
SH511 TCTTATTATC AAATGTTGAA GACCATGCCT TCTTCAGTAA AAATCTCGCG
310 320 330 340 350
Identity
B26 ATTGGGACTG GAGGCGCGAT TGCTTGCCAG GGAGCCTGCA CAATCACGAA
LPCoLN ATTGGGACTG GAGGCGCGAT TGCTTGCCAG GGAGCCTGCA CAATCACGAA
DE177 ATTGGGACTG GAGGCGCGAT TGCTTGCCAG GGAGCCTGCA CAATCACGAA
AR39 ATTGGGACTG GAGGCGCGAT TGCTTGCCAG GGAGCCTGCA CAATCACGAA
CWL029 ATTGGGACTG GAGGCGCGAT TGCTTGCCAG GGAGCCTGCA CAATCACGAA
J138 ATTGGGACTG CAGGCGCGAT TGCTTGCCAG GGAGCCTGCA CAATCACGAA
TW183 ATTGGGACTG CAGGCGCGAT TGCTTGCCAG GGAGCCTGCA CAATCACGAA
TOR1 ATTGGGACTG GAGGCGCGAT TGCTTGCCAG GGAGCCTGCA CAATCACGAA
WA97001 ATTGGGACTG GAGGCGCGAT TGCTTGCCAG GGAGCCTGCA CAATCACGAA
1979 ATTGGGACTG GAGGCGCGAT TGCTTGCCAG GGAGCCTGCA CAATCACGAA
SH511 ATTGGGACTG GAGGCGCGAT TGCTTGCCAG GGAGCCTGCA CAATCACGAA
360 370 380 390 400
Identity
B26 GAATAAAGGA CCTCTTATTT TTTTCAGCAA TCGAGGCCTT AACAATGCAA
LPCoLN GAATAAAGGA CCTCTTATTT TTTTCAGCAA TCGAGGCCTT AACAATGCAA
DE177 GAATAGAGGA CCCCTTATTT TTTTCAGCAA TCGAGGTCTT AACAATGCGA
AR39 GAATAGAGGA CCCCTTATTT TTTTCAGCAA TCGAGGTCTT AACAATGCGA
CWL029 GAATAGAGGA CCCCTTATTT TTTTCAGCAA TCGAGGTCTT AACAATGCGA
J138 GAATAGAGGA CCCCTTATTT TTTTCAGCAA TCGAGGTCTT AACAATGCGA
TW183 GAATAGAGGA CCCCTTATTT TTTTCAGCAA TCGAGGTCTT AACAATGCGA
TOR1 GAATAGAGGA CCCCTTATTT TTTTCAGCAA TCGAGGTCTT AACAATGCGA
WA97001 GAATAGAGGA CCCCTTATTT TTTTCAGCAA TCGAGGTCTT AACAATGCGA
1979 GAATAGAGGA CCCCTTATTT TTTTCAGCAA TCGAGGTCTT AACAATGCGA
SH511 GAATAGAGGA CCCCTTATTT TTTTCAGCAA TCGAGGTCTT AACAATGCGA

	410	420	430	440	450
Identity					
B26	GTACAGGAGG	AGAAACCCGT	GGGGGTGCGA	TTGCCTGTAA	TGGAGACTTT
LPCoLN	GTACAGGAGG	AGAAACCCGT	GGGGGTGCGA	TTGCCTGTAA	TGGAGACTTT
DE177	TTACAGGAGG	AGAAACTCGT	GGAGGTGCGA	TTGCCTGTAA	TGGAGACTTC
AR39	GTACAGGAGG	AGAAACTCGT	GGGGGTGCGA	TTGCCTGTAA	TGGAGACTTC
CWL029	GTACAGGAGG	AGAAACTCGT	GGGGGTGCGA	TTGCCTGTAA	TGGAGACTTC
J138	GTACAGGAGG	AGAAACTCGT	GGGGGTGCGA	TTGCCTGTAA	TGGAGACTTC
TW183	GTACAGGAGG	AGAAACTCGT	GGGGGTGCGA	TTGCCTGTAA	TGGAGACTTC
TOR1	GTACAGGAGG	AGAAACTCGT	GGGGGTGCGA	TTGCCTGTAA	TGGAGACTTC
WA97001	GTACAGGAGG	AGAAACTCGT	GGGGGTGCGA	TTGCCTGTAA	TGGAGACTTC
1979	GTACAGGAGG	AGAAACTCGT	GGGGGTGCGA	TTGCCTGTAA	TGGAGACTTC
SH511	GTACAGGAGG	AGAAACTCGT	GGGGGTGCGA	TTGCCTGTAA	TGGAGACTTC

	460	470	480	490	500
Identity					
B26	ATGATTTCTC	AAAATCAAGG	GACTTTCTAC	TTTGTCAACA	ATTCCGTCAA
LPCoLN	ATGATTTCTC	AAAATCAAGG	GACTTTCTAC	TTTGTCAACA	ATTCCGTCAA
DE177	ACGATTTCTC	AAAATCAAGG	GACTTTCTAC	TTTGTCAACA	ATTCCGTCAA
AR39	ACGATTTCTC	AAAATCAAGG	GACTTTCTAC	TTTGTCAACA	ATTCCGTCAA
CWL029	ACGATTTCTC	AAAATCAAGG	GACTTTCTAC	TTTGTCAACA	ATTCCGTCAA
J138	ACGATTTCTC	AAAATCAAGG	GACTTTCTAC	TTTGTCAACA	ATTCCGTCAA
TW183	ACGATTTCTC	AAAATCAAGG	GACTTTCTAC	TTTGTCAACA	ATTCCGTCAA
TOR1	ACGATTTCTC	AAAATCAAGG	GACTTTCTAC	TTTGTCAACA	ATTCCGTCAA
WA97001	ACGATTTCTC	AAAATCAAGG	GACTTTCTAC	TTTGTCAACA	ATTCCGTCAA
1979	ACGATTTCTC	AAAATCAAGG	GACTTTCTAC	TTTGTCAACA	ATTCCGTCAA
SH511	ACGATTTCTC	AAAATCAAGG	GACTTTCTAC	TTTGTCAACA	ATTCCGTCAA

	510	520	530	540	550
Identity					
B26	CAACTGGGGA	GGTGCTCTCT	CCACCAATGG	ACACTGCCGC	ATTCAAAGTA
LPCoLN	CAACTGGGGA	GGTGCTCTCT	CCACCAATGG	ACACTGCCGC	ATTCAAAGTA
DE177	CAACTGGGGA	GGAGCCCTCT	CCACCAATGG	ACACTGCCGC	ATCCAAAGCA
AR39	CAACTGGGGA	GGAGCCCTCT	CCACCAATGG	ACACTGCCGC	ATCCAAAGCA
CWL029	CAACTGGGGA	GGAGCCCTCT	CCACCAATGG	ACACTGCCGC	ATCCAAAGCA
J138	CAACTGGGGA	GGAGCCCTCT	CCACCAATGG	ACACTGCCGC	ATCCAAAGCA
TW183	CAACTGGGGA	GGAGCCCTCT	CCACCAATGG	ACACTGCCGC	ATCCAAAGCA
TOR1	CAACTGGGGA	GGAGCCCTCT	CCACCAATGG	ACACTGCCGC	ATCCAAAGCA
WA97001	CAACTGGGGA	GGAGCCCTCT	CCACCAATGG	ACACTGCCGC	ATCCAAAGCA
1979	CAACTGGGGA	GGAGCCCTCT	CCACCAATGG	ACACTGCCGC	ATCCAAAGCA
SH511	CAACTGGGGA	GGAGCCCTCT	CCACCAATGG	ACACTGCCGC	ATCCAAAGCA

	560	570	580	590	600
Identity					
B26	ACACAGGACC	CTTACTCTTT	TTTAACAATA	CTGCTCCTAG	TGCAGGAGGC
LPCoLN	ACACAGGACC	CTTACTCTTT	TTTAACAATA	CTGCTCCTAG	TGCAGGAGGC
DE177	ACAGGGCACC	TCTACTCTTT	TTTAACAATA	CAGCCCCTAG	TGGAGGGGGT
AR39	ACAGGGCACC	TCTACTCTTT	TTTAACAATA	CAGCCCCTAG	TGGAGGGGGT
CWL029	ACAGGGCACC	TCTACTCTTT	TTTAACAATA	CAGCCCCTAG	TGGAGGGGGT
J138	ACAGGGCACC	TCTACTCTTT	TTTAACAATA	CAGCCCCTAG	TGGAGGGGGT
TW183	ACAGGGCACC	TCTACTCTTT	TTTAACAATA	CAGCCCCTAG	TGGAGGGGGT
TOR1	ACAGGGCACC	TCTACTCTTT	TTTAACAATA	CAGCCCCTAG	TGGAGGGGGT
WA97001	ACAGGGCACC	TCTACTCTTT	TTTAACAATA	CAGCCCCTAG	TGGAGGGGGT
1979	ACAGGGCACC	TCTACTCTTT	TTTAACAATA	CAGCCCCTAG	TGGAGGGGGT
SH511	ACAGGGCACC	TCTACTCTTT	TTTAACAATA	CAGCCCCTAG	TGGAGGGGGT

	610	620	630	640	650
Identity					
B26	GCGCTTCGTA	GTGAAAATAC	AACGATCTCT	GATAACACAC	GTCCTATTTA
LPCoLN	GCGCTTCGTA	GTGAAAATAC	AACGATCTCT	GATAACACAC	GTCCTATTTA
DE177	GCGCTTCGTA	GTGAAAATAC	AACGATCTCT	GATAACACGC	GTCCTATTTA
AR39	GCGCTTCGTA	GTGAAAATAC	AACGATCTCT	GATAACACGC	GTCCTATTTA
CWL029	GCGCTTCGTA	GTGAAAATAC	AACGATCTCT	GATAACACGC	GTCCTATTTA
J138	GCGCTTCGTA	GTGAAAATAC	AACGATCTCT	GATAACACGC	GTCCTATTTA
TW183	GCGCTTCGTA	GTGAAAATAC	AACGATCTCT	GATAACACGC	GTCCTATTTA
TOR1	GCGCTTCGTA	GTGAAAATAC	AACGATCTCT	GATAACACGC	GTCCTATTTA
WA97001	GCGCTTCGTA	GTGAAAATAC	AACGATCTCT	GATAACACGC	GTCCTATTTA
1979	GCGCTTCGTA	GTGAAAATAC	AACGATCTCT	GATAACACGC	GTCCTATTTA
SH511	GCGCTTCGTA	GTGAAAATAC	AACGATCTCT	GATAACACGC	GTCCTATTTA

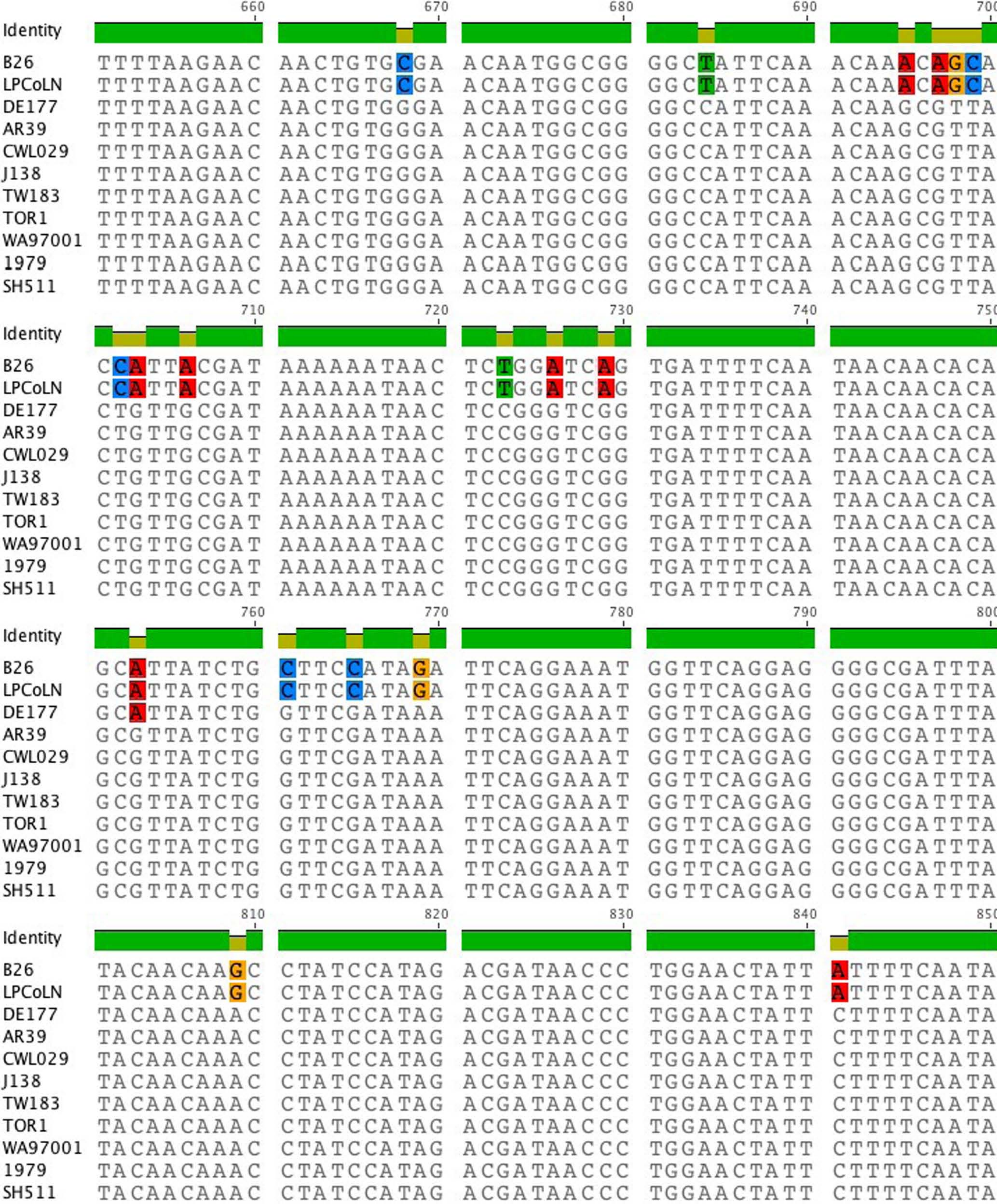
660 670 680 690 700
Identity
B26 TTTTAAGAAC AACTGTGCGA ACAATGGCGG GGCTATTCAA ACAAACAGCA
LPCoLN TTTTAAGAAC AACTGTGCGA ACAATGGCGG GGCTATTCAA ACAAACAGCA
DE177 TTTTAAGAAC AACTGTGGGA ACAATGGCGG GGCCATTCAA ACAAGCGTTA
AR39 TTTTAAGAAC AACTGTGGGA ACAATGGCGG GGCCATTCAA ACAAGCGTTA
CWL029 TTTTAAGAAC AACTGTGGGA ACAATGGCGG GGCCATTCAA ACAAGCGTTA
J138 TTTTAAGAAC AACTGTGGGA ACAATGGCGG GGCCATTCAA ACAAGCGTTA
TW183 TTTTAAGAAC AACTGTGGGA ACAATGGCGG GGCCATTCAA ACAAGCGTTA
TOR1 TTTTAAGAAC AACTGTGGGA ACAATGGCGG GGCCATTCAA ACAAGCGTTA
WA97001 TTTTAAGAAC AACTGTGGGA ACAATGGCGG GGCCATTCAA ACAAGCGTTA
1979 TTTTAAGAAC AACTGTGGGA ACAATGGCGG GGCCATTCAA ACAAGCGTTA
SH511 TTTTAAGAAC AACTGTGGGA ACAATGGCGG GGCCATTCAA ACAAGCGTTA
710 720 730 740 750
Identity
B26 CCATTACGAT AAAAAATAAC TCTGGATCAG TGATTTTCAA TAACAACACA
LPCoLN CCATTACGAT AAAAAATAAC TCTGGATCAG TGATTTTCAA TAACAACACA
DE177 CTGTTGCGAT AAAAAATAAC TCCGGGTCGG TGATTTTCAA TAACAACACA
AR39 CTGTTGCGAT AAAAAATAAC TCCGGGTCGG TGATTTTCAA TAACAACACA
CWL029 CTGTTGCGAT AAAAAATAAC TCCGGGTCGG TGATTTTCAA TAACAACACA
J138 CTGTTGCGAT AAAAAATAAC TCCGGGTCGG TGATTTTCAA TAACAACACA
TW183 CTGTTGCGAT AAAAAATAAC TCCGGGTCGG TGATTTTCAA TAACAACACA
TOR1 CTGTTGCGAT AAAAAATAAC TCCGGGTCGG TGATTTTCAA TAACAACACA
WA97001 CTGTTGCGAT AAAAAATAAC TCCGGGTCGG TGATTTTCAA TAACAACACA
1979 CTGTTGCGAT AAAAAATAAC TCCGGGTCGG TGATTTTCAA TAACAACACA
SH511 CTGTTGCGAT AAAAAATAAC TCCGGGTCGG TGATTTTCAA TAACAACACA
760 770 780 790 800
Identity
B26 GCATTATCTG CTTCCATAGA TTCAGGAAAT GGTTCAGGAG GGGCGATTTA
LPCoLN GCATTATCTG CTTCCATAGA TTCAGGAAAT GGTTCAGGAG GGGCGATTTA
DE177 GCATTATCTG GTTCGATAAA TTCAGGAAAT GGTTCAGGAG GGGCGATTTA
AR39 GCGTTATCTG GTTCGATAAA TTCAGGAAAT GGTTCAGGAG GGGCGATTTA
CWL029 GCGTTATCTG GTTCGATAAA TTCAGGAAAT GGTTCAGGAG GGGCGATTTA
J138 GCGTTATCTG GTTCGATAAA TTCAGGAAAT GGTTCAGGAG GGGCGATTTA
TW183 GCGTTATCTG GTTCGATAAA TTCAGGAAAT GGTTCAGGAG GGGCGATTTA
TOR1 GCGTTATCTG GTTCGATAAA TTCAGGAAAT GGTTCAGGAG GGGCGATTTA
WA97001 GCGTTATCTG GTTCGATAAA TTCAGGAAAT GGTTCAGGAG GGGCGATTTA
1979 GCGTTATCTG GTTCGATAAA TTCAGGAAAT GGTTCAGGAG GGGCGATTTA
SH511 GCGTTATCTG GTTCGATAAA TTCAGGAAAT GGTTCAGGAG GGGCGATTTA
810 820 830 840 850
Identity
B26 TACAACAAGC CTATCCATAG ACGATAACCC TGGAACTATT ATTTTCAATA
LPCoLN TACAACAAGC CTATCCATAG ACGATAACCC TGGAACTATT ATTTTCAATA
DE177 TACAACAAAC CTATCCATAG ACGATAACCC TGGAACTATT CTTTTCAATA
AR39 TACAACAAAC CTATCCATAG ACGATAACCC TGGAACTATT CTTTTCAATA
CWL029 TACAACAAAC CTATCCATAG ACGATAACCC TGGAACTATT CTTTTCAATA
J138 TACAACAAAC CTATCCATAG ACGATAACCC TGGAACTATT CTTTTCAATA
TW183 TACAACAAAC CTATCCATAG ACGATAACCC TGGAACTATT CTTTTCAATA
TOR1 TACAACAAAC CTATCCATAG ACGATAACCC TGGAACTATT CTTTTCAATA
WA97001 TACAACAAAC CTATCCATAG ACGATAACCC TGGAACTATT CTTTTCAATA
1979 TACAACAAAC CTATCCATAG ACGATAACCC TGGAACTATT CTTTTCAATA
SH511 TACAACAAAC CTATCCATAG ACGATAACCC TGGAACTATT CTTTTCAATA

Identity	860	870	880	890	900
B26	ACAACTACTG	CAGTCGCGAT	GGCGGAGCTA	TCTGTATACA	ATCTTTGACA
LPCoLN	ACAACTACTG	CAGTCGCGAT	GGCGGAGCTA	TCTGTATACA	ATCTTTGACA
DE177	ATAACTACTG	CAGTCGCGAT	GGCGGAGCTA	TCTGTACACA	ATCTTTGACA
AR39	ATAACTACTG	CATTCGCGAT	GGCGGAGCTA	TCTGTACACA	ATTTTTGACA
CWL029	ATAACTACTG	CATTCGCGAT	GGCGGAGCTA	TCTGTACACA	ATTTTTGACA
J138	ATAACTACTG	CATTCGCGAT	GGCGGAGCTA	TCTGTACACA	ATTTTTGACA
TW183	ATAACTACTG	CATTCGCGAT	GGCGGAGCTA	TCTGTACACA	ATTTTTGACA
TOR1	ATAACTACTG	CATTCGCGAT	GGCGGAGCTA	TCTGTACACA	ATTTTTGACA
WA97001	ATAACTACTG	CATTCGCGAT	GGCGGAGCTA	TCTGTACACA	ATTTTTGACA
1979	ATAACTACTG	CATTCGCGAT	GGCGGAGCTA	TCTGTACACA	ATCTTTGACA
SH511	ATAACTACTG	CATTCGCGAT	GGCGGAGCTA	TCTGTACACA	ATCTTTGACA

Identity	910	920	930	940	950
B26	ATCAAAAATA	GTGGCCACGT	ATATTTCACC	AACAATCAAG	GAAACTGGGG
LPCoLN	ATCAAAAATA	GTGGCCACGT	ATATTTCACC	AACAATCAAG	GAAACTGGGG
DE177	ATCAAAAATA	GTGGCCACGT	ATATTTCACC	AACAATCAAG	GAAACTGGGG
AR39	ATCAAAAATA	GTGGCCACGT	ATATTTCACC	AACAATCAAG	GAAACTGGGG
CWL029	ATCAAAAATA	GTGGCCACGT	ATATTTCACC	AACAATCAAG	GAAACTGGGG
J138	ATCAAAAATA	GTGGCCACGT	ATATTTCACC	AACAATCAAG	GAAACTGGGG
TW183	ATCAAAAATA	GTGGCCACGT	ATATTTCACC	AACAATCAAG	GAAACTGGGG
TOR1	ATCAAAAATA	GTGGCCACGT	ATATTTCACC	AACAATCAAG	GAAACTGGGG
WA97001	ATCAAAAATA	GTGGCCACGT	ATATTTCACC	AACAATCAAG	GAAACTGGGG
1979	ATCAAAAATA	GTGGCCACGT	ATATTTCACC	AACAATCAAG	GAAACTGGGG
SH511	ATCAAAAATA	GTGGCCACGT	ATATTTCACC	AACAATCAAG	GAAACTGGGG

Identity	960	970	980	990	1,000
B26	TGGTGCTCTT	ATGCTCCGAC	GGGACAGCAC	CTGCCTACTC	TTCGCGGAAC
LPCoLN	TGGTGCTCTT	ATGCTCCGAC	GGGACAGCAC	CTGCCTACTC	TTCGCGGAAC
DE177	AGGTGCTCTT	ATGCTCCGAC	AGGACAGCAC	CTGCCTACTC	TTCGCGGAAC
AR39	AGGTGCTCTT	ATGCTCCTAC	AGGACAGCAC	CTGCCTACTC	TTCGCGGAAC
CWL029	AGGTGCTCTT	ATGCTCCTAC	AGGACAGCAC	CTGCCTACTC	TTCGCGGAAC
J138	AGGTGCTCTT	ATGCTCCTAC	AGGACAGCAC	CTGCCTACTC	TTCGCGGAAC
TW183	AGGTGCTCTT	ATGCTCCTAC	AGGACAGCAC	CTGCCTACTC	TTCGCGGAAC
TOR1	AGGTGCTCTT	ATGCTCCTAC	AGGACAGCAC	CTGCCTACTC	TTCGCGGAAC
WA97001	AGGTGCTCTT	ATGCTCCTAC	AGGACAGCAC	CTGCCTACTC	TTCGCGGAAC
1979	AGGTGCTCTT	ATGCTCCGAC	AGGACAGCAC	CTGCCTACTC	TTCGCGGAAC
SH511	AGGTGCTCTT	ATGCTCCGAC	AGGACAGCAC	CTGCCTACTC	TTCGCGGAAC

Identity	1,010	1,020	1,030	1,040	1,050
B26	AAGGAAATAT	CGCATTTCAA	AATAATGAGG	TTTTCCTCAC	CGCATTTGGT
LPCoLN	AAGGAAATAT	CGCATTTCAA	AATAATGAGG	TTTTCCTCAC	CGCATTTGGT
DE177	AAGGAAATAT	CGCATTTCAA	AATAATGAGG	TTTTCCTCAC	CACATTTGGT
AR39	AAGGAAATAT	CGCATTTCAA	AATAATGAGG	TTTTCCTCAC	CACATTTGGT
CWL029	AAGGAAATAT	CGCATTTCAA	AATAATGAGG	TTTTCCTCAC	CACATTTGGT
J138	AAGGAAATAT	CGCATTTCAA	AATAATGAGG	TTTTCCTCAC	CACATTTGGT
TW183	AAGGAAATAT	CGCATTTCAA	AATAATGAGG	TTTTCCTCAC	CACATTTGGT
TOR1	AAGGAAATAT	CGCATTTCAA	AATAATGAGG	TTTTCCTCAC	CACATTTGGT
WA97001	AAGGAAATAT	CGCATTTCAA	AATAATGAGG	TTTTCCTCAC	CACATTTGGT
1979	AAGGAAATAT	CGCATTTCAA	AATAATGAGG	TTTTCCTCAC	CACATTTGGT
SH511	AAGGAAATAT	CGCATTTCAA	AATAATGAGG	TTTTCCTCAC	CACATTTGGT

Identity	1,060	1,070	1,080	1,090	1,100
B26	AGATACAACG	CCATACATTG	TACACCCAAT	AGCAACTTAC	AACTTGGAGC
LPCoLN	AGATACAACG	CCATACATTG	TACACCCAAT	AGCAACTTAC	AACTTGGAGC
DE177	AGATACAACG	CCATACATTG	TACACCAAAT	AGCAACTTAC	AACTTGGAGC
AR39	AGATACAACG	CCATACATTG	TACACCAAAT	AGCAACTTAC	AACTTGGAGC
CWL029	AGATACAACG	CCATACATTG	TACACCAAAT	AGCAACTTAC	AACTTGGAGC
J138	AGATACAACG	CCATACATTG	TACACCAAAT	AGCAACTTAC	AACTTGGAGC
TW183	AGATACAACG	CCATACATTG	TACACCAAAT	AGCAACTTAC	AACTTGGAGC
TOR1	AGATACAACG	CCATACATTG	TACACCAAAT	AGCAACTTAC	AACTTGGAGC
WA97001	AGATACAACG	CCATACATTG	TACACCAAAT	AGCAACTTAC	AACTTGGAGC
1979	AGATACAACG	CCATACATTG	TACACCAAAT	AGCAACTTAC	AACTTGGAGC
SH511	AGATACAACG	CCATACATTG	TACACCAAAT	AGCAACTTAC	AACTTGGAGC

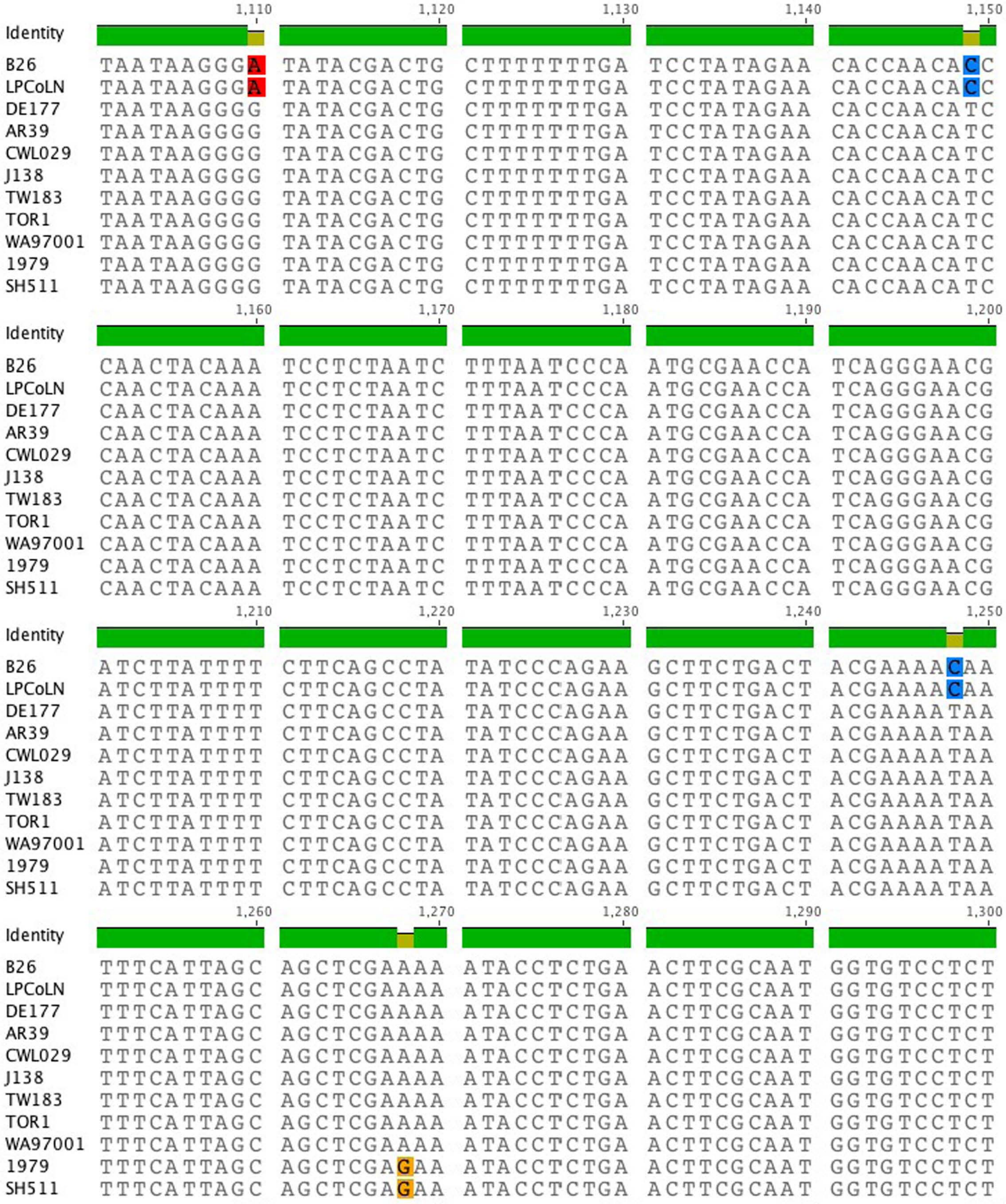
1,110 1,120 1,130 1,140 1,150
Identity
B26 TAATAAGGGA TATACGACTG CTTTTTTTGA TCCTATAGAA CACCAACACC
LPCoLN TAATAAGGGA TATACGACTG CTTTTTTTGA TCCTATAGAA CACCAACACC
DE177 TAATAAGGGG TATACGACTG CTTTTTTTGA TCCTATAGAA CACCAACATC
AR39 TAATAAGGGG TATACGACTG CTTTTTTTGA TCCTATAGAA CACCAACATC
CWL029 TAATAAGGGG TATACGACTG CTTTTTTTGA TCCTATAGAA CACCAACATC
J138 TAATAAGGGG TATACGACTG CTTTTTTTGA TCCTATAGAA CACCAACATC
TW183 TAATAAGGGG TATACGACTG CTTTTTTTGA TCCTATAGAA CACCAACATC
TOR1 TAATAAGGGG TATACGACTG CTTTTTTTGA TCCTATAGAA CACCAACATC
WA97001 TAATAAGGGG TATACGACTG CTTTTTTTGA TCCTATAGAA CACCAACATC
1979 TAATAAGGGG TATACGACTG CTTTTTTTGA TCCTATAGAA CACCAACATC
SH511 TAATAAGGGG TATACGACTG CTTTTTTTGA TCCTATAGAA CACCAACATC
1,160 1,170 1,180 1,190 1,200
Identity
B26 CAACTACAAA TCCTCTAATC TTTAATCCCA ATGCGAACCA TCAGGGAACG
LPCoLN CAACTACAAA TCCTCTAATC TTTAATCCCA ATGCGAACCA TCAGGGAACG
DE177 CAACTACAAA TCCTCTAATC TTTAATCCCA ATGCGAACCA TCAGGGAACG
AR39 CAACTACAAA TCCTCTAATC TTTAATCCCA ATGCGAACCA TCAGGGAACG
CWL029 CAACTACAAA TCCTCTAATC TTTAATCCCA ATGCGAACCA TCAGGGAACG
J138 CAACTACAAA TCCTCTAATC TTTAATCCCA ATGCGAACCA TCAGGGAACG
TW183 CAACTACAAA TCCTCTAATC TTTAATCCCA ATGCGAACCA TCAGGGAACG
TOR1 CAACTACAAA TCCTCTAATC TTTAATCCCA ATGCGAACCA TCAGGGAACG
WA97001 CAACTACAAA TCCTCTAATC TTTAATCCCA ATGCGAACCA TCAGGGAACG
1979 CAACTACAAA TCCTCTAATC TTTAATCCCA ATGCGAACCA TCAGGGAACG
SH511 CAACTACAAA TCCTCTAATC TTTAATCCCA ATGCGAACCA TCAGGGAACG
1,210 1,220 1,230 1,240 1,250
Identity
B26 ATCTTATTTT CTTCAGCCTA TATCCCAGAA GCTTCTGACT ACGAAAACAA
LPCoLN ATCTTATTTT CTTCAGCCTA TATCCCAGAA GCTTCTGACT ACGAAAACAA
DE177 ATCTTATTTT CTTCAGCCTA TATCCCAGAA GCTTCTGACT ACGAAAATAA
AR39 ATCTTATTTT CTTCAGCCTA TATCCCAGAA GCTTCTGACT ACGAAAATAA
CWL029 ATCTTATTTT CTTCAGCCTA TATCCCAGAA GCTTCTGACT ACGAAAATAA
J138 ATCTTATTTT CTTCAGCCTA TATCCCAGAA GCTTCTGACT ACGAAAATAA
TW183 ATCTTATTTT CTTCAGCCTA TATCCCAGAA GCTTCTGACT ACGAAAATAA
TOR1 ATCTTATTTT CTTCAGCCTA TATCCCAGAA GCTTCTGACT ACGAAAATAA
WA97001 ATCTTATTTT CTTCAGCCTA TATCCCAGAA GCTTCTGACT ACGAAAATAA
1979 ATCTTATTTT CTTCAGCCTA TATCCCAGAA GCTTCTGACT ACGAAAATAA
SH511 ATCTTATTTT CTTCAGCCTA TATCCCAGAA GCTTCTGACT ACGAAAATAA
1,260 1,270 1,280 1,290 1,300
Identity
B26 TTTCATTAGC AGCTCGAAAA ATACCTCTGA ACTTCGCAAT GGTGTCCTCT
LPCoLN TTTCATTAGC AGCTCGAAAA ATACCTCTGA ACTTCGCAAT GGTGTCCTCT
DE177 TTTCATTAGC AGCTCGAAAA ATACCTCTGA ACTTCGCAAT GGTGTCCTCT
AR39 TTTCATTAGC AGCTCGAAAA ATACCTCTGA ACTTCGCAAT GGTGTCCTCT
CWL029 TTTCATTAGC AGCTCGAAAA ATACCTCTGA ACTTCGCAAT GGTGTCCTCT
J138 TTTCATTAGC AGCTCGAAAA ATACCTCTGA ACTTCGCAAT GGTGTCCTCT
TW183 TTTCATTAGC AGCTCGAAAA ATACCTCTGA ACTTCGCAAT GGTGTCCTCT
TOR1 TTTCATTAGC AGCTCGAAAA ATACCTCTGA ACTTCGCAAT GGTGTCCTCT
WA97001 TTTCATTAGC AGCTCGAAAA ATACCTCTGA ACTTCGCAAT GGTGTCCTCT
1979 TTTCATTAGC AGCTCGAGAA ATACCTCTGA ACTTCGCAAT GGTGTCCTCT
SH511 TTTCATTAGC AGCTCGAGAA ATACCTCTGA ACTTCGCAAT GGTGTCCTCT

	1,310	1,320	1,330	1,340	1,350
Identity					
B26	CTATCGAGGA	TCGTGCGGGA	TGGCAATTCT	ATAAGTTCAC	TCAAAAAGGA
LPCoLN	CTATCGAGGA	TCGTGCGGGA	TGGCAATTCT	ATAAGTTCAC	TCAAAAAGGA
DE177	CTATCGAGGA	TCATGCGGGA	TGGCAATTCT	ATAAGTTCAC	TCAAAAAGGA
AR39	CTATCGAGGA	TCGTGCGGGA	TGGCAATTCT	ATAAGTTCAC	TCAAAAAGGA
CWL029	CTATCGAGGA	TCGTGCGGGA	TGGCAATTCT	ATAAGTTCAC	TCAAAAAGGA
J138	CTATCGAGGA	TCGTGCGGGA	TGGCAATTCT	ATAAGTTCAC	TCAAAAAGGA
TW183	CTATCGAGGA	TCGTGCGGGA	TGGCAATTCT	ATAAGTTCAC	TCAAAAAGGA
TOR1	CTATCGAGGA	TCGTGCGGGA	TGGCAATTCT	ATAAGTTCAC	TCAAAAAGGA
WA97001	CTATCGAGGA	TCGTGCGGGA	TGGCAATTCT	ATAAGTTCAC	TCAAAAAGGA
1979	CTATCGAGGA	TCGTGCGGGA	TGGCAATTCT	ATAAGTTCAC	TCAAAAAGGA
SH511	CTATCGAGGA	TCGTGCGGGA	TGGCAATTCT	ATAAGTTCAC	TCAAAAAGGA

	1,360	1,370	1,380	1,390	1,400
Identity					
B26	GGTATCCTTA	AATTAGGGCA	TGCGGCGAGT	ATTGCAACAA	CTGCCAACTC
LPCoLN	GGTATCCTTA	AATTAGGGCA	TGCGGCGAGT	ATTGCAACAA	CTGCCAACTC
DE177	GGTATCCTTA	AATTAGGGCA	TGCGGCGAGT	ATTGCAACAA	CTGCCAACTC
AR39	GGTATCCTTA	AATTAGGGCA	TGCGGCGAGT	ATTGCAACAA	CTGCCAACTC
CWL029	GGTATCCTTA	AATTAGGGCA	TGCGGCGAGT	ATTGCAACAA	CTGCCAACTC
J138	GGTATCCTTA	AATTAGGGCA	TGCGGCGAGT	ATTGCAACAA	CTGCCAACTC
TW183	GGTATCCTTA	AATTAGGGCA	TGCGGCGAGT	ATTGCAACAA	CTGCCAACTC
TOR1	GGTATCCTTA	AATTAGGGCA	TGCGGCGAGT	ATTGCAACAA	CTGCCAACTC
WA97001	GGTATCCTTA	AATTAGGGCA	TGCGGCGAGT	ATTGCAACAA	CTGCCAACTC
1979	GGTATCCTTA	AATTAGGGCA	TGCGGCGAGT	ATTGCAACAA	CTGCCAACTC
SH511	GGTATCCTTA	AATTAGGGCA	TGCGGCGAGT	ATTGCAACAA	CTGCCAACTC

	1,410	1,420	1,430	1,440	1,450
Identity					
B26	TGAGACTCCA	TCAACTAGTG	TAGGCTCCCA	GGTCATCATT	AATAACCTTG
LPCoLN	TGAGACTCCA	TCAACTAGTG	TAGGCTCCCA	GGTCATCATT	AATAACCTTG
DE177	TGAGACTCCA	TCAACTAGTG	TAGGCTCCCA	GGTCATCATT	AATAACCTTG
AR39	TGAGACTCCA	TCAACTAGTG	TAGGCTCCCA	GGTCATCATT	AATAACCTTG
CWL029	TGAGACTCCA	TCAACTAGTG	TAGGCTCCCA	GGTCATCATT	AATAACCTTG
J138	TGAGACTCCA	TCAACTAGTG	TAGGCTCCCA	GGTCATCATT	AATAACCTTG
TW183	TGAGACTCCA	TCAACTAGTG	TAGGCTCCCA	GGTCATCATT	AATAACCTTG
TOR1	TGAGACTCCA	TCAACTAGTG	TAGGCTCCCA	GGTCATCATT	AATAACCTTG
WA97001	TGAGACTCCA	TCAACTAGTG	TAGGCTCCCA	GGTCATCATT	AATAACCTTG
1979	TGAGACTCCA	TCAACTAGTG	TAGGCTCCCA	GGTCATCATT	AATAACCTTG
SH511	TGAGACTCCA	TCAACTAGTG	TAGGCTCCCA	GGTCATCATT	AATAACCTTG

	1,460	1,470	1,480	1,490	1,500
Identity					
B26	CGATTAACCT	CCCCTCGATC	TTAGCAAAAG	GAAAAGCTCC	TACCTTGTGG
LPCoLN	CGATTAACCT	CCCCTCGATC	TTAGCAAAAG	GAAAAGCTCC	TACCTTGTGG
DE177	CGATTAACCT	CCCCTCGATC	TTAGCAAAAG	GAAAAGCTCC	TACCTTGTGG
AR39	CGATTAACCT	CCCCTCGATC	TTAGCAAAAG	GAAAAGCTCC	TACCTTGTGG
CWL029	CGATTAACCT	CCCCTCGATC	TTAGCAAAAG	GAAAAGCTCC	TACCTTGTGG
J138	CGATTAACCT	CCCCTCGATC	TTAGCAAAAG	GAAAAGCTCC	TACCTTGTGG
TW183	CGATTAACCT	CCCCTCGATC	TTAGCAAAAG	GAAAAGCTCC	TACCTTGTGG
TOR1	CGATTAACCT	CCCCTCGATC	TTAGCAAAAG	GAAAAGCTCC	TACCTTGTGG
WA97001	CGATTAACCT	CCCCTCGATC	TTAGCAAAAG	GAAAAGCTCC	TACCTTGTGG
1979	CGATTAACCT	CCCCTCGATC	TTAGCAAAAG	GAAAAGCTCC	TACCTTGTGG
SH511	CGATTAACCT	CCCCTCGATC	TTAGCAAAAG	GAAAAGCTCC	TACCTTGTGG

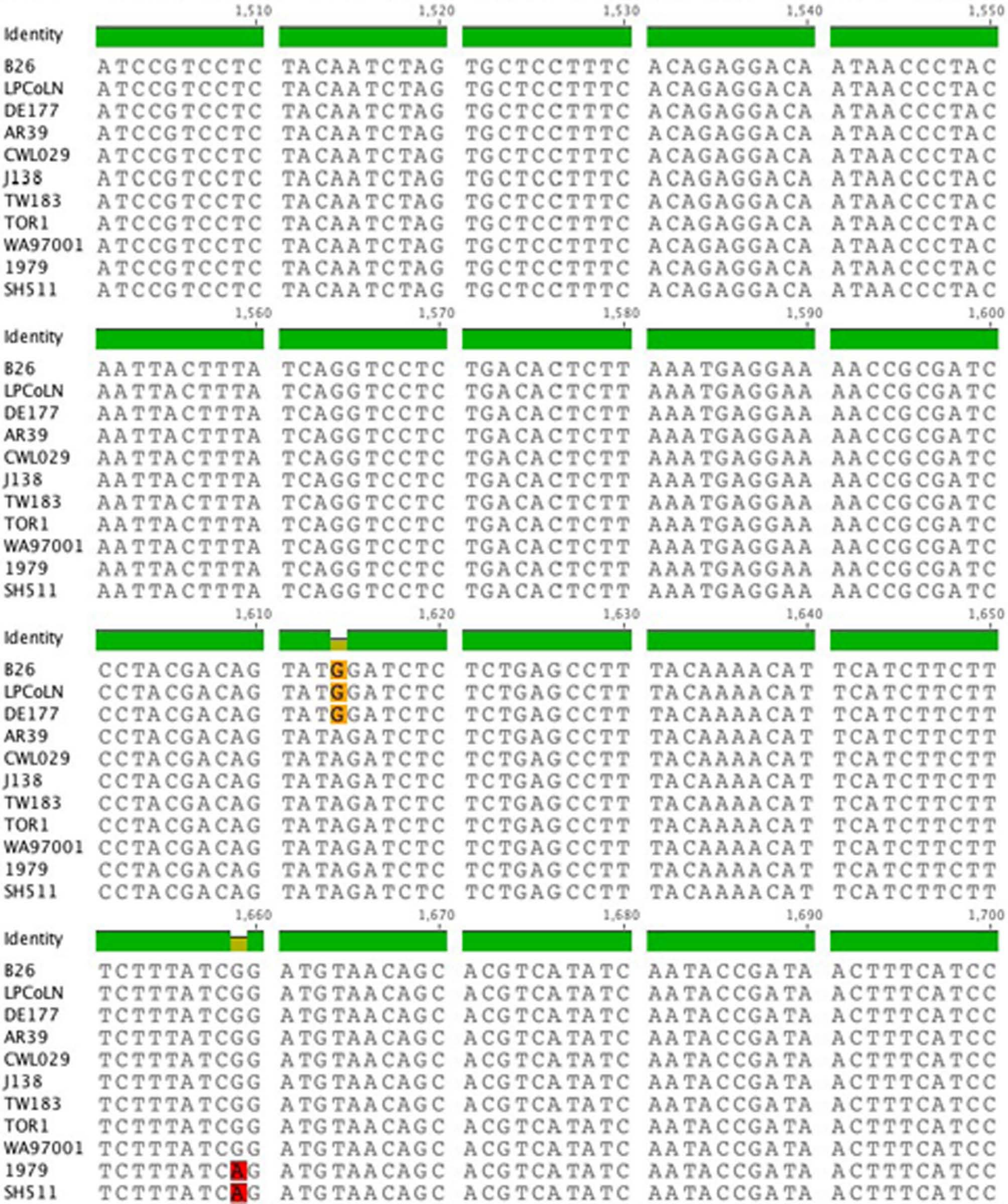
1,510 1,520 1,530 1,540 1,550
Identity
B26 ATCCGTCCTC TACAATCTAG TGCTCCTTTC ACAGAGGACA ATAACCCTAC
LPCoLN ATCCGTCCTC TACAATCTAG TGCTCCTTTC ACAGAGGACA ATAACCCTAC
DE177 ATCCGTCCTC TACAATCTAG TGCTCCTTTC ACAGAGGACA ATAACCCTAC
AR39 ATCCGTCCTC TACAATCTAG TGCTCCTTTC ACAGAGGACA ATAACCCTAC
CWL029 ATCCGTCCTC TACAATCTAG TGCTCCTTTC ACAGAGGACA ATAACCCTAC
J138 ATCCGTCCTC TACAATCTAG TGCTCCTTTC ACAGAGGACA ATAACCCTAC
TW183 ATCCGTCCTC TACAATCTAG TGCTCCTTTC ACAGAGGACA ATAACCCTAC
TOR1 ATCCGTCCTC TACAATCTAG TGCTCCTTTC ACAGAGGACA ATAACCCTAC
WA97001 ATCCGTCCTC TACAATCTAG TGCTCCTTTC ACAGAGGACA ATAACCCTAC
1979 ATCCGTCCTC TACAATCTAG TGCTCCTTTC ACAGAGGACA ATAACCCTAC
SH511 ATCCGTCCTC TACAATCTAG TGCTCCTTTC ACAGAGGACA ATAACCCTAC
1,560 1,570 1,580 1,590 1,600
Identity
B26 AATTACTTTA TCAGGTCCTC TGACACTCTT AAATGAGGAA AACCGCGATC
LPCoLN AATTACTTTA TCAGGTCCTC TGACACTCTT AAATGAGGAA AACCGCGATC
DE177 AATTACTTTA TCAGGTCCTC TGACACTCTT AAATGAGGAA AACCGCGATC
AR39 AATTACTTTA TCAGGTCCTC TGACACTCTT AAATGAGGAA AACCGCGATC
CWL029 AATTACTTTA TCAGGTCCTC TGACACTCTT AAATGAGGAA AACCGCGATC
J138 AATTACTTTA TCAGGTCCTC TGACACTCTT AAATGAGGAA AACCGCGATC
TW183 AATTACTTTA TCAGGTCCTC TGACACTCTT AAATGAGGAA AACCGCGATC
TOR1 AATTACTTTA TCAGGTCCTC TGACACTCTT AAATGAGGAA AACCGCGATC
WA97001 AATTACTTTA TCAGGTCCTC TGACACTCTT AAATGAGGAA AACCGCGATC
1979 AATTACTTTA TCAGGTCCTC TGACACTCTT AAATGAGGAA AACCGCGATC
SH511 AATTACTTTA TCAGGTCCTC TGACACTCTT AAATGAGGAA AACCGCGATC
1,610 1,620 1,630 1,640 1,650
Identity
B26 CCTACGACAG TATGGATCTC TCTGAGCCTT TACAAAACAT TCATCTTCTT
LPCoLN CCTACGACAG TATGGATCTC TCTGAGCCTT TACAAAACAT TCATCTTCTT
DE177 CCTACGACAG TATGGATCTC TCTGAGCCTT TACAAAACAT TCATCTTCTT
AR39 CCTACGACAG TATAGATCTC TCTGAGCCTT TACAAAACAT TCATCTTCTT
CWL029 CCTACGACAG TATAGATCTC TCTGAGCCTT TACAAAACAT TCATCTTCTT
J138 CCTACGACAG TATAGATCTC TCTGAGCCTT TACAAAACAT TCATCTTCTT
TW183 CCTACGACAG TATAGATCTC TCTGAGCCTT TACAAAACAT TCATCTTCTT
TOR1 CCTACGACAG TATAGATCTC TCTGAGCCTT TACAAAACAT TCATCTTCTT
WA97001 CCTACGACAG TATAGATCTC TCTGAGCCTT TACAAAACAT TCATCTTCTT
1979 CCTACGACAG TATAGATCTC TCTGAGCCTT TACAAAACAT TCATCTTCTT
SH511 CCTACGACAG TATAGATCTC TCTGAGCCTT TACAAAACAT TCATCTTCTT
1,660 1,670 1,680 1,690 1,700
Identity
B26 TCTTTATCGG ATGTAACAGC ACGTCATATC AATACCGATA ACTTTCATCC
LPCoLN TCTTTATCGG ATGTAACAGC ACGTCATATC AATACCGATA ACTTTCATCC
DE177 TCTTTATCGG ATGTAACAGC ACGTCATATC AATACCGATA ACTTTCATCC
AR39 TCTTTATCGG ATGTAACAGC ACGTCATATC AATACCGATA ACTTTCATCC
CWL029 TCTTTATCGG ATGTAACAGC ACGTCATATC AATACCGATA ACTTTCATCC
J138 TCTTTATCGG ATGTAACAGC ACGTCATATC AATACCGATA ACTTTCATCC
TW183 TCTTTATCGG ATGTAACAGC ACGTCATATC AATACCGATA ACTTTCATCC
TOR1 TCTTTATCGG ATGTAACAGC ACGTCATATC AATACCGATA ACTTTCATCC
WA97001 TCTTTATCGG ATGTAACAGC ACGTCATATC AATACCGATA ACTTTCATCC
1979 TCTTTATCAG ATGTAACAGC ACGTCATATC AATACCGATA ACTTTCATCC
SH511 TCTTTATCAG ATGTAACAGC ACGTCATATC AATACCGATA ACTTTCATCC

	1,710	1,720	1,730	1,740	1,750
Identity					
B26	TGAAAGCTTA	AATACGACTG	AGCATTACGG	TTATCAAGGC	ATCTGGTCTC
LPCoLN	TGAAAGCTTA	AATACGACTG	AGCATTACGG	TTATCAAGGC	ATCTGGTCTC
DE177	TGAAAGCTTA	AATGCGACTG	AGCATTACGG	TTATCAAGGC	ATCTGGTCTC
AR39	TGAAAGCTTA	AATGCGACTG	AGCATTACGG	TTATCAAGGC	ATCTGGTCTC
CWL029	TGAAAGCTTA	AATGCGACTG	AGCATTACGG	TTATCAAGGC	ATCTGGTCTC
J138	TGAAAGCTTA	AATGCGACTG	AGCATTACGG	TTATCAAGGC	ATCTGGTCTC
TW183	TGAAAGCTTA	AATGCGACTG	AGCATTACGG	TTATCAAGGC	ATCTGGTCTC
TOR1	TGAAAGCTTA	AATGCGACTG	AGCATTACGG	TTATCAAGGC	ATCTGGTCTC
WA97001	TGAAAGCTTA	AATGCGACTG	AGCATTACGG	TTATCAAGGC	ATCTGGTCTC
1979	TGAAAGCTTA	AATGCGACTG	AGCATTACGG	TTATCAAGGC	ATCTGGTCTC
SH511	TGAAAGCTTA	AATGCGACTG	AGCATTACGG	TTATCAAGGC	ATCTGGTCTC

	1,760	1,770	1,780	1,790	1,800
Identity					
B26	CTTATTGGGT	AGAGACGATA	ACAACAACAA	ATAACGCTTC	TATAGAGACG
LPCoLN	CTTATTGGGT	AGAGACGATA	ACAACAACAA	ATAACGCTTC	TATAGAGACG
DE177	CTTATTGGGT	AGAGACGATA	ACAACAACAA	ATAACGCTTC	TATAGAGACG
AR39	CTTATTGGGT	AGAGACGATA	ACAACAACAA	ATAACGCTTC	TATAGAGACG
CWL029	CTTATTGGGT	AGAGACGATA	ACAACAACAA	ATAACGCTTC	TATAGAGACG
J138	CTTATTGGGT	AGAGACGATA	ACAACAACAA	ATAACGCTTC	TATAGAGACG
TW183	CTTATTGGGT	AGAGACGATA	ACAACAACAA	ATAACGCTTC	TATAGAGACG
TOR1	CTTATTGGGT	AGAGACGATA	ACAACAACAA	ATAACGCTTC	TATAGAGACG
WA97001	CTTATTGGGT	AGAGACGATA	ACAACAACAA	ATAACGCTTC	TATAGAGACG
1979	CTTATTGGGT	AGAGGCGATA	ACAACAACAA	ATAACGCTTC	TATAGAGACG
SH511	CTTATTGGGT	AGAGGCGATA	ACAACAACAA	ATAACGCTTC	TATAGAGACG

	1,810	1,820	1,830	1,840	1,850
Identity					
B26	GCAAACACCC	TCTATAGAGC	TCTGTATGCC	AATTGGACTC	CCTTAGGATA
LPCoLN	GCAAACACCC	TCTATAGAGC	TCTGTATGCC	AATTGGACTC	CCTTAGGATA
DE177	GCAAACACCC	TCTATAGAGC	TCTGTATGCC	AATTGGACTC	CCTTAGGATA
AR39	GCAAACACCC	TCTACAGAGC	TCTGTATGCC	AATTGGACTC	CCTTAGGATA
CWL029	GCAAACACCC	TCTACAGAGC	TCTGTATGCC	AATTGGACTC	CCTTAGGATA
J138	GCAAACACCC	TCTACAGAGC	TCTGTATGCC	AATTGGACTC	CCTTAGGATA
TW183	GCAAACACCC	TCTACAGAGC	TCTGTATGCC	AATTGGACTC	CCTTAGGATA
TOR1	GCAAACACCC	TCTACAGAGC	TCTGTATGCC	AATTGGACTC	CCTTAGGATA
WA97001	GCAAACACCC	TCTACAGAGC	TCTGTATGCC	AATTGGACTC	CCTTAGGATA
1979	GCAAACACCC	TCTATAGAGC	TCTGTATGCC	AATTGGACTC	CCTTAGGATA
SH511	GCAAACACCC	TCTATAGAGC	TCTGTATGCC	AATTGGACTC	CCTTAGGATA

	1,860	1,870	1,880	1,890	1,900
Identity					
B26	TAAGGTCAAT	CCTGAATACC	AAGGAGATCT	TGCTACGACT	CCCCTATGGC
LPCoLN	TAAGGTCAAT	CCTGAATACC	AAGGAGATCT	TGCTACGACT	CCCCTATGGC
DE177	TAAGGTCAAT	CCTGAATACC	AAGGAGATCT	TGCTACGACT	CCCCTATGGC
AR39	TAAGGTCAAT	CCTGAATACC	AAGGAGATCT	TGCTACGACT	CCCCTATGGC
CWL029	TAAGGTCAAT	CCTGAATACC	AAGGAGATCT	TGCTACGACT	CCCCTATGGC
J138	TAAGGTCAAT	CCTGAATACC	AAGGAGATCT	TGCTACGACT	CCCCTATGGC
TW183	TAAGGTCAAT	CCTGAATACC	AAGGAGATCT	TGCTACGACT	CCCCTATGGC
TOR1	TAAGGTCAAT	CCTGAATACC	AAGGAGATCT	TGCTACGACT	CCCCTATGGC
WA97001	TAAGGTCAAT	CCTGAATACC	AAGGAGATCT	TGCTACGACT	CCCCTATGGC
1979	TAAGGTCAAT	CCTGAATACC	AAGGAAATCT	TGCTACGACT	CCCCTATGGC
SH511	TAAGGTCAAT	CCTGAATACC	AAGGAAATCT	TGCTACGACT	CCCCTATGGC

	1,910	1,920	1,930	1,940	1,950
Identity					
B26	AATCCTTTCA	TACTATGTTC	TCTCTATTAA	GAAGTTATAA	TCGAACTGGT
LPCoLN	AATCCTTTCA	TACTATGTTC	TCTCTATTAA	GAAGTTATAA	TCGAACTGGT
DE177	AATCCTTTCA	TACTATGTTC	TCTCTATTAA	GAAGTTATAA	TCGAACTGGT
AR39	AATCCTTTCA	TACTATGTTC	TCTCTATTAA	GAAGTTATAA	TCGAACTGGT
CWL029	AATCCTTTCA	TACTATGTTC	TCTCTATTAA	GAAGTTATAA	TCGAACTGGT
J138	AATCCTTTCA	TACTATGTTC	TCTCTATTAA	GAAGTTATAA	TCGAACTGGT
TW183	AATCCTTTCA	TACTATGTTC	TCTCTATTAA	GAAGTTATAA	TCGAACTGGT
TOR1	AATCCTTTCA	TACTATGTTC	TCTCTATTAA	GAAGTTATAA	TCGAACTGGT
WA97001	AATCCTTTCA	TACTATGTTC	TCTCTATTAA	GAAGTTATAA	TCGAACTGGT
1979	AATCCTTTCA	TACTATGTTC	TCTCTATTAA	GAAGTTATAA	TCGAACTGGT
SH511	AATCCTTTCA	TACTATGTTC	TCTCTATTAA	GAAGTTATAA	TCGAACTGGT

	1,960	1,970	1,980	1,990	2,000
Identity					
B26	GATTCTGATA	TCGAGAGGCC	TTTCTTAGAA	ATTCAAGGAA	TTGCCGACGG
LPCoLN	GATTCTGATA	TCGAGAGGCC	TTTCTTAGAA	ATTCAAGGAA	TTGCCGACGG
DE177	GATTCTGATA	TCGAGAGGCC	TTTCTTAGAA	ATTCAAGGGA	TTGCCGACGG
AR39	GATTCTGATA	TCGAGAGGCC	TTTCTTAGAA	ATTCAAGGGA	TTGCCGACGG
CWL029	GATTCTGATA	TCGAGAGGCC	TTTCTTAGAA	ATTCAAGGGA	TTGCCGACGG
J138	GATTCTGATA	TCGAGAGGCC	TTTCTTAGAA	ATTCAAGGGA	TTGCCGACGG
TW183	GATTCTGATA	TCGAGAGGCC	TTTCTTAGAA	ATTCAAGGGA	TTGCCGACGG
TOR1	GATTCTGATA	TCGAGAGGCC	TTTCTTAGAA	ATTCAAGGGA	TTGCCGACGG
WA97001	GATTCTGATA	TCGAGAGGCC	TTTCTTAGAA	ATTCAAGGGA	TTGCCGACGG
1979	GATTCTGATA	TCGAGAGGCC	TTTCTTAGAA	ATTCAAGGGA	TTGCCGACGG
SH511	GATTCTGATA	TCGAGAGGCC	TTTCTTAGAA	ATTCAAGGGA	TTGCCGACGG

	2,010	2,020	2,030	2,040	2,050
Identity					
B26	CCTCTTTGTT	CATCAAAATA	GCATCCCCGG	GACTCCAGGA	TTCCGTATCC
LPCoLN	CCTCTTTGTT	CATCAAAATA	GCATCCCCGG	GACTCCAGGA	TTCCGTATCC
DE177	CCTCTTTGTT	CATCAAAATA	GCATCCCCGG	GGCTCCAGGA	TTCCGTATCC
AR39	CCTCTTTGTT	CATCAAAATA	GCATCCCCGG	GGCTCCAGGA	TTCCGTATCC
CWL029	CCTCTTTGTT	CATCAAAATA	GCATCCCCGG	GGCTCCAGGA	TTCCGTATCC
J138	CCTCTTTGTT	CATCAAAATA	GCATCCCCGG	GGCTCCAGGA	TTCCGTATCC
TW183	CCTCTTTGTT	CATCAAAATA	GCATCCCCGG	GGCTCCAGGA	TTCCGTATCC
TOR1	CCTCTTTGTT	CATCAAAATA	GCATCCCCGG	GGCTCCAGGA	TTCCGTATCC
WA97001	CCTCTTTGTT	CATCAAAATA	GCATCCCCGG	GGCTCCAGGA	TTCCGTATCC
1979	CCTCTTTGTT	CATCAAAATA	GCATCCCCGG	GGCTCCAGGA	TTCCGTATCC
SH511	CCTCTTTGTT	CATCAAAATA	GCATCCCCGG	GGCTCCAGGA	TTCCGTATCC

	2,060	2,070	2,080	2,090	2,100
Identity					
B26	AATCTACAGG	GTATTCCTTA	CAAGCATCCT	CCGAAACTTC	TTTGCATCAG
LPCoLN	AATCTACAGG	GTATTCCTTA	CAAGCATCCT	CCGAAACTTC	TTTGCATCAG
DE177	AATCTACAGG	GTATTCCTTA	CAAGCATCCT	CCGAAACTTC	TTTACATCAG
AR39	AATCTACAGG	GTATTCCTTA	CAAGCATCCT	CCGAAACTTC	TTTACATCAG
CWL029	AATCTACAGG	GTATTCCTTA	CAAGCATCCT	CCGAAACTTC	TTTACATCAG
J138	AATCTACAGG	GTATTCCTTA	CAAGCATCCT	CCGAAACTTC	TTTACATCAG
TW183	AATCTACAGG	GTATTCCTTA	CAAGCATCCT	CCGAAACTTC	TTTACATCAG
TOR1	AATCTACAGG	GTATTCCTTA	CAAGCATCCT	CCGAAACTTC	TTTACATCAG
WA97001	AATCTACAGG	GTATTCCTTA	CAAGCATCCT	CCGAAACTTC	TTTACATCAG
1979	AATCTACAGG	GTATTCCTTA	CAAGCATCCT	CCGAAACTTC	TTTACATCAG
SH511	AATCTACAGG	GTATTCCTTA	CAAGCATCCT	CCGAAACTTC	TTTACATCAG

	2,110	2,120	2,130	2,140	2,150
Identity					
B26	AAAATCTCCT	TAGGTTTTGC	ACAGTTCTTC	ACCCGCACTA	AAGAAATCGG
LPCoLN	AAAATCTCCT	TAGGTTTTGC	ACAGTTCTTC	ACCCGCACTA	AAGAAATCGG
DE177	AAAATCTCCT	TAGGTTTTGC	ACAGTTCTTC	ACCCGCACTA	AAGAAATCGG
AR39	AAAATCTCCT	TAGGTTTTGC	ACAGTTCTTC	ACCCGCACTA	AAGAAATCGG
CWL029	AAAATCTCCT	TAGGTTTTGC	ACAGTTCTTC	ACCCGCACTA	AAGAAATCGG
J138	AAAATCTCCT	TAGGTTTTGC	ACAGTTCTTC	ACCCGCACTA	AAGAAATCGG
TW183	AAAATCTCCT	TAGGTTTTGC	ACAGTTCTTC	ACCCGCACTA	AAGAAATCGG
TOR1	AAAATCTCCT	TAGGTTTTGC	ACAGTTCTTC	ACCCGCACTA	AAGAAATCGG
WA97001	AAAATCTCCT	TAGGTTTTGC	ACAGTTCTTC	ACCCGCACTA	AAGAAATCGG
1979	AAAATCTCCT	TAGGTTTTGC	ACAGTTCTTC	ACCCGCACTA	AAGAAATCGG
SH511	AAAATCTCCT	TAGGTTTTGC	ACAGTTCTTC	ACCCGCACTA	AAGAAATCGG

	2,160	2,169
Identity		
B26	ATCAAGCAAC	AACGTCTCG
LPCoLN	ATCAAGCAAC	AACGTCTCG
DE177	ATCAAGCAAC	AACGTCTCG
AR39	ATCAAGCAAC	AACGTCTCG
CWL029	ATCAAGCAAC	AACGTCTCG
J138	ATCAAGCAAC	AACGTCTCG
TW183	ATCAAGCAAC	AACGTCTCG
TOR1	ATCAAGCAAC	AACGTCTCG
WA97001	ATCAAGCAAC	AACGTCTCG
1979	ATCAAGCAAC	AACGTCTCG
SH511	ATCAAGCAAC	AACGTCTCG

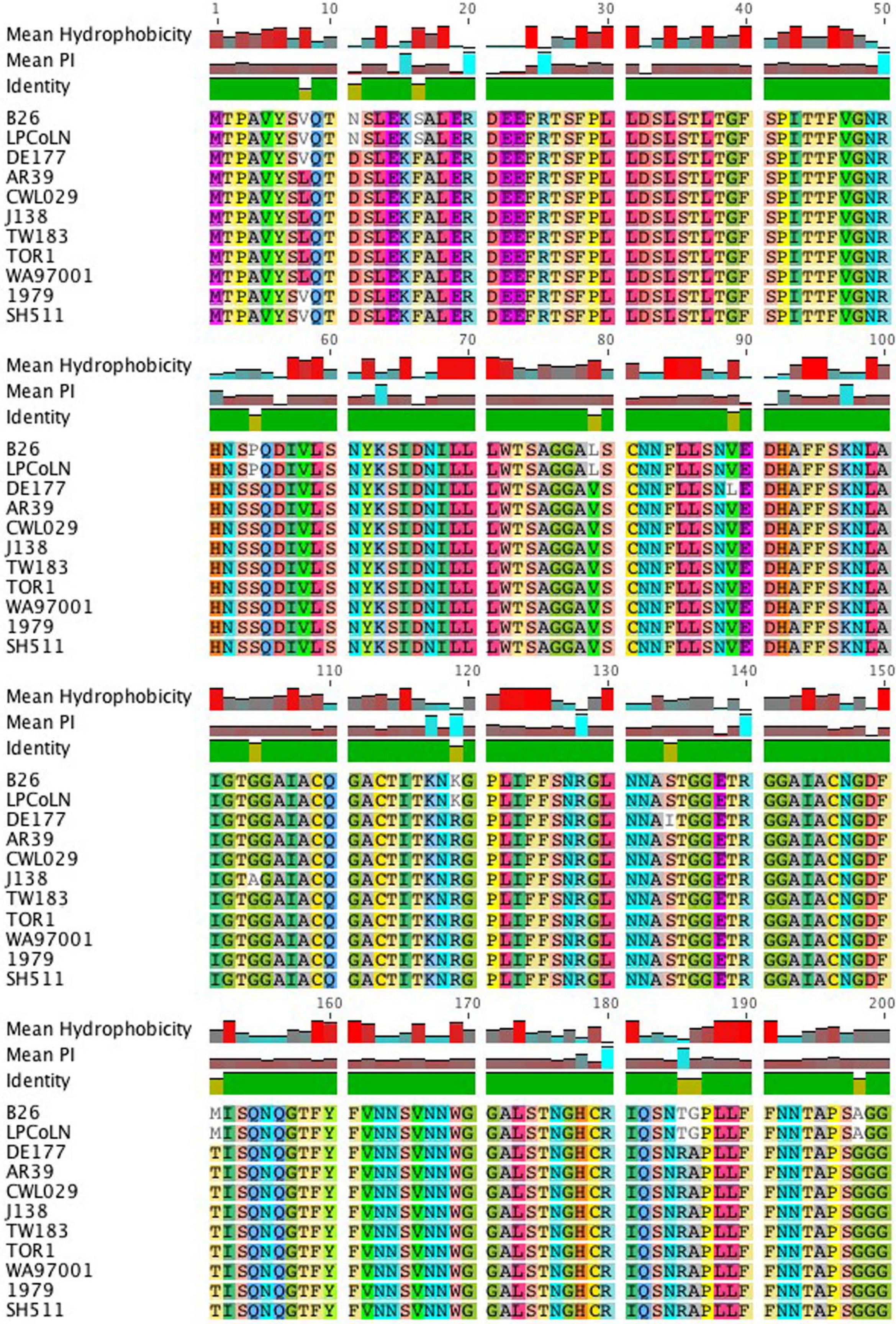

1 10 20 30 40 50
Mean Hydrophobicity
Mean PI
Identity
B26 MTPAVYSVQT NSLEKSALER DEEFRTSFPL LDSLSTLTGF SPITTFVGNR
LPCoLN MTPAVYSVQT NSLEKSALER DEEFRTSFPL LDSLSTLTGF SPITTFVGNR
DE177 MTPAVYSVQT DSLEKFALER DEEFRTSFPL LDSLSTLTGF SPITTFVGNR
AR39 MTPAVYSLQT DSLEKFALER DEEFRTSFPL LDSLSTLTGF SPITTFVGNR
CWL029 MTPAVYSLQT DSLEKFALER DEEFRTSFPL LDSLSTLTGF SPITTFVGNR
J138 MTPAVYSLQT DSLEKFALER DEEFRTSFPL LDSLSTLTGF SPITTFVGNR
TW183 MTPAVYSLQT DSLEKFALER DEEFRTSFPL LDSLSTLTGF SPITTFVGNR
TOR1 MTPAVYSLQT DSLEKFALER DEEFRTSFPL LDSLSTLTGF SPITTFVGNR
WA97001 MTPAVYSLQT DSLEKFALER DEEFRTSFPL LDSLSTLTGF SPITTFVGNR
1979 MTPAVYSVQT DSLEKFALER DEEFRTSFPL LDSLSTLTGF SPITTFVGNR
SH511 MTPAVYSVQT DSLEKFALER DEEFRTSFPL LDSLSTLTGF SPITTFVGNR
60 70 80 90 100
Mean Hydrophobicity
Mean PI
Identity
B26 HNSPQDIVLS NYKSIDNILL LWTSAGGALS CNNFLLSNVE DHAFFSKNLA
LPCoLN HNSPQDIVLS NYKSIDNILL LWTSAGGALS CNNFLLSNVE DHAFFSKNLA
DE177 HNSSQDIVLS NYKSIDNILL LWTSAGGAVS CNNFLLSNLE DHAFFSKNLA
AR39 HNSSQDIVLS NYKSIDNILL LWTSAGGAVS CNNFLLSNVE DHAFFSKNLA
CWL029 HNSSQDIVLS NYKSIDNILL LWTSAGGAVS CNNFLLSNVE DHAFFSKNLA
J138 HNSSQDIVLS NYKSIDNILL LWTSAGGAVS CNNFLLSNVE DHAFFSKNLA
TW183 HNSSQDIVLS NYKSIDNILL LWTSAGGAVS CNNFLLSNVE DHAFFSKNLA
TOR1 HNSSQDIVLS NYKSIDNILL LWTSAGGAVS CNNFLLSNVE DHAFFSKNLA
WA97001 HNSSQDIVLS NYKSIDNILL LWTSAGGAVS CNNFLLSNVE DHAFFSKNLA
1979 HNSSQDIVLS NYKSIDNILL LWTSAGGAVS CNNFLLSNVE DHAFFSKNLA
SH511 HNSSQDIVLS NYKSIDNILL LWTSAGGAVS CNNFLLSNVE DHAFFSKNLA
110 120 130 140 150
Mean Hydrophobicity
Mean PI
Identity
B26 IGTGGAIACQ GACTITKNKG PLIFFSNRGL NNASTGGETR GGAIACNGDF
LPCoLN IGTGGAIACQ GACTITKNKG PLIFFSNRGL NNASTGGETR GGAIACNGDF
DE177 IGTGGAIACQ GACTITKNRG PLIFFSNRGL NNAITGGETR GGAIACNGDF
AR39 IGTGGAIACQ GACTITKNRG PLIFFSNRGL NNASTGGETR GGAIACNGDF
CWL029 IGTGGAIACQ GACTITKNRG PLIFFSNRGL NNASTGGETR GGAIACNGDF
J138 IGTAGAIACQ GACTITKNRG PLIFFSNRGL NNASTGGETR GGAIACNGDF
TW183 IGTGGAIACQ GACTITKNRG PLIFFSNRGL NNASTGGETR GGAIACNGDF
TOR1 IGTGGAIACQ GACTITKNRG PLIFFSNRGL NNASTGGETR GGAIACNGDF
WA97001 IGTGGAIACQ GACTITKNRG PLIFFSNRGL NNASTGGETR GGAIACNGDF
1979 IGTGGAIACQ GACTITKNRG PLIFFSNRGL NNASTGGETR GGAIACNGDF
SH511 IGTGGAIACQ GACTITKNRG PLIFFSNRGL NNASTGGETR GGAIACNGDF
160 170 180 190 200
Mean Hydrophobicity
Mean PI
Identity
B26 MISQNQGTFY FVNNSVNNWG GALSTNGHCR IQSNTGPLLF FNNTAPSAGG
LPCoLN MISQNQGTFY FVNNSVNNWG GALSTNGHCR IQSNTGPLLF FNNTAPSAGG
DE177 TISQNQGTFY FVNNSVNNWG GALSTNGHCR IQSNRAPLLF FNNTAPSGGG
AR39 TISQNQGTFY FVNNSVNNWG GALSTNGHCR IQSNRAPLLF FNNTAPSGGG
CWL029 TISQNQGTFY FVNNSVNNWG GALSTNGHCR IQSNRAPLLF FNNTAPSGGG
J138 TISQNQGTFY FVNNSVNNWG GALSTNGHCR IQSNRAPLLF FNNTAPSGGG
TW183 TISQNQGTFY FVNNSVNNWG GALSTNGHCR IQSNRAPLLF FNNTAPSGGG
TOR1 TISQNQGTFY FVNNSVNNWG GALSTNGHCR IQSNRAPLLF FNNTAPSGGG
WA97001 TISQNQGTFY FVNNSVNNWG GALSTNGHCR IQSNRAPLLF FNNTAPSGGG
1979 TISQNQGTFY FVNNSVNNWG GALSTNGHCR IQSNRAPLLF FNNTAPSGGG
SH511 TISQNQGTFY FVNNSVNNWG GALSTNGHCR IQSNRAPLLF FNNTAPSGGG

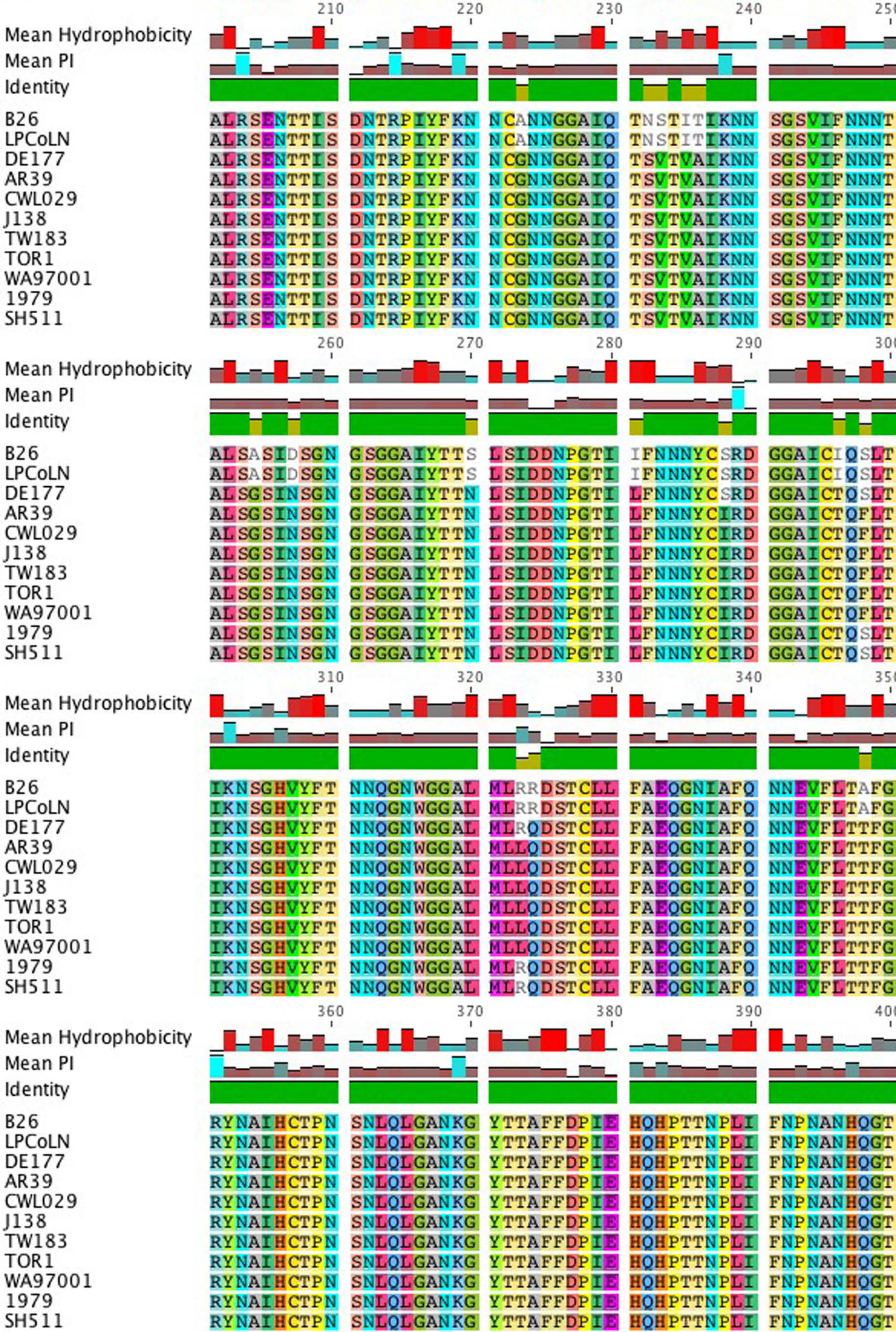

210 220 230 240
Mean Hydrophobicity
Mean PI
Identity
B26 ALRSENTTIS DNTRPIYFKN NCANNGGAIQ TNSTITIKNN SGSVIFNNNT
LPCoLN ALRSENTTIS DNTRPIYFKN NCANNGGAIQ TNSTITIKNN SGSVIFNNNT
DE177 ALRSENTTIS DNTRPIYFKN NCGNNGGAIQ TSVTVAIKNN SGSVIFNNNT
AR39 ALRSENTTIS DNTRPIYFKN NCGNNGGAIQ TSVTVAIKNN SGSVIFNNNT
CWL029 ALRSENTTIS DNTRPIYFKN NCGNNGGAIQ TSVTVAIKNN SGSVIFNNNT
J138 ALRSENTTIS DNTRPIYFKN NCGNNGGAIQ TSVTVAIKNN SGSVIFNNNT
TW183 ALRSENTTIS DNTRPIYFKN NCGNNGGAIQ TSVTVAIKNN SGSVIFNNNT
TOR1 ALRSENTTIS DNTRPIYFKN NCGNNGGAIQ TSVTVAIKNN SGSVIFNNNT
WA97001 ALRSENTTIS DNTRPIYFKN NCGNNGGAIQ TSVTVAIKNN SGSVIFNNNT
1979 ALRSENTTIS DNTRPIYFKN NCGNNGGAIQ TSVTVAIKNN SGSVIFNNNT
SH511 ALRSENTTIS DNTRPIYFKN NCGNNGGAIQ TSVTVAIKNN SGSVIFNNNT
260 270 280 290
Mean Hydrophobicity
Mean PI
Identity
B26 ALSASIDSGN GSGGAIYTTS LSIDDNPGTI IFNNNYCSRD GGAICIQSLT
LPCoLN ALSASIDSGN GSGGAIYTTS LSIDDNPGTI IFNNNYCSRD GGAICIQSLT
DE177 ALSGSINSGN GSGGAIYTTN LSIDDNPGTI LFNNNYCSRD GGAICTQSLT
AR39 ALSGSINSGN GSGGAIYTTN LSIDDNPGTI LFNNNYCIRD GGAICTQFLT
CWL029 ALSGSINSGN GSGGAIYTTN LSIDDNPGTI LFNNNYCIRD GGAICTQFLT
J138 ALSGSINSGN GSGGAIYTTN LSIDDNPGTI LFNNNYCIRD GGAICTQFLT
TW183 ALSGSINSGN GSGGAIYTTN LSIDDNPGTI LFNNNYCIRD GGAICTQFLT
TOR1 ALSGSINSGN GSGGAIYTTN LSIDDNPGTI LFNNNYCIRD GGAICTQFLT
WA97001 ALSGSINSGN GSGGAIYTTN LSIDDNPGTI LFNNNYCIRD GGAICTQFLT
1979 ALSGSINSGN GSGGAIYTTN LSIDDNPGTI LFNNNYCIRD GGAICTQSLT
SH511 ALSGSINSGN GSGGAIYTTN LSIDDNPGTI LFNNNYCIRD GGAICTQSLT
310 320 330 340
Mean Hydrophobicity
Mean PI
Identity
B26 IKNSGHVYFT NNQGNWGGAL MLRRDSTCLL FAEQGNIAFQ NNEVFLTAFG
LPCoLN IKNSGHVYFT NNQGNWGGAL MLRRDSTCLL FAEQGNIAFQ NNEVFLTAFG
DE177 IKNSGHVYFT NNQGNWGGAL MLRQDSTCLL FAEQGNIAFQ NNEVFLTTFG
AR39 IKNSGHVYFT NNQGNWGGAL MLLQDSTCLL FAEQGNIAFQ NNEVFLTTFG
CWL029 IKNSGHVYFT NNQGNWGGAL MLLQDSTCLL FAEQGNIAFQ NNEVFLTTFG
J138 IKNSGHVYFT NNQGNWGGAL MLLQDSTCLL FAEQGNIAFQ NNEVFLTTFG
TW183 IKNSGHVYFT NNQGNWGGAL MLLQDSTCLL FAEQGNIAFQ NNEVFLTTFG
TOR1 IKNSGHVYFT NNQGNWGGAL MLLQDSTCLL FAEQGNIAFQ NNEVFLTTFG
WA97001 IKNSGHVYFT NNQGNWGGAL MLLQDSTCLL FAEQGNIAFQ NNEVFLTTFG
1979 IKNSGHVYFT NNQGNWGGAL MLRQDSTCLL FAEQGNIAFQ NNEVFLTTFG
SH511 IKNSGHVYFT NNQGNWGGAL MLRQDSTCLL FAEQGNIAFQ NNEVFLTTFG
360 370 380 390
Mean Hydrophobicity
Mean PI
Identity
B26 RYNAIHCTPN SNLQLGANKG YTTAFFDPIE HQHPTTNPLI FNPNANHQGT
LPCoLN RYNAIHCTPN SNLQLGANKG YTTAFFDPIE HQHPTTNPLI FNPNANHQGT
DE177 RYNAIHCTPN SNLQLGANKG YTTAFFDPIE HQHPTTNPLI FNPNANHQGT
AR39 RYNAIHCTPN SNLQLGANKG YTTAFFDPIE HQHPTTNPLI FNPNANHQGT
CWL029 RYNAIHCTPN SNLQLGANKG YTTAFFDPIE HQHPTTNPLI FNPNANHQGT
J138 RYNAIHCTPN SNLQLGANKG YTTAFFDPIE HQHPTTNPLI FNPNANHQGT
TW183 RYNAIHCTPN SNLQLGANKG YTTAFFDPIE HQHPTTNPLI FNPNANHQGT
TOR1 RYNAIHCTPN SNLQLGANKG YTTAFFDPIE HQHPTTNPLI FNPNANHQGT
WA97001 RYNAIHCTPN SNLQLGANKG YTTAFFDPIE HQHPTTNPLI FNPNANHQGT
1979 RYNAIHCTPN SNLQLGANKG YTTAFFDPIE HQHPTTNPLI FNPNANHQGT
SH511 RYNAIHCTPN SNLQLGANKG YTTAFFDPIE HQHPTTNPLI FNPNANHQGT

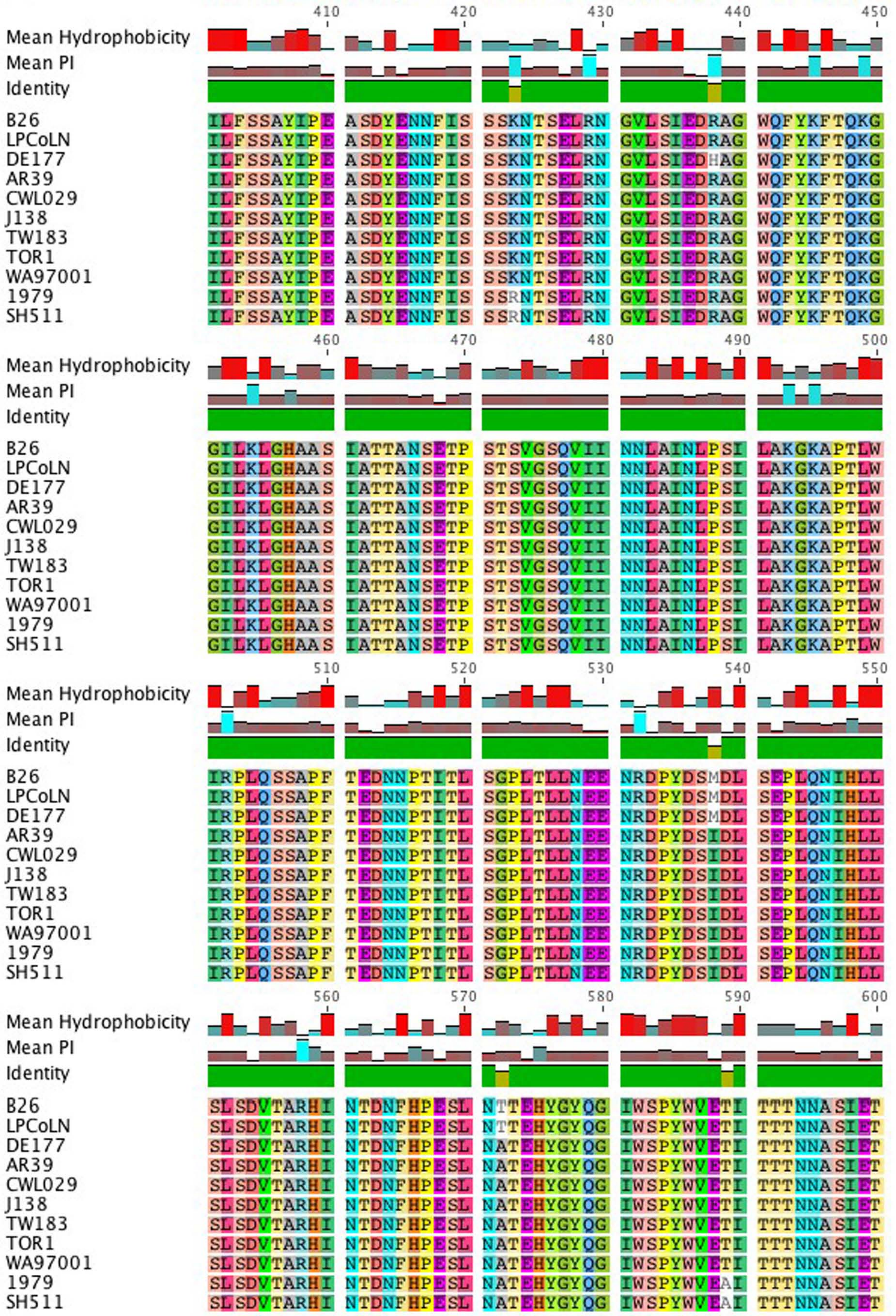
410 420 430 440 450
Mean Hydrophobicity
Mean PI
Identity
B26 ILFSSAYIPE ASDYENNFIS SSKNTSELRN GVLSIEDRAG WQFYKFTQKG
LPCoLN ILFSSAYIPE ASDYENNFIS SSKNTSELRN GVLSIEDRAG WQFYKFTQKG
DE177 ILFSSAYIPE ASDYENNFIS SSKNTSELRN GVLSIEDHAG WQFYKFTQKG
AR39 ILFSSAYIPE ASDYENNFIS SSKNTSELRN GVLSIEDRAG WQFYKFTQKG
CWL029 ILFSSAYIPE ASDYENNFIS SSKNTSELRN GVLSIEDRAG WQFYKFTQKG
J138 ILFSSAYIPE ASDYENNFIS SSKNTSELRN GVLSIEDRAG WQFYKFTQKG
TW183 ILFSSAYIPE ASDYENNFIS SSKNTSELRN GVLSIEDRAG WQFYKFTQKG
TOR1 ILFSSAYIPE ASDYENNFIS SSKNTSELRN GVLSIEDRAG WQFYKFTQKG
WA97001 ILFSSAYIPE ASDYENNFIS SSKNTSELRN GVLSIEDRAG WQFYKFTQKG
1979 ILFSSAYIPE ASDYENNFIS SSRNTSELRN GVLSIEDRAG WQFYKFTQKG
SH511 ILFSSAYIPE ASDYENNFIS SSRNTSELRN GVLSIEDRAG WQFYKFTQKG
460 470 480 490 500
Mean Hydrophobicity
Mean PI
Identity
B26 GILKLGHAAS IATTANSETP STSVGSQVII NNLAINLPSI LAKGKAPTLW
LPCoLN GILKLGHAAS IATTANSETP STSVGSQVII NNLAINLPSI LAKGKAPTLW
DE177 GILKLGHAAS IATTANSETP STSVGSQVII NNLAINLPSI LAKGKAPTLW
AR39 GILKLGHAAS IATTANSETP STSVGSQVII NNLAINLPSI LAKGKAPTLW
CWL029 GILKLGHAAS IATTANSETP STSVGSQVII NNLAINLPSI LAKGKAPTLW
J138 GILKLGHAAS IATTANSETP STSVGSQVII NNLAINLPSI LAKGKAPTLW
TW183 GILKLGHAAS IATTANSETP STSVGSQVII NNLAINLPSI LAKGKAPTLW
TOR1 GILKLGHAAS IATTANSETP STSVGSQVII NNLAINLPSI LAKGKAPTLW
WA97001 GILKLGHAAS IATTANSETP STSVGSQVII NNLAINLPSI LAKGKAPTLW
1979 GILKLGHAAS IATTANSETP STSVGSQVII NNLAINLPSI LAKGKAPTLW
SH511 GILKLGHAAS IATTANSETP STSVGSQVII NNLAINLPSI LAKGKAPTLW
510 520 530 540 550
Mean Hydrophobicity
Mean PI
Identity
B26 IRPLQSSAPF TEDNNPTITL SGPLTLLNEE NRDPYDSMDL SEPLQNIHLL
LPCoLN IRPLQSSAPF TEDNNPTITL SGPLTLLNEE NRDPYDSMDL SEPLQNIHLL
DE177 IRPLQSSAPF TEDNNPTITL SGPLTLLNEE NRDPYDSMDL SEPLQNIHLL
AR39 IRPLQSSAPF TEDNNPTITL SGPLTLLNEE NRDPYDSIDL SEPLQNIHLL
CWL029 IRPLQSSAPF TEDNNPTITL SGPLTLLNEE NRDPYDSIDL SEPLQNIHLL
J138 IRPLQSSAPF TEDNNPTITL SGPLTLLNEE NRDPYDSIDL SEPLQNIHLL
TW183 IRPLQSSAPF TEDNNPTITL SGPLTLLNEE NRDPYDSIDL SEPLQNIHLL
TOR1 IRPLQSSAPF TEDNNPTITL SGPLTLLNEE NRDPYDSIDL SEPLQNIHLL
WA97001 IRPLQSSAPF TEDNNPTITL SGPLTLLNEE NRDPYDSIDL SEPLQNIHLL
1979 IRPLQSSAPF TEDNNPTITL SGPLTLLNEE NRDPYDSIDL SEPLQNIHLL
SH511 IRPLQSSAPF TEDNNPTITL SGPLTLLNEE NRDPYDSIDL SEPLQNIHLL
560 570 580 590 600
Mean Hydrophobicity
Mean PI
Identity
B26 SLSDVTARHI NTDNFHPESL NTTEHYGYQG IWSPYWVETI TTTNNASIET
LPCoLN SLSDVTARHI NTDNFHPESL NTTEHYGYQG IWSPYWVETI TTTNNASIET
DE177 SLSDVTARHI NTDNFHPESL NATEHYGYQG IWSPYWVETI TTTNNASIET
AR39 SLSDVTARHI NTDNFHPESL NATEHYGYQG IWSPYWVETI TTTNNASIET
CWL029 SLSDVTARHI NTDNFHPESL NATEHYGYQG IWSPYWVETI TTTNNASIET
J138 SLSDVTARHI NTDNFHPESL NATEHYGYQG IWSPYWVETI TTTNNASIET
TW183 SLSDVTARHI NTDNFHPESL NATEHYGYQG IWSPYWVETI TTTNNASIET
TOR1 SLSDVTARHI NTDNFHPESL NATEHYGYQG IWSPYWVETI TTTNNASIET
WA97001 SLSDVTARHI NTDNFHPESL NATEHYGYQG IWSPYWVETI TTTNNASIET
1979 SLSDVTARHI NTDNFHPESL NATEHYGYQG IWSPYWVEAI TTTNNASIET
SH511 SLSDVTARHI NTDNFHPESL NATEHYGYQG IWSPYWVEAI TTTNNASIET

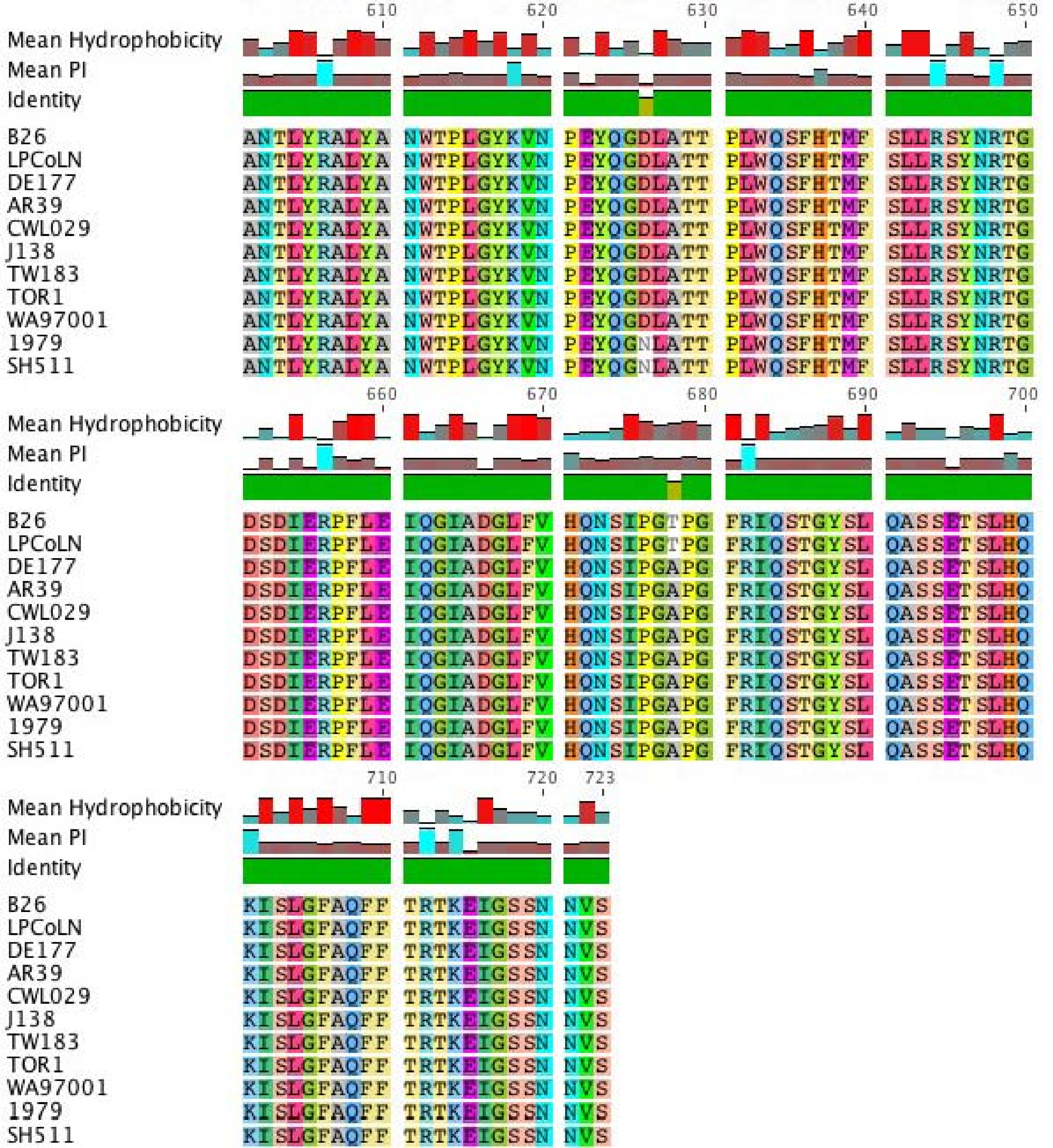

610 620 630 640 650
Mean Hydrophobicity
Mean PI
Identity
B26 ANTLYRALYA NWTPLGYKVN PEYQGDLATT PLWQSFHTMF SLLRSYNRTG
LPCoLN ANTLYRALYA NWTPLGYKVN PEYQGDLATT PLWQSFHTMF SLLRSYNRTG
DE177 ANTLYRALYA NWTPLGYKVN PEYQGDLATT PLWQSFHTMF SLLRSYNRTG
AR39 ANTLYRALYA NWTPLGYKVN PEYQGDLATT PLWQSFHTMF SLLRSYNRTG
CWL029 ANTLYRALYA NWTPLGYKVN PEYQGDLATT PLWQSFHTMF SLLRSYNRTG
J138 ANTLYRALYA NWTPLGYKVN PEYQGDLATT PLWQSFHTMF SLLRSYNRTG
TW183 ANTLYRALYA NWTPLGYKVN PEYQGDLATT PLWQSFHTMF SLLRSYNRTG
TOR1 ANTLYRALYA NWTPLGYKVN PEYQGDLATT PLWQSFHTMF SLLRSYNRTG
WA97001 ANTLYRALYA NWTPLGYKVN PEYQGDLATT PLWQSFHTMF SLLRSYNRTG
1979 ANTLYRALYA NWTPLGYKVN PEYQGNLATT PLWQSFHTMF SLLRSYNRTG
SH511 ANTLYRALYA NWTPLGYKVN PEYQGNLATT PLWQSFHTMF SLLRSYNRTG
660 670 680 690 700
Mean Hydrophobicity
Mean PI
Identity
B26 DSDIERPFLE IQGIADGLFV HQNSIPGTPG FRIQSTGYSL QASSETSLHQ
LPCoLN DSDIERPFLE IQGIADGLFV HQNSIPGTPG FRIQSTGYSL QASSETSLHQ
DE177 DSDIERPFLE IQGIADGLFV HQNSIPGAPG FRIQSTGYSL QASSETSLHQ
AR39 DSDIERPFLE IQGIADGLFV HQNSIPGAPG FRIQSTGYSL QASSETSLHQ
CWL029 DSDIERPFLE IQGIADGLFV HQNSIPGAPG FRIQSTGYSL QASSETSLHQ
J138 DSDIERPFLE IQGIADGLFV HQNSIPGAPG FRIQSTGYSL QASSETSLHQ
TW183 DSDIERPFLE IQGIADGLFV HQNSIPGAPG FRIQSTGYSL QASSETSLHQ
TOR1 DSDIERPFLE IQGIADGLFV HQNSIPGAPG FRIQSTGYSL QASSETSLHQ
WA97001 DSDIERPFLE IQGIADGLFV HQNSIPGAPG FRIQSTGYSL QASSETSLHQ
1979 DSDIERPFLE IQGIADGLFV HQNSIPGAPG FRIQSTGYSL QASSETSLHQ
SH511 DSDIERPFLE IQGIADGLFV HQNSIPGAPG FRIQSTGYSL QASSETSLHQ
710 720 723
Mean Hydrophobicity
Mean PI
Identity
B26 KISLGFAQFF TRTKEIGSSN NVS
LPCoLN KISLGFAQFF TRTKEIGSSN NVS
DE177 KISLGFAQFF TRTKEIGSSN NVS
AR39 KISLGFAQFF TRTKEIGSSN NVS
CWL029 KISLGFAQFF TRTKEIGSSN NVS
J138 KISLGFAQFF TRTKEIGSSN NVS
TW183 KISLGFAQFF TRTKEIGSSN NVS
TOR1 KISLGFAQFF TRTKEIGSSN NVS
WA97001 KISLGFAQFF TRTKEIGSSN NVS
1979 KISLGFAQFF TRTKEIGSSN NVS
SH511 KISLGFAQFF TRTKEIGSSN NVS
