# Supplementary material for: Chlamydia pneumoniae Is Genetically Diverse in Animals and Appears to Have Crossed the Host Barrier to Humans on (At Least) Two Occasions
Source: PLoS Pathog. 2010 May 20;6(5):e1000903. doi: 10.1371/journal.ppat.1000903 (PMC2873915; doi:10.1371/journal.ppat.1000903)
Supplement: Figure S6 — Multiple sequence alignment of pmp E/F3. The nucleotide and amino acid alignments were generated using Geneious version 4.7, where each nucleotide and amino acid is assigned its own colour. White shading indicates an amino acid variant. (1.72 MB PDF) [file ppat.1000903.s006.pdf]

	1–10	11–20	21–30	31–40	41–50
Identity					
LPCoLN	TTATAGAGAT	CAGCAATAAC	TCCGCATCCT	CAGTTAACAC	AGCATCAGGA
DE177	TTATAGAGAT	CAGCAATCAC	TCCGCATCCT	CAATTAACAC	AGCATCAGGA
AR39	TTATAGAGAT	CAGCAATCAC	TCCGCATCCT	CAATTAACAC	AGCATCAGGA
CWL029	TTATAGAGAT	CAGCAATCAC	TCCGCATCCT	CAATTAACAC	AGCATCAGGA
J138	TTATAGAGAT	CAGCAATCAC	TCCGCATCCT	CAATTAACAC	AGCATCAGGA
TW183	TTATAGAGAT	CAGCAATCAC	TCCGCATCCT	CAATTAACAC	AGCATCAGGA
WA97001	TTATAGAGAT	CAGCAATCAC	TCCGCATCCT	CAATTAACAC	AGCATCAGGA
SH511	TTATAGAGAT	CAGCAATCAC	TCCGCATCCT	TAATTAACAC	AGCATCAGGA
TOR1	TTATAGAGAT	CAGCAATCAC	TCCGCATCCT	CAATTAACAC	AGCATCAGGA

	51–60	61–70	71–80	81–90	91–100
Identity					
LPCoLN	AAACTATATC	CCGGTGGTGG	CGGTATTATG	TGTACCTCCT	TAATCGTTGA
DE177	AAACTATATC	CCGGTGGTGG	CGGTATTATG	TGTACCTCCT	TAGTCATTGA
AR39	AAACTATATC	CCGGTGGTGG	CGGTATCATG	TGTACCTCCT	TAGTCATTGA
CWL029	AAACTATATC	CCGGTGGTGG	CGGTATCATG	TGTACCTCCT	TAGTCATTGA
J138	AAACTATATC	CCGGTGGTGG	CGGTATCATG	TGTACCTCCT	TAGTCATTGA
TW183	AAACTATATC	CCGGTGGTGG	CGGTATCATG	TGTACCTCCT	TAGTCATTGA
WA97001	AAACTATATC	CCGGTGGTGG	CGGTATCATG	TGTACCTCCT	TAGTCATTGA
SH511	AAACTATATC	CCGGTGGTGG	CGGTATCATG	TGTACCTCCT	TAGTCATTGA
TOR1	AAACTATATC	CCGGTGGTGG	CGGTATCATG	TGTACCTCCT	TAGTCATTGA

	101–110	111–120	121–130	131–140	141–150
Identity					
LPCoLN	GAACAATCCC	AAAGGTCTTA	TCTTTAACAA	TAACACGGCA	GCACTTAGCG
DE177	GAACAATCCC	AAAGGTCTTA	TCTTTAACAA	TAACACGGCA	GCACTTAGCG
AR39	GAACAATCCC	AAAGGTCTTA	TCTTTAACAA	TAAAACGGCA	GCACTTAGCG
CWL029	GAACAATCCC	AAAGGTCTTA	TCTTTAACAA	TAAAACGGCA	GCACTTAGCG
J138	GAACAATCCC	AAAGGTCTTA	TCTTTAACAA	TAAAACGGCA	GCACTTAGCG
TW183	GAACAATCCC	AAAGGTCTTA	TCTTTAACAA	TAAAACGGCA	GCACTTAGCG
WA97001	GAACAATCCC	AAAGGTCTTA	TCTTTAACAA	TAAAACGGCA	GCACTTAGCG
SH511	GAACAATCCC	AAAGGTCTTA	TCTTTAACAA	TAAAACGGCA	GCACTTAGCG
TOR1	GAACAATCCC	AAAGGTCTTA	TCTTTAACAA	TAAAACGGCA	GCACTTAGCG

	151–160	161–170	171–180	181–190	191–200
Identity					
LPCoLN	GAGGAGCTAT	ACACACGAGA	TCTTTTATTT	TCCAAAATAA	CGGTCCGACA
DE177	GCGGAGCTAT	ACACACGAGA	TCTTTCATCT	TCCAAAATAA	CGGTCCGACA
AR39	GCGGAGCTAT	ACACACGAGA	TCTTTCATCT	TCCAAAATAA	CGGTCCGACA
CWL029	GCGGAGCTAT	ACACACGAGA	TCTTTCATCT	TCCAAAATAA	CGGTCCGACA
J138	GCGGAGCTAT	ACACACGAGA	TCTTTCATCT	TCCAAAATAA	CGGTCCGACA
TW183	GCGGAGCTAT	ACACACGAGA	TCTTTCATCT	TCCAAAATAA	CGGTCCGACA
WA97001	GCGGAGCTAT	ACACACGAGA	TCTTTCATCT	TCCAAAATAA	CGGTCCGACA
SH511	GCGGAGCTAT	ACACACGAGA	TCTTTCATCT	TCCAAAATAA	CGGTCCGACA
TOR1	GCGGAGCTAT	ACACACGAGA	TCTTTCATCT	TCCAAAATAA	CGGTCCGACA

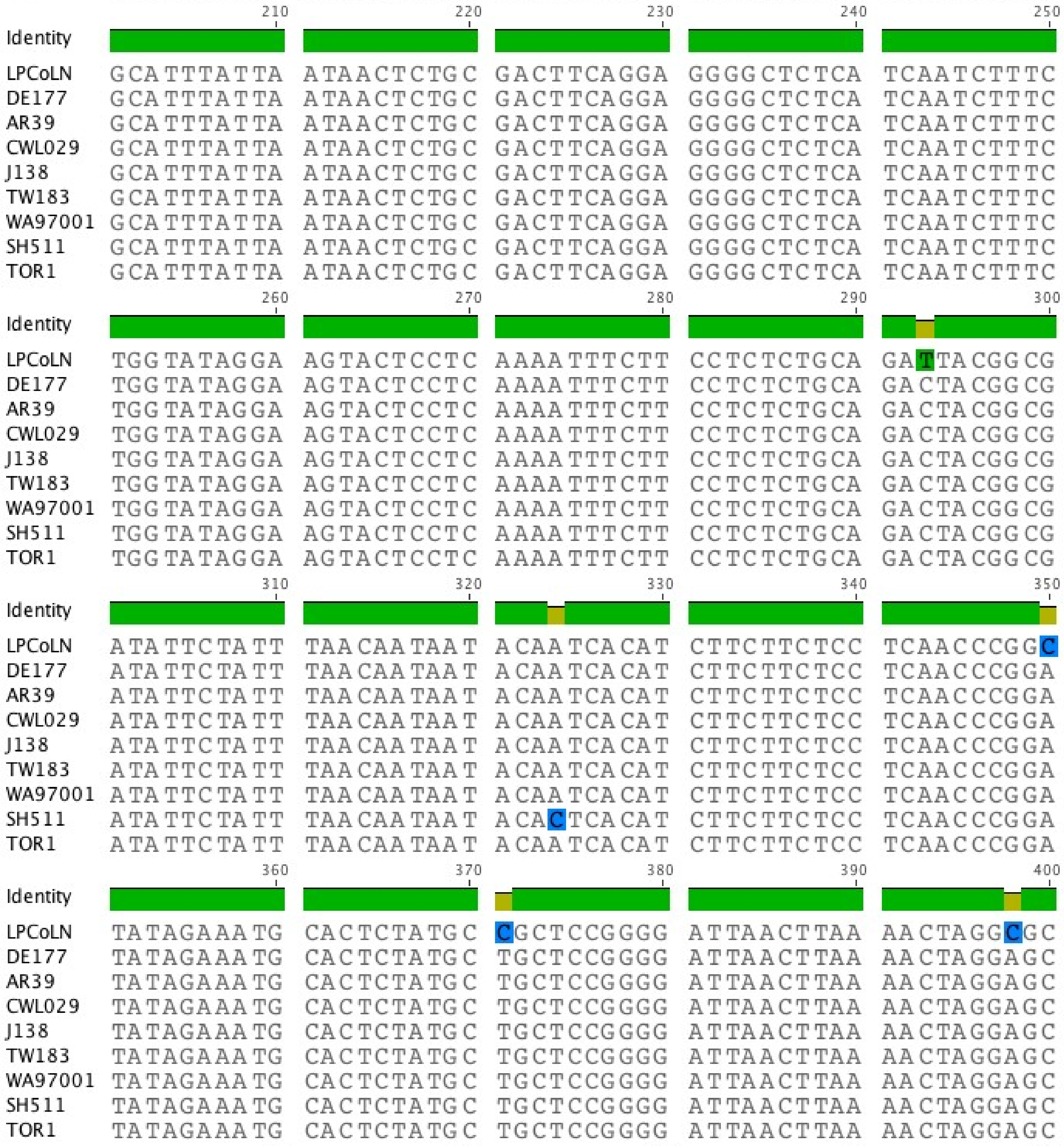

210 220 230 240 250
Identity
LPCoLN GCATTTATTA ATAACTCTGC GACTTCAGGA GGGGCTCTCA TCAATCTTTC
DE177 GCATTTATTA ATAACTCTGC GACTTCAGGA GGGGCTCTCA TCAATCTTTC
AR39 GCATTTATTA ATAACTCTGC GACTTCAGGA GGGGCTCTCA TCAATCTTTC
CWL029 GCATTTATTA ATAACTCTGC GACTTCAGGA GGGGCTCTCA TCAATCTTTC
J138 GCATTTATTA ATAACTCTGC GACTTCAGGA GGGGCTCTCA TCAATCTTTC
TW183 GCATTTATTA ATAACTCTGC GACTTCAGGA GGGGCTCTCA TCAATCTTTC
WA97001 GCATTTATTA ATAACTCTGC GACTTCAGGA GGGGCTCTCA TCAATCTTTC
SH511 GCATTTATTA ATAACTCTGC GACTTCAGGA GGGGCTCTCA TCAATCTTTC
TOR1 GCATTTATTA ATAACTCTGC GACTTCAGGA GGGGCTCTCA TCAATCTTTC
260 270 280 290 300
Identity
LPCoLN TGGTATAGGA AGTACTCCTC AAAATTTCTT CCTCTCTGCA GATTACGGCG
DE177 TGGTATAGGA AGTACTCCTC AAAATTTCTT CCTCTCTGCA GACTACGGCG
AR39 TGGTATAGGA AGTACTCCTC AAAATTTCTT CCTCTCTGCA GACTACGGCG
CWL029 TGGTATAGGA AGTACTCCTC AAAATTTCTT CCTCTCTGCA GACTACGGCG
J138 TGGTATAGGA AGTACTCCTC AAAATTTCTT CCTCTCTGCA GACTACGGCG
TW183 TGGTATAGGA AGTACTCCTC AAAATTTCTT CCTCTCTGCA GACTACGGCG
WA97001 TGGTATAGGA AGTACTCCTC AAAATTTCTT CCTCTCTGCA GACTACGGCG
SH511 TGGTATAGGA AGTACTCCTC AAAATTTCTT CCTCTCTGCA GACTACGGCG
TOR1 TGGTATAGGA AGTACTCCTC AAAATTTCTT CCTCTCTGCA GACTACGGCG
310 320 330 340 350
Identity
LPCoLN ATATTCTATT TAACAATAAT ACAATCACAT CTTCTTCTCC TCAACCCGGC
DE177 ATATTCTATT TAACAATAAT ACAATCACAT CTTCTTCTCC TCAACCCGGA
AR39 ATATTCTATT TAACAATAAT ACAATCACAT CTTCTTCTCC TCAACCCGGA
CWL029 ATATTCTATT TAACAATAAT ACAATCACAT CTTCTTCTCC TCAACCCGGA
J138 ATATTCTATT TAACAATAAT ACAATCACAT CTTCTTCTCC TCAACCCGGA
TW183 ATATTCTATT TAACAATAAT ACAATCACAT CTTCTTCTCC TCAACCCGGA
WA97001 ATATTCTATT TAACAATAAT ACAATCACAT CTTCTTCTCC TCAACCCGGA
SH511 ATATTCTATT TAACAATAAT ACACTCACAT CTTCTTCTCC TCAACCCGGA
TOR1 ATATTCTATT TAACAATAAT ACAATCACAT CTTCTTCTCC TCAACCCGGA
360 370 380 390 400
Identity
LPCoLN TATAGAAATG CACTCTATGC CGCTCCGGGG ATTAACTTAA AACTAGGCGC
DE177 TATAGAAATG CACTCTATGC TGCTCCGGGG ATTAACTTAA AACTAGGAGC
AR39 TATAGAAATG CACTCTATGC TGCTCCGGGG ATTAACTTAA AACTAGGAGC
CWL029 TATAGAAATG CACTCTATGC TGCTCCGGGG ATTAACTTAA AACTAGGAGC
J138 TATAGAAATG CACTCTATGC TGCTCCGGGG ATTAACTTAA AACTAGGAGC
TW183 TATAGAAATG CACTCTATGC TGCTCCGGGG ATTAACTTAA AACTAGGAGC
WA97001 TATAGAAATG CACTCTATGC TGCTCCGGGG ATTAACTTAA AACTAGGAGC
SH511 TATAGAAATG CACTCTATGC TGCTCCGGGG ATTAACTTAA AACTAGGAGC
TOR1 TATAGAAATG CACTCTATGC TGCTCCGGGG ATTAACTTAA AACTAGGAGC

	410	420	430	440	450
LPCoLN	TAGACAGGGT	TATAAAATTC	TCTTTTATGA	TCCTATAGAT	CACGATCAGA
DE177	AAGACAGGGT	TATAAAATTC	TCTTTTATGA	TCCTATAGAT	CACGATCAGA
AR39	AAGACAGGGT	TATAAAATTC	TCTTTTATGA	TCCTATAGAT	CACGATCAGA
CWL029	AAGACAGGGT	TATAAAATTC	TCTTTTATGA	TCCTATAGAT	CACGATCAGA
J138	AAGACAGGGT	TATAAAATTC	TCTTTTATGA	TCCTATAGAT	CACGATCAGA
TW183	AAGACAGGGT	TATAAAATTC	TCTTTTATGA	TCCTATAGAT	CACGATCAGA
WA97001	AAGACAGGGT	TATAAAATTC	TCTTTTATGA	TCCTATAGAT	CACGATCAGA
SH511	AAGACAGGGT	TATAAAATTC	TCTTTTATGA	TCCTATAGAT	CACGATCAGA
TOR1	AAGACAGGGT	TATAAAATTC	TCTTTTATGA	TCCTATAGAT	CACGATCAGA

	460	470	480	490	500
LPCoLN	CGACAACAGA	TCCTATAGTA	TTTAATTATG	AACCCCATCA	CCTTGGTACC
DE177	CGACAACAGA	TCCTATAGTA	TTTAATTATG	AACCCCATCA	CCTTGGCACC
AR39	CGACAACAGA	TCCTATAGTA	TTTAATTATG	AACCCCATCA	CCTTGGCACC
CWL029	CGACAACAGA	TCCTATAGTA	TTTAATTATG	AACCCCATCA	CCTTGGCACC
J138	CGACAACAGA	TCCTATAGTA	TTTAATTATG	AACCCCATCA	CCTTGGCACC
TW183	CGACAACAGA	TCCTATAGTA	TTTAATTATG	AACCCCATCA	CCTTGGCACC
WA97001	CGACAACAGA	TCCTATAGTA	TTTAATTATG	AACCCCATCA	CCTTGGCACC
SH511	CGACAACAGA	TCCTATAGTA	TTTAATTATG	AACCCCATCA	CCTTGGCACC
TOR1	CGACAACAGA	TCCTATAGTA	TTTAATTATG	AACCCCATCA	CCTTGGCACC

	510	520	530	540	550
LPCoLN	GTGTTGTTTT	CCGGAATCAA	TGTAGATTCT	AACGCAACAA	ATCCATTGAA
DE177	GTGTTGTTTT	CCGGAATCAA	TGTAGATTCT	AACGCAACAA	ATCCATTGAA
AR39	GTGTTGTTTT	CCGGAATCAA	TGTAGATTCT	AACGCAACAA	ATCCATTGAA
CWL029	GTGTTGTTTT	CCGGAATCAA	TGTAGATTCT	AACGCAACAA	ATCCATTGAA
J138	GTGTTGTTTT	CCGGAATCAA	TGTAGATTCT	AACGCAACAA	ATCCATTGAA
TW183	GTGTTGTTTT	CCGGAATCAA	TGTAGATTCT	AACGCAACAA	ATCCATTGAA
WA97001	GTGTTGTTTT	CCGGAATCAA	TGTAGATTCT	AACGCAACAA	ATCCATTGAA
SH511	GTGTTGTTTT	CCGGAATCAA	TGTAGATTCT	AACGCAATAA	ATCCATTGAA
TOR1	GTGTTGTTTT	CCGGAATCAA	TGTAGATTCT	AACGCAACAA	ATCCATTGAA

	560	570	580	590	600
LPCoLN	CTTCCTATCA	AAACTTTCTA	ACTCTTCACG	ACTTGAAAGG	GGTGTGCTCG
DE177	CTTCCTATCA	AAATTTTCTA	ACTCTTCACG	ACTTGAAAGG	GGTGTGCTCG
AR39	CTTCCTATCA	AAATTTTCTA	ACTCTTCACG	ACTTGAAAGG	GGTGTGCTCG
CWL029	CTTCCTATCA	AAATTTTCTA	ACTCTTCACG	ACTTGAAAGG	GGTGTGCTCG
J138	CTTCCTATCA	AAATTTTCTA	ACTCTTCACG	ACTTGAAAGG	GGTGTGCTCG
TW183	CTTCCTATCA	AAATTTTCTA	ACTCTTCACG	ACTTGAAAGG	GGTGTGCTCG
WA97001	CTTCCTATCA	AAATTTTCTA	ACTCTTCACG	ACTTGAAAGG	GGTGTGCTCG
SH511	CTTCCTATCA	AAATTTTCTA	ACTCTTCACG	ACTTGAAAGG	GGTGTGCTCG
TOR1	CTTCCTATCA	AAATTTTCTA	ACTCTTCACG	ACTTGAAAGG	GGTGTGCTCG

	610	620	630	640	650
Identity					
LPCoLN	CTATTGAAGA	TCGGGCTGCT	ATTTCTTGCA	AAACCCTATC	GCAAACTGGG
DE177	CTATTGAAGA	TCGGGCTGCT	ATTTCTTGCA	AAACCCTATC	GCAAACTGGG
AR39	CTATTGAAGA	TCGGGCTGCT	ATTTCTTGCA	AAACCCTATC	GCAAACTGGG
CWL029	CTATTGAAGA	TCGGGCTGCT	ATTTCTTGCA	AAACCCTATC	GCAAACTGGG
J138	CTATTGAAGA	TCGGGCTGCT	ATTTCTTGCA	AAACCCTATC	GCAAACTGGG
TW183	CTATTGAAGA	TCGGGCTGCT	ATTTCTTGCA	AAACCCTATC	GCAAACTGGG
WA97001	CTATTGAAGA	TCGGGCTGCT	ATTTCTTGCA	AAACCCTATC	GCAAACTGGG
SH511	CTATTGAAGA	TCGGGCTGCT	ATTTCTTGCA	AAACCCTATC	GCAAACTGGG
TOR1	CTATTGAAGA	TCGGGCTGCT	ATTTCTTGCA	AAACCCTATC	GCAAACTGGG

	660	670	680	690	700
Identity					
LPCoLN	GGCACTCTAC	GTTTAGGAAA	CGCAGCATTA	ATCAGGACGA	AAGGCCCGGG
DE177	GGCATTCTAC	GTTTAGGAAA	CGCAGCATTA	ATCAGGACGA	AAGGCCCGGG
AR39	GGCATTCTAC	GTTTAGGAAA	CGCAGCATTA	ATCAGGACGA	AAGGCCCGGG
CWL029	GGCATTCTAC	GTTTAGGAAA	CGCAGCATTA	ATCAGGACGA	AAGGCCCGGG
J138	GGCATTCTAC	GTTTAGGAAA	CGCAGCATTA	ATCAGGACGA	AAGGCCCGGG
TW183	GGCATTCTAC	GTTTAGGAAA	CGCAGCATTA	ATCAGGACGA	AAGGCCCGGG
WA97001	GGCATTCTAC	GTTTAGGAAA	CGCAGCATTA	ATCAGGACGA	AAGGCCCGGG
SH511	GGCATTCTAC	GTTTAGGAAA	CGCAGCATTA	ATCAGGACGA	AAGGCCCGGG
TOR1	GGCATTCTAC	GTTTAGGAAA	CGCAGCATTA	ATCAGGACGA	AAGGCCCGGG

	710	720	730	740	750
Identity					
LPCoLN	AAGCTCCATA	AATTTTAATG	CAGTCGCTAT	CAACCTTCCT	TCTATTTTAC
DE177	AAGCTCCATA	AATTTTAATG	CAATCGCGAT	CAATCTTCCT	TCTATTTTAC
AR39	AAGCTCCATA	AATTTTAATG	CAATCGCGAT	CAATCTTCCT	TCTATTTTAC
CWL029	AAGCTCCATA	AATTTTAATG	CAATCGCGAT	CAATCTTCCT	TCTATTTTAC
J138	AAGCTCCATA	AATTTTAATG	CAATCGCGAT	CAATCTTCCT	TCTATTTTAC
TW183	AAGCTCCATA	AATTTTAATG	CAATCGCGAT	CAATCTTCCT	TCTATTTTAC
WA97001	AAGCTCCATA	AATTTTAATG	CAATCGCGAT	CAATCTTCCT	TCTATTTTAC
SH511	AAGCTCCATA	AATTTTAATG	CAATCGCGAT	CAATCTTCCT	TCTATTTTAC
TOR1	AAGCTCCATA	AATTTTAATG	CAATCGCGAT	CAATCTTCCT	TCTATTTTAC

	760	770	780	790	800
Identity					
LPCoLN	AATCAGAAGC	CTCAGCTCCA	AAGTTCTGGA	TTTATCCTAC	ATTAACAGGA
DE177	AATCAGAAGC	CTCAGCTCCA	AAGTTCTGGA	TTTATCCTAC	ATTAACAGGA
AR39	AATCAGAAGC	CTCAGCTCCA	AAGTTCTGGA	TTTATCCTAC	ATTAACAGGA
CWL029	AATCAGAAGC	CTCAGCTCCA	AAGTTCTGGA	TTTATCCTAC	ATTAACAGGA
J138	AATCAGAAGC	CTCAGCTCCA	AAGTTCTGGA	TTTATCCTAC	ATTAACAGGA
TW183	AATCAGAAGC	CTCAGCTCCA	AAGTTCTGGA	TTTATCCTAC	ATTAACAGGA
WA97001	AATCAGAAGC	CTCAGCTCCA	AAGTTCTGGA	TTTATCCTAC	ATTAACAGGA
SH511	AATCAGAAGC	CTCAGCTCCA	AAGTTCTGGA	TTTATCCTAC	ATTAACAGGA
TOR1	AATCAGAAGC	CTCAGCTCCA	AAGTTCTGGA	TTTATCCTAC	ATTAACAGGA

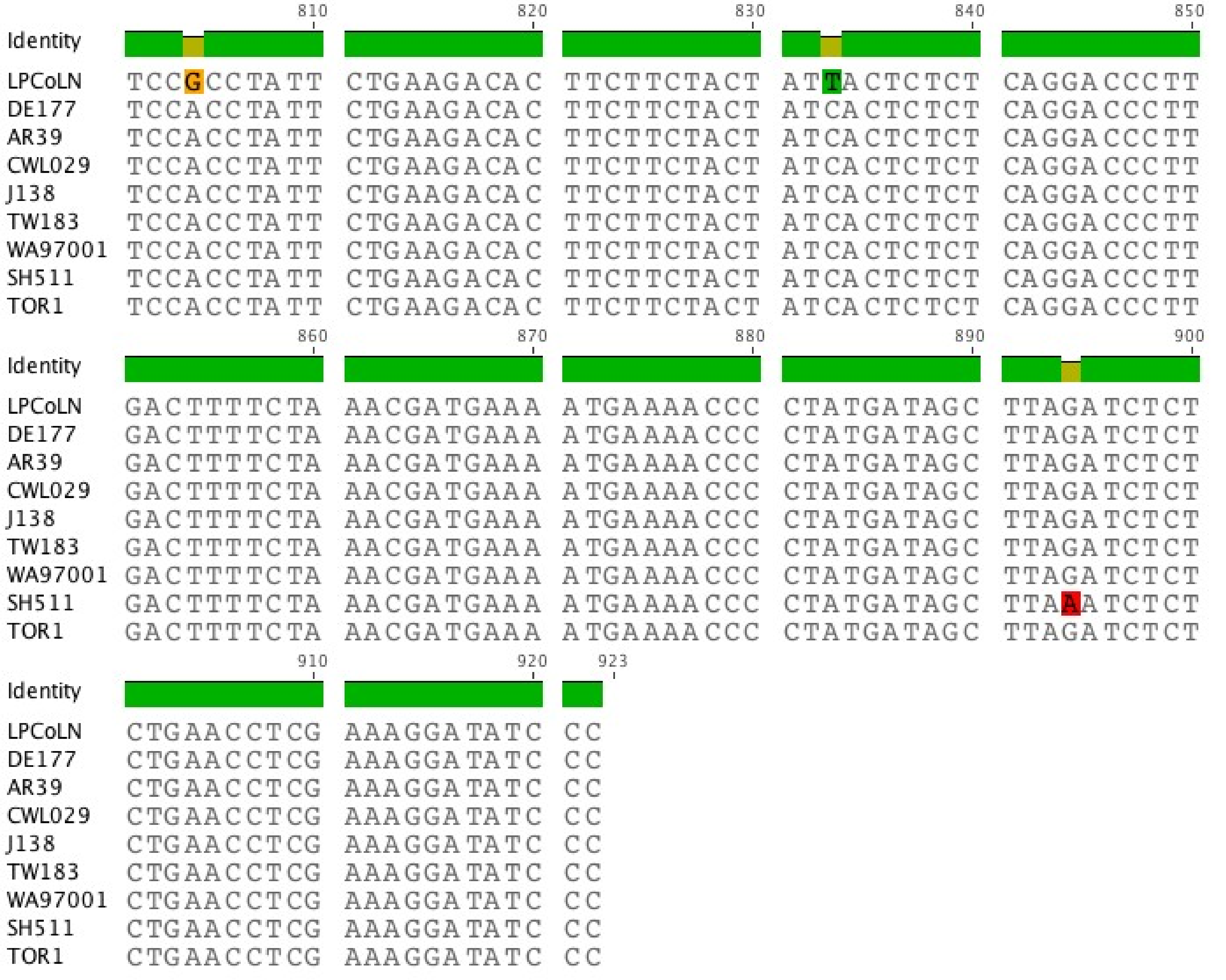
810
820
830
840
850
Identity
LPCoLN TCCGCCTATT CTGAAGACAC TTCTTCTACT ATTACTCTCT CAGGACCCTT
DE177 TCCACCTATT CTGAAGACAC TTCTTCTACT ATCACTCTCT CAGGACCCTT
AR39 TCCACCTATT CTGAAGACAC TTCTTCTACT ATCACTCTCT CAGGACCCTT
CWL029 TCCACCTATT CTGAAGACAC TTCTTCTACT ATCACTCTCT CAGGACCCTT
J138 TCCACCTATT CTGAAGACAC TTCTTCTACT ATCACTCTCT CAGGACCCTT
TW183 TCCACCTATT CTGAAGACAC TTCTTCTACT ATCACTCTCT CAGGACCCTT
WA97001 TCCACCTATT CTGAAGACAC TTCTTCTACT ATCACTCTCT CAGGACCCTT
SH511 TCCACCTATT CTGAAGACAC TTCTTCTACT ATCACTCTCT CAGGACCCTT
TOR1 TCCACCTATT CTGAAGACAC TTCTTCTACT ATCACTCTCT CAGGACCCTT
860
870
880
890
900
Identity
LPCoLN GACTTTTCTA AACGATGAAA ATGAAAACCC CTATGATAGC TTAGATCTCT
DE177 GACTTTTCTA AACGATGAAA ATGAAAACCC CTATGATAGC TTAGATCTCT
AR39 GACTTTTCTA AACGATGAAA ATGAAAACCC CTATGATAGC TTAGATCTCT
CWL029 GACTTTTCTA AACGATGAAA ATGAAAACCC CTATGATAGC TTAGATCTCT
J138 GACTTTTCTA AACGATGAAA ATGAAAACCC CTATGATAGC TTAGATCTCT
TW183 GACTTTTCTA AACGATGAAA ATGAAAACCC CTATGATAGC TTAGATCTCT
WA97001 GACTTTTCTA AACGATGAAA ATGAAAACCC CTATGATAGC TTAGATCTCT
SH511 GACTTTTCTA AACGATGAAA ATGAAAACCC CTATGATAGC TTAAATCTCT
TOR1 GACTTTTCTA AACGATGAAA ATGAAAACCC CTATGATAGC TTAGATCTCT
910
920
923
Identity
LPCoLN CTGAACCTCG AAAGGATATC CC
DE177 CTGAACCTCG AAAGGATATC CC
AR39 CTGAACCTCG AAAGGATATC CC
CWL029 CTGAACCTCG AAAGGATATC CC
J138 CTGAACCTCG AAAGGATATC CC
TW183 CTGAACCTCG AAAGGATATC CC
WA97001 CTGAACCTCG AAAGGATATC CC
SH511 CTGAACCTCG AAAGGATATC CC
TOR1 CTGAACCTCG AAAGGATATC CC

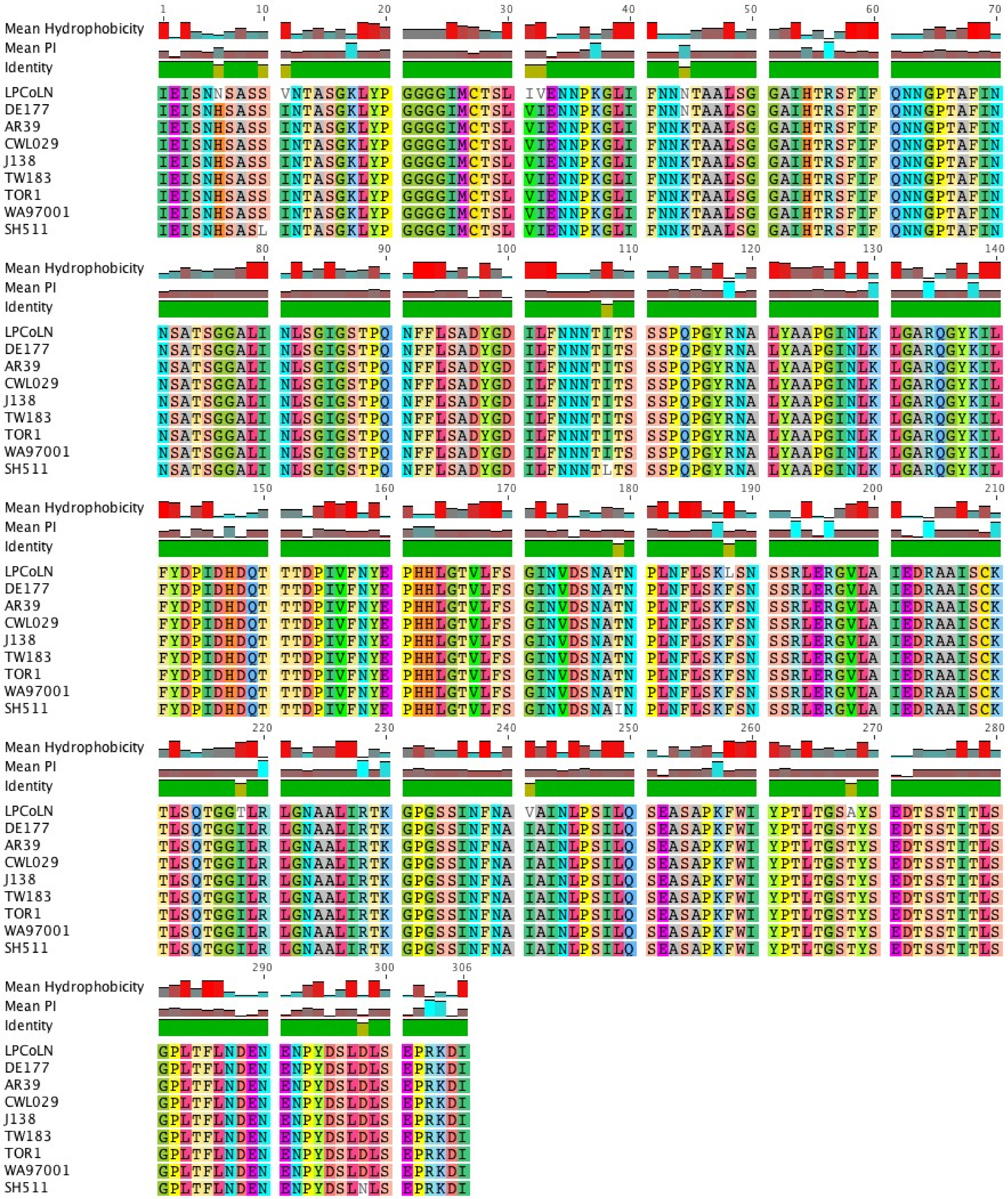

Mean Hydrophobicity
Mean PI
Identity
1 10 20 30 40 50 60 70
LPCoLN IEISNNSASS VNTASGKLYP GGGGIMCTSL IVENNPKGLI FNNNTAALSG GAIHTRSFIF QNNGPTAFIN
DE177 IEISNHSASS INTASGKLYP GGGGIMCTSL VIENNPKGLI FNNNTAALSG GAIHTRSFIF QNNGPTAFIN
AR39 IEISNHSASS INTASGKLYP GGGGIMCTSL VIENNPKGLI FNNKTAALSG GAIHTRSFIF QNNGPTAFIN
CWL029 IEISNHSASS INTASGKLYP GGGGIMCTSL VIENNPKGLI FNNKTAALSG GAIHTRSFIF QNNGPTAFIN
J138 IEISNHSASS INTASGKLYP GGGGIMCTSL VIENNPKGLI FNNKTAALSG GAIHTRSFIF QNNGPTAFIN
TW183 IEISNHSASS INTASGKLYP GGGGIMCTSL VIENNPKGLI FNNKTAALSG GAIHTRSFIF QNNGPTAFIN
TOR1 IEISNHSASS INTASGKLYP GGGGIMCTSL VIENNPKGLI FNNKTAALSG GAIHTRSFIF QNNGPTAFIN
WA97001 IEISNHSASS INTASGKLYP GGGGIMCTSL VIENNPKGLI FNNKTAALSG GAIHTRSFIF QNNGPTAFIN
SH511 IEISNHSASL INTASGKLYP GGGGIMCTSL VIENNPKGLI FNNKTAALSG GAIHTRSFIF QNNGPTAFIN
80 90 100 110 120 130 140
LPCoLN NSATSGGALI NLSGIGSTPQ NFFLSADYGD ILFNNNTITS SSPQPGYRNA LYAAPGINLK LGARQGYKIL
DE177 NSATSGGALI NLSGIGSTPQ NFFLSADYGD ILFNNNTITS SSPQPGYRNA LYAAPGINLK LGARQGYKIL
AR39 NSATSGGALI NLSGIGSTPQ NFFLSADYGD ILFNNNTITS SSPQPGYRNA LYAAPGINLK LGARQGYKIL
CWL029 NSATSGGALI NLSGIGSTPQ NFFLSADYGD ILFNNNTITS SSPQPGYRNA LYAAPGINLK LGARQGYKIL
J138 NSATSGGALI NLSGIGSTPQ NFFLSADYGD ILFNNNTITS SSPQPGYRNA LYAAPGINLK LGARQGYKIL
TW183 NSATSGGALI NLSGIGSTPQ NFFLSADYGD ILFNNNTITS SSPQPGYRNA LYAAPGINLK LGARQGYKIL
TOR1 NSATSGGALI NLSGIGSTPQ NFFLSADYGD ILFNNNTITS SSPQPGYRNA LYAAPGINLK LGARQGYKIL
WA97001 NSATSGGALI NLSGIGSTPQ NFFLSADYGD ILFNNNTITS SSPQPGYRNA LYAAPGINLK LGARQGYKIL
SH511 NSATSGGALI NLSGIGSTPQ NFFLSADYGD ILFNNNTLTS SSPQPGYRNA LYAAPGINLK LGARQGYKIL
150 160 170 180 190 200 210
LPCoLN FYDPIDHDQT TTDPIVFNYE PHHLGTVLFS GINVDSNATN PLNFLSKLSN SSRLERGVLA IEDRAAISCK
DE177 FYDPIDHDQT TTDPIVFNYE PHHLGTVLFS GINVDSNATN PLNFLSKFSN SSRLERGVLA IEDRAAISCK
AR39 FYDPIDHDQT TTDPIVFNYE PHHLGTVLFS GINVDSNATN PLNFLSKFSN SSRLERGVLA IEDRAAISCK
CWL029 FYDPIDHDQT TTDPIVFNYE PHHLGTVLFS GINVDSNATN PLNFLSKFSN SSRLERGVLA IEDRAAISCK
J138 FYDPIDHDQT TTDPIVFNYE PHHLGTVLFS GINVDSNATN PLNFLSKFSN SSRLERGVLA IEDRAAISCK
TW183 FYDPIDHDQT TTDPIVFNYE PHHLGTVLFS GINVDSNATN PLNFLSKFSN SSRLERGVLA IEDRAAISCK
TOR1 FYDPIDHDQT TTDPIVFNYE PHHLGTVLFS GINVDSNATN PLNFLSKFSN SSRLERGVLA IEDRAAISCK
WA97001 FYDPIDHDQT TTDPIVFNYE PHHLGTVLFS GINVDSNATN PLNFLSKFSN SSRLERGVLA IEDRAAISCK
SH511 FYDPIDHDQT TTDPIVFNYE PHHLGTVLFS GINVDSNAIN PLNFLSKFSN SSRLERGVLA IEDRAAISCK
220 230 240 250 260 270 280
LPCoLN TLSQTGGTLR LGNAALIRTK GPGSSINFNA VAINLPSILQ SEASAPKFWI YPTLTGSAYS EDTSSTITLS
DE177 TLSQTGGILR LGNAALIRTK GPGSSINFNA IAINLPSILQ SEASAPKFWI YPTLTGSTYS EDTSSTITLS
AR39 TLSQTGGILR LGNAALIRTK GPGSSINFNA IAINLPSILQ SEASAPKFWI YPTLTGSTYS EDTSSTITLS
CWL029 TLSQTGGILR LGNAALIRTK GPGSSINFNA IAINLPSILQ SEASAPKFWI YPTLTGSTYS EDTSSTITLS
J138 TLSQTGGILR LGNAALIRTK GPGSSINFNA IAINLPSILQ SEASAPKFWI YPTLTGSTYS EDTSSTITLS
TW183 TLSQTGGILR LGNAALIRTK GPGSSINFNA IAINLPSILQ SEASAPKFWI YPTLTGSTYS EDTSSTITLS
TOR1 TLSQTGGILR LGNAALIRTK GPGSSINFNA IAINLPSILQ SEASAPKFWI YPTLTGSTYS EDTSSTITLS
WA97001 TLSQTGGILR LGNAALIRTK GPGSSINFNA IAINLPSILQ SEASAPKFWI YPTLTGSTYS EDTSSTITLS
SH511 TLSQTGGILR LGNAALIRTK GPGSSINFNA IAINLPSILQ SEASAPKFWI YPTLTGSTYS EDTSSTITLS
290 300 306
LPCoLN GPLTFLNDEN ENPYDSLDLS EPRKDI
DE177 GPLTFLNDEN ENPYDSLDLS EPRKDI
AR39 GPLTFLNDEN ENPYDSLDLS EPRKDI
CWL029 GPLTFLNDEN ENPYDSLDLS EPRKDI
J138 GPLTFLNDEN ENPYDSLDLS EPRKDI
TW183 GPLTFLNDEN ENPYDSLDLS EPRKDI
TOR1 GPLTFLNDEN ENPYDSLDLS EPRKDI
WA97001 GPLTFLNDEN ENPYDSLDLS EPRKDI
SH511 GPLTFLNDEN ENPYDSLNLS EPRKDI
