# Supplementary material for: Chlamydia pneumoniae Is Genetically Diverse in Animals and Appears to Have Crossed the Host Barrier to Humans on (At Least) Two Occasions
Source: PLoS Pathog. 2010 May 20;6(5):e1000903. doi: 10.1371/journal.ppat.1000903 (PMC2873915; doi:10.1371/journal.ppat.1000903)
Supplement: Figure S7 — Multiple sequence alignment of 16S rRNA. The nucleotide alignment was generated using Geneious version 4.7, where each nucleotide is assigned its own colour. The total alignment length is 215 bp. (4.92 MB PDF) [file ppat.1000903.s007.pdf]

	1–10	11–20	21–30	31–40	41–50
B10	TAGTGGCGGA	AGGGTTAGTA	ATACATA-GA	TAATCTGCCC	TCAACTTGGG
B26	TAGTGGCGGA	AGGGTTAGTA	ATACATA-GA	TAATCTGCCC	TCAACTTGGG
B37	TAGTGGCGGA	AGGGTTAGTA	ATACATA-GA	TAATCTGCCC	TCAACTTGGG
WBB	TAGTGGCGGA	AGGGTTAGTA	ATACATA-GA	TAATCTGCCC	TCAACTTGGG
EBB	TAGTGGCGGA	AGGGTTAGTA	ATACATA-GA	TAATCTGCCC	TCAACTTGGG
LPCoLN	TAGTGGCGGA	AGGGTTAGTA	ATACATA-GA	TAATCTGCCC	TCAACTTGGG
Pot37	TAGTGGCGGA	AGGGTTAGTA	ATACATA-GA	TAATCTGCCC	TCAACTTGGG
GBF	TAGTGGCGGA	AGGGTTAGTA	ATACATA-GA	TAATCTGCCC	TCAACTTGGG
2040.3	TAGTGGCGGA	AGGGTTAGTA	ATACATA-GA	TAATCTGCCC	TCAACTTGGG
CPXT1	TAGTGGCGGA	AGGGTTAGTA	ATACATA-GA	TAATCTGCCC	TCAACTTGGG
DE177	TAGTGGCGGA	AGGGTTAGTA	ATACATA-GA	TAATCTGCCC	TCAACTTGGG
BMTF-type1	TAGTGGCGGA	AGGGTTAGTA	GTACATAAGA	TAATCTGCCC	TCAACTTGGG
BMTF-type2	TAGTGGCGGA	AGGGTTAGTA	GTACATA-GA	TAATCTGCCC	TCAACTTGGG
Iguana	TAGTGGCGGA	AGGGTTAGTA	GTACATA-GA	TAATCTGCCC	TCAACTTGGG
Puffadd	TAGTGGCGGA	AGGGTTAGTA	GTACATAAGA	TAATCTGCCC	TCAACTTGGG
N16	TAGTGGCGGA	AGGGTTAGTA	ATACATA-GA	TAATCTGCCC	TCAACTTGGG
AR39	TAGTGGCGGA	AGGGTTAGTA	GTACATA-GA	TAATCTGCCC	TCAACTTGGG
CWL029	TAGTGGCGGA	AGGGTTAGTA	GTACATA-GA	TAATCTGCCC	TCAACTTGGG
J138	TAGTGGCGGA	AGGGTTAGTA	GTACATA-GA	TAATCTGCCC	TCAACTTGGG
TW183	TAGTGGCGGA	AGGGTTAGTA	GTACATA-GA	TAATCTGCCC	TCAACTTGGG
IOL207	TAGTGGCGGA	AGGGTTAGTA	GTACATA-GA	TAATCTGCCC	TCAACTTGGG
LKK1	TAGTGGCGGA	AGGGTTAGTA	GTACATA-GA	TAATCTGCCC	TCAACTTGGG
TOR1	TAGTGGCGGA	AGGGTTAGTA	GTACATA-GA	TAATCTGCCC	TCAACTTGGG
WA97001	TAGTGGCGGA	AGGGTTAGTA	GTACATA-GA	TAATCTGCCC	TCAACTTGGG
1979	TAGTGGCGGA	AGGGTTAGTA	ATACATA-GA	TAATCTGCCC	TCAACTTGGG
SH511	TAGTGGCGGA	AGGGTTAGTA	ATACATA-GA	TAATCTGCCC	TCAACTTGGG

	51–60	61–70	71–80	81–90	91–100
B10	AATAACGGTT	GGAAACGATC	GCTAATACCG	AAT--GTAGT	GTAATTAGGC
B26	AATAACGGTT	GGAAACGATC	GCTAATACCG	AAT--GTAGT	GTAATTAGGC
B37	AATAACGGTT	GGAAACGATC	GCTAATACCG	AAT--GTAGT	GTAATTAGGC
WBB	AATAACGGTT	GGAAACGATC	GCTAATACCG	AAT--GTAGT	GTAATTAGGC
EBB	AATAACGGTT	GGAAACGATC	GCTAATACCG	AAT--GTAGT	GTAATTAGGC
LPCoLN	AATAACGGTT	GGAAACGATC	GCTAATACCG	AAT--GTAGT	GTAATTAGGC
Pot37	AATAACGGTT	GGAAACGATC	GCTAATACCG	AAT--GTAGT	GTAATTAGGC
GBF	AATAACGGTT	GGAAACGATC	GCTAATACCG	AAT--GTAGT	GTAATTAGGC
2040.3	AATAACGGTT	GGAAACGATC	GCTAATACCG	AAT--GTAGT	GTAATTAGGC
CPXT1	AATAACGGTT	GGAAACGATC	GCTAATACCG	AAT--GTAGT	GTAATTAGGC
DE177	AATAACGGTT	GGAAACGATC	GCTAATACCG	AATTTGTAGT	GTAATTAGGC
BMTF-type1	GATAACGGTT	GGAAACGATC	GCTAATACCG	AAT--GTAGT	GTAATTAGGC
BMTF-type2	GATAACGGTT	GGAAACGATC	GCTAATACCG	AAT--GTAGT	GTAATTAGGC
Iguana	GATAACGGTT	GGAAACGATC	GCTAATACCG	AAT--GTAGT	GTAATTAGGC
Puffadd	GATAACGGTT	GGAAACGATC	GCTAATACCG	AAT--GTAGT	GTAATTAGGC
N16	AATAACGGTT	GGAAACGATC	GCTAATACCG	AAT--GTGGT	GTAATTAGGA
AR39	GATAACGGTT	GGAAACGATC	GCTAATACCG	AAT--GTAGT	GTAATTAGGC
CWL029	GATAACGGTT	GGAAACGATC	GCTAATACCG	AAT--GTAGT	GTAATTAGGC
J138	GATAACGGTT	GGAAACGATC	GCTAATACCG	AAT--GTAGT	GTAATTAGGC
TW183	GATAACGGTT	GGAAACGATC	GCTAATACCG	AAT--GTAGT	GTAATTAGGC
IOL207	GATAACGGTT	GGAAACGATC	GCTAATACCG	AAT--GTAGT	GTAATTAGGC
LKK1	GATAACGGTT	GGAAACGATC	GCTAATACCG	AAT--GTAGT	GTAATTAGGC
TOR1	GATAACGGTT	GGAAACGATC	GCTAATACCG	AAT--GTAGT	GTAATTAGGC
WA97001	GATAACGGTT	GGAAACGATC	GCTAATACCG	AAT--GTAGT	GTAATTAGGC
1979	AATAACGGTT	GGAAACGATC	GCTAATACCG	AAT--GTAGT	GTAATTAGGC
SH511	AATAACGGTT	GGAAACGATC	GCTAATACCG	AAT--GTAGT	GTAATTAGGC

	110	120	130	140	150
Identity					
B10	ATCTAATATA	TATTAAAGAA	GGGGATCTTC	GGACCTTTCG	GTTGAGGAAG
B26	ATCTAATATA	TATTAAAGAA	GGGGATCTTC	GGACCTTTCG	GTTGAGGAAG
B37	ATCTAATATA	TATTAAAGAA	GGGGATCTTC	GGACCTTTCG	GTTGAGGAAG
WBB	ATCTAATATA	TATTAAAGAA	GGGGATCTTC	GGACCTTTCG	GTTGAGGAAG
EBB	ATCTAATATA	TATTAAAGAA	GGGGATCTTC	GGACCTTTCG	GTTGAGGAAG
LPCoLN	ATCTAATATA	TATTAAAGAA	GGGGATCTTC	GGACCTTTCG	GTTGAGGAAG
Pot37	ATCTAATATA	TATTAAAGAA	GGGGATCTTC	GGACCTTTCG	GTTGAGGAAG
GBF	ATCTAATATA	TATTAAAGAA	GGGGATCTTC	GGACCTTTCG	GTTGAGGAAG
2040.3	ATCTAATATA	TATTAAAGAA	GGGGATCTTC	GGACCTTTCG	GTTGAGGAAG
CPXT1	ATCTAATATA	TATTAAAGAA	GGGGATCTTC	GGACCTTTCG	GTTGAGGAAG
DE177	ATCTAATATA	TATTAAAGAA	GGGGATCTTC	GGACCTTTCG	GTTGAGGAAG
BMTF-type1	ATCTAATATA	TATTAAAGAA	GGGGATCTTC	GGACCTTTCG	GTTGAGGAAG
BMTF-type2	ATCTAATATA	TATTAAAGAA	GGGGATCTTC	GGACCTTTCG	GTTGAGGAAG
Iguana	ATCTAATATA	TATTAAAGAA	GGGGATCTTC	GGACCTTTCG	GTTGAGGAAG
Puffadd	ATCTAATATA	TATTAAAGAA	GGGGATCTTC	GGACCTTTCG	GTTGAGGAAG
N16	ATCTAATATA	TATTAAAGAA	GGGGATCTTC	GGACCTTTCG	GTTGAGGAAG
AR39	ATCTAATATA	TATTAAAGAA	GGGGATCTTC	GGACCTTTCG	GTTGAGGAAG
CWL029	ATCTAATATA	TATTAAAGAA	GGGGATCTTC	GGACCTTTCG	GTTGAGGAAG
J138	ATCTAATATA	TATTAAAGAA	GGGGATCTTC	GGACCTTTCG	GTTGAGGAAG
TW183	ATCTAATATA	TATTAAAGAA	GGGGATCTTC	GGACCTTTCG	GTTGAGGAAG
IOL207	ATCTAATATA	TATTAAAGAA	GGGGATCTTC	GGACCTTTCG	GTTGAGGAAG
LKK1	ATCTAATATA	TATTAAAGAA	GGGGATCTTC	GGACCTTTCG	GTTGAGGAAG
TOR1	ATCTAATATA	TATTAAAGAA	GGGGATCTTC	GGACCTTTCG	GTTGAGGAAG
WA97001	ATCTAATATA	TATTAAAGAA	GGGGATCTTC	GGACCTTTCG	GTTGAGGAAG
1979	ATCTAATATA	TATTAAAGAA	GGGGATCTTC	GGACCTTTCG	GTTGAGGAAG
SH511	ATCTAATATA	TATTAAAGAA	GGGGATCTTC	GGACCTTTCG	GTTGAGGAAG

	160	170	180	190	200
Identity					
B10	AGTTTATGCG	ATATCAGCTT	GTTGGTGGGG	TAAAAGCCCA	CCAAGGCGAT
B26	AGTTTATGCG	ATATCAGCTT	GTTGGTGGGG	TAAAAGCCCA	CCAAGGCGAT
B37	AGTTTATGCG	ATATCAGCTT	GTTGGTGGGG	TAAAAGCCCA	CCAAGGCGAT
WBB	AGTTTATGCG	ATATCAGCTT	GTTGGTGGGG	TAAAAGCCCA	CCAAGGCGAT
EBB	AGTTTATGCG	ATATCAGCTT	GTTGGTGGGG	TAAAAGCCCA	CCAAGGCGAT
LPCoLN	AGTTTATGCG	ATATCAGCTT	GTTGGTGGGG	TAAAAGCCCA	CCAAGGCGAT
Pot37	AGTTTATGCG	ATATCAGCTT	GTTGGTGGGG	TAAAAGCCCA	CCAAGGCGAT
GBF	AGTTTATGCG	ATATCAGCTT	GTTGGTGGGG	TAAAAGCCCA	CCAAGGCGAT
2040.3	AGTTTATGCG	ATATCAGCTT	GTTGGTGGGG	TAAAAGCCCA	CCAAGGCGAT
CPXT1	AGTTTATGCG	ATATCAGCTT	GTTGGTGGGG	TAAAAGCCCA	CCAAGGCGAT
DE177	AGTTTATGCG	ATATCAGCTT	GTTGGTGGGG	TAAAAGCCCA	CCAAGGCGAT
BMTF-type1	AGTTTATGCG	ATATCAGCTT	GTTGGTGGGG	TAAAAGCCCA	CCAAGGCGAT
BMTF-type2	AGTTTATGCG	ATATCAGCTT	GTTGGTGGGG	TAAAAGCCCA	CCAAGGCGAT
Iguana	AGTTTATGCG	ATATCAGCTT	GTTGGTGGGG	TAAAAGCCCA	CCAAGGCGAT
Puffadd	AGTTTATGCG	ATATCAGCTT	GTTGGTGGGG	TAAAAGCCCA	CCAAGGCGAT
N16	AGTTTATGCG	ATATCAGCTT	GTTGGTGGGG	TAAAAGCCCA	CCAAGGCGAT
AR39	AGTTTATGCG	ATATCAGCTT	GTTGGTGGGG	TAAAAGCCCA	CCAAGGCGAT
CWL029	AGTTTATGCG	ATATCAGCTT	GTTGGTGGGG	TAAAAGCCCA	CCAAGGCGAT
J138	AGTTTATGCG	ATATCAGCTT	GTTGGTGGGG	TAAAAGCCCA	CCAAGGCGAT
TW183	AGTTTATGCG	ATATCAGCTT	GTTGGTGGGG	TAAAAGCCCA	CCAAGGCGAT
IOL207	AGTTTATGCG	ATATCAGCTT	GTTGGTGGGG	TAAAAGCCCA	CCAAGGCGAT
LKK1	AGTTTATGCG	ATATCAGCTT	GTTGGTGGGG	TAAAAGCCCA	CCAAGGCGAT
TOR1	AGTTTATGCG	ATATCAGCTT	GTTGGTGGGG	TAAAAGCCCA	CCAAGGCGAT
WA97001	AGTTTATGCG	ATATCAGCTT	GTTGGTGGGG	TAAAAGCCCA	CCAAGGCGAT
1979	AGTTTATGCG	ATATCAGCTT	GTTGGTGGGG	TAAAAGCCCA	CCAAGGCGAT
SH511	AGTTTATGCG	ATATCAGCTT	GTTGGTGGGG	TAAAAGCCCA	CCAAGGCGAT

	210	215
Identity		
B10	GACGTCTAGG	CGGAT
B26	GACGTCTAGG	CGGAT
B37	GACGTCTAGG	CGGAT
WBB	GACGTCTAGG	CGGAT
EBB	GACGTCTAGG	CGGAT
LPCoLN	GACGTCTAGG	CGGAT
Pot37	GACGTCTAGG	CGGAT
GBF	GACGTCTAGG	CGGAT
2040.3	GACGTCTAGG	CGGAT
CPXT1	GACGTCTAGG	CGGAT
DE177	GACGTCTAGG	CGGAT
BMTF-type1	GACGTCTAGG	CGGAT
BMTF-type2	GACGTCTAGG	CGGAT
Iguana	GACGTCTAGG	CGGAT
Puffadd	GACGTCTAGG	CGGAT
N16	GACGTCTAGG	CGGAT
AR39	GACGTCTAGG	CGGAT
CWL029	GACGTCTAGG	CGGAT
J138	GACGTCTAGG	CGGAT
TW183	GACGTCTAGG	CGGAT
IOL207	GACGTCTAGG	CGGAT
LKK1	GACGTCTAGG	CGGAT
TOR1	GACGTCTAGG	CGGAT
WA97001	GACGTCTAGG	CGGAT
1979	GACGTCTAGG	CGGAT
SH511	GACGTCTAGG	CGGAT
