# Supplementary material for: Chlamydia pneumoniae Is Genetically Diverse in Animals and Appears to Have Crossed the Host Barrier to Humans on (At Least) Two Occasions
Source: PLoS Pathog. 2010 May 20;6(5):e1000903. doi: 10.1371/journal.ppat.1000903 (PMC2873915; doi:10.1371/journal.ppat.1000903)
Supplement: Figure S8 — Multiple sequence alignment of ompA . The nucleotide and amino acid alignments were generated using Geneious version 4.7, where each nucleotide and amino acid is assigned its own colour. White shading indicates an amino acid variant. (1.42 MB PDF) [file ppat.1000903.s008.pdf]

	1–10	11–20	21–30	31–40	41–50
Identity					
B26	ACGCTGGCGT	AGCAACAGCT	ACTGGAACAA	AGTCTGCGAC	CATCAATTAT
B37	ACGCTGGCGT	AGCAACAGCT	ACTGGAACAA	AGTCTGCGAC	CATCAATTAT
WBB	ACGCTGGCGT	AGCAACAGCT	ACTGGAACAA	AGTCTGCGAC	CATCAATTAT
EBB	ACGCTGGCGT	AGCAACAGCT	ACTGGAACAA	AGTCTGCGAC	CATCAATTAT
LPCoLN	ACGCTGGCGT	AGCAACAGCT	ACTGGAACAA	AGTCTGCGAC	CATCAATTAT
Pot37	ACGCTGGCGT	AGCAACAGCT	ACTGGAACAA	AGTCTGCGAC	CATCAATTAT
CPXT1	ACGCTGGCGT	AGCAACAGCT	ACTGGAACAA	AGTCTGCGAC	CATCAATTAT
DE177	ACGCTGGCGT	AGCAACAGCT	ACTGGAACAA	AGTCTGCGAC	CATCAATTAT
GBF	ACGCTGGCGT	AGCAACAGCT	ACTGGAACAA	AGTCTGCGAC	CATCAATTAT
BMTF-type1	ACGCTGGCGT	AGCAACAGCT	ACTGGAACAA	AGTCTGCGAC	CATCAATTAT
Burpyth	ACGCTGGCGT	AGCAACAGCT	ACTGGAACAA	AGTCTGCGAC	CATCAATTAT
PuffAdd	ACGCTGGCGT	AGCAACAGCT	ACTGGAACAA	AGTCTGCGAC	CATCAATTAT
cham	ACGCTGGCGT	AGCAACAGCT	ACTGGAACAA	AGTCTGCGAC	CATCAATTAT
Iguana	ACGCTGGCGT	AGCAACAGCT	ACTGGAACAA	AGTCTGCGAC	CATCAATTAT
GST	ACGCTGGCGT	AGCAACAGCT	ACTGGAACAA	AGTCTGCGAC	CATCAATTAT
N16	ATGCTGGCGT	AGTAACAGCT	GCTGGAACAA	AGTCTGCGAC	CATCAATTAT
AR39	ACGCTGGCGT	AGCAACAGCT	ACTGGAACAA	AGTCTGCGAC	CATCAATTAT
CWL029	ACGCTGGCGT	AGCAACAGCT	ACTGGAACAA	AGTCTGCGAC	CATCAATTAT
J138	ACGCTGGCGT	AGCAACAGCT	ACTGGAACAA	AGTCTGCGAC	CATCAATTAT
TW183	ACGCTGGCGT	AGCAACAGCT	ACTGGAACAA	AGTCTGCGAC	CATCAATTAT
IOL207	ACGCTGGCGT	AGCAACAGCT	ACTGGAACAA	AGTCTGCGAC	CATCAATTAT
LKK1	ACGCTGGCGT	AGCAACAGCT	ACTGGAACAA	AGTCTGCGAC	CATCAATTAT
TOR1	ACGCTGGCGT	AGCAACAGCT	ACTGGAACAA	AGTCTGCGAC	CATCAATTAT
WA97001	ACGCTGGCGT	AGCAACAGCT	ACTGGAACAA	AGTCTGCGAC	CATCAATTAT
1979	ACGCTGGCGT	AGCAACAGCT	ACTGGAACAA	AGTCTGCGAC	CATCAATTAT
SH511	ACGCTGGCGT	AGCAACAGCT	ACTGGAACAA	AGTCTGCGAC	CATCAATTAT

	51–60	61–70	71–80	81–90	91–100
Identity					
B26	CATGAATGGC	AAGTAGGAGC	CTCTCTATCT	TATAGACTAA	ACTCTTTAGT
B37	CATGAATGGC	AAGTAGGAGC	CTCTCTATCT	TATAGACTAA	ACTCTTTAGT
WBB	CATGAATGGC	AAGTAGGAGC	CTCTCTATCT	TATAGACTAA	ACTCTTTAGT
EBB	CATGAATGGC	AAGTAGGAGC	CTCTCTATCT	TATAGACTAA	ACTCTTTAGT
LPCoLN	CATGAATGGC	AAGTAGGAGC	CTCTCTATCT	TATAGACTAA	ACTCTTTAGT
Pot37	CATGAATGGC	AAGTAGGAGC	CTCTCTATCT	TATAGACTAA	ACTCTTTAGT
CPXT1	CATGAATGGC	AAGTAGGAGC	CTCTCTATCT	TACAGGCTAA	ACTCTTTAGT
DE177	CATGAATGGC	AAGTAGGAGC	CTCTCTATCT	TACAGACTAA	ACTCTTTAGT
GBF	CATGAATGGC	AAGTAGGAGC	CTCTCTATCT	TATAGACTAA	ACTCTTTAGT
BMTF-type1	CATGAATGGC	AAGTAGGAGC	CTCTCTATCT	TACAGACTAA	ACTCTTTAGT
Burpyth	CATGAATGGC	AAGTAGGAGC	CTCTCTATCT	TACAGACTAA	ACTCTTTAGT
PuffAdd	CATGAATGGC	AAGTAGGAGC	CTCTCTATCT	TACAGACTAA	ACTCTTTAGT
cham	CATGAATGGC	AAGTAGGAGC	CTCTCTATCT	TACAGACTAA	ACTCTTTAGT
Iguana	CATGAATGGC	AAGTAGGAGC	CTCTCTATCT	TACAGACTAA	ACTCTTTAGT
GST	CATGAATGGC	AAGTAGGAGC	CTCTCTATCT	TACAGACTAA	ACTCTTTAGT
N16	CATGAATGGC	AGGTAGGAGC	CTCTCTATCT	TATAGACTCA	ACTCTTTAGT
AR39	CATGAATGGC	AAGTAGGAGC	CTCTCTATCT	TACAGACTAA	ACTCTTTAGT
CWL029	CATGAATGGC	AAGTAGGAGC	CTCTCTATCT	TACAGACTAA	ACTCTTTAGT
J138	CATGAATGGC	AAGTAGGAGC	CTCTCTATCT	TACAGACTAA	ACTCTTTAGT
TW183	CATGAATGGC	AAGTAGGAGC	CTCTCTATCT	TACAGACTAA	ACTCTTTAGT
IOL207	CATGAATGGC	AAGTAGGAGC	CTCTCTATCT	TACAGACTAA	ACTCTTTAGT
LKK1	CATGAATGGC	AAGTAGGAGC	CTCTCTATCT	TACAGACTAA	ACTCTTTAGT
TOR1	CATGAATGGC	AAGTAGGAGC	CTCTCTATCT	TACAGACTAA	ACTCTTTAGT
WA97001	CATGAATGGC	AAGTAGGAGC	CTCTCTATCT	TACAGACTAA	ACTCTTTAGT
1979	CATGAATGGC	AAGTAGGAGC	CTCTCTATCT	TACAGACTAA	ACTCTTTAGT
SH511	CATGAATGGC	AAGTAGGAGC	CTCTCTATCT	TACAGACTAA	ACTCTTTAGT

	110	120	130	140	150
Identity					
B26	GCCATACATT	GGAGTACAAT	GGTCTCGAGC	AACTTTTGAT	GCTGATAACA
B37	GCCATACATT	GGAGTACAAT	GGTCTCGAGC	AACTTTTGAT	GCTGATAACA
WBB	GCCATACATT	GGAGTACAAT	GGTCTCGAGC	AACTTTTGAT	GCTGATAACA
EBB	GCCATACATT	GGAGTACAAT	GGTCTCGAGC	AACTTTTGAT	GCTGATAACA
LPCoLN	GCCATACATT	GGAGTACAAT	GGTCTCGAGC	AACTTTTGAT	GCTGATAACA
Pot37	GCCATACATT	GGAGTACAAT	GGTCTCGAGC	AACTTTTGAT	GCTGATAACA
CPXT1	GCCATACATT	GGAGTACAAT	GGTCTCGAGC	AACTTTTGAT	GCTGATAACA
DE177	GCCATACATT	GGAGTACAAT	GGTCTCGAGC	AACTTTTGAT	GCTGATAACA
GBF	GCCATACATT	GGAGTACAAT	GGTCTCGAGC	AACTTTTGAT	GCTGATAACA
BMTF-type1	GCCATACATT	GGAGTACAAT	GGTCTCGAGC	AACTTTTGAT	GCTGATAACA
Burpyth	GCCATACATT	GGAGTACAAT	GGTCTCGAGC	AACTTTTGAT	GCTGATAACA
PuffAdd	GCCATACATT	GGAGTACAAT	GGTCTCGAGC	AACTTTTGAT	GCTGATAACA
cham	GCCATACATT	GGAGTACAAT	GGTCTCGAGC	AACTTTTGAT	GCTGATAACA
Iguana	GCCATACATT	GGAGTACAAT	GGTCTCGAGC	AACTTTTGAT	GCTGATAACA
GST	GCCATACATT	GGAGTACAAT	GGTCTCGAGC	AACTTTTGAT	GCTGATAACA
N16	GCCATACATT	GGAGTCCAAT	GGTCTCGAGC	AACTTTTGAT	GCTGATAACA
AR39	GCCATACATT	GGAGTACAAT	GGTCTCGAGC	AACTTTTGAT	GCTGATAACA
CWL029	GCCATACATT	GGAGTACAAT	GGTCTCGAGC	AACTTTTGAT	GCTGATAACA
J138	GCCATACATT	GGAGTACAAT	GGTCTCGAGC	AACTTTTGAT	GCTGATAACA
TW183	GCCATACATT	GGAGTACAAT	GGTCTCGAGC	AACTTTTGAT	GCTGATAACA
IOL207	GCCATACATT	GGAGTACAAT	GGTCTCGAGC	AACTTTTGAT	GCTGATAACA
LKK1	GCCATACATT	GGAGTACAAT	GGTCTCGAGC	AACTTTTGAT	GCTGATAACA
TOR1	GCCATACATT	GGAGTACAAT	GGTCTCGAGC	AACTTTTGAT	GCTGATAACA
WA97001	GCCATACATT	GGAGTACAAT	GGTCTCGAGC	AACTTTTGAT	GCTGATAACA
1979	GCCATACATT	GGAGTACAAT	GGTCTCGAGC	AACTTTTGAT	GCTGATAACA
SH511	GCCATACATT	GGAGTACAAT	GGTCTCGAGC	AACTTTTGAT	GCTGATAACA

	160	170	180	190	200
Identity					
B26	TCCGCATTGC	TCAGCCAAAA	CTACCTACAG	CTGTTTTAAA	CTTAACTGCA
B37	TCCGCATTGC	TCAGCCAAAA	CTACCTACAG	CTGTTTTAAA	CTTAACTGCA
WBB	TCCGCATTGC	TCAGCCAAAA	CTACCTACAG	CTGTTTTAAA	CTTAACTGCA
EBB	TCCGCATTGC	TCAGCCAAAA	CTACCTACAG	CTGTTTTAAA	CTTAACTGCA
LPCoLN	TCCGCATTGC	TCAGCCAAAA	CTACCTACAG	CTGTTTTAAA	CTTAACTGCA
Pot37	TCCGCATTGC	TCAGCCAAAA	CTACCTACAG	CTGTTTTAAA	CTTAACTGCA
CPXT1	TCCGCATTGC	TCAGCCAAAA	CTACCTACAG	CTGTTTTAAA	CTTAACTGCA
DE177	TCCGCATTGC	TCAGCCAAAA	CTACCTACAG	CTGTTTTAAA	CTTAACTGCA
GBF	TCCGCATTGC	TCAGCCAAAA	CTACCTACAG	CTGTTTTAAA	CTTAACTGCA
BMTF-type1	TCCGCATTGC	TCAGCCAAAA	CTACCTACAG	CTGTTTTAAA	CTTAACTGCA
Burpyth	TCCGCATTGC	TCAGCCAAAA	CTACCTACAG	CTGTTTTAAA	CTTAACTGCA
PuffAdd	TCCGCATTGC	TCAGCCAAAA	CTACCTACAG	CTGTTTTAAA	CTTAACTGCA
cham	TCCGCATTGC	TCAGCCAAAA	CTACCTACAG	TTGTTTTAAA	CTTAACTGCA
Iguana	TCCGCATTGC	TCAGCCAAAA	CTACCTACAG	CTGTTTTAAA	CTTAACTGCA
GST	TCCGCATTGC	TCAGCCAAAA	CTACCTACAG	CTGTTTTAAA	CTTAACTGCA
N16	TCCGCATTGC	TCAGCCAAAG	CTACCTACAG	CTATTTTAAA	CTTAACTGCA
AR39	TCCGCATTGC	TCAGCCAAAA	CTACCTACAG	CTGTTTTAAA	CTTAACTGCA
CWL029	TCCGCATTGC	TCAGCCAAAA	CTACCTACAG	CTGTTTTAAA	CTTAACTGCA
J138	TCCGCATTGC	TCAGCCAAAA	CTACCTACAG	CTGTTTTAAA	CTTAACTGCA
TW183	TCCGCATTGC	TCAGCCAAAA	CTACCTACAG	CTGTTTTAAA	CTTAACTGCA
IOL207	TCCGCATTGC	TCAGCCAAAA	CTACCTACAG	CTGTTTTAAA	CTTAACTGCA
LKK1	TCCGCATTGC	TCAGCCAAAA	CTACCTACAG	CTGTTTTAAA	CTTAACTGCA
TOR1	TCCGCATTGC	TCAGCCAAAA	CTACCTACAG	CTGTTTTAAA	CTTAACTGCA
WA97001	TCCGCATTGC	TCAGCCAAAA	CTACCTACAG	CTGTTTTAAA	CTTAACTGCA
1979	TCCGCATTGC	TCAGCCAAAA	CTACCTACAG	CTGTTTTAAA	CTTAACTGCA
SH511	TCCGCATTGC	TCAGCCAAAA	CTACCTACAG	CTGTTTTAAA	CTTAACTGCA

210 220 230 240

		210	220	230	240
Identity					
B26	TGGAACCCTT	CTTTACTAGG	AAATACGACA	ACATTGCCTA	CTAGTGATTC
B37	TGGAACCCTT	CTTTACTAGG	AAATACGACA	ACATTGCCTA	CTAGTGATTC
WBB	TGGAACCCTT	CTTTACTAGG	AAATACGACA	ACATTGCCTA	CTAGTGATTC
EBB	TGGAACCCTT	CTTTACTAGG	AAATACGACA	ACATTGCCTA	CTAGTGATTC
LPCoLN	TGGAACCCTT	CTTTACTAGG	AAATACGACA	ACATTGCCTA	CTAGTGATTC
Pot37	TGGAACCCTT	CTTTACTAGG	AAATACGACA	ACATTGCCTA	CTAGTGATTC
CPXT1	TGGAACCCTT	CTTTACTAGG	AAATGCCACA	ACATTGTCTA	CTAATGATTC
DE177	TGGAACCCTT	CTTTACTAGG	AAATGCCACA	ACATTGCCTA	CTAATGATTC
GBF	TGGAACCCTT	CTTTACTAGG	AAATACGACA	ACATTGCCTA	CTAGTGATTC
BMTF-type1	TGGAACCCTT	CTTTACTAGG	AAATGCCACA	GCATTGTCTA	CTACTGATTC
Burpyth	TGGAACCCTT	CTTTACTAGG	AAATGCCACA	GCATTGTCTA	CTACTGATTC
PuffAdd	TGGAACCCTT	CTTTACTAGG	AAATGCCACA	GCATTGTCTA	CTACTGATTC
cham	TGGAACCCTT	CTTTACTAGG	AAATGCCACA	GCATTGTCTA	CTACTGATTC
Iguana	TGGAACCCTT	CTTTACTAGG	AAATGCCACA	GCATTGTCTA	CTACTGATTC
GST	TGGAACCCTT	CTTTACTAGG	AAATGCCACA	GCATTGTCTA	CTACTGATTC
N16	TGGAACCCTT	CTTTACTAGG	GAGTGCCACA	GCTGTTTCTT	CATCTGATCA
AR39	TGGAACCCTT	CTTTACTAGG	AAATGCCACA	GCATTGTCTA	CTACTGATTC
CWL029	TGGAACCCTT	CTTTACTAGG	AAATGCCACA	GCATTGTCTA	CTACTGATTC
J138	TGGAACCCTT	CTTTACTAGG	AAATGCCACA	GCATTGTCTA	CTACTGATTC
TW183	TGGAACCCTT	CTTTACTAGG	AAATGCCACA	GCATTGTCTA	CTACTGATTC
IOL207	TGGAACCCTT	CTTTACTAGG	AAATGCCACA	GCATTGTCTA	CTACTGATTC
LKK1	TGGAACCCTT	CTTTACTAGG	AAATGCCACA	GCATTGTCTA	CTACTGATTC
TOR1	TGGAACCCTT	CTTTACTAGG	AAATGCCACA	GCATTGTCTA	CTACTGATTC
WA97001	TGGAACCCTT	CTTTACTAGG	AAATGCCACA	GCATTGTCTA	CTACTGATTC
1979	TGGAACCCTT	CTTTACTAGG	AAATGCCACA	GCATTGTCTA	CTACTGATTC
SH511	TGGAACCCTT	CTTTACTAGG	AAATGCCACA	GCATTGTCTA	CTACTGATTC

	258
Identity	
B26	GTTCTCAG
B37	GTTCTCAG
WBB	GTTCTCAG
EBB	GTTCTCAG
LPCoLN	GTTCTCAG
Pot37	GTTCTCAG
CPXT1	GTTCTCAG
DE177	GTTCTCAG
GBF	GTTCTCAG
BMTF-type1	GTTCTCAG
Burpyth	GTTCTCAG
PuffAdd	GTTCTCAG
cham	GTTCTCAG
Iguana	GTTCTCAG
GST	GTTCTCAG
N16	ATTCTCAG
AR39	GTTCTCAG
CWL029	GTTCTCAG
J138	GTTCTCAG
TW183	GTTCTCAG
IOL207	GTTCTCAG
LKK1	GTTCTCAG
TOR1	GTTCTCAG
WA97001	GTTCTCAG
1979	GTTCTCAG
SH511	GTTCTCAG

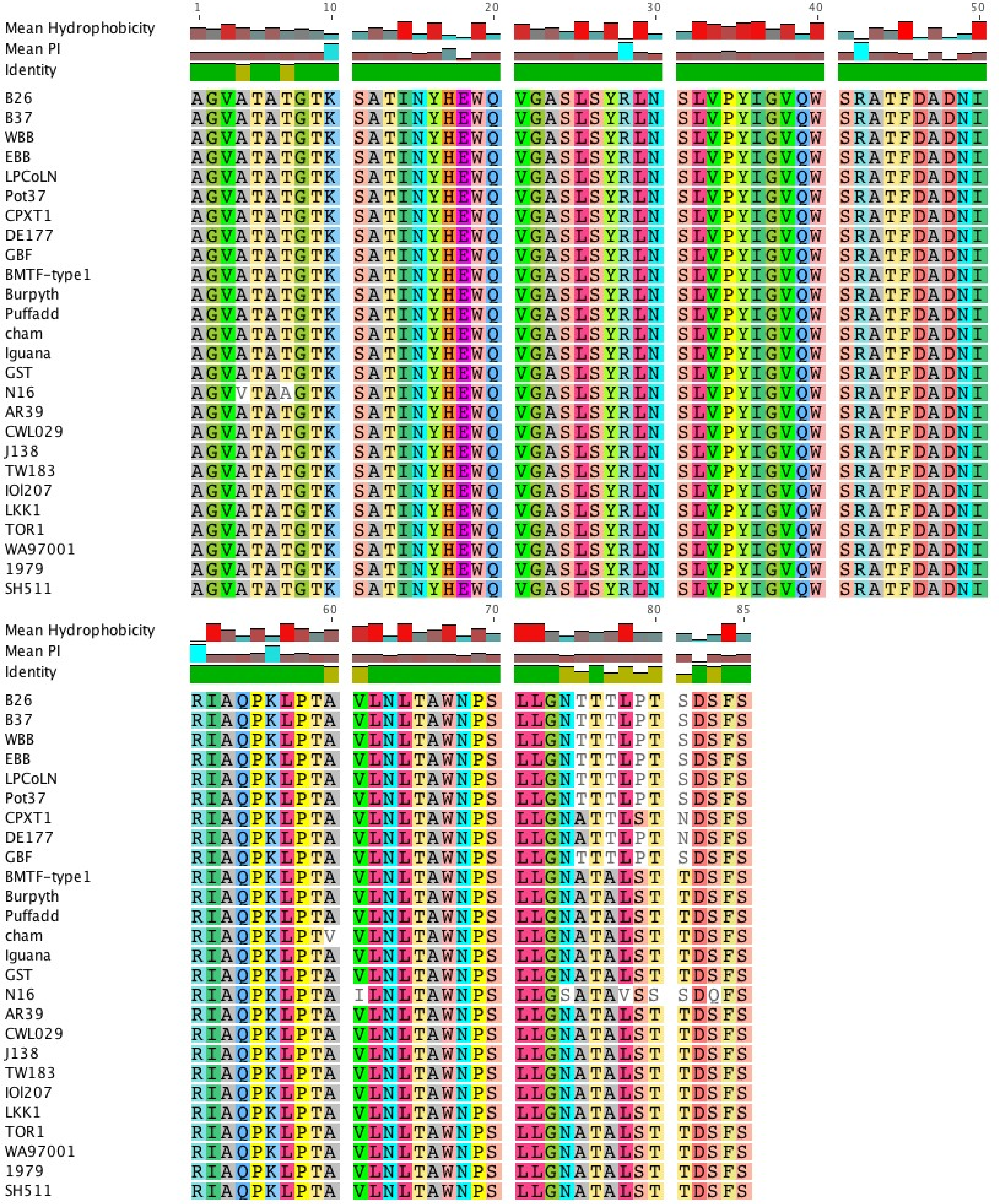

1 10 20 30 40 50
Mean Hydrophobicity
Mean PI
Identity
B26 AGVATATGTK SATINYHEWQ VGASLSYRLN SLVPYIGVQW SRATFDADNI
B37 AGVATATGTK SATINYHEWQ VGASLSYRLN SLVPYIGVQW SRATFDADNI
WBB AGVATATGTK SATINYHEWQ VGASLSYRLN SLVPYIGVQW SRATFDADNI
EBB AGVATATGTK SATINYHEWQ VGASLSYRLN SLVPYIGVQW SRATFDADNI
LPCoLN AGVATATGTK SATINYHEWQ VGASLSYRLN SLVPYIGVQW SRATFDADNI
Pot37 AGVATATGTK SATINYHEWQ VGASLSYRLN SLVPYIGVQW SRATFDADNI
CPXT1 AGVATATGTK SATINYHEWQ VGASLSYRLN SLVPYIGVQW SRATFDADNI
DE177 AGVATATGTK SATINYHEWQ VGASLSYRLN SLVPYIGVQW SRATFDADNI
GBF AGVATATGTK SATINYHEWQ VGASLSYRLN SLVPYIGVQW SRATFDADNI
BMTF-type1 AGVATATGTK SATINYHEWQ VGASLSYRLN SLVPYIGVQW SRATFDADNI
Burpyth AGVATATGTK SATINYHEWQ VGASLSYRLN SLVPYIGVQW SRATFDADNI
Puffadd AGVATATGTK SATINYHEWQ VGASLSYRLN SLVPYIGVQW SRATFDADNI
cham AGVATATGTK SATINYHEWQ VGASLSYRLN SLVPYIGVQW SRATFDADNI
Iguana AGVATATGTK SATINYHEWQ VGASLSYRLN SLVPYIGVQW SRATFDADNI
GST AGVATATGTK SATINYHEWQ VGASLSYRLN SLVPYIGVQW SRATFDADNI
N16 AGVVTAAGTK SATINYHEWQ VGASLSYRLN SLVPYIGVQW SRATFDADNI
AR39 AGVATATGTK SATINYHEWQ VGASLSYRLN SLVPYIGVQW SRATFDADNI
CWL029 AGVATATGTK SATINYHEWQ VGASLSYRLN SLVPYIGVQW SRATFDADNI
J138 AGVATATGTK SATINYHEWQ VGASLSYRLN SLVPYIGVQW SRATFDADNI
TW183 AGVATATGTK SATINYHEWQ VGASLSYRLN SLVPYIGVQW SRATFDADNI
IOl207 AGVATATGTK SATINYHEWQ VGASLSYRLN SLVPYIGVQW SRATFDADNI
LKK1 AGVATATGTK SATINYHEWQ VGASLSYRLN SLVPYIGVQW SRATFDADNI
TOR1 AGVATATGTK SATINYHEWQ VGASLSYRLN SLVPYIGVQW SRATFDADNI
WA97001 AGVATATGTK SATINYHEWQ VGASLSYRLN SLVPYIGVQW SRATFDADNI
1979 AGVATATGTK SATINYHEWQ VGASLSYRLN SLVPYIGVQW SRATFDADNI
SH511 AGVATATGTK SATINYHEWQ VGASLSYRLN SLVPYIGVQW SRATFDADNI
60 70 80 85
Mean Hydrophobicity
Mean PI
Identity
B26 RIAQPKLPTA VLNLTAWNPS LLGNTTTLPT SDSFS
B37 RIAQPKLPTA VLNLTAWNPS LLGNTTTLPT SDSFS
WBB RIAQPKLPTA VLNLTAWNPS LLGNTTTLPT SDSFS
EBB RIAQPKLPTA VLNLTAWNPS LLGNTTTLPT SDSFS
LPCoLN RIAQPKLPTA VLNLTAWNPS LLGNTTTLPT SDSFS
Pot37 RIAQPKLPTA VLNLTAWNPS LLGNTTTLPT SDSFS
CPXT1 RIAQPKLPTA VLNLTAWNPS LLGNATTLST NDSFS
DE177 RIAQPKLPTA VLNLTAWNPS LLGNATTLPT NDSFS
GBF RIAQPKLPTA VLNLTAWNPS LLGNTTTLPT SDSFS
BMTF-type1 RIAQPKLPTA VLNLTAWNPS LLGNATALST TDSFS
Burpyth RIAQPKLPTA VLNLTAWNPS LLGNATALST TDSFS
Puffadd RIAQPKLPTA VLNLTAWNPS LLGNATALST TDSFS
cham RIAQPKLPTV VLNLTAWNPS LLGNATALST TDSFS
Iguana RIAQPKLPTA VLNLTAWNPS LLGNATALST TDSFS
GST RIAQPKLPTA VLNLTAWNPS LLGNATALST TDSFS
N16 RIAQPKLPTA ILNLTAWNPS LLGSATAVSS SDQFS
AR39 RIAQPKLPTA VLNLTAWNPS LLGNATALST TDSFS
CWL029 RIAQPKLPTA VLNLTAWNPS LLGNATALST TDSFS
J138 RIAQPKLPTA VLNLTAWNPS LLGNATALST TDSFS
TW183 RIAQPKLPTA VLNLTAWNPS LLGNATALST TDSFS
IOl207 RIAQPKLPTA VLNLTAWNPS LLGNATALST TDSFS
LKK1 RIAQPKLPTA VLNLTAWNPS LLGNATALST TDSFS
TOR1 RIAQPKLPTA VLNLTAWNPS LLGNATALST TDSFS
WA97001 RIAQPKLPTA VLNLTAWNPS LLGNATALST TDSFS
1979 RIAQPKLPTA VLNLTAWNPS LLGNATALST TDSFS
SH511 RIAQPKLPTA VLNLTAWNPS LLGNATALST TDSFS
