# Supplementary material for: Chlamydia pneumoniae Is Genetically Diverse in Animals and Appears to Have Crossed the Host Barrier to Humans on (At Least) Two Occasions
Source: PLoS Pathog. 2010 May 20;6(5):e1000903. doi: 10.1371/journal.ppat.1000903 (PMC2873915; doi:10.1371/journal.ppat.1000903)
Supplement: Figure S9 — Multiple sequence alignment of omcB . The nucleotide and amino acid alignments were generated using Geneious version 4.7, where each nucleotide and amino acid is assigned its own colour. White shading indicates an amino acid variant. (0.94 MB PDF) [file ppat.1000903.s009.pdf]

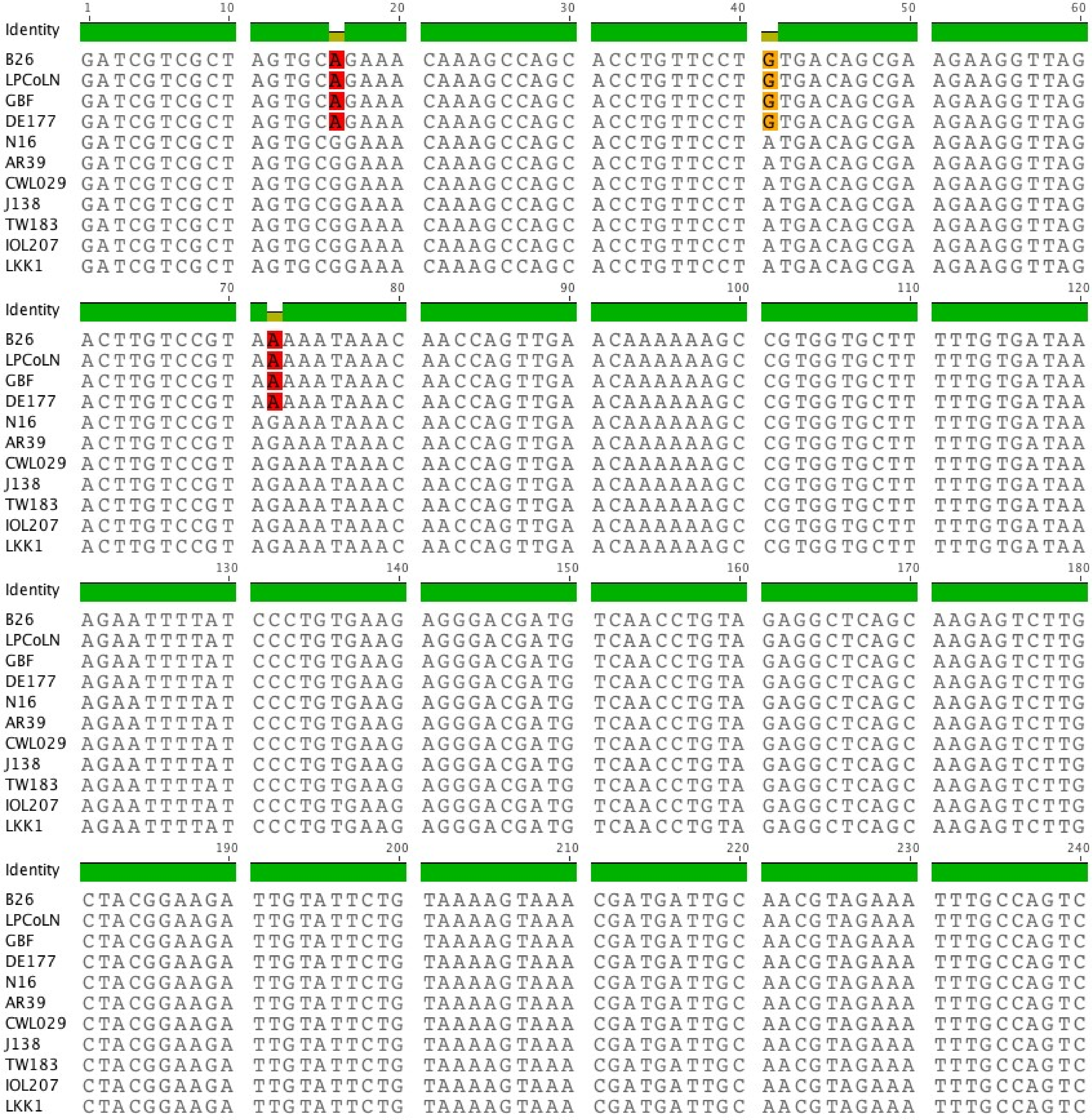
1 10 20 30 40 50 60
Identity
B26 GATCGTCGCT AGTGCAGAAA CAAAGCCAGC ACCTGTTCCT GTGACAGCGA AGAAGGTTAG
LPCoLN GATCGTCGCT AGTGCAGAAA CAAAGCCAGC ACCTGTTCCT GTGACAGCGA AGAAGGTTAG
GBF GATCGTCGCT AGTGCAGAAA CAAAGCCAGC ACCTGTTCCT GTGACAGCGA AGAAGGTTAG
DE177 GATCGTCGCT AGTGCAGAAA CAAAGCCAGC ACCTGTTCCT GTGACAGCGA AGAAGGTTAG
N16 GATCGTCGCT AGTGCGGAAA CAAAGCCAGC ACCTGTTCCT ATGACAGCGA AGAAGGTTAG
AR39 GATCGTCGCT AGTGCGGAAA CAAAGCCAGC ACCTGTTCCT ATGACAGCGA AGAAGGTTAG
CWL029 GATCGTCGCT AGTGCGGAAA CAAAGCCAGC ACCTGTTCCT ATGACAGCGA AGAAGGTTAG
J138 GATCGTCGCT AGTGCGGAAA CAAAGCCAGC ACCTGTTCCT ATGACAGCGA AGAAGGTTAG
TW183 GATCGTCGCT AGTGCGGAAA CAAAGCCAGC ACCTGTTCCT ATGACAGCGA AGAAGGTTAG
IOL207 GATCGTCGCT AGTGCGGAAA CAAAGCCAGC ACCTGTTCCT ATGACAGCGA AGAAGGTTAG
LKK1 GATCGTCGCT AGTGCGGAAA CAAAGCCAGC ACCTGTTCCT ATGACAGCGA AGAAGGTTAG
70 80 90 100 110 120
Identity
B26 ACTTGTCCGT AAAAATAAAC AACCAGTTGA ACAAAAAAGC CGTGGTGCTT TTTGTGATAA
LPCoLN ACTTGTCCGT AAAAATAAAC AACCAGTTGA ACAAAAAAGC CGTGGTGCTT TTTGTGATAA
GBF ACTTGTCCGT AAAAATAAAC AACCAGTTGA ACAAAAAAGC CGTGGTGCTT TTTGTGATAA
DE177 ACTTGTCCGT AAAAATAAAC AACCAGTTGA ACAAAAAAGC CGTGGTGCTT TTTGTGATAA
N16 ACTTGTCCGT AGAAATAAAC AACCAGTTGA ACAAAAAAGC CGTGGTGCTT TTTGTGATAA
AR39 ACTTGTCCGT AGAAATAAAC AACCAGTTGA ACAAAAAAGC CGTGGTGCTT TTTGTGATAA
CWL029 ACTTGTCCGT AGAAATAAAC AACCAGTTGA ACAAAAAAGC CGTGGTGCTT TTTGTGATAA
J138 ACTTGTCCGT AGAAATAAAC AACCAGTTGA ACAAAAAAGC CGTGGTGCTT TTTGTGATAA
TW183 ACTTGTCCGT AGAAATAAAC AACCAGTTGA ACAAAAAAGC CGTGGTGCTT TTTGTGATAA
IOL207 ACTTGTCCGT AGAAATAAAC AACCAGTTGA ACAAAAAAGC CGTGGTGCTT TTTGTGATAA
LKK1 ACTTGTCCGT AGAAATAAAC AACCAGTTGA ACAAAAAAGC CGTGGTGCTT TTTGTGATAA
130 140 150 160 170 180
Identity
B26 AGAATTTTAT CCCTGTGAAG AGGGACGATG TCAACCTGTA GAGGCTCAGC AAGAGTCTTG
LPCoLN AGAATTTTAT CCCTGTGAAG AGGGACGATG TCAACCTGTA GAGGCTCAGC AAGAGTCTTG
GBF AGAATTTTAT CCCTGTGAAG AGGGACGATG TCAACCTGTA GAGGCTCAGC AAGAGTCTTG
DE177 AGAATTTTAT CCCTGTGAAG AGGGACGATG TCAACCTGTA GAGGCTCAGC AAGAGTCTTG
N16 AGAATTTTAT CCCTGTGAAG AGGGACGATG TCAACCTGTA GAGGCTCAGC AAGAGTCTTG
AR39 AGAATTTTAT CCCTGTGAAG AGGGACGATG TCAACCTGTA GAGGCTCAGC AAGAGTCTTG
CWL029 AGAATTTTAT CCCTGTGAAG AGGGACGATG TCAACCTGTA GAGGCTCAGC AAGAGTCTTG
J138 AGAATTTTAT CCCTGTGAAG AGGGACGATG TCAACCTGTA GAGGCTCAGC AAGAGTCTTG
TW183 AGAATTTTAT CCCTGTGAAG AGGGACGATG TCAACCTGTA GAGGCTCAGC AAGAGTCTTG
IOL207 AGAATTTTAT CCCTGTGAAG AGGGACGATG TCAACCTGTA GAGGCTCAGC AAGAGTCTTG
LKK1 AGAATTTTAT CCCTGTGAAG AGGGACGATG TCAACCTGTA GAGGCTCAGC AAGAGTCTTG
190 200 210 220 230 240
Identity
B26 CTACGGAAGA TTGTATTCTG TAAAAGTAAA CGATGATTGC AACGTAGAAA TTTGCCAGTC
LPCoLN CTACGGAAGA TTGTATTCTG TAAAAGTAAA CGATGATTGC AACGTAGAAA TTTGCCAGTC
GBF CTACGGAAGA TTGTATTCTG TAAAAGTAAA CGATGATTGC AACGTAGAAA TTTGCCAGTC
DE177 CTACGGAAGA TTGTATTCTG TAAAAGTAAA CGATGATTGC AACGTAGAAA TTTGCCAGTC
N16 CTACGGAAGA TTGTATTCTG TAAAAGTAAA CGATGATTGC AACGTAGAAA TTTGCCAGTC
AR39 CTACGGAAGA TTGTATTCTG TAAAAGTAAA CGATGATTGC AACGTAGAAA TTTGCCAGTC
CWL029 CTACGGAAGA TTGTATTCTG TAAAAGTAAA CGATGATTGC AACGTAGAAA TTTGCCAGTC
J138 CTACGGAAGA TTGTATTCTG TAAAAGTAAA CGATGATTGC AACGTAGAAA TTTGCCAGTC
TW183 CTACGGAAGA TTGTATTCTG TAAAAGTAAA CGATGATTGC AACGTAGAAA TTTGCCAGTC
IOL207 CTACGGAAGA TTGTATTCTG TAAAAGTAAA CGATGATTGC AACGTAGAAA TTTGCCAGTC
LKK1 CTACGGAAGA TTGTATTCTG TAAAAGTAAA CGATGATTGC AACGTAGAAA TTTGCCAGTC

	250	260	270	280	290	300
B26	CGTTCCAGAA	TACGCTACTG	TAGGATCTCC	TTACCCTATT	GAAATCCTTG	CTATAGGCAA
LPCoLN	CGTTCCAGAA	TACGCTACTG	TAGGATCTCC	TTACCCTATT	GAAATCCTTG	CTATAGGCAA
GBF	CGTTCCAGAA	TACGCTACTG	TAGGATCTCC	TTACCCTATT	GAAATCCTTG	CTATAGGCAA
DE177	CGTTCCAGAA	TACGCTACTG	TAGGATCTCC	TTACCCTATT	GAAATCCTTG	CTATAGGCAA
N16	CGTTCCAGAA	TACGCTACTG	TAGGATCTCC	TTATCCTATT	GAAATCCTTG	CTATAGGCAA
AR39	CGTTCCAGAA	TACGCTACTG	TAGGATCTCC	TTACCCTATT	GAAATCCTTG	CTATAGGCAA
CWL029	CGTTCCAGAA	TACGCTACTG	TAGGATCTCC	TTACCCTATT	GAAATCCTTG	CTATAGGCAA
J138	CGTTCCAGAA	TACGCTACTG	TAGGATCTCC	TTACCCTATT	GAAATCCTTG	CTATAGGCAA
TW183	CGTTCCAGAA	TACGCTACTG	TAGGATCTCC	TTACCCTATT	GAAATCCTTG	CTATAGGCAA
IOL207	CGTTCCAGAA	TACGCTACTG	TAGGATCTCC	TTACCCTATT	GAAATCCTTG	CTATAGGCAA
LKK1	CGTTCCAGAA	TACGCTACTG	TAGGATCTCC	TTACCCTATT	GAAATCCTTG	CTATAGGCAA

	310	320	330	340	350	360
B26	AAAAGATTGT	GTTGATGTTG	TGATTACACA	ACAGCTACCT	TGCGAAGCTG	AATTCGTAAG
LPCoLN	AAAAGATTGT	GTTGATGTTG	TGATTACACA	ACAGCTACCT	TGCGAAGCTG	AATTCGTAAG
GBF	AAAAGATTGT	GTTGATGTTG	TGATTACACA	ACAGCTACCT	TGCGAAGCTG	AATTCGTAAG
DE177	AAAAGATTGT	GTTGATGTTG	TGATTACACA	ACAGCTACCT	TGCGAAGCTG	AATTCGTAAG
N16	AAAAGATTGT	GTTGATGTTG	TGATTACACA	ACAGCTACCT	TGCGAAGCTG	AATTCGTAAG
AR39	AAAAGATTGT	GTTGATGTTG	TGATTACACA	ACAGCTACCT	TGCGAAGCTG	AATTCGTAAG
CWL029	AAAAGATTGT	GTTGATGTTG	TGATTACACA	ACAGCTACCT	TGCGAAGCTG	AATTCGTAAG
J138	AAAAGATTGT	GTTGATGTTG	TGATTACACA	ACAGCTACCT	TGCGAAGCTG	AATTCGTAAG
TW183	AAAAGATTGT	GTTGATGTTG	TGATTACACA	ACAGCTACCT	TGCGAAGCTG	AATTCGTAAG
IOL207	AAAAGATTGT	GTTGATGTTG	TGATTACACA	ACAGCTACCT	TGCGAAGCTG	AATTCGTAAG
LKK1	AAAAGATTGT	GTTGATGTTG	TGATTACACA	ACAGCTACCT	TGCGAAGCTG	AATTCGTAAG

	370	380	386
B26	CAGTGATCCA	GAAACAACCC	CTACAA
LPCoLN	CAGTGATCCA	GAAACAACCC	CTACAA
GBF	CAGTGATCCA	GAAACAACCC	CTACAA
DE177	CAGTGATCCA	GAAACAACTC	CTACAA
N16	CAGTGATCCA	GAAACAACTC	CTACAA
AR39	CAGTGATCCA	GAAACAACTC	CTACAA
CWL029	CAGTGATCCA	GAAACAACTC	CTACAA
J138	CAGTGATCCA	GAAACAACTC	CTACAA
TW183	CAGTGATCCA	GAAACAACTC	CTACAA
IOL207	CAGTGATCCA	GAAACAACTC	CTACAA
LKK1	CAGTGATCCA	GAAACAACTC	CTACAA

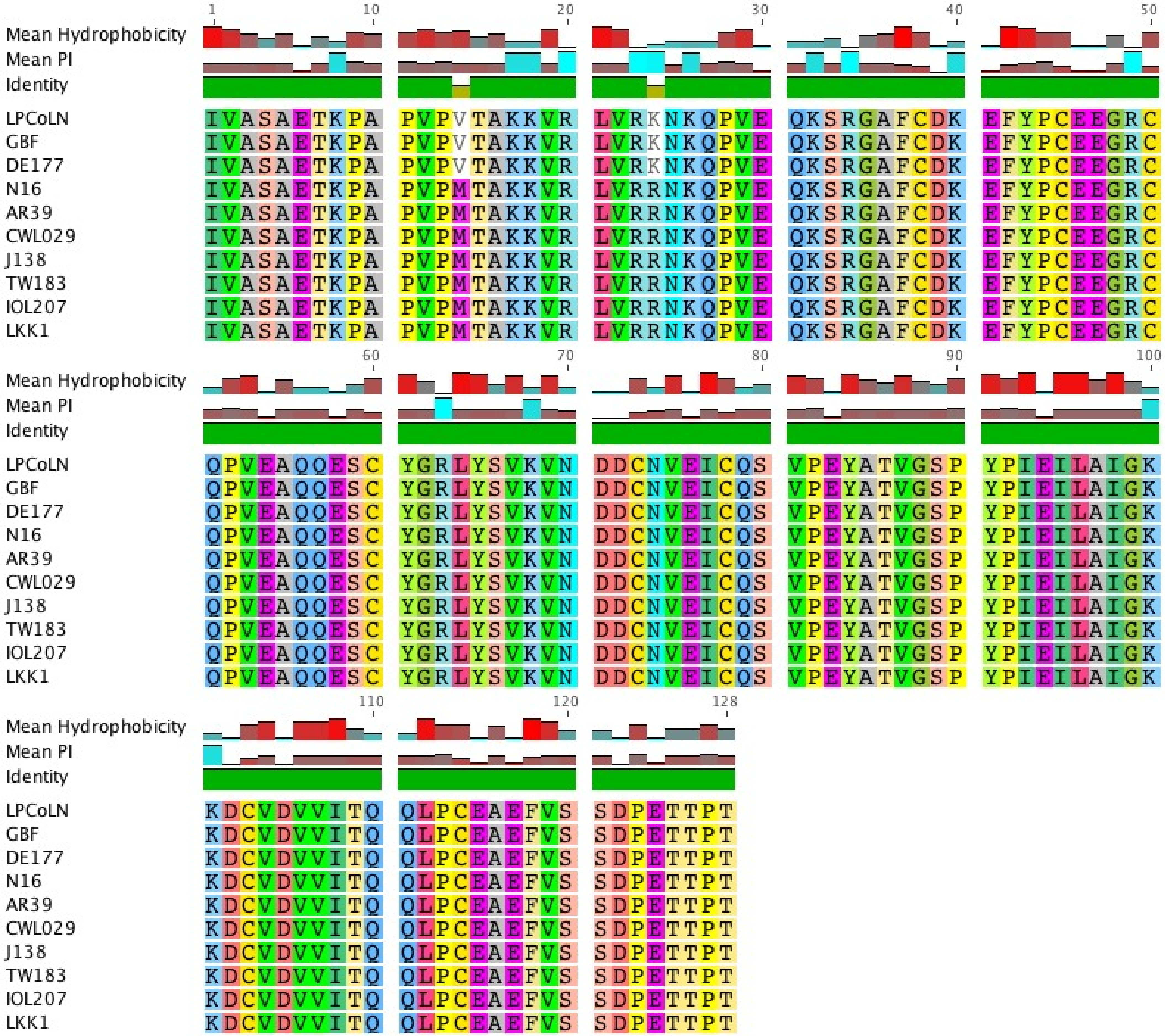

1
10
20
30
40
50
Mean Hydrophobicity
Mean PI
Identity
LPCoLN IVASAETKPA PVPVTAKKVR LVRKNKQPVE QKSRGAFCDK EFYPCEEGRC
GBF IVASAETKPA PVPVTAKKVR LVRKNKQPVE QKSRGAFCDK EFYPCEEGRC
DE177 IVASAETKPA PVPVTAKKVR LVRKNKQPVE QKSRGAFCDK EFYPCEEGRC
N16 IVASAETKPA PVPMTAKKVR LVRRNKQPVE QKSRGAFCDK EFYPCEEGRC
AR39 IVASAETKPA PVPMTAKKVR LVRRNKQPVE QKSRGAFCDK EFYPCEEGRC
CWL029 IVASAETKPA PVPMTAKKVR LVRRNKQPVE QKSRGAFCDK EFYPCEEGRC
J138 IVASAETKPA PVPMTAKKVR LVRRNKQPVE QKSRGAFCDK EFYPCEEGRC
TW183 IVASAETKPA PVPMTAKKVR LVRRNKQPVE QKSRGAFCDK EFYPCEEGRC
IOL207 IVASAETKPA PVPMTAKKVR LVRRNKQPVE QKSRGAFCDK EFYPCEEGRC
LKK1 IVASAETKPA PVPMTAKKVR LVRRNKQPVE QKSRGAFCDK EFYPCEEGRC
60
70
80
90
100
Mean Hydrophobicity
Mean PI
Identity
LPCoLN QPVEAQQESC YGRLYSVKVN DDCNVEICQS VPEYATVGSP YPIEILAIGK
GBF QPVEAQQESC YGRLYSVKVN DDCNVEICQS VPEYATVGSP YPIEILAIGK
DE177 QPVEAQQESC YGRLYSVKVN DDCNVEICQS VPEYATVGSP YPIEILAIGK
N16 QPVEAQQESC YGRLYSVKVN DDCNVEICQS VPEYATVGSP YPIEILAIGK
AR39 QPVEAQQESC YGRLYSVKVN DDCNVEICQS VPEYATVGSP YPIEILAIGK
CWL029 QPVEAQQESC YGRLYSVKVN DDCNVEICQS VPEYATVGSP YPIEILAIGK
J138 QPVEAQQESC YGRLYSVKVN DDCNVEICQS VPEYATVGSP YPIEILAIGK
TW183 QPVEAQQESC YGRLYSVKVN DDCNVEICQS VPEYATVGSP YPIEILAIGK
IOL207 QPVEAQQESC YGRLYSVKVN DDCNVEICQS VPEYATVGSP YPIEILAIGK
LKK1 QPVEAQQESC YGRLYSVKVN DDCNVEICQS VPEYATVGSP YPIEILAIGK
110
120
128
Mean Hydrophobicity
Mean PI
Identity
LPCoLN KDCVDVVITQ QLPCEAEFVS SDPETTPT
GBF KDCVDVVITQ QLPCEAEFVS SDPETTPT
DE177 KDCVDVVITQ QLPCEAEFVS SDPETTPT
N16 KDCVDVVITQ QLPCEAEFVS SDPETTPT
AR39 KDCVDVVITQ QLPCEAEFVS SDPETTPT
CWL029 KDCVDVVITQ QLPCEAEFVS SDPETTPT
J138 KDCVDVVITQ QLPCEAEFVS SDPETTPT
TW183 KDCVDVVITQ QLPCEAEFVS SDPETTPT
IOL207 KDCVDVVITQ QLPCEAEFVS SDPETTPT
LKK1 KDCVDVVITQ QLPCEAEFVS SDPETTPT
