# Supplementary material for: Chlamydia pneumoniae Is Genetically Diverse in Animals and Appears to Have Crossed the Host Barrier to Humans on (At Least) Two Occasions
Source: PLoS Pathog. 2010 May 20;6(5):e1000903. doi: 10.1371/journal.ppat.1000903 (PMC2873915; doi:10.1371/journal.ppat.1000903)
Supplement: Figure S10 — Multiple sequence alignment of accC . The nucleotide and amino acid alignments were generated using Geneious version 4.7, where each nucleotide and amino acid is assigned its own colour. White shading indicates an amino acid variant. (0.71 MB PDF) [file ppat.1000903.s010.pdf]

	1–10	20	30	40	50
Identity					
B26	TGTTTATATT	GAAAAGTTTA	TAGAAAATCC	ACGGCATTTA	GAAATCCAAG
B37	TGTTTATATT	GAAAAGTTTA	TAGAAAATCC	ACGGCATTTA	GAAATCCAAG
EBB	TGTTTATATT	GAAAAGTTTA	TAGAAAATCC	ACGGCATTTA	GAAATCCAAG
LPCoLN	TGTTTATATT	GAAAAGTTTA	TAGAAAATCC	ACGGCATTTA	GAAATCCAAG
Pot37	TGTTTATATT	GAAAAGTTTA	TAGAAAATCC	ACGGCATTTA	GAAATCCAAG
2040.3	TGTTTATATT	GAAAAGTTTA	TAGAAAATCC	ACGGCATTTA	GAAATCCAAG
GBF	TGTTTATATT	GAAAAGTTTA	TAGAAAATCC	ACGGCATTTA	GAAATCCAAG
DE177	TGTTTATATT	GAAAAGTTTA	TAGAAAATCC	ACGGCATTTA	GAAATCCAAG
N16	TGTTTATATT	GAAAAGTTTA	TAGAAAATCC	ACGGCATTTA	GAAATCCAAG
AR39	TGTTTATATT	GAAAAGTTTA	TAGAAAATCC	AAGGCATTTA	GAAATCCAAG
CWL029	TGTTTATATT	GAAAAGTTTA	TAGAAAATCC	AAGGCATTTA	GAAATCCAAG
J138	TGTTTATATT	GAAAAGTTTA	TAGAAAATCC	AAGGCATTTA	GAAATCCAAG
TW183	TGTTTATATT	GAAAAGTTTA	TAGAAAATCC	AAGGCATTTA	GAAATCCAAG
A03	TGTTTATATT	GAAAAGTTTA	TAGAAAATCC	AAGGCATTTA	GAAATCCAAG
1979	TGTTTATATT	GAAAAGTTTA	TAGAAAATCC	ACGGCATTTA	GAAATCCAAG
SH511	TGTTTATATT	GAAAAGTTTA	TAGAAAATCC	ACGGCATTTA	GAAATCCAAG

	60	70	80	90	100
Identity					
B26	TCATTGGGGA	TACCCATGGA	AATTATGTGC	ATTTAGGAGA	AAGAGACTGC
B37	TCATTGGGGA	TACCCATGGA	AATTATGTGC	ATTTAGGAGA	AAGAGACTGC
EBB	TCATTGGGGA	TACCCATGGA	AATTATGTGC	ATTTAGGAGA	AAGAGACTGC
LPCoLN	TCATTGGGGA	TACCCATGGA	AATTATGTGC	ATTTAGGAGA	AAGAGACTGC
Pot37	TCATTGGGGA	TACCCATGGA	AATTATGTGC	ATTTAGGAGA	AAGAGACTGC
2040.3	TCATTGGGGA	TACCCATGGA	AATTATGTGC	ATTTAGGAGA	AAGAGACTGC
GBF	TCATTGGGGA	TACCCATGGA	AATTATGTGC	ATTTAGGAGA	AAGAGACTGC
DE177	TCATTGGGGA	TACCCATGGA	AATTATGTGC	ATTTAGGAGA	AAGAGACTGC
N16	TCATTGGGGA	TACCCATGGA	AATTATGTGC	ATTTAGGAGA	AAGAGACTGC
AR39	TCATTGGGGA	TACCCATGGA	AATTATGTGC	ATTTAGGAGA	AAGAGACTGC
CWL029	TCATTGGGGA	TACCCATGGA	AATTATGTGC	ATTTAGGAGA	AAGAGACTGC
J138	TCATTGGGGA	TACCCATGGA	AATTATGTGC	ATTTAGGAGA	AAGAGACTGC
TW183	TCATTGGGGA	TACCCATGGA	AATTATGTGC	ATTTAGGAGA	AAGAGACTGC
A03	TCATTGGGGA	TACCCATGGA	AATTATGTGC	ATTTAGGAGA	AAGAGACTGC
1979	TCATTGGGGA	TACCCATGGA	AATTATGTGC	ATTTAGGAGA	AAGAGACTGC
SH511	TCATTGGGGA	TACCCATGGA	AATTATGTGC	ATTTAGGAGA	AAGAGACTGC

	110	120	130	140	150
Identity					
B26	ACCATTCAAC	GGCGACGTCA	AAAGTTGATT	GAAGAGACTC	CTAGTCCCAT
B37	ACCATTCAAC	GGCGACGTCA	AAAGTTGATT	GAAGAGACTC	CTAGTCCCAT
EBB	ACCATTCAAC	GGCGACGTCA	AAAGTTGATT	GAAGAGACTC	CTAGTCCCAT
LPCoLN	ACCATTCAAC	GGCGACGTCA	AAAGTTGATT	GAAGAGACTC	CTAGTCCCAT
Pot37	ACCATTCAAC	GGCGACGTCA	AAAGTTGATT	GAAGAGACTC	CTAGTCCCAT
2040.3	ACCATTCAAC	GGCGACGTCA	AAAGTTGATT	GAAGAGACTC	CTAGTCCCAT
GBF	ACCATTCAAC	GGCGACGTCA	AAAGTTGATT	GAAGAGACTC	CTAGTCCCAT
DE177	ACCATTCAAC	GGCGACGTCA	AAAGTTGATT	GAAGAGACTC	CTAGTCCCAT
N16	ACCATTCAAC	GGCGACGTCA	AAAGTTGATT	GAAGAGACTC	CTAGTCCCAT
AR39	ACCATTCAAC	GGCGACGTCA	AAAGTTGATT	GAAGAGACTC	CTAGTCCCAT
CWL029	ACCATTCAAC	GGCGACGTCA	AAAGTTGATT	GAAGAGACTC	CTAGTCCCAT
J138	ACCATTCAAC	GGCGACGTCA	AAAGTTGATT	GAAGAGACTC	CTAGTCCCAT
TW183	ACCATTCAAC	GGCGACGTCA	AAAGTTGATT	GAAGAGACTC	CTAGTCCCAT
A03	ACCATTCAAC	GGCGACGTCA	AAAGTTGATT	GAAGAGACTC	CTAGTCCCAT
1979	ACCATTCAAC	GGCGACGTCA	AAAGTTGATT	GAAGAGACTC	CTAGTCCCAT
SH511	ACCATTCAAC	GGCGACGTCA	AAAGTTGATT	GAAGAGACTC	CTAGTCCCAT

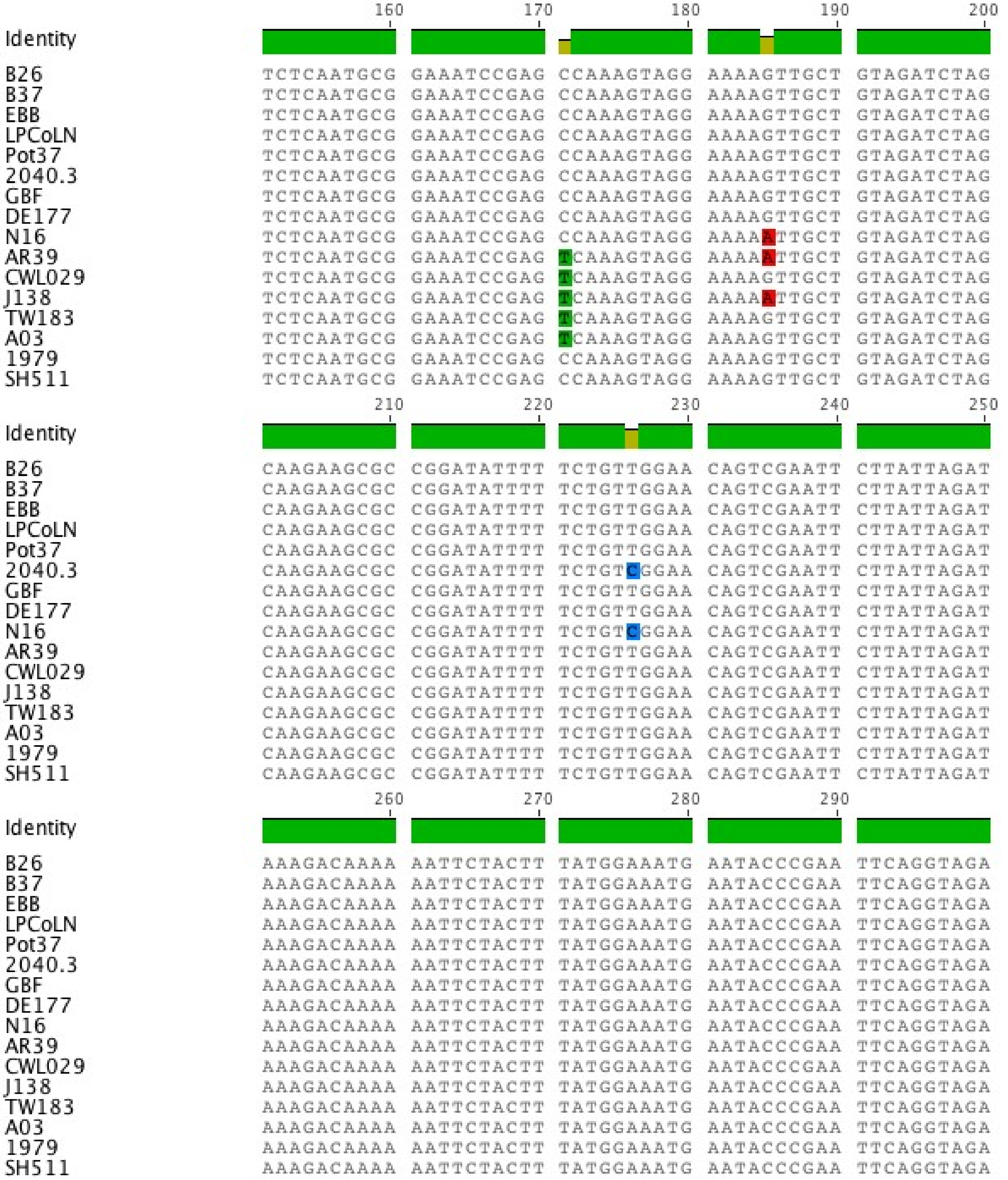

160 170 180 190 200
Identity
B26 TCTCAATGCG GAAATCCGAG CCAAAGTAGG AAAAGTTGCT GTAGATCTAG
B37 TCTCAATGCG GAAATCCGAG CCAAAGTAGG AAAAGTTGCT GTAGATCTAG
EBB TCTCAATGCG GAAATCCGAG CCAAAGTAGG AAAAGTTGCT GTAGATCTAG
LPCoLN TCTCAATGCG GAAATCCGAG CCAAAGTAGG AAAAGTTGCT GTAGATCTAG
Pot37 TCTCAATGCG GAAATCCGAG CCAAAGTAGG AAAAGTTGCT GTAGATCTAG
2040.3 TCTCAATGCG GAAATCCGAG CCAAAGTAGG AAAAGTTGCT GTAGATCTAG
GBF TCTCAATGCG GAAATCCGAG CCAAAGTAGG AAAAGTTGCT GTAGATCTAG
DE177 TCTCAATGCG GAAATCCGAG CCAAAGTAGG AAAAGTTGCT GTAGATCTAG
N16 TCTCAATGCG GAAATCCGAG CCAAAGTAGG AAAAATTGCT GTAGATCTAG
AR39 TCTCAATGCG GAAATCCGAG TCAAAGTAGG AAAAATTGCT GTAGATCTAG
CWL029 TCTCAATGCG GAAATCCGAG TCAAAGTAGG AAAAGTTGCT GTAGATCTAG
J138 TCTCAATGCG GAAATCCGAG TCAAAGTAGG AAAAATTGCT GTAGATCTAG
TW183 TCTCAATGCG GAAATCCGAG TCAAAGTAGG AAAAGTTGCT GTAGATCTAG
A03 TCTCAATGCG GAAATCCGAG TCAAAGTAGG AAAAGTTGCT GTAGATCTAG
1979 TCTCAATGCG GAAATCCGAG CCAAAGTAGG AAAAGTTGCT GTAGATCTAG
SH511 TCTCAATGCG GAAATCCGAG CCAAAGTAGG AAAAGTTGCT GTAGATCTAG
210 220 230 240 250
Identity
B26 CAAGAAGCGC CGGATATTTT TCTGTTGGAA CAGTCGAATT CTTATTAGAT
B37 CAAGAAGCGC CGGATATTTT TCTGTTGGAA CAGTCGAATT CTTATTAGAT
EBB CAAGAAGCGC CGGATATTTT TCTGTTGGAA CAGTCGAATT CTTATTAGAT
LPCoLN CAAGAAGCGC CGGATATTTT TCTGTTGGAA CAGTCGAATT CTTATTAGAT
Pot37 CAAGAAGCGC CGGATATTTT TCTGTTGGAA CAGTCGAATT CTTATTAGAT
2040.3 CAAGAAGCGC CGGATATTTT TCTGTCGGAA CAGTCGAATT CTTATTAGAT
GBF CAAGAAGCGC CGGATATTTT TCTGTTGGAA CAGTCGAATT CTTATTAGAT
DE177 CAAGAAGCGC CGGATATTTT TCTGTTGGAA CAGTCGAATT CTTATTAGAT
N16 CAAGAAGCGC CGGATATTTT TCTGTCGGAA CAGTCGAATT CTTATTAGAT
AR39 CAAGAAGCGC CGGATATTTT TCTGTTGGAA CAGTCGAATT CTTATTAGAT
CWL029 CAAGAAGCGC CGGATATTTT TCTGTTGGAA CAGTCGAATT CTTATTAGAT
J138 CAAGAAGCGC CGGATATTTT TCTGTTGGAA CAGTCGAATT CTTATTAGAT
TW183 CAAGAAGCGC CGGATATTTT TCTGTTGGAA CAGTCGAATT CTTATTAGAT
A03 CAAGAAGCGC CGGATATTTT TCTGTTGGAA CAGTCGAATT CTTATTAGAT
1979 CAAGAAGCGC CGGATATTTT TCTGTTGGAA CAGTCGAATT CTTATTAGAT
SH511 CAAGAAGCGC CGGATATTTT TCTGTTGGAA CAGTCGAATT CTTATTAGAT
260 270 280 290
Identity
B26 AAAGACAAAA AATTCTACTT TATGGAAATG AATACCCGAA TTCAGGTAGA
B37 AAAGACAAAA AATTCTACTT TATGGAAATG AATACCCGAA TTCAGGTAGA
EBB AAAGACAAAA AATTCTACTT TATGGAAATG AATACCCGAA TTCAGGTAGA
LPCoLN AAAGACAAAA AATTCTACTT TATGGAAATG AATACCCGAA TTCAGGTAGA
Pot37 AAAGACAAAA AATTCTACTT TATGGAAATG AATACCCGAA TTCAGGTAGA
2040.3 AAAGACAAAA AATTCTACTT TATGGAAATG AATACCCGAA TTCAGGTAGA
GBF AAAGACAAAA AATTCTACTT TATGGAAATG AATACCCGAA TTCAGGTAGA
DE177 AAAGACAAAA AATTCTACTT TATGGAAATG AATACCCGAA TTCAGGTAGA
N16 AAAGACAAAA AATTCTACTT TATGGAAATG AATACCCGAA TTCAGGTAGA
AR39 AAAGACAAAA AATTCTACTT TATGGAAATG AATACCCGAA TTCAGGTAGA
CWL029 AAAGACAAAA AATTCTACTT TATGGAAATG AATACCCGAA TTCAGGTAGA
J138 AAAGACAAAA AATTCTACTT TATGGAAATG AATACCCGAA TTCAGGTAGA
TW183 AAAGACAAAA AATTCTACTT TATGGAAATG AATACCCGAA TTCAGGTAGA
A03 AAAGACAAAA AATTCTACTT TATGGAAATG AATACCCGAA TTCAGGTAGA
1979 AAAGACAAAA AATTCTACTT TATGGAAATG AATACCCGAA TTCAGGTAGA
SH511 AAAGACAAAA AATTCTACTT TATGGAAATG AATACCCGAA TTCAGGTAGA

	302
Identity	
B26	GC
B37	GC
EBB	GC
LPCoLN	GC
Pot37	GC
2040.3	GC
GBF	GC
DE177	GC
N16	GC
AR39	GC
CWL029	GC
J138	GC
TW183	GC
A03	GC
1979	GC
SH511	GC

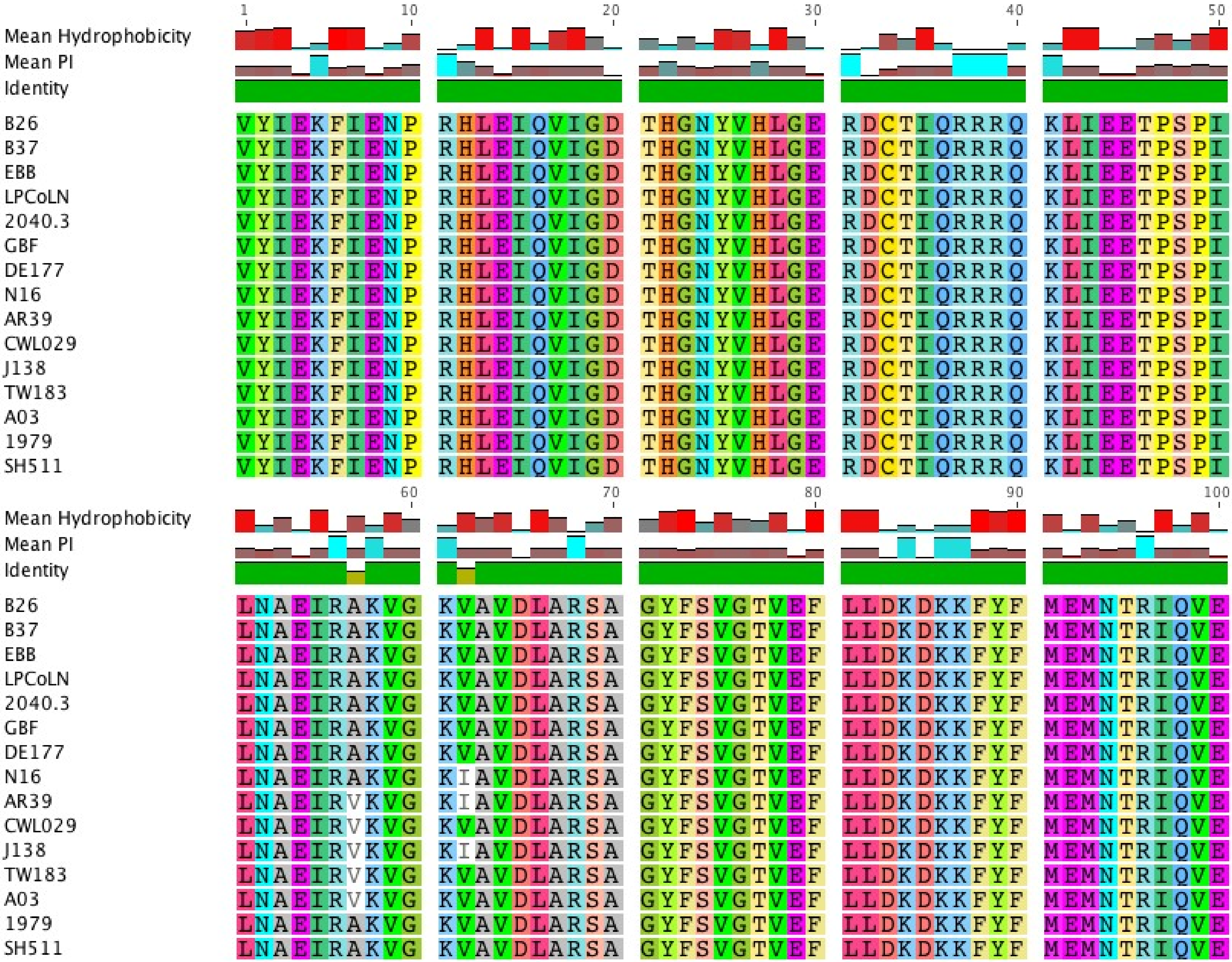
1 10 20 30 40 50
Mean Hydrophobicity
Mean PI
Identity
B26 VYIEKFIENP RHLEIQVIGD THGNYVHLGE RDCTIQRRRQ KLIEETPSPI
B37 VYIEKFIENP RHLEIQVIGD THGNYVHLGE RDCTIQRRRQ KLIEETPSPI
EBB VYIEKFIENP RHLEIQVIGD THGNYVHLGE RDCTIQRRRQ KLIEETPSPI
LPCoLN VYIEKFIENP RHLEIQVIGD THGNYVHLGE RDCTIQRRRQ KLIEETPSPI
2040.3 VYIEKFIENP RHLEIQVIGD THGNYVHLGE RDCTIQRRRQ KLIEETPSPI
GBF VYIEKFIENP RHLEIQVIGD THGNYVHLGE RDCTIQRRRQ KLIEETPSPI
DE177 VYIEKFIENP RHLEIQVIGD THGNYVHLGE RDCTIQRRRQ KLIEETPSPI
N16 VYIEKFIENP RHLEIQVIGD THGNYVHLGE RDCTIQRRRQ KLIEETPSPI
AR39 VYIEKFIENP RHLEIQVIGD THGNYVHLGE RDCTIQRRRQ KLIEETPSPI
CWL029 VYIEKFIENP RHLEIQVIGD THGNYVHLGE RDCTIQRRRQ KLIEETPSPI
J138 VYIEKFIENP RHLEIQVIGD THGNYVHLGE RDCTIQRRRQ KLIEETPSPI
TW183 VYIEKFIENP RHLEIQVIGD THGNYVHLGE RDCTIQRRRQ KLIEETPSPI
A03 VYIEKFIENP RHLEIQVIGD THGNYVHLGE RDCTIQRRRQ KLIEETPSPI
1979 VYIEKFIENP RHLEIQVIGD THGNYVHLGE RDCTIQRRRQ KLIEETPSPI
SH511 VYIEKFIENP RHLEIQVIGD THGNYVHLGE RDCTIQRRRQ KLIEETPSPI
60 70 80 90 100
Mean Hydrophobicity
Mean PI
Identity
B26 LNAEIRAKVG KVAVDLARSA GYFSVGTVEF LLDKDKKFYF MEMNTRIQVE
B37 LNAEIRAKVG KVAVDLARSA GYFSVGTVEF LLDKDKKFYF MEMNTRIQVE
EBB LNAEIRAKVG KVAVDLARSA GYFSVGTVEF LLDKDKKFYF MEMNTRIQVE
LPCoLN LNAEIRAKVG KVAVDLARSA GYFSVGTVEF LLDKDKKFYF MEMNTRIQVE
2040.3 LNAEIRAKVG KVAVDLARSA GYFSVGTVEF LLDKDKKFYF MEMNTRIQVE
GBF LNAEIRAKVG KVAVDLARSA GYFSVGTVEF LLDKDKKFYF MEMNTRIQVE
DE177 LNAEIRAKVG KVAVDLARSA GYFSVGTVEF LLDKDKKFYF MEMNTRIQVE
N16 LNAEIRAKVG KIAVDLARSA GYFSVGTVEF LLDKDKKFYF MEMNTRIQVE
AR39 LNAEIRVKVG KIAVDLARSA GYFSVGTVEF LLDKDKKFYF MEMNTRIQVE
CWL029 LNAEIRVKVG KVAVDLARSA GYFSVGTVEF LLDKDKKFYF MEMNTRIQVE
J138 LNAEIRVKVG KIAVDLARSA GYFSVGTVEF LLDKDKKFYF MEMNTRIQVE
TW183 LNAEIRVKVG KVAVDLARSA GYFSVGTVEF LLDKDKKFYF MEMNTRIQVE
A03 LNAEIRVKVG KVAVDLARSA GYFSVGTVEF LLDKDKKFYF MEMNTRIQVE
1979 LNAEIRAKVG KVAVDLARSA GYFSVGTVEF LLDKDKKFYF MEMNTRIQVE
SH511 LNAEIRAKVG KVAVDLARSA GYFSVGTVEF LLDKDKKFYF MEMNTRIQVE
