# Supplementary material for: Chlamydia pneumoniae Is Genetically Diverse in Animals and Appears to Have Crossed the Host Barrier to Humans on (At Least) Two Occasions
Source: PLoS Pathog. 2010 May 20;6(5):e1000903. doi: 10.1371/journal.ppat.1000903 (PMC2873915; doi:10.1371/journal.ppat.1000903)
Supplement: Figure S11 — Multiple sequence alignment of pfk . The nucleotide and amino acid alignments were generated using Geneious version 4.7, where each nucleotide and amino acid is assigned its own colour. White shading indicates an amino acid variant. (0.96 MB PDF) [file ppat.1000903.s011.pdf]

	1–10	11–20	21–30	31–40	41–50
Identity					
B26	CCAATCTATG	TCGGATCCCT	GAAGTTTCTA	TTTATACTGA	ACAGGAAACG
B37	CCAATCTATG	TCGGATCCCT	GAAGTTTCTA	TTTATACTGA	ACAGGAAACG
EBB	CCAATCTATG	TCGGATCCCT	GAAGTTTCTA	TTTATACTGA	ACAGGAAACG
LPCoLN	CCAATCTATG	TCGGATCCCT	GAAGTTTCTA	TTTATACTGA	ACAGGAAACG
Pot37	CCAATCTATG	TCGGATCCCT	GAAGTTTCTA	TTTATACTGA	ACAGGAAACG
GBF	CCAATCTATG	TCGGATCCCT	GAAGTTTCTA	TTTATACTGA	ACAGGAAACG
DE177	CCAATCTATG	TCGGATCCCT	GAAGTTTCTA	TTTATACTGA	ACAGGAAACG
N16	CCAATCTATG	TCGGATCCCT	GAAGTTTCTA	TTTATACTGA	ACAGGAAACG
AR39	CCAATCTATG	TCGGATCCCT	GAAGTTTCTA	TTTATACTGA	ACAGGAAACG
CWL029	CCAATCTATG	TCGGATCCCT	GAAGTTTCTA	TTTATACTGA	ACAGGAAACG
J138	CCAATCTATG	TCGGATCCCT	GAAGTTTCTA	TTTATACTGA	ACAGGAAACG
TW183	CCAATCTATG	TCGGATCCCT	GAAGTTTCTA	TTTATACTGA	ACAGGAAACG
IOL207	CCAATCTATG	TCGGATCCCT	GAAGTTTCTA	TTTATACTGA	ACAGGAAACG
A03	CCAATCTATG	TCGGATCCCT	GAAGTTTCTA	TTTATACTGA	ACAGGAAACG
WA97001	CCAATCTATG	TCGGATCCCT	GAAGTTTCTA	TTTATACTGA	ACAGGAAACG
SH511	CCAATCTATG	TCGGATCCCT	GAAGTTTCTA	TTTATACTGA	ACAGGAAACG
1979	CCAATCTATG	TCGGATCCCT	GAAGTTTCTA	TTTATACTGA	ACAGGAAACG

	51–60	61–70	71–80	81–90	91–100
Identity					
B26	TCATCAAAAC	CATTAAAGAT	TGGGGTTTTA	CTATCAGGAG	GACAAGCTCC
B37	TCATCAAAAC	CATTAAAGAT	TGGGGTTTTA	CTATCAGGAG	GACAAGCTCC
EBB	TCATCAAAAC	CATTAAAGAT	TGGGGTTTTA	CTATCAGGAG	GACAAGCTCC
LPCoLN	TCATCAAAAC	CATTAAAGAT	TGGGGTTTTA	CTATCAGGAG	GACAAGCTCC
Pot37	TCATCAAAAC	CATTAAAGAT	TGGGGTTTTA	CTATCAGGAG	GACAAGCTCC
GBF	TCATCAAAAC	CATTAAAGAT	TGGGGTTTTA	CTATCAGGAG	GACAAGCTCC
DE177	TCATCAAAAC	CATTAAAGAT	TGGGGTTTTA	CTATCAGGAG	GACAAGCTCC
N16	TCATCAAAAC	CATTAAAGAT	TGGGGTTTTA	CTATCAGGAG	GACAAGCTCC
AR39	TCATCAAAAC	CATTAAAGAT	TGGGGTTTTA	CTATCAGGAG	GACAAGCTCC
CWL029	TCATCAAAAC	CATTAAAGAT	TGGGGTTTTA	CTATCAGGAG	GACAAGCTCC
J138	TCATCAAAAC	CATTAAAGAT	TGGGGTTTTA	CTATCAGGAG	GACAAGCTCC
TW183	TCATCAAAAC	CATTAAAGAT	TGGGGTTTTA	CTATCAGGAG	GACAAGCTCC
IOL207	TCATCAAAAC	CATTAAAGAT	TGGGGTTTTA	CTATCAGGAG	GACAAGCTCC
A03	TCATCAAAAC	CATTAAAGAT	TGGGGTTTTA	CTATCAGGAG	GACAAGCTCC
WA97001	TCATCAAAAC	CATTAAAGAT	TGGGGTTTTA	CTATCAGGAG	GACAAGCTCC
SH511	TCATCAAAAC	CATTAAAGAT	TGGGGTTTTA	CTATCAGGAG	GACAAGCTCC
1979	TCATCAAAAC	CATTAAAGAT	TGGGGTTTTA	CTATCAGGAG	GACAAGCTCC

	101–110	111–120	121–130	131–140	141–150
Identity					
B26	TGGGGGGCAC	AATGTCGTTA	TTGGTCTTTT	TGATGCTTTA	CGAGTATTCA
B37	TGGGGGGCAC	AATGTCGTTA	TTGGTCTTTT	TGATGCTTTA	CGAGTATTCA
EBB	TGGGGGGCAC	AATGTCGTTA	TTGGTCTTTT	TGATGCTTTA	CGAGTATTCA
LPCoLN	TGGGGGGCAC	AATGTCGTTA	TTGGTCTTTT	TGATGCTTTA	CGAGTATTCA
Pot37	TGGGGGGCAC	AATGTCGTTA	TTGGTCTTTT	TGATGCTTTA	CGAGTATTCA
GBF	TGGGGGGCAC	AATGTCGTTA	TTGGTCTTTT	TGATGCTTTA	CGAGTATTCA
DE177	TGGGGGGCAT	AATGTCGTTA	TTGGTCTTTT	TGATGCTTTA	CGAGTATTCA
N16	TGGGGGGCAT	AATGTCATTA	TTGGTCTTTT	TGATGCTTTA	CGAGTATTCA
AR39	TGGGGGGCAT	AATGTCGTTA	TTGGTCTTTT	TGATGCTTTA	CGAGTATTCA
CWL029	TGGGGGGCAT	AATGTCGTTA	TTGGTCTTTT	TGATGCTTTA	CGAGTATTCA
J138	TGGGGGGCAT	AATGTCGTTA	TTGGTCTTTT	TGATGCTTTA	CGAGTATTCA
TW183	TGGGGGGCAT	AATGTCGTTA	TTGGTCTTTT	TGATGCTTTA	CGAGTATTCA
IOL207	TGGGGGGCAT	AATGTCGTTA	TTGGTCTTTT	TGATGCTTTA	CGAGTATTCA
A03	TGGGGGGCAT	AATGTCGTTA	TTGGTCTTTT	TGATGCTTTA	CGAGTATTCA
WA97001	TGGGGGGCAT	AATGTCGTTA	TTGGTCTTTT	TGATGCTTTA	CGAGTATTCA
SH511	TGGGGGGCAT	AATGTCGTTA	TTGGTCTTTT	TGATGCTTTA	CGAGTATTCA
1979	TGGGGGGCAT	AATGTCGTTA	TTGGTCTTTT	TGATGCTTTA	CGAGTATTCA

Identity	160	170	180	190	200
B26	ATCCCGAGAC	TCGCTTATTT	GGATTCATCA	AAGGACCTTT	AGGGCTTACT
B37	ATCCCGAGAC	TCGCTTATTT	GGATTCATCA	AAGGACCTTT	AGGGCTTACT
EBB	ATCCCGAGAC	TCGCTTATTT	GGATTCATCA	AAGGACCTTT	AGGGCTTACT
LPCoLN	ATCCCGAGAC	TCGCTTATTT	GGATTCATCA	AAGGACCTTT	AGGGCTTACT
Pot37	ATCCCGAGAC	TCGCTTATTT	GGATTCATCA	AAGGACCTTT	AGGGCTTACT
GBF	ATCCCGAGAC	TCGCTTATTT	GGATTCATCA	AAGGACCTTT	AGGGCTTACT
DE177	ATCCCAAGAC	TCGCTTATTT	GGATTCATCA	AAGGACCTTT	AGGGCTTACT
N16	ATCCCAAGAC	TCGCTTATTT	GGATTCATCA	AAGGACCTTT	AGGGCTTATT
AR39	ATCCCAAGAC	TCGCTTATTT	GGATTCATCA	AAGGACCTTT	AGGGCTTACT
CWL029	ATCCCAAGAC	TCGCTTATTT	GGATTCATCA	AAGGACCTTT	AGGGCTTACT
J138	ATCCCAAGAC	TCGCTTATTT	GGATTCATCA	AAGGACCTTT	AGGGCTTACT
TW183	ATCCCAAGAC	TCGCTTATTT	GGATTCATCA	AAGGACCTTT	AGGGCTTACT
IOL207	ATCCCAAGAC	TCGCTTATTT	GGATTCATCA	AAGGACCTTT	AGGGCTTACT
A03	ATCCCAAGAC	TCGCTTATTT	GGATTCATCA	AAGGACCTTT	AGGGCTTACT
WA97001	ATCCCAAGAC	TCGCTTATTT	GGATTCATCA	AAGGACCTTT	AGGGCTTACT
SH511	ATCCCAAGAC	TCGCTTATTT	GGATTCATCA	AAGGACCTTT	AGGGCTTACT
1979	ATCCCAAGAC	TCGCTTATTT	GGATTCATCA	AAGGACCTTT	AGGGCTTACT

Identity	210	220	230	240	250
B26	CGTGGGCTCT	ATAAAGATCT	GGATATCTCC	GTAATCTATG	ATTATTACAA
B37	CGTGGGCTCT	ATAAAGATCT	GGATATCTCC	GTAATCTATG	ATTATTACAA
EBB	CGTGGGCTCT	ATAAAGATCT	GGATATCTCC	GTAATCTATG	ATTATTACAA
LPCoLN	CGTGGGCTCT	ATAAAGATCT	GGATATCTCC	GTAATCTATG	ATTATTACAA
Pot37	CGTGGGCTCT	ATAAAGATCT	GGATATCTCC	GTAATCTATG	ATTATTACAA
GBF	CGTGGGCTCT	ATAAAGATCT	GGATATCTCC	GTAATCTATG	ATTATTACAA
DE177	CGTGGGCTCT	ATAAAGATCT	GGATATCTCC	GTAATCTATG	ATTATTACAA
N16	CGTGGGCTCT	ATAAAGATCT	GGATATCTCC	GTAATCTATG	ATTATTACAA
AR39	CGTGGGCTCT	ATAAAGATCT	GGATATCTCC	GTAATCTATG	ATTATTACAA
CWL029	CGTGGGCTCT	ATAAAGATCT	GGATATCTCC	GTAATCTATG	ATTATTACAA
J138	CGTGGGCTCT	ATAAAGATCT	GGATATCTCC	GTAATCTATG	ATTATTACAA
TW183	CGTGGGCTCT	ATAAAGATCT	GGATATCTCC	GTAATCTATG	ATTATTACAA
IOL207	CGTGGGCTCT	ATAAAGATCT	GGATATCTCC	GTAATCTATG	ATTATTACAA
A03	CGTGGGCTCT	ATAAAGATCT	GGATATCTCC	GTAATCTATG	ATTATTACAA
WA97001	CGTGGGCTCT	ATAAAGATCT	GGATATCTCC	GTAATCTATG	ATTATTACAA
SH511	CGTGGGCTCT	ATAAAGATCT	GGATATCTCC	GTAATCTATG	ATTATTACAA
1979	CGTGGGCTCT	ATAAAGATCT	GGATATCTCC	GTAATCTATG	ATTATTACAA

Identity	260	263
B26	CATGGGAGGG	TTC
B37	CATGGGAGGG	TTC
EBB	CATGGGAGGG	TTC
LPCoLN	CATGGGAGGG	TTC
Pot37	CATGGGAGGG	TTC
GBF	CATGGGAGGG	TTC
DE177	CATGGGAGGG	TTC
N16	CATGGGAGGG	TTC
AR39	CATGGGAGGG	TTC
CWL029	CATGGGAGGG	TTC
J138	CATGGGAGGG	TTC
TW183	CATGGGAGGG	TTC
IOL207	CATGGGAGGG	TTC
A03	CATGGGAGGG	TTC
WA97001	CATGGGAGGG	TTC
SH511	CATGGGAGGG	TTC
1979	CATGGGAGGG	TTC

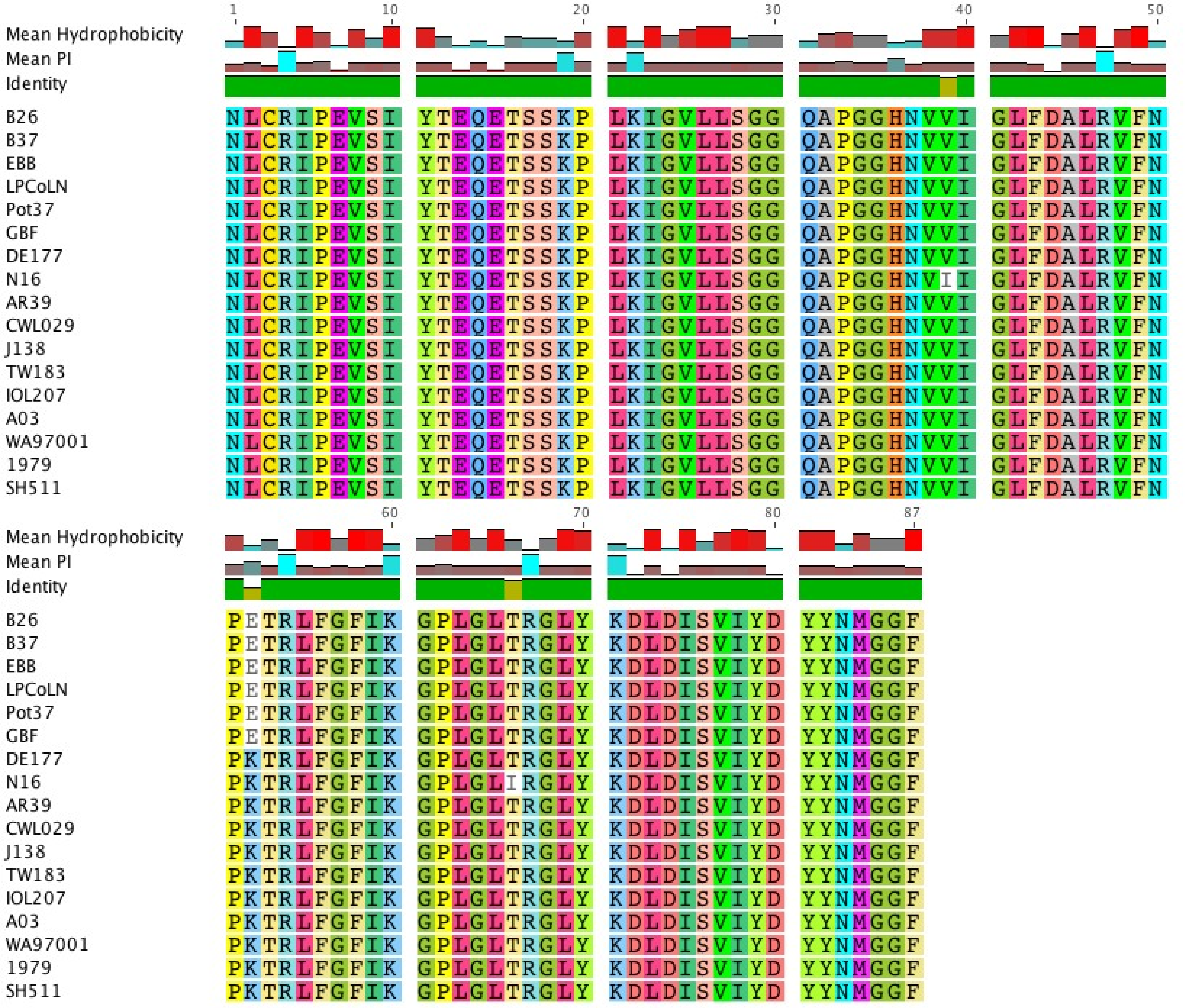

1
10
20
30
40
50
Mean Hydrophobicity
Mean PI
Identity
B26 NLCRIPEVSI YTEQETSSKP LKIGVLLSGG QAPGGHNVVI GLFDALRVFN
B37 NLCRIPEVSI YTEQETSSKP LKIGVLLSGG QAPGGHNVVI GLFDALRVFN
EBB NLCRIPEVSI YTEQETSSKP LKIGVLLSGG QAPGGHNVVI GLFDALRVFN
LPCoLN NLCRIPEVSI YTEQETSSKP LKIGVLLSGG QAPGGHNVVI GLFDALRVFN
Pot37 NLCRIPEVSI YTEQETSSKP LKIGVLLSGG QAPGGHNVVI GLFDALRVFN
GBF NLCRIPEVSI YTEQETSSKP LKIGVLLSGG QAPGGHNVVI GLFDALRVFN
DE177 NLCRIPEVSI YTEQETSSKP LKIGVLLSGG QAPGGHNVVI GLFDALRVFN
N16 NLCRIPEVSI YTEQETSSKP LKIGVLLSGG QAPGGHNVII GLFDALRVFN
AR39 NLCRIPEVSI YTEQETSSKP LKIGVLLSGG QAPGGHNVVI GLFDALRVFN
CWL029 NLCRIPEVSI YTEQETSSKP LKIGVLLSGG QAPGGHNVVI GLFDALRVFN
J138 NLCRIPEVSI YTEQETSSKP LKIGVLLSGG QAPGGHNVVI GLFDALRVFN
TW183 NLCRIPEVSI YTEQETSSKP LKIGVLLSGG QAPGGHNVVI GLFDALRVFN
IOL207 NLCRIPEVSI YTEQETSSKP LKIGVLLSGG QAPGGHNVVI GLFDALRVFN
A03 NLCRIPEVSI YTEQETSSKP LKIGVLLSGG QAPGGHNVVI GLFDALRVFN
WA97001 NLCRIPEVSI YTEQETSSKP LKIGVLLSGG QAPGGHNVVI GLFDALRVFN
1979 NLCRIPEVSI YTEQETSSKP LKIGVLLSGG QAPGGHNVVI GLFDALRVFN
SH511 NLCRIPEVSI YTEQETSSKP LKIGVLLSGG QAPGGHNVVI GLFDALRVFN
60
70
80
87
Mean Hydrophobicity
Mean PI
Identity
B26 PETRLFGFIK GPLGLTRGLY KDLDISVIYD YYNMGGF
B37 PETRLFGFIK GPLGLTRGLY KDLDISVIYD YYNMGGF
EBB PETRLFGFIK GPLGLTRGLY KDLDISVIYD YYNMGGF
LPCoLN PETRLFGFIK GPLGLTRGLY KDLDISVIYD YYNMGGF
Pot37 PETRLFGFIK GPLGLTRGLY KDLDISVIYD YYNMGGF
GBF PETRLFGFIK GPLGLTRGLY KDLDISVIYD YYNMGGF
DE177 PKTRLFGFIK GPLGLTRGLY KDLDISVIYD YYNMGGF
N16 PKTRLFGFIK GPLGLIRGLY KDLDISVIYD YYNMGGF
AR39 PKTRLFGFIK GPLGLTRGLY KDLDISVIYD YYNMGGF
CWL029 PKTRLFGFIK GPLGLTRGLY KDLDISVIYD YYNMGGF
J138 PKTRLFGFIK GPLGLTRGLY KDLDISVIYD YYNMGGF
TW183 PKTRLFGFIK GPLGLTRGLY KDLDISVIYD YYNMGGF
IOL207 PKTRLFGFIK GPLGLTRGLY KDLDISVIYD YYNMGGF
A03 PKTRLFGFIK GPLGLTRGLY KDLDISVIYD YYNMGGF
WA97001 PKTRLFGFIK GPLGLTRGLY KDLDISVIYD YYNMGGF
1979 PKTRLFGFIK GPLGLTRGLY KDLDISVIYD YYNMGGF
SH511 PKTRLFGFIK GPLGLTRGLY KDLDISVIYD YYNMGGF
