# Supplementary material for: Chlamydia pneumoniae Is Genetically Diverse in Animals and Appears to Have Crossed the Host Barrier to Humans on (At Least) Two Occasions
Source: PLoS Pathog. 2010 May 20;6(5):e1000903. doi: 10.1371/journal.ppat.1000903 (PMC2873915; doi:10.1371/journal.ppat.1000903)
Supplement: Figure S12 — Multiple sequence alignment of CP_1042. Sequence from koala (LPCoLN), frog (DE177), horse (N16), and six human isolates (AR39, CWL029, J138, TW183, SH511 and 1979) revealed gene fragmentation in the koala LPCoLN isolate. (1.13 MB PDF) [file ppat.1000903.s012.pdf]

	1–10	11–20	21–30	31–40	41–50
DE177	AATCCTTTTA	TATAAATATA	AGAGATTCAA	GGTTTTATTC	ATGGCTTTGC
N16	AATCCTTTTA	TATAAACATA	AGAGATGCGG	GATTTTATTA	ATGATTTTGC
AR39	AATCCTTTTA	TATAAATATA	AGAGATTCAA	GGTTTTATTC	ATGGCTTTGC
CWL029	AATCCTTTTA	TATAAATATA	AGAGATTCAA	GGTTTTATTC	ATGGCTTTGC
J138	AATCCTTTTA	TATAAATATA	AGAGATTCAA	GGTTTTATTC	ATGGCTTTGC
TW183	AATCCTTTTA	TATAAATATA	AGAGATTCAA	GGTTTTATTC	ATGGCTTTGC
TOR1	AATCCTTTTA	TATAAATATA	AGAGATTCAA	GGTTTTATTC	ATGGCTTTGC
WA97001	AATCCTTTTA	TATAAATATA	AGAGATTCAA	GGTTTTATTC	ATGGCTTTGC
1979	AATCCTTTTA	TATAAATATA	AGAGATTCAA	GGTTTTATTC	ATGGCTTTGC
SH511	AATCCTTTTA	TATAAATATA	AGAGATTCAA	GGTTTTATTC	ATGGCTTTGC

	51–60	61–70	71–80	81–90	91–100
DE177	TTTATAATGA	AGGAAACGTA	CTACCGCGAC	TTTCTTCATG	AGAACTATTT
N16	TTTATAATGA	AGTACGCGTC	CTTCCGTGAC	TTTATTAAAA	AGAAATATTT
AR39	TTTATAATGA	AGGAAACGTA	CTACCGCGAC	TTTCTTCATG	AGAACTATTT
CWL029	TTTATAATGA	AGGAAACGTA	CTACCGCGAC	TTTCTTCATG	AGAACTATTT
J138	TTTATAATGA	AGGAAACGTA	CTACCGCGAC	TTTCTTCATG	AGAACTATTT
TW183	TTTATAATGA	AGGAAACGTA	CTACCGCGAC	TTTCTTCATG	AGAACTATTT
TOR1	TTTATAATGA	AGGAAACGTA	CTACCGCGAC	TTTCTTCATG	AGAACTATTT
WA97001	TTTATAATGA	AGGAAACGTA	CTACCGCGAC	TTTCTTCATG	AGAACTATTT
1979	TTTATAATGA	AGGAAACGTA	CTACCGCGAC	TTTCTTCATG	AGAACTATTT
SH511	TTTATAATGA	AGGAAACGTA	CTACCGCGAC	TTTCTTCATG	AGAACTATTT

	101–110	111–120	121–130	131–140	141–150
DE177	GAAAAACAAG	AAGAGCATGT	TTATGAAAAT	TTATAAAACT	GCAGGGGAGT
N16	TAAA--TAAG	AAGAGCATGT	TTATGAAAAT	TTATAAAACC	GTTGGGGAAT
AR39	GAAAAACAAG	AAGAGCATGT	TTATGAAAAT	TTATAAAACT	GCAGGGGAGT
CWL029	GAAAAACAAG	AAGAGCATGT	TTATGAAAAT	TTATAAAACT	GCAGGGGAGT
J138	GAAAAACAAG	AAGAGCATGT	TTATGAAAAT	TTATAAAACT	GCAGGGGAGT
TW183	GAAAAACAAG	AAGAGCATGT	TTATGAAAAT	TTATAAAACT	GCAGGGGAGT
TOR1	GAAAAACAAG	AAGAGCATGT	TTATGAAAAT	TTATAAAACT	GCAGGGGAGT
WA97001	GAAAAACAAG	AAGAGCATGT	TTATGAAAAT	TTATAAAACT	GCAGGGGAGT
1979	GAAAAACAAG	AAGAGCATGT	TTATGAAAAT	TTATAAAACT	GCAGGGGAGT
SH511	GAAAAACAAG	AAGAGCATGT	TTATGAAAAT	TTATAAAACT	GCAGGGGAGT

	151–160	161–170	171–180	181–190	191–200
DE177	TTTTTTTAGC	AAATGCAAAA	TGGCCCTTGG	TACCGGCTGG	GTATCGACGT
N16	TTTTTGTAGC	AAATGCAGAC	TGGCCGTTGA	TATGGGCTGG	GTATCGACGT
AR39	TTTTTTTAGC	AAATGCAAAA	TGGCCCTTGG	TACCGGCTGG	GTATCGACGT
CWL029	TTTTTTTAGC	AAATGCAAAA	TGGCCCTTGG	TACCGGCTGG	GTATCGACGT
J138	TTTTTTTAGC	AAATGCAAAA	TGGCCCTTGG	TACCGGCTGG	GTATCGACGT
TW183	TTTTTTTAGC	AAATGCAAAA	TGGCCCTTGG	TACCGGCTGG	GTATCGACGT
TOR1	TTTTTTTAGC	AAATGCAAAA	TGGCCCTTGG	TACCGGCTGG	GTATCGACGT
WA97001	TTTTTTTAGC	AAATGCAAAA	TGGCCCTTGG	TACCGGCTGG	GTATCGACGT
1979	TTTTTTTAGC	AAATGCAAAA	TGGCCCTTGG	TACCGGCTGG	GTATCGACGT
SH511	TTTTTTTAGC	AAATGCAAAA	TGGCCCTTGG	TACCGGCTGG	GTATCGACGT

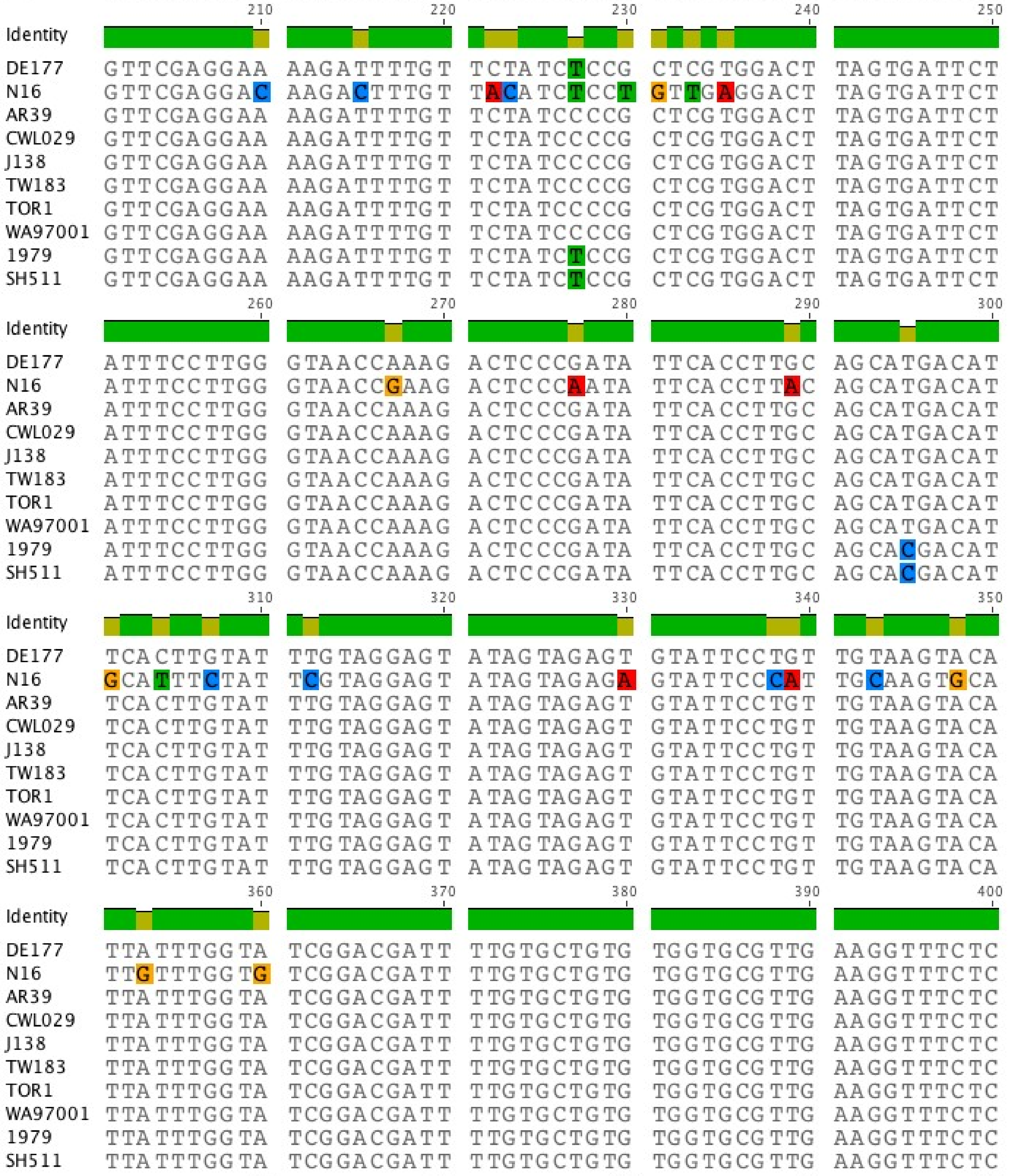

210 220 230 240 250
Identity
DE177 GTTCGAGGAA AAGATTTTGT TCTATCTCCG CTCGTGGACT TAGTGATTCT
N16 GTTCGAGGAC AAGACTTTGT TACATCTCCT GTTGAGGACT TAGTGATTCT
AR39 GTTCGAGGAA AAGATTTTGT TCTATCCCCG CTCGTGGACT TAGTGATTCT
CWL029 GTTCGAGGAA AAGATTTTGT TCTATCCCCG CTCGTGGACT TAGTGATTCT
J138 GTTCGAGGAA AAGATTTTGT TCTATCCCCG CTCGTGGACT TAGTGATTCT
TW183 GTTCGAGGAA AAGATTTTGT TCTATCCCCG CTCGTGGACT TAGTGATTCT
TOR1 GTTCGAGGAA AAGATTTTGT TCTATCCCCG CTCGTGGACT TAGTGATTCT
WA97001 GTTCGAGGAA AAGATTTTGT TCTATCCCCG CTCGTGGACT TAGTGATTCT
1979 GTTCGAGGAA AAGATTTTGT TCTATCTCCG CTCGTGGACT TAGTGATTCT
SH511 GTTCGAGGAA AAGATTTTGT TCTATCTCCG CTCGTGGACT TAGTGATTCT
260 270 280 290 300
Identity
DE177 ATTTCCTTGG GTAACCAAAG ACTCCCGATA TTCACCTTGC AGCATGACAT
N16 ATTTCCTTGG GTAACCGAAG ACTCCCAATA TTCACCTTAC AGCATGACAT
AR39 ATTTCCTTGG GTAACCAAAG ACTCCCGATA TTCACCTTGC AGCATGACAT
CWL029 ATTTCCTTGG GTAACCAAAG ACTCCCGATA TTCACCTTGC AGCATGACAT
J138 ATTTCCTTGG GTAACCAAAG ACTCCCGATA TTCACCTTGC AGCATGACAT
TW183 ATTTCCTTGG GTAACCAAAG ACTCCCGATA TTCACCTTGC AGCATGACAT
TOR1 ATTTCCTTGG GTAACCAAAG ACTCCCGATA TTCACCTTGC AGCATGACAT
WA97001 ATTTCCTTGG GTAACCAAAG ACTCCCGATA TTCACCTTGC AGCATGACAT
1979 ATTTCCTTGG GTAACCAAAG ACTCCCGATA TTCACCTTGC AGCACGACAT
SH511 ATTTCCTTGG GTAACCAAAG ACTCCCGATA TTCACCTTGC AGCACGACAT
310 320 330 340 350
Identity
DE177 TCACTTGTAT TTGTAGGAGT ATAGTAGAGT GTATTCCTGT TGTAAGTACA
N16 GCATTTCTAT TCGTAGGAGT ATAGTAGAGA GTATTCCCAT TGCAAGTGCA
AR39 TCACTTGTAT TTGTAGGAGT ATAGTAGAGT GTATTCCTGT TGTAAGTACA
CWL029 TCACTTGTAT TTGTAGGAGT ATAGTAGAGT GTATTCCTGT TGTAAGTACA
J138 TCACTTGTAT TTGTAGGAGT ATAGTAGAGT GTATTCCTGT TGTAAGTACA
TW183 TCACTTGTAT TTGTAGGAGT ATAGTAGAGT GTATTCCTGT TGTAAGTACA
TOR1 TCACTTGTAT TTGTAGGAGT ATAGTAGAGT GTATTCCTGT TGTAAGTACA
WA97001 TCACTTGTAT TTGTAGGAGT ATAGTAGAGT GTATTCCTGT TGTAAGTACA
1979 TCACTTGTAT TTGTAGGAGT ATAGTAGAGT GTATTCCTGT TGTAAGTACA
SH511 TCACTTGTAT TTGTAGGAGT ATAGTAGAGT GTATTCCTGT TGTAAGTACA
360 370 380 390 400
Identity
DE177 TTATTTGGTA TCGGACGATT TTGTGCTGTG TGGTGCGTTG AAGGTTTCTC
N16 TTGTTTGGTG TCGGACGATT TTGTGCTGTG TGGTGCGTTG AAGGTTTCTC
AR39 TTATTTGGTA TCGGACGATT TTGTGCTGTG TGGTGCGTTG AAGGTTTCTC
CWL029 TTATTTGGTA TCGGACGATT TTGTGCTGTG TGGTGCGTTG AAGGTTTCTC
J138 TTATTTGGTA TCGGACGATT TTGTGCTGTG TGGTGCGTTG AAGGTTTCTC
TW183 TTATTTGGTA TCGGACGATT TTGTGCTGTG TGGTGCGTTG AAGGTTTCTC
TOR1 TTATTTGGTA TCGGACGATT TTGTGCTGTG TGGTGCGTTG AAGGTTTCTC
WA97001 TTATTTGGTA TCGGACGATT TTGTGCTGTG TGGTGCGTTG AAGGTTTCTC
1979 TTATTTGGTA TCGGACGATT TTGTGCTGTG TGGTGCGTTG AAGGTTTCTC
SH511 TTATTTGGTA TCGGACGATT TTGTGCTGTG TGGTGCGTTG AAGGTTTCTC

	410	420	430	440	450
Identity					
DE177	AGGCTCTACG	TTTGATAAGA	TCTATCATAC	AATTGTCGCC	GTTCTAGGAA
N16	AGGCTCTACG	TTTGATAAGA	TCTATCATAC	AATTGTCGCC	GTTCTAGGAA
AR39	AGGCTCTACG	TTTGATAAGA	TCTATCATAC	AATTGTCGCC	GTTCTAGGAA
CWL029	AGGCTCTACG	TTTGATAAGA	TCTATCATAC	AATTGTCGCC	GTTCTAGGAA
J138	AGGCTCTACG	TTTGATAAGA	TCTATCATAC	AATTGTCGCC	GTTCTAGGAA
TW183	AGGCTCTACG	TTTGATAAGA	TCTATCATAC	AATTGTCGCC	GTTCTAGGAA
TOR1	AGGCTCTACG	TTTGATAAGA	TCTATCATAC	AATTGTCGCC	GTTCTAGGAA
WA97001	AGGCTCTACG	TTTGATAAGA	TCTATCATAC	AATTGTCGCC	GTTCTAGGAA
1979	AGGCTCTACG	TTTGATAAGA	TCTATCATAC	AATTGTCGCC	GTTCTAGGAA
SH511	AGGCTCTACG	TTTGATAAGA	TCTATCATAC	AATTGTCGCC	GTTCTAGGAA

	460	470	480	490	
Identity					
DE177	TTCTTGGTTT	GGGAATTCTT	ACGTTCATTT	TAAGAATTAT	TTTTTCTGTG
N16	TTCTTGGTTT	GGGAATTCTT	ACGTTCATTT	TAAGAATTAT	TTTTTCTGTG
AR39	TTCTTGGTTT	GGGAATTCTT	ACGTTCATTT	TAAGAATTAT	TTTTTCTGTG
CWL029	TTCTTGGTTT	GGGAATTCTT	ACGTTCATTT	TAAGAATTAT	TTTTTCTGTG
J138	TTCTTGGTTT	GGGAATTCTT	ACGTTCATTT	TAAGAATTAT	TTTTTCTGTG
TW183	TTCTTGGTTT	GGGAATTCTT	ACGTTCATTT	TAAGAATTAT	TTTTTCTGTG
TOR1	TTCTTGGTTT	GGGAATTCTT	ACGTTCATTT	TAAGAATTAT	TTTTTCTGTG
WA97001	TTCTTGGTTT	GGGAATTCTT	ACGTTCATTT	TAAGAATTAT	TTTTTCTGTG
1979	TTCTTGGTTT	GGGAATTCTT	ACGTTCATTT	TAAGAATTAT	TTTTTCTGTG
SH511	TTCTTGGTTT	GGGAATTCTT	ACGTTCATTT	TAAGAATTAT	TTTTTCTGTG

	505
Identity	
DE177	CTTAT
N16	CTTAT
AR39	CTTAT
CWL029	CTTAT
J138	CTTAT
TW183	CTTAT
TOR1	CTTAT
WA97001	CTTAT
1979	CTTAT
SH511	CTTAT

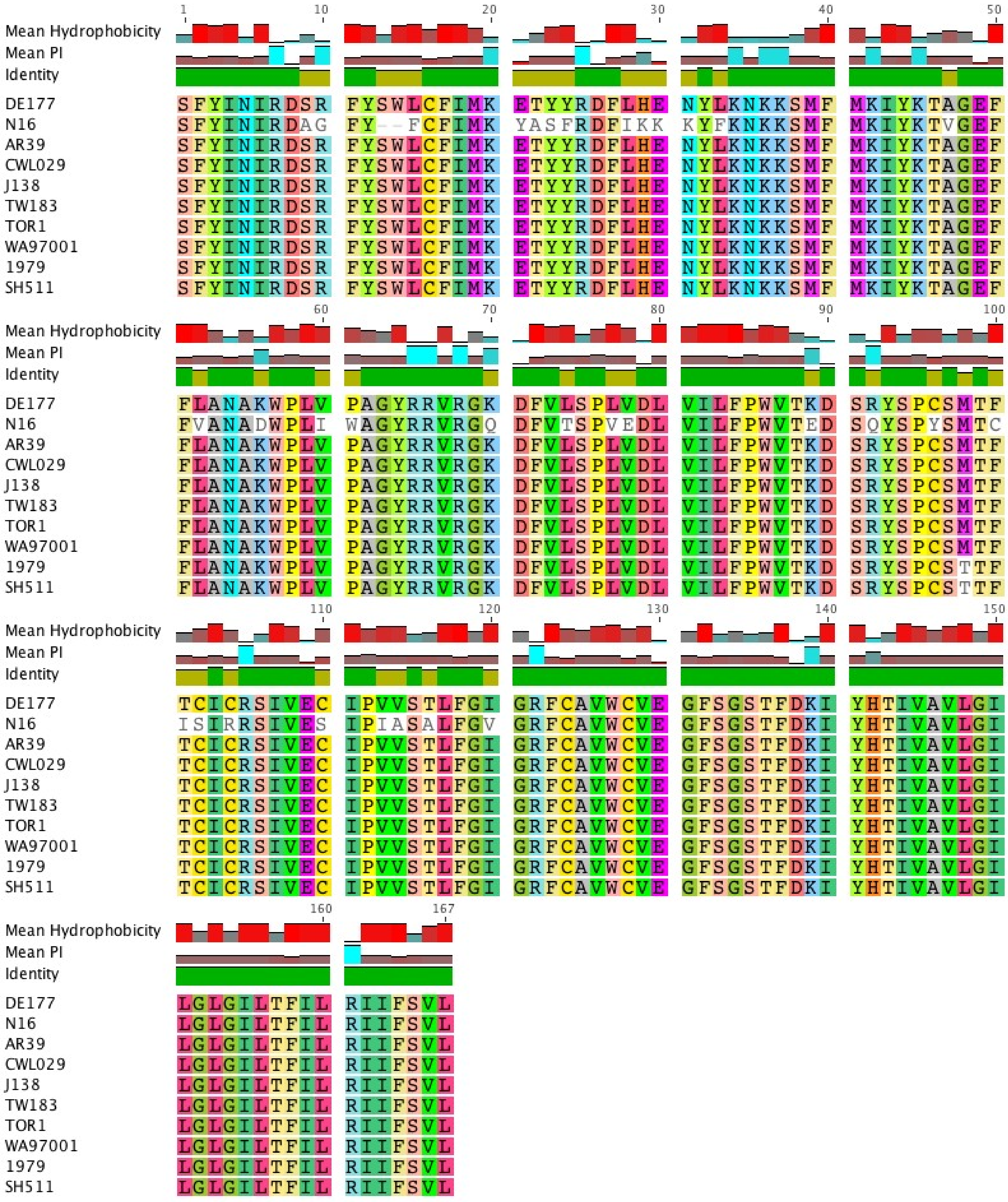
1 10 20 30 40 50
Mean Hydrophobicity
Mean PI
Identity
DE177 SFYINIRDSR FYSWLCFIMK ETYYRDFLHE NYLKNKKSMF MKIYKTAGEF
N16 SFYINIRDAG FY--FCFIMK YASFRDFIKK KYFKNKKSMF MKIYKTVGEF
AR39 SFYINIRDSR FYSWLCFIMK ETYYRDFLHE NYLKNKKSMF MKIYKTAGEF
CWL029 SFYINIRDSR FYSWLCFIMK ETYYRDFLHE NYLKNKKSMF MKIYKTAGEF
J138 SFYINIRDSR FYSWLCFIMK ETYYRDFLHE NYLKNKKSMF MKIYKTAGEF
TW183 SFYINIRDSR FYSWLCFIMK ETYYRDFLHE NYLKNKKSMF MKIYKTAGEF
TOR1 SFYINIRDSR FYSWLCFIMK ETYYRDFLHE NYLKNKKSMF MKIYKTAGEF
WA97001 SFYINIRDSR FYSWLCFIMK ETYYRDFLHE NYLKNKKSMF MKIYKTAGEF
1979 SFYINIRDSR FYSWLCFIMK ETYYRDFLHE NYLKNKKSMF MKIYKTAGEF
SH511 SFYINIRDSR FYSWLCFIMK ETYYRDFLHE NYLKNKKSMF MKIYKTAGEF
60 70 80 90 100
Mean Hydrophobicity
Mean PI
Identity
DE177 FLANAKWPLV PAGYRRVRGK DFVLSPLVDL VILFPWVTKD SRYSPCSMTF
N16 FVANADWPLI WAGYRRVRGQ DFVTSPVEDL VILFPWVTED SQYSPYSMTC
AR39 FLANAKWPLV PAGYRRVRGK DFVLSPLVDL VILFPWVTKD SRYSPCSMTF
CWL029 FLANAKWPLV PAGYRRVRGK DFVLSPLVDL VILFPWVTKD SRYSPCSMTF
J138 FLANAKWPLV PAGYRRVRGK DFVLSPLVDL VILFPWVTKD SRYSPCSMTF
TW183 FLANAKWPLV PAGYRRVRGK DFVLSPLVDL VILFPWVTKD SRYSPCSMTF
TOR1 FLANAKWPLV PAGYRRVRGK DFVLSPLVDL VILFPWVTKD SRYSPCSMTF
WA97001 FLANAKWPLV PAGYRRVRGK DFVLSPLVDL VILFPWVTKD SRYSPCSMTF
1979 FLANAKWPLV PAGYRRVRGK DFVLSPLVDL VILFPWVTKD SRYSPCSTTF
SH511 FLANAKWPLV PAGYRRVRGK DFVLSPLVDL VILFPWVTKD SRYSPCSTTF
110 120 130 140 150
Mean Hydrophobicity
Mean PI
Identity
DE177 TCICRSIVEC IPVVSTLFGI GRFCAVWCVE GFSGSTFDKI YHTIVAVLGI
N16 ISIRRSIVES IPIASALFGV GRFCAVWCVE GFSGSTFDKI YHTIVAVLGI
AR39 TCICRSIVEC IPVVSTLFGI GRFCAVWCVE GFSGSTFDKI YHTIVAVLGI
CWL029 TCICRSIVEC IPVVSTLFGI GRFCAVWCVE GFSGSTFDKI YHTIVAVLGI
J138 TCICRSIVEC IPVVSTLFGI GRFCAVWCVE GFSGSTFDKI YHTIVAVLGI
TW183 TCICRSIVEC IPVVSTLFGI GRFCAVWCVE GFSGSTFDKI YHTIVAVLGI
TOR1 TCICRSIVEC IPVVSTLFGI GRFCAVWCVE GFSGSTFDKI YHTIVAVLGI
WA97001 TCICRSIVEC IPVVSTLFGI GRFCAVWCVE GFSGSTFDKI YHTIVAVLGI
1979 TCICRSIVEC IPVVSTLFGI GRFCAVWCVE GFSGSTFDKI YHTIVAVLGI
SH511 TCICRSIVEC IPVVSTLFGI GRFCAVWCVE GFSGSTFDKI YHTIVAVLGI
160 167
Mean Hydrophobicity
Mean PI
Identity
DE177 LGLGILTFIL RIIFSVL
N16 LGLGILTFIL RIIFSVL
AR39 LGLGILTFIL RIIFSVL
CWL029 LGLGILTFIL RIIFSVL
J138 LGLGILTFIL RIIFSVL
TW183 LGLGILTFIL RIIFSVL
TOR1 LGLGILTFIL RIIFSVL
WA97001 LGLGILTFIL RIIFSVL
1979 LGLGILTFIL RIIFSVL
SH511 LGLGILTFIL RIIFSVL
