# Supplementary material for: Chlamydia pneumoniae Is Genetically Diverse in Animals and Appears to Have Crossed the Host Barrier to Humans on (At Least) Two Occasions
Source: PLoS Pathog. 2010 May 20;6(5):e1000903. doi: 10.1371/journal.ppat.1000903 (PMC2873915; doi:10.1371/journal.ppat.1000903)
Supplement: Figure S13 — Multiple sequence alignment of CP_0880. The two koala isolates (LPCoLN and EBB) and bandicoot isolate (B26) are identical in sequence and have diverged genetically (110 SNPs) from the eight human isolates (AR39, CWL029, J138, TW183, TOR1, WA97001, SH511 and 1979). There are two shared polymorphisms (positions 714 and 818 bp) among koala LPCoLN, bandicoot B26 and Indigenous human isolates SH511 and 1979. (5.80 MB PDF) [file ppat.1000903.s013.pdf]

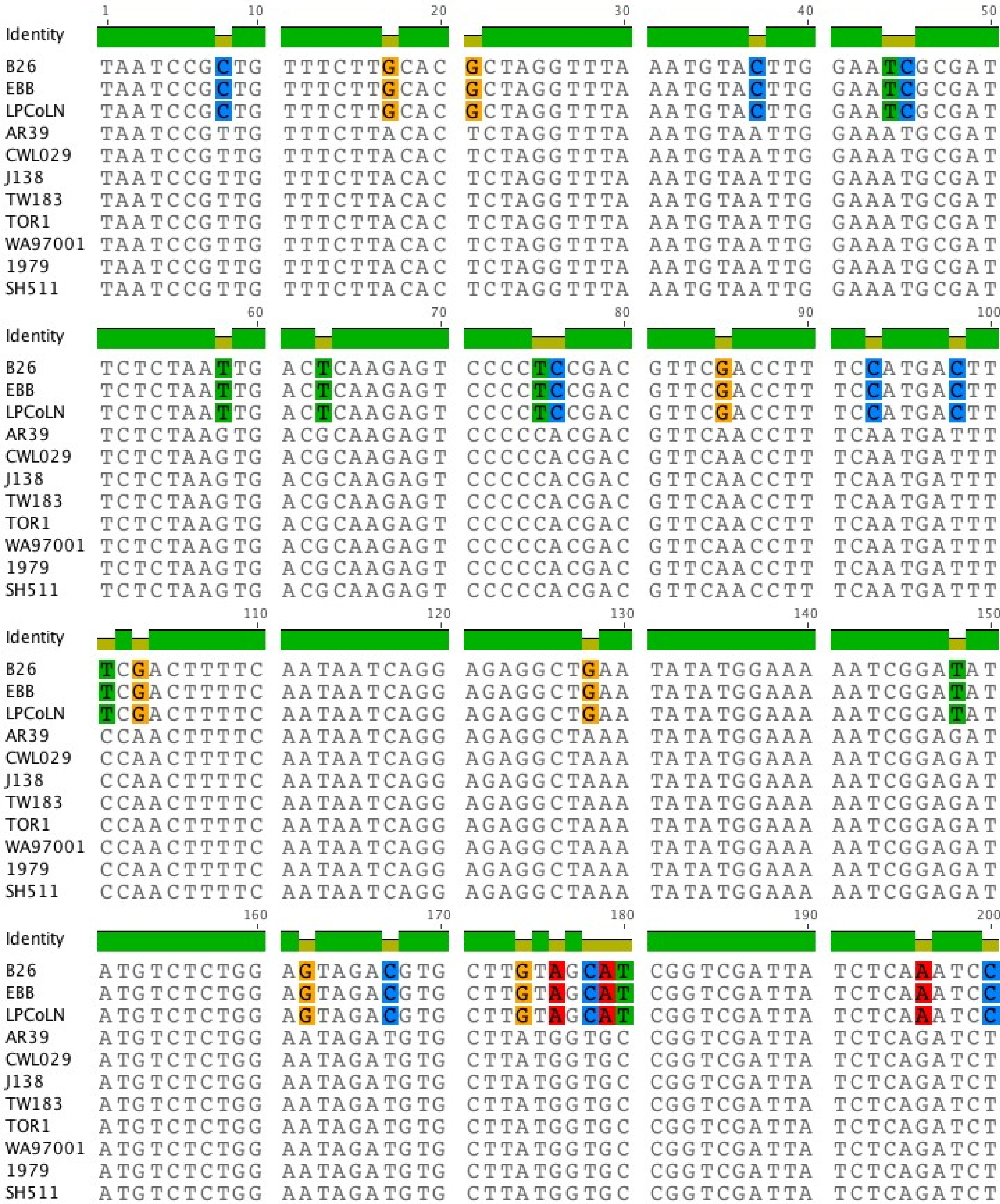
1 10 20 30 40 50
Identity
B26 TAATCCGCTG TTTCTTGCAC GCTAGGTTTA AATGTACTTG GAATCGCGAT
EBB TAATCCGCTG TTTCTTGCAC GCTAGGTTTA AATGTACTTG GAATCGCGAT
LPCoLN TAATCCGCTG TTTCTTGCAC GCTAGGTTTA AATGTACTTG GAATCGCGAT
AR39 TAATCCGTTG TTTCTTACAC TCTAGGTTTA AATGTAATTG GAAATGCGAT
CWL029 TAATCCGTTG TTTCTTACAC TCTAGGTTTA AATGTAATTG GAAATGCGAT
J138 TAATCCGTTG TTTCTTACAC TCTAGGTTTA AATGTAATTG GAAATGCGAT
TW183 TAATCCGTTG TTTCTTACAC TCTAGGTTTA AATGTAATTG GAAATGCGAT
TOR1 TAATCCGTTG TTTCTTACAC TCTAGGTTTA AATGTAATTG GAAATGCGAT
WA97001 TAATCCGTTG TTTCTTACAC TCTAGGTTTA AATGTAATTG GAAATGCGAT
1979 TAATCCGTTG TTTCTTACAC TCTAGGTTTA AATGTAATTG GAAATGCGAT
SH511 TAATCCGTTG TTTCTTACAC TCTAGGTTTA AATGTAATTG GAAATGCGAT
60 70 80 90 100
Identity
B26 TCTCTAATTG ACTCAAGAGT CCCCTCCGAC GTTCGACCTT TCCATGACTT
EBB TCTCTAATTG ACTCAAGAGT CCCCTCCGAC GTTCGACCTT TCCATGACTT
LPCoLN TCTCTAATTG ACTCAAGAGT CCCCTCCGAC GTTCGACCTT TCCATGACTT
AR39 TCTCTAAGTG ACGCAAGAGT CCCCCACGAC GTTCAACCTT TCAATGATTT
CWL029 TCTCTAAGTG ACGCAAGAGT CCCCCACGAC GTTCAACCTT TCAATGATTT
J138 TCTCTAAGTG ACGCAAGAGT CCCCCACGAC GTTCAACCTT TCAATGATTT
TW183 TCTCTAAGTG ACGCAAGAGT CCCCCACGAC GTTCAACCTT TCAATGATTT
TOR1 TCTCTAAGTG ACGCAAGAGT CCCCCACGAC GTTCAACCTT TCAATGATTT
WA97001 TCTCTAAGTG ACGCAAGAGT CCCCCACGAC GTTCAACCTT TCAATGATTT
1979 TCTCTAAGTG ACGCAAGAGT CCCCCACGAC GTTCAACCTT TCAATGATTT
SH511 TCTCTAAGTG ACGCAAGAGT CCCCCACGAC GTTCAACCTT TCAATGATTT
110 120 130 140 150
Identity
B26 TCGACTTTTC AATAATCAGG AGAGGCTGAA TATATGGAAA AATCGGATAT
EBB TCGACTTTTC AATAATCAGG AGAGGCTGAA TATATGGAAA AATCGGATAT
LPCoLN TCGACTTTTC AATAATCAGG AGAGGCTGAA TATATGGAAA AATCGGATAT
AR39 CCAACTTTTC AATAATCAGG AGAGGCTAAA TATATGGAAA AATCGGAGAT
CWL029 CCAACTTTTC AATAATCAGG AGAGGCTAAA TATATGGAAA AATCGGAGAT
J138 CCAACTTTTC AATAATCAGG AGAGGCTAAA TATATGGAAA AATCGGAGAT
TW183 CCAACTTTTC AATAATCAGG AGAGGCTAAA TATATGGAAA AATCGGAGAT
TOR1 CCAACTTTTC AATAATCAGG AGAGGCTAAA TATATGGAAA AATCGGAGAT
WA97001 CCAACTTTTC AATAATCAGG AGAGGCTAAA TATATGGAAA AATCGGAGAT
1979 CCAACTTTTC AATAATCAGG AGAGGCTAAA TATATGGAAA AATCGGAGAT
SH511 CCAACTTTTC AATAATCAGG AGAGGCTAAA TATATGGAAA AATCGGAGAT
160 170 180 190 200
Identity
B26 ATGTCTCTGG AGTAGACGTG CTTGTAGCAT CGGTCGATTA TCTCAAATCC
EBB ATGTCTCTGG AGTAGACGTG CTTGTAGCAT CGGTCGATTA TCTCAAATCC
LPCoLN ATGTCTCTGG AGTAGACGTG CTTGTAGCAT CGGTCGATTA TCTCAAATCC
AR39 ATGTCTCTGG AATAGATGTG CTTATGGTGC CGGTCGATTA TCTCAGATCT
CWL029 ATGTCTCTGG AATAGATGTG CTTATGGTGC CGGTCGATTA TCTCAGATCT
J138 ATGTCTCTGG AATAGATGTG CTTATGGTGC CGGTCGATTA TCTCAGATCT
TW183 ATGTCTCTGG AATAGATGTG CTTATGGTGC CGGTCGATTA TCTCAGATCT
TOR1 ATGTCTCTGG AATAGATGTG CTTATGGTGC CGGTCGATTA TCTCAGATCT
WA97001 ATGTCTCTGG AATAGATGTG CTTATGGTGC CGGTCGATTA TCTCAGATCT
1979 ATGTCTCTGG AATAGATGTG CTTATGGTGC CGGTCGATTA TCTCAGATCT
SH511 ATGTCTCTGG AATAGATGTG CTTATGGTGC CGGTCGATTA TCTCAGATCT

	210	220	230	240	250
B26	CAGTTTTCTG	GTTTTAACCA	CATTCCAAAA	GCTATCCGTT	GCGAAAACTA
EBB	CAGTTTTCTG	GTTTTAACCA	CATTCCAAAA	GCTATCCGTT	GCGAAAACTA
LPCoLN	CAGTTTTCTG	GTTTTAACCA	CATTCCAAAA	GCTATCCGTT	GCGAAAACTA
AR39	CAGTTTCCCG	GTTTTAAGGA	GATTCCAGAA	GCTATCCGTT	GCGAAAACTA
CWL029	CAGTTTCCCG	GTTTTAAGGA	GATTCCAGAA	GCTATCCGTT	GCGAAAACTA
J138	CAGTTTCCCG	GTTTTAAGGA	GATTCCAGAA	GCTATCCGTT	GCGAAAACTA
TW183	CAGTTTCCCG	GTTTTAAGGA	GATTCCAGAA	GCTATCCGTT	GCGAAAACTA
TOR1	CAGTTTCCCG	GTTTTAAGGA	GATTCCAGAA	GCTATCCGTT	GCGAAAACTA
WA97001	CAGTTTCCCG	GTTTTAAGGA	GATTCCAGAA	GCTATCCGTT	GCGAAAACTA
1979	CAGTTTCCTG	GTTTTAAGGA	GATTCCAGAA	GCTATCCGTT	GCGAAAACTA
SH511	CAGTTTCCTG	GTTTTAAGGA	GATTCCAGAA	GCTATCCGTT	GCGAAAACTA

	260	270	280	290	300
B26	TGTAAGAAAT	GGTCAGTTTT	CTGAAGAAGG	TAAAAAGAGT	TATCTTAGAG
EBB	TGTAAGAAAT	GGTCAGTTTT	CTGAAGAAGG	TAAAAAGAGT	TATCTTAGAG
LPCoLN	TGTAAGAAAT	GGTCAGTTTT	CTGAAGAAGG	TAAAAAGAGT	TATCTTAGAG
AR39	TGTAAGTGAT	GGTCAGTTTT	CTGAAGAAAG	TAAAACAAGC	TATCTTAGAG
CWL029	TGTAAGTGAT	GGTCAGTTTT	CTGAAGAAAG	TAAAACAAGC	TATCTTAGAG
J138	TGTAAGTGAT	GGTCAGTTTT	CTGAAGAAAG	TAAAACAAGC	TATCTTAGAG
TW183	TGTAAGTGAT	GGTCAGTTTT	CTGAAGAAAG	TAAAACAAGC	TATCTTAGAG
TOR1	TGTAAGTGAT	GGTCAGTTTT	CTGAAGAAAG	TAAAACAAGC	TATCTTAGAG
WA97001	TGTAAGTGAT	GGTCAGTTTT	CTGAAGAAAG	TAAAACAAGC	TATCTTAGAG
1979	TGTAAGTGAT	GGTCAGTTTT	CTGAAGAAAG	TAAAACAAGC	TATCTTAGAG
SH511	TGTAAGTGAT	GGTCAGTTTT	CTGAAGAAAG	TAAAACAAGC	TATCTTAGAG

	310	320	330	340	350
B26	GGATGCTGAC	CCACATTGTT	GGGTATATCT	TGTCAGTAGA	TGAGACCTAT
EBB	GGATGCTGAC	CCACATTGTT	GGGTATATCT	TGTCAGTAGA	TGAGACCTAT
LPCoLN	GGATGCTGAC	CCACATTGTT	GGGTATATCT	TGTCAGTAGA	TGAGACCTAT
AR39	CGATGCTGAC	CGACATTGTT	GGGTATATCT	TGTCATTAGA	TGAGACCTAT
CWL029	CGATGCTGAC	CGACATTGTT	GGGTATATCT	TGTCATTAGA	TGAGACCTAT
J138	CGATGCTGAC	CGACATTGTT	GGGTATATCT	TGTCATTAGA	TGAGACCTAT
TW183	CGATGCTGAC	CGACATTGTT	GGGTATATCT	TGTCATTAGA	TGAGACCTAT
TOR1	CGATGCTGAC	CGACATTGTT	GGGTATATCT	TGTCATTAGA	TGAGACCTAT
WA97001	CGATGCTGAC	CGACATTGTT	GGGTATATCT	TGTCATTAGA	TGAGACCTAT
1979	CGATGCTGAC	CGACATTGTT	GGGTATATCT	TGTCATTAGA	TGAGACCTAT
SH511	CGATGCTGAC	CGACATTGTT	GGGTATATCT	TGTCATTAGA	TGAGACCTAT

	360	370	380	390	400
B26	TGGGAGGATG	TGATCCTCAA	GATCCCTGCG	ATGAACATCC	TTAGCGAAGC
EBB	TGGGAGGATG	TGATCCTCAA	GATCCCTGCG	ATGAACATCC	TTAGCGAAGC
LPCoLN	TGGGAGGATG	TGATCCTCAA	GATCCCTGCG	ATGAACATCC	TTAGCGAAGC
AR39	TGGACGAATG	TGATCCTCAA	GATCCGTGCG	ATGTGCATCA	CCTTCGAAAG
CWL029	TGGACGAATG	TGATCCTCAA	GATCCGTGCG	ATGTGCATCA	CCTTCGAAAG
J138	TGGACGAATG	TGATCCTCAA	GATCCGTGCG	ATGTGCATCA	CCTTCGAAAG
TW183	TGGACGAATG	TGATCCTCAA	GATCCGTGCG	ATGTGCATCA	CCTTCGAAAG
TOR1	TGGACGAATG	TGATCCTCAA	GATCCGTGCG	ATGTGCATCA	CCTTCGAAAG
WA97001	TGGACGAATG	TGATCCTCAA	GATCCGTGCG	ATGTGCATCA	CCTTCGAAAG
1979	TGGACGAATG	TGATCCTCAA	GATCCGTGCG	ATGTGCATCA	CCTTCGAAAG
SH511	TGGACGAATG	TGATCCTCAA	GATCCGTGCG	ATGTGCATCA	CCTTCGAAAG

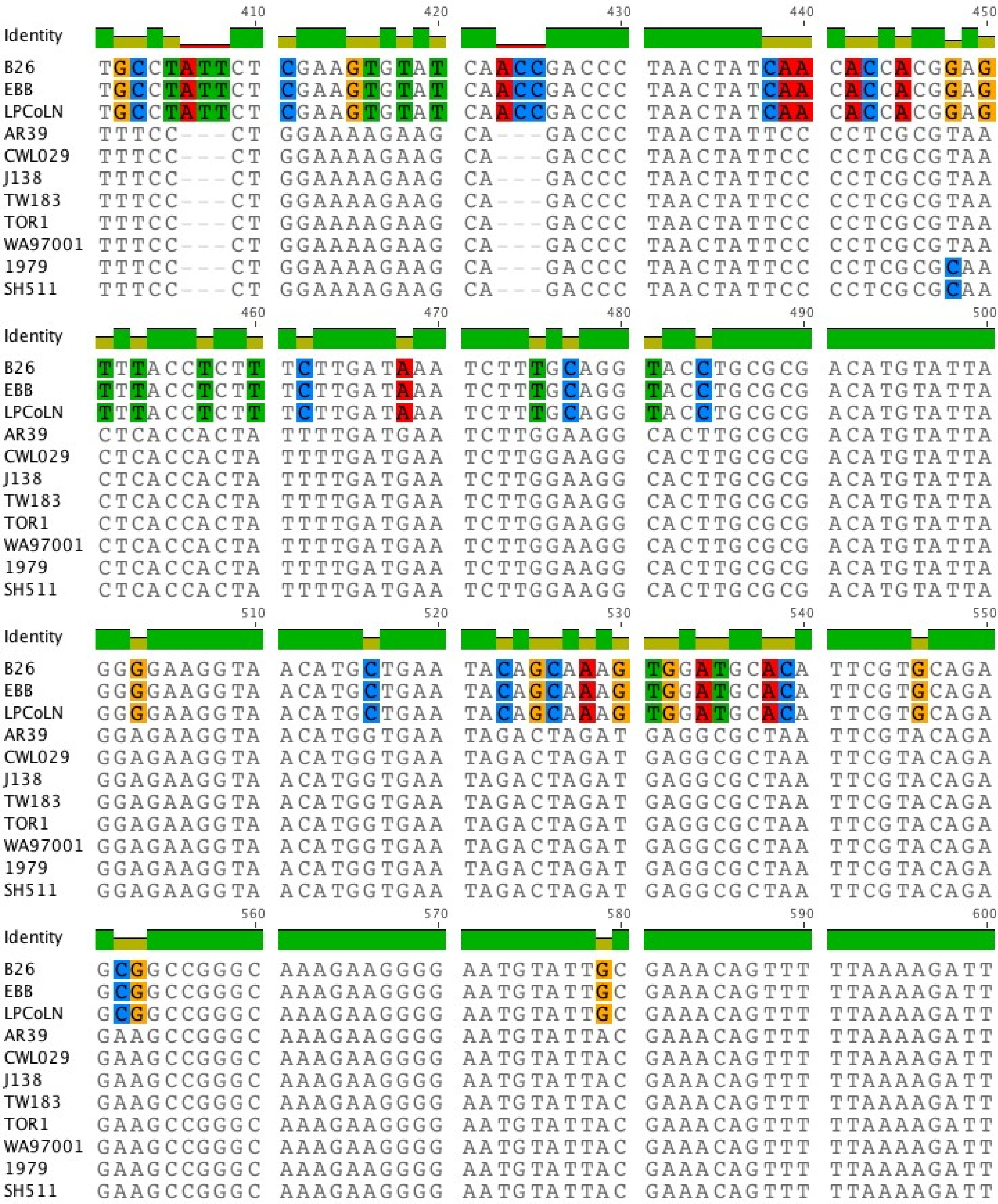
410 420 430 440 450
Identity
B26 TGCCTATTCT CGAAGTGTAT CAACCGACCC TAACTATCAA CACCACGGAG
EBB TGCCTATTCT CGAAGTGTAT CAACCGACCC TAACTATCAA CACCACGGAG
LPCoLN TGCCTATTCT CGAAGTGTAT CAACCGACCC TAACTATCAA CACCACGGAG
AR39 TTTCC---CT GGAAAAGAAG CA---GACCC TAACTATTCC CCTCGCGTAA
CWL029 TTTCC---CT GGAAAAGAAG CA---GACCC TAACTATTCC CCTCGCGTAA
J138 TTTCC---CT GGAAAAGAAG CA---GACCC TAACTATTCC CCTCGCGTAA
TW183 TTTCC---CT GGAAAAGAAG CA---GACCC TAACTATTCC CCTCGCGTAA
TOR1 TTTCC---CT GGAAAAGAAG CA---GACCC TAACTATTCC CCTCGCGTAA
WA97001 TTTCC---CT GGAAAAGAAG CA---GACCC TAACTATTCC CCTCGCGTAA
1979 TTTCC---CT GGAAAAGAAG CA---GACCC TAACTATTCC CCTCGCGCAA
SH511 TTTCC---CT GGAAAAGAAG CA---GACCC TAACTATTCC CCTCGCGCAA
460 470 480 490 500
Identity
B26 TTTACCTCTT TCTTGATAAA TCTTTGCAGG TACCTGCGCG ACATGTATTA
EBB TTTACCTCTT TCTTGATAAA TCTTTGCAGG TACCTGCGCG ACATGTATTA
LPCoLN TTTACCTCTT TCTTGATAAA TCTTTGCAGG TACCTGCGCG ACATGTATTA
AR39 CTCACCACTA TTTTGATGAA TCTTGGAAGG CACTTGCGCG ACATGTATTA
CWL029 CTCACCACTA TTTTGATGAA TCTTGGAAGG CACTTGCGCG ACATGTATTA
J138 CTCACCACTA TTTTGATGAA TCTTGGAAGG CACTTGCGCG ACATGTATTA
TW183 CTCACCACTA TTTTGATGAA TCTTGGAAGG CACTTGCGCG ACATGTATTA
TOR1 CTCACCACTA TTTTGATGAA TCTTGGAAGG CACTTGCGCG ACATGTATTA
WA97001 CTCACCACTA TTTTGATGAA TCTTGGAAGG CACTTGCGCG ACATGTATTA
1979 CTCACCACTA TTTTGATGAA TCTTGGAAGG CACTTGCGCG ACATGTATTA
SH511 CTCACCACTA TTTTGATGAA TCTTGGAAGG CACTTGCGCG ACATGTATTA
510 520 530 540 550
Identity
B26 GGGGAAGGTA ACATGCTGAA TACAGCAAAG TGGATGCACA TTCGTGCAGA
EBB GGGGAAGGTA ACATGCTGAA TACAGCAAAG TGGATGCACA TTCGTGCAGA
LPCoLN GGGGAAGGTA ACATGCTGAA TACAGCAAAG TGGATGCACA TTCGTGCAGA
AR39 GGAGAAGGTA ACATGGTGAA TAGACTAGAT GAGGCGCTAA TTCGTACAGA
CWL029 GGAGAAGGTA ACATGGTGAA TAGACTAGAT GAGGCGCTAA TTCGTACAGA
J138 GGAGAAGGTA ACATGGTGAA TAGACTAGAT GAGGCGCTAA TTCGTACAGA
TW183 GGAGAAGGTA ACATGGTGAA TAGACTAGAT GAGGCGCTAA TTCGTACAGA
TOR1 GGAGAAGGTA ACATGGTGAA TAGACTAGAT GAGGCGCTAA TTCGTACAGA
WA97001 GGAGAAGGTA ACATGGTGAA TAGACTAGAT GAGGCGCTAA TTCGTACAGA
1979 GGAGAAGGTA ACATGGTGAA TAGACTAGAT GAGGCGCTAA TTCGTACAGA
SH511 GGAGAAGGTA ACATGGTGAA TAGACTAGAT GAGGCGCTAA TTCGTACAGA
560 570 580 590 600
Identity
B26 GCGGCCGGGC AAAGAAGGGG AATGTATTGC GAAACAGTTT TTAAAAGATT
EBB GCGGCCGGGC AAAGAAGGGG AATGTATTGC GAAACAGTTT TTAAAAGATT
LPCoLN GCGGCCGGGC AAAGAAGGGG AATGTATTGC GAAACAGTTT TTAAAAGATT
AR39 GAAGCCGGGC AAAGAAGGGG AATGTATTAC GAAACAGTTT TTAAAAGATT
CWL029 GAAGCCGGGC AAAGAAGGGG AATGTATTAC GAAACAGTTT TTAAAAGATT
J138 GAAGCCGGGC AAAGAAGGGG AATGTATTAC GAAACAGTTT TTAAAAGATT
TW183 GAAGCCGGGC AAAGAAGGGG AATGTATTAC GAAACAGTTT TTAAAAGATT
TOR1 GAAGCCGGGC AAAGAAGGGG AATGTATTAC GAAACAGTTT TTAAAAGATT
WA97001 GAAGCCGGGC AAAGAAGGGG AATGTATTAC GAAACAGTTT TTAAAAGATT
1979 GAAGCCGGGC AAAGAAGGGG AATGTATTAC GAAACAGTTT TTAAAAGATT
SH511 GAAGCCGGGC AAAGAAGGGG AATGTATTAC GAAACAGTTT TTAAAAGATT

	610	620	630	640	650
B26	ACTGTAAAAA	GCATCTCGAA	GTTATGAATT	GTCCAGATTT	CATTGAATCT
EBB	ACTGTAAAAA	GCATCTCGAA	GTTATGAATT	GTCCAGATTT	CATTGAATCT
LPCoLN	ACTGTAAAAA	GCATCTCGAA	GTTATGAATT	GTCCAGATTT	CATTGAATCT
AR39	ACTGTAAAAA	GCATCTCGAA	GTTATGAGTT	GTCCAGATTT	CATTGAATCT
CWL029	ACTGTAAAAA	GCATCTCGAA	GTTATGAGTT	GTCCAGATTT	CATTGAATCT
J138	ACTGTAAAAA	GCATCTCGAA	GTTATGAGTT	GTCCAGATTT	CATTGAATCT
TW183	ACTGTAAAAA	GCATCTCGAA	GTTATGAGTT	GTCCAGATTT	CATTGAATCT
TOR1	ACTGTAAAAA	GCATCTCGAA	GTTATGAGTT	GTCCAGATTT	CATTGAATCT
WA97001	ACTGTAAAAA	GCATCTCGAA	GTTATGAGTT	GTCCAGATTT	CATTGAATCT
1979	ACTGTAAAAA	GCATCTCGAA	GTTATGAGTT	GTCCAGATTT	CATTGAATCT
SH511	ACTGTAAAAA	GCATCTCGAA	GTTATGAGTT	GTCCAGATTT	CATTGAATCT

	660	670	680	690	700
B26	CTGCTAGATG	AAAAAATCCG	AGAGTTTCAT	TGTCCAAGTG	TTTTAAACTC
EBB	CTGCTAGATG	AAAAAATCCG	AGAGTTTCAT	TGTCCAAGTG	TTTTAAACTC
LPCoLN	CTGCTAGATG	AAAAAATCCG	AGAGTTTCAT	TGTCCAAGTG	TTTTAAACTC
AR39	CTGGTAGATG	AAAAAATCCG	AGAGTTTCGT	TGTCCAAGTA	TTTTAAACTC
CWL029	CTGGTAGATG	AAAAAATCCG	AGAGTTTCGT	TGTCCAAGTA	TTTTAAACTC
J138	CTGGTAGATG	AAAAAATCCG	AGAGTTTCGT	TGTCCAAGTA	TTTTAAACTC
TW183	CTGGTAGATG	AAAAAATCCG	AGAGTTTCGT	TGTCCAAGTA	TTTTAAACTC
TOR1	CTGGTAGATG	AAAAAATCCG	AGAGTTTCGT	TGTCCAAGTA	TTTTAAACTC
WA97001	CTGGTAGATG	AAAAAATCCG	AGAGTTTCGT	TGTCCAAGTA	TTTTAAACTC
1979	CTGGTAGATG	AAAAAATCCG	AGAGTTTCGT	TGTCCAAGTA	TTTTAAACTC
SH511	CTGGTAGATG	AAAAAATCCG	AGAGTTTCGT	TGTCCAAGTA	TTTTAAACTC

	710	720	730	740	750
B26	AGCAGTATGT	GATATAATAG	ATCACAAATG	CCAAGAACAT	TTGTTAAAGG
EBB	AGCAGTATGT	GATATAATAG	ATCACAAATG	CCAAGAACAT	TTGTTAAAGG
LPCoLN	AGCAGTATGT	GATATAATAG	ATCACAAATG	CCAAGAACAT	TTGTTAAAGG
AR39	AGCAGTATGT	GATGTAATAG	ATCGCAAATG	CCAAGAACAT	TTGTTAAAGG
CWL029	AGCAGTATGT	GATGTAATAG	ATCGCAAATG	CCAAGAACAT	TTGTTAAAGG
J138	AGCAGTATGT	GATGTAATAG	ATCGCAAATG	CCAAGAACAT	TTGTTAAAGG
TW183	AGCAGTATGT	GATGTAATAG	ATCGCAAATG	CCAAGAACAT	TTGTTAAAGG
TOR1	AGCAGTATGT	GATGTAATAG	ATCGCAAATG	CCAAGAACAT	TTGTTAAAGG
WA97001	AGCAGTATGT	GATGTAATAG	ATCGCAAATG	CCAAGAACAT	TTGTTAAAGG
1979	AGCAGTATGT	GATATAATAG	ATCGCAAATG	CCAAGAACAT	TTGTTAAAGG
SH511	AGCAGTATGT	GATATAATAG	ATCGCAAATG	CCAAGAACAT	TTGTTAAAGG

	760	770	780	790	800
B26	CAATAATAAA	CGAAGCAAAC	CGCAGGCTCC	CTGGGATGAA	AAATAGCTCA
EBB	CAATAATAAA	CGAAGCAAAC	CGCAGGCTCC	CTGGGATGAA	AAATAGCTCA
LPCoLN	CAATAATAAA	CGAAGCAAAC	CGCAGGCTCC	CTGGGATGAA	AAATAGCTCA
AR39	CAATAATAAA	CGAAGCAAAC	CGCAGGCTCC	CTGGGATGAA	AAATAGCTCA
CWL029	CAATAATAAA	CGAAGCAAAC	CGCAGGCTCC	CTGGGATGAA	AAATAGCTCA
J138	CAATAATAAA	CGAAGCAAAC	CGCAGGCTCC	CTGGGATGAA	AAATAGCTCA
TW183	CAATAATAAA	CGAAGCAAAC	CGCAGGCTCC	CTGGGATGAA	AAATAGCTCA
TOR1	CAATAATAAA	CGAAGCAAAC	CGCAGGCTCC	CTGGGATGAA	AAATAGCTCA
WA97001	CAATAATAAA	CGAAGCAAAC	CGCAGGCTCC	CTGGGATGAA	AAATAGCTCA
1979	CAATAATAAA	CGAAGCAAAC	CGCAGGCTCC	CTGGGATGAA	AAATAGCTCA
SH511	CAATAATAAA	CGAAGCAAAC	CGCAGGCTCC	CTGGGATGAA	AAATAGCTCA

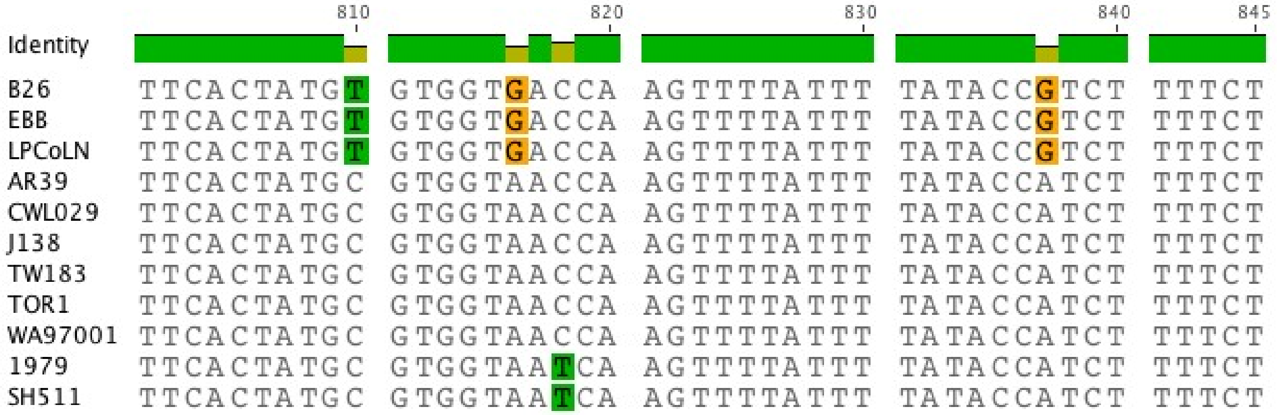
810
820
830
840
845
Identity
B26 TTCACTATGT GTGGTGACCA AGTTTTATTT TATACCGTCT TTTCT
EBB TTCACTATGT GTGGTGACCA AGTTTTATTT TATACCGTCT TTTCT
LPCoLN TTCACTATGT GTGGTGACCA AGTTTTATTT TATACCGTCT TTTCT
AR39 TTCACTATGC GTGGTAACCA AGTTTTATTT TATACCATCT TTTCT
CWL029 TTCACTATGC GTGGTAACCA AGTTTTATTT TATACCATCT TTTCT
J138 TTCACTATGC GTGGTAACCA AGTTTTATTT TATACCATCT TTTCT
TW183 TTCACTATGC GTGGTAACCA AGTTTTATTT TATACCATCT TTTCT
TOR1 TTCACTATGC GTGGTAACCA AGTTTTATTT TATACCATCT TTTCT
WA97001 TTCACTATGC GTGGTAACCA AGTTTTATTT TATACCATCT TTTCT
1979 TTCACTATGC GTGGTAATCA AGTTTTATTT TATACCATCT TTTCT
SH511 TTCACTATGC GTGGTAATCA AGTTTTATTT TATACCATCT TTTCT

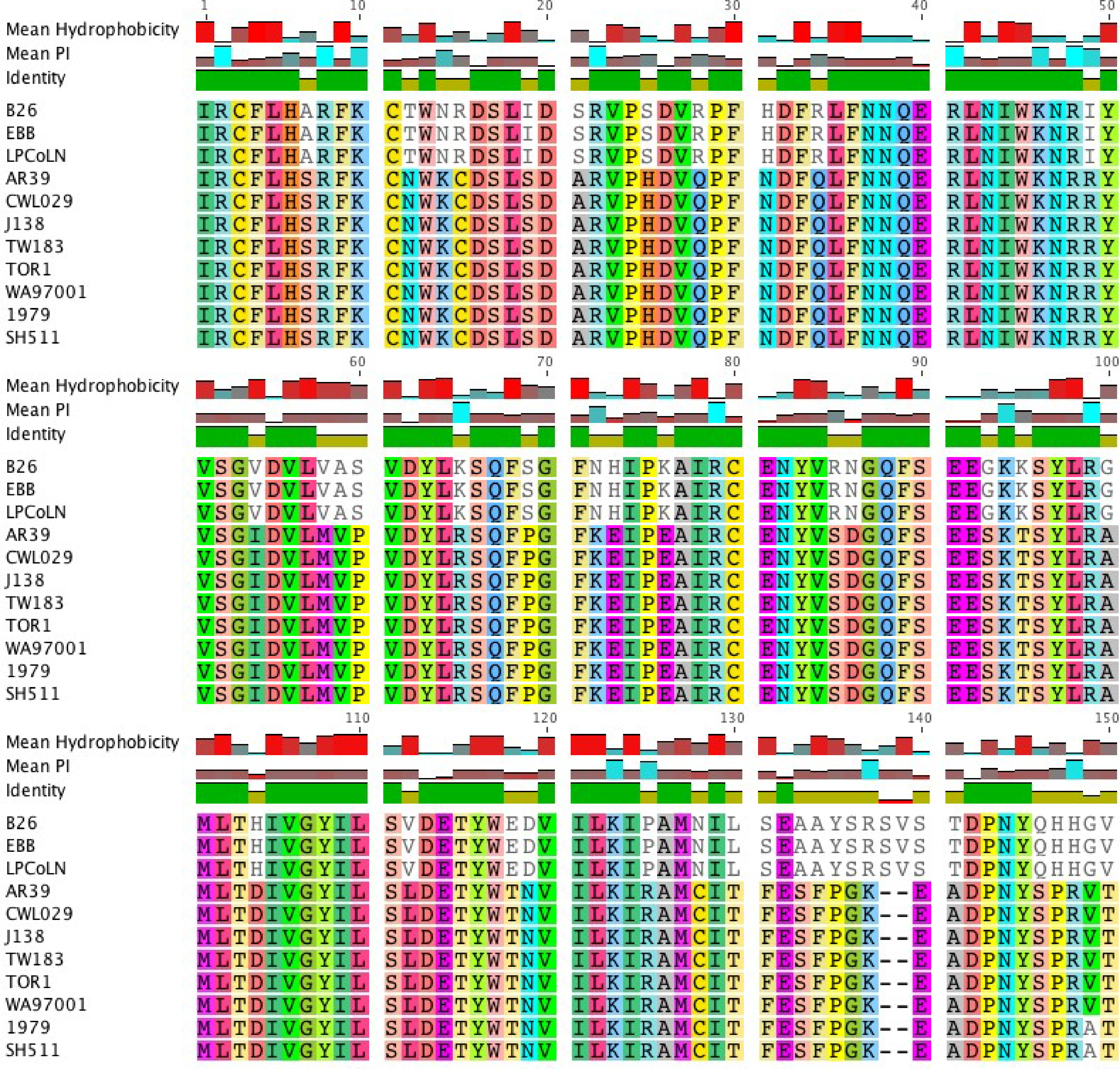

1 10 20 30 40 50
Mean Hydrophobicity
Mean PI
Identity
B26 IRCFLHARFK CTWNRDSLID SRVPSDVRPF HDFRLFNNQE RLNIWKNRIY
EBB IRCFLHARFK CTWNRDSLID SRVPSDVRPF HDFRLFNNQE RLNIWKNRIY
LPCoLN IRCFLHARFK CTWNRDSLID SRVPSDVRPF HDFRLFNNQE RLNIWKNRIY
AR39 IRCFLHSRFK CNWKCDSLSD ARVPHDVQPF NDFQLFNNQE RLNIWKNRRY
CWL029 IRCFLHSRFK CNWKCDSLSD ARVPHDVQPF NDFQLFNNQE RLNIWKNRRY
J138 IRCFLHSRFK CNWKCDSLSD ARVPHDVQPF NDFQLFNNQE RLNIWKNRRY
TW183 IRCFLHSRFK CNWKCDSLSD ARVPHDVQPF NDFQLFNNQE RLNIWKNRRY
TOR1 IRCFLHSRFK CNWKCDSLSD ARVPHDVQPF NDFQLFNNQE RLNIWKNRRY
WA97001 IRCFLHSRFK CNWKCDSLSD ARVPHDVQPF NDFQLFNNQE RLNIWKNRRY
1979 IRCFLHSRFK CNWKCDSLSD ARVPHDVQPF NDFQLFNNQE RLNIWKNRRY
SH511 IRCFLHSRFK CNWKCDSLSD ARVPHDVQPF NDFQLFNNQE RLNIWKNRRY
60 70 80 90 100
Mean Hydrophobicity
Mean PI
Identity
B26 VSGVDVLVAS VDYLKSQFSG FNHIPKAIRC ENYVRNGQFS EEGKKSYLRG
EBB VSGVDVLVAS VDYLKSQFSG FNHIPKAIRC ENYVRNGQFS EEGKKSYLRG
LPCoLN VSGVDVLVAS VDYLKSQFSG FNHIPKAIRC ENYVRNGQFS EEGKKSYLRG
AR39 VSGIDVLMVP VDYLRSQFPG FKEIPEAIRC ENYVSDGQFS EESKTSYLRA
CWL029 VSGIDVLMVP VDYLRSQFPG FKEIPEAIRC ENYVSDGQFS EESKTSYLRA
J138 VSGIDVLMVP VDYLRSQFPG FKEIPEAIRC ENYVSDGQFS EESKTSYLRA
TW183 VSGIDVLMVP VDYLRSQFPG FKEIPEAIRC ENYVSDGQFS EESKTSYLRA
TOR1 VSGIDVLMVP VDYLRSQFPG FKEIPEAIRC ENYVSDGQFS EESKTSYLRA
WA97001 VSGIDVLMVP VDYLRSQFPG FKEIPEAIRC ENYVSDGQFS EESKTSYLRA
1979 VSGIDVLMVP VDYLRSQFPG FKEIPEAIRC ENYVSDGQFS EESKTSYLRA
SH511 VSGIDVLMVP VDYLRSQFPG FKEIPEAIRC ENYVSDGQFS EESKTSYLRA
110 120 130 140 150
Mean Hydrophobicity
Mean PI
Identity
B26 MLTHIVGYIL SVDETYWEDV ILKIPAMNIL SEAAYSRSVS TDPNYQHHGV
EBB MLTHIVGYIL SVDETYWEDV ILKIPAMNIL SEAAYSRSVS TDPNYQHHGV
LPCoLN MLTHIVGYIL SVDETYWEDV ILKIPAMNIL SEAAYSRSVS TDPNYQHHGV
AR39 MLTDIVGYIL SLDETYWTNV ILKIRAMCIT FESFPGK--E ADPNYSPRVT
CWL029 MLTDIVGYIL SLDETYWTNV ILKIRAMCIT FESFPGK--E ADPNYSPRVT
J138 MLTDIVGYIL SLDETYWTNV ILKIRAMCIT FESFPGK--E ADPNYSPRVT
TW183 MLTDIVGYIL SLDETYWTNV ILKIRAMCIT FESFPGK--E ADPNYSPRVT
TOR1 MLTDIVGYIL SLDETYWTNV ILKIRAMCIT FESFPGK--E ADPNYSPRVT
WA97001 MLTDIVGYIL SLDETYWTNV ILKIRAMCIT FESFPGK--E ADPNYSPRVT
1979 MLTDIVGYIL SLDETYWTNV ILKIRAMCIT FESFPGK--E ADPNYSPRAT
SH511 MLTDIVGYIL SLDETYWTNV ILKIRAMCIT FESFPGK--E ADPNYSPRAT

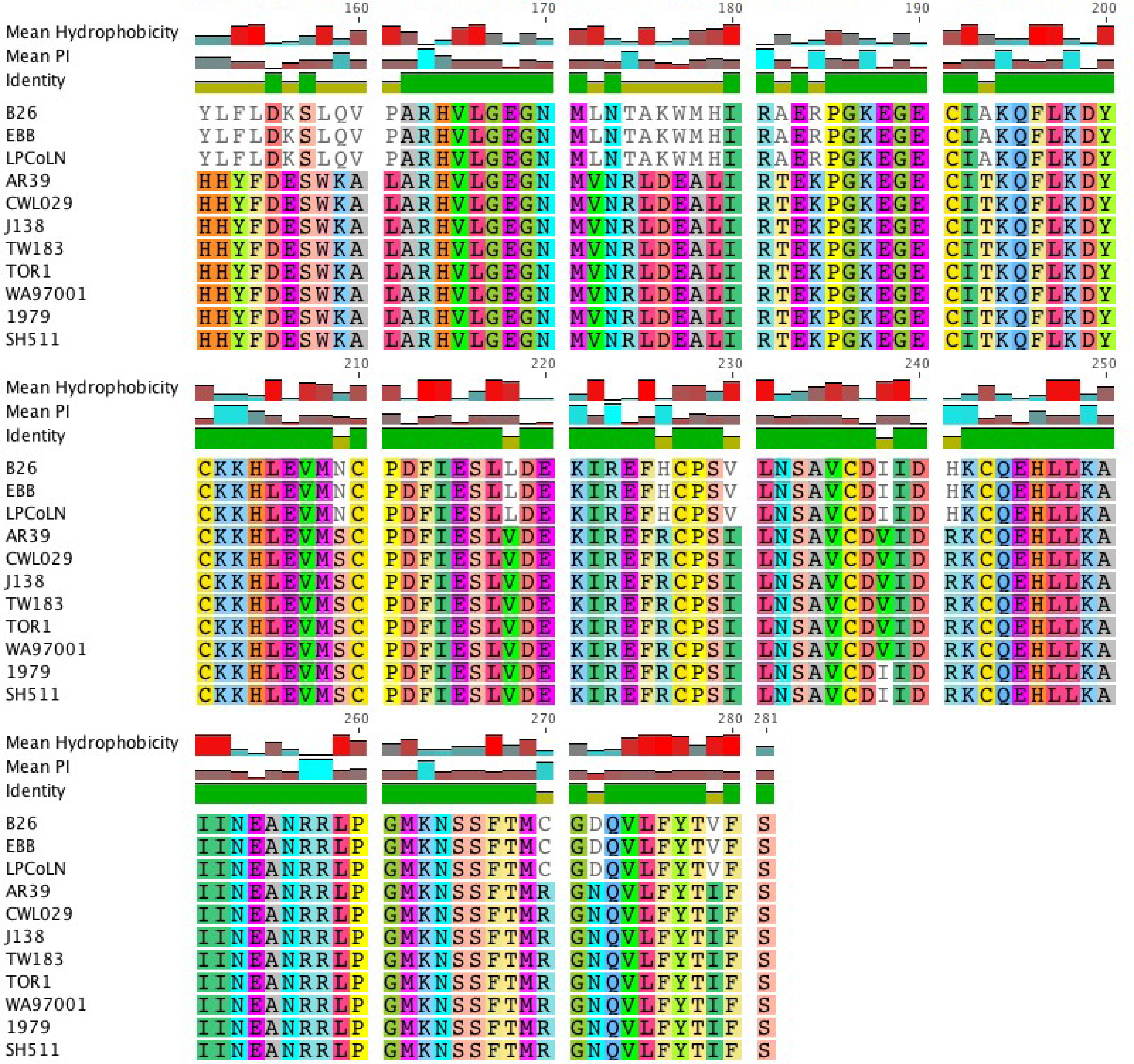
160 170 180 190 200
Mean Hydrophobicity
Mean PI
Identity
B26 YLFLDKSLQV PARHVLGEGN MLNTAKWMHI RAERPGKEGE CIAKQFLKDY
EBB YLFLDKSLQV PARHVLGEGN MLNTAKWMHI RAERPGKEGE CIAKQFLKDY
LPCoLN YLFLDKSLQV PARHVLGEGN MLNTAKWMHI RAERPGKEGE CIAKQFLKDY
AR39 HHYFDESWKA LARHVLGEGN MVNRLDEALI RTEKPGKEGE CITKQFLKDY
CWL029 HHYFDESWKA LARHVLGEGN MVNRLDEALI RTEKPGKEGE CITKQFLKDY
J138 HHYFDESWKA LARHVLGEGN MVNRLDEALI RTEKPGKEGE CITKQFLKDY
TW183 HHYFDESWKA LARHVLGEGN MVNRLDEALI RTEKPGKEGE CITKQFLKDY
TOR1 HHYFDESWKA LARHVLGEGN MVNRLDEALI RTEKPGKEGE CITKQFLKDY
WA97001 HHYFDESWKA LARHVLGEGN MVNRLDEALI RTEKPGKEGE CITKQFLKDY
1979 HHYFDESWKA LARHVLGEGN MVNRLDEALI RTEKPGKEGE CITKQFLKDY
SH511 HHYFDESWKA LARHVLGEGN MVNRLDEALI RTEKPGKEGE CITKQFLKDY
210 220 230 240 250
Mean Hydrophobicity
Mean PI
Identity
B26 CKKHLEVMNC PDFIESLLDE KIREFHCPSV LNSAVCDIID HKCQEHLLKA
EBB CKKHLEVMNC PDFIESLLDE KIREFHCPSV LNSAVCDIID HKCQEHLLKA
LPCoLN CKKHLEVMNC PDFIESLLDE KIREFHCPSV LNSAVCDIID HKCQEHLLKA
AR39 CKKHLEVMSC PDFIESLVDE KIREFRCPSI LNSAVCDVID RKCQEHLLKA
CWL029 CKKHLEVMSC PDFIESLVDE KIREFRCPSI LNSAVCDVID RKCQEHLLKA
J138 CKKHLEVMSC PDFIESLVDE KIREFRCPSI LNSAVCDVID RKCQEHLLKA
TW183 CKKHLEVMSC PDFIESLVDE KIREFRCPSI LNSAVCDVID RKCQEHLLKA
TOR1 CKKHLEVMSC PDFIESLVDE KIREFRCPSI LNSAVCDVID RKCQEHLLKA
WA97001 CKKHLEVMSC PDFIESLVDE KIREFRCPSI LNSAVCDVID RKCQEHLLKA
1979 CKKHLEVMSC PDFIESLVDE KIREFRCPSI LNSAVCDIID RKCQEHLLKA
SH511 CKKHLEVMSC PDFIESLVDE KIREFRCPSI LNSAVCDIID RKCQEHLLKA
260 270 280 281
Mean Hydrophobicity
Mean PI
Identity
B26 IINEANRRLP GMKNSSFTMC GDQVLFYTVF S
EBB IINEANRRLP GMKNSSFTMC GDQVLFYTVF S
LPCoLN IINEANRRLP GMKNSSFTMC GDQVLFYTVF S
AR39 IINEANRRLP GMKNSSFTMR GNQVLFYTIF S
CWL029 IINEANRRLP GMKNSSFTMR GNQVLFYTIF S
J138 IINEANRRLP GMKNSSFTMR GNQVLFYTIF S
TW183 IINEANRRLP GMKNSSFTMR GNQVLFYTIF S
TOR1 IINEANRRLP GMKNSSFTMR GNQVLFYTIF S
WA97001 IINEANRRLP GMKNSSFTMR GNQVLFYTIF S
1979 IINEANRRLP GMKNSSFTMR GNQVLFYTIF S
SH511 IINEANRRLP GMKNSSFTMR GNQVLFYTIF S
