# Supplementary material for: Chlamydia pneumoniae Is Genetically Diverse in Animals and Appears to Have Crossed the Host Barrier to Humans on (At Least) Two Occasions
Source: PLoS Pathog. 2010 May 20;6(5):e1000903. doi: 10.1371/journal.ppat.1000903 (PMC2873915; doi:10.1371/journal.ppat.1000903)
Supplement: Figure S14 — Multiple sequence alignment of CP_0505. There are four features of interest: (i) 13 SNPs unique to koala LPCoLN and bandicoots B26 and B37, (ii) 42 SNPs unique to horse N16, (iii) two SNPs unique to Australian Indigenous SH511 and 1979 isolates, and a third sSNP is shared with horse N16, koala LPCoLN, bandicoot B26 and bandicoot B37 isolates, and (iv) a single bp deletion distinguishing frog DE177 from the non-Indigenous isolates (identical sequence). (5.43 MB PDF) [file ppat.1000903.s014.pdf]

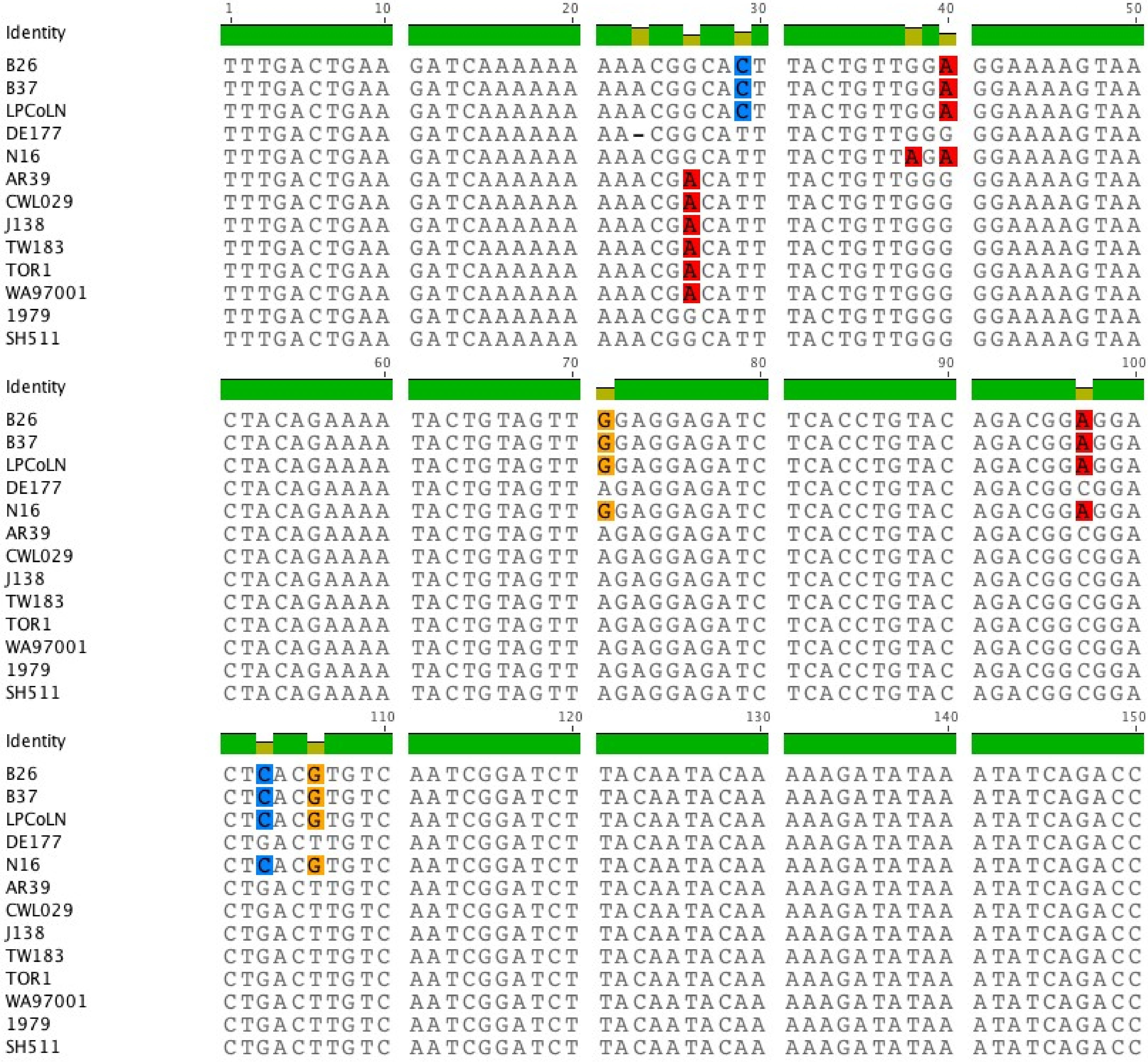
1 10 20 30 40 50
Identity
B26 TTTGACTGAA GATCAAAAAA AAACGGCACT TACTGTTGGA GGAAAAGTAA
B37 TTTGACTGAA GATCAAAAAA AAACGGCACT TACTGTTGGA GGAAAAGTAA
LPCoLN TTTGACTGAA GATCAAAAAA AAACGGCACT TACTGTTGGA GGAAAAGTAA
DE177 TTTGACTGAA GATCAAAAAA AA-CGGCATT TACTGTTGGG GGAAAAGTAA
N16 TTTGACTGAA GATCAAAAAA AAACGGCATT TACTGTTAGA GGAAAAGTAA
AR39 TTTGACTGAA GATCAAAAAA AAACGACATT TACTGTTGGG GGAAAAGTAA
CWL029 TTTGACTGAA GATCAAAAAA AAACGACATT TACTGTTGGG GGAAAAGTAA
J138 TTTGACTGAA GATCAAAAAA AAACGACATT TACTGTTGGG GGAAAAGTAA
TW183 TTTGACTGAA GATCAAAAAA AAACGACATT TACTGTTGGG GGAAAAGTAA
TOR1 TTTGACTGAA GATCAAAAAA AAACGACATT TACTGTTGGG GGAAAAGTAA
WA97001 TTTGACTGAA GATCAAAAAA AAACGACATT TACTGTTGGG GGAAAAGTAA
1979 TTTGACTGAA GATCAAAAAA AAACGGCATT TACTGTTGGG GGAAAAGTAA
SH511 TTTGACTGAA GATCAAAAAA AAACGGCATT TACTGTTGGG GGAAAAGTAA
60 70 80 90 100
Identity
B26 CTACAGAAAA TACTGTAGTT GGAGGAGATC TCACCTGTAC AGACGGAGGA
B37 CTACAGAAAA TACTGTAGTT GGAGGAGATC TCACCTGTAC AGACGGAGGA
LPCoLN CTACAGAAAA TACTGTAGTT GGAGGAGATC TCACCTGTAC AGACGGAGGA
DE177 CTACAGAAAA TACTGTAGTT AGAGGAGATC TCACCTGTAC AGACGGCGGA
N16 CTACAGAAAA TACTGTAGTT GGAGGAGATC TCACCTGTAC AGACGGAGGA
AR39 CTACAGAAAA TACTGTAGTT AGAGGAGATC TCACCTGTAC AGACGGCGGA
CWL029 CTACAGAAAA TACTGTAGTT AGAGGAGATC TCACCTGTAC AGACGGCGGA
J138 CTACAGAAAA TACTGTAGTT AGAGGAGATC TCACCTGTAC AGACGGCGGA
TW183 CTACAGAAAA TACTGTAGTT AGAGGAGATC TCACCTGTAC AGACGGCGGA
TOR1 CTACAGAAAA TACTGTAGTT AGAGGAGATC TCACCTGTAC AGACGGCGGA
WA97001 CTACAGAAAA TACTGTAGTT AGAGGAGATC TCACCTGTAC AGACGGCGGA
1979 CTACAGAAAA TACTGTAGTT AGAGGAGATC TCACCTGTAC AGACGGCGGA
SH511 CTACAGAAAA TACTGTAGTT AGAGGAGATC TCACCTGTAC AGACGGCGGA
110 120 130 140 150
Identity
B26 CTCACGTGTC AATCGGATCT TACAATACAA AAAGATATAA ATATCAGACC
B37 CTCACGTGTC AATCGGATCT TACAATACAA AAAGATATAA ATATCAGACC
LPCoLN CTCACGTGTC AATCGGATCT TACAATACAA AAAGATATAA ATATCAGACC
DE177 CTGACTTGTC AATCGGATCT TACAATACAA AAAGATATAA ATATCAGACC
N16 CTCACGTGTC AATCGGATCT TACAATACAA AAAGATATAA ATATCAGACC
AR39 CTGACTTGTC AATCGGATCT TACAATACAA AAAGATATAA ATATCAGACC
CWL029 CTGACTTGTC AATCGGATCT TACAATACAA AAAGATATAA ATATCAGACC
J138 CTGACTTGTC AATCGGATCT TACAATACAA AAAGATATAA ATATCAGACC
TW183 CTGACTTGTC AATCGGATCT TACAATACAA AAAGATATAA ATATCAGACC
TOR1 CTGACTTGTC AATCGGATCT TACAATACAA AAAGATATAA ATATCAGACC
WA97001 CTGACTTGTC AATCGGATCT TACAATACAA AAAGATATAA ATATCAGACC
1979 CTGACTTGTC AATCGGATCT TACAATACAA AAAGATATAA ATATCAGACC
SH511 CTGACTTGTC AATCGGATCT TACAATACAA AAAGATATAA ATATCAGACC

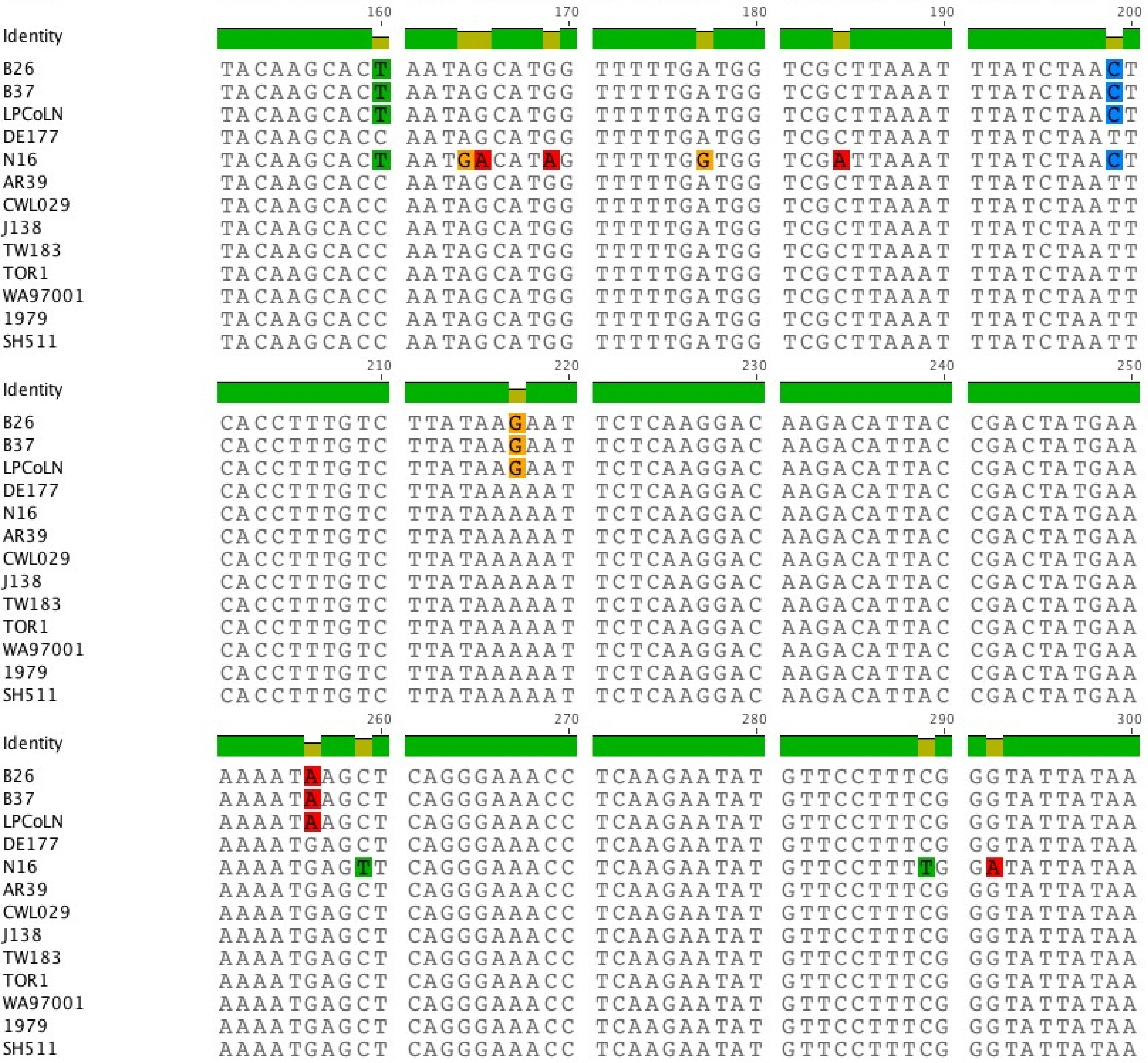

160 170 180 190 200
Identity
B26 TACAAGCACT AATAGCATGG TTTTTGATGG TCGCTTAAAT TTATCTAACT
B37 TACAAGCACT AATAGCATGG TTTTTGATGG TCGCTTAAAT TTATCTAACT
LPCoLN TACAAGCACT AATAGCATGG TTTTTGATGG TCGCTTAAAT TTATCTAACT
DE177 TACAAGCACC AATAGCATGG TTTTTGATGG TCGCTTAAAT TTATCTAATT
N16 TACAAGCACT AATGACATAG TTTTTGGTGG TCGATTAAAT TTATCTAACT
AR39 TACAAGCACC AATAGCATGG TTTTTGATGG TCGCTTAAAT TTATCTAATT
CWL029 TACAAGCACC AATAGCATGG TTTTTGATGG TCGCTTAAAT TTATCTAATT
J138 TACAAGCACC AATAGCATGG TTTTTGATGG TCGCTTAAAT TTATCTAATT
TW183 TACAAGCACC AATAGCATGG TTTTTGATGG TCGCTTAAAT TTATCTAATT
TOR1 TACAAGCACC AATAGCATGG TTTTTGATGG TCGCTTAAAT TTATCTAATT
WA97001 TACAAGCACC AATAGCATGG TTTTTGATGG TCGCTTAAAT TTATCTAATT
1979 TACAAGCACC AATAGCATGG TTTTTGATGG TCGCTTAAAT TTATCTAATT
SH511 TACAAGCACC AATAGCATGG TTTTTGATGG TCGCTTAAAT TTATCTAATT
210 220 230 240 250
Identity
B26 CACCTTTGTC TTATAAGAAT TCTCAAGGAC AAGACATTAC CGACTATGAA
B37 CACCTTTGTC TTATAAGAAT TCTCAAGGAC AAGACATTAC CGACTATGAA
LPCoLN CACCTTTGTC TTATAAGAAT TCTCAAGGAC AAGACATTAC CGACTATGAA
DE177 CACCTTTGTC TTATAAAAAT TCTCAAGGAC AAGACATTAC CGACTATGAA
N16 CACCTTTGTC TTATAAAAAT TCTCAAGGAC AAGACATTAC CGACTATGAA
AR39 CACCTTTGTC TTATAAAAAT TCTCAAGGAC AAGACATTAC CGACTATGAA
CWL029 CACCTTTGTC TTATAAAAAT TCTCAAGGAC AAGACATTAC CGACTATGAA
J138 CACCTTTGTC TTATAAAAAT TCTCAAGGAC AAGACATTAC CGACTATGAA
TW183 CACCTTTGTC TTATAAAAAT TCTCAAGGAC AAGACATTAC CGACTATGAA
TOR1 CACCTTTGTC TTATAAAAAT TCTCAAGGAC AAGACATTAC CGACTATGAA
WA97001 CACCTTTGTC TTATAAAAAT TCTCAAGGAC AAGACATTAC CGACTATGAA
1979 CACCTTTGTC TTATAAAAAT TCTCAAGGAC AAGACATTAC CGACTATGAA
SH511 CACCTTTGTC TTATAAAAAT TCTCAAGGAC AAGACATTAC CGACTATGAA
260 270 280 290 300
Identity
B26 AAAATAAGCT CAGGGAAACC TCAAGAATAT GTTCCTTTCG GGTATTATAA
B37 AAAATAAGCT CAGGGAAACC TCAAGAATAT GTTCCTTTCG GGTATTATAA
LPCoLN AAAATAAGCT CAGGGAAACC TCAAGAATAT GTTCCTTTCG GGTATTATAA
DE177 AAAATGAGCT CAGGGAAACC TCAAGAATAT GTTCCTTTCG GGTATTATAA
N16 AAAATGAGTT CAGGGAAACC TCAAGAATAT GTTCCTTTTG GATATTATAA
AR39 AAAATGAGCT CAGGGAAACC TCAAGAATAT GTTCCTTTCG GGTATTATAA
CWL029 AAAATGAGCT CAGGGAAACC TCAAGAATAT GTTCCTTTCG GGTATTATAA
J138 AAAATGAGCT CAGGGAAACC TCAAGAATAT GTTCCTTTCG GGTATTATAA
TW183 AAAATGAGCT CAGGGAAACC TCAAGAATAT GTTCCTTTCG GGTATTATAA
TOR1 AAAATGAGCT CAGGGAAACC TCAAGAATAT GTTCCTTTCG GGTATTATAA
WA97001 AAAATGAGCT CAGGGAAACC TCAAGAATAT GTTCCTTTCG GGTATTATAA
1979 AAAATGAGCT CAGGGAAACC TCAAGAATAT GTTCCTTTCG GGTATTATAA
SH511 AAAATGAGCT CAGGGAAACC TCAAGAATAT GTTCCTTTCG GGTATTATAA

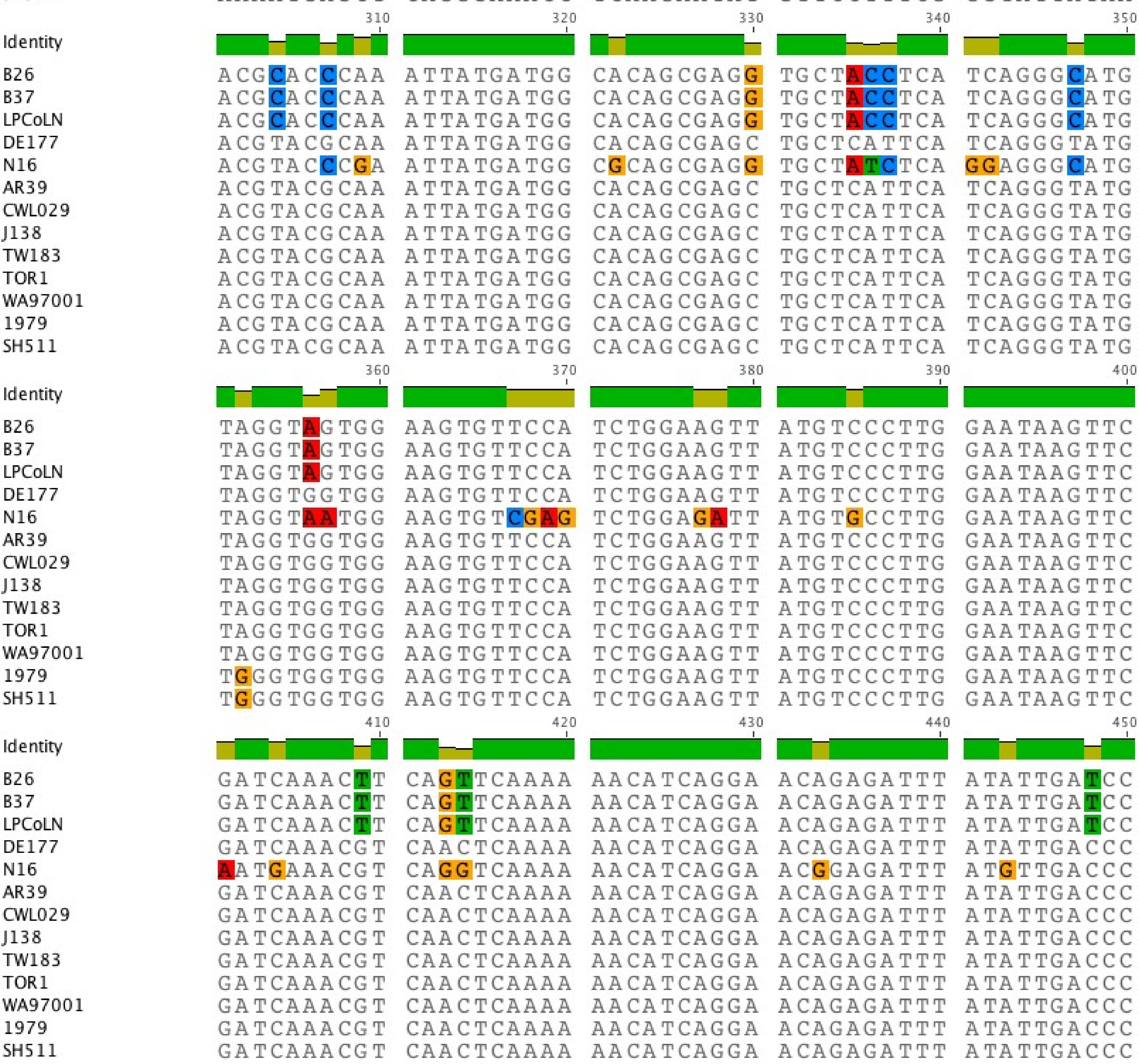
310 320 330 340 350
Identity
B26 ACGCACCCAA ATTATGATGG CACAGCGAGG TGCTACCTCA TCAGGGCATG
B37 ACGCACCCAA ATTATGATGG CACAGCGAGG TGCTACCTCA TCAGGGCATG
LPCoLN ACGCACCCAA ATTATGATGG CACAGCGAGG TGCTACCTCA TCAGGGCATG
DE177 ACGTACGCAA ATTATGATGG CACAGCGAGC TGCTCATTCA TCAGGGTATG
N16 ACGTACCCGA ATTATGATGG CGCAGCGAGG TGCTATCTCA GGAGGGCATG
AR39 ACGTACGCAA ATTATGATGG CACAGCGAGC TGCTCATTCA TCAGGGTATG
CWL029 ACGTACGCAA ATTATGATGG CACAGCGAGC TGCTCATTCA TCAGGGTATG
J138 ACGTACGCAA ATTATGATGG CACAGCGAGC TGCTCATTCA TCAGGGTATG
TW183 ACGTACGCAA ATTATGATGG CACAGCGAGC TGCTCATTCA TCAGGGTATG
TOR1 ACGTACGCAA ATTATGATGG CACAGCGAGC TGCTCATTCA TCAGGGTATG
WA97001 ACGTACGCAA ATTATGATGG CACAGCGAGC TGCTCATTCA TCAGGGTATG
1979 ACGTACGCAA ATTATGATGG CACAGCGAGC TGCTCATTCA TCAGGGTATG
SH511 ACGTACGCAA ATTATGATGG CACAGCGAGC TGCTCATTCA TCAGGGTATG
360 370 380 390 400
Identity
B26 TAGGTAGTGG AAGTGTTCCA TCTGGAAGTT ATGTCCCTTG GAATAAGTTC
B37 TAGGTAGTGG AAGTGTTCCA TCTGGAAGTT ATGTCCCTTG GAATAAGTTC
LPCoLN TAGGTAGTGG AAGTGTTCCA TCTGGAAGTT ATGTCCCTTG GAATAAGTTC
DE177 TAGGTGGTGG AAGTGTTCCA TCTGGAAGTT ATGTCCCTTG GAATAAGTTC
N16 TAGGTAATGG AAGTGTCGAG TCTGGAGATT ATGTGCCTTG GAATAAGTTC
AR39 TAGGTGGTGG AAGTGTTCCA TCTGGAAGTT ATGTCCCTTG GAATAAGTTC
CWL029 TAGGTGGTGG AAGTGTTCCA TCTGGAAGTT ATGTCCCTTG GAATAAGTTC
J138 TAGGTGGTGG AAGTGTTCCA TCTGGAAGTT ATGTCCCTTG GAATAAGTTC
TW183 TAGGTGGTGG AAGTGTTCCA TCTGGAAGTT ATGTCCCTTG GAATAAGTTC
TOR1 TAGGTGGTGG AAGTGTTCCA TCTGGAAGTT ATGTCCCTTG GAATAAGTTC
WA97001 TAGGTGGTGG AAGTGTTCCA TCTGGAAGTT ATGTCCCTTG GAATAAGTTC
1979 TGGGTGGTGG AAGTGTTCCA TCTGGAAGTT ATGTCCCTTG GAATAAGTTC
SH511 TGGGTGGTGG AAGTGTTCCA TCTGGAAGTT ATGTCCCTTG GAATAAGTTC
410 420 430 440 450
Identity
B26 GATCAAACTT CAGTTCAAAA AACATCAGGA ACAGAGATTT ATATTGATCC
B37 GATCAAACTT CAGTTCAAAA AACATCAGGA ACAGAGATTT ATATTGATCC
LPCoLN GATCAAACTT CAGTTCAAAA AACATCAGGA ACAGAGATTT ATATTGATCC
DE177 GATCAAACGT CAACTCAAAA AACATCAGGA ACAGAGATTT ATATTGACCC
N16 AATGAAACGT CAGGTCAAAA AACATCAGGA ACGGAGATTT ATGTTGACCC
AR39 GATCAAACGT CAACTCAAAA AACATCAGGA ACAGAGATTT ATATTGACCC
CWL029 GATCAAACGT CAACTCAAAA AACATCAGGA ACAGAGATTT ATATTGACCC
J138 GATCAAACGT CAACTCAAAA AACATCAGGA ACAGAGATTT ATATTGACCC
TW183 GATCAAACGT CAACTCAAAA AACATCAGGA ACAGAGATTT ATATTGACCC
TOR1 GATCAAACGT CAACTCAAAA AACATCAGGA ACAGAGATTT ATATTGACCC
WA97001 GATCAAACGT CAACTCAAAA AACATCAGGA ACAGAGATTT ATATTGACCC
1979 GATCAAACGT CAACTCAAAA AACATCAGGA ACAGAGATTT ATATTGACCC
SH511 GATCAAACGT CAACTCAAAA AACATCAGGA ACAGAGATTT ATATTGACCC

Identity	460	470	480	490	500
B26	AAATGACGGA	ACTAAATTAG	TTTTCGAAGT	CAATAGTAAG	GTGCCAAAAC
B37	AAATGACGGA	ACTAAATTAG	TTTTCGAAGT	CAATAGTAAG	GTGCCAAAAC
LPCoLN	AAATGACGGA	ACTAAATTAG	TTTTCGAAGT	CAATAGTAAG	GTGCCAAAAC
DE177	AAATGACTCA	ACTAAATTGG	TTTTCGAAGT	AAATAATAAG	GTGCCAAAAC
N16	AAGTGAACCA	TATAAATTAG	TTTTCAAAGT	AAATACTAAG	ATACCAAAAC
AR39	AAATGACTCA	ACTAAATTGG	TTTTCGAAGT	AAATAATAAG	GTGCCAAAAC
CWL029	AAATGACTCA	ACTAAATTGG	TTTTCGAAGT	AAATAATAAG	GTGCCAAAAC
J138	AAATGACTCA	ACTAAATTGG	TTTTCGAAGT	AAATAATAAG	GTGCCAAAAC
TW183	AAATGACTCA	ACTAAATTGG	TTTTCGAAGT	AAATAATAAG	GTGCCAAAAC
TOR1	AAATGACTCA	ACTAAATTGG	TTTTCGAAGT	AAATAATAAG	GTGCCAAAAC
WA97001	AAATGACTCA	ACTAAATTGG	TTTTCGAAGT	AAATAATAAG	GTGCCAAAAC
1979	AAATGACACA	ACTAAATTGG	TTTTCGAAGT	AAATAATAAG	GTGCCAAAAC
SH511	AAATGACACA	ACTAAATTGG	TTTTCGAAGT	AAATAATAAG	GTGCCAAAAC

Identity	510	520	530	540	550
B26	TTTTTCGTAT	TAGTATTATT	ATGGCTAAAA	ATGGTAGTTG	GTTGGATAAC
B37	TTTTTCGTAT	TAGTATTATT	ATGGCTAAAA	ATGGTAGTTG	GTTGGATAAC
LPCoLN	TTTTTCGTAT	TAGTATTATT	ATGGCTAAAA	ATGGTAGTTG	GTTGGATAAC
DE177	TTTTTCGTAT	TAGTGTTATT	ATGGCTAAAC	ATGGTTCTTG	GTTGGATAAC
N16	TTTTTCGTAT	TAGTATTATT	ATGGCTAAAC	ATGGTAGTTG	GTTGGATAAC
AR39	TTTTTCGTAT	TAGTGTTATT	ATGGCTAAAC	ATGGTTCTTG	GTTGGATAAC
CWL029	TTTTTCGTAT	TAGTGTTATT	ATGGCTAAAC	ATGGTTCTTG	GTTGGATAAC
J138	TTTTTCGTAT	TAGTGTTATT	ATGGCTAAAC	ATGGTTCTTG	GTTGGATAAC
TW183	TTTTTCGTAT	TAGTGTTATT	ATGGCTAAAC	ATGGTTCTTG	GTTGGATAAC
TOR1	TTTTTCGTAT	TAGTGTTATT	ATGGCTAAAC	ATGGTTCTTG	GTTGGATAAC
WA97001	TTTTTCGTAT	TAGTGTTATT	ATGGCTAAAC	ATGGTTCTTG	GTTGGATAAC
1979	TTTTTCGTAT	TAGTGTTATT	ATGGCTAAAC	ATGGTTCTTG	GTTGGATAAC
SH511	TTTTTCGTAT	TAGTGTTATT	ATGGCTAAAC	ATGGTTCTTG	GTTGGATAAC

Identity	560	570	580	590	600
B26	GGAACAGGAG	CAGATGTTCT	CTTAGCAGCG	AATGAATATG	AGCAAAGCGG
B37	GGAACAGGAG	CAGATGTTCT	CTTAGCAGCG	AATGAATATG	AGCAAAGCGG
LPCoLN	GGAACAGGAG	CAGATGTTCT	CTTAGCAGCG	AATGAATATG	AGCAAAGCGG
DE177	GGAACAGGAG	CAGATATTCT	CTTAGCGGCG	AATGAATATG	AGCAAGGCGG
N16	GGAACAGGAG	CAGATGTTCT	CTTAGCAGCG	AATGAATATG	AGCAAAGCGG
AR39	GGAACAGGAG	CAGATATTCT	CTTAGCGGCG	AATGAATATG	AGCAAGGCGG
CWL029	GGAACAGGAG	CAGATATTCT	CTTAGCGGCG	AATGAATATG	AGCAAGGCGG
J138	GGAACAGGAG	CAGATATTCT	CTTAGCGGCG	AATGAATATG	AGCAAGGCGG
TW183	GGAACAGGAG	CAGATATTCT	CTTAGCGGCG	AATGAATATG	AGCAAGGCGG
TOR1	GGAACAGGAG	CAGATATTCT	CTTAGCGGCG	AATGAATATG	AGCAAGGCGG
WA97001	GGAACAGGAG	CAGATATTCT	CTTAGCGGCG	AATGAATATG	AGCAAGGCGG
1979	GGAACAGGAG	CAGATATTCT	CTTAGCGGCG	AATGAATATG	AGCAAGGCGG
SH511	GGAACAGGAG	CAGATATTCT	CTTAGCGGCG	AATGAATATG	AGCAAGGCGG

	610	620	630	640	650
Identity					
B26	AGGGAAGATA	AAAGTCACAG	AGCTTGCGAT	GACGAGCTCT	AGAGGAACAA
B37	AGGGAAGATA	AAAGTCACAG	AGCTTGCGAT	GACGAGCTCT	AGAGGAACAA
LPCoLN	AGGGAAGATA	AAAGTCACAG	AGCTTGCGAT	GACGAGCTCT	AGAGGAACAA
DE177	AGGGAGGATA	AATGTCACAG	ATCTTGCAAT	GACGACCTCT	AGAGGGTCCA
N16	AGTGAAGATA	AAAGTCACAG	AGCTTGCGAT	GACGAGCTCT	AGAGGAACAA
AR39	AGGGAGGATA	AATGTCACAG	ATCTTGCAAT	GACGACCTCT	AGAGGGTCCA
CWL029	AGGGAGGATA	AATGTCACAG	ATCTTGCAAT	GACGACCTCT	AGAGGGTCCA
J138	AGGGAGGATA	AATGTCACAG	ATCTTGCAAT	GACGACCTCT	AGAGGGTCCA
TW183	AGGGAGGATA	AATGTCACAG	ATCTTGCAAT	GACGACCTCT	AGAGGGTCCA
TOR1	AGGGAGGATA	AATGTCACAG	ATCTTGCAAT	GACGACCTCT	AGAGGGTCCA
WA97001	AGGGAGGATA	AATGTCACAG	ATCTTGCAAT	GACGACCTCT	AGAGGGTCCA
1979	AGGGAGGATA	AATGTCACAG	ATCTTGCAAT	GACGACCTCT	AGAGGGTCCA
SH511	AGGGAGGATA	AATGTCACAG	ATCTTGCAAT	GACGACCTCT	AGAGGGTCCA

	660	670	680	690	700
Identity					
B26	GTTATTATGA	AACACGACCT	TTACAGGTAG	TTTGCGTTAC	ATACTATGCT
B37	GTTATTATGA	AACACGACCT	TTACAGGTAG	TTTGCGTTAC	ATACTATGCT
LPCoLN	GTTATTATGA	AACACGACCT	TTACAGGTAG	TTTGCGTTAC	ATACTATGCT
DE177	GTTATTATGA	AACACGTCCT	TTACAGGTAG	TTTGCGTTAC	ATACTATGCT
N16	GTTATTATGA	AGCACGACCT	TTACAGGTAG	TTTGCGTTGT	GTATTATGCT
AR39	GTTATTATGA	AACACGTCCT	TTACAGGTAG	TTTGCGTTAC	ATACTATGCT
CWL029	GTTATTATGA	AACACGTCCT	TTACAGGTAG	TTTGCGTTAC	ATACTATGCT
J138	GTTATTATGA	AACACGTCCT	TTACAGGTAG	TTTGCGTTAC	ATACTATGCT
TW183	GTTATTATGA	AACACGTCCT	TTACAGGTAG	TTTGCGTTAC	ATACTATGCT
TOR1	GTTATTATGA	AACACGTCCT	TTACAGGTAG	TTTGCGTTAC	ATACTATGCT
WA97001	GTTATTATGA	AACACGTCCT	TTACAGGTAG	TTTGCGTTAC	ATACTATGCT
1979	GTTATTATGA	AACACGTCCT	TTACAGGTAG	TTTGCGTTAC	ATACTATGCT
SH511	GTTATTATGA	AACACGTCCT	TTACAGGTAG	TTTGCGTTAC	ATACTATGCT

	710	720	723
Identity			
B26	AAAAATAATG	GTTACTTTAC	TTT
B37	AAAAATAATG	GTTACTTTAC	TTT
LPCoLN	AAAAATAATG	GTTACTTTAC	TTT
DE177	CAAAATAATG	GTTACTTTAC	TTT
N16	AAAAATAATG	GTTACTTTAC	TTT
AR39	CAAAATAATG	GTTACTTTAC	TTT
CWL029	CAAAATAATG	GTTACTTTAC	TTT
J138	CAAAATAATG	GTTACTTTAC	TTT
TW183	CAAAATAATG	GTTACTTTAC	TTT
TOR1	CAAAATAATG	GTTACTTTAC	TTT
WA97001	CAAAATAATG	GTTACTTTAC	TTT
1979	CAAAATAATG	GTTACTTTAC	TTT
SH511	CAAAATAATG	GTTACTTTAC	TTT

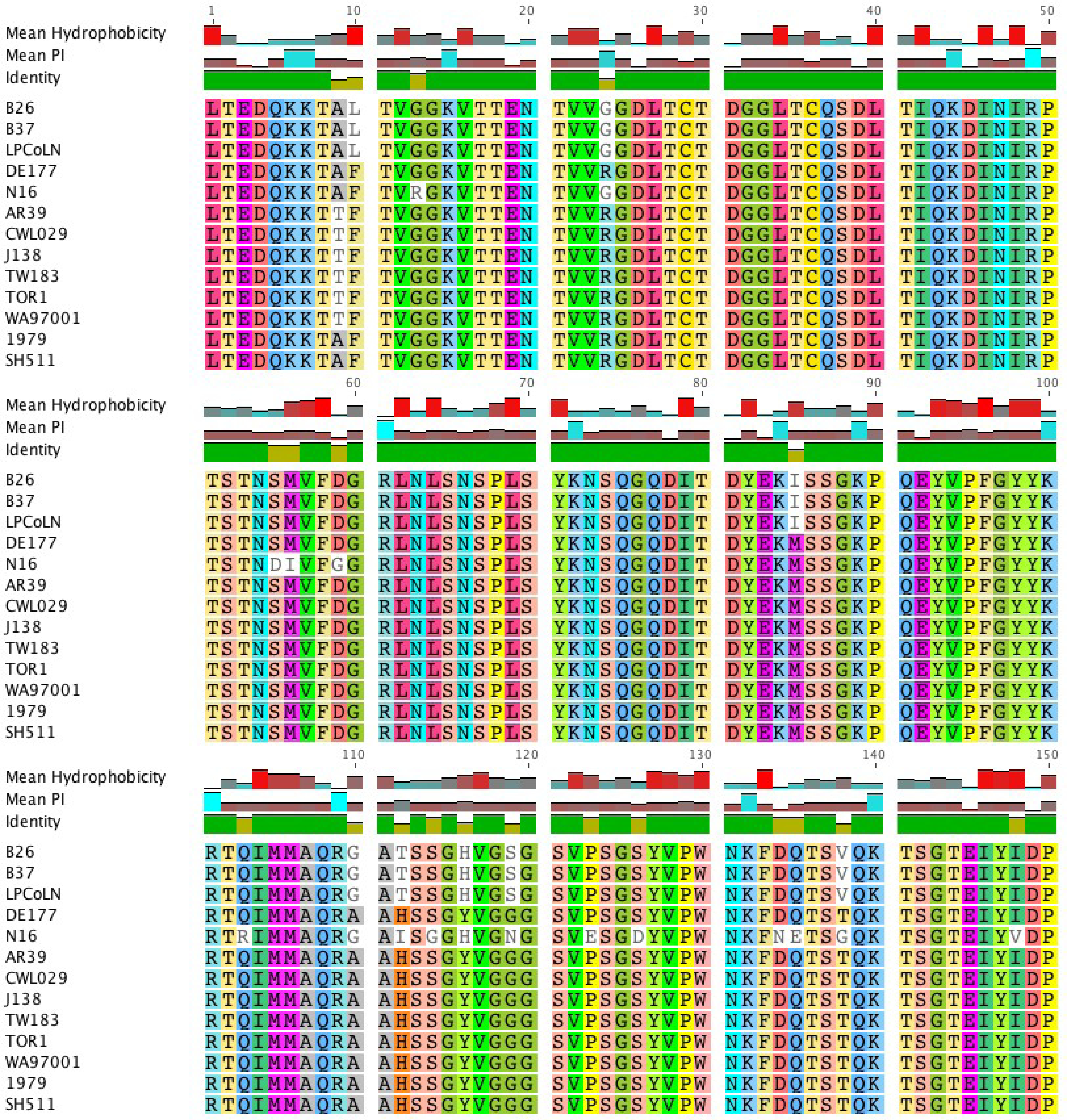

1 10 20 30 40 50
Mean Hydrophobicity
Mean PI
Identity
B26 LTEDQKKTAL TVGGKVTTEN TVVGGDLTCT DGGLTCQSDL TIQKDINIRP
B37 LTEDQKKTAL TVGGKVTTEN TVVGGDLTCT DGGLTCQSDL TIQKDINIRP
LPCoLN LTEDQKKTAL TVGGKVTTEN TVVGGDLTCT DGGLTCQSDL TIQKDINIRP
DE177 LTEDQKKTAF TVGGKVTTEN TVVRGDLTCT DGGLTCQSDL TIQKDINIRP
N16 LTEDQKKTAF TVRGKVTTEN TVVGGDLTCT DGGLTCQSDL TIQKDINIRP
AR39 LTEDQKKTTF TVGGKVTTEN TVVRGDLTCT DGGLTCQSDL TIQKDINIRP
CWL029 LTEDQKKTTF TVGGKVTTEN TVVRGDLTCT DGGLTCQSDL TIQKDINIRP
J138 LTEDQKKTTF TVGGKVTTEN TVVRGDLTCT DGGLTCQSDL TIQKDINIRP
TW183 LTEDQKKTTF TVGGKVTTEN TVVRGDLTCT DGGLTCQSDL TIQKDINIRP
TOR1 LTEDQKKTTF TVGGKVTTEN TVVRGDLTCT DGGLTCQSDL TIQKDINIRP
WA97001 LTEDQKKTTF TVGGKVTTEN TVVRGDLTCT DGGLTCQSDL TIQKDINIRP
1979 LTEDQKKTAF TVGGKVTTEN TVVRGDLTCT DGGLTCQSDL TIQKDINIRP
SH511 LTEDQKKTAF TVGGKVTTEN TVVRGDLTCT DGGLTCQSDL TIQKDINIRP
60 70 80 90 100
Mean Hydrophobicity
Mean PI
Identity
B26 TSTNSMVFDG RLNLSNSPLS YKNSQGQDIT DYEKISSGKP QEYVPFGYYK
B37 TSTNSMVFDG RLNLSNSPLS YKNSQGQDIT DYEKISSGKP QEYVPFGYYK
LPCoLN TSTNSMVFDG RLNLSNSPLS YKNSQGQDIT DYEKISSGKP QEYVPFGYYK
DE177 TSTNSMVFDG RLNLSNSPLS YKNSQGQDIT DYEKMSSGKP QEYVPFGYYK
N16 TSTNDIVFGG RLNLSNSPLS YKNSQGQDIT DYEKMSSGKP QEYVPFGYYK
AR39 TSTNSMVFDG RLNLSNSPLS YKNSQGQDIT DYEKMSSGKP QEYVPFGYYK
CWL029 TSTNSMVFDG RLNLSNSPLS YKNSQGQDIT DYEKMSSGKP QEYVPFGYYK
J138 TSTNSMVFDG RLNLSNSPLS YKNSQGQDIT DYEKMSSGKP QEYVPFGYYK
TW183 TSTNSMVFDG RLNLSNSPLS YKNSQGQDIT DYEKMSSGKP QEYVPFGYYK
TOR1 TSTNSMVFDG RLNLSNSPLS YKNSQGQDIT DYEKMSSGKP QEYVPFGYYK
WA97001 TSTNSMVFDG RLNLSNSPLS YKNSQGQDIT DYEKMSSGKP QEYVPFGYYK
1979 TSTNSMVFDG RLNLSNSPLS YKNSQGQDIT DYEKMSSGKP QEYVPFGYYK
SH511 TSTNSMVFDG RLNLSNSPLS YKNSQGQDIT DYEKMSSGKP QEYVPFGYYK
110 120 130 140 150
Mean Hydrophobicity
Mean PI
Identity
B26 RTQIMMAQRG ATSSGHVGSG SVPSGSYVPW NKFDQTSVQK TSGTEIYIDP
B37 RTQIMMAQRG ATSSGHVGSG SVPSGSYVPW NKFDQTSVQK TSGTEIYIDP
LPCoLN RTQIMMAQRG ATSSGHVGSG SVPSGSYVPW NKFDQTSVQK TSGTEIYIDP
DE177 RTQIMMAQRA AHSSGYVGGG SVPSGSYVPW NKFDQTSTQK TSGTEIYIDP
N16 RTRIMMAQRG AISGGHVGNG SVESGDYVPW NKFNETSGQK TSGTEIYVDP
AR39 RTQIMMAQRA AHSSGYVGGG SVPSGSYVPW NKFDQTSTQK TSGTEIYIDP
CWL029 RTQIMMAQRA AHSSGYVGGG SVPSGSYVPW NKFDQTSTQK TSGTEIYIDP
J138 RTQIMMAQRA AHSSGYVGGG SVPSGSYVPW NKFDQTSTQK TSGTEIYIDP
TW183 RTQIMMAQRA AHSSGYVGGG SVPSGSYVPW NKFDQTSTQK TSGTEIYIDP
TOR1 RTQIMMAQRA AHSSGYVGGG SVPSGSYVPW NKFDQTSTQK TSGTEIYIDP
WA97001 RTQIMMAQRA AHSSGYVGGG SVPSGSYVPW NKFDQTSTQK TSGTEIYIDP
1979 RTQIMMAQRA AHSSGYVGGG SVPSGSYVPW NKFDQTSTQK TSGTEIYIDP
SH511 RTQIMMAQRA AHSSGYVGGG SVPSGSYVPW NKFDQTSTQK TSGTEIYIDP

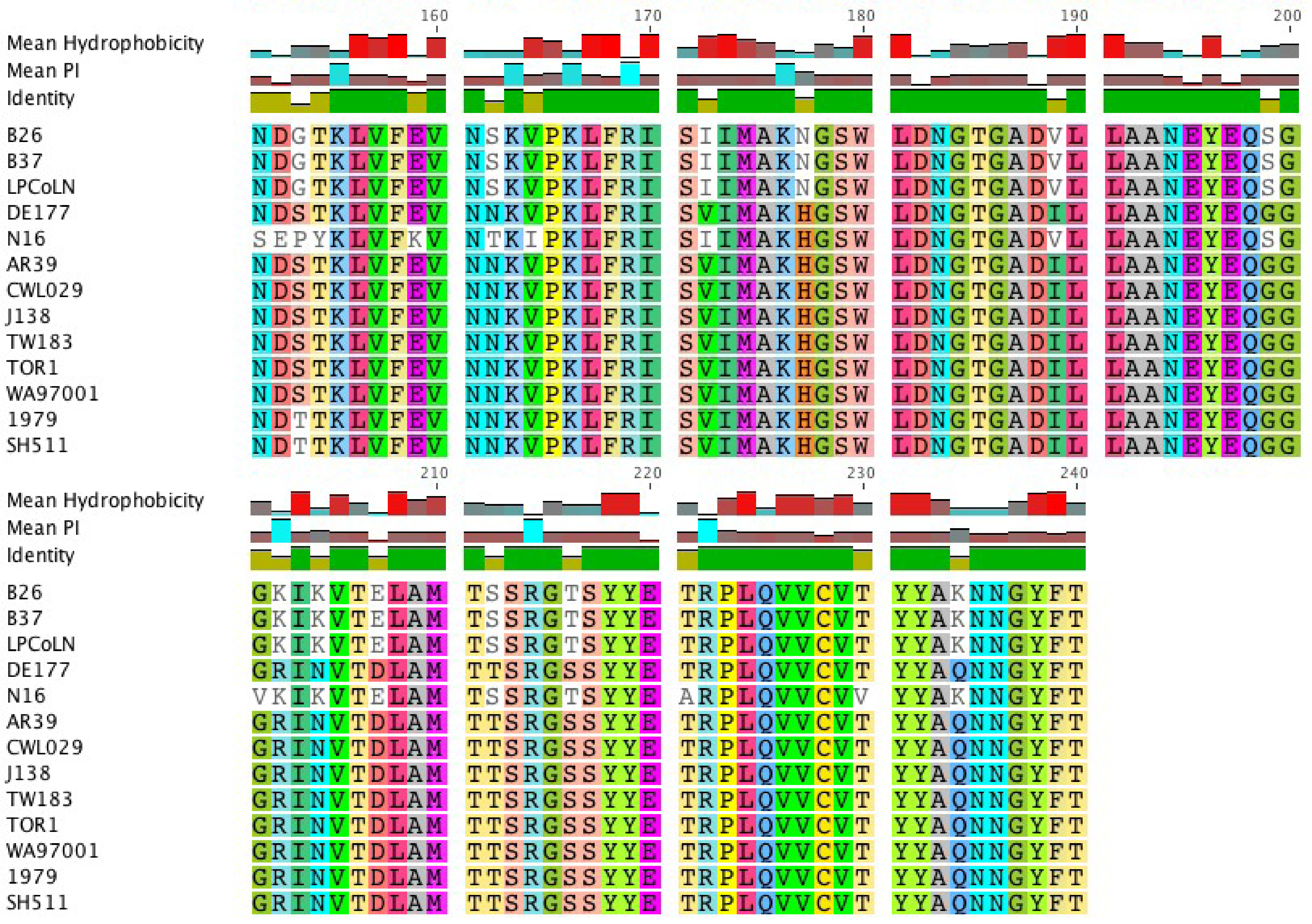
160
170
180
190
200
Mean Hydrophobicity
Mean PI
Identity
B26 NDGTKLVFEV NSKVPKLFRI SIIMAKNGSW LDNGTGADVL LAANEYEQSG
B37 NDGTKLVFEV NSKVPKLFRI SIIMAKNGSW LDNGTGADVL LAANEYEQSG
LPCoLN NDGTKLVFEV NSKVPKLFRI SIIMAKNGSW LDNGTGADVL LAANEYEQSG
DE177 NDSTKLVFEV NNKVPKLFRI SVIMAKHGSW LDNGTGADIL LAANEYEQGG
N16 SEPYKLVFKV NTKIPKLFRI SIIMAKHGSW LDNGTGADVL LAANEYEQSG
AR39 NDSTKLVFEV NNKVPKLFRI SVIMAKHGSW LDNGTGADIL LAANEYEQGG
CWL029 NDSTKLVFEV NNKVPKLFRI SVIMAKHGSW LDNGTGADIL LAANEYEQGG
J138 NDSTKLVFEV NNKVPKLFRI SVIMAKHGSW LDNGTGADIL LAANEYEQGG
TW183 NDSTKLVFEV NNKVPKLFRI SVIMAKHGSW LDNGTGADIL LAANEYEQGG
TOR1 NDSTKLVFEV NNKVPKLFRI SVIMAKHGSW LDNGTGADIL LAANEYEQGG
WA97001 NDSTKLVFEV NNKVPKLFRI SVIMAKHGSW LDNGTGADIL LAANEYEQGG
1979 NDTTKLVFEV NNKVPKLFRI SVIMAKHGSW LDNGTGADIL LAANEYEQGG
SH511 NDTTKLVFEV NNKVPKLFRI SVIMAKHGSW LDNGTGADIL LAANEYEQGG
210
220
230
240
Mean Hydrophobicity
Mean PI
Identity
B26 GKIKVTELAM TSSRGTSYYE TRPLQVVCVT YYAKNNGYFT
B37 GKIKVTELAM TSSRGTSYYE TRPLQVVCVT YYAKNNGYFT
LPCoLN GKIKVTELAM TSSRGTSYYE TRPLQVVCVT YYAKNNGYFT
DE177 GRINVTDLAM TTSRGSSYYE TRPLQVVCVT YYAQNNGYFT
N16 VKIKVTELAM TSSRGTSYYE ARPLQVVCVV YYAKNNGYFT
AR39 GRINVTDLAM TTSRGSSYYE TRPLQVVCVT YYAQNNGYFT
CWL029 GRINVTDLAM TTSRGSSYYE TRPLQVVCVT YYAQNNGYFT
J138 GRINVTDLAM TTSRGSSYYE TRPLQVVCVT YYAQNNGYFT
TW183 GRINVTDLAM TTSRGSSYYE TRPLQVVCVT YYAQNNGYFT
TOR1 GRINVTDLAM TTSRGSSYYE TRPLQVVCVT YYAQNNGYFT
WA97001 GRINVTDLAM TTSRGSSYYE TRPLQVVCVT YYAQNNGYFT
1979 GRINVTDLAM TTSRGSSYYE TRPLQVVCVT YYAQNNGYFT
SH511 GRINVTDLAM TTSRGSSYYE TRPLQVVCVT YYAQNNGYFT
