# Supplementary material for: Chlamydia pneumoniae Is Genetically Diverse in Animals and Appears to Have Crossed the Host Barrier to Humans on (At Least) Two Occasions
Source: PLoS Pathog. 2010 May 20;6(5):e1000903. doi: 10.1371/journal.ppat.1000903 (PMC2873915; doi:10.1371/journal.ppat.1000903)
Supplement: Figure S15 — Multiple sequence alignment of SctC. This gene segment can differentiate animal isolates from Indigenous and non-Indigenous human isolates, on the basis of five key SNPs: Two SNPs (position 232; A/C and 273; A/C) distinguish bandicoot B26, koala LPCoLN, frog DE177 and Indigenous human isolates SH511 and 1979 from the non-Indigenous human isolates AR39, CWL029, J138, TW183, TOR1 and WA97001 (identical sequences); frog DE177 has one unique SNP (position 342; T); koala LPCoLN and bandicoot B26 have one shared SNP (position 429; G); Australian Indigenous human isolates SH511 1979 have one shared SNP (position 469; A). (1.17 MB PDF) [file ppat.1000903.s015.pdf]

	1 … 10	20	30	40	50
Identity					
B26	CAGCCTCACT	TTCAGCAAAG	AAAAGTCATA	CCAAGAAAAC	CACTCCAGGT
LPCoLN	CAGCCTCACT	TTCAGCAAAG	AAAAGTCATA	CCAAGAAAAC	CACTCCAGGT
DE177	CAGCCTCACT	TTCAGCAAAG	AAAAGTCATA	CCAAGAAAAC	CACTCCAGGT
AR39	CAGCCTCACT	TTCAGCAAAG	AAAAGTCATA	CCAAGAAAAC	CACTCCAGGT
CWL029	CAGCCTCACT	TTCAGCAAAG	AAAAGTCATA	CCAAGAAAAC	CACTCCAGGT
J138	CAGCCTCACT	TTCAGCAAAG	AAAAGTCATA	CCAAGAAAAC	CACTCCAGGT
TW183	CAGCCTCACT	TTCAGCAAAG	AAAAGTCATA	CCAAGAAAAC	CACTCCAGGT
TOR1	CAGCCTCACT	TTCAGCAAAG	AAAAGTCATA	CCAAGAAAAC	CACTCCAGGT
WA97001	CAGCCTCACT	TTCAGCAAAG	AAAAGTCATA	CCAAGAAAAC	CACTCCAGGT
1979	CAGCCTCACT	TTCAGCAAAG	AAAAGTCATA	CCAAGAAAAC	CACTCCAGGT
SH511	CAGCCTCACT	TTCAGCAAAG	AAAAGTCATA	CCAAGAAAAC	CACTCCAGGT

	60	70	80	90	100
Identity					
B26	TCTATCCCCA	GTAAAGTCTT	CTCTAAATTC	GACGCAACCC	AAGATAAAAC
LPCoLN	TCTATCCCCA	GTAAAGTCTT	CTCTAAATTC	GACGCAACCC	AAGATAAAAC
DE177	TCTATCCCCA	GTAAAGTCTT	CTCTAAATTC	GACGCAACCC	AAGATAAAAC
AR39	TCTATCCCCA	GTAAAGTCTT	CTCTAAATTC	GACGCAACCC	AAGATAAAAC
CWL029	TCTATCCCCA	GTAAAGTCTT	CTCTAAATTC	GACGCAACCC	AAGATAAAAC
J138	TCTATCCCCA	GTAAAGTCTT	CTCTAAATTC	GACGCAACCC	AAGATAAAAC
TW183	TCTATCCCCA	GTAAAGTCTT	CTCTAAATTC	GACGCAACCC	AAGATAAAAC
TOR1	TCTATCCCCA	GTAAAGTCTT	CTCTAAATTC	GACGCAACCC	AAGATAAAAC
WA97001	TCTATCCCCA	GTAAAGTCTT	CTCTAAATTC	GACGCAACCC	AAGATAAAAC
1979	TCTATCCCCA	GTAAAGTCTT	CTCTAAATTC	GACGCAACCC	AAGATAAAAC
SH511	TCTATCCCCA	GTAAAGTCTT	CTCTAAATTC	GACGCAACCC	AAGATAAAAC

	110	120	130	140	150
Identity					
B26	TTTCCAAAAG	ACTTCAGGAT	CCGCATTCCC	AGCAAAACCT	ACCACCCTGA
LPCoLN	TTTCCAAAAG	ACTTCAGGAT	CCGCATTCCC	AGCAAAACCT	ACCACCCTGA
DE177	TTTCCAAAAG	ACTTCAGGAT	CCGCATTCCC	AGCAAAACCT	ACCACCCTGA
AR39	TTTCCAAAAG	ACTTCAGGAT	CCGCATTCCC	AGCAAAACCT	ACCACCCTGA
CWL029	TTTCCAAAAG	ACTTCAGGAT	CCGCATTCCC	AGCAAAACCT	ACCACCCTGA
J138	TTTCCAAAAG	ACTTCAGGAT	CCGCATTCCC	AGCAAAACCT	ACCACCCTGA
TW183	TTTCCAAAAG	ACTTCAGGAT	CCGCATTCCC	AGCAAAACCT	ACCACCCTGA
TOR1	TTTCCAAAAG	ACTTCAGGAT	CCGCATTCCC	AGCAAAACCT	ACCACCCTGA
WA97001	TTTCCAAAAG	ACTTCAGGAT	CCGCATTCCC	AGCAAAACCT	ACCACCCTGA
1979	TTTCCAAAAG	ACTTCAGGAT	CCGCATTCCC	AGCAAAACCT	ACCACCCTGA
SH511	TTTCCAAAAG	ACTTCAGGAT	CCGCATTCCC	AGCAAAACCT	ACCACCCTGA

	160	170	180	190	200
Identity					
B26	AAGAACTGGA	AGAGAGAAAA	AAACCTCGAC	CAGAGCGTAG	AACTACTGCC
LPCoLN	AAGAACTGGA	AGAGAGAAAA	AAACCTCGAC	CAGAGCGTAG	AACTACTGCC
DE177	AAGAACTGGA	AGAGAGAAAA	AAACCTCGAC	CAGAGCGTAG	AACTACTGCC
AR39	AAGAACTGGA	AGAGAGAAAA	AAACCTCGAC	CAGAGCGTAG	AACTACTGCC
CWL029	AAGAACTGGA	AGAGAGAAAA	AAACCTCGAC	CAGAGCGTAG	AACTACTGCC
J138	AAGAACTGGA	AGAGAGAAAA	AAACCTCGAC	CAGAGCGTAG	AACTACTGCC
TW183	AAGAACTGGA	AGAGAGAAAA	AAACCTCGAC	CAGAGCGTAG	AACTACTGCC
TOR1	AAGAACTGGA	AGAGAGAAAA	AAACCTCGAC	CAGAGCGTAG	AACTACTGCC
WA97001	AAGAACTGGA	AGAGAGAAAA	AAACCTCGAC	CAGAGCGTAG	AACTACTGCC
1979	AAGAACTGGA	AGAGAGAAAA	AAACCTCGAC	CAGAGCGTAG	AACTACTGCC
SH511	AAGAACTGGA	AGAGAGAAAA	AAACCTCGAC	CAGAGCGTAG	AACTACTGCC

	210	220	230	240	250
Identity					
B26	GATGTAAAAA	GATCCCCACG	CTTCTTACCA	AAACAAGAAG	TCGAAGAGCC
LPCoLN	GATGTAAAAA	GATCCCCACG	CTTCTTACCA	AAACAAGAAG	TCGAAGAGCC
DE177	GATGTAAAAA	GATCCCCACG	CTTCTTACCA	AAACAAGAAG	TCGAAGAGCC
AR39	GATGTAAAAA	GATCCCCACG	CTTCTTACCA	ACACAAGAAG	TCGAAGAGCC
CWL029	GATGTAAAAA	GATCCCCACG	CTTCTTACCA	ACACAAGAAG	TCGAAGAGCC
J138	GATGTAAAAA	GATCCCCACG	CTTCTTACCA	ACACAAGAAG	TCGAAGAGCC
TW183	GATGTAAAAA	GATCCCCACG	CTTCTTACCA	ACACAAGAAG	TCGAAGAGCC
TOR1	GATGTAAAAA	GATCCCCACG	CTTCTTACCA	ACACAAGAAG	TCGAAGAGCC
WA97001	GATGTAAAAA	GATCCCCACG	CTTCTTACCA	ACACAAGAAG	TCGAAGAGCC
1979	GATGTAAAAA	GATCCCCACG	CTTCTTACCA	AAACAAGAAG	TCGAAGAGCC
SH511	GATGTAAAAA	GATCCCCACG	CTTCTTACCA	AAACAAGAAG	TCGAAGAGCC

	260	270	280	290	300
Identity					
B26	TGTACCTGCC	GCCTCTAAAG	AAAAATTAGA	TAGTATACAG	GTTTGGGAAG
LPCoLN	TGTACCTGCC	GCCTCTAAAG	AAAAATTAGA	TAGTATACAG	GTTTGGGAAG
DE177	TGTACCTGCC	GCCTCTAAAG	AAAAATTAGA	TAGTATACAG	GTTTGGGAAG
AR39	TGTACCTGCC	GCCTCTAAAG	AACAATTAGA	TAGTATACAG	GTTTGGGAAG
CWL029	TGTACCTGCC	GCCTCTAAAG	AACAATTAGA	TAGTATACAG	GTTTGGGAAG
J138	TGTACCTGCC	GCCTCTAAAG	AACAATTAGA	TAGTATACAG	GTTTGGGAAG
TW183	TGTACCTGCC	GCCTCTAAAG	AACAATTAGA	TAGTATACAG	GTTTGGGAAG
TOR1	TGTACCTGCC	GCCTCTAAAG	AACAATTAGA	TAGTATACAG	GTTTGGGAAG
WA97001	TGTACCTGCC	GCCTCTAAAG	AACAATTAGA	TAGTATACAG	GTTTGGGAAG
1979	TGTACCTGCC	GCCTCTAAAG	AAAAATTAGA	TAGTATACAG	GTTTGGGAAG
SH511	TGTACCTGCC	GCCTCTAAAG	AAAAATTAGA	TAGTATACAG	GTTTGGGAAG

	310	320	330	340	350
Identity					
B26	AAAAACAAAA	TTATGCTCGC	AGAGCCGTAA	ATGCTATCAA	TCTGAGTATA
LPCoLN	AAAAACAAAA	TTATGCTCGC	AGAGCCGTAA	ATGCTATCAA	TCTGAGTATA
DE177	AAAAACAAAA	TTATGCTCGC	AGAGCCGTAA	ATGCTATCAA	TTTGAGTATA
AR39	AAAAACAAAA	TTATGCTCGC	AGAGCCGTAA	ATGCTATCAA	TCTGAGTATA
CWL029	AAAAACAAAA	TTATGCTCGC	AGAGCCGTAA	ATGCTATCAA	TCTGAGTATA
J138	AAAAACAAAA	TTATGCTCGC	AGAGCCGTAA	ATGCTATCAA	TCTGAGTATA
TW183	AAAAACAAAA	TTATGCTCGC	AGAGCCGTAA	ATGCTATCAA	TCTGAGTATA
TOR1	AAAAACAAAA	TTATGCTCGC	AGAGCCGTAA	ATGCTATCAA	TCTGAGTATA
WA97001	AAAAACAAAA	TTATGCTCGC	AGAGCCGTAA	ATGCTATCAA	TCTGAGTATA
1979	AAAAACAAAA	TTATGCTCGC	AGAGCCGTAA	ATGCTATCAA	TCTGAGTATA
SH511	AAAAACAAAA	TTATGCTCGC	AGAGCCGTAA	ATGCTATCAA	TCTGAGTATA

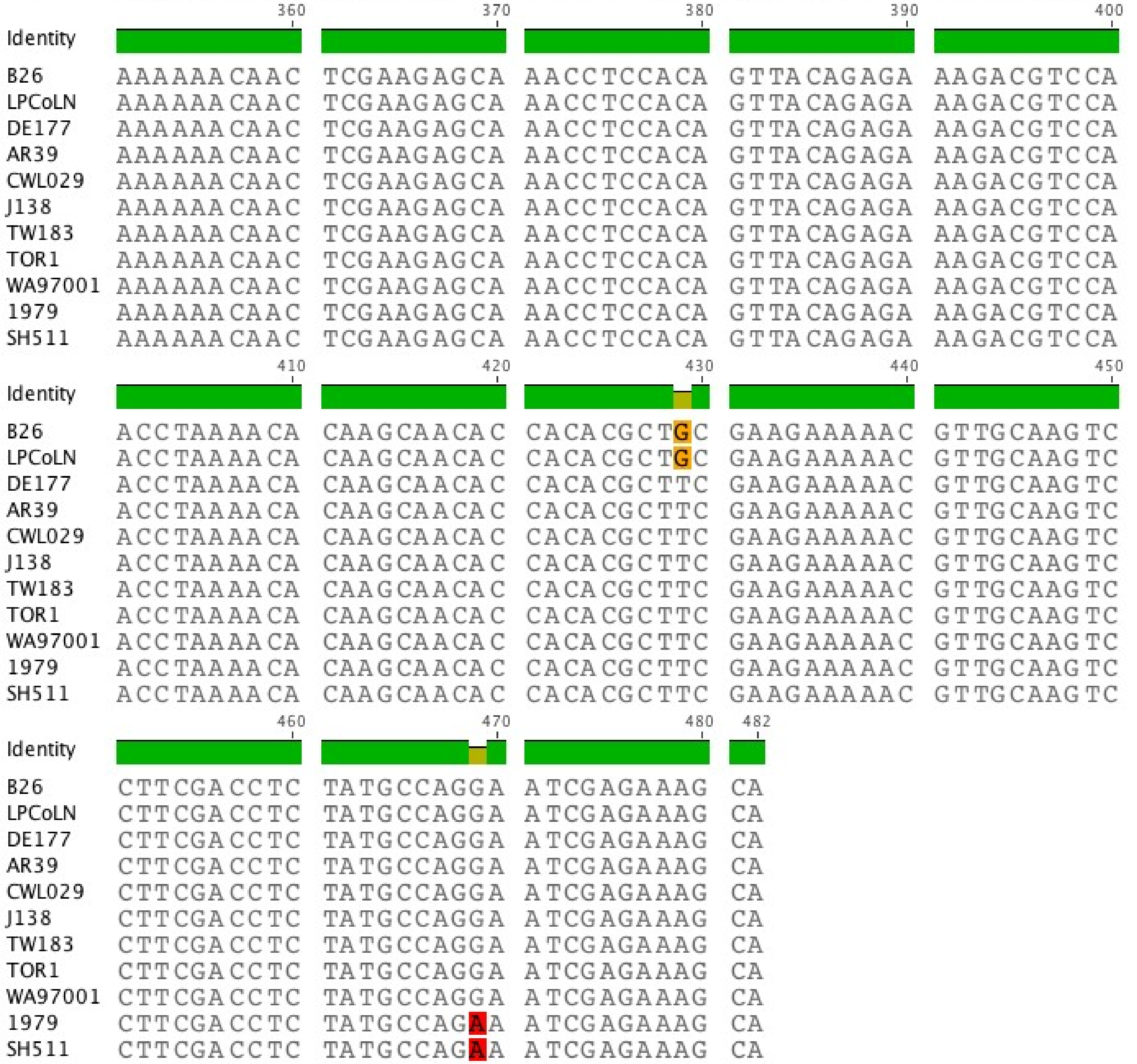

360 370 380 390 400
Identity
B26 AAAAAACAAC TCGAAGAGCA AACCTCCACA GTTACAGAGA AAGACGTCCA
LPCoLN AAAAAACAAC TCGAAGAGCA AACCTCCACA GTTACAGAGA AAGACGTCCA
DE177 AAAAAACAAC TCGAAGAGCA AACCTCCACA GTTACAGAGA AAGACGTCCA
AR39 AAAAAACAAC TCGAAGAGCA AACCTCCACA GTTACAGAGA AAGACGTCCA
CWL029 AAAAAACAAC TCGAAGAGCA AACCTCCACA GTTACAGAGA AAGACGTCCA
J138 AAAAAACAAC TCGAAGAGCA AACCTCCACA GTTACAGAGA AAGACGTCCA
TW183 AAAAAACAAC TCGAAGAGCA AACCTCCACA GTTACAGAGA AAGACGTCCA
TOR1 AAAAAACAAC TCGAAGAGCA AACCTCCACA GTTACAGAGA AAGACGTCCA
WA97001 AAAAAACAAC TCGAAGAGCA AACCTCCACA GTTACAGAGA AAGACGTCCA
1979 AAAAAACAAC TCGAAGAGCA AACCTCCACA GTTACAGAGA AAGACGTCCA
SH511 AAAAAACAAC TCGAAGAGCA AACCTCCACA GTTACAGAGA AAGACGTCCA
410 420 430 440 450
Identity
B26 ACCTAAAACA CAAGCAACAC CACACGCTGC GAAGAAAAAC GTTGCAAGTC
LPCoLN ACCTAAAACA CAAGCAACAC CACACGCTGC GAAGAAAAAC GTTGCAAGTC
DE177 ACCTAAAACA CAAGCAACAC CACACGCTTC GAAGAAAAAC GTTGCAAGTC
AR39 ACCTAAAACA CAAGCAACAC CACACGCTTC GAAGAAAAAC GTTGCAAGTC
CWL029 ACCTAAAACA CAAGCAACAC CACACGCTTC GAAGAAAAAC GTTGCAAGTC
J138 ACCTAAAACA CAAGCAACAC CACACGCTTC GAAGAAAAAC GTTGCAAGTC
TW183 ACCTAAAACA CAAGCAACAC CACACGCTTC GAAGAAAAAC GTTGCAAGTC
TOR1 ACCTAAAACA CAAGCAACAC CACACGCTTC GAAGAAAAAC GTTGCAAGTC
WA97001 ACCTAAAACA CAAGCAACAC CACACGCTTC GAAGAAAAAC GTTGCAAGTC
1979 ACCTAAAACA CAAGCAACAC CACACGCTTC GAAGAAAAAC GTTGCAAGTC
SH511 ACCTAAAACA CAAGCAACAC CACACGCTTC GAAGAAAAAC GTTGCAAGTC
460 470 480 482
Identity
B26 CTTCGACCTC TATGCCAGGA ATCGAGAAAG CA
LPCoLN CTTCGACCTC TATGCCAGGA ATCGAGAAAG CA
DE177 CTTCGACCTC TATGCCAGGA ATCGAGAAAG CA
AR39 CTTCGACCTC TATGCCAGGA ATCGAGAAAG CA
CWL029 CTTCGACCTC TATGCCAGGA ATCGAGAAAG CA
J138 CTTCGACCTC TATGCCAGGA ATCGAGAAAG CA
TW183 CTTCGACCTC TATGCCAGGA ATCGAGAAAG CA
TOR1 CTTCGACCTC TATGCCAGGA ATCGAGAAAG CA
WA97001 CTTCGACCTC TATGCCAGGA ATCGAGAAAG CA
1979 CTTCGACCTC TATGCCAGAA ATCGAGAAAG CA
SH511 CTTCGACCTC TATGCCAGAA ATCGAGAAAG CA

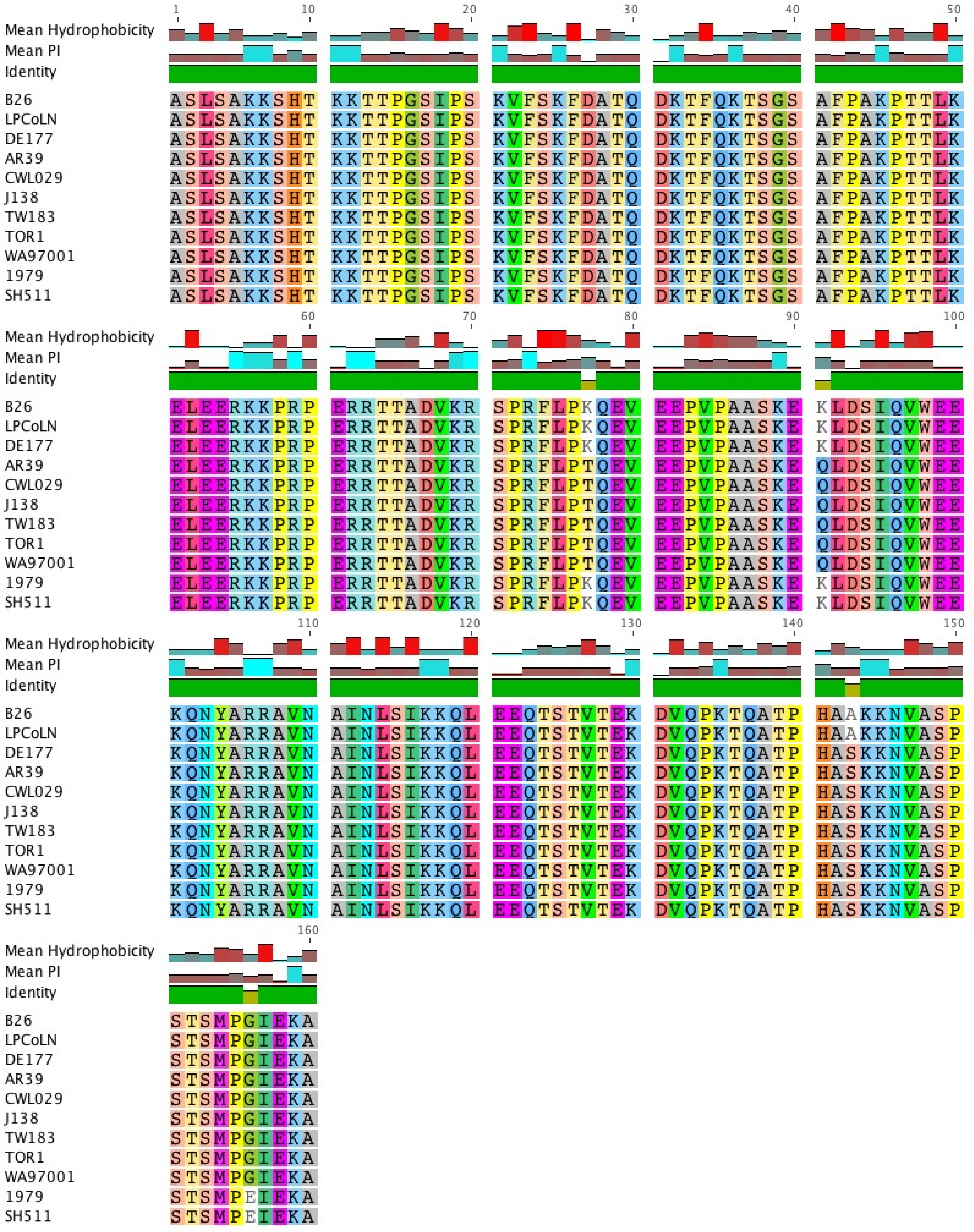

1 10 20 30 40 50
Mean Hydrophobicity
Mean PI
Identity
B26 ASLSAKKSHT KKTTPGSIPS KVFSKFDATQ DKTFQKTSGS AFPAKPTTLK
LPCoLN ASLSAKKSHT KKTTPGSIPS KVFSKFDATQ DKTFQKTSGS AFPAKPTTLK
DE177 ASLSAKKSHT KKTTPGSIPS KVFSKFDATQ DKTFQKTSGS AFPAKPTTLK
AR39 ASLSAKKSHT KKTTPGSIPS KVFSKFDATQ DKTFQKTSGS AFPAKPTTLK
CWL029 ASLSAKKSHT KKTTPGSIPS KVFSKFDATQ DKTFQKTSGS AFPAKPTTLK
J138 ASLSAKKSHT KKTTPGSIPS KVFSKFDATQ DKTFQKTSGS AFPAKPTTLK
TW183 ASLSAKKSHT KKTTPGSIPS KVFSKFDATQ DKTFQKTSGS AFPAKPTTLK
TOR1 ASLSAKKSHT KKTTPGSIPS KVFSKFDATQ DKTFQKTSGS AFPAKPTTLK
WA97001 ASLSAKKSHT KKTTPGSIPS KVFSKFDATQ DKTFQKTSGS AFPAKPTTLK
1979 ASLSAKKSHT KKTTPGSIPS KVFSKFDATQ DKTFQKTSGS AFPAKPTTLK
SH511 ASLSAKKSHT KKTTPGSIPS KVFSKFDATQ DKTFQKTSGS AFPAKPTTLK
60 70 80 90 100
Mean Hydrophobicity
Mean PI
Identity
B26 ELEERKKPRP ERRTTADVKR SPRFLPKQEV EEPVPAASKE KLDSIQVWEE
LPCoLN ELEERKKPRP ERRTTADVKR SPRFLPKQEV EEPVPAASKE KLDSIQVWEE
DE177 ELEERKKPRP ERRTTADVKR SPRFLPKQEV EEPVPAASKE KLDSIQVWEE
AR39 ELEERKKPRP ERRTTADVKR SPRFLPTQEV EEPVPAASKE QLDSIQVWEE
CWL029 ELEERKKPRP ERRTTADVKR SPRFLPTQEV EEPVPAASKE QLDSIQVWEE
J138 ELEERKKPRP ERRTTADVKR SPRFLPTQEV EEPVPAASKE QLDSIQVWEE
TW183 ELEERKKPRP ERRTTADVKR SPRFLPTQEV EEPVPAASKE QLDSIQVWEE
TOR1 ELEERKKPRP ERRTTADVKR SPRFLPTQEV EEPVPAASKE QLDSIQVWEE
WA97001 ELEERKKPRP ERRTTADVKR SPRFLPTQEV EEPVPAASKE QLDSIQVWEE
1979 ELEERKKPRP ERRTTADVKR SPRFLPKQEV EEPVPAASKE KLDSIQVWEE
SH511 ELEERKKPRP ERRTTADVKR SPRFLPKQEV EEPVPAASKE KLDSIQVWEE
110 120 130 140 150
Mean Hydrophobicity
Mean PI
Identity
B26 KQNYARRAVN AINLSIKKQL EEQTSTVTEK DVQPKTQATP HAAKKNVASP
LPCoLN KQNYARRAVN AINLSIKKQL EEQTSTVTEK DVQPKTQATP HAAKKNVASP
DE177 KQNYARRAVN AINLSIKKQL EEQTSTVTEK DVQPKTQATP HASKKNVASP
AR39 KQNYARRAVN AINLSIKKQL EEQTSTVTEK DVQPKTQATP HASKKNVASP
CWL029 KQNYARRAVN AINLSIKKQL EEQTSTVTEK DVQPKTQATP HASKKNVASP
J138 KQNYARRAVN AINLSIKKQL EEQTSTVTEK DVQPKTQATP HASKKNVASP
TW183 KQNYARRAVN AINLSIKKQL EEQTSTVTEK DVQPKTQATP HASKKNVASP
TOR1 KQNYARRAVN AINLSIKKQL EEQTSTVTEK DVQPKTQATP HASKKNVASP
WA97001 KQNYARRAVN AINLSIKKQL EEQTSTVTEK DVQPKTQATP HASKKNVASP
1979 KQNYARRAVN AINLSIKKQL EEQTSTVTEK DVQPKTQATP HASKKNVASP
SH511 KQNYARRAVN AINLSIKKQL EEQTSTVTEK DVQPKTQATP HASKKNVASP
160
Mean Hydrophobicity
Mean PI
Identity
B26 STSMPGIEKA
LPCoLN STSMPGIEKA
DE177 STSMPGIEKA
AR39 STSMPGIEKA
CWL029 STSMPGIEKA
J138 STSMPGIEKA
TW183 STSMPGIEKA
TOR1 STSMPGIEKA
WA97001 STSMPGIEKA
1979 STSMPEIEKA
SH511 STSMPEIEKA
