# Supplementary material for: Chlamydia pneumoniae Is Genetically Diverse in Animals and Appears to Have Crossed the Host Barrier to Humans on (At Least) Two Occasions
Source: PLoS Pathog. 2010 May 20;6(5):e1000903. doi: 10.1371/journal.ppat.1000903 (PMC2873915; doi:10.1371/journal.ppat.1000903)
Supplement: Figure S16 — Multiple sequence alignment of HAF. The sequenced human C. pneumoniae genomes are truncated (120 bp deletion), whereas additional human isolates (TOR1, WA97001, SH511, and 1979) and animal isolates (B26, DE177 and N16) are not truncated. (2.31 MB PDF) [file ppat.1000903.s016.pdf]

	1 10	20	30	40	50
Identity					
B26	TTGGGTCTAT	GGAGAGCACG	ACAACAAGGA	AAACTACAGC	TGTAAAATGG
LPCoLN	TTGGGTCTAT	GGAGAGCACG	ACAACAAGGA	AAACTACAGC	TGTAAAATGG
DE177	TTGGGTCTAT	GGAGAGCACG	ATAACAAGGA	AAACTACAGC	TGTAAAATGG
N16	TTGGGTCTAT	GGAGAGCACA	ATAACAAGGA	AAACTACAGC	TGTAAAATGG
AR39	TTGGGTCTAT	GGAGAGCACG	ATAACAAGGA	AAACTACAGC	TGTAAAATGG
CWL029	TTGGGTCTAT	GGAGAGCACG	ATAACAAGGA	AAACTACAGC	TGTAAAATGG
J138	TTGGGTCTAT	GGAGAGCACG	ATAACAAGGA	AAACTACAGC	TGTAAAATGG
TW183	TTGGGTCTAT	GGAGAGCACG	ATAACAAGGA	AAACTACAGC	TGTAAAATGG
TOR1	TTGGGTCTAT	GGAGAGCACG	ATAACAAGGA	AAACTACAGC	TGTAAAATGG
WA97001	TTGGGTCTAT	GGAGAGCACG	ATAACAAGGA	AAACTACAGC	TGTAAAATGG
1979	TTGGGTCTAT	GGAGAGCACG	ATAACAAGGA	AAACTACAGC	TGTAAAATGG
SH511	TTGGGTCTAT	GGAGAGCACG	ATAACAAGGA	AAACTACAGC	TGTAAAATGG

	60	70	80	90	100
Identity					
B26	GTAAACAATG	TTCCTACGTA	TCTGGGAACC	TTAGGAGGAG	ATGCTTCTAC
LPCoLN	GTAAACAATG	TTCCTACGTA	TCTGGGAACC	TTAGGAGGAG	ATGCTTCTAC
DE177	GTAAATAATG	TTCCTACGTA	TCTGGGAACC	TTAGGAGGAG	ATGCTTCTAC
N16	GTAAACAATG	TTCCTACGTA	TCTGGGAACC	TTAGGAGGAG	ATGCTTCTAC
AR39	GTAAATAATG	TTCCTACGTA	TCTGGGAACC	TTAGGAGGAG	ATGCTTCTAC
CWL029	GTAAATAATG	TTCCTACGTA	TCTGGGAACC	TTAGGAGGAG	ATGCTTCTAC
J138	GTAAATAATG	TTCCTACGTA	TCTGGGAACC	TTAGGAGGAG	ATGCTTCTAC
TW183	GTAAATAATG	TTCCTACGTA	TCTGGGAACC	TTAGGAGGAG	ATGCTTCTAC
TOR1	GTAAATAATG	TTCCTACGTA	TCTGGGAACC	TTAGGAGGAG	ATGCTTCTAC
WA97001	GTAAATAATG	TTCCTACGTA	TCTGGGAACC	TTAGGAGGAG	ATGCTTCTAC
1979	GTAAATAATG	TTCCTACGTA	TCTGGGAACC	TTAGGAGGAG	ATGCTTCTAC
SH511	GTAAATAATG	TTCCTACGTA	TCTGGGAACC	TTAGGAGGAG	ATGCTTCTAC

	110	120	130	140	150
Identity					
B26	AGGTCTTTAT	ATTTCTGGAG	ACGGCACCGT	GATTGTAGGT	GCGGCAAATA
LPCoLN	AGGTCTTTAT	ATTTCTGGAG	ACGGCACCGT	GATTGTAGGT	GCGGCAAATA
DE177	AGGTCTTTAT	ATTTCTGGAG	ACGGCACCGT	GATTGTAGGT	GCGGCAAATA
N16	AGGTCTCTAT	ATTTCTGGAG	ACGGCACCGT	GATTGTAGGT	GCGGCAAATA
AR39	AGGTCTTTAT	ATTTCTGGAG	ACGGCACCGT	GATTGTAGGT	GCGGCAAATA
CWL029	AGGTCTTTAT	ATTTCTGGAG	ACGGCACCGT	GATTGTAGGT	GCGGCAAATA
J138	AGGTCTTTAT	ATTTCTGGAG	ACGGCACCGT	GATTGTAGGT	GCGGCAAATA
TW183	AGGTCTTTAT	ATTTCTGGAG	ACGGCACCGT	GATTGTAGGT	GCGGCAAATA
TOR1	AGGTCTTTAT	ATTTCTGGAG	ACGGCACCGT	GATTGTAGGT	GCGGCAAATA
WA97001	AGGTCTTTAT	ATTTCTGGAG	ACGGCACCGT	GATTGTAGGT	GCGGCAAATA
1979	AGGTCTTTAT	ATTTCTGGAG	ACGGCACCGT	GATTGTAGGT	GCGGCAAATA
SH511	AGGTCTTTAT	ATTTCTGGAG	ACGGCACCGT	GATTGTAGGT	GCGGCAAATA

	160	170	180	190	200
Identity					
B26	CAGCAACTGT	AACCAATGGG	AATCAGGAAT	CCCACGCCTA	TATGTATAAA
LPCoLN	CAGCAACTGT	AACCAATGGG	AATCAGGAAT	CCCACGCCTA	TATGTATAAA
DE177	CAGCAACTGT	AACCAATGGG	AATCAGGAAT	CCCACGCCTA	TATGTATAAA
N16	CAGCAACTGT	AACCAATGGG	AATCAGGAAT	CCCACGCCTA	TATGTATAAA
AR39	CAGCAACTGT	AACCAATGGG	AATCAGGAAT	CCCACGCCTA	TATGTATAAA
CWL029	CAGCAACTGT	AACCAATGGG	AATCAGGAAT	CCCACGCCTA	TATGTATAAA
J138	CAGCAACTGT	AACCAATGGG	AATCAGGAAT	CCCACGCCTA	TATGTATAAA
TW183	CAGCAACTGT	AACCAATGGG	AATCAGGAAT	CCCACGCCTA	TATGTATAAA
TOR1	CAGCAACTGT	AACCAATGGG	AATCAGGAAT	CCCACGCCTA	TATGTATAAA
WA97001	CAGCAACTGT	AACCAATGGG	AATCAGGAAT	CCCACGCCTA	TATGTATAAA
1979	CAGCAACTGT	AACCAATGGG	AATCAGGAAT	CCCACGCCTA	TATGTATAAA
SH511	CAGCAACTGT	AACCAATGGG	AATCAGGAAT	CCCACGCCTA	TATGTATAAA

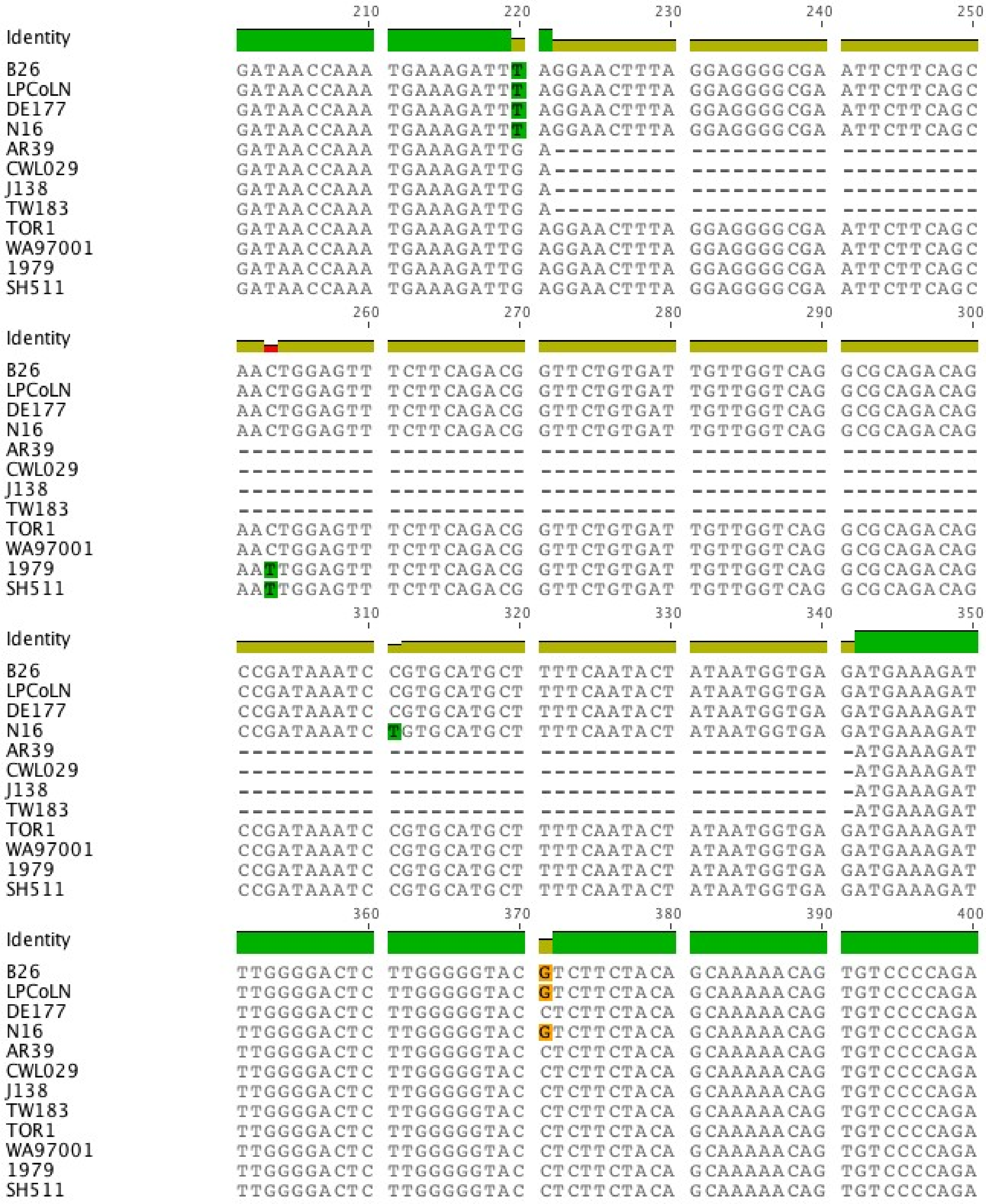
210 220 230 240 250
Identity
B26 GATAACCAAA TGAAAGATTT AGGAACTTTA GGAGGGGCGA ATTCTTCAGC
LPCoLN GATAACCAAA TGAAAGATTT AGGAACTTTA GGAGGGGCGA ATTCTTCAGC
DE177 GATAACCAAA TGAAAGATTT AGGAACTTTA GGAGGGGCGA ATTCTTCAGC
N16 GATAACCAAA TGAAAGATTT AGGAACTTTA GGAGGGGCGA ATTCTTCAGC
AR39 GATAACCAAA TGAAAGATTG A--------- ---------- ----------
CWL029 GATAACCAAA TGAAAGATTG A--------- ---------- ----------
J138 GATAACCAAA TGAAAGATTG A--------- ---------- ----------
TW183 GATAACCAAA TGAAAGATTG A--------- ---------- ----------
TOR1 GATAACCAAA TGAAAGATTG AGGAACTTTA GGAGGGGCGA ATTCTTCAGC
WA97001 GATAACCAAA TGAAAGATTG AGGAACTTTA GGAGGGGCGA ATTCTTCAGC
1979 GATAACCAAA TGAAAGATTG AGGAACTTTA GGAGGGGCGA ATTCTTCAGC
SH511 GATAACCAAA TGAAAGATTG AGGAACTTTA GGAGGGGCGA ATTCTTCAGC
260 270 280 290 300
Identity
B26 AACTGGAGTT TCTTCAGACG GTTCTGTGAT TGTTGGTCAG GCGCAGACAG
LPCoLN AACTGGAGTT TCTTCAGACG GTTCTGTGAT TGTTGGTCAG GCGCAGACAG
DE177 AACTGGAGTT TCTTCAGACG GTTCTGTGAT TGTTGGTCAG GCGCAGACAG
N16 AACTGGAGTT TCTTCAGACG GTTCTGTGAT TGTTGGTCAG GCGCAGACAG
AR39 ---------- ---------- ---------- ---------- ----------
CWL029 ---------- ---------- ---------- ---------- ----------
J138 ---------- ---------- ---------- ---------- ----------
TW183 ---------- ---------- ---------- ---------- ----------
TOR1 AACTGGAGTT TCTTCAGACG GTTCTGTGAT TGTTGGTCAG GCGCAGACAG
WA97001 AACTGGAGTT TCTTCAGACG GTTCTGTGAT TGTTGGTCAG GCGCAGACAG
1979 AATTGGAGTT TCTTCAGACG GTTCTGTGAT TGTTGGTCAG GCGCAGACAG
SH511 AATTGGAGTT TCTTCAGACG GTTCTGTGAT TGTTGGTCAG GCGCAGACAG
310 320 330 340 350
Identity
B26 CCGATAAATC CGTGCATGCT TTTCAATACT ATAATGGTGA GATGAAAGAT
LPCoLN CCGATAAATC CGTGCATGCT TTTCAATACT ATAATGGTGA GATGAAAGAT
DE177 CCGATAAATC CGTGCATGCT TTTCAATACT ATAATGGTGA GATGAAAGAT
N16 CCGATAAATC TGTGCATGCT TTTCAATACT ATAATGGTGA GATGAAAGAT
AR39 ---------- ---------- ---------- ---------- -ATGAAAGAT
CWL029 ---------- ---------- ---------- ---------- -ATGAAAGAT
J138 ---------- ---------- ---------- ---------- -ATGAAAGAT
TW183 ---------- ---------- ---------- ---------- -ATGAAAGAT
TOR1 CCGATAAATC CGTGCATGCT TTTCAATACT ATAATGGTGA GATGAAAGAT
WA97001 CCGATAAATC CGTGCATGCT TTTCAATACT ATAATGGTGA GATGAAAGAT
1979 CCGATAAATC CGTGCATGCT TTTCAATACT ATAATGGTGA GATGAAAGAT
SH511 CCGATAAATC CGTGCATGCT TTTCAATACT ATAATGGTGA GATGAAAGAT
360 370 380 390 400
Identity
B26 TTGGGGACTC TTGGGGGTAC GTCTTCTACA GCAAAAACAG TGTCCCCAGA
LPCoLN TTGGGGACTC TTGGGGGTAC GTCTTCTACA GCAAAAACAG TGTCCCCAGA
DE177 TTGGGGACTC TTGGGGGTAC CTCTTCTACA GCAAAAACAG TGTCCCCAGA
N16 TTGGGGACTC TTGGGGGTAC GTCTTCTACA GCAAAAACAG TGTCCCCAGA
AR39 TTGGGGACTC TTGGGGGTAC CTCTTCTACA GCAAAAACAG TGTCCCCAGA
CWL029 TTGGGGACTC TTGGGGGTAC CTCTTCTACA GCAAAAACAG TGTCCCCAGA
J138 TTGGGGACTC TTGGGGGTAC CTCTTCTACA GCAAAAACAG TGTCCCCAGA
TW183 TTGGGGACTC TTGGGGGTAC CTCTTCTACA GCAAAAACAG TGTCCCCAGA
TOR1 TTGGGGACTC TTGGGGGTAC CTCTTCTACA GCAAAAACAG TGTCCCCAGA
WA97001 TTGGGGACTC TTGGGGGTAC CTCTTCTACA GCAAAAACAG TGTCCCCAGA
1979 TTGGGGACTC TTGGGGGTAC CTCTTCTACA GCAAAAACAG TGTCCCCAGA
SH511 TTGGGGACTC TTGGGGGTAC CTCTTCTACA GCAAAAACAG TGTCCCCAGA

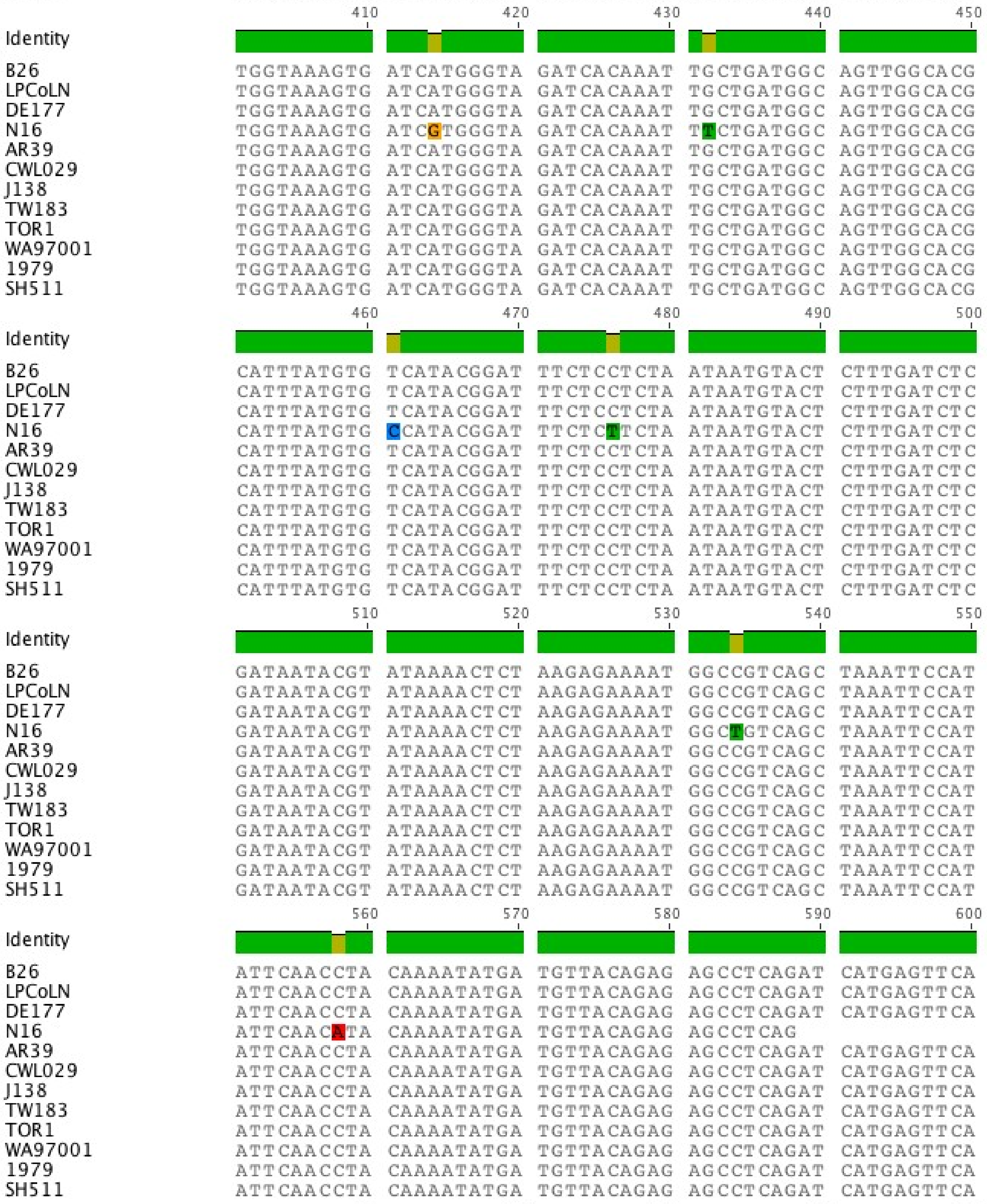

410 420 430 440 450
Identity
B26 TGGTAAAGTG ATCATGGGTA GATCACAAAT TGCTGATGGC AGTTGGCACG
LPCoLN TGGTAAAGTG ATCATGGGTA GATCACAAAT TGCTGATGGC AGTTGGCACG
DE177 TGGTAAAGTG ATCATGGGTA GATCACAAAT TGCTGATGGC AGTTGGCACG
N16 TGGTAAAGTG ATCGTGGGTA GATCACAAAT TTCTGATGGC AGTTGGCACG
AR39 TGGTAAAGTG ATCATGGGTA GATCACAAAT TGCTGATGGC AGTTGGCACG
CWL029 TGGTAAAGTG ATCATGGGTA GATCACAAAT TGCTGATGGC AGTTGGCACG
J138 TGGTAAAGTG ATCATGGGTA GATCACAAAT TGCTGATGGC AGTTGGCACG
TW183 TGGTAAAGTG ATCATGGGTA GATCACAAAT TGCTGATGGC AGTTGGCACG
TOR1 TGGTAAAGTG ATCATGGGTA GATCACAAAT TGCTGATGGC AGTTGGCACG
WA97001 TGGTAAAGTG ATCATGGGTA GATCACAAAT TGCTGATGGC AGTTGGCACG
1979 TGGTAAAGTG ATCATGGGTA GATCACAAAT TGCTGATGGC AGTTGGCACG
SH511 TGGTAAAGTG ATCATGGGTA GATCACAAAT TGCTGATGGC AGTTGGCACG
460 470 480 490 500
Identity
B26 CATTTATGTG TCATACGGAT TTCTCCTCTA ATAATGTACT CTTTGATCTC
LPCoLN CATTTATGTG TCATACGGAT TTCTCCTCTA ATAATGTACT CTTTGATCTC
DE177 CATTTATGTG TCATACGGAT TTCTCCTCTA ATAATGTACT CTTTGATCTC
N16 CATTTATGTG CCATACGGAT TTCTCTTCTA ATAATGTACT CTTTGATCTC
AR39 CATTTATGTG TCATACGGAT TTCTCCTCTA ATAATGTACT CTTTGATCTC
CWL029 CATTTATGTG TCATACGGAT TTCTCCTCTA ATAATGTACT CTTTGATCTC
J138 CATTTATGTG TCATACGGAT TTCTCCTCTA ATAATGTACT CTTTGATCTC
TW183 CATTTATGTG TCATACGGAT TTCTCCTCTA ATAATGTACT CTTTGATCTC
TOR1 CATTTATGTG TCATACGGAT TTCTCCTCTA ATAATGTACT CTTTGATCTC
WA97001 CATTTATGTG TCATACGGAT TTCTCCTCTA ATAATGTACT CTTTGATCTC
1979 CATTTATGTG TCATACGGAT TTCTCCTCTA ATAATGTACT CTTTGATCTC
SH511 CATTTATGTG TCATACGGAT TTCTCCTCTA ATAATGTACT CTTTGATCTC
510 520 530 540 550
Identity
B26 GATAATACGT ATAAAACTCT AAGAGAAAAT GGCCGTCAGC TAAATTCCAT
LPCoLN GATAATACGT ATAAAACTCT AAGAGAAAAT GGCCGTCAGC TAAATTCCAT
DE177 GATAATACGT ATAAAACTCT AAGAGAAAAT GGCCGTCAGC TAAATTCCAT
N16 GATAATACGT ATAAAACTCT AAGAGAAAAT GGCTGTCAGC TAAATTCCAT
AR39 GATAATACGT ATAAAACTCT AAGAGAAAAT GGCCGTCAGC TAAATTCCAT
CWL029 GATAATACGT ATAAAACTCT AAGAGAAAAT GGCCGTCAGC TAAATTCCAT
J138 GATAATACGT ATAAAACTCT AAGAGAAAAT GGCCGTCAGC TAAATTCCAT
TW183 GATAATACGT ATAAAACTCT AAGAGAAAAT GGCCGTCAGC TAAATTCCAT
TOR1 GATAATACGT ATAAAACTCT AAGAGAAAAT GGCCGTCAGC TAAATTCCAT
WA97001 GATAATACGT ATAAAACTCT AAGAGAAAAT GGCCGTCAGC TAAATTCCAT
1979 GATAATACGT ATAAAACTCT AAGAGAAAAT GGCCGTCAGC TAAATTCCAT
SH511 GATAATACGT ATAAAACTCT AAGAGAAAAT GGCCGTCAGC TAAATTCCAT
560 570 580 590 600
Identity
B26 ATTCAACCTA CAAAATATGA TGTTACAGAG AGCCTCAGAT CATGAGTTCA
LPCoLN ATTCAACCTA CAAAATATGA TGTTACAGAG AGCCTCAGAT CATGAGTTCA
DE177 ATTCAACCTA CAAAATATGA TGTTACAGAG AGCCTCAGAT CATGAGTTCA
N16 ATTCAACATA CAAAATATGA TGTTACAGAG AGCCTCAG
AR39 ATTCAACCTA CAAAATATGA TGTTACAGAG AGCCTCAGAT CATGAGTTCA
CWL029 ATTCAACCTA CAAAATATGA TGTTACAGAG AGCCTCAGAT CATGAGTTCA
J138 ATTCAACCTA CAAAATATGA TGTTACAGAG AGCCTCAGAT CATGAGTTCA
TW183 ATTCAACCTA CAAAATATGA TGTTACAGAG AGCCTCAGAT CATGAGTTCA
TOR1 ATTCAACCTA CAAAATATGA TGTTACAGAG AGCCTCAGAT CATGAGTTCA
WA97001 ATTCAACCTA CAAAATATGA TGTTACAGAG AGCCTCAGAT CATGAGTTCA
1979 ATTCAACCTA CAAAATATGA TGTTACAGAG AGCCTCAGAT CATGAGTTCA
SH511 ATTCAACCTA CAAAATATGA TGTTACAGAG AGCCTCAGAT CATGAGTTCA

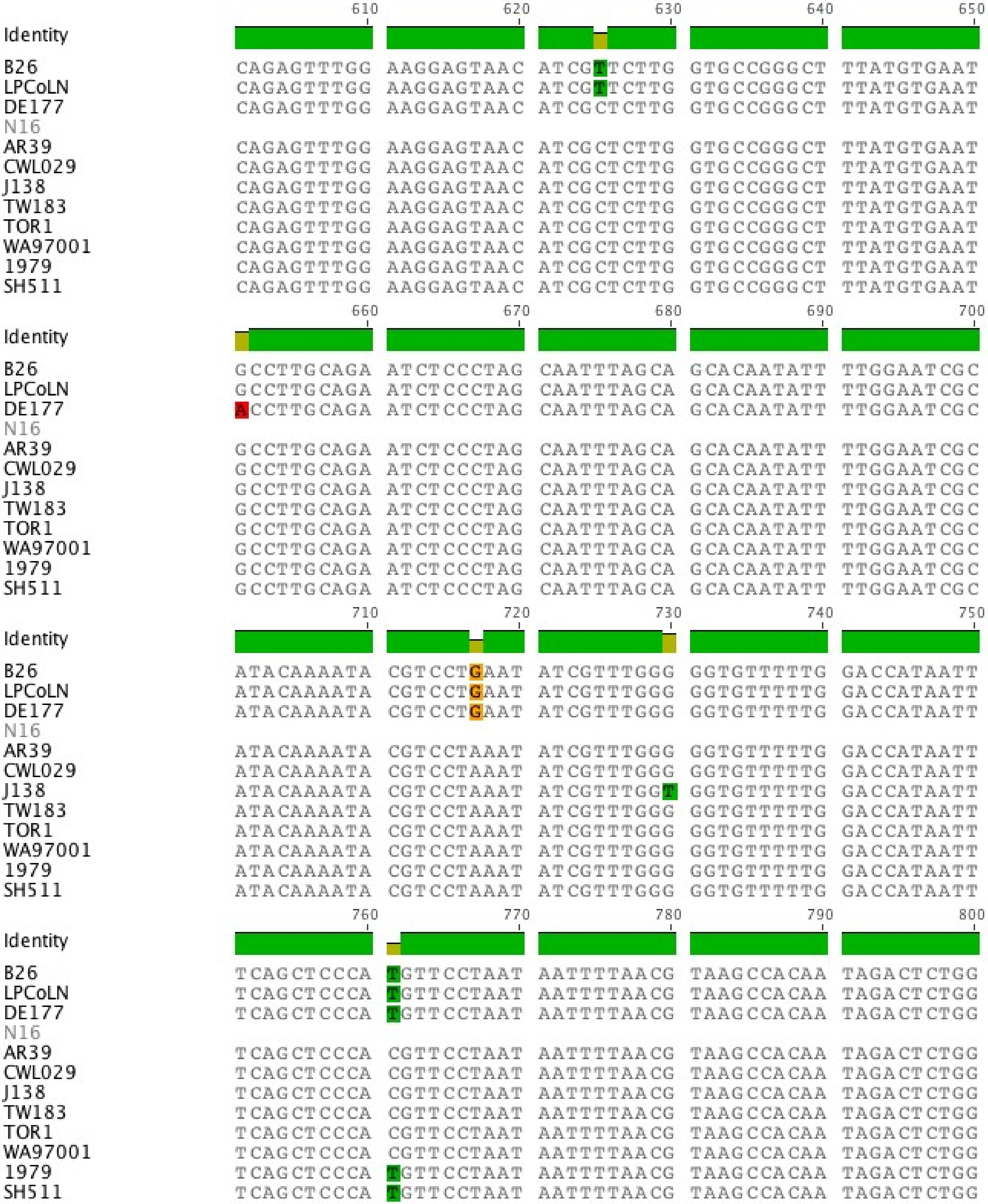

610 620 630 640 650
Identity
B26 CAGAGTTTGG AAGGAGTAAC ATCGTTCTTG GTGCCGGGCT TTATGTGAAT
LPCoLN CAGAGTTTGG AAGGAGTAAC ATCGTTCTTG GTGCCGGGCT TTATGTGAAT
DE177 CAGAGTTTGG AAGGAGTAAC ATCGCTCTTG GTGCCGGGCT TTATGTGAAT
N16
AR39 CAGAGTTTGG AAGGAGTAAC ATCGCTCTTG GTGCCGGGCT TTATGTGAAT
CWL029 CAGAGTTTGG AAGGAGTAAC ATCGCTCTTG GTGCCGGGCT TTATGTGAAT
J138 CAGAGTTTGG AAGGAGTAAC ATCGCTCTTG GTGCCGGGCT TTATGTGAAT
TW183 CAGAGTTTGG AAGGAGTAAC ATCGCTCTTG GTGCCGGGCT TTATGTGAAT
TOR1 CAGAGTTTGG AAGGAGTAAC ATCGCTCTTG GTGCCGGGCT TTATGTGAAT
WA97001 CAGAGTTTGG AAGGAGTAAC ATCGCTCTTG GTGCCGGGCT TTATGTGAAT
1979 CAGAGTTTGG AAGGAGTAAC ATCGCTCTTG GTGCCGGGCT TTATGTGAAT
SH511 CAGAGTTTGG AAGGAGTAAC ATCGCTCTTG GTGCCGGGCT TTATGTGAAT
660 670 680 690 700
Identity
B26 GCCTTGCAGA ATCTCCCTAG CAATTTAGCA GCACAATATT TTGGAATCGC
LPCoLN GCCTTGCAGA ATCTCCCTAG CAATTTAGCA GCACAATATT TTGGAATCGC
DE177 ACCTTGCAGA ATCTCCCTAG CAATTTAGCA GCACAATATT TTGGAATCGC
N16
AR39 GCCTTGCAGA ATCTCCCTAG CAATTTAGCA GCACAATATT TTGGAATCGC
CWL029 GCCTTGCAGA ATCTCCCTAG CAATTTAGCA GCACAATATT TTGGAATCGC
J138 GCCTTGCAGA ATCTCCCTAG CAATTTAGCA GCACAATATT TTGGAATCGC
TW183 GCCTTGCAGA ATCTCCCTAG CAATTTAGCA GCACAATATT TTGGAATCGC
TOR1 GCCTTGCAGA ATCTCCCTAG CAATTTAGCA GCACAATATT TTGGAATCGC
WA97001 GCCTTGCAGA ATCTCCCTAG CAATTTAGCA GCACAATATT TTGGAATCGC
1979 GCCTTGCAGA ATCTCCCTAG CAATTTAGCA GCACAATATT TTGGAATCGC
SH511 GCCTTGCAGA ATCTCCCTAG CAATTTAGCA GCACAATATT TTGGAATCGC
710 720 730 740 750
Identity
B26 ATACAAAATA CGTCCTGAAT ATCGTTTGGG GGTGTTTTTG GACCATAATT
LPCoLN ATACAAAATA CGTCCTGAAT ATCGTTTGGG GGTGTTTTTG GACCATAATT
DE177 ATACAAAATA CGTCCTGAAT ATCGTTTGGG GGTGTTTTTG GACCATAATT
N16
AR39 ATACAAAATA CGTCCTAAAT ATCGTTTGGG GGTGTTTTTG GACCATAATT
CWL029 ATACAAAATA CGTCCTAAAT ATCGTTTGGG GGTGTTTTTG GACCATAATT
J138 ATACAAAATA CGTCCTAAAT ATCGTTTGGT GGTGTTTTTG GACCATAATT
TW183 ATACAAAATA CGTCCTAAAT ATCGTTTGGG GGTGTTTTTG GACCATAATT
TOR1 ATACAAAATA CGTCCTAAAT ATCGTTTGGG GGTGTTTTTG GACCATAATT
WA97001 ATACAAAATA CGTCCTAAAT ATCGTTTGGG GGTGTTTTTG GACCATAATT
1979 ATACAAAATA CGTCCTAAAT ATCGTTTGGG GGTGTTTTTG GACCATAATT
SH511 ATACAAAATA CGTCCTAAAT ATCGTTTGGG GGTGTTTTTG GACCATAATT
760 770 780 790 800
Identity
B26 TCAGCTCCCA TGTTCCTAAT AATTTTAACG TAAGCCACAA TAGACTCTGG
LPCoLN TCAGCTCCCA TGTTCCTAAT AATTTTAACG TAAGCCACAA TAGACTCTGG
DE177 TCAGCTCCCA TGTTCCTAAT AATTTTAACG TAAGCCACAA TAGACTCTGG
N16
AR39 TCAGCTCCCA CGTTCCTAAT AATTTTAACG TAAGCCACAA TAGACTCTGG
CWL029 TCAGCTCCCA CGTTCCTAAT AATTTTAACG TAAGCCACAA TAGACTCTGG
J138 TCAGCTCCCA CGTTCCTAAT AATTTTAACG TAAGCCACAA TAGACTCTGG
TW183 TCAGCTCCCA CGTTCCTAAT AATTTTAACG TAAGCCACAA TAGACTCTGG
TOR1 TCAGCTCCCA CGTTCCTAAT AATTTTAACG TAAGCCACAA TAGACTCTGG
WA97001 TCAGCTCCCA CGTTCCTAAT AATTTTAACG TAAGCCACAA TAGACTCTGG
1979 TCAGCTCCCA TGTTCCTAAT AATTTTAACG TAAGCCACAA TAGACTCTGG
SH511 TCAGCTCCCA TGTTCCTAAT AATTTTAACG TAAGCCACAA TAGACTCTGG

	810	820	830	840	850
Identity					
B26	ATGGGAGCCT	TTATTGGATG	GCAGGATTCT	GATGCTCTAG	GATCTAGTGT
LPCoLN	ATGGGAGCCT	TTATTGGATG	GCAGGATTCT	GATGCTCTAG	GATCTAGTGT
DE177	ATGGGAGCCT	TTATTGGATG	GCAGGATTCT	GATGCTCTAG	GATCTAGTGT
N16					
AR39	ATGGGAGCCT	TTATTGGATG	GCAGGATTCT	GATGCTCTAG	GATCTAGTGT
CWL029	ATGGGAGCCT	TTATTGGATG	GCAGGATTCT	GATGCTCTAG	GATCTAGTGT
J138	ATGGGAGCCT	TTATTGGATG	GCAGGATTCT	GATGCTCTAG	GATCTAGTGT
TW183	ATGGGAGCCT	TTATTGGATG	GCAGGATTCT	GATGCTCTAG	GATCTAGTGT
TOR1	ATGGGAGCCT	TTATTGGATG	GCAGGATTCT	GATGCTCTAG	GATCTAGTGT
WA97001	ATGGGAGCCT	TTATTGGATG	GCAGGATTCT	GATGCTCTAG	GATCTAGTGT
1979	ATGGGAGCCT	TTATTGGATG	GCAGGATTCT	GATGCTCTAG	GATCTAGTGT
SH511	ATGGGAGCCT	TTATTGGATG	GCAGGATTCT	GATGCTCTAG	GATCTAGTGT

	860	870	880	890	900
Identity					
B26	CAAGGTGTCT	TTCGGATATG	GAAAACAAAA	AGCCACGATT	ACAAGAGAGC
LPCoLN	CAAGGTGTCT	TTCGGATATG	GAAAACAAAA	AGCCACGATT	ACAAGAGAGC
DE177	CAAGGTGTCT	TTCGGATATG	GAAAACAAAA	AGCCACGATT	ACAAGAGAGC
N16					
AR39	CAAGGTGTCT	TTCGGATATG	GAAAACAAAA	AGCCACGATT	ACAAGAGAGC
CWL029	CAAGGTGTCT	TTCGGATATG	GAAAACAAAA	AGCCACGATT	ACAAGAGAGC
J138	CAAGGTGTCT	TTCGGATATG	GAAAACAAAA	AGCCACGATT	ACAAGAGAGC
TW183	CAAGGTGTCT	TTCGGATATG	GAAAACAAAA	AGCCACGATT	ACAAGAGAGC
TOR1	CAAGGTGTCT	TTCGGATATG	GAAAACAAAA	AGCCACGATT	ACAAGAGAGC
WA97001	CAAGGTGTCT	TTCGGATATG	GAAAACAAAA	AGCCACGATT	ACAAGAGAGC
1979	CAAGGTGTCT	TTCGGATATG	GAAAACAAAA	AGCCACGATT	ACAAGAGAGC
SH511	CAAGGTGTCT	TTCGGATATG	GAAAACAAAA	AGCCACGATT	ACAAGAGAGC

	910	920	930	940	950
Identity					
B26	AATTAGAGAA	TACAGAAGCC	GGGAGTGGGG	AGAGCCATTT	TGAAGGGGTC
LPCoLN	AATTAGAGAA	TACAGAAGCC	GGGAGTGGGG	AGAGCCATTT	TGAAGGGGTC
DE177	AATTAGAGAA	TACAGAAGCC	GGGAGTGGGG	AGAGCCATTT	TGAAGGGGTC
N16					
AR39	AATTAGAGAA	TACAGAAGCC	GGGAGTGGGG	AGAGCCATTT	TGAAGGGGTC
CWL029	AATTAGAGAA	TACAGAAGCC	GGGAGTGGGG	AGAGCCATTT	TGAAGGGGTC
J138	AATTAGAGAA	TACAGAAGCC	GGGAGTGGGG	AGAGCCATTT	TGAAGGGGTC
TW183	AATTAGAGAA	TACAGAAGCC	GGGAGTGGGG	AGAGCCATTT	TGAAGGGGTC
TOR1	AATTAGAGAA	TACAGAAGCC	GGGAGTGGGG	AGAGCCATTT	TGAAGGGGTC
WA97001	AATTAGAGAA	TACAGAAGCC	GGGAGTGGGG	AGAGCCATTT	TGAAGGGGTC
1979	AATTAGAGAA	TACAGAAGCC	GGGAGTGGGG	AGAGCCATTT	TGAAGGGGTC
SH511	AATTAGAGAA	TACAGAAGCC	GGGAGTGGGG	AGAGCCATTT	TGAAGGGGTC

	960	970	980	990	1,000
Identity					
B26	GCTGCTCAGA	TAGAAGGGCG	GTATGGTAAG	AGCCTCGGAG	GACATGTCAG
LPCoLN	GCTGCTCAGA	TAGAAGGGCG	GTATGGTAAG	AGCCTCGGAG	GACATGTCAG
DE177	GCTGCTCAGA	TAGAAGGGCG	GTATGGTAAG	AGCCTCGGAG	GACATGTCAG
N16					
AR39	GCTGCTCAGA	TAGAAGGGCG	GTATGGTAAG	AGCCTCGGAG	GACATGTCAG
CWL029	GCTGCTCAGA	TAGAAGGGCG	GTATGGTAAG	AGCCTCGGAG	GACATGTCAG
J138	GCTGCTCAGA	TAGAAGGGCG	GTATGGTAAG	AGCCTCGGAG	GACATGTCAG
TW183	GCTGCTCAGA	TAGAAGGGCG	GTATGGTAAG	AGCCTCGGAG	GACATGTCAG
TOR1	GCTGCTCAGA	TAGAAGGGCG	GTATGGTAAG	AGCCTCGGAG	GACATGTCAG
WA97001	GCTGCTCAGA	TAGAAGGGCG	GTATGGTAAG	AGCCTCGGAG	GACATGTCAG
1979	GCTGCTCAGA	TAGAAGGGCG	GTATGGTAAG	AGCCTCGGAG	GACATGTCAG
SH511	GCTGCTCAGA	TAGAAGGGCG	GTATGGTAAG	AGCCTCGGAG	GACATGTCAG

	1,010	1,020	1,030	1,040	1,050
Identity					
B26	GGTCCAGCCT	TTCCTAGGAT	TGCAGTTTGT	CCACATTACA	AGGAAAGAAT
LPCoLN	GGTCCAGCCT	TTCCTAGGAT	TGCAGTTTGT	CCACATTACA	AGGAAAGAAT
DE177	GGTCCAGCCT	TTCCTAGGAT	TGCAGTTTGT	CCACATTACA	AGGAAAGAAT
N16					
AR39	GGTCCAGCCT	TTCCTAGGAC	TGCAGTTTGT	CCACATTACA	AGGAAAGAAT
CWL029	GGTCCAGCCT	TTCCTAGGAC	TGCAGTTTGT	CCACATTACA	AGGAAAGAAT
J138	GGTCCAGCCT	TTCCTAGGAC	TGCAGTTTGT	CCACATTACA	AGGAAAGAAT
TW183	GGTCCAGCCT	TTCCTAGGAC	TGCAGTTTGT	CCACATTACA	AGGAAAGAAT
TOR1	GGTCCAGCCT	TTCCTAGGAC	TGCAGTTTGT	CCACATTACA	AGGAAAGAAT
WA97001	GGTCCAGCCT	TTCCTAGGAC	TGCAGTTTGT	CCACATTACA	AGGAAAGAAT
1979	GGTCCAGCCT	TTCCTAGGAT	TGCAGTTTGT	CCACATTACA	AGGAAAGAAT
SH511	GGTCCAGCCT	TTCCTAGGAT	TGCAGTTTGT	CCACATTACA	AGGAAAGAAT

	1,060	1,070	1,080	1,090	1,100
Identity					
B26	ATACCGAAAA	TGGAGTGCAA	TTTCCTGTAC	ACTATGATCC	TATAGACTAT
LPCoLN	ATACCGAAAA	TGGAGTGCAA	TTTCCTGTAC	ACTATGATCC	TATAGACTAT
DE177	ATACCGAAAA	TGGAGTGCAA	TTTCCTGTAC	ACTATGATCC	TATAGACTAT
N16					
AR39	ATACCGAAAA	TGCAGTGCAA	TTTCCTGTAC	ACTATGATCC	TATAGACTAT
CWL029	ATACCGAAAA	TGCAGTGCAA	TTTCCTGTAC	ACTATGATCC	TATAGACTAT
J138	ATACCGAAAA	TGCAGTGCAA	TTTCCTGTAC	ACTATGATCC	TATAGACTAT
TW183	ATACCGAAAA	TGCAGTGCAA	TTTCCTGTAC	ACTATGATCC	TATAGACTAT
TOR1	ATACCGAAAA	TGCAGTGCAA	TTTCCTGTAC	ACTATGATCC	TATAGACTAT
WA97001	ATACCGAAAA	TGCAGTGCAA	TTTCCTGTAC	ACTATGATCC	TATAGACTAT
1979	ATACCGAAAA	TGCAGTGCAA	TTTCCTGTAC	ACTATGATCC	TATAGACTAT
SH511	ATACCGAAAA	TGCAGTGCAA	TTTCCTGTAC	ACTATGATCC	TATAGACTAT

	1,110	1,120	1,130	1,140	1,150
Identity					
B26	TCTACAGGTG	TAGTGTATTT	AGGAATTGGA	TCTCATATTG	CACTTGTAGA
LPCoLN	TCTACAGGTG	TAGTGTATTT	AGGAATTGGA	TCTCATATTG	CACTTGTAGA
DE177	TCTACAGGTG	TAGTGTATTT	AGGAATTGGA	TCTCATATTG	CACTTGTAGA
N16					
AR39	TCTACAGGTG	TAGTGTATTT	AGGAATTGGA	TCTCATATTG	CACTTGTAGA
CWL029	TCTACAGGTG	TAGTGTATTT	AGGAATTGGA	TCTCATATTG	CACTTGTAGA
J138	TCTACAGGTG	TAGTGTATTT	AGGAATTGGA	TCTCATATTG	CACTTGTAGA
TW183	TCTACAGGTG	TAGTGTATTT	AGGAATTGGA	TCTCATATTG	CACTTGTAGA
TOR1	TCTACAGGTG	TAGTGTATTT	AGGAATTGGA	TCTCATATTG	CACTTGTAGA
WA97001	TCTACAGGTG	TAGTGTATTT	AGGAATTGGA	TCTCATATTG	CACTTGTAGA
1979	TCTACAGGTG	TAGTGTATTT	AGGAATTGGA	TCTCATATTG	CACTTGTAGA
SH511	TCTACAGGTG	TAGTGTATTT	AGGAATTGGA	TCTCATATTG	CACTTGTAGA

	1,160	1,170	1,180	1,190	1,200
Identity					
B26	TTCTTTACAT	GTAGGCACAC	GCATGGGAAT	GGAGCAAAAC	TTTGCAGCCC
LPCoLN	TTCTTTACAT	GTAGGCACAC	GCATGGGAAT	GGAGCAAAAC	TTTGCAGCCC
DE177	TTCTTTACAT	GTAGGCATAC	GCATGGGAAT	GGAGCAAAAC	TTTGCAGCCC
N16					
AR39	TTCTTTACAT	GTAGGCACAC	GCATGGGAAT	GGAGCAAAAC	TTTGCAGCCC
CWL029	TTCTTTACAT	GTAGGCACAC	GCATGGGAAT	GGAGCAAAAC	TTTGCAGCCC
J138	TTCTTTACAT	GTAGGCACAC	GCATGGGAAT	GGAGCAAAAC	TTTGCAGCCC
TW183	TTCTTTACAT	GTAGGCACAC	GCATGGGAAT	GGAGCAAAAC	TTTGCAGCCC
TOR1	TTCTTTACAT	GTAGGCACAC	GCATGGGAAT	GGAGCAAAAC	TTTGCAGCCC
WA97001	TTCTTTACAT	GTAGGCACAC	GCATGGGAAT	GGAGCAAAAC	TTTGCAGCCC
1979	TTCTTTACAT	GTAGGCACAC	GCATGGGAAT	GGAGCAAAAC	TTTGCAGCCC
SH511	TTCTTTACAT	GTAGGCACAC	GCATGGGAAT	GGAGCAAAAC	TTTGCAGCCC

```
                     1,210      1,220      1,230      1,240      1,250
Identity
B26        ATACGGATAG GTTCTCAGGA TCTATAGCGT CTATTGGAAA CTTTGTGTTT
LPCoLN     ATACGGATAG GTTCTCAGGA TCTATAGCGT CTATTGGAAA CTTTGTGTTT
DE177      ATACGGACAG GTTCTCAGGA TCTATAGCGT CTATTGGAAA CTTTGTGTTT
N16
AR39       ATACGGACAG GTTCTCAGGA TCTATAGCGT CTATTGGAAA CTTTGTGTTT
CWL029     ATACGGACAG GTTCTCAGGA TCTATAGCGT CTATTGGAAA CTTTGTGTTT
J138       ATACGGACAG GTTCTCAGGA TCTATAGCGT CTATTGGAAA CTTTGTGTTT
TW183      ATACGGACAG GTTCTCAGGA TCTATAGCGT CTATTGGAAA CTTTGTGTTT
TOR1       ATACGGACAG GTTCTCAGGA TCTATAGCGT CTATTGGAAA CTTTGTGTTT
WA97001    ATACGGACAG GTTCTCAGGA TCTATAGCGT CTATTGGAAA CTTTGTGTTT
1979       ATACGGACAG GTTCTCAGGA TCTATAGCGT CTATTGGAAA CTTTGTGTTT
SH511      ATACGGACAG GTTCTCAGGA TCTATAGCGT CTATTGGAAA CTTTGTGTTT

                     1,260      1,270      1,280      1,290      1,300
Identity
B26        GAAAAGCTTG ATGTGACTCA CACAAGGGCA TTTGCGGAAA TGCGTGTCAA
LPCoLN     GAAAAGCTTG ATGTGACTCA CACAAGGGCA TTTGCGGAAA TGCGTGTCAA
DE177      GAAAAGCTTG ATGTGACTCA CACAAGGGCA TTTGCGGAAA TGCCTGTCAA
N16
AR39       GAAAAGCTTG ATGTGACTCA CACAAGGGCA TTTGCGGAAA TGCGTGTCAA
CWL029     GAAAAGCTTG ATGTGACTCA CACAAGGGCA TTTGCGGAAA TGCGTGTCAA
J138       GAAAAGCTTG ATGTGACTCA CACAAGGGCA TTTGCGGAAA TGCGTGTCAA
TW183      GAAAAGCTTG ATGTGACTCA CACAAGGGCA TTTGCGGAAA TGCGTGTCAA
TOR1       GAAAAGCTTG ATGTGACTCA CACAAGGGCA TTTGCGGAAA TGCGTGTCAA
WA97001    GAAAAGCTTG ATGTGACTCA CACAAGGGCA TTTGCGGAAA TGCGTGTCAA
1979       GAAAAGCTTG ATGTGACTCA CACAAGGGCA TTTGCGGAAA TGCGTGTCAA
SH511      GAAAAGCTTG ATGTGACTCA CACAAGGGCA TTTGCGGAAA TGCGTGTCAA

                     1,310      1,320      1,330      1,340      1,350
Identity
B26        CTATGAGCTT CCCTATCTAC AGTCTCTGAA TCTTATTCTA CGAGTTAATC
LPCoLN     CTATGAGCTT CCCTATCTAC AGTCTCTGAA TCTTATTCTA CGAGTTAATC
DE177      CTATGAGCTT CCCTATCTAC AGTCTCTGAA TCTTATTCTA CGAGTTAATC
N16
AR39       CTATGAGCTT CCCTATCTAC AGTCTCTGAA TCTTATTCTA CGAGTTAATC
CWL029     CTATGAGCTT CCCTATCTAC AGTCTCTGAA TCTTATTCTA CGAGTTAATC
J138       CTATGAGCTT CCCTATCTAC AGTCTCTGAA TCTTATTCTA CGAGTTAATC
TW183      CTATGAGCTT CCCTATCTAC AGTCTCTGAA TCTTATTCTA CGAGTTAATC
TOR1       CTATGAGCTT CCCTATCTAC AGTCTCTGAA TCTTATTCTA CGAGTTAATC
WA97001    CTATGAGCTT CCCTATCTAC AGTCTCTGAA TCTTATTCTA CGAGTTAATC
1979       CTATGAGCTT CCCTATCTAC AGTCTCTGAA TCTTATTCTA CGAGTTAATC
SH511      CTATGAGCTT CCCTATCTAC AGTCTCTGAA TCTTATTCTA CGAGTTAATC

                     1,360      1,370      1,380      1,390      1,400
Identity
B26        AACAGCCTCT ACAAGGGGTT ATGGGATTTT CCAGTGATCT TAGGTATGCC
LPCoLN     AACAGCCTCT ACAAGGGGTT ATGGGATTTT CCAGTGATCT TAGGTATGCC
DE177      AACAGCCTCT ACAAGGGGTT ATGGGATTTT CCAGTGATCT TAGGTATGCC
N16
AR39       AACAGCCTCT ACAAGGGGTT ATGGGATTTT CCAGTGATCT TAGGTATGCC
CWL029     AACAGCCTCT ACAAGGGGTT ATGGGATTTT CCAGTGATCT TAGGTATGCC
J138       AACAGCCTCT ACAAGGGGTT ATGGGATTTT CCAGTGATCT TAGGTATGCC
TW183      AACAGCCTCT ACAAGGGGTT ATGGGATTTT CCAGTGATCT TAGGTATGCC
TOR1       AACAGCCTCT ACAAGGGGTT ATGGGATTTT CCAGTGATCT TAGGTATGCC
WA97001    AACAGCCTCT ACAAGGGGTT ATGGGATTTT CCAGTGATCT TAGGTATGCC
1979       AACAGCCTCT ACAAGGGGTT ATGGGATTTT CCAGTGATCT TAGGTATGCC
SH511      AACAGCCTCT ACAAGGGGTT ATGGGATTTT CCAGTGATCT TAGGTATGCC

                     1,410  1,413
Identity
B26        TTAGGATTCT AA
LPCoLN     TTAGGATTCT AA
DE177      TTAGGATTCT AA
N16
AR39       TTAGGATTCT AAC
CWL029     TTAGGATTCT AA
J138       TTAGGATTCT AA
TW183      TTAGGATTCT AA
TOR1       TTAGGATTCT AA
WA97001    TTAGGATTCT AA
1979       TTAGGATTCT AA
SH511      TTAGGATTCT AA
```

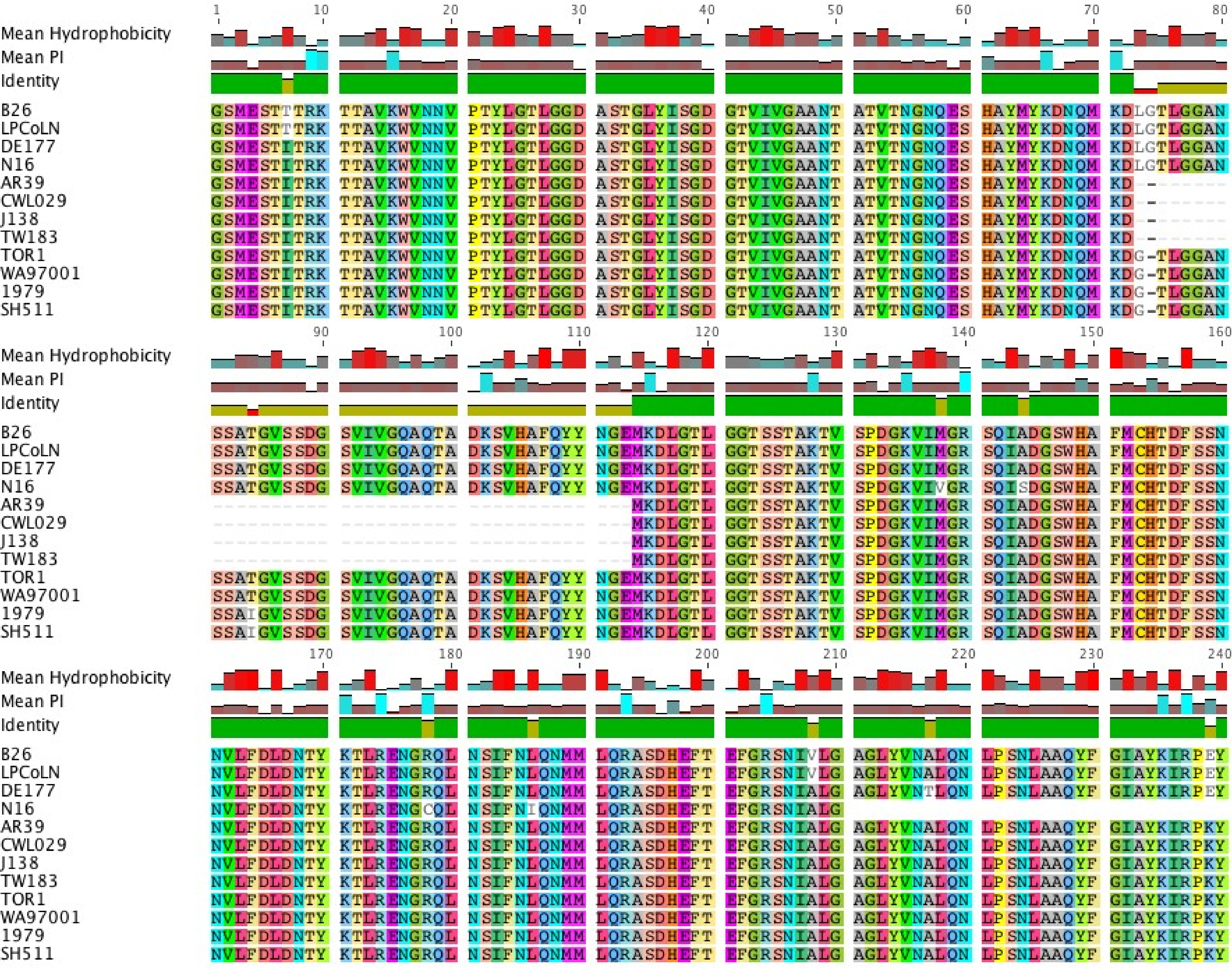

1 10 20 30 40 50 60 70 80
Mean Hydrophobicity
Mean PI
Identity
B26 GSMESTTTRK TTAVKWVNNV PTYLGTLGGD ASTGLYISGD GTVIVGAANT ATVTNGNQES HAYMYKDNQM KDLGTLGGAN
LPCoLN GSMESTTTRK TTAVKWVNNV PTYLGTLGGD ASTGLYISGD GTVIVGAANT ATVTNGNQES HAYMYKDNQM KDLGTLGGAN
DE177 GSMESTITRK TTAVKWVNNV PTYLGTLGGD ASTGLYISGD GTVIVGAANT ATVTNGNQES HAYMYKDNQM KDLGTLGGAN
N16 GSMESTITRK TTAVKWVNNV PTYLGTLGGD ASTGLYISGD GTVIVGAANT ATVTNGNQES HAYMYKDNQM KDLGTLGGAN
AR39 GSMESTITRK TTAVKWVNNV PTYLGTLGGD ASTGLYISGD GTVIVGAANT ATVTNGNQES HAYMYKDNQM KD--------
CWL029 GSMESTITRK TTAVKWVNNV PTYLGTLGGD ASTGLYISGD GTVIVGAANT ATVTNGNQES HAYMYKDNQM KD--------
J138 GSMESTITRK TTAVKWVNNV PTYLGTLGGD ASTGLYISGD GTVIVGAANT ATVTNGNQES HAYMYKDNQM KD--------
TW183 GSMESTITRK TTAVKWVNNV PTYLGTLGGD ASTGLYISGD GTVIVGAANT ATVTNGNQES HAYMYKDNQM KD--------
TOR1 GSMESTITRK TTAVKWVNNV PTYLGTLGGD ASTGLYISGD GTVIVGAANT ATVTNGNQES HAYMYKDNQM KDG-TLGGAN
WA97001 GSMESTITRK TTAVKWVNNV PTYLGTLGGD ASTGLYISGD GTVIVGAANT ATVTNGNQES HAYMYKDNQM KDG-TLGGAN
1979 GSMESTITRK TTAVKWVNNV PTYLGTLGGD ASTGLYISGD GTVIVGAANT ATVTNGNQES HAYMYKDNQM KDG-TLGGAN
SH511 GSMESTITRK TTAVKWVNNV PTYLGTLGGD ASTGLYISGD GTVIVGAANT ATVTNGNQES HAYMYKDNQM KDG-TLGGAN
90 100 110 120 130 140 150 160
Mean Hydrophobicity
Mean PI
Identity
B26 SSATGVSSDG SVIVGQAQTA DKSVHAFQYY NGEMKDLGTL GGTSSTAKTV SPDGKVIMGR SQIADGSWHA FMCHTDFSSN
LPCoLN SSATGVSSDG SVIVGQAQTA DKSVHAFQYY NGEMKDLGTL GGTSSTAKTV SPDGKVIMGR SQIADGSWHA FMCHTDFSSN
DE177 SSATGVSSDG SVIVGQAQTA DKSVHAFQYY NGEMKDLGTL GGTSSTAKTV SPDGKVIMGR SQIADGSWHA FMCHTDFSSN
N16 SSATGVSSDG SVIVGQAQTA DKSVHAFQYY NGEMKDLGTL GGTSSTAKTV SPDGKVIVGR SQISDGSWHA FMCHTDFSSN
AR39 ---------- ---------- ---------- ---MKDLGTL GGTSSTAKTV SPDGKVIMGR SQIADGSWHA FMCHTDFSSN
CWL029 ---------- ---------- ---------- ---MKDLGTL GGTSSTAKTV SPDGKVIMGR SQIADGSWHA FMCHTDFSSN
J138 ---------- ---------- ---------- ---MKDLGTL GGTSSTAKTV SPDGKVIMGR SQIADGSWHA FMCHTDFSSN
TW183 ---------- ---------- ---------- ---MKDLGTL GGTSSTAKTV SPDGKVIMGR SQIADGSWHA FMCHTDFSSN
TOR1 SSATGVSSDG SVIVGQAQTA DKSVHAFQYY NGEMKDLGTL GGTSSTAKTV SPDGKVIMGR SQIADGSWHA FMCHTDFSSN
WA97001 SSATGVSSDG SVIVGQAQTA DKSVHAFQYY NGEMKDLGTL GGTSSTAKTV SPDGKVIMGR SQIADGSWHA FMCHTDFSSN
1979 SSAIGVSSDG SVIVGQAQTA DKSVHAFQYY NGEMKDLGTL GGTSSTAKTV SPDGKVIMGR SQIADGSWHA FMCHTDFSSN
SH511 SSAIGVSSDG SVIVGQAQTA DKSVHAFQYY NGEMKDLGTL GGTSSTAKTV SPDGKVIMGR SQIADGSWHA FMCHTDFSSN
170 180 190 200 210 220 230 240
Mean Hydrophobicity
Mean PI
Identity
B26 NVLFDLDNTY KTLRENGRQL NSIFNLQNMM LQRASDHEFT EFGRSNIVLG AGLYVNALQN LPSNLAAQYF GIAYKIRPEY
LPCoLN NVLFDLDNTY KTLRENGRQL NSIFNLQNMM LQRASDHEFT EFGRSNIVLG AGLYVNALQN LPSNLAAQYF GIAYKIRPEY
DE177 NVLFDLDNTY KTLRENGRQL NSIFNLQNMM LQRASDHEFT EFGRSNIALG AGLYVNTLQN LPSNLAAQYF GIAYKIRPEY
N16 NVLFDLDNTY KTLRENGCQL NSIFNIQNMM LQRASDHEFT EFGRSNIALG
AR39 NVLFDLDNTY KTLRENGRQL NSIFNLQNMM LQRASDHEFT EFGRSNIALG AGLYVNALQN LPSNLAAQYF GIAYKIRPKY
CWL029 NVLFDLDNTY KTLRENGRQL NSIFNLQNMM LQRASDHEFT EFGRSNIALG AGLYVNALQN LPSNLAAQYF GIAYKIRPKY
J138 NVLFDLDNTY KTLRENGRQL NSIFNLQNMM LQRASDHEFT EFGRSNIALG AGLYVNALQN LPSNLAAQYF GIAYKIRPKY
TW183 NVLFDLDNTY KTLRENGRQL NSIFNLQNMM LQRASDHEFT EFGRSNIALG AGLYVNALQN LPSNLAAQYF GIAYKIRPKY
TOR1 NVLFDLDNTY KTLRENGRQL NSIFNLQNMM LQRASDHEFT EFGRSNIALG AGLYVNALQN LPSNLAAQYF GIAYKIRPKY
WA97001 NVLFDLDNTY KTLRENGRQL NSIFNLQNMM LQRASDHEFT EFGRSNIALG AGLYVNALQN LPSNLAAQYF GIAYKIRPKY
1979 NVLFDLDNTY KTLRENGRQL NSIFNLQNMM LQRASDHEFT EFGRSNIALG AGLYVNALQN LPSNLAAQYF GIAYKIRPKY
SH511 NVLFDLDNTY KTLRENGRQL NSIFNLQNMM LQRASDHEFT EFGRSNIALG AGLYVNALQN LPSNLAAQYF GIAYKIRPKY

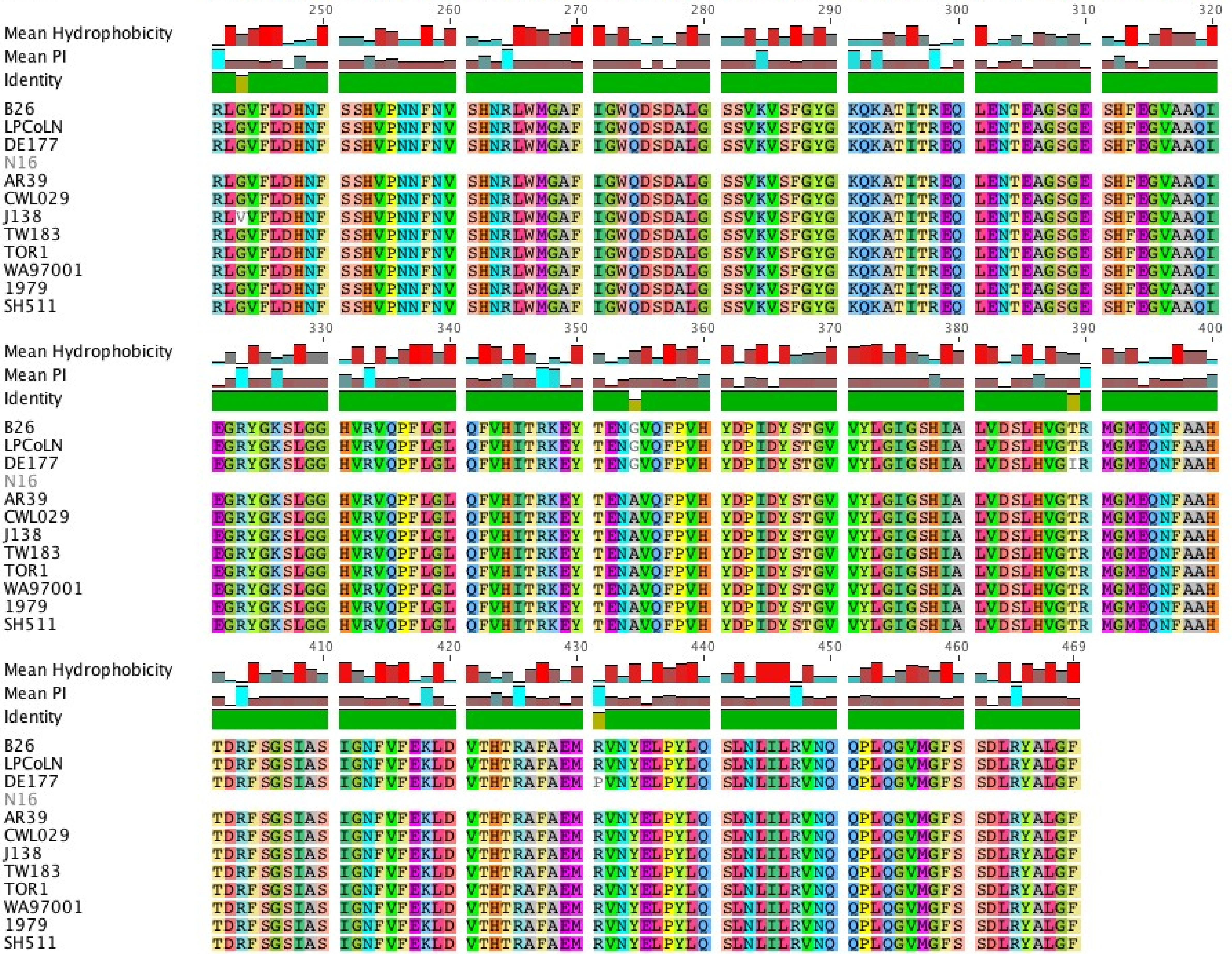

250 260 270 280 290 300 310 320
Mean Hydrophobicity
Mean PI
Identity
B26 RLGVFLDHNF SSHVPNNFNV SHNRLWMGAF IGWQDSDALG SSVKVSFGYG KQKATITREQ LENTEAGSGE SHFEGVAAQI
LPCoLN RLGVFLDHNF SSHVPNNFNV SHNRLWMGAF IGWQDSDALG SSVKVSFGYG KQKATITREQ LENTEAGSGE SHFEGVAAQI
DE177 RLGVFLDHNF SSHVPNNFNV SHNRLWMGAF IGWQDSDALG SSVKVSFGYG KQKATITREQ LENTEAGSGE SHFEGVAAQI
N16
AR39 RLGVFLDHNF SSHVPNNFNV SHNRLWMGAF IGWQDSDALG SSVKVSFGYG KQKATITREQ LENTEAGSGE SHFEGVAAQI
CWL029 RLGVFLDHNF SSHVPNNFNV SHNRLWMGAF IGWQDSDALG SSVKVSFGYG KQKATITREQ LENTEAGSGE SHFEGVAAQI
J138 RLVVFLDHNF SSHVPNNFNV SHNRLWMGAF IGWQDSDALG SSVKVSFGYG KQKATITREQ LENTEAGSGE SHFEGVAAQI
TW183 RLGVFLDHNF SSHVPNNFNV SHNRLWMGAF IGWQDSDALG SSVKVSFGYG KQKATITREQ LENTEAGSGE SHFEGVAAQI
TOR1 RLGVFLDHNF SSHVPNNFNV SHNRLWMGAF IGWQDSDALG SSVKVSFGYG KQKATITREQ LENTEAGSGE SHFEGVAAQI
WA97001 RLGVFLDHNF SSHVPNNFNV SHNRLWMGAF IGWQDSDALG SSVKVSFGYG KQKATITREQ LENTEAGSGE SHFEGVAAQI
1979 RLGVFLDHNF SSHVPNNFNV SHNRLWMGAF IGWQDSDALG SSVKVSFGYG KQKATITREQ LENTEAGSGE SHFEGVAAQI
SH511 RLGVFLDHNF SSHVPNNFNV SHNRLWMGAF IGWQDSDALG SSVKVSFGYG KQKATITREQ LENTEAGSGE SHFEGVAAQI
330 340 350 360 370 380 390 400
Mean Hydrophobicity
Mean PI
Identity
B26 EGRYGKSLGG HVRVQPFLGL QFVHITRKEY TENGVQFPVH YDPIDYSTGV VYLGIGSHIA LVDSLHVGTR MGMEQNFAAH
LPCoLN EGRYGKSLGG HVRVQPFLGL QFVHITRKEY TENGVQFPVH YDPIDYSTGV VYLGIGSHIA LVDSLHVGTR MGMEQNFAAH
DE177 EGRYGKSLGG HVRVQPFLGL QFVHITRKEY TENGVQFPVH YDPIDYSTGV VYLGIGSHIA LVDSLHVGIR MGMEQNFAAH
N16
AR39 EGRYGKSLGG HVRVQPFLGL QFVHITRKEY TENAVQFPVH YDPIDYSTGV VYLGIGSHIA LVDSLHVGTR MGMEQNFAAH
CWL029 EGRYGKSLGG HVRVQPFLGL QFVHITRKEY TENAVQFPVH YDPIDYSTGV VYLGIGSHIA LVDSLHVGTR MGMEQNFAAH
J138 EGRYGKSLGG HVRVQPFLGL QFVHITRKEY TENAVQFPVH YDPIDYSTGV VYLGIGSHIA LVDSLHVGTR MGMEQNFAAH
TW183 EGRYGKSLGG HVRVQPFLGL QFVHITRKEY TENAVQFPVH YDPIDYSTGV VYLGIGSHIA LVDSLHVGTR MGMEQNFAAH
TOR1 EGRYGKSLGG HVRVQPFLGL QFVHITRKEY TENAVQFPVH YDPIDYSTGV VYLGIGSHIA LVDSLHVGTR MGMEQNFAAH
WA97001 EGRYGKSLGG HVRVQPFLGL QFVHITRKEY TENAVQFPVH YDPIDYSTGV VYLGIGSHIA LVDSLHVGTR MGMEQNFAAH
1979 EGRYGKSLGG HVRVQPFLGL QFVHITRKEY TENAVQFPVH YDPIDYSTGV VYLGIGSHIA LVDSLHVGTR MGMEQNFAAH
SH511 EGRYGKSLGG HVRVQPFLGL QFVHITRKEY TENAVQFPVH YDPIDYSTGV VYLGIGSHIA LVDSLHVGTR MGMEQNFAAH
410 420 430 440 450 460 469
Mean Hydrophobicity
Mean PI
Identity
B26 TDRFSGSIAS IGNFVFEKLD VTHTRAFAEM RVNYELPYLQ SLNLILRVNQ QPLQGVMGFS SDLRYALGF
LPCoLN TDRFSGSIAS IGNFVFEKLD VTHTRAFAEM RVNYELPYLQ SLNLILRVNQ QPLQGVMGFS SDLRYALGF
DE177 TDRFSGSIAS IGNFVFEKLD VTHTRAFAEM PVNYELPYLQ SLNLILRVNQ QPLQGVMGFS SDLRYALGF
N16
AR39 TDRFSGSIAS IGNFVFEKLD VTHTRAFAEM RVNYELPYLQ SLNLILRVNQ QPLQGVMGFS SDLRYALGF
CWL029 TDRFSGSIAS IGNFVFEKLD VTHTRAFAEM RVNYELPYLQ SLNLILRVNQ QPLQGVMGFS SDLRYALGF
J138 TDRFSGSIAS IGNFVFEKLD VTHTRAFAEM RVNYELPYLQ SLNLILRVNQ QPLQGVMGFS SDLRYALGF
TW183 TDRFSGSIAS IGNFVFEKLD VTHTRAFAEM RVNYELPYLQ SLNLILRVNQ QPLQGVMGFS SDLRYALGF
TOR1 TDRFSGSIAS IGNFVFEKLD VTHTRAFAEM RVNYELPYLQ SLNLILRVNQ QPLQGVMGFS SDLRYALGF
WA97001 TDRFSGSIAS IGNFVFEKLD VTHTRAFAEM RVNYELPYLQ SLNLILRVNQ QPLQGVMGFS SDLRYALGF
1979 TDRFSGSIAS IGNFVFEKLD VTHTRAFAEM RVNYELPYLQ SLNLILRVNQ QPLQGVMGFS SDLRYALGF
SH511 TDRFSGSIAS IGNFVFEKLD VTHTRAFAEM RVNYELPYLQ SLNLILRVNQ QPLQGVMGFS SDLRYALGF
