# Supplementary material for: Chlamydia pneumoniae Is Genetically Diverse in Animals and Appears to Have Crossed the Host Barrier to Humans on (At Least) Two Occasions
Source: PLoS Pathog. 2010 May 20;6(5):e1000903. doi: 10.1371/journal.ppat.1000903 (PMC2873915; doi:10.1371/journal.ppat.1000903)
Supplement: Figure S17 — Multiple sequence alignment of guaB . The nucleotide and amino acid alignments were generated using Geneious version 4.7, where each nucleotide and amino acid is assigned its own colour. White shading indicates an amino acid variant. (1.26 MB PDF) [file ppat.1000903.s017.pdf]

	1–10	20	30	40	50
DE177	CAGTTTTAGA	AATAAAATCC	CAGTTCCCAC	AAATTTCTTT	AGTTGTAGGG
N16	CAGTTTTAGA	AATAAAATCC	TAGTTCCCAC	AAATTTCTTT	AGTTGTAGGG
AR39	CAGTTTTAGA	AATAAAATCC	CAGTTCCCAC	AAATTTCTTT	AGTTGTAGGG
CWL029	CAGTTTTAGA	AATAAAATCC	CAGTTCCCAC	AAATTTCTTT	AGTTGTAGGG
TW183	CAGTTTTAGA	AATAAAATCC	CAGTTCCCAC	AAATTTCTTT	AGTTGTAGGG
IOL207	CAGTTTTAGA	AATAAAATCC	CAGTTCCCAC	AAATTTCTTT	AGTTGTAGGG
TOR1	CAGTTTTAGA	AATAAAATCC	CAGTTCCCAC	AAATTTCTTT	AGTTGTAGGG
WA97001	CAGTTTTAGA	AATAAAATCC	CAGTTCCCAC	AAATTTCTTT	AGTTGTAGGG
1979	CAGTTTTAGA	AATAAAATCC	CAGTTCCCAC	AAATTTCTTT	AGTTGTAGGG
SH511	CAGTTTTAGA	AATAAAATCC	CAGTTCCCAC	AAATTTCTTT	AGTTGTAGGG

	60	70	80	90	100
DE177	AATCTTGTTA	TAGCTGAAGC	CGCAGTTTCC	TTAGCTGAGA	TTGGAGTTGA
N16	AATCTTGTTA	CAGCTGAAGC	CGCAGTTTCC	TTAGCTGAGA	TTGGAGTTGA
AR39	AATCTTGTTA	CAGCTGAAGC	CGCAGTTTCC	TTAGCTGAGA	TTGGAGTTGA
CWL029	AATCTTGTTA	CAGCTGAAGC	CGCAGTTTCC	TTAGCTGAGA	TTGGAGTTGA
TW183	AATCTTGTTA	CAGCTGAAGC	CGCAGTTTCC	TTAGCTGAGA	TTGGAGTTGA
IOL207	AATCTTGTTA	CAGCTGAAGC	CGCAGTTTCC	TTAGCTGAGA	TTGGAGTTGA
TOR1	AATCTTGTTA	CAGCTGAAGC	CGCAGTTTCC	TTAGCTGAGA	TTGGAGTTGA
WA97001	AATCTTGTTA	CAGCTGAAGC	CGCAGTTTCC	TTAGCTGAGA	TTGGAGTTGA
1979	AATCTTGTTA	CAGCTGAAGC	TGCAGTCTCC	TTAGCTGAGA	TTGGAGTTGA
SH511	AATCTTGTTA	CAGCTGAAGC	TGCAGTCTCC	TTAGCTGAGA	TTGGAGTTGA

	110	120	130	140	150
DE177	CGCTGTAAAG	GTAGGTATTG	GCCCAGGATC	TATCTGTACA	ACTAGAATCG
N16	CGCTGTAAAG	GTAGGTATTG	GCCCAGGATC	TATCTGTACA	ACTAGAATCG
AR39	CGCTGTAAAG	GTAGGTATTG	GCCCAGGATC	TATCTGTACA	ACTAGAATCG
CWL029	CGCTGTAAAG	GTAGGTATTG	GCCCAGGATC	TATCTGTACA	ACTAGAATCG
TW183	CGCTGTAAAG	GTAGGTATTG	GCCCAGGATC	TATCTGTACA	ACTAGAATCG
IOL207	CGCTGTAAAG	GTAGGTATTG	GCCCAGGATC	TATCTGTACA	ACTAGAATCG
TOR1	CGCTGTAAAG	GTAGGTATTG	GCCCAGGATC	TATCTGTACA	ACTAGAATCG
WA97001	CGCTGTAAAG	GTAGGTATTG	GCCCAGGATC	TATCTGTACA	ACTAGAATCG
1979	CGCTGTAAAG	GTAGGTATTG	GCCCAGGATC	TATCTGTACA	ACTAGAATCG
SH511	CGCTGTAAAG	GTAGGTATTG	GCCCAGGATC	TATCTGTACA	ACTAGAATCG

	160	170	180	190	200
DE177	TTTCAGGGGT	CGGTTATCCA	CAAATTACTG	CCATTACAAA	CGTAGCAAAA
N16	TTTCAGGGGT	CGGTTATCCA	TAAATTACTG	CCATTACAAA	CGTAGCAAAA
AR39	TTTCAGGGGT	CGGTTATCCA	CAAATTACTG	CCATTACAAA	CGTAGCAAAA
CWL029	TTTCAGGGGT	CGGTTATCCA	CAAATTACTG	CCATTACAAA	CGTAGCAAAA
TW183	TTTCAGGGGT	CGGTTATCCA	CAAATTACTG	CCATTACAAA	CGTAGCAAAA
IOL207	TTTCAGGGGT	CGGTTATCCA	CAAATTACTG	CCATTACAAA	CGTAGCAAAA
TOR1	TTTCAGGGGT	CGGTTATCCA	CAAATTACTG	CCATTACAAA	CGTAGCAAAA
WA97001	TTTCAGGGGT	CGGTTATCCA	CAAATTACTG	CCATTACAAA	CGTAGCAAAA
1979	TTTCAGGGGT	CGGTTATCCA	CAAATTACTG	CCATTACAAA	CGTAGCAAAA
SH511	TTTCAGGGGT	CGGTTATCCA	CAAATTACTG	CCATTACAAA	CGTAGCAAAA

	210	220	230	240	250
Identity					
DE177	GCTCTTAAAA	ACTCTGCCGT	GACTGTAATT	GCTGATGGGG	GAATCCGCTA
N16	GCTCTTAAAA	ACTCTGCCGT	GACTGTAATT	GCTGATGGGG	GAATCCACTA
AR39	GCTCTTAAAA	ACTCTGCCGT	GACTGTAATT	GCTGATGGGA	GAATCCGCTA
CWL029	GCTCTTAAAA	ACTCTGCCGT	GACTGTAATT	GCTGATGGGA	GAATCCGCTA
TW183	GCTCTTAAAA	ACTCTGCCGT	GACTGTAATT	GCTGATGGGA	GAATCCGCTA
IOL207	GCTCTTAAAA	ACTCTGCCGT	GACTGTAATT	GCTGATGGGA	GAATCCGCTA
TOR1	GCTCTTAAAA	ACTCTGCCGT	GACTGTAATT	GCTGATGGGA	GAATCCGCTA
WA97001	GCTCTTAAAA	ACTCTGCCGT	GACTGTAATT	GCTGATGGGA	GAATCCGCTA
1979	GCTCTTAAAA	ACTCTGCCGT	GACTGTAATT	GCTGATGGGG	GAATCCGCTA
SH511	GCTCTTAAAA	ACTCTGCCGT	GACTGTAATT	GCTGATGGGG	GAATCCGCTA

	260	270	280	290	300
Identity					
DE177	TTCTGGAGAT	GTGGTAAAAG	CATTAGCAGC	AGGAGCAGAC	TGTGTCATGC
N16	TTCTGGAGAT	GTGGTAAAAG	CATTAGCAGC	AGGAGCAGAC	TGTGTCATGC
AR39	TTCTGGAGAT	GTGGTAAAAG	CATTAGCAGC	AGGAGCAGAC	TGTGTCATGC
CWL029	TTCTGGAGAT	GTGGTAAAAG	CATTAGCAGC	AGGAGCAGAC	TGTGTCATGC
TW183	TTCTGGAGAT	GTGGTAAAAG	CATTAGCAGC	AGGAGCAGAC	TGTGTCATGC
IOL207	TTCTGGAGAT	GTGGTAAAAG	CATTAGCAGC	AGGAGCAGAC	TGTGTCATGC
TOR1	TTCTGGAGAT	GTGGTAAAAG	CATTAGCAGC	AGGAGCAGAC	TGTGTCATGC
WA97001	TTCTGGAGAT	GTGGTAAAAG	CATTAGCAGC	AGGAGCAGAC	TGTGTCATGC
1979	TTCTGGAGAT	GTGGTAAAAG	CATTAGCAGC	AGGAGCAGAC	TGTGTCATGC
SH511	TTCTGGAGAT	GTGGTAAAAG	CATTAGCAGC	AGGAGCAGAC	TGTGTCATGC

	310	320	330	340	350
Identity					
DE177	TAGGAAGTTT	GCTTGCAGGG	ACTGATGAAG	CTCCTGGGGA	TATCGTTTCT
N16	TAGGAAGTTT	GCTTGCAGGG	ACTGATGAAG	CTCCTGGGGA	TATCGTTTCT
AR39	TAGGAAGTTT	GCTTGCAGGG	ACTGATGAAG	CTCCTGGGGA	TATCGTTTCT
CWL029	TAGGAAGTTT	GCTTGCAGGG	ACTGATGAAG	CTCCTGGGGA	TATCGTTTCT
TW183	TAGGAAGTTT	GCTTGCAGGG	ACTGATGAAG	CTCCTGGGGA	TATCGTTTCT
IOL207	TAGGAAGTTT	GCTTGCAGGG	ACTGATGAAG	CTCCTGGGGA	TATCGTTTCT
TOR1	TAGGAAGTTT	GCTTGCAGGG	ACTGATGAAG	CTCCTGGGGA	TATCGTTTCT
WA97001	TAGGAAGTTT	GCTTGCAGGG	ACTGATGAAG	CTCCTGGGGA	TATCGTTTCT
1979	TAGGAAGTTT	GCTTGCAGGG	ACTGCTGAAG	CTCCTGGGGA	TATCGTTTCT
SH511	TAGGAAGTTT	GCTTGCAGGG	ACTGCTGAAG	CTCCTGGGGA	TATCGTTTCT

	360	370	380	390	400
Identity					
DE177	ATCGATGAGA	AGCTTTTTAA	AAGGTACCGC	GGCATGGGAT	CTTTAGGCGC
N16	ATCGATGAGA	AGCTTTTTAA	AAGGTACCGC	GGCATGGGAT	CTTTAGGCGC
AR39	ATCGATGAGA	AGCTTTTTAA	AAGGTACCGC	GGCATGGGAT	CTTTAGGCGC
CWL029	ATCGATGAGA	AGCTTTTTAA	AAGGTACCGC	GGCATGGGAT	CTTTAGGCGC
TW183	ATCGATGAGA	AGCTTTTTAA	AAGGTACCGC	GGCATGGGAT	CTTTAGGCGC
IOL207	ATCGATGAGA	AGCTTTTTAA	AAGGTACCGC	GGCATGGGAT	CTTTAGGCGC
TOR1	ATCGATGAGA	AGCTTTTTAA	AAGGTACCGC	GGCATGGGAT	CTTTAGGCGC
WA97001	ATCGATGAGA	AGCTTTTTAA	AAGGTACCGC	GGCATGGGAT	CTTTAGGCGC
1979	ATCGATGAGA	GGCTTTTTAA	AAGGTACCGC	GGCATGGGAT	CTTTAGGCGC
SH511	ATCGATGAGA	GGCTTTTTAA	AAGGTACCGC	GGCATGGGAT	CTTTAGGCGC

```
                410        420        430        440        450
Identity
DE177    TATGAAACAA GGAAGTGCTG ACCGGTATTT TCAAACACAG GGACAGAAAA
N16      TATGAAACAA GGAAGTGCTG ACCGGTATTT TCAAACACAG GGACAGAAAA
AR39     TATGAAACAA GGAAGTGCTG ACCGGTATTT TCAAACACAG GGACAGAAAA
CWL029   TATGAAACAA GGAAGTGCTG ACCGGTATTT TCAAACACAG GGACAGAAAA
TW183    TATGAAACAA GGAAGTGCTG ACCGGTATTT TCAAACACAG GGACAGAAAA
IOL207   TATGAAACAA GGAAGTGCTG ACCGGTATTT TCAAACACAG GGACAGAAAA
TOR1     TATGAAACAA GGAAGTGCTG ACCGGTATTT TCAAACACAG GGACAGAAAA
WA97001  TATGAAACAA GGAAGTGCTG ACCGGTATTT TCAAACACAG GGACAGAAAA
1979     TATGAAACAA GGAAGTGCTG ACCGGTATTT TCAAACACAG GGACAGAAAA
SH511    TATGAAACAA GGAAGTGCTG ACCGGTATTT TCAAACACAG GGACAGAAAA

                460        470        480        490        500
Identity
DE177    AGCTGGTTCC TGAGGGAGTT GAAGGACTAG TCGCTTATAA AGGCTCTGTC
N16      AGCTGGTTCC TGAGGGAGTT GAAGGACTAG TCACTTATAA AGGCTCTGTC
AR39     AGCTGGTTCC TGGGGGAGTT GAAGGACTAG TCGCTTATAA AGGCTCTGTC
CWL029   AGCTGGTTCC TGGGGGAGTT GAAGGACTAG TCGCTTATAA AGGCTCTGTC
TW183    AGCTGGTTCC TGGGGGAGTT GAAGGACTAG TCGCTTATAA AGGCTCTGTC
IOL207   AGCTGGTTCC TGGGGGAGTT GAAGGACTAG TCGCTTATAA AGGCTCTGTC
TOR1     AGCTGGTTCC TGGGGGAGTT GAAGGACTAG TCGCTTATAA AGGCTCTGTC
WA97001  AGCTGGTTCC TGGGGGAGTT GAAGGACTAG TCGCTTATAA AGGCTCTGTC
1979     AGCTGGTTCC TGAGGGAGTT GAAGGACTAG TCGCTTATAA AGGCTCTGTC
SH511    AGCTGGTTCC TGAGGGAGTT GAAGGACTAG TCGCTTATAA AGGCTCTGTC

                510        520        530        540        550
Identity
DE177    CACGATGTCC TCTATCAAAT TTTAGGAGGA ATACGCTCAG GTATGGGGTA
N16      CACGATGTCC TCTATCAAAT TTTAGGAGGA ATACGCTCAG GTATGGGGTA
AR39     CACGATGTCC TCTATCAAAT TTTAGGAGGA ATACGCTCAG GTATGGGGTA
CWL029   CACGATGTCC TCTATCAAAT TTTAGGAGGA ATACGCTCAG GTATGGGGTA
TW183    CACGATGTCC TCTATCAAAT TTTAGGAGGA ATACGCTCAG GTATGGGGTA
IOL207   CACGATGTCC TCTATCAAAT TTTAGGAGGA ATACGCTCAG GTATGGGGTA
TOR1     CACGATGTCC TCTATCAAAT TTTAGGAGGA ATACGCTCAG GTATGGGGTA
WA97001  CACGATGTCC TCTATCAAAT TTTAGGAGGA ATACGCTCAG GTATGGGGTA
1979     CACGATGTCC TCTATCAAAT TTTAGGAGGA ATACGCTCAG GTATGGGGTA
SH511    CACGATGTCC TCTATCAAAT TTTAGGAGGA ATACGCTCAG GTATGGGGTA

                560        570        580        590
Identity
DE177    TGTTGGAGCT GAAACTCTCA AAGATTTAAA AACTAAGGCT TCCTTTGTTC
N16      TGTTGGAGCT GAAACTCTCA AAGATTTAAA AACTAAGGCT TCCTTTGTTC
AR39     TGTTGGAGCT GAAACTCTCA AAGATTTAAA AACTAAGGCT TCCTTTGTTC
CWL029   TGTTGGAGCT GAAACTCTCA AAGATTTAAA AACTAAGGCT TCCTTTGTTC
TW183    TGTTGGAGCT GAAACTCTCA AAGATTTAAA AACTAAGGCT TCCTTTGTTC
IOL207   TGTTGGAGCT GAAACTCTCA AAGATTTAAA AACTAAGGCT TCCTTTGTTC
TOR1     TGTTGGAGCT GAAACTCTCA AAGATTTAAA AACTAAGGCT TCCTTTGTTC
WA97001  TGTTGGAGCT GAAACTCTCA AAGATTTAAA AACTAAGGCT TCCTTTGTTC
1979     TGTTGGAGCT GAAACTCTCA AAGATTTAAA AACTAAGGCT TCCTTTGTTC
SH511    TGTTGGAGCT GAAACTCTCA AAGATTTAAA AACTAAGGCT TCCTTTGTTC

             606
Identity
DE177    GAATTA
N16      GAATTA
AR39     GAATTA
CWL029   GAATTA
TW183    GAATTA
IOL207   GAATTA
TOR1     GAATTA
WA97001  GAATTA
1979     GAATTA
SH511    GAATTA
```

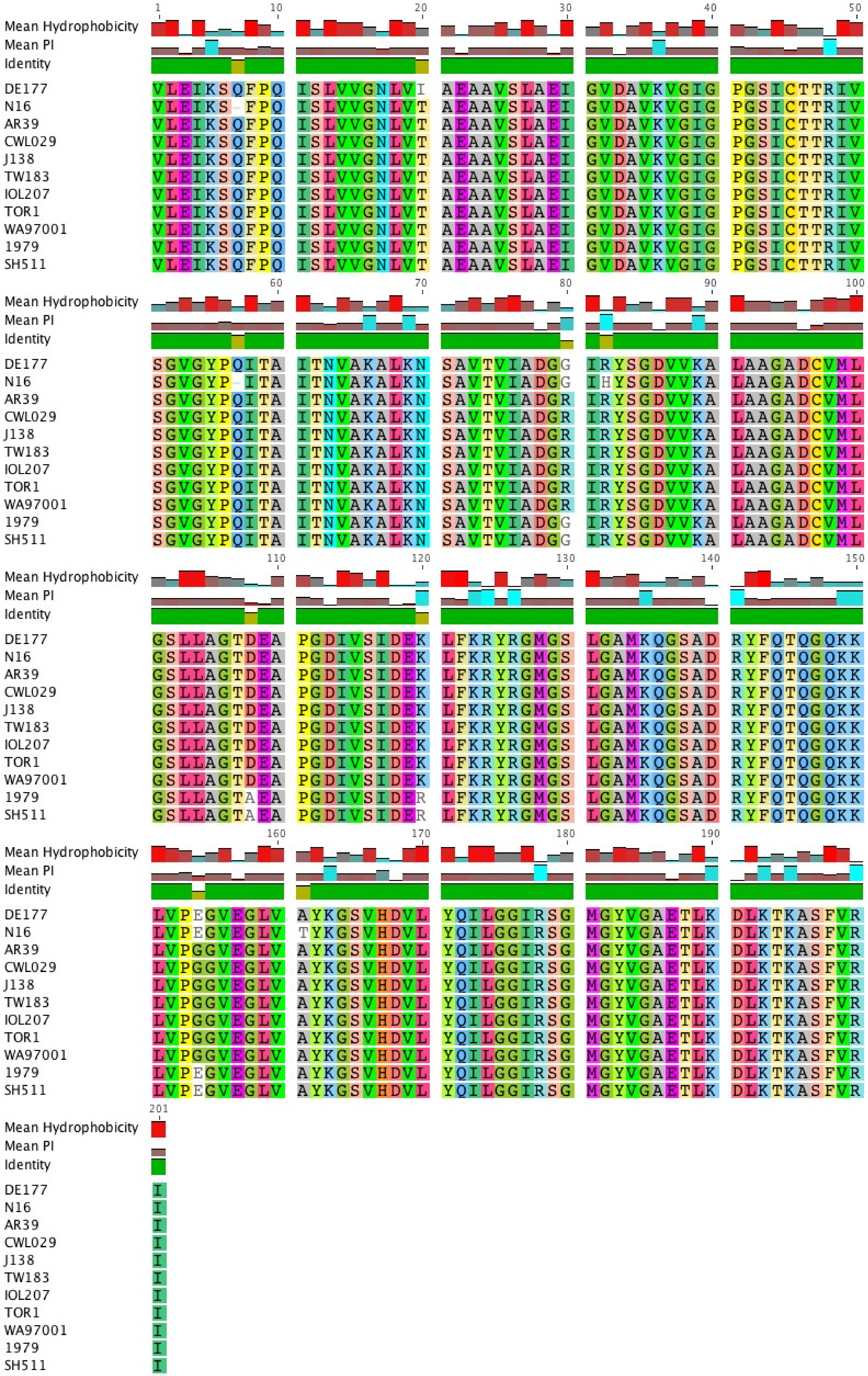

Mean Hydrophobicity
Mean PI
Identity
1 10 20 30 40 50 60 70 80 90 100 110 120 130 140 150 160 170 180 190 201
DE177 VLEIKSQFPQ ISLVVGNLVI AEAAVSLAEI GVDAVKVGIG PGSICTTRIV SGVGYPQITA ITNVAKALKN SAVTVIADGG IRYSGDVVKA LAAGADCVML GSLLAGTDEA PGDIVSIDEK LFKRYRGMGS LGAMKQGSAD RYFQTQGQKK LVPEGVEGLV AYKGSVHDVL YQILGGIRSG MGYVGAETLK DLKTKASFVR I
N16 VLEIKS-FPQ ISLVVGNLVT AEAAVSLAEI GVDAVKVGIG PGSICTTRIV SGVGYP-ITA ITNVAKALKN SAVTVIADGG IHYSGDVVKA LAAGADCVML GSLLAGTDEA PGDIVSIDEK LFKRYRGMGS LGAMKQGSAD RYFQTQGQKK LVPEGVEGLV TYKGSVHDVL YQILGGIRSG MGYVGAETLK DLKTKASFVR I
AR39 VLEIKSQFPQ ISLVVGNLVT AEAAVSLAEI GVDAVKVGIG PGSICTTRIV SGVGYPQITA ITNVAKALKN SAVTVIADGR IRYSGDVVKA LAAGADCVML GSLLAGTDEA PGDIVSIDEK LFKRYRGMGS LGAMKQGSAD RYFQTQGQKK LVPGGVEGLV AYKGSVHDVL YQILGGIRSG MGYVGAETLK DLKTKASFVR I
CWL029 VLEIKSQFPQ ISLVVGNLVT AEAAVSLAEI GVDAVKVGIG PGSICTTRIV SGVGYPQITA ITNVAKALKN SAVTVIADGR IRYSGDVVKA LAAGADCVML GSLLAGTDEA PGDIVSIDEK LFKRYRGMGS LGAMKQGSAD RYFQTQGQKK LVPGGVEGLV AYKGSVHDVL YQILGGIRSG MGYVGAETLK DLKTKASFVR I
J138 VLEIKSQFPQ ISLVVGNLVT AEAAVSLAEI GVDAVKVGIG PGSICTTRIV SGVGYPQITA ITNVAKALKN SAVTVIADGR IRYSGDVVKA LAAGADCVML GSLLAGTDEA PGDIVSIDEK LFKRYRGMGS LGAMKQGSAD RYFQTQGQKK LVPGGVEGLV AYKGSVHDVL YQILGGIRSG MGYVGAETLK DLKTKASFVR I
TW183 VLEIKSQFPQ ISLVVGNLVT AEAAVSLAEI GVDAVKVGIG PGSICTTRIV SGVGYPQITA ITNVAKALKN SAVTVIADGR IRYSGDVVKA LAAGADCVML GSLLAGTDEA PGDIVSIDEK LFKRYRGMGS LGAMKQGSAD RYFQTQGQKK LVPGGVEGLV AYKGSVHDVL YQILGGIRSG MGYVGAETLK DLKTKASFVR I
IOL207 VLEIKSQFPQ ISLVVGNLVT AEAAVSLAEI GVDAVKVGIG PGSICTTRIV SGVGYPQITA ITNVAKALKN SAVTVIADGR IRYSGDVVKA LAAGADCVML GSLLAGTDEA PGDIVSIDEK LFKRYRGMGS LGAMKQGSAD RYFQTQGQKK LVPGGVEGLV AYKGSVHDVL YQILGGIRSG MGYVGAETLK DLKTKASFVR I
TOR1 VLEIKSQFPQ ISLVVGNLVT AEAAVSLAEI GVDAVKVGIG PGSICTTRIV SGVGYPQITA ITNVAKALKN SAVTVIADGR IRYSGDVVKA LAAGADCVML GSLLAGTDEA PGDIVSIDEK LFKRYRGMGS LGAMKQGSAD RYFQTQGQKK LVPGGVEGLV AYKGSVHDVL YQILGGIRSG MGYVGAETLK DLKTKASFVR I
WA97001 VLEIKSQFPQ ISLVVGNLVT AEAAVSLAEI GVDAVKVGIG PGSICTTRIV SGVGYPQITA ITNVAKALKN SAVTVIADGR IRYSGDVVKA LAAGADCVML GSLLAGTDEA PGDIVSIDEK LFKRYRGMGS LGAMKQGSAD RYFQTQGQKK LVPGGVEGLV AYKGSVHDVL YQILGGIRSG MGYVGAETLK DLKTKASFVR I
1979 VLEIKSQFPQ ISLVVGNLVT AEAAVSLAEI GVDAVKVGIG PGSICTTRIV SGVGYPQITA ITNVAKALKN SAVTVIADGG IRYSGDVVKA LAAGADCVML GSLLAGTAEA PGDIVSIDER LFKRYRGMGS LGAMKQGSAD RYFQTQGQKK LVPEGVEGLV AYKGSVHDVL YQILGGIRSG MGYVGAETLK DLKTKASFVR I
SH511 VLEIKSQFPQ ISLVVGNLVT AEAAVSLAEI GVDAVKVGIG PGSICTTRIV SGVGYPQITA ITNVAKALKN SAVTVIADGG IRYSGDVVKA LAAGADCVML GSLLAGTAEA PGDIVSIDER LFKRYRGMGS LGAMKQGSAD RYFQTQGQKK LVPEGVEGLV AYKGSVHDVL YQILGGIRSG MGYVGAETLK DLKTKASFVR I
