# Supplementary material for: Chlamydia pneumoniae Is Genetically Diverse in Animals and Appears to Have Crossed the Host Barrier to Humans on (At Least) Two Occasions
Source: PLoS Pathog. 2010 May 20;6(5):e1000903. doi: 10.1371/journal.ppat.1000903 (PMC2873915; doi:10.1371/journal.ppat.1000903)
Supplement: Figure S18 — Multiple sequence alignment of guaA . The nucleotide and amino acid alignments were generated using Geneious version 4.7, where each nucleotide and amino acid is assigned its own colour. White shading indicates an amino acid variant. (1.08 MB PDF) [file ppat.1000903.s018.pdf]

	1 … 10	20	30	40	50
Identity					
DE177	ATATACTTAT	GTATTAGCAA	AGCAAGTGCG	GAAGTTATTT	GTATATTGCG
AR39	ATATACTTAT	GTATTAGCAA	AGCAAGTGCG	GAAGTTATTT	GTATATTGCG
CWL029	ATATACTTAT	GTATTAGCAA	AGCAAGTGCG	GAAGTTATTT	GTATATTGCG
J138	ATATACTTAT	GTATTAGCAA	AGCAAGTGCG	GAAGTTATTT	GTATATTGCG
TW183	ATATACTTAT	GTATTAGCAA	AGCAAGTGCG	GAAGTTATTT	GTATATTGCG
TOR1	ATATACTTAT	GTATTAGCAA	AGCAAGTGCG	GAAGTTATTT	GTATATTGCG
WA97001	ATATACTTAT	GTATTAGCAA	AGCAAGTGCG	GAAGTTATTT	GTATATTGCG

	60	70	80	90	100
Identity					
DE177	AAGTTCTTCC	CTGGAATATC	TCTGTGCAAT	GTTTAAAAGA	AAGAGCGCCT
AR39	AAGTTCTTCC	CTGGAATATC	TCTGTGCAAT	GTTTAAAAGA	AAGAGCGCCT
CWL029	AAGTTCTTCC	CTGGAATATC	TCTGTGCAAT	GTTTAAAAGA	AAGAGCGCCT
J138	AAGTTCTTCC	CTGGAATATC	TCTGTGCAAT	GTTTAAAAGA	AAGAGCGCCT
TW183	AAGTTCTTCC	CTGGAATATC	TCTGTGCAAT	GTTTAAAAGA	AAGAGCGCCT
TOR1	AAGTTCTTCC	CTGGAATATC	TCTGTGCAAT	GTTTAAAAGA	AAGAGCGCCT
WA97001	AAGTTCTTCC	CTGGAATATC	TCTGTGCAAT	GTTTAAAAGA	AAGAGCGCCT

	110	120	130	140	150
Identity					
DE177	TTGGGGATCA	TTCTCTCAGG	AGGTCCTCAC	TCTGTCTATG	AAAACAAGGC
AR39	TTGGGGATCA	TTCTCTCAGG	AGGTCCTCAC	TCTGTCTATG	AAAACAAGGC
CWL029	TTGGGGATCA	TTCTCTCAGG	AGGTCCTCAC	TCTGTCTATG	AAAACAAGGC
J138	TTGGGGATCA	TTCTCTCAGG	AGGTCCTCAC	TCTGTCTATG	AAAACAAGGC
TW183	TTGGGGATCA	TTCTCTCAGG	AGGTCCTCAC	TCTGTCTATG	AAAACAAGGC
TOR1	TTGGGGATCA	TTCTCTCAGG	AGGTCCTCAC	TCTGTCTATG	AAAACAAGGC
WA97001	TTGGGGATCA	TTCTCTCAGG	AGGTCCTCAC	TCTGTCTATG	AAAACAAGGC

	160	170	180	190	200
Identity					
DE177	TCCACATTTA	GATCCTGAAA	TCTATAAACT	TGGCATTCCA	ATTCTAGCTA
AR39	TCCACATTTA	GATCCTGAAA	TCTATAAACT	TGGCATTCCA	ATTCTAGCTA
CWL029	TCCACATTTA	GATCCTGAAA	TCTATAAACT	TGGCATTCCA	ATTCTAGCTA
J138	TCCACATTTA	GATCCTGAAA	TCTATAAACT	TGGCATTCCA	ATTCTAGCTA
TW183	TCCACATTTA	GATCCTGAAA	TCTATAAACT	TGGCATTCCA	ATTCTAGCTA
TOR1	TCCACATTTA	GATCCTGAAA	TCTATAAACT	TGGCATTCCA	ATTCTAGCTA
WA97001	TCCACATTTA	GATCCTGAAA	TCTATAAACT	TGGCATTCCA	ATTCTAGCTA

	210	220	230	240	250
Identity					
DE177	TTTGCTATGG	CATGCAGCTT	ATGGCTAGAG	ATTTTGGAGG	GACTGTAAGC
AR39	TTTGCTATGG	CATGCAGCTT	ATGGCTAGAG	ATTTTGGAGG	GACTGTAAGC
CWL029	TTTGCTATGG	CATGCAGCTT	ATGGCTAGAG	ATTTTGGAGG	GACTGTAAGC
J138	TTTGCTATGG	CATGCAGCTT	ATGGCTAGAG	ATTTTGGAGG	GACTGTAAGC
TW183	TTTGCTATGG	CATGCAGCTT	ATGGCTAGAG	ATTTTGGAGG	GACTGTAAGC
TOR1	TTTGCTATGG	CATGCAGCTT	ATGGCTAGAG	ATTTTGGAGG	GACTGTAAGC
WA97001	TTTGCTATGG	CATGCAGCTT	ATGGCTAGAG	ATTTTGGAGG	GACTGTAAGC

	260	270	280	290	300
Identity					
DE177	CCTGGTGTAG	GAGAATTTGG	ATATACGCCC	ATCCATCTGT	ATCCTTGTGA
AR39	CCTGGTGTAG	GAGAATTTGG	ATATACGCCC	ATCCATCTGT	ATCCTTGTGA
CWL029	CCTGGTGTAG	GAGAATTTGG	ATATACGCCC	ATCCATCTGT	ATCCTTGTGA
J138	CCTGGTGTAG	GAGAATTTGG	ATATACGCCC	ATCCATCTGT	ATCCTTGTGA
TW183	CCTGGTGTAG	GAGAATTTGG	ATATACGCCC	ATCCATCTGT	ATCCTTGTGA
TOR1	CCTGGTGTAG	GAGAATTTGG	ATATACGCCC	ATCCATCTGT	ATCCTTGTGA
WA97001	CCTGGTGTAG	GAGAATTTGG	ATATACGCCC	ATCCATCTGT	ATCCTTGTGA

	310	320	330	340	350
Identity					
DE177	GCTCTTCAAA	CACATCGTCG	ACTGCGAATC	TCTAGACACA	GAGATTCGGA
AR39	GCTCTTCAAA	CACATCGTCG	ACTGCGAATC	TCTAGACACA	GAGATTCGGA
CWL029	GCTCTTCAAA	CACATCGTCG	ACTGCGAATC	TCTAGACACA	GAGATTCGGA
J138	GCTCTTCAAA	CACATCGTCG	ACTGCGAATC	TCTAGACACA	GAGATTCGGA
TW183	GCTCTTCAAA	CACATCGTCG	ACTGCGAATC	TCTAGACACA	GAGATTCGGA
TOR1	GCTCTTCAAA	CACATCGTCG	ACTGCGAATC	TCTAGACACA	GAGATTCGGA
WA97001	GCTCTTCAAA	CACATCGTCG	ACTGCGAATC	TCTAGACACA	GAGATTCGGA

	360	370	380	390	400
Identity					
DE177	TGAGCCATCG	GGATCATGTT	ACGACAATTC	CTGAAGGATT	TAATGTAATC
AR39	TGAGCCATCG	GGATCATGTT	ACGACAATTC	CTGAAGGATT	TAATGTAATC
CWL029	TGAGCCATCG	GGATCATGTT	ACGACAATTC	CTGAAGGATT	TAATGTAATC
J138	TGAGCCATCG	GGATCATGTT	ACGACAATTC	CTGAAGGATT	TAATGTAATC
TW183	TGAGCCATCG	GGATCATGTT	ACGACAATTC	CTGAAGGATT	TAATGTAATC
TOR1	TGAGCCATCG	GGATCATGTT	ACGACAATTC	CTGAAGGATT	TAATGTAATC
WA97001	TGAGCCATCG	GGATCATGTT	ACGACAATTC	CTGAAGGATT	TAATGTAATC

	410	420	430	440	450
Identity					
DE177	GCATCCACCT	CACAATGCTC	GATCTCAGGA	ATAGAAAATA	CCAAACAACG
AR39	GCATCCACCT	CACAATGCTC	GATCTCAGGA	ATAGAAAATA	CCAAACAACG
CWL029	GCATCCACCT	CACAATGCTC	GATCTCAGGA	ATAGAAAATA	CCAAACAACG
J138	GCATCCACCT	CACAATGCTC	GATCTCAGGA	ATAGAAAATA	CCAAACAACG
TW183	GCATCCACCT	CACAATGCTC	GATCTCAGGA	ATAGAAAATA	CCAAACAACG
TOR1	GCATCCACCT	CACAATGCTC	GATCTCAGGA	ATAGAAAATA	CCAAACAACG
WA97001	GCATCCACCT	CACAATGCTC	GATCTCAGGA	ATAGAAAATA	CCAAACAACG

	460	470	480	490	500
Identity					
DE177	GTTGTACGGG	CTGCAATTTC	ATCCCGAGGT	TTCTGACTCC	ACTCCAACGG
AR39	GTTGTACGGG	CTGCAATTTC	ATCCCGAGGT	TTCTGACTCC	ACTCCAACGG
CWL029	GTTGTACGGG	CTGCAATTTC	ATCCCGAGGT	TTCTGACTCC	ACTCCAACGG
J138	GTTGTACGGG	CTGCAATTTC	ATCCCGAGGT	TTCTGACTCC	ACTCCAACGG
TW183	GTTGTACGGG	CTGCAATTTC	ATCCCGAGGT	TTCTGACTCC	ACTCCAACGG
TOR1	GTTGTACGGG	CTGCAATTTC	ATCCCGAGGT	TTCTGACTCC	ACTCCAACGG
WA97001	GTTGTACGGG	CTGCAATTTC	ATCCCGAGGT	TTCTGACTCC	ACTCCAACGG

	510	520	530	540	550
Identity					
DE177	GAAATAAGAT	TCTAGAAACT	TTTGTTCAAG	AGATCTGTTC	TGCTCCCACA
AR39	GAAATAAGAT	TCTAGAAACT	TTTGTTCAAG	AGATCTGTTC	TGCTCCCACA
CWL029	GAAATAAGAT	TCTAGAAACT	TTTGTTCAAG	AGATCTGTTC	TGCTCCCACA
J138	GAAATAAGAT	TCTAGAAACT	TTTGTTCAAG	AGATCTGTTC	TGCTCCCACA
TW183	GAAATAAGAT	TCTAGAAACT	TTTGTTCAAG	AGATCTGTTC	TGCTCCCACA
TOR1	GAAATAAGAT	TCTAGAAACT	TTTGTTCAAG	AGATCTGTTC	TGCTCCCACA
WA97001	GAAATAAGAT	TCTAGAAACT	TTTGTTCAAG	AGATCTGTTC	TGCTCCCACA

	560	570	580	590	600
Identity					
DE177	CTATGGAATC	CCTTGTATAT	TCAGCAAGAC	CTTGTAAGTA	AAATTCAAGA
AR39	CTATGGAATC	CCTTGTATAT	TCAGCAAGAC	CTTGTAAGTA	AAATTCAAGA
CWL029	CTATGGAATC	CCTTGTATAT	TCAGCAAGAC	CTTGTAAGTA	AAATTCAAGA
J138	CTATGGAATC	CCTTGTATAT	TCAGCAAGAC	CTTGTAAGTA	AAATTCAAGA
TW183	CTATGGAATC	CCTTGTATAT	TCAGCAAGAC	CTTGTAAGTA	AAATTCAAGA
TOR1	CTATGGAATC	CCTTGTATAT	TCAGCAAGAC	CTTGTAAGTA	AAATTCAAGA
WA97001	CTATGGAATC	CCTTGTATAT	TCAGCAAGAC	CTTGTAAGTA	AAATTCAAGA

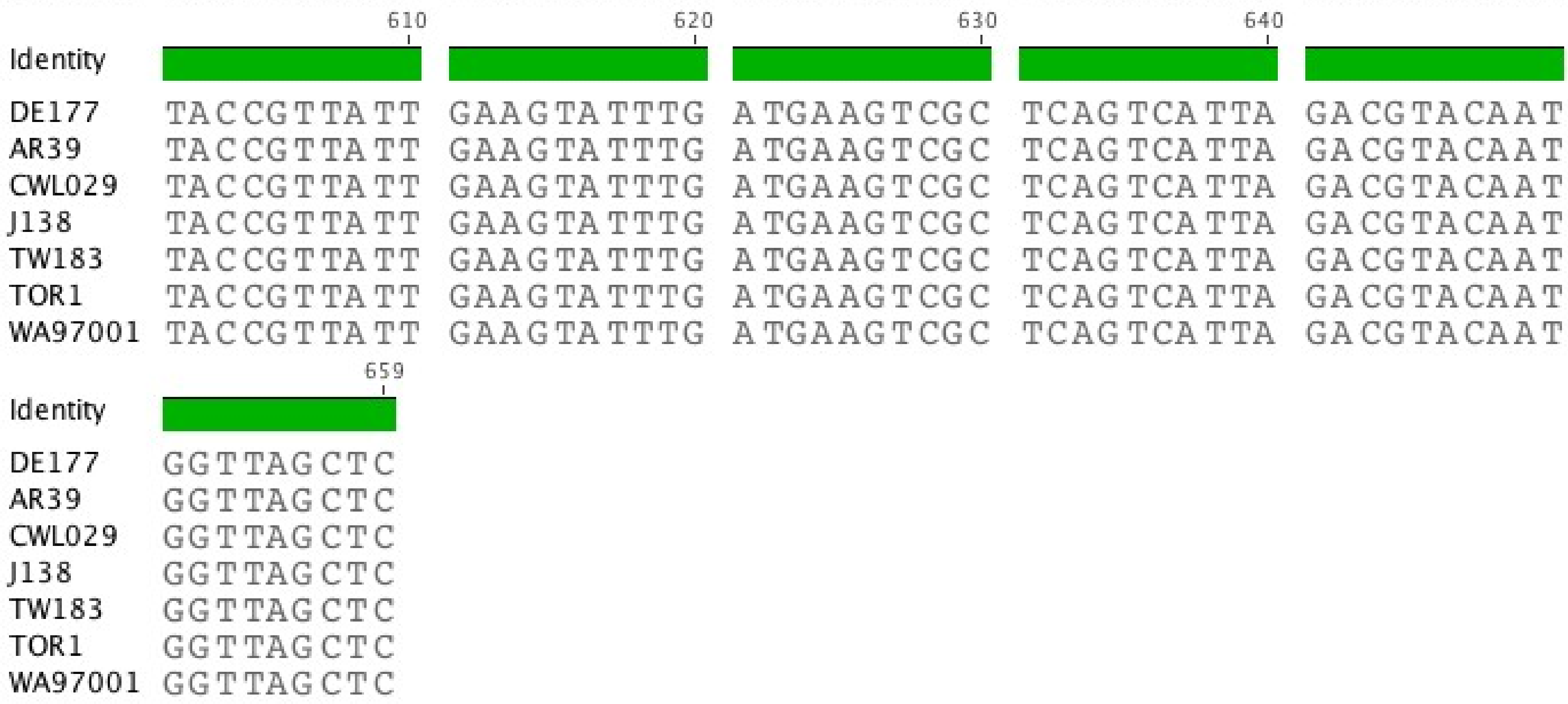

610
620
630
640
Identity
DE177 TACCGTTATT GAAGTATTTG ATGAAGTCGC TCAGTCATTA GACGTACAAT
AR39 TACCGTTATT GAAGTATTTG ATGAAGTCGC TCAGTCATTA GACGTACAAT
CWL029 TACCGTTATT GAAGTATTTG ATGAAGTCGC TCAGTCATTA GACGTACAAT
J138 TACCGTTATT GAAGTATTTG ATGAAGTCGC TCAGTCATTA GACGTACAAT
TW183 TACCGTTATT GAAGTATTTG ATGAAGTCGC TCAGTCATTA GACGTACAAT
TOR1 TACCGTTATT GAAGTATTTG ATGAAGTCGC TCAGTCATTA GACGTACAAT
WA97001 TACCGTTATT GAAGTATTTG ATGAAGTCGC TCAGTCATTA GACGTACAAT
659
Identity
DE177 GGTTAGCTC
AR39 GGTTAGCTC
CWL029 GGTTAGCTC
J138 GGTTAGCTC
TW183 GGTTAGCTC
TOR1 GGTTAGCTC
WA97001 GGTTAGCTC

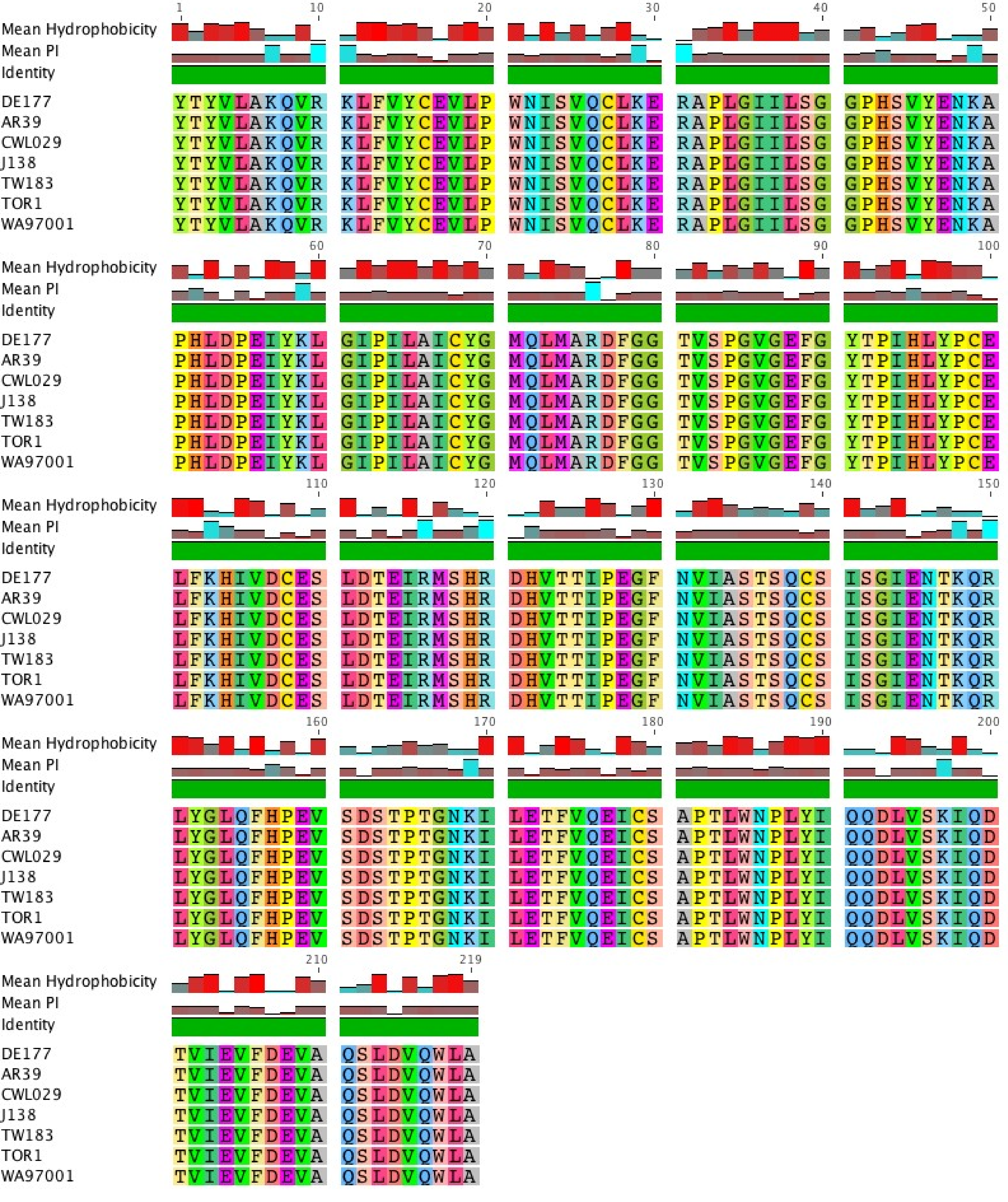
1 10 20 30 40 50
Mean Hydrophobicity
Mean PI
Identity
DE177 YTYVLAKQVR KLFVYCEVLP WNISVQCLKE RAPLGIILSG GPHSVYENKA
AR39 YTYVLAKQVR KLFVYCEVLP WNISVQCLKE RAPLGIILSG GPHSVYENKA
CWL029 YTYVLAKQVR KLFVYCEVLP WNISVQCLKE RAPLGIILSG GPHSVYENKA
J138 YTYVLAKQVR KLFVYCEVLP WNISVQCLKE RAPLGIILSG GPHSVYENKA
TW183 YTYVLAKQVR KLFVYCEVLP WNISVQCLKE RAPLGIILSG GPHSVYENKA
TOR1 YTYVLAKQVR KLFVYCEVLP WNISVQCLKE RAPLGIILSG GPHSVYENKA
WA97001 YTYVLAKQVR KLFVYCEVLP WNISVQCLKE RAPLGIILSG GPHSVYENKA
60 70 80 90 100
Mean Hydrophobicity
Mean PI
Identity
DE177 PHLDPEIYKL GIPILAICYG MQLMARDFGG TVSPGVGEFG YTPIHLYPCE
AR39 PHLDPEIYKL GIPILAICYG MQLMARDFGG TVSPGVGEFG YTPIHLYPCE
CWL029 PHLDPEIYKL GIPILAICYG MQLMARDFGG TVSPGVGEFG YTPIHLYPCE
J138 PHLDPEIYKL GIPILAICYG MQLMARDFGG TVSPGVGEFG YTPIHLYPCE
TW183 PHLDPEIYKL GIPILAICYG MQLMARDFGG TVSPGVGEFG YTPIHLYPCE
TOR1 PHLDPEIYKL GIPILAICYG MQLMARDFGG TVSPGVGEFG YTPIHLYPCE
WA97001 PHLDPEIYKL GIPILAICYG MQLMARDFGG TVSPGVGEFG YTPIHLYPCE
110 120 130 140 150
Mean Hydrophobicity
Mean PI
Identity
DE177 LFKHIVDCES LDTEIRMSHR DHVTTIPEGF NVIASTSQCS ISGIENTKQR
AR39 LFKHIVDCES LDTEIRMSHR DHVTTIPEGF NVIASTSQCS ISGIENTKQR
CWL029 LFKHIVDCES LDTEIRMSHR DHVTTIPEGF NVIASTSQCS ISGIENTKQR
J138 LFKHIVDCES LDTEIRMSHR DHVTTIPEGF NVIASTSQCS ISGIENTKQR
TW183 LFKHIVDCES LDTEIRMSHR DHVTTIPEGF NVIASTSQCS ISGIENTKQR
TOR1 LFKHIVDCES LDTEIRMSHR DHVTTIPEGF NVIASTSQCS ISGIENTKQR
WA97001 LFKHIVDCES LDTEIRMSHR DHVTTIPEGF NVIASTSQCS ISGIENTKQR
160 170 180 190 200
Mean Hydrophobicity
Mean PI
Identity
DE177 LYGLQFHPEV SDSTPTGNKI LETFVQEICS APTLWNPLYI QQDLVSKIQD
AR39 LYGLQFHPEV SDSTPTGNKI LETFVQEICS APTLWNPLYI QQDLVSKIQD
CWL029 LYGLQFHPEV SDSTPTGNKI LETFVQEICS APTLWNPLYI QQDLVSKIQD
J138 LYGLQFHPEV SDSTPTGNKI LETFVQEICS APTLWNPLYI QQDLVSKIQD
TW183 LYGLQFHPEV SDSTPTGNKI LETFVQEICS APTLWNPLYI QQDLVSKIQD
TOR1 LYGLQFHPEV SDSTPTGNKI LETFVQEICS APTLWNPLYI QQDLVSKIQD
WA97001 LYGLQFHPEV SDSTPTGNKI LETFVQEICS APTLWNPLYI QQDLVSKIQD
210 219
Mean Hydrophobicity
Mean PI
Identity
DE177 TVIEVFDEVA QSLDVQWLA
AR39 TVIEVFDEVA QSLDVQWLA
CWL029 TVIEVFDEVA QSLDVQWLA
J138 TVIEVFDEVA QSLDVQWLA
TW183 TVIEVFDEVA QSLDVQWLA
TOR1 TVIEVFDEVA QSLDVQWLA
WA97001 TVIEVFDEVA QSLDVQWLA
