# Supplementary material for: Chlamydia pneumoniae Is Genetically Diverse in Animals and Appears to Have Crossed the Host Barrier to Humans on (At Least) Two Occasions
Source: PLoS Pathog. 2010 May 20;6(5):e1000903. doi: 10.1371/journal.ppat.1000903 (PMC2873915; doi:10.1371/journal.ppat.1000903)
Supplement: Figure S19 — Multiple sequence alignment of add . The nucleotide and amino acid alignments were generated using Geneious version 4.7, where each nucleotide and amino acid is assigned its own colour. White shading indicates an amino acid variant. (2.87 MB PDF) [file ppat.1000903.s019.pdf]

	1–10	20	30	40	50	60
Identity						
AR39	TGAGATCTTT	GCATTAGTAC	CACGACTCAA	TACAATCGCT	TGCACCGAAG	CTATCATCAA
CWL029	TGAGATCTTT	GCATTAGTAC	CACGACTCAA	TACAATCGCT	TGCACCGAAG	CTATCATCAA
J138	TGAGATCTTT	GCATTAGTAC	CACGACTCAA	TACAATCGCT	TGCACCGAAG	CTATCATCAA
TW183	TGAGATCTTT	GCATTAGTAC	CACGACTCAA	TACAATCGCT	TGCACCGAAG	CTATCATCAA
TOR1	TGAGATCTTT	GCATTAGTAC	CACGACTCAA	TACAATCGCT	TGCACCGAAG	CTATCATCAA
WA97001	TGAGATCTTT	GCATTAGTAC	CACGACTCAA	TACAATCGCT	TGCACCGAAG	CTATCATCAA

	70	80	90	100	110	120
Identity						
AR39	AAACCTCCCC	AAAGCAGATA	TCCATGTACA	CCTTCCTGGG	ACCATAACAC	CTCAATTAGC
CWL029	AAACCTCCCC	AAAGCAGATA	TCCATGTACA	CCTTCCTGGG	ACCATAACAC	CTCAATTAGC
J138	AAACCTCCCC	AAAGCAGATA	TCCATGTACA	CCTTCCTGGG	ACCATAACAC	CTCAATTAGC
TW183	AAACCTCCCC	AAAGCAGATA	TCCATGTACA	CCTTCCTGGG	ACCATAACAC	CTCAATTAGC
TOR1	AAACCTCCCC	AAAGCAGATA	TCCATGTACA	CCTTCCTGGG	ACCATAACAC	CTCAATTAGC
WA97001	AAACCTCCCC	AAAGCAGATA	TCCATGTACA	CCTTCCTGGG	ACCATAACAC	CTCAATTAGC

	130	140	150	160	170	180
Identity						
AR39	TTGGATTTTA	GGTGTGAAAA	ATGGGTTCTT	AAAATGGTCT	TATAATTCTT	GGACCAATCA
CWL029	TTGGATTTTA	GGTGTGAAAA	ATGGGTTCTT	AAAATGGTCT	TATAATTCTT	GGACCAATCA
J138	TTGGATTTTA	GGTGTGAAAA	ATGGGTTCTT	AAAATGGTCT	TATAATTCTT	GGACCAATCA
TW183	TTGGATTTTA	GGTGTGAAAA	ATGGGTTCTT	AAAATGGTCT	TATAATTCTT	GGACCAATCA
TOR1	TTGGATTTTA	GGTGTGAAAA	ATGGGTTCTT	AAAATGGTCT	TATAATTCTT	GGACCAATCA
WA97001	TTGGATTTTA	GGTGTGAAAA	ATGGGTTCTT	AAAATGGTCT	TATAATTCTT	GGACCAATCA

	190	200	210	220	230	240
Identity						
AR39	TCGATTACTT	TCTCCTAAGA	ATCCTCATAA	ACAATACTCC	AATATTTTCC	GAAACTTTCA
CWL029	TCGATTACTT	TCTCCTAAGA	ATCCTCATAA	ACAATACTCC	AATATTTTCC	GAAACTTTCA
J138	TCGATTACTT	TCTCCTAAGA	ATCCTCATAA	ACAATACTCC	AATATTTTCC	GAAACTTTCA
TW183	TCGATTACTT	TCTCCTAAGA	ATCCTCATAA	ACAATACTCC	AATATTTTCC	GAAACTTTCA
TOR1	TCGATTACTT	TCTCCTAAGA	ATCCTCATAA	ACAATACTCC	AATATTTTCC	GAAACTTTCA
WA97001	TCGATTACTT	TCTCCTAAGA	ATCCTCATAA	ACAATACTCC	AATATTTTCC	GAAACTTTCA

	250	260	270	280	290	300
Identity						
AR39	AGATATCTGT	CACGAAAAGG	ATCCGGATTT	AAGTGTATTA	CAATATAATA	TCTTAAATTA
CWL029	AGATATCTGT	CACGAAAAGG	ATCCGGATTT	AAGTGTATTA	CAATATAATA	TCTTAAATTA
J138	AGATATCTGT	CACGAAAAGG	ATCCGGATTT	AAGTGTATTA	CAATATAATA	TCTTAAATTA
TW183	AGATATCTGT	CACGAAAAGG	ATCCGGATTT	AAGTGTATTA	CAATATAATA	TCTTAAATTA
TOR1	AGATATCTGT	CACGAAAAGG	ATCCGGATTT	AAGTGTATTA	CAATATAATA	TCTTAAATTA
WA97001	AGATATCTGT	CACGAAAAGG	ATCCGGATTT	AAGTGTATTA	CAATATAATA	TCTTAAATTA

	310	320	330	340	350	360
Identity						
AR39	CGATTTTAAT	AGCTTTGATA	GAGTGATGGC	TACAGTACAA	GGACATCGCT	TTCCTCCTGG
CWL029	CGATTTTAAT	AGCTTTGATA	GAGTGATGGC	TACAGTACAA	GGACATCGCT	TTCCTCCTGG
J138	CGATTTTAAT	AGCTTTGATA	GAGTGATGGC	TACAGTACAA	GGACATCGCT	TTCCTCCTGG
TW183	CGATTTTAAT	AGCTTTGATA	GAGTGATGGC	TACAGTACAA	GGACATCGCT	TTCCTCCTGG
TOR1	CGATTTTAAT	AGCTTTGATA	GAGTGATGGC	TACAGTACAA	GGACATCGCT	TTCCTCCTGG
WA97001	CGATTTTAAT	AGCTTTGATA	GAGTGATGGC	TACAGTACAA	GGACATCGCT	TTCCTCCTGG

	370	380	390	400	410	420
Identity						
AR39	AGGAATCCAA	AATGAAGAAG	ACCTTCTTCT	CATTTTCAAT	AACTATCTCC	AGCAATGTCT
CWL029	AGGAATCCAA	AATGAAGAAG	ACCTTCTTCT	CATTTTCAAT	AACTATCTCC	AGCAATGTCT
J138	AGGAATCCAA	AATGAAGAAG	ACCTTCTTCT	CATTTTCAAT	AACTATCTCC	AGCAATGTCT
TW183	AGGAATCCAA	AATGAAGAAG	ACCTTCTTCT	CATTTTCAAT	AACTATCTCC	AGCAATGTCT
TOR1	AGGAATCCAA	AATGAAGAAG	ACCTTCTTCT	CATTTTCAAT	AACTATCTCC	AGCAATGTCT
WA97001	AGGAATCCAA	AATGAAGAAG	ACCTTCTTCT	CATTTTCAAT	AACTATCTCC	AGCAATGTCT

	430	440	450	460	470	480
Identity						
AR39	GGACGATACT	ATCGTGTATA	CTGAAGTACA	ACAAAATATC	CGCCTTGCCC	ATGTTTTGTA
CWL029	GGACGATACT	ATCGTGTATA	CTGAAGTACA	ACAAAATATC	CGCCTTGCCC	ATGTTTTGTA
J138	GGACGATACT	ATCGTGTATA	CTGAAGTACA	ACAAAATATC	CGCCTTGCCC	ATGTTTTGTA
TW183	GGACGATACT	ATCGTGTATA	CTGAAGTACA	ACAAAATATC	CGCCTTGCCC	ATGTTTTGTA
TOR1	GGACGATACT	ATCGTGTATA	CTGAAGTACA	ACAAAATATC	CGCCTTGCCC	ATGTTTTGTA
WA97001	GGACGATACT	ATCGTGTATA	CTGAAGTACA	ACAAAATATC	CGCCTTGCCC	ATGTTTTGTA

	490	500	510	520	530	540
Identity						
AR39	TCCTTCATTA	CCTGAAAAGC	ACGCGCGTAT	GAAGTTTTAT	CAAATCTTGT	ATCGTGCTTC
CWL029	TCCTTCATTA	CCTGAAAAGC	ACGCGCGTAT	GAAGTTTTAT	CAAATCTTGT	ATCGTGCTTC
J138	TCCTTCATTA	CCTGAAAAGC	ACGCGCGTAT	GAAGTTTTAT	CAAATCTTGT	ATCGTGCTTC
TW183	TCCTTCATTA	CCTGAAAAGC	ACGCGCGTAT	GAAGTTTTAT	CAAATCTTGT	ATCGTGCTTC
TOR1	TCCTTCATTA	CCTGAAAAGC	ACGCGCGTAT	GAAGTTTTAT	CAAATCTTGT	ATCGTGCTTC
WA97001	TCCTTCATTA	CCTGAAAAGC	ACGCGCGTAT	GAAGTTTTAT	CAAATCTTGT	ATCGTGCTTC

	550	560	570	580	590	600
Identity						
AR39	GCAAACGTTT	TCAAAACACG	GGATTACTTT	ACGATTTTTA	AACTGCTTCA	ATAAAACATT
CWL029	GCAAACGTTT	TCAAAACACG	GGATTACTTT	ACGATTTTTA	AACTGCTTCA	ATAAAACATT
J138	GCAAACGTTT	TCAAAACACG	GGATTACTTT	ACGATTTTTA	AACTGCTTCA	ATAAAACATT
TW183	GCAAACGTTT	TCAAAACACG	GGATTACTTT	ACGATTTTTA	AACTGCTTCA	ATAAAACATT
TOR1	GCAAACGTTT	TCAAAACACG	GGATTACTTT	ACGATTTTTA	AACTGCTTCA	ATAAAACATT
WA97001	GCAAACGTTT	TCAAAACACG	GGATTACTTT	ACGATTTTTA	AACTGCTTCA	ATAAAACATT

	610	620	630	640	650	660
Identity						
AR39	TGCTCCACAA	ATAAACACAC	AAGAACCTGC	CCAAGAAGCT	GTTCAATGGC	TCCAAGAGGT
CWL029	TGCTCCACAA	ATAAACACAC	AAGAACCTGC	CCAAGAAGCT	GTTCAATGGC	TCCAAGAGGT
J138	TGCTCCACAA	ATAAACACAC	AAGAACCTGC	CCAAGAAGCT	GTTCAATGGC	TCCAAGAGGT
TW183	TGCTCCACAA	ATAAACACAC	AAGAACCTGC	CCAAGAAGCT	GTTCAATGGC	TCCAAGAGGT
TOR1	TGCTCCACAA	ATAAACACAC	AAGAACCTGC	CCAAGAAGCT	GTTCAATGGC	TCCAAGAGGT
WA97001	TGCTCCACAA	ATAAACACAC	AAGAACCTGC	CCAAGAAGCT	GTTCAATGGC	TCCAAGAGGT

	670	680	690	700	710	720
Identity						
AR39	TGATTCTACA	TTTCCTGGTC	TATTTGTAGG	GATACAATCC	GCAGGATCAG	AATCTGCGCC
CWL029	TGATTCTACA	TTTCCTGGTC	TATTTGTAGG	GATACAATCC	GCAGGATCAG	AATCTGCGCC
J138	TGATTCTACA	TTTCCTGGTC	TATTTGTAGG	GATACAATCC	GCAGGATCAG	AATCTGCGCC
TW183	TGATTCTACA	TTTCCTGGTC	TATTTGTAGG	GATACAATCC	GCAGGATCAG	AATCTGCGCC
TOR1	TGATTCTACA	TTTCCTGGTC	TATTTGTAGG	GATACAATCC	GCAGGATCAG	AATCTGCGCC
WA97001	TGATTCTACA	TTTCCTGGTC	TATTTGTAGG	GATACAATCC	GCAGGATCAG	AATCTGCGCC

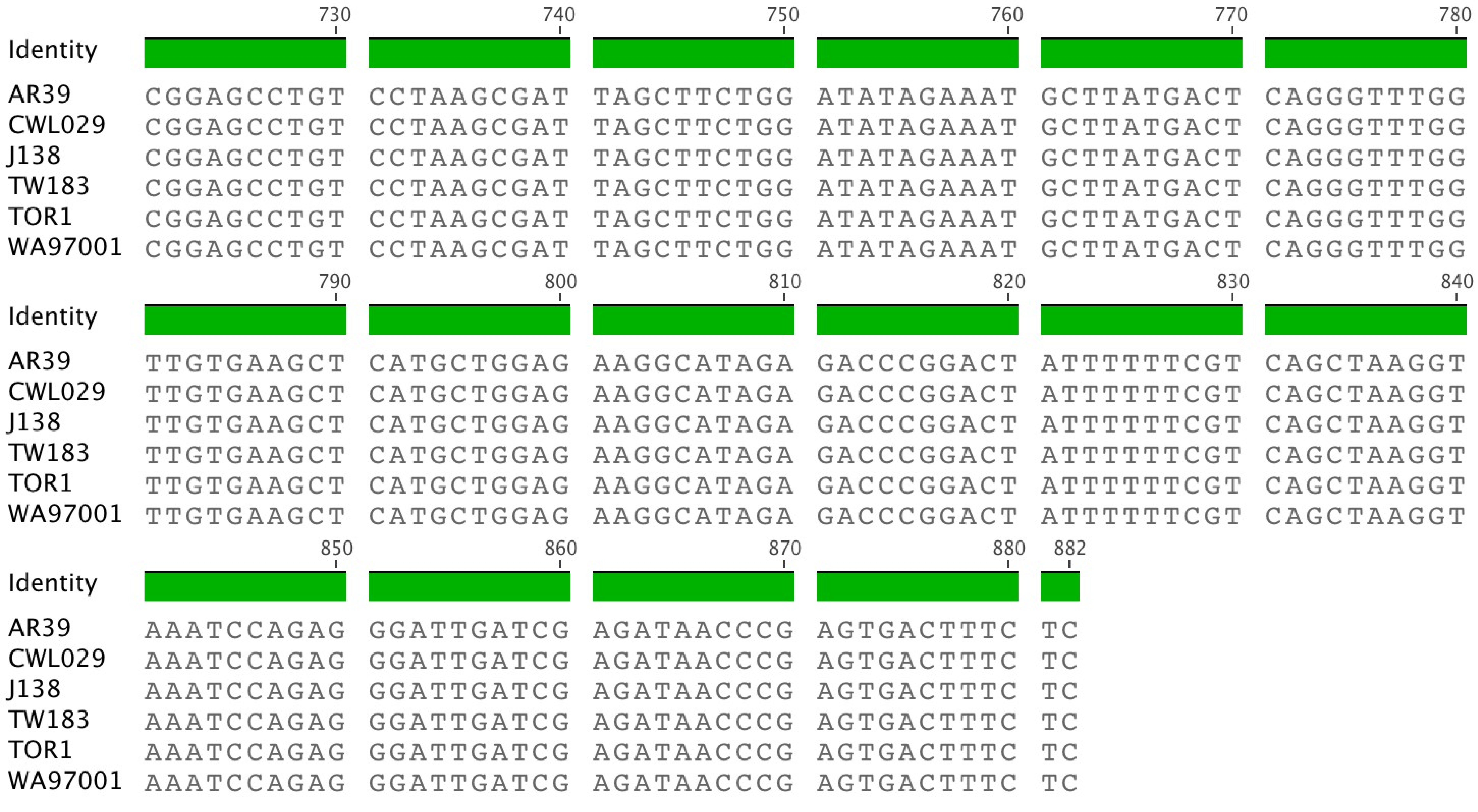
730 740 750 760 770 780
Identity
AR39 CGGAGCCTGT CCTAAGCGAT TAGCTTCTGG ATATAGAAAT GCTTATGACT CAGGGTTTGG
CWL029 CGGAGCCTGT CCTAAGCGAT TAGCTTCTGG ATATAGAAAT GCTTATGACT CAGGGTTTGG
J138 CGGAGCCTGT CCTAAGCGAT TAGCTTCTGG ATATAGAAAT GCTTATGACT CAGGGTTTGG
TW183 CGGAGCCTGT CCTAAGCGAT TAGCTTCTGG ATATAGAAAT GCTTATGACT CAGGGTTTGG
TOR1 CGGAGCCTGT CCTAAGCGAT TAGCTTCTGG ATATAGAAAT GCTTATGACT CAGGGTTTGG
WA97001 CGGAGCCTGT CCTAAGCGAT TAGCTTCTGG ATATAGAAAT GCTTATGACT CAGGGTTTGG
790 800 810 820 830 840
Identity
AR39 TTGTGAAGCT CATGCTGGAG AAGGCATAGA GACCCGGACT ATTTTTTCGT CAGCTAAGGT
CWL029 TTGTGAAGCT CATGCTGGAG AAGGCATAGA GACCCGGACT ATTTTTTCGT CAGCTAAGGT
J138 TTGTGAAGCT CATGCTGGAG AAGGCATAGA GACCCGGACT ATTTTTTCGT CAGCTAAGGT
TW183 TTGTGAAGCT CATGCTGGAG AAGGCATAGA GACCCGGACT ATTTTTTCGT CAGCTAAGGT
TOR1 TTGTGAAGCT CATGCTGGAG AAGGCATAGA GACCCGGACT ATTTTTTCGT CAGCTAAGGT
WA97001 TTGTGAAGCT CATGCTGGAG AAGGCATAGA GACCCGGACT ATTTTTTCGT CAGCTAAGGT
850 860 870 880 882
Identity
AR39 AAATCCAGAG GGATTGATCG AGATAACCCG AGTGACTTTC TC
CWL029 AAATCCAGAG GGATTGATCG AGATAACCCG AGTGACTTTC TC
J138 AAATCCAGAG GGATTGATCG AGATAACCCG AGTGACTTTC TC
TW183 AAATCCAGAG GGATTGATCG AGATAACCCG AGTGACTTTC TC
TOR1 AAATCCAGAG GGATTGATCG AGATAACCCG AGTGACTTTC TC
WA97001 AAATCCAGAG GGATTGATCG AGATAACCCG AGTGACTTTC TC

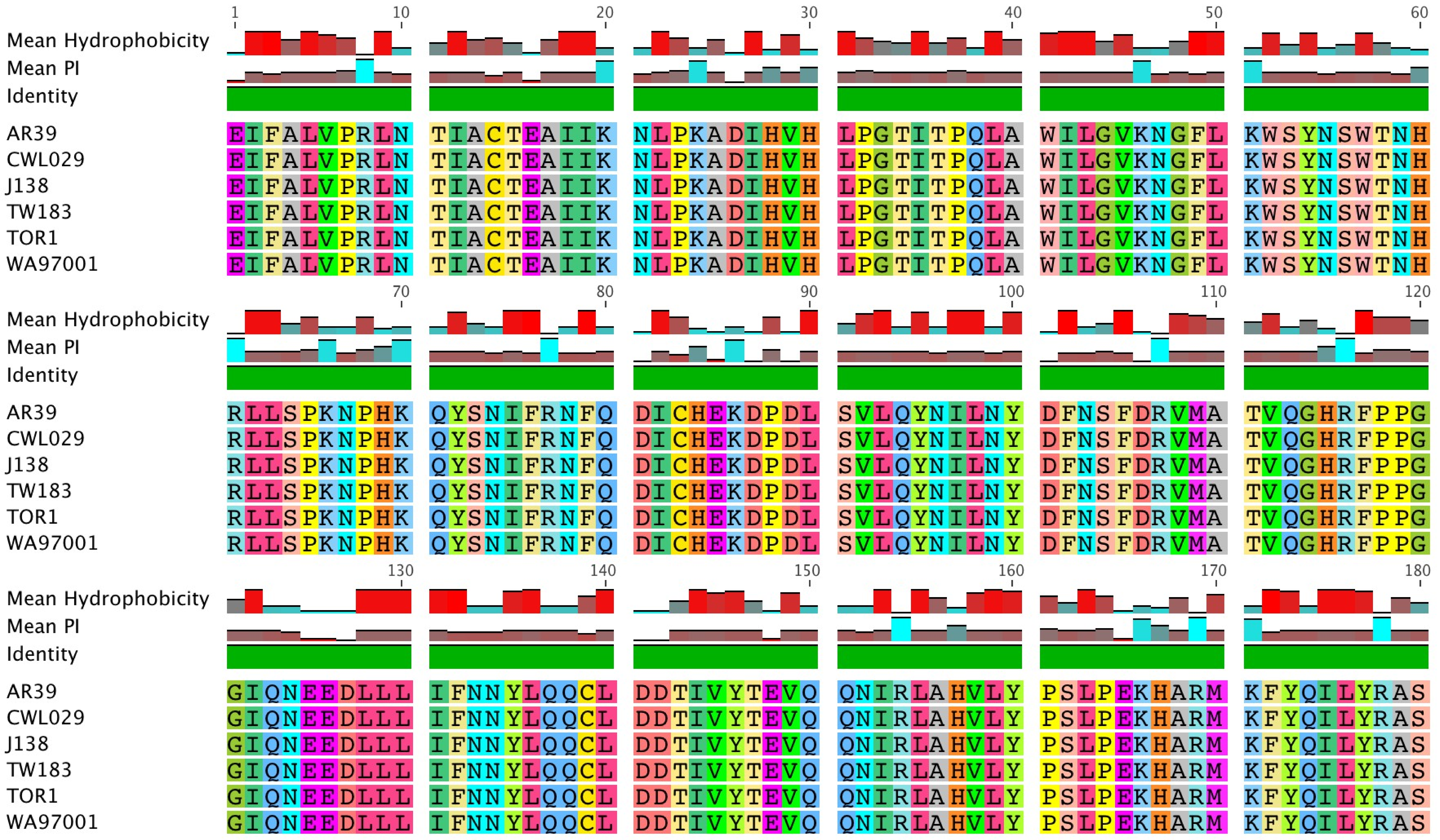
1 10 20 30 40 50 60
Mean Hydrophobicity
Mean PI
Identity
AR39 EIFALVPRLN TIACTEAIIK NLPKADIHVH LPGTITPQLA WILGVKNGFL KWSYNSWTNH
CWL029 EIFALVPRLN TIACTEAIIK NLPKADIHVH LPGTITPQLA WILGVKNGFL KWSYNSWTNH
J138 EIFALVPRLN TIACTEAIIK NLPKADIHVH LPGTITPQLA WILGVKNGFL KWSYNSWTNH
TW183 EIFALVPRLN TIACTEAIIK NLPKADIHVH LPGTITPQLA WILGVKNGFL KWSYNSWTNH
TOR1 EIFALVPRLN TIACTEAIIK NLPKADIHVH LPGTITPQLA WILGVKNGFL KWSYNSWTNH
WA97001 EIFALVPRLN TIACTEAIIK NLPKADIHVH LPGTITPQLA WILGVKNGFL KWSYNSWTNH
70 80 90 100 110 120
Mean Hydrophobicity
Mean PI
Identity
AR39 RLLSPKNPHK QYSNIFRNFQ DICHEKDPDL SVLQYNILNY DFNSFDRVMA TVQGHRFPPG
CWL029 RLLSPKNPHK QYSNIFRNFQ DICHEKDPDL SVLQYNILNY DFNSFDRVMA TVQGHRFPPG
J138 RLLSPKNPHK QYSNIFRNFQ DICHEKDPDL SVLQYNILNY DFNSFDRVMA TVQGHRFPPG
TW183 RLLSPKNPHK QYSNIFRNFQ DICHEKDPDL SVLQYNILNY DFNSFDRVMA TVQGHRFPPG
TOR1 RLLSPKNPHK QYSNIFRNFQ DICHEKDPDL SVLQYNILNY DFNSFDRVMA TVQGHRFPPG
WA97001 RLLSPKNPHK QYSNIFRNFQ DICHEKDPDL SVLQYNILNY DFNSFDRVMA TVQGHRFPPG
130 140 150 160 170 180
Mean Hydrophobicity
Mean PI
Identity
AR39 GIQNEEDLLL IFNNYLQQCL DDTIVYTEVQ QNIRLAHVLY PSLPEKHARM KFYQILYRAS
CWL029 GIQNEEDLLL IFNNYLQQCL DDTIVYTEVQ QNIRLAHVLY PSLPEKHARM KFYQILYRAS
J138 GIQNEEDLLL IFNNYLQQCL DDTIVYTEVQ QNIRLAHVLY PSLPEKHARM KFYQILYRAS
TW183 GIQNEEDLLL IFNNYLQQCL DDTIVYTEVQ QNIRLAHVLY PSLPEKHARM KFYQILYRAS
TOR1 GIQNEEDLLL IFNNYLQQCL DDTIVYTEVQ QNIRLAHVLY PSLPEKHARM KFYQILYRAS
WA97001 GIQNEEDLLL IFNNYLQQCL DDTIVYTEVQ QNIRLAHVLY PSLPEKHARM KFYQILYRAS

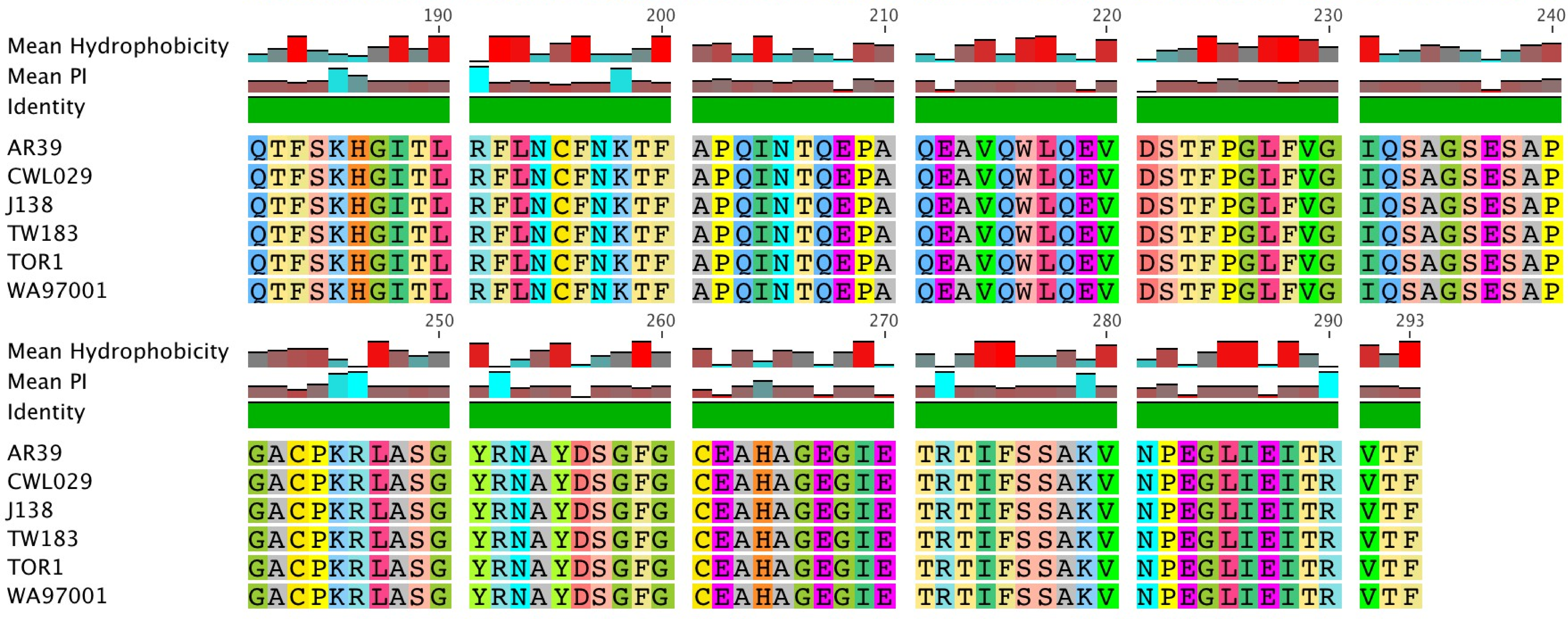

190
200
210
220
230
240
Mean Hydrophobicity
Mean PI
Identity
AR39 QTFSKHGITL RFLNCFNKTF APQINTQEPA QEAVQWLQEV DSTFPGLFVG IQSAGSESAP
CWL029 QTFSKHGITL RFLNCFNKTF APQINTQEPA QEAVQWLQEV DSTFPGLFVG IQSAGSESAP
J138 QTFSKHGITL RFLNCFNKTF APQINTQEPA QEAVQWLQEV DSTFPGLFVG IQSAGSESAP
TW183 QTFSKHGITL RFLNCFNKTF APQINTQEPA QEAVQWLQEV DSTFPGLFVG IQSAGSESAP
TOR1 QTFSKHGITL RFLNCFNKTF APQINTQEPA QEAVQWLQEV DSTFPGLFVG IQSAGSESAP
WA97001 QTFSKHGITL RFLNCFNKTF APQINTQEPA QEAVQWLQEV DSTFPGLFVG IQSAGSESAP
250
260
270
280
290
293
Mean Hydrophobicity
Mean PI
Identity
AR39 GACPKRLASG YRNAYDSGFG CEAHAGEGIE TRTIFSSAKV NPEGLIEITR VTF
CWL029 GACPKRLASG YRNAYDSGFG CEAHAGEGIE TRTIFSSAKV NPEGLIEITR VTF
J138 GACPKRLASG YRNAYDSGFG CEAHAGEGIE TRTIFSSAKV NPEGLIEITR VTF
TW183 GACPKRLASG YRNAYDSGFG CEAHAGEGIE TRTIFSSAKV NPEGLIEITR VTF
TOR1 GACPKRLASG YRNAYDSGFG CEAHAGEGIE TRTIFSSAKV NPEGLIEITR VTF
WA97001 GACPKRLASG YRNAYDSGFG CEAHAGEGIE TRTIFSSAKV NPEGLIEITR VTF
