# Supplementary material for: Chlamydia pneumoniae Is Genetically Diverse in Animals and Appears to Have Crossed the Host Barrier to Humans on (At Least) Two Occasions
Source: PLoS Pathog. 2010 May 20;6(5):e1000903. doi: 10.1371/journal.ppat.1000903 (PMC2873915; doi:10.1371/journal.ppat.1000903)
Supplement: Figure S20 — Multiple sequence alignment of CPK_ORF00678. Horse N16 has four indels (positions 73-84, 151-305, 387-395 and 419-425), relative to frog DE177, bandicoot B26 and koala LPCoLN. (0.77 MB PDF) [file ppat.1000903.s020.pdf]

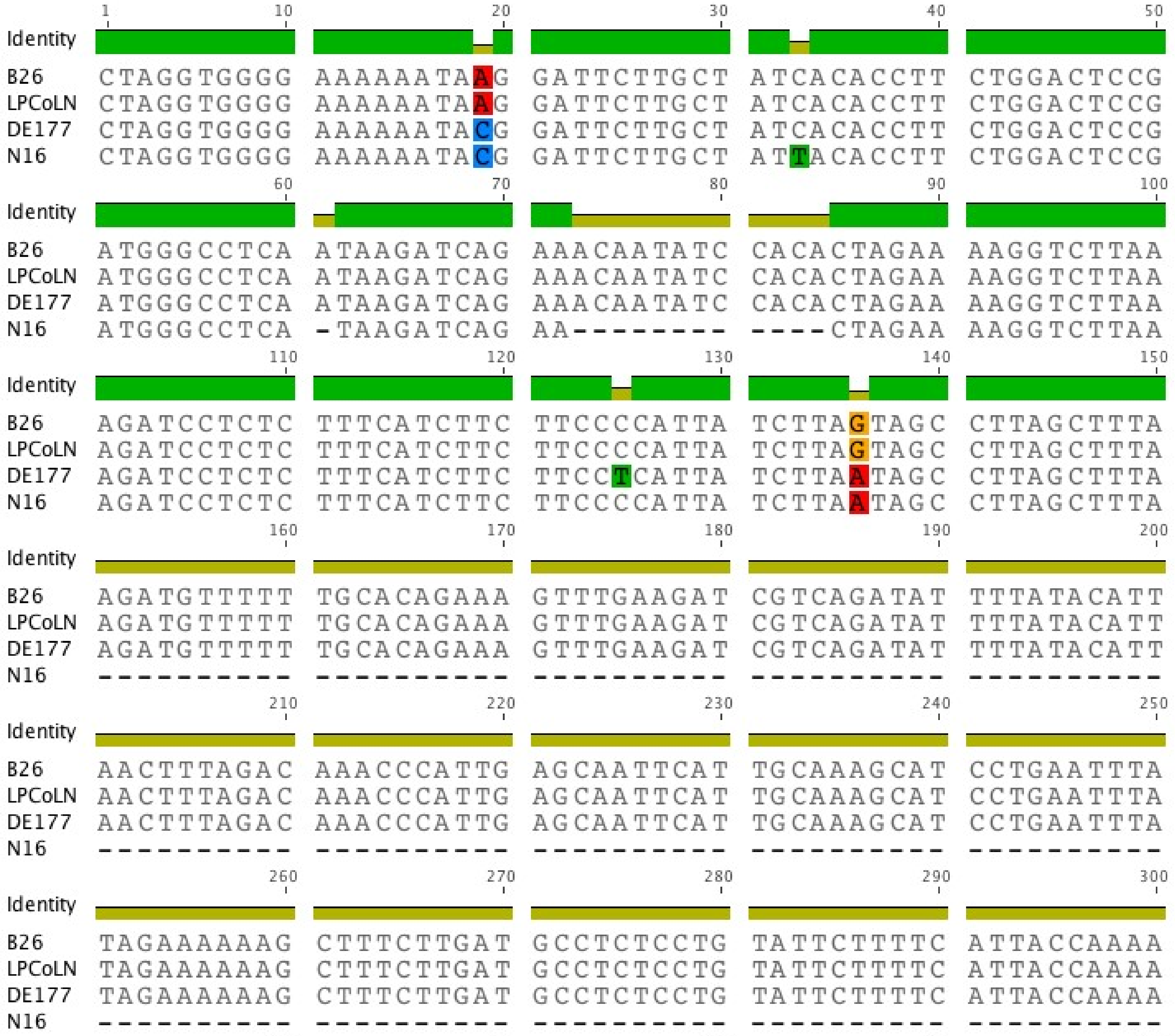

1 10 20 30 40 50
Identity
B26 CTAGGTGGGG AAAAAATAAG GATTCTTGCT ATCACACCTT CTGGACTCCG
LPCoLN CTAGGTGGGG AAAAAATAAG GATTCTTGCT ATCACACCTT CTGGACTCCG
DE177 CTAGGTGGGG AAAAAATACG GATTCTTGCT ATCACACCTT CTGGACTCCG
N16 CTAGGTGGGG AAAAAATACG GATTCTTGCT ATTTACACCTT CTGGACTCCG
60 70 80 90 100
Identity
B26 ATGGGCCTCA ATAAGATCAG AAACAATATC CACACTAGAA AAGGTCTTAA
LPCoLN ATGGGCCTCA ATAAGATCAG AAACAATATC CACACTAGAA AAGGTCTTAA
DE177 ATGGGCCTCA ATAAGATCAG AAACAATATC CACACTAGAA AAGGTCTTAA
N16 ATGGGCCTCA -TAAGATCAG AA-------- ----CTAGAA AAGGTCTTAA
110 120 130 140 150
Identity
B26 AGATCCTCTC TTTCATCTTC TTCCCCATTA TCTTAGTAGC CTTAGCTTTA
LPCoLN AGATCCTCTC TTTCATCTTC TTCCCCATTA TCTTAGTAGC CTTAGCTTTA
DE177 AGATCCTCTC TTTCATCTTC TTCCTCATTA TCTTAATAGC CTTAGCTTTA
N16 AGATCCTCTC TTTCATCTTC TTCCCCATTA TCTTAATAGC CTTAGCTTTA
160 170 180 190 200
Identity
B26 AGATGTTTTT TGCACAGAAA GTTTGAAGAT CGTCAGATAT TTTATACATT
LPCoLN AGATGTTTTT TGCACAGAAA GTTTGAAGAT CGTCAGATAT TTTATACATT
DE177 AGATGTTTTT TGCACAGAAA GTTTGAAGAT CGTCAGATAT TTTATACATT
N16 ---------- ---------- ---------- ---------- ----------
210 220 230 240 250
Identity
B26 AACTTTAGAC AAACCCATTG AGCAATTCAT TGCAAAGCAT CCTGAATTTA
LPCoLN AACTTTAGAC AAACCCATTG AGCAATTCAT TGCAAAGCAT CCTGAATTTA
DE177 AACTTTAGAC AAACCCATTG AGCAATTCAT TGCAAAGCAT CCTGAATTTA
N16 ---------- ---------- ---------- ---------- ----------
260 270 280 290 300
Identity
B26 TAGAAAAAAG CTTTCTTGAT GCCTCTCCTG TATTCTTTTC ATTACCAAAA
LPCoLN TAGAAAAAAG CTTTCTTGAT GCCTCTCCTG TATTCTTTTC ATTACCAAAA
DE177 TAGAAAAAAG CTTTCTTGAT GCCTCTCCTG TATTCTTTTC ATTACCAAAA
N16 ---------- ---------- ---------- ---------- ----------

	310	320	330	340	350
B26	ACTATGCGTT	TTTTCGATAT	CTCCATACCT	GAAGGCACTT	CTTGTGTAAA
LPCoLN	ACTATGCGTT	TTTTCGATAT	CTCCATACCT	GAAGGCACTT	CTTGTGTAAA
DE177	ACTATGCGTT	TTTTCGATAT	CTCCATACCT	GAAGGCACTT	CTTGTGTAAA
N16	-----GCGTT	TTTTCGACAT	CTCTATACCT	GAAGGCACTT	CTTGTGTAAA

	360	370	380	390	400
B26	GATTACTCAA	TCCATAAAAA	CGGATAAAAT	AGTAAACTCT	ATTAATTCTA
LPCoLN	GATTACTCAA	TCCATAAAAA	CGGATAAAAT	AGTAAACTCT	ATTAATTCTA
DE177	GATTACTCAA	TCCATAAAAA	CGGATAAAAT	AGTAAATTCT	ATTAATTCTA
N16	GATTACTCAA	TCCATAAAAA	CGGATAAAAT	AGTAAA----	-----TTCTA

	410	420	430	440	450
B26	TTAATATAGA	TACTCTCCCC	TGGGCCTCTT	CATGTCTTCA	CTTAGATATG
LPCoLN	TTAATATAGA	TACTCTCCCC	TGGGCCTCTT	CATGTCTTCA	CTTAGATATG
DE177	TTAATATAGA	TACTCTCCCC	TGGGCCTCTT	CATGTCTTCA	CTTAGATATG
N16	TTAATATAGA	TACTCTCC--	-----CTCTT	CATGTCTTCA	CTTAGATATG

	460	470	480	490	500
B26	GGGTTTACAA	GTGAGCACCA	TCGCCTTCTT	ATTGAAGACC	TAAAAAAGAA
LPCoLN	GGGTTTACAA	GTGAGCACCA	TCGCCTTCTT	ATTGAAGACC	TAAAAAAGAA
DE177	GGGTTTACAA	ATGAGCACCA	TCGCCTTCTT	ATTGAAGACC	TAAAAAAGAA
N16	GGGTTTACAA	ATGAGCACCA	TCGCCTTCTT	ATTGAAGACC	TAAAAAAGAA

	510	520	530	540	550
B26	AGAATATCTT	TCACAAATTA	GTCACGAAGG	TAAGAAACGC	CTTATAAAAA
LPCoLN	AGAATATCTT	TCACAAATTA	GTCACGAAGG	TAAGAAACGC	CTTATAAAAA
DE177	AGAATATCTT	TCACAAATTA	GTCACGAAGG	TAAGAAACGC	CTTATAAAAA
N16	AGAATATCTT	TCACAAATTA	GTCACGAAGG	TAAGAAAACC	CTTTTAAAAA

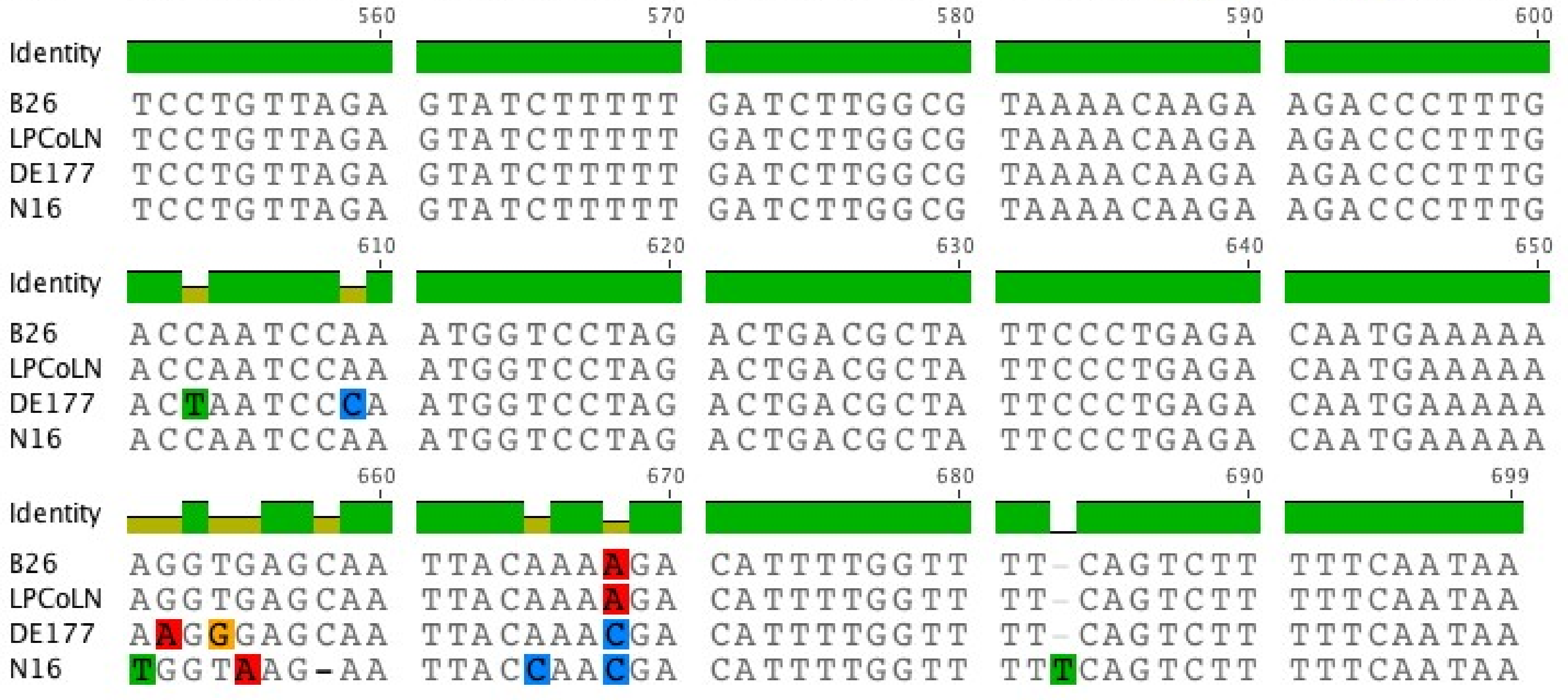
560 570 580 590 600
Identity
B26 TCCTGTTAGA GTATCTTTTT GATCTTGGCG TAAAACAAGA AGACCCTTTG
LPCoLN TCCTGTTAGA GTATCTTTTT GATCTTGGCG TAAAACAAGA AGACCCTTTG
DE177 TCCTGTTAGA GTATCTTTTT GATCTTGGCG TAAAACAAGA AGACCCTTTG
N16 TCCTGTTAGA GTATCTTTTT GATCTTGGCG TAAAACAAGA AGACCCTTTG
610 620 630 640 650
Identity
B26 ACCAATCCAA ATGGTCCTAG ACTGACGCTA TTCCCTGAGA CAATGAAAAA
LPCoLN ACCAATCCAA ATGGTCCTAG ACTGACGCTA TTCCCTGAGA CAATGAAAAA
DE177 ACTAATCCCA ATGGTCCTAG ACTGACGCTA TTCCCTGAGA CAATGAAAAA
N16 ACCAATCCAA ATGGTCCTAG ACTGACGCTA TTCCCTGAGA CAATGAAAAA
660 670 680 690 699
Identity
B26 AGGTGAGCAA TTACAAAAGA CATTTTGGTT TT-CAGTCTT TTTCAATAA
LPCoLN AGGTGAGCAA TTACAAAAGA CATTTTGGTT TT-CAGTCTT TTTCAATAA
DE177 AAGGGAGCAA TTACAAACGA CATTTTGGTT TT-CAGTCTT TTTCAATAA
N16 TGGTAAG-AA TTACCAACGA CATTTTGGTT TTTCAGTCTT TTTCAATAA

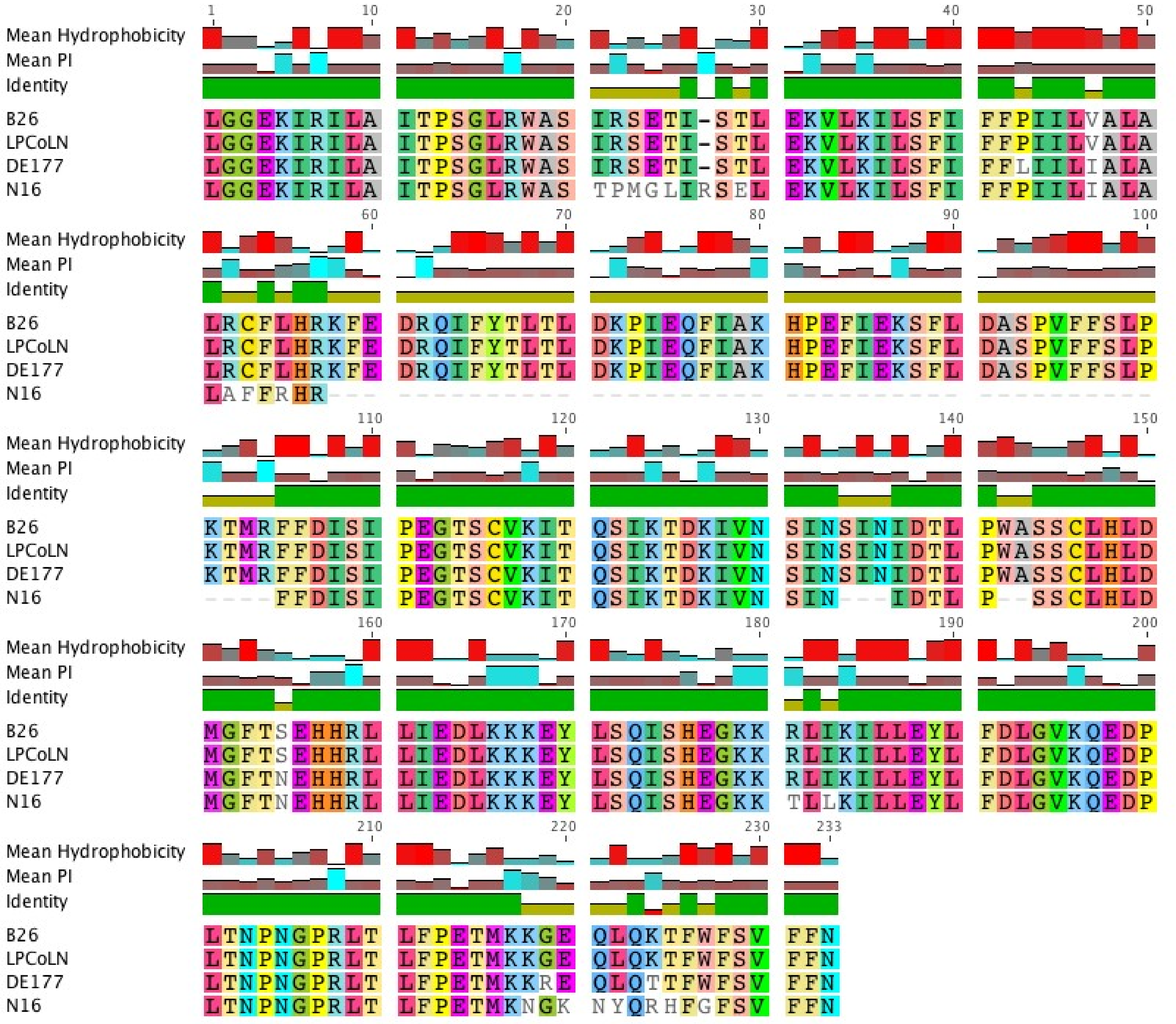

Mean Hydrophobicity
Mean PI
Identity
B26 LGGEKIRILA ITPSGLRWAS IRSETI-STL EKVLKILSFI FFPIILVALA
LPCoLN LGGEKIRILA ITPSGLRWAS IRSETI-STL EKVLKILSFI FFPIILVALA
DE177 LGGEKIRILA ITPSGLRWAS IRSETI-STL EKVLKILSFI FFLIILIALA
N16 LGGEKIRILA ITPSGLRWAS TPMGLIRSEL EKVLKILSFI FFPIILIALA
B26 LRCFLHRKFE DRQIFYTLTL DKPIEQFIAK HPEFIEKSFL DASPVFFSLP
LPCoLN LRCFLHRKFE DRQIFYTLTL DKPIEQFIAK HPEFIEKSFL DASPVFFSLP
DE177 LRCFLHRKFE DRQIFYTLTL DKPIEQFIAK HPEFIEKSFL DASPVFFSLP
N16 LAFFRHR---
B26 KTMRFFDISI PEGTSCVKIT QSIKTDKIVN SINSINIDTL PWASSCLHLD
LPCoLN KTMRFFDISI PEGTSCVKIT QSIKTDKIVN SINSINIDTL PWASSCLHLD
DE177 KTMRFFDISI PEGTSCVKIT QSIKTDKIVN SINSINIDTL PWASSCLHLD
N16 ----FFDISI PEGTSCVKIT QSIKTDKIVN SIN---IDTL P--SSCLHLD
B26 MGFTSEHHRL LIEDLKKKEY LSQISHEGKK RLIKILLEYL FDLGVKQEDP
LPCoLN MGFTSEHHRL LIEDLKKKEY LSQISHEGKK RLIKILLEYL FDLGVKQEDP
DE177 MGFTNEHHRL LIEDLKKKEY LSQISHEGKK RLIKILLEYL FDLGVKQEDP
N16 MGFTNEHHRL LIEDLKKKEY LSQISHEGKK TLLKILLEYL FDLGVKQEDP
B26 LTNPNGPRLT LFPETMKKGE QLQKTFWFSV FFN
LPCoLN LTNPNGPRLT LFPETMKKGE QLQKTFWFSV FFN
DE177 LTNPNGPRLT LFPETMKKRE QLQTTFWFSV FFN
N16 LTNPNGPRLT LFPETMKNGK NYQRHFGFSV FFN
