# Supplementary material for: Chlamydia pneumoniae Is Genetically Diverse in Animals and Appears to Have Crossed the Host Barrier to Humans on (At Least) Two Occasions
Source: PLoS Pathog. 2010 May 20;6(5):e1000903. doi: 10.1371/journal.ppat.1000903 (PMC2873915; doi:10.1371/journal.ppat.1000903)
Supplement: Figure S21 — Multiple sequence alignment of SSR2. Horse N16 has a 165 bp indel at positions 66-230 bp. (0.58 MB PDF) [file ppat.1000903.s021.pdf]

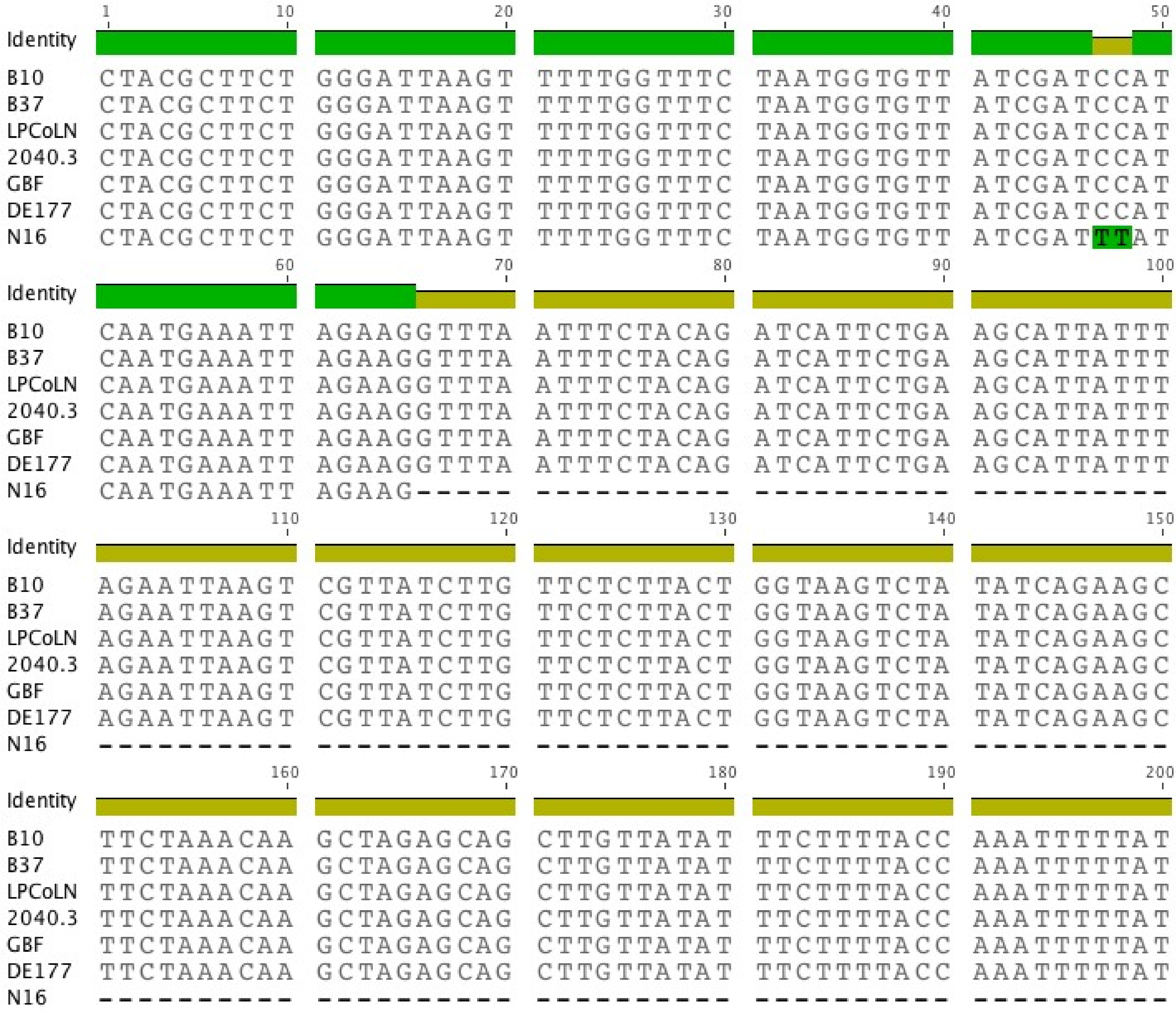

1 10 20 30 40 50
Identity
B10 CTACGCTTCT GGGATTAAGT TTTTGGTTTC TAATGGTGTT ATCGATCCAT
B37 CTACGCTTCT GGGATTAAGT TTTTGGTTTC TAATGGTGTT ATCGATCCAT
LPCoLN CTACGCTTCT GGGATTAAGT TTTTGGTTTC TAATGGTGTT ATCGATCCAT
2040.3 CTACGCTTCT GGGATTAAGT TTTTGGTTTC TAATGGTGTT ATCGATCCAT
GBF CTACGCTTCT GGGATTAAGT TTTTGGTTTC TAATGGTGTT ATCGATCCAT
DE177 CTACGCTTCT GGGATTAAGT TTTTGGTTTC TAATGGTGTT ATCGATCCAT
N16 CTACGCTTCT GGGATTAAGT TTTTGGTTTC TAATGGTGTT ATCGATTTAT
60 70 80 90 100
Identity
B10 CAATGAAATT AGAAGGTTTA ATTTCTACAG ATCATTCTGA AGCATTATTT
B37 CAATGAAATT AGAAGGTTTA ATTTCTACAG ATCATTCTGA AGCATTATTT
LPCoLN CAATGAAATT AGAAGGTTTA ATTTCTACAG ATCATTCTGA AGCATTATTT
2040.3 CAATGAAATT AGAAGGTTTA ATTTCTACAG ATCATTCTGA AGCATTATTT
GBF CAATGAAATT AGAAGGTTTA ATTTCTACAG ATCATTCTGA AGCATTATTT
DE177 CAATGAAATT AGAAGGTTTA ATTTCTACAG ATCATTCTGA AGCATTATTT
N16 CAATGAAATT AGAAG----- ---------- ---------- ----------
110 120 130 140 150
Identity
B10 AGAATTAAGT CGTTATCTTG TTCTCTTACT GGTAAGTCTA TATCAGAAGC
B37 AGAATTAAGT CGTTATCTTG TTCTCTTACT GGTAAGTCTA TATCAGAAGC
LPCoLN AGAATTAAGT CGTTATCTTG TTCTCTTACT GGTAAGTCTA TATCAGAAGC
2040.3 AGAATTAAGT CGTTATCTTG TTCTCTTACT GGTAAGTCTA TATCAGAAGC
GBF AGAATTAAGT CGTTATCTTG TTCTCTTACT GGTAAGTCTA TATCAGAAGC
DE177 AGAATTAAGT CGTTATCTTG TTCTCTTACT GGTAAGTCTA TATCAGAAGC
N16 ---------- ---------- ---------- ---------- ----------
160 170 180 190 200
Identity
B10 TTCTAAACAA GCTAGAGCAG CTTGTTATAT TTCTTTTACC AAATTTTTAT
B37 TTCTAAACAA GCTAGAGCAG CTTGTTATAT TTCTTTTACC AAATTTTTAT
LPCoLN TTCTAAACAA GCTAGAGCAG CTTGTTATAT TTCTTTTACC AAATTTTTAT
2040.3 TTCTAAACAA GCTAGAGCAG CTTGTTATAT TTCTTTTACC AAATTTTTAT
GBF TTCTAAACAA GCTAGAGCAG CTTGTTATAT TTCTTTTACC AAATTTTTAT
DE177 TTCTAAACAA GCTAGAGCAG CTTGTTATAT TTCTTTTACC AAATTTTTAT
N16 ---------- ---------- ---------- ---------- ----------

	210	220	230	240	250
Identity					
B10	ATCGCTTAAC	CAAAGGTGTT	GTTAGACAAG	CTTCTCCAAT	TAGGGACTTT
B37	ATCGCTTAAC	CAAAGGTGTT	GTTAGACAAG	CTTCTCCAAT	TAGGGACTTT
LPCoLN	ATCGCTTAAC	CAAAGGTGTT	GTTAGACAAG	CTTCTCCAAT	TAGGGACTTT
2040.3	ATCGCTTAAC	CAAAGGTGTT	GTTAGACAAG	CTTCTCCAAT	TAGGGACTTT
GBF	ATCGCTTAAC	CAAAGGTGTT	GTTAGACAAG	CTTCTCCAAT	TAGGGACTTT
DE177	ATCGCTTAAC	CAAAGGTGTT	GTTAGACAAG	CTTCTCCAAT	TAGGGACTTT
N16	----------	----------	----------	CTTCTCCAAT	TAGGGACTTT

	260	270	280	290	300
Identity					
B10	GGCAACGCAA	CATTTTATAA	AATCCGAGAC	AAAGTAAAGA	CGGAGTTTAT
B37	GGCAACGCAA	CATTTTATAA	AATCCGAGAC	AAAGTAAAGA	CGGAGTTTAT
LPCoLN	GGCAACGCAA	CATTTTATAA	AATCCGAGAC	AAAGTAAAGA	CGGAGTTTAT
2040.3	GGCAACGCAA	CATTTTATAA	AATCCGAGAC	AAAGTAAAGA	CGGAGTTTAT
GBF	GGCAACGCAA	CATTTTATAA	AATCCGAGAC	AAAGTAAAGA	CGGAGTTTAT
DE177	GGCAACGCAA	CATTTTATAA	AATCCGAGAC	AAAGTAAAGA	CGGAGTTTAT
N16	GGCAATGCAA	CATTTTATAA	AATCCGAGAC	AAAGTAAGGA	CGGAGTTTAT

	310	320	330	340	350
Identity					
B10	TTCTAAACGA	GAGTGGCTAT	TGTTTTTTGA	TTCTTTAAAA	AAAAGAAGCT
B37	TTCTAAACGA	GAGTGGCTAT	TGTTTTTTGA	TTCTTTAAAA	AAAAGAAGCT
LPCoLN	TTCTAAACGA	GAGTGGCTAT	TGTTTTTTGA	TTCTTTAAAA	AAAAGAAGCT
2040.3	TTCTAAACGA	GAGTGGCTAT	TGTTTTTTGA	TTCTTTAAAA	AAAAGAAGCT
GBF	TTCTAAACGA	GAGTGGCTAT	TGTTTTTTGA	TTCTTTAAAA	AAAAGAAGCT
DE177	TTCTAAACGA	GAGTGGCTAT	TGTTTTTTGA	TTCTTTAAAA	AAAAGAAGCT
N16	TTCTAAACGA	GAGTAGCTAT	TGTTTTTTGA	TTCTTTAAAA	AAAAGAAGCT

	360	369
Identity		
B10	ACAGAGATTA	TTTGATTGG
B37	ACAGAGATTA	TTTGATTGG
LPCoLN	ACAGAGATTA	TTTGATTGG
2040.3	ACAGAGATTA	TTTGATTGG
GBF	ACAGAGATTA	TTTGATTGG
DE177	ACAGAGATTA	TTTGATTGG
N16	ACAGAGATTA	TTTGATTGG

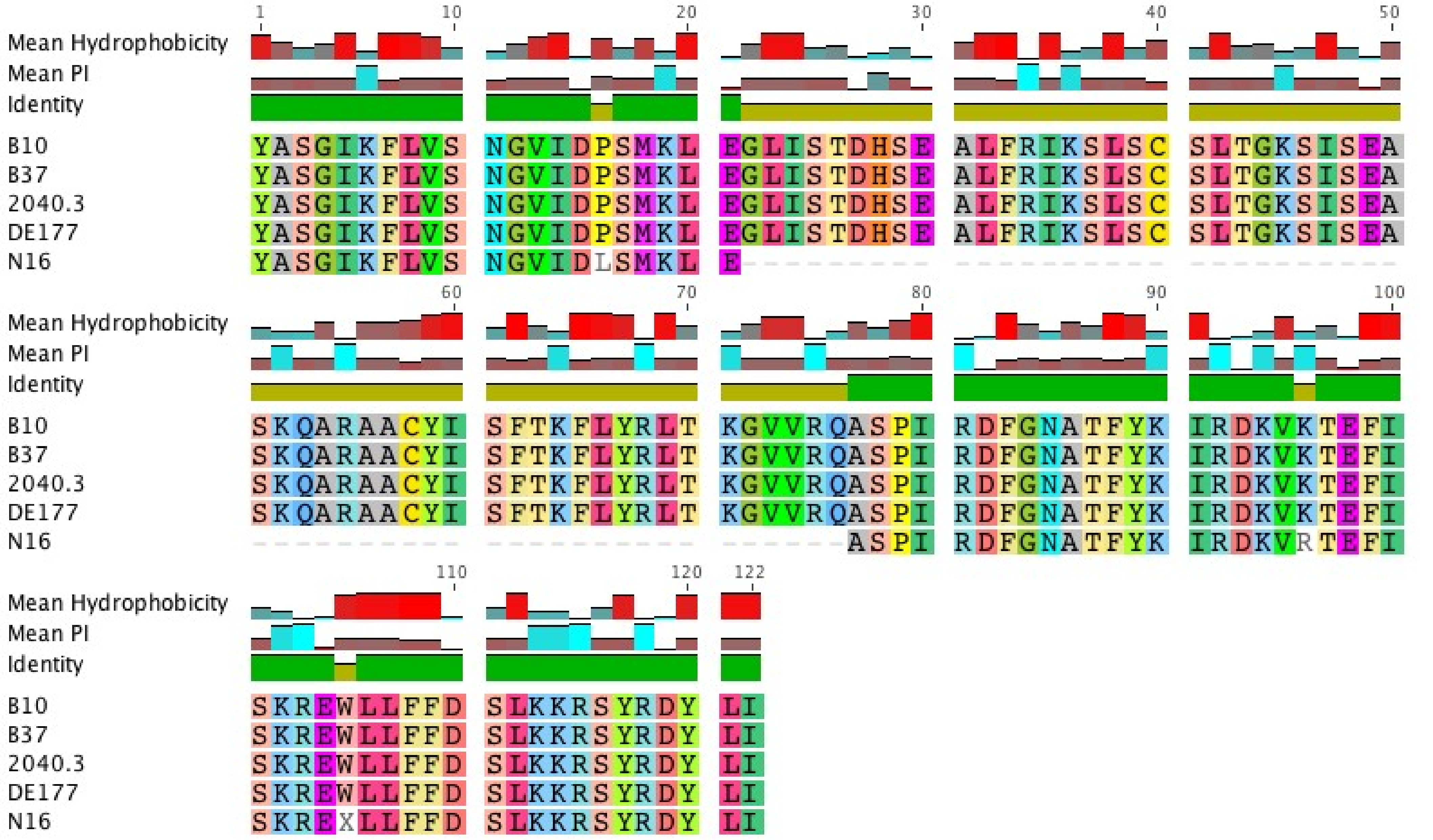

1 10 20 30 40 50
Mean Hydrophobicity
Mean PI
Identity
B10 YASGIKFLVS NGVIDPSMKL EGLISTDHSE ALFRIKSLSC SLTGKSISEA
B37 YASGIKFLVS NGVIDPSMKL EGLISTDHSE ALFRIKSLSC SLTGKSISEA
2040.3 YASGIKFLVS NGVIDPSMKL EGLISTDHSE ALFRIKSLSC SLTGKSISEA
DE177 YASGIKFLVS NGVIDPSMKL EGLISTDHSE ALFRIKSLSC SLTGKSISEA
N16 YASGIKFLVS NGVIDLSMKL E
60 70 80 90 100
Mean Hydrophobicity
Mean PI
Identity
B10 SKQARAACYI SFTKFLYRLT KGVVRQASPI RDFGNATFYK IRDKVKTEFI
B37 SKQARAACYI SFTKFLYRLT KGVVRQASPI RDFGNATFYK IRDKVKTEFI
2040.3 SKQARAACYI SFTKFLYRLT KGVVRQASPI RDFGNATFYK IRDKVKTEFI
DE177 SKQARAACYI SFTKFLYRLT KGVVRQASPI RDFGNATFYK IRDKVKTEFI
N16 ASPI RDFGNATFYK IRDKVRTEFI
110 120 122
Mean Hydrophobicity
Mean PI
Identity
B10 SKREWLLFFD SLKKRSYRDY LI
B37 SKREWLLFFD SLKKRSYRDY LI
2040.3 SKREWLLFFD SLKKRSYRDY LI
DE177 SKREWLLFFD SLKKRSYRDY LI
N16 SKREXLLFFD SLKKRSYRDY LI
