# Supplementary material for: Chlamydia pneumoniae Is Genetically Diverse in Animals and Appears to Have Crossed the Host Barrier to Humans on (At Least) Two Occasions
Source: PLoS Pathog. 2010 May 20;6(5):e1000903. doi: 10.1371/journal.ppat.1000903 (PMC2873915; doi:10.1371/journal.ppat.1000903)
Supplement: Figure S22 — Multiple sequence alignment of helicase. The nucleotide and amino acid alignments were generated using Geneious version 4.7, where each nucleotide and amino acid is assigned its own colour. White shading indicates an amino acid variant. (0.64 MB PDF) [file ppat.1000903.s022.pdf]

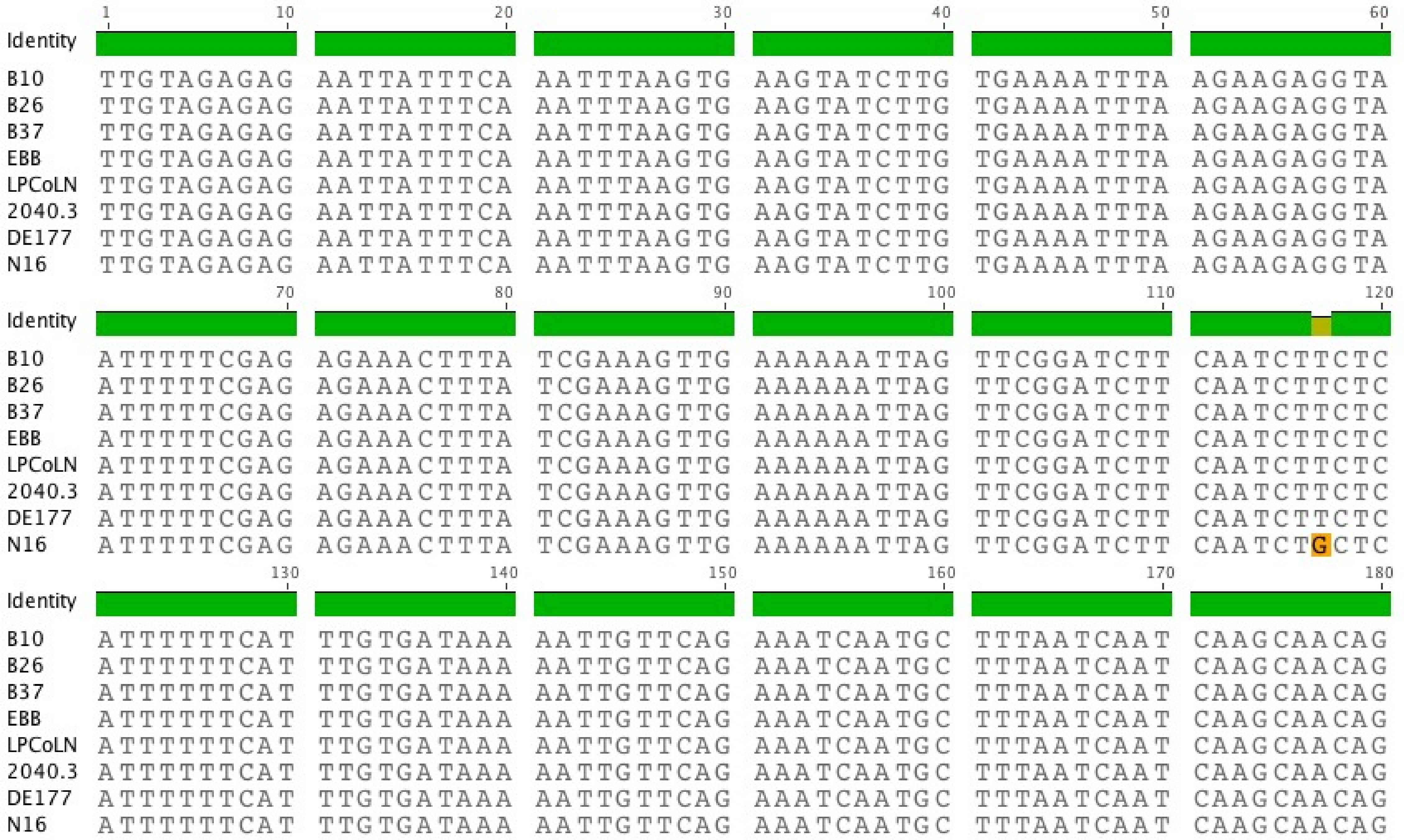
1 10 20 30 40 50 60
Identity
B10 TTGTAGAGAG AATTATTTCA AATTTAAGTG AAGTATCTTG TGAAAATTTA AGAAGAGGTA
B26 TTGTAGAGAG AATTATTTCA AATTTAAGTG AAGTATCTTG TGAAAATTTA AGAAGAGGTA
B37 TTGTAGAGAG AATTATTTCA AATTTAAGTG AAGTATCTTG TGAAAATTTA AGAAGAGGTA
EBB TTGTAGAGAG AATTATTTCA AATTTAAGTG AAGTATCTTG TGAAAATTTA AGAAGAGGTA
LPCoLN TTGTAGAGAG AATTATTTCA AATTTAAGTG AAGTATCTTG TGAAAATTTA AGAAGAGGTA
2040.3 TTGTAGAGAG AATTATTTCA AATTTAAGTG AAGTATCTTG TGAAAATTTA AGAAGAGGTA
DE177 TTGTAGAGAG AATTATTTCA AATTTAAGTG AAGTATCTTG TGAAAATTTA AGAAGAGGTA
N16 TTGTAGAGAG AATTATTTCA AATTTAAGTG AAGTATCTTG TGAAAATTTA AGAAGAGGTA
70 80 90 100 110 120
Identity
B10 ATTTTTCGAG AGAAACTTTA TCGAAAGTTG AAAAAATTAG TTCGGATCTT CAATCTTCTC
B26 ATTTTTCGAG AGAAACTTTA TCGAAAGTTG AAAAAATTAG TTCGGATCTT CAATCTTCTC
B37 ATTTTTCGAG AGAAACTTTA TCGAAAGTTG AAAAAATTAG TTCGGATCTT CAATCTTCTC
EBB ATTTTTCGAG AGAAACTTTA TCGAAAGTTG AAAAAATTAG TTCGGATCTT CAATCTTCTC
LPCoLN ATTTTTCGAG AGAAACTTTA TCGAAAGTTG AAAAAATTAG TTCGGATCTT CAATCTTCTC
2040.3 ATTTTTCGAG AGAAACTTTA TCGAAAGTTG AAAAAATTAG TTCGGATCTT CAATCTTCTC
DE177 ATTTTTCGAG AGAAACTTTA TCGAAAGTTG AAAAAATTAG TTCGGATCTT CAATCTTCTC
N16 ATTTTTCGAG AGAAACTTTA TCGAAAGTTG AAAAAATTAG TTCGGATCTT CAATCTGCTC
130 140 150 160 170 180
Identity
B10 ATTTTTTCAT TTGTGATAAA AATTGTTCAG AAATCAATGC TTTAATCAAT CAAGCAACAG
B26 ATTTTTTCAT TTGTGATAAA AATTGTTCAG AAATCAATGC TTTAATCAAT CAAGCAACAG
B37 ATTTTTTCAT TTGTGATAAA AATTGTTCAG AAATCAATGC TTTAATCAAT CAAGCAACAG
EBB ATTTTTTCAT TTGTGATAAA AATTGTTCAG AAATCAATGC TTTAATCAAT CAAGCAACAG
LPCoLN ATTTTTTCAT TTGTGATAAA AATTGTTCAG AAATCAATGC TTTAATCAAT CAAGCAACAG
2040.3 ATTTTTTCAT TTGTGATAAA AATTGTTCAG AAATCAATGC TTTAATCAAT CAAGCAACAG
DE177 ATTTTTTCAT TTGTGATAAA AATTGTTCAG AAATCAATGC TTTAATCAAT CAAGCAACAG
N16 ATTTTTTCAT TTGTGATAAA AATTGTTCAG AAATCAATGC TTTAATCAAT CAAGCAACAG

	190	200	210	220	230	240
Identity						
B10	CCCTTAAACA	TTCCTATGGT	ATAGATATTT	TATTCATAGA	CTATTTACAG	CTCATAGAAG
B26	CCCTTAAACA	TTCCTATGGT	ATAGATATTT	TATTCATAGA	CTATTTACAG	CTCATAGAAG
B37	CCCTTAAACA	TTCCTATGGT	ATAGATATTT	TATTCATAGA	CTATTTACAG	CTCATAGAAG
EBB	CCCTTAAACA	TTCCTATGGT	ATAGATATTT	TATTCATAGA	CTATTTACAG	CTCATAGAAG
LPCoLN	CCCTTAAACA	TTCCTATGGT	ATAGATATTT	TATTCATAGA	CTATTTACAG	CTCATAGAAG
2040.3	CCCTTAAACA	TTCCTATGGT	ATAGATATTT	TATTCATAGA	CTATTTACAG	CTCATAGAAG
DE177	CCCTTAAACA	TTCCTATGGT	ATAGATATTT	TATTCATAGA	CTATTTACAG	CTCATAGAAG
N16	CCCTTAAACA	TTCCTATGGT	ATAGATATTT	TATTCATAGA	CTATTTACAG	CTCATAGAAG

	250	260	270	280	290	300
Identity						
B10	CAAATGGACG	CTCTGAAAAC	AGACAAAATG	AAATAGCATC	AATTTCAAGA	AAACTACGTA
B26	CAAATGGACG	CTCTGAAAAC	AGACAAAATG	AAATAGCATC	AATTTCAAGA	AAACTACGTA
B37	CAAATGGACG	CTCTGAAAAC	AGACAAAATG	AAATAGCATC	AATTTCAAGA	AAACTACGTA
EBB	CAAATGGACG	CTCTGAAAAC	AGACAAAATG	AAATAGCATC	AATTTCAAGA	AAACTACGTA
LPCoLN	CAAATGGACG	CTCTGAAAAC	AGACAAAATG	AAATAGCATC	AATTTCAAGA	AAACTACGTA
2040.3	CAAATGGACG	CTCTGAAAAC	AGACAAAATG	AAATAGCATC	AATTTCAAGA	AAACTACGTA
DE177	CAAATGGACG	CTCTGAAAAC	AGACAAAATG	AAATAGCATC	AATTTCAAGA	AAACTACGTA
N16	CAAATGGACG	CTCTGAAAAC	AGACAAAATG	AAATAGCATC	AATTTCAAGA	AAACTACGTA

	310	320	330	340	350	359
Identity						
B10	TGTTGTCCGT	AGATCTTGAA	ATACCAATAG	TTTGTTTATC	ACAGTTATCT	AGAAAGGTT
B26	TGTTGTCCGT	AGATCTTGAA	ATACCAATAG	TTTGTTTATC	ACAGTTATCT	AGAAAGGTT
B37	TGTTGTCCGT	AGATCTTGAA	ATACCAATAG	TTTGTTTATC	ACAGTTATCT	AGAAAGGTT
EBB	TGTTGTCCGT	AGATCTTGAA	ATACCAATAG	TTTGTTTATC	ACAGTTATCT	AGAAAGGTT
LPCoLN	TGTTGTCCGT	AGATCTTGAA	ATACCAATAG	TTTGTTTATC	ACAGTTATCT	AGAAAGGTT
2040.3	TGTTGTCCGT	AGATCTTGAA	ATACCAATAG	TTTGTTTATC	ACAGTTATCT	AGAAAGGTT
DE177	TGTTGTCCGT	AGATCTTGAA	ATACCTATAG	TTTGTTTATC	ACAGTTATCT	AGAAAGGTT
N16	TGTTGTCCGT	AGATCTTGAA	ATACCAATAG	TTTGTTTATC	ACAGTTATCT	AGAAAGGTT

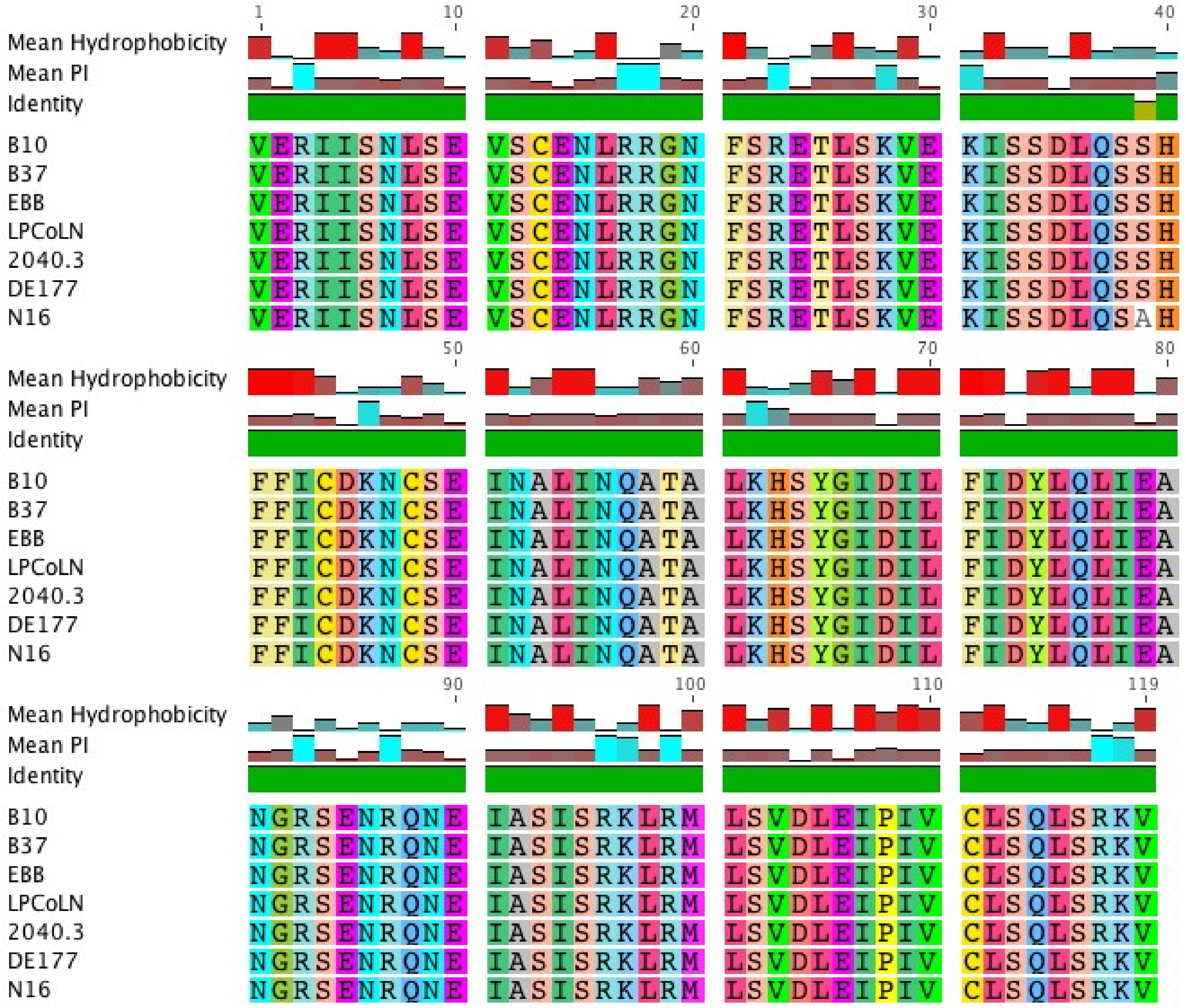
1 10 20 30 40
Mean Hydrophobicity
Mean PI
Identity
B10 VERIISNLSE VSCENLRRGN FSRETLSKVE KISSDLQSSH
B37 VERIISNLSE VSCENLRRGN FSRETLSKVE KISSDLQSSH
EBB VERIISNLSE VSCENLRRGN FSRETLSKVE KISSDLQSSH
LPCoLN VERIISNLSE VSCENLRRGN FSRETLSKVE KISSDLQSSH
2040.3 VERIISNLSE VSCENLRRGN FSRETLSKVE KISSDLQSSH
DE177 VERIISNLSE VSCENLRRGN FSRETLSKVE KISSDLQSSH
N16 VERIISNLSE VSCENLRRGN FSRETLSKVE KISSDLQSAH
50 60 70 80
Mean Hydrophobicity
Mean PI
Identity
B10 FFICDKNCSE INALINQATA LKHSYGIDIL FIDYLQLIEA
B37 FFICDKNCSE INALINQATA LKHSYGIDIL FIDYLQLIEA
EBB FFICDKNCSE INALINQATA LKHSYGIDIL FIDYLQLIEA
LPCoLN FFICDKNCSE INALINQATA LKHSYGIDIL FIDYLQLIEA
2040.3 FFICDKNCSE INALINQATA LKHSYGIDIL FIDYLQLIEA
DE177 FFICDKNCSE INALINQATA LKHSYGIDIL FIDYLQLIEA
N16 FFICDKNCSE INALINQATA LKHSYGIDIL FIDYLQLIEA
90 100 110 119
Mean Hydrophobicity
Mean PI
Identity
B10 NGRSENRQNE IASISRKLRM LSVDLEIPIV CLSQLSRKV
B37 NGRSENRQNE IASISRKLRM LSVDLEIPIV CLSQLSRKV
EBB NGRSENRQNE IASISRKLRM LSVDLEIPIV CLSQLSRKV
LPCoLN NGRSENRQNE IASISRKLRM LSVDLEIPIV CLSQLSRKV
2040.3 NGRSENRQNE IASISRKLRM LSVDLEIPIV CLSQLSRKV
DE177 NGRSENRQNE IASISRKLRM LSVDLEIPIV CLSQLSRKV
N16 NGRSENRQNE IASISRKLRM LSVDLEIPIV CLSQLSRKV
