# Supplementary material for: Chlamydia pneumoniae Is Genetically Diverse in Animals and Appears to Have Crossed the Host Barrier to Humans on (At Least) Two Occasions
Source: PLoS Pathog. 2010 May 20;6(5):e1000903. doi: 10.1371/journal.ppat.1000903 (PMC2873915; doi:10.1371/journal.ppat.1000903)
Supplement: Figure S23 — Multiple sequence alignment of PGP3D. The nucleotide and amino acid alignments were generated using Geneious version 4.7, where each nucleotide and amino acid is assigned its own colour. White shading indicates an amino acid variant. (0.47 MB PDF) [file ppat.1000903.s023.pdf]

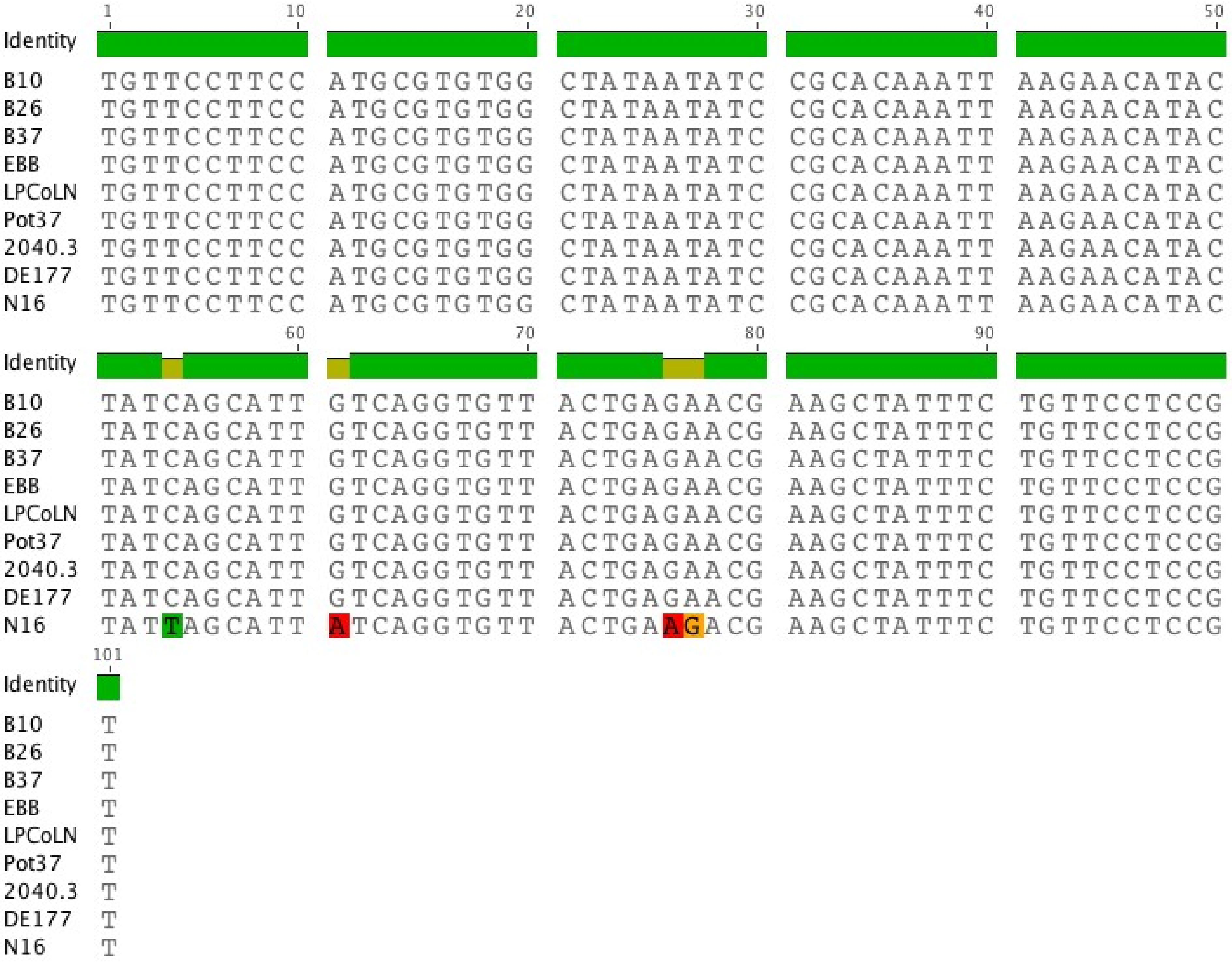

1 10 20 30 40 50
Identity
B10 TGTTCCTTCC ATGCGTGTGG CTATAATATC CGCACAAATT AAGAACATAC
B26 TGTTCCTTCC ATGCGTGTGG CTATAATATC CGCACAAATT AAGAACATAC
B37 TGTTCCTTCC ATGCGTGTGG CTATAATATC CGCACAAATT AAGAACATAC
EBB TGTTCCTTCC ATGCGTGTGG CTATAATATC CGCACAAATT AAGAACATAC
LPCoLN TGTTCCTTCC ATGCGTGTGG CTATAATATC CGCACAAATT AAGAACATAC
Pot37 TGTTCCTTCC ATGCGTGTGG CTATAATATC CGCACAAATT AAGAACATAC
2040.3 TGTTCCTTCC ATGCGTGTGG CTATAATATC CGCACAAATT AAGAACATAC
DE177 TGTTCCTTCC ATGCGTGTGG CTATAATATC CGCACAAATT AAGAACATAC
N16 TGTTCCTTCC ATGCGTGTGG CTATAATATC CGCACAAATT AAGAACATAC
60 70 80 90
Identity
B10 TATCAGCATT GTCAGGTGTT ACTGAGAACG AAGCTATTTC TGTTCCTCCG
B26 TATCAGCATT GTCAGGTGTT ACTGAGAACG AAGCTATTTC TGTTCCTCCG
B37 TATCAGCATT GTCAGGTGTT ACTGAGAACG AAGCTATTTC TGTTCCTCCG
EBB TATCAGCATT GTCAGGTGTT ACTGAGAACG AAGCTATTTC TGTTCCTCCG
LPCoLN TATCAGCATT GTCAGGTGTT ACTGAGAACG AAGCTATTTC TGTTCCTCCG
Pot37 TATCAGCATT GTCAGGTGTT ACTGAGAACG AAGCTATTTC TGTTCCTCCG
2040.3 TATCAGCATT GTCAGGTGTT ACTGAGAACG AAGCTATTTC TGTTCCTCCG
DE177 TATCAGCATT GTCAGGTGTT ACTGAGAACG AAGCTATTTC TGTTCCTCCG
N16 TATTAGCATT ATCAGGTGTT ACTGAAGACG AAGCTATTTC TGTTCCTCCG
101
Identity
B10 T
B26 T
B37 T
EBB T
LPCoLN T
Pot37 T
2040.3 T
DE177 T
N16 T

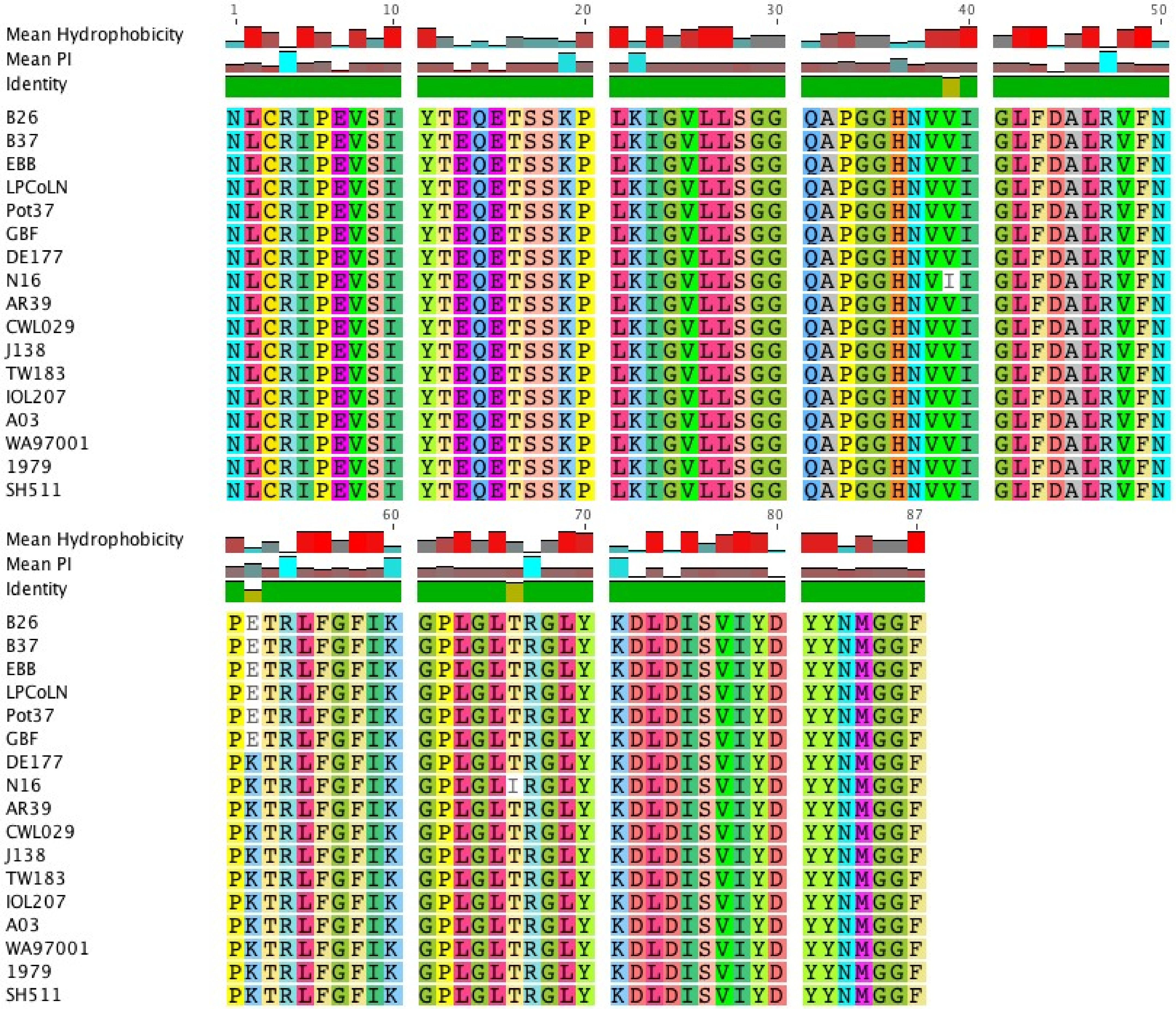
1 10 20 30 40 50
Mean Hydrophobicity
Mean PI
Identity
B26 NLCRIPEVSI YTEQETSSKP LKIGVLLSGG QAPGGHNVVI GLFDALRVFN
B37 NLCRIPEVSI YTEQETSSKP LKIGVLLSGG QAPGGHNVVI GLFDALRVFN
EBB NLCRIPEVSI YTEQETSSKP LKIGVLLSGG QAPGGHNVVI GLFDALRVFN
LPCoLN NLCRIPEVSI YTEQETSSKP LKIGVLLSGG QAPGGHNVVI GLFDALRVFN
Pot37 NLCRIPEVSI YTEQETSSKP LKIGVLLSGG QAPGGHNVVI GLFDALRVFN
GBF NLCRIPEVSI YTEQETSSKP LKIGVLLSGG QAPGGHNVVI GLFDALRVFN
DE177 NLCRIPEVSI YTEQETSSKP LKIGVLLSGG QAPGGHNVVI GLFDALRVFN
N16 NLCRIPEVSI YTEQETSSKP LKIGVLLSGG QAPGGHNVII GLFDALRVFN
AR39 NLCRIPEVSI YTEQETSSKP LKIGVLLSGG QAPGGHNVVI GLFDALRVFN
CWL029 NLCRIPEVSI YTEQETSSKP LKIGVLLSGG QAPGGHNVVI GLFDALRVFN
J138 NLCRIPEVSI YTEQETSSKP LKIGVLLSGG QAPGGHNVVI GLFDALRVFN
TW183 NLCRIPEVSI YTEQETSSKP LKIGVLLSGG QAPGGHNVVI GLFDALRVFN
IOL207 NLCRIPEVSI YTEQETSSKP LKIGVLLSGG QAPGGHNVVI GLFDALRVFN
A03 NLCRIPEVSI YTEQETSSKP LKIGVLLSGG QAPGGHNVVI GLFDALRVFN
WA97001 NLCRIPEVSI YTEQETSSKP LKIGVLLSGG QAPGGHNVVI GLFDALRVFN
1979 NLCRIPEVSI YTEQETSSKP LKIGVLLSGG QAPGGHNVVI GLFDALRVFN
SH511 NLCRIPEVSI YTEQETSSKP LKIGVLLSGG QAPGGHNVVI GLFDALRVFN
60 70 80 87
Mean Hydrophobicity
Mean PI
Identity
B26 PETRLFGFIK GPLGLTRGLY KDLDISVIYD YYNMGGF
B37 PETRLFGFIK GPLGLTRGLY KDLDISVIYD YYNMGGF
EBB PETRLFGFIK GPLGLTRGLY KDLDISVIYD YYNMGGF
LPCoLN PETRLFGFIK GPLGLTRGLY KDLDISVIYD YYNMGGF
Pot37 PETRLFGFIK GPLGLTRGLY KDLDISVIYD YYNMGGF
GBF PETRLFGFIK GPLGLTRGLY KDLDISVIYD YYNMGGF
DE177 PKTRLFGFIK GPLGLTRGLY KDLDISVIYD YYNMGGF
N16 PKTRLFGFIK GPLGLIRGLY KDLDISVIYD YYNMGGF
AR39 PKTRLFGFIK GPLGLTRGLY KDLDISVIYD YYNMGGF
CWL029 PKTRLFGFIK GPLGLTRGLY KDLDISVIYD YYNMGGF
J138 PKTRLFGFIK GPLGLTRGLY KDLDISVIYD YYNMGGF
TW183 PKTRLFGFIK GPLGLTRGLY KDLDISVIYD YYNMGGF
IOL207 PKTRLFGFIK GPLGLTRGLY KDLDISVIYD YYNMGGF
A03 PKTRLFGFIK GPLGLTRGLY KDLDISVIYD YYNMGGF
WA97001 PKTRLFGFIK GPLGLTRGLY KDLDISVIYD YYNMGGF
1979 PKTRLFGFIK GPLGLTRGLY KDLDISVIYD YYNMGGF
SH511 PKTRLFGFIK GPLGLTRGLY KDLDISVIYD YYNMGGF
