# Supplementary material for: Chlamydia pneumoniae Is Genetically Diverse in Animals and Appears to Have Crossed the Host Barrier to Humans on (At Least) Two Occasions
Source: PLoS Pathog. 2010 May 20;6(5):e1000903. doi: 10.1371/journal.ppat.1000903 (PMC2873915; doi:10.1371/journal.ppat.1000903)
Supplement: Table S1 — Chlamydia pneumoniae isolates used in this study. Complete list of C. pneumoniae isolates and description of their natural host, year of isolation, specimen type and reference. NA, information not available. (0.05 MB DOC) [file ppat.1000903.s024.doc]

Table S1. *Chlamydia pneumoniae* isolates used in this study

| Isolate | Host | Location / Year  of isolation if known | Specimen type | Reference |
| --- | --- | --- | --- | --- |
| AR39 | Human | United States of America, 1983 | Pharyngeal | [14] |
| CWL029 | Human | United States of America, 1987 | Oropharyngeal | [15] |
| J138 | Human | Japan, 1994 | Pharyngeal | [16] |
| TW183 | Human | Taiwan, 1965 | Conjunctival | [17] |
| IOL207 | Human | Iran, 1967 | Conjunctival | [39] |
| WA97001 | Human | Australia, 2001 | Nasopharyngeal | [40] |
| TOR1 | Human | Canada, NA | Brain | [41] |
| A03 | Human | United States of America, NA | Coronary atheroma tissue | [42] |
| LKK1 | Human | Korea, NA | Pharyngeal | [43] |
| SH511 | Human | Australia, 1992 | Nasopharyngeal | [44] |
| 1979 | Human | Australia, 1992 | Nasopharyngeal | [45] |
| LPCoLN | Marsupial -Koala | Australia, 1998 | Nasal | [6,22] |
| EBB | Marsupial - Koala | Australia, 2007 | Pharyngeal | This study |
| B10; B26; B37; WBB | Marsupial –Bandicoot | Australia, 2000 | Nasal | [46] |
| Pot37 | Marsupial – Potoroo | Australia, 2007 | Pharyngeal | This study |
| N16 | Equine – Horse | United Kingdom, 1989 | Nasal | [2] |
| DE177 | Amphibian – Frog | Central African Republic, NA | Liver | [5] |
| GBF | Amphibian – Frog | Australia, 1997 | Lung | [4] |
| 2040.3 | Amphibian – Frog | Switzerland, NA | Paraffin: lung, heart, liver | [47] |
| CPXT1 | Amphibian – Frog | Western Africa, 1998 | Liver | [28] |
| BMTF- type 1 | Amphibian – Frog | Australia, NA | Paraffin: lung, gastrointestinal and brain | [3] |
| BMTF- type 2 | Amphibian – Frog | Australia, NA | Paraffin: lung, gastrointestinal and brain | [3] |
| Pufadd | Reptile – Snake | United States of America, NA | Paraffin: respiratory tract and heart | [3] |
| Burpyth | Reptile – Snake | United States of America, NA | Lung | [3] |
| GST | Reptile – Turtle | United States of America, 1990 | Heart | [3] |
| Cham | Reptile – Lizard | Tanzania, NA | Paraffin: Spleen and Liver | [3] |
| Iguana | Reptile – Lizard | Central America, NA | Paraffin: liver, lung, spleen, stomach, intestine | [3] |

NA, not available
