# Supplementary material for: Chlamydia pneumoniae Is Genetically Diverse in Animals and Appears to Have Crossed the Host Barrier to Humans on (At Least) Two Occasions
Source: PLoS Pathog. 2010 May 20;6(5):e1000903. doi: 10.1371/journal.ppat.1000903 (PMC2873915; doi:10.1371/journal.ppat.1000903)
Supplement: Table S3 — Oligonucleotide primers used in this study. The fragment sizes are estimated from koala LPCoLN and human AR39 sequences. * Designed using Primer3 v.0.4.0. (0.06 MB DOC) [file ppat.1000903.s026.doc]

Table S3. Oligonucleotide primers used in this study

| Gene name^a^ | Primer name | Sequence (5’🡪3’) | Koala LPCoLN Nucleotide position | Human AR39 Nucleotide position | Primer annealing  temperature (°C) | Predicted  length (bp) | Reference |
| --- | --- | --- | --- | --- | --- | --- | --- |
| CPK_ORF00679* | K_00679F  K_00679R  K_00679HF^b^ | TTGTTTTATTGGGTGCTTTGC  TTGAGCTAAGGTCGAGGGAAG  TCGAAAAGGCGATTGTATATTG | 23-43  802-822  269-290 | NA  520-540  2-23 | 60  60 | 800  539 | This study  This study |
| MACPF* | MACaF  MACaR  MACbF  MACbR  MACupstreamF  MACupstreamR | GCAACCCAATTGTGTGATTC  TTCTCTAACTCCTCGACTTGG  TGGCATTGGTTTTCAAGTGC  CTCGTCTGTGTCGTGCAAGT  TCAAGCCGAAGAAAGAGAAGA  TCTGCGCACTATAAGGAACG | 6-25  1,551-1,571  1,515-1,534  2,410-2,429  NA  723-742 | 6-25  711-731  675-694  1,570-1,589  NA | 60  60  60 | 1,556 / 726  915  845 | This study  This study  This study |
| AroAA-HS | Cpn1046F  Cpn1046R | CACCGTGCACTACTGCGAGAGAACCC  CTATTGGCAAAGTACCTCAAAACC | NA  787-810 | 1-22  1,067-1,089 | 60 | 810 / 1,093 | [23] |
| *pmp*E/F2* | pmpEF2aF  pmpEF2aR  pmpEF2bF  pmpEF2bR | GTGTCAAAGACTCCTCCTAAGTT  AGGAGCTTTTCCTTTTGCTA GCAACAACTGCCAACTCTGA  TATTGTGAGCCGAGACGTTG | 1-23  1,535-1,554  1,447-1,466  2,223-2,242 | 1-23  1,535-1,554  1,447-1,466  2,223-2,242 | 56  60 | 1,554  795 | This study  This study |
| *pmp*E/F3* | pmpEF3aF  pmpEF3aR  pmpEF3bF  pmpEF3bR | CGACCCATTTGTCTCAGCAT  TGTTCCGGTACTGTAGAGCTTG  TGGTCTCCCTATTGGATGGA  CGGCTTTCAGCTTTCAGGTA | 4-23  1,838-1,859  1,804-1,823  2,818-2,837 | 55-74  1,889-1,910  1,855-1,874  2,872-2,891 | 60  60 | 1,856  1,034 | This study  This study |
| *pmp*G6* | pmpG6F  pmpG6R | ACAGCTCGCTGACTGGAAAT  TGCCATCAAAGGTAAGAGTCG | 1,181-1,200  1,817-1,837 | 1,181-1,200  1,424-1,444 | 60 | 657 / 264 | This study |
| 16S rRNA | 16SIGF  16SIGR | CGGCGTGGATGAGGCAT  TCAGTCCCAGTGTTGGC | 36-52  313-329 | 36-52  313-329 | 56 | 294 | [27] |
| *ompA** | ompaF  ompaR | CCCTTCTGATCCAAGCTTAT  TGAGCAGCTCTCTCGTTAAT | 84-103  1,126-1,145 | 84-103  1,126-1,145 | 56 | 1,062 | This study |
| *accC* | accCF  accCR | CTATAGAGCGTTTTCTGCCG  GTAATGGTATGCTCTACCTG | 528-547  876-896 | 528-547  876-896 | 56 | 369 | This study |
| *pfk* | pfkF  pfkR | AACTTCGTCTCCCCTTTCTCCCC  CGAACCCTCCCATGTTGTAA | 108-130  399-418 | 108-130  399-418 | 56 | 311 | This study |
| CP1042* | CP1042F  CP1042R | CAACTTTGGCGAAATCCT  GGCAACATAAGCACAGAAAA | NA | 5-22  508-527 | 56 | 523 | This study |
| CP0880* | CP0880F  CP0880R | GTCCTTATTGCTTTGCTAATCC  GCAATTTTGGTGGAGAAAA | 211-232  1,066-1,084 | 208-229  1,057-1,075 | 56 | 874 / 868 | This study |
| CP0505* | CP0505F  CP0505R | CAAATCCTACACCGAAAACA  CAAGGCAACTATGTTCCAAG | 5-24  836-855 | 5-24  836-855 | 56 | 855 | This study |
| SctC* | SctCF  SctCR | TAAAAATTCAGCAGCCTCAC  ACTGTTGTTGCTGCTTTCTC | 192-211  676-695 | 192-211  676-695 | 56 | 504 | This study |
| HAF* | HAFaF  HAFaR  HAFbF  HAFbR | GGCACGATTATTGTTGGGTCT  ATAAAGCCCGGCACCAAG  AGCCTCAGATCATGAGTTCACA  TCCATGGAGTAAGGACTTTCA | 601-624  1,240-1,257  1,194-1,215  2,114-2,134 | 601-624  1,120-1,237  1,074-1,120  1,994-2,014 | 60  60 | 657 / 537  941 | This study  This study |
| *guaB* | guaBF  guaBR | TTCTAGTCATTGACACAGCT  GCTCTTCCAGATTCAGTAAT | NA | 344-363  994-1,013 | 56 | 670 | This study |
| *guaA** | guaAF  guaAR | GAGTGCAAGGAGACATTTGA  CTATAGTTGCTGGTGGCTTG | NA | 6-25  1,260-1,279 | 56 | 1,274 | This study |
| *add** | addF  addR | GAATCTTGAAAAGGAAGATTCTG  ATCCTAACTGGCAAGTAACTCTT | NA | 24-46  981-1,003 | 56 | 1,005 | This study |
| CPK_ORF00678* | K_00678F  K_00678R | TTTCCCCCTACATAAAGCTGTC  CAGTAAATCCTCAGGCCATCA | 17-38  897-917 | NA | 60 | 901 | This study |
| SSR2 | SSR2F  SSR2R | CTACGCTTCTGGGATTAAG  CGAAGAGAGATAACTTCTG | 102-120 | NA | 54 | 439 | This study |
| Helicase | HelicaseF  HelicaseR | CTGCTATGGGTAAAACAGC  GCATCTTGCTCTATTTGACC | 674-692 | NA | 57 | 538 | This study |
| PGP3D | PGP3DF  PGP3DR | ACGGAGGAACAGAAATAGC  GCATTTACCCACACAACACC | 440-458 | NA | 57 | 336 | This study |

^a^Gene name as annotated in this study

^b^Primer sequence designed based on human AR39 sequence CP_0608

* Primer sequence designed using Primer 3

NA, not available
