# Supplementary material for: Chlamydia pneumoniae Is Genetically Diverse in Animals and Appears to Have Crossed the Host Barrier to Humans on (At Least) Two Occasions
Source: PLoS Pathog. 2010 May 20;6(5):e1000903. doi: 10.1371/journal.ppat.1000903 (PMC2873915; doi:10.1371/journal.ppat.1000903)
Supplement: Text S1 — Key genomic data supporting two evolutionary lineages. (0.03 MB DOC) [file ppat.1000903.s027.doc]

(i) The two Australian Indigenous human isolates (SH511 and 1979) have an extended 251 bp segment at the upstream region of CPK_ORF00679. This extended sequence is identical to the bandicoot (B26), koala (LPCoLN) and frog (DE177) isolates, yet all non-Indigenous human isolates lack this segment (Figure S2). The two Australian Indigenous human isolates, as well as frog DE177, bandicoot B26, koala LPCoLN and horse N16, all have the indel IADRF (positions 244-248) which is absent from the non-Indigenous human isolates (Figure 1).

(ii) The *pmp*E/F2 family protein gene is another example where the Australian Indigenous human isolates have several polymorphisms in common with the animal isolates; both Australian Indigenous human isolates (SH511 and 1979), one frog (DE177), one bandicoot (B26) and one koala (LPCoLN) isolate have a V at position 8, an S at position 298, and an R at position 323 of the amino acid alignment, whereas all non-Indigenous human isolates have the residues, LFL at these positions (Figure S5). Interestingly, the Australian Indigenous human isolates SH511 and 1979 share more sequence identity to the frog DE177 isolate and have the profile VQTDSLEKF at positions 8-16, whereas the koala and bandicoot isolates have the profile VQTNSLEKS at these positions (Figure S5).

(iii) The well-studied 16S rRNA gene also provides evolutionary clues (Figure S7). Both Australian Indigenous human isolates (SH511 and 1979) have the SNP profile AA at positions 21 and 51, matching four amphibian ‘frog’ isolates (DE177, CPXT1, 2040.3 and GBF), and seven marsupial isolates (bandicoot B10, bandicoot B26, bandicoot B37, bandicoot WBB, koala EBB, koala LPCoLN, and potoroo Pot37), whereas the eight non-Indigenous human isolates (AR39, CWL029, TW183, J138, TOR1, WA97001, LKK1, and IOL207), two amphibians ‘frogs’ (BMTF-type 1 and BMTF-type 2), and two reptiles ‘snakes’ (Pufadd and Iguana) have the profile GG at these positions. Overall these data suggest that; (i) the common human genotype (*C. pneumoniae*) evident by isolates AR39, CWL029, J138, TW183, LKK1, IOL207, TOR1 and WA97001, is apparently circulating in most continents, and probably originated from a cross species transmission event from an amphibian or reptile source (frogs, turtles and snakes); (ii) the lack of genetic variability within eight non-Indigenous human isolates and the minimal divergence observed is likely to be the result of a recent (last few centuries) cross species transfer event to humans; (iii) the Australian Indigenous human genotype evident in the two isolates SH511 and 1979, originated from a separate cross host transmission event from one of the animals (amphibian or marsupial); and, (iv) the Australian marsupial genotype also appears to have originated from a cross host transmission event from an amphibian. We suggest that the sampling of native populations from other geographic regions will be necessary to confirm these hypotheses.

(iv) The *ompA* gene has six characteristic SNPs (Figure S8): (i) six marsupial isolates (koala LPCoLN, koala EBB, bandicoot WBB, bandicoot B26, bandicoot B37, and Pot37), one frog (GBF) and one horse (N16) have a ‘T’ at nucleotide position 83, while the remaining 19 isolates have a ‘C’ at this position, (ii) six marsupial isolates (koala LPCoLN, koala EBB, koala WBB, bandicoot B26, bandicoot B37 and potoroo Pot37), and one frog (GBF) have an ‘A’ at position 225 and a ‘G’ at position 227, while the remaining 18 isolates have the profile ‘GC’ at these positions, (iii) six marsupial isolates (koala LPCoLN, koala EBB, koala WBB, koala B26, koala B37 and koala Pot37), and three frogs (GBF, CPXT1 and DE177) have an ‘A’ at position 231, while the remaining 16 isolates have a ‘G’ at this position, (iv) six marsupial isolates (koala LPCoLN, koala EBB, bandicoot WBB, bandicoot B26, bandicoot B37 and potoroo Pot37), and two frogs (GBF and DE177) have a ‘C’ at position 237, while the remaining 17 isolates have a ‘T’ at this position and, (v) six marsupial isolates (koala LPCoLN, koala EBB, bandicoot WBB, bandicoot B26, bandicoot B37 and potoroo Pot37) and one frog (GBF) have a ‘G’ at position 244, while two frogs (CPXT1 and DE177) have an ‘A’ at this position, and the remaining 16 isolates have a ‘C’ at this position. The *ompA* gene, along with 16S rRNA also provides evidence of two separate animal-to-human transmission events.

(v) The frog (DE177), horse (N16), koala (LPCoLN) and Australian Indigenous human isolates (SH511 and 1979) have a K at position 77 of the SctC type III secretion protein, and another K at position 91, whereas the non-Indigenous human isolates all have a T and Q at these positions (Figure S15).

(vi) The present or absent genes are another example of a closer association of the Australian Indigenous human isolates with frog DE177. For instance, the frog DE177 isolate contains all three *guaBA-add* genes (Figure S17-19), while the koala LPCoLN isolate contains none of them. Sequence comparisons with all eight available isolates revealed that the frog DE177, horse N16 and both Australian Indigenous human isolates SH511 and 1979 have a G at position 240, and an A at position 463 of *guaB*, whereas all six non-Indigenous isolates have AG at these positions (Figure S17).
